# Efficient Access to New Thienobenzo-1,2,3-Triazolium Salts as Preferred Dual Cholinesterase Inhibitors

**DOI:** 10.3390/biom14111391

**Published:** 2024-10-31

**Authors:** Milena Mlakić, Maja Sviben, Ana Ratković, Anamarija Raspudić, Danijela Barić, Ivana Šagud, Zlata Lasić, Ilijana Odak, Irena Škorić

**Affiliations:** 1 Department of Organic Chemistry, Faculty of Chemical Engineering and Technology, University of Zagreb, Trg Marka Marulića 19, HR-10 000 Zagreb, Croatia; mdragojev@fkit.unizg.hr (M.M.); mratajec@fkit.unizg.hr (M.S.); 2 Chemistry, Selvita Ltd., Prilaz Baruna Filipovića 29, HR-10 000 Zagreb, Croatia; ana.ratkovic@selvita.com; 3 Department of Chemistry, Faculty of Science and Education, University of Mostar, Matice hrvatske bb, 88 000 Mostar, Bosnia and Herzegovina; anamarijaraspudic1@gmail.com; 4 Group for Computational Life Sciences, Division of Physical Chemistry, Ruđer Bošković Institute, Bijenička cesta 54, HR-10 000 Zagreb, Croatia; dbaric@irb.hr; 5 Croatian Agency for Medicinal Products and Medical Devices, Ksaverska cesta 4, HR-10 000 Zagreb, Croatia; ivana.sagud@halmed.hr; 6 TEVA Global R&D, E&L R&D, Pliva Hrvatska d.o.o., Prilaz baruna Filipovića 25, HR-10 000 Zagreb, Croatia; zlata.lasic01@pliva.com

**Keywords:** cholinesterase inhibition, docking, genotoxicity, 1,2,3-triazolium salts

## Abstract

In previous research, 1,2,3-triazolium salts showed significant biological activity as potential inhibitors of cholinesterase enzymes (ChEs), which are crucial for neurotransmission. In this research, pairs of uncharged thienobenzo-triazoles and their charged salts were prepared in order to further examine the role of the positive charge on the nitrogen of the triazole ring in interactions within the active site of the enzymes, and to compare the selectivity of 1,2,3-triazolium salts in relation to their uncharged analogs obtained by photochemical cyclization. Neutral thienobenzo-triazoles showed very good selective activity toward butyrylcholinesterase (BChE), while their salts showed excellent non-selective inhibition toward both BChE (the most active **23**: IC_50_ 0.47 μM) and acetylcholinesterase (AChE) enzymes (the most active **23**: IC_50_ 4.4 μM). These new structures with incorporated 1,2,3-triazolium salts present the new scaffold for drug development as it is known that the current therapy in Alzheimer’s disease (AD) comprises selective AChE inhibitors, while in Parkinson’s and all stages of AD, non-selective inhibitors of ChEs are preferred. Molecular docking of the selected compounds and their corresponding salts into the active sites of ChEs was conducted to identify the interactions responsible for the stability of the non-covalent cholinesterase–ligand complexes. As genotoxicity studies are crucial when developing new active substances and finished drug forms, in silico studies for all the synthesized compounds have shown that compound **18** is the most promising candidate for genotoxic safety.

## 1. Introduction

Direct synthesis of 1,2,3-triazolium salts can occur in several steps, including CuAAC, *N*-alkylation, and salt metathesis. These methods can achieve a wide range of 1,2,3-triazole derivatives with different substituents and substitution patterns [1]. In addition to their impressive applications as ionic liquids, catalysts, and metal ligands, 1,2,3-triazolium salts exhibit a wide range of biological activities, such as antibacterial [2,3,4], antifungal [5], anticancer [6], and antileishmanial [7] properties. They are valuable in developing pesticides and pharmaceutical products [1]. Incorporating 1,2,3-triazole groups into polymeric materials via simple synthetic methods can generate antibacterial properties. The use of such materials can solve the problem of microbial contamination in medical devices or specialized antibacterial environments. The incorporation of 1,2,3-triazolium salts into anticancer drug carriers may bring additional opportunities. Modifying special nanotubes with triazolium salts can result in positively charged carriers of anticancer drugs with improved encapsulation efficiency, controlled release ability, increased water solubility, and thus improved anticancer activity. Based on the structure–activity relationship of the 1,2,3-triazolium salt derivatives, the substituents at the N-1 and N-3 positions are believed to play key roles in biological activities. The lipophilicity/hydrophilicity balance of 1,2,3-triazolium salts is crucial for their high biological activity and selectivity. In addition, 1,2,3-triazole derivatives can have multiple modes of action, and electrostatic interactions with anionic components on the cell membrane are among the most important.

Compared with neutral triazoles, 1,2,3-triazolium salts have superior biological activities and water solubility and are promising in developing new drugs for drug-resistant pathogens. They possess unique chemical properties, such as high stability, tunable reactivity, and electronic properties [8]. They are valuable building blocks in organic synthesis and have also been used to develop new catalysts and fluorescent dyes [9]. These salts have three active sites, including the N-1, N-3, and C-4 positions, which can bind to different functional groups and thus regulate their biological activities and cytotoxicity.

Numerous examples of 1,2,3-triazole derivatives that act as inhibitors of cholinesterase enzymes (ChEs), inspired by tacrine and acridones, can be found in the literature [10,11,12,13,14,15]. These are molecules with a substituted benzyl group on the 1,2,3-triazole ring. The structure–activity relationship (SAR) showed that by replacing hydrogen with chlorine in the *para*-position of the benzyl ring, the inhibitory activity against acetylcholinesterase (AChE) is improved [11]. Similarly, the structure–activity relationship showed that the methoxy group in the *para*-position of the benzyl ring has the best inhibitory activity, while chlorine, fluorine, methyl, and the unsubstituted benzyl ring decrease the activity [12]. Since it has a large dipole moment and can form hydrogen bonds with active sites, benzyl-1,2,3-triazole was also used for molecular hybridization with methylindolinone [15]. The synthesized compound showed good to moderate activity towards butyrylcholinesterase (BChE), but weak inhibition of AChE. The IC_50_ value for BChE was better than that of AChE inhibitor donepezil.

**Figure 1:**
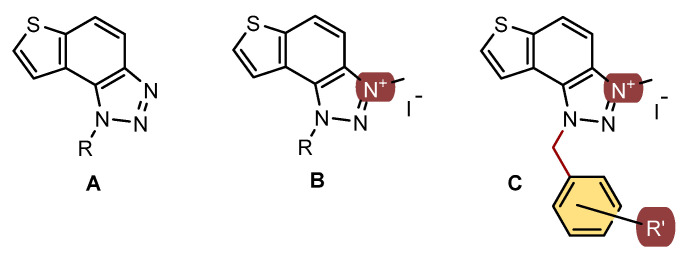
Structures of previously evaluated thienobenzo-triazoles **A** and triazolium salts **B**, and new triazolium salts **C** with the substituted benzyl group on the charged 1,2,3-triazole ring in this study.

In our previous study, different thienobenzo-triazoles **A** and triazolium salts **B** possessing various substituents on the triazole ring (Figure 1) were synthesized [16,17,18,19,20] and spectroscopically and biologically evaluated. They exhibited the capability to bind to proteins, displaying diverse inhibition properties towards AChE and BChE. Triazolium salts **B** exhibited excellent inhibition and demonstrated very good non-selective inhibition of both BChE and AChE enzymes. In contrast, neutral thienobenzo-triazoles **A** were shown to be selective towards BChE, even with very good inhibition potential.

Additionally, the representatives of those salts remained stable in an aqueous solution and displayed intriguing fluorimetric properties characterized by strong Stokes shifts exceeding 160 nm [20]. The molecular docking of the best-performing candidates to cholinesterases’ active site identified cation–π and π–π interactions as responsible for the stability of the enzyme–ligand complexes. Regarding the safety of triazolium salts **B**, no structural alerts were found by the Derek Nexus considering potential genotoxicity, so they could be considered the safest choices for further development into drug formulations, particularly given their high inhibitory potential against AChE and BChE.

In this study, the second series of triazolium salts **C** (Figure 1) with the substituted benzyl group on the charged 1,2,3-triazole ring were designed and biologically evaluated as cholinesterase inhibitors. These structures present the new scaffold of 1,2,3-triazolium salts for drug development as the current therapy in Alzheimer’s disease (AD) are selective AChE inhibitors, while in Parkinson’s and all stages of AD, non-selective inhibitors of ChE are preferred.

## 2. Materials and Methods

### 2.1. General Procedure

^1^H NMR and ^13^C NMR (CDCl_3_) techniques were used to confirm the structure of the synthesized compounds, and the spectra were recorded on a Bruker Avance instrument at 300 and 600 MHz for ^1^H NMR and at 75 and 150 MHz for ^13^C NMR. Chemical shifts (*δ*) are expressed in parts per million values (ppm) and coupling constants (*J*) in Hz. As a standard, tetramethylsilane (TMS) was used. The following signals in the ^1^H NMR spectra were specific and did not correspond to compounds: the signal for water in chloroform at about 1.50 ppm; the signal for chloroform at 7.24 ppm; the signal for dichloromethane in chloroform at 5.26 ppm; and the signal for acetone in chloroform at 2.17 ppm. Each ^13^C NMR spectrum contained one specific signal (group of three peak lines) at 77 ppm corresponding to the solvent-deuterated chloroform used. Triazolium salts **23**, **25**, **26**, **30** and **31** were also recorded in CD_3_OD to solve the problem of relatively low solubility. High-resolution mass spectrometry (HRMS) analyses were performed on a MALDI TOF/TOF analyzer mass spectrometer fitted with an Nd:YAG laser at 355 nm (fitting rate of 200 Hz). Photochemical reactions were carried out in a 50.0 mL solution in quartz cuvettes that transmitted light. For this purpose, a Rayonet photochemical reactor equipped with UV lamps (10) with a wavelength of 313 nm was used. All solvents used in this work were purified by distillation and were commercially available. The phosphonium salts were synthesized in our laboratory, and 1-(4-nitrophenyl)-1*H*-1,2,3-triazole-4-carbaldehyde used was previously synthesized in our laboratory [21]. After each Wittig reaction, an extraction was performed three times, separating the organic and aqueous layers.

The organic layer was dried over anhydrous magnesium sulfate, MgSO_4_. Thin-layer chromatography was performed on plates coated with silica gel (0.2 mm, 60/Kieselguhr F_254_) immersed in 10 mL of the dissolution system. Column chromatography was performed in glass columns of different diameters. The columns were filled with silica gel (60 Å, technical grade) of different heights. Abbreviations used in the experimental part of the work are the following: CAN—acetonitrile; DCM—dichloromethane; E—diethyl ether; EtOAc—ethyl acetate; PE—petroleum ether; NMR—nuclear magnetic resonance; UV—ultraviolet spectroscopy; NaOEt—sodium ethoxide; s—singlet; d—doublet; t—triplet; m—multiplet; dd—doublet of doublets; and q—quartet. UV/Vis spectra were recorded on a Varian Cary 100 Bio spectrometer and fluorescence spectra on a Varian Cary Eclipse fluorimeter in appropriate quartz cuvettes (path length = 1 cm).

### 2.2. Synthesis of Phosphonium Salts ***1′*** and ***2′***

In a 250 mL three-necked flask, 7.9 g (0.063 mol) of 2-thiophene methanol was dissolved in 72.3 mL of dry solvent diethyl ether and added to the flask. Then, 5.71 g (0.022 mol) of phosphorus tribromide (PBr_3_) was dissolved in 7.23 mL of dry diethyl ether and added dropwise to the reaction flask over 40 min. The reaction mixture was stirred at room temperature on a magnetic stirrer for 1 h. Subsequently, 15 mL of methanol and 100 mL of distilled water were added, and the mixture was extracted with a total of 300 mL of diethyl ether. The ether layer was dried overnight with anhydrous magnesium sulfate (MgSO_4_) filtered, and evaporated to dryness using a rotary evaporator, leaving 2-thiophene bromide as a yellow oil in the flask. This oil was then dissolved in 20 mL of toluene, while 16.52 g (0.037 mol) of triphenylphosphine (PPh_3_) was dissolved in 50 mL of toluene, and both solutions were added to the reaction flask. The mixture was stirred on a magnetic stirrer for three days and then filtered through a Büchner funnel under reduced pressure. The resulting light-yellow salt was dried in a desiccator for 12 h. The dry thiophene-phosphonium salt **1′** obtained was used in all subsequent experiments for synthesizing compounds **6**–**8**.

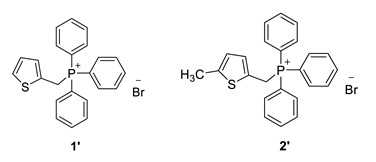



In a 250 mL round flask, 4.925 g (0.0440 mol) of dimethylthiophene was dissolved in 133 mL of carbon tetrachloride (CCl_4_). Then, 7.83 g (1 equivalent) of *N*-bromosuccinimide (NBS) was added to the mixture. The chemicals were stirred in an oil bath at a reflux temperature (150 °C). Once reflux started, a small amount of *α,α*-azobisisobutyronitrile (AIBN) catalyst was added. The oil bath temperature was then reduced to approximately 100 °C due to excessive reflux. With reflux established, the reaction mixture was illuminated with a lamp to initiate the reaction. After 1.5 h, bromide formation began, accompanied by the appearance of white, fluffy succinimide crystals on the surface. After another hour, a second dose of AIBN was added, and the mixture was refluxed for an additional 3 h. The succinimide was filtered out using pleated filter paper, and the clear orange liquid was evaporated using a rotary evaporator, leaving a brown, oily precipitate of thiophene bromide in the flask. In the second step, the thiophene bromide was dissolved in 10 mL of benzene with added PPh_3_. The mixture was heated to reflux (80 °C, with the mixer set to 110 °C) and stirred at that temperature for 1 h. The heat was then turned off, and the mixture was stirred at room temperature for the next 24 h. The resulting salt was filtered using a Büchner funnel, and the crystallizer was weighed before and after adding the salt. The 2-methylthiophene salt **2′** was dried in a desiccator under vacuum for 4–5 h and then used for synthesizing compounds **9**–**13**.

### 2.3. Synthesis of Triazole Aldehydes ***1**–**5***

Triazole aldehydes **1**–**5** were synthesized in small glass vials from 200 mg of 1-(4-nitrophenyl)-1*H*-1,2,3-triazole-4-carbaldehyde [21] dissolved in 2 mL of dry 1,4-dioxane. The 1.2 eq of appropriate benzylamine was then added to the reaction mixture, and the mixture was briefly purged with argon. Depending on the applied benzylamine, the reaction took place for a certain period of time (24–48 h). The course of the reaction was monitored by thin-layer chromatography. At the end of the reaction, the solvent was removed by evaporation on a rotary vacuum evaporator. The solid product was purified by column chromatography using an appropriate solvent system, mostly DCM/EtOAc.

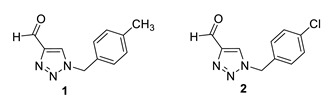



1-(4-methylbenzyl)-1*H*-1,2,3-triazole-4-carbaldehyde (**1**): 46 mg (isolated 23%), yellow oil; *Rf* (DCM) = 0.15; ^1^H NMR (CDCl_3_, 600 MHz) *δ*/ppm: 10.11 (s, 1H), 7.95 (s, 1H), 7.22 (d, *J =* 8.7 Hz, 2H), 7.20 (d, *J* = 8.7 Hz, 2H), 5.54 (s, 2H), 2.37 (s, 3H).

1-(4-chlorobenzyl)-1*H*-1,2,3-triazole-4-carbaldehyde (**2**): 131 mg (isolated 65%), yellow oil; *Rf* (DCM) = 0.40; ^1^H NMR (CDCl_3_, 300 MHz) *δ*/ppm: 10.13 (s, 1H), 8.01 (s, 1H), 7.39 (d, *J* = 7.4 Hz, 2H), 7.25 (d, *J* = 8.9 Hz, 2H), 5.57 (s, 2H).

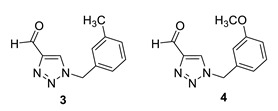



1-(3-methylbenzyl)-1*H*-1,2,3-triazole-4-carbaldehyde (**3**): 130 mg (isolated 65%), yellow oil; *Rf* (DCM) = 0.22; ^1^H NMR (CDCl_3_, 600 MHz) *δ*/ppm: 10.12 (s, 1H), 7.98 (s, 1H), 7.29 (t, *J* = 7.3 Hz, 1H), 7.21 (d, *J* = 8.1 Hz, 1H), 7.10 (d, *J* = 6.5 Hz, 2H), 5.54 (s, 2H), 2.36 (s, 3H).

1-(3-methoxybenzyl)-1*H*-1,2,3-triazole-4-carbaldehyde (**4**): 121 mg (isolated 61%), yellow oil; *Rf* (DCM) = 0.30; ^1^H NMR (CDCl_3_, 300 MHz) *δ*/ppm: 10.13 (s, 1H), 7.98 (s, 1H), 7.32 (t, *J* = 7.8 Hz, 1H), 6.93 (dd, *J* = 8.7, 2.6 Hz, 1H), 6.88 (d, *J* = 7.8 Hz, 1H), 6.82 (s, 1H), 5.55 (s, 2H), 3.80 (s, 3H).

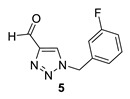



1-(3-fluorobenzyl)-1*H*-1,2,3-triazole-4-carbaldehyde (**5**): 163 mg (isolated 82%), yellow oil; *Rf* (DCM) = 0.42; ^1^H NMR (CDCl_3_, 600 MHz) *δ*/ppm: 10.14 (s, 1H), 8.03 (s, 1H), 7.41–7.37 (m, 1H), 7.11 (dd, *J* = 8.0, 2.4 Hz, 1H), 7.08 (d, *J* = 8.0 Hz, 1H), 7.00 (d, *J* = 8.8 Hz, 1H), 5.59 (s, 2H).

### 2.4. Synthesis of Triazolostilbenes ***6**–**13*** by Wittig Reaction

The apparatus, in a three-necked flask, a dropping funnel, a chlorine-calcium tube, and a balloon filled with nitrogen, was blown with argon for 5 min. A magnet was placed in the flask, the addition funnel was closed, and 30 mL of absolute ethanol was poured in (depending on the amount of starting reactants). A portion of absolute ethanol (10 mL) was poured into the flask, and the required amount of triphenylphosphonium salt was added. Sodium previously weighed in PE on an analytical balance with a precision of 0.0001 g was added to the remaining amount of absolute ethanol. After all the sodium had reacted in the ethanol with the evolution of hydrogen, a little portion of NaOEt was added to the flask. The aldehyde was dissolved in ethanol and transferred to a flask, and then the rest of the NaOEt from the funnel was added dropwise. The flask was closed with a glass stopper, and the reaction mixture was left to stir in a magnetic stirrer at room temperature (**6**—108 h, **7**—72 h, **8**—48 h, **9**—96 h, **10**—96 h, **11**—96 h, **12**—168 h, and **13**—120 h). Compounds **6**–**13** were synthesized as mixtures of isomers and their pure isomers were not isolated due to the next reaction step.

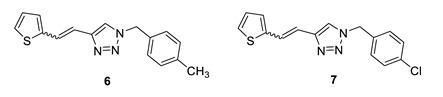



1-(4-methylbenzyl)-4-(2-(thiophen-2-yl)vinyl)-1*H*-1,2,3-triazole (**6**): 37 mg (mixture of isomers, *cis*: *trans* = 1: 1, 80%), white oil; *Rf* (PE/E = 80%) = 0.48.

1-(4-chlorobenzyl)-4-(2-(thiophen-2-yl)vinyl)-1*H*-1,2,3-triazole (**7**): 145 mg (mixture of isomers, *cis*: *trans* = 1: 0.6, 90%), white oil; *Rf* (PE/E = 60%) = 0.25. MS (ESI) *m*/*z* (%, fragment): 302 (100); HRMS (*m*/*z*) for C_15_H_12_ClN_3_S: [M + H]^+^_calcd_ = 302.0441, and [M + H]^+^_measured_ = 302.0440.

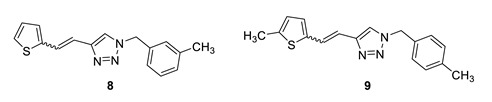



1-(3-methylbenzyl)-4-(2-(thiophen-2-yl)vinyl)-1*H*-1,2,3-triazole (**8**): 108 mg (mixture of isomers, *cis*: *trans* = 1: 0.8, 91%), white oil; *Rf* (PE/E = 80%) = 0.67.

1-(4-methylbenzyl)-4-(2-(5-methylthiophen-2-yl)vinyl)-1*H*-1,2,3-triazole (**9**): 13 mg (mixture of isomers, *cis*: *trans* = 1: 0.9, 13%), white oil; *Rf* (PE/E = 50%) = 0.60. MS (ESI) *m*/*z* (%, fragment): 296 (100); HRMS (*m*/*z*) for C_17_H_17_N_3_S: [M + H]^+^_calcd_ = 296.1143, and [M + H]^+^_measured_ = 296.1147.

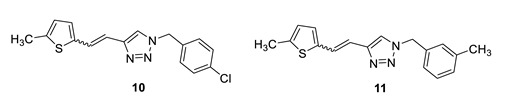



1-(4-chlorobenzyl)-4-(2-(5-methylthiophen-2-yl)vinyl)-1*H*-1,2,3-triazole (**10**): 50 mg (mixture of isomers, *cis*: *trans* = 1: 0.8, 32%), white oil; *Rf* (PE/E = 50%) = 0.50. MS (ESI) *m*/*z* (%, fragment): 316 (100); HRMS (*m*/*z*) for C_16_H_14_ClN_3_S: [M + H]^+^_calcd_ = 316.0597, and [M + H]^+^_measured_ = 316.0599.

1-(3-methylbenzyl)-4-(2-(5-methylthiophen-2-yl)vinyl)-1*H*-1,2,3-triazole (**11**): 12 mg (mixture of isomers, *cis*: *trans* = 1: 0.7, 10%), white oil; *Rf* (PE/E = 90%) = 0.58. MS (ESI) *m*/*z* (%, fragment): 296 (100); HRMS (*m*/*z*) for C_17_H_17_N_3_S: [M + H]^+^_calcd_ = 296.1143, and [M + H]^+^_measured_ = 296.1139.

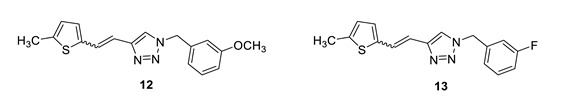



1-(3-methoxybenzyl)-4-(2-(5-methylthiophen-2-yl)vinyl)-1*H*-1,2,3-triazole (**12**): 35 mg (mixture of isomers, *cis*: *trans* = 1: 0.7, 24%), white oil; *Rf* (PE/E = 50%) = 0.50. MS (ESI) *m*/*z* (%, fragment): 312 (100); HRMS (*m*/*z*) for C_17_H_17_N_3_OS: [M + H]^+^_calcd_ = 312.1092, and [M + H]^+^_measured_ = 312.1098.

1-(3-fluorobenzyl)-4-(2-(5-methylthiophen-2-yl)vinyl)-1*H*-1,2,3-triazole (**13**): 16 mg (mixture of isomers, *cis*: *trans* = 1: 0.7, 23%), white oil; *Rf* (PE/E = 50%) = 0.54.

### 2.5. Synthesis of Photoproducts ***14**–**21***

The corresponding 1,2,3-triazolostilbenes **6**–**13**, as mixtures of isomers, were dissolved in 50 mL of toluene. These mixtures were then transferred to quartz tubes, which allowed light to pass through. A small amount of iodine was added as an oxidizing agent. The solutions, with a concentration of approximately 10^−3^ mol L^−1^, were exposed to light for 1–4 h using ten lamps emitting at a wavelength of about 313 nm in a Rayonet photoreactor. The resulting products, **14**–**21**, were purified through column chromatography. Spectroscopic data for photoproducts **14**–**21** are provided below.

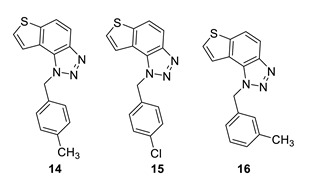



1-(4-methylbenzyl)-1*H*-thieno [3’,2’:3,4]benzo [1,2-*d*][1,2,3]triazole (**14**): 27 mg (isolated 73%), yellow oil; *Rf* (PE/E (40%)) = 0.34; ^1^H NMR (CDCl_3_, 600 MHz) *δ*/ppm: 7.99 (d, *J* = 9.0 Hz, 1H), 7.80 (d, *J* = 9.0 Hz, 1H), 7.55 (d, *J* = 6.0 Hz, 1H), 7.51 (d, *J* = 5.6 Hz, 1H), 7.12 (d, *J* = 8.2 Hz, 2H), 7.09 (d, *J* = 8.2 Hz, 2H), 6.08 (s, 2H), 2.29 (s, 3H); ^13^C NMR (CDCl_3_, 75 MHz) *δ*/ppm: 144.7, 140.0, 138.1, 132.1, 129.8, 128.7, 127.7, 126.5, 122.7, 120.3, 119.2, 116.1, 52.9, 21.1; MS (ESI) *m*/*z* (%, fragment): 280 (100); HRMS (*m*/*z*) for C_16_H_13_N_3_S: [M + H]^+^_calcd_ = 280.0830, and [M + H]^+^_measured_ = 280.0832.

1-(4-chlorobenzyl)-1*H*-thieno [3’,2’:3,4]benzo [1,2-*d*][1,2,3]triazole (**15**): 36 mg (isolated 25%), yellow oil; *Rf* (PE/E (80%)) = 0.25; ^1^H NMR (CDCl_3_, 600 MHz) *δ*/ppm: 8.00 (d, *J* = 8.6 Hz, 1H), 7.83 (d, *J* = 8.1 Hz, 1H), 7.58 (d, *J* = 5.4 Hz, 1H), 7.46 (d, *J* = 5.4 Hz, 1H), 7.29 (d, *J* = 8.6 Hz, 2H), 7.13 (d, *J* = 9.2 Hz, 2H), 6.09 (s, 2H); ^13^C NMR (CDCl_3_, 150 MHz) *δ*/ppm: 144.7, 140.2, 134.3, 133.6, 129.4, 128.6, 128.0, 127.9, 122.5, 119.9, 119.4, 116.1, 52.4; MS (ESI) *m*/*z* (%, fragment): 300 (100); HRMS (*m*/*z*) for C_15_H_10_ClN_3_S: [M + H]^+^_calcd_ = 300.0284, and [M + H]^+^_measured_ = 300.0283.

1-(3-methylbenzyl)-1*H*-thieno [3’,2’:3,4]benzo [1,2-*d*][1,2,3]triazole (**16**): 26 mg (isolated 24%), yellow oil; *Rf* (PE/E (40%)) = 0.35; ^1^H NMR (CDCl_3_, 600 MHz) *δ*/ppm: 8.00 (d, *J* = 8.9 Hz, 1H), 7.81 (d, *J* = 9.1 Hz, 1H), 7.55 (d, *J* = 5.9 Hz, 1H), 7.51 (d, *J* = 5.7 Hz, 1H), 7.20 (t, *J* = 8.0 Hz, 1H), 7.09 (d, *J* = 8.0 Hz, 1H), 6.99 (d, *J* = 8.4 Hz, 2H), 6.09 (s, 2H), 2.27 (s, 3H); ^13^C NMR (CDCl_3_, 75 MHz) *δ*/ppm: 144.7, 140.0, 139.0, 135.1, 129.1, 128.9, 127.7, 127.2, 123.6, 122.8, 120.3, 119.2, 116.1, 53.1, 21.4; MS (ESI) *m*/*z* (%, fragment): 280 (100); HRMS (*m*/*z*) for C_16_H_13_N_3_S: [M + H]^+^_calcd_ = 280.0830, and [M + H]^+^_measured_ = 280.0828.

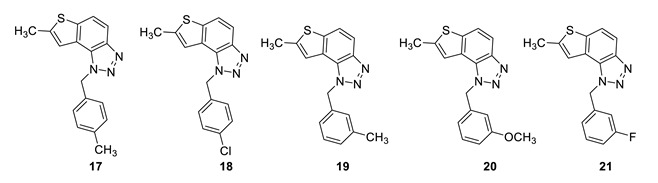



7-methyl-1-(4-methylbenzyl)-1*H*-thieno [3’,2’:3,4]benzo [1,2-*d*][1,2,3]triazole (**17**): 12 mg (isolated 15%), yellow oil; *R_f_* (PE/E (30%)) = 0.46; ^1^H NMR (CDCl_3_, 300 MHz) *δ*/ppm: 7.91 (d, *J* = 8.6 Hz, 1H), 7.68 (d, *J* = 8.7 Hz, 1H), 7.18 (s, 1H), 7.12 (d, *J* = 8.3 Hz, 2H), 7.06 (d, *J* = 8.3 Hz, 2H), 6.04 (s, 2H), 2.60 (s, 3H), 2.30 (s, 3H); ^13^C NMR (CDCl_3_, 75 MHz) *δ*/ppm: 142.6, 138.0, 132.3, 129.7, 126.6, 123.0, 118.9, 118.1, 115.0, 52.8, 21.1, 16.1; MS (ESI) *m*/*z* (%, fragment): 294 (100); HRMS (*m*/*z*) for C_17_H_15_N_3_S: [M + H]^+^_calcd_ = 294.0987, and [M + H]^+^_measured_ = 294.0988.

1-(4-chlorobenzyl)-7-methyl-1*H*-thieno [3’,2’:3,4]benzo [1,2-*d*][1,2,3]triazole (**18**): 14 mg (isolated 28%), colourless oil; *R_f_* (PE/E = 50%) = 0.51; ^1^H NMR (CDCl_3_, 300 MHz) *δ*/ppm: 7.91 (d, *J* = 9.0 Hz, 1H), 7.70 (d, *J* = 9.0 Hz, 1H), 7.30 (d, *J* = 8.4 Hz, 2H), 7.15–7.09 (m, 3H), 6.05 (s, 2H), 2.61 (s, 3H); ^13^C NMR (CDCl_3_, 75 MHz) *δ*/ppm: 144.7, 143.0, 139.5, 134.3, 133.9, 129.3, 128.0, 122.8, 119.1, 117.7, 115.1, 52.3, 16.2; MS (ESI) *m*/*z* (%, fragment): 314 (100); HRMS (*m*/*z*) for C_16_H_12_ClN_3_S: [M + H]^+^_calcd_ = 314.0440, and [M + H]^+^_measured_ = 314.0441.

7-methyl-1-(3-methylbenzyl)-1*H*-thieno [3’,2’:3,4]benzo [1,2-*d*][1,2,3]triazole (**19**): 13 mg (isolated 29%), yellow oil; *R_f_* (PE/E (40%)) = 0.46; ^1^H NMR (CDCl_3_, 300 MHz) *δ*/ppm: 7.92 (d, *J* = 8.9 Hz, 1H), 7.65 (d, *J* = 8.8 Hz, 1H), 7.23–7.18 (m, 2H), 7.09 (d, *J* = 7.5 Hz, 1H), 7.03–6.96 (m, 2H), 6.04 (s, 2H), 2.60 (s, 3H), 2.28 (s, 3H); ^13^C NMR (CDCl_3_, 75 MHz) *δ*/ppm: 142.6, 139.3, 138.9, 135.3, 129.1, 128.9, 127.3, 123.6, 123.0, 118.9, 118.1, 115.1, 52.9, 21.4, 16.2; MS (ESI) *m*/*z* (%, fragment): 294 (100); HRMS (*m*/*z*) for C_17_H_15_N_3_S: [M + H]^+^_calcd_ = 294.0987, and [M + H]^+^_measured_ = 294.0981.

1-(3-methoxybenzyl)-7-methyl-1*H*-thieno [3’,2’:3,4]benzo [1,2-*d*][1,2,3]triazole (**20**): 14 mg (isolated 35%), yellow oil; *R_f_* (PE/E (40%)) = 0.38; ^1^H NMR (CDCl_3_, 300 MHz) *δ*/ppm: 7.91 (d, *J* = 8.9 Hz, 1H), 7.68 (d, *J* = 8.9 Hz, 1H), 7.22 (d, *J* = 7.9 Hz, 1H), 7.17 (s, 1H), 6.85–6.75 (m, 2H), 6.70 (s, 1H), 6.05 (s, 2H), 3.71 (s, 3H), 2.60 (s, 3H); ^13^C NMR (CDCl_3_, 75 MHz) *δ*/ppm: 160.1, 144.7, 142.6, 139.3, 136.9, 130.2, 128.3, 123.0, 118.9, 118.8, 118.1, 115.0, 113.6, 112.3, 55.2, 52.8, 16.2; MS (ESI) *m*/*z* (%, fragment): 310 (100); HRMS (*m*/*z*) for C_17_H_15_N_3_OS: [M + H]^+^_calcd_ = 310.0936, and [M + H]^+^_measured_ = 310.0939.

1-(3-fluorobenzyl)-7-methyl-1*H*-thieno [3’,2’:3,4]benzo [1,2-*d*][1,2,3]triazole (**21**): 8 mg (isolated 16%), colourless oil; *R_f_* (PE/E (40%)) = 0.44; ^1^H NMR (CDCl_3_, 300 MHz) *δ*/ppm: 7.85 (d, *J* = 8.9 Hz, 1H), 7.63 (d, *J* = 8.9 Hz, 1H), 7.27–7.19 (m, 1H), 7.05 (s, 1H), 6.96–6.87 (m, 2H), 6.80 (d, *J* = 9.5 Hz, 1H), 6.01 (s, 2H), 2.51 (s, 3H); ^13^C NMR (CDCl_3_, 75 MHz) *δ*/ppm: 161.9 (d, *J*_CF_ = 247.8 Hz), 143.7, 141.9, 138.5, 136.8 (d, *J*_CF_ = 7.4 Hz), 129.8 (d, *J*_CF_ = 8.5 Hz), 127.1, 121.8, 121.1 (d, *J*_CF_ = 3.1 Hz), 118.1, 116.7, 114.3 (d, *J*_CF_ = 20.9 Hz), 114.1, 112.7 (d, *J*_CF_ = 22.4 Hz), 51.4, 15.3; MS (ESI) *m*/*z* (%, fragment): 298 (100); HRMS (*m*/*z*) for C_16_H_12_FN_3_S: [M + H]^+^_calcd_ = 298.0736, and [M + H]^+^_measured_ = 298.0734.

### 2.6. Synthesis of Triazolium Salts ***22**–**31***

In a glass reaction tube with a magnetic stirring bar, the corresponding thienobenzo-triazoles **14**–**21** (0.2 mmol, 1 equivalent), dry dichloromethane (0.4 mL), and iodomethane (2 mmol, 10 equivalents) were added. The reaction mixtures were purged with argon and then stirred at 60 °C for 24 h. After this period, the mixtures were cooled to 0 °C and diluted with 5 mL of diethyl ether. Following precipitation, the tubes were centrifuged three times for 10 min at 3000 rpm. The solvent was decanted, and the precipitate was washed three to five times with diethyl ether. The crude powders were then dried under high vacuum, yielding the pure triazolium salts **22**–**29**. The solubility of triazolium salts **22**–**29** is low in CDCl_3_, which is reflected in the signals, especially in the ^13^C NMR spectra.

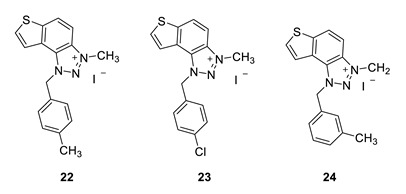



3-methyl-1-(4-methylbenzyl)-1*H*-thieno [3’,2’:3,4]benzo [1,2-*d*][1,2,3]triazol-3-ium (**22**): 8 mg (isolated 30%), yellow powder; *R_f_* (DCM (100%)) = 0.19; ^1^H NMR (CDCl_3_, 600 MHz) *δ*/ppm: 7.99 (d, *J* = 9.1 Hz, 1H), 7.80 (dd, *J* = 9.1, 0.7 Hz, 1H), 7.55 (d, *J* = 5.5 Hz, 1H), 7.51 (dd, *J* = 5.5, 0.7 Hz, 1H), 7.10 (q, *J* = 17.3, 8.7 Hz, 4H), 2.29 (s, 3H), 1.59 (s, 3H); ^13^C NMR (CDCl_3_, 75 MHz) *δ*/ppm: 143.1, 139.3, 135.1, 132.8, 130.9, 130.4, 127.8, 127.5, 127.2, 126.5, 120.6, 109.0, 57.2, 40.2, 21.2; MS (ESI) *m*/*z* (%, fragment): 294 (100); HRMS (*m*/*z*) for C_17_H_16_N_3_S^+^: [M + H]^+^_calcd_ = 294.0987, and [M + H]^+^_measured_ = 294.0985.

1-(4-chlorobenzyl)-3-methyl-1*H*-thieno [3’,2’:3,4]benzo [1,2-*d*][1,2,3]triazol-3-ium (**23**): 15 mg (isolated 42%), yellow powder; *R_f_* (DCM (100%)) = 0.15; ^1^H NMR (CDCl_3_, 600 MHz) *δ*/ppm: 8.34 (d, *J* = 8.6 Hz, 1H), 8.10 (d, *J* = 8.8 Hz, 1H), 7.98 (d, *J* = 4.9 Hz, 1H), 7.85 (d, *J* = 5.7 Hz, 1H), 7.43–7.40 (m, 4H), 6.45 (s, 2H), 4.86 (s, 3H); ^13^C NMR (CDCl_3_, 75 MHz) *δ*/ppm: 150.9, 130.9, 130.1, 129.5, 129.4, 128.9, 122.6, 121.3, 57.3, 34.6 (signals for 4 quaternary carbons are missing); MS (ESI) *m*/*z* (%, fragment): 314 (100); HRMS (*m*/*z*) for C_16_H_13_ClN_3_S^+^: [M + H]^+^_calcd_ = 314.0440, and [M + H]^+^_measured_ = 314.0436.

3-methyl-1-(3-methylbenzyl)-1*H*-thieno [3’,2’:3,4]benzo [1,2-*d*][1,2,3]triazol-3-ium (**24**): 4 mg (isolated 15%), yellow powder; *R_f_* (DCM (100%)) = 0.20; ^1^H NMR (CDCl_3_, 600 MHz) *δ*/ppm: 8.34 (d, *J* = 11.1 Hz, 1H), 8.25 (d, *J* = 11.1 Hz, 1H), 7.94 (d, *J* = 6.3 Hz, 1H), 7.78 (d, *J* = 6.3 Hz, 1H), 7.31 (t, *J* = 7.4 Hz, 1H), 7.22 (d, *J* = 7.4 Hz, 1H), 7.18–7.16 (m, 2H), 6.34 (s, 2H), 4.92 (s, 3H), 2.35 (s, 3H); ^13^C NMR (CDCl_3_, 75 MHz) *δ*/ppm: 142.7, 132.8, 130.7, 127.6, 127.1, 124.5, 122.7, 120.5, 116.0, 53.4, 30.9, 21.5 (signals for 5 quaternary carbons are missing); MS (ESI) *m*/*z* (%, fragment): 294 (100); HRMS (*m*/*z*) for C_17_H_16_N_3_S^+^: [M + H]^+^_calcd_ = 294.0987, and [M + H]^+^_measured_ = 294.0991.

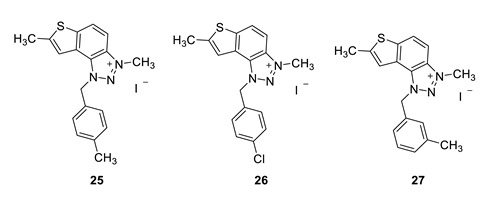



3,7-dimethyl-1-(4-methylbenzyl)-1*H*-thieno [3’,2’:3,4]benzo [1,2-*d*][1,2,3]triazol-3-ium (**25**): 6 mg (isolated 27%), white powder; *R_f_* (DCM (100%)) = 0.22; ^1^H NMR (CDCl_3_, 300 MHz) *δ*/ppm: 8.21 (d, *J* = 6.6 Hz, 1H), 8.07–8.04 (m, 1H), 7.52 (s, 1H), 7.30–7.28 (m, 1H), 7.28–7.27 (m, 1H), 7.24–7.23 (m, 1H), 7.23–7.21 (m, 1H), 6.31 (s, 2H), 4.84 (s, 3H), 2.74 (s, 3H), 2.36 (s, 3H); MS (ESI) *m*/*z* (%, fragment): 308 (100); HRMS (*m*/*z*) for C_18_H_18_N_3_S^+^: [M + H]^+^_calcd_ = 308.1216, and [M + H]^+^_measured_ = 308.1218.

1-(4-chlorobenzyl)-3,7-dimethyl-1*H*-thieno [3’,2’:3,4]benzo [1,2-*d*][1,2,3]triazol-3-ium (**26**): 5 mg, (isolated 22%), white powder; *Ry_f_* (DCM (100%)) = 0.21; ^1^H NMR (CDCl_3_, 300 MHz) *δ*/ppm: 8.20 (d, *J* = 6.2 Hz, 1H), 7.98–7.90 (m, 1H), 7.58 (s, 1H), 7.44–7.38 (m, 4H), 6.41 (s, 2H), 4.79 (s, 3H), 2.75 (s, 3H); ^13^C NMR (CDCl_3_, 75 MHz) *δ*/ppm: 156.2, 149.1, 136.0, 129.9, 129.4, 127.3, 126.9, 123.4, 118.4, 65.9, 16.6, 15.3 (signals for 3 quaternary carbons are missing); MS (ESI) *m*/*z* (%, fragment): 328 (100); HRMS (*m*/*z*) for C_17_H_15_ClN_3_S^+^: [M + H]^+^_calcd_ = 328.0122, and [M + H]^+^_measured_ = 328.0120.

3,7-dimethyl-1-(3-methylbenzyl)-1*H*-thieno [3’,2’:3,4]benzo [1,2-*d*][1,2,3]triazol-3-ium (**27**): 2 mg (isolated 15%), white powder; *R_f_* (DCM (100%)) = 0.24; ^1^H NMR (CDCl_3_, 300 MHz) *δ*/ppm: 8.21 (d, *J* = 9.3 Hz), 8.11–8.09 (m, 1H), 7.49 (s, 1H), 7.31 (t, *J* = 7.6 Hz, 1H), 7.23 (d, *J* = 7.1 Hz, 1H), 7.19–7.16 (m, 2H), 6.31 (s, 2H), 4.86 (s, 3H), 2.73 (s, 3H), 2.36 (s, 3H); MS (ESI) *m*/*z* (%, fragment): MS (ESI) *m*/*z* (%, fragment): 308 (100); HRMS (*m*/*z*) for C_18_H_18_N_3_S^+^: [M + H]^+^_calcd_ = 308.1216, and [M + H]^+^_measured_ = 308.1218.

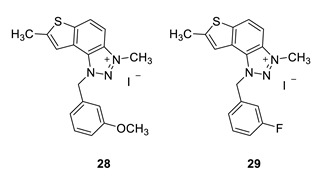



1-(3-methoxybenzyl)-3,7-dimethyl-1*H*-thieno [3’,2’:3,4]benzo [1,2-*d*][1,2,3]triazol-3-ium (**28**): 4 mg, (isolated 26%), yellow powder; *R_f_* (DCM (100%)) = 0.18; ^1^H NMR (CDCl_3_, 300 MHz) *δ*/ppm: 8.19 (br s, 1H), 8.00 (br s, 1H), 7.53 (br s, 1H), 7.30 (d, *J* = 8.4 Hz, 1H), 7.13–7.10 (m, 1H), 6.95–6.91 (m, 2H), 6.35 (br s, 2H), 4.81 (br s, 3H), 3.81 (s, 3H), 2.73 (s, 3H); MS (ESI) *m*/*z* (%, fragment): 324 (100).

1-(3-fluorobenzyl)-3,7-dimethyl-1*H*-thieno [3’,2’:3,4]benzo [1,2-*d*][1,2,3]triazol-3-ium (**29**): obtained only in traces according to NMR analysis, not isolated and evaluated. Its synthesis should be optimized in the following investigations.

Two additional triazolium salts, **30** and **31**, were also prepared from the previously obtained and characterized neutral thienobenzo-triazole [20] for comparison of the non-charged and charged analogues.

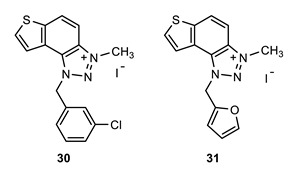



1-(3-chlorobenzyl)-3-methyl-1*H*-thieno [3’,2’:3,4]benzo [1,2-*d*][1,2,3]triazol-3-ium (**30**): 10 mg (isolated 43%), yellow powder; *R_f_* (DCM (100%)) = 0.20; ^1^H NMR (CDCl_3_, 300 MHz) *δ*/ppm: 8.39–8.31 (m, 2H), 8.13–8.07 (m, 2H), 8.02–7.96 (m, 2H), 7.86–7.82 (m, 2H), 6.47 (s, 2H), 4.87 (s, 3H); MS (ESI) *m*/*z* (%, fragment): 314 (100); HRMS (*m*/*z*) for C_16_H_13_ClN_3_S^+^: [M + H]^+^_calcd_ = 314.0440, and [M + H]^+^_measured_ = 314.0436.

1-(furan-2-ylmethyl)-3-methyl-1*H*-thieno [3’,2’:3,4]benzo [1,2-*d*][1,2,3]triazol-3-ium (**31**): 6 mg (isolated 29%), yellow powder; *R_f_* (DCM (100%)) = 0.17; ^1^H NMR (CDCl_3_, 600 MHz) *δ*/ppm: 8.34 (d, *J* = 9.0 Hz, 1H), 8.11 (d, *J* = 9.0 Hz, 1H), 7.99 (d, *J* = 5.8 Hz, 1H), 7.86 (d, *J* = 5.8 Hz, 1H), 7.50–7.47 (m, 2H), 7.13 (d, *J* = 3.8 Hz, 2H), 6.43 (s, 2H), 4.82 (s, 3H); MS (ESI) *m*/*z* (%, fragment): 270 (100); HRMS (*m*/*z*) for C_14_H_12_N_3_OS^+^: [M + H]^+^_calcd_ = 270.0623, and [M + H]^+^_measured_ = 270.0627.

### 2.7. Inhibition Activity

The modified Ellman’s method was used to determine the synthesized triazole compounds’ cholinesterase inhibitory potential [22]. Acetylcholinesterase from electric eel (eeAChE, type VI-S), butyrylcholinesterase from equine serum (eqBChE, E.C. 3.1.1.8), acetylthiocholine iodide (ATChI), *S*-butyrylthiocholine iodide (BTChI), Trisma base, and reference standard galantamine were purchased from Sigma Aldrich (ST. Louis, MO). Ellman’s reagent 5,50-dithiobis-(2-nitrobenzoic acid) (DTNB) was purchased from Zwijndrecht (Belgium). All tested samples were dissolved in ethanol in concentrations ranging from 0.1 to 500 μM (final concentration in reaction mixture). Reaction mixtures were prepared as follows: 10 μL of tested solution of different concentrations, 10 μL of enzyme (AChE and BChE final concentrations 0.03 U/mL, prepared in 20 mM of Tris buffer, pH 7.5), and 10 μL of DTNB (final concentration 0.3 mM, prepared in 50 mM of Tris buffer, pH 8) were added to 180 μL of Tris buffer (50 mM, pH 8.0). The reaction was started by adding 10 μL of substrate ATChI/BTChI (final concentration 0.5 mM, prepared in Tris buffer, pH 8.0). The tested sample was replaced by 10 μL of buffer solution in the control measurement. A blank test for each sample contained the same solutions only without enzymes; its volume was replaced by adding 10 μL of buffer. The absorbance of the reaction mixture was measured at 405 nm over 6 min at room temperature using a 96-well microplate reader (Bio Tek 800TSUV Absorbance Reader, Agilent). Experiments were run in triplicate. The percentage of enzyme inhibition was calculated according to the equation: Inhibition (%) = [(A_C_ − A_T_)/A_C_] × 100 where A_C_ is the control enzyme activity, without the test sample, and A_T_ is the enzyme activity with the test sample, and represented as mean value ± standard deviation. Data of mean inhibition values were used for the IC_50_ value calculation by nonlinear curve fitting of compound concentrations (log) vs. response. The inhibition contribution from ethanol was measured and subtracted from the calculation.

### 2.8. Computational Study

The structures of the ligands used for the docking study were obtained by geometry optimization at the B3LYP/6-31G(d) level of theory using the Gaussian16 program package [23]. A vibrational analysis of the optimized geometries confirmed the absence of imaginary frequencies (NImag = 0), indicating that the structures correspond to minima on the potential energy surface. Optimized ligand structures were further prepared for docking by adding Gasteiger charges, detecting rotatable bonds, identifying aromatic carbons, and setting torsional degrees of freedom. Crystallographic data for AChE (PDB ID: 4EY7) and BChE (PDB ID: 7AIY) were obtained from the Protein Data Bank [24,25] and prepared for docking by removing non-amino acid residues, adding polar hydrogens, and assigning Kollman charges. Molecular docking was performed using Autodock4.0 [26], employing the Lamarckian Genetic Algorithm, which generated 25 docking poses for each ligand. The enzyme residues were kept rigid throughout the process. The search space was defined by setting a GridBox around the center of the active site for each ChE (details given in Supplementary Materials), with box dimensions of 60 × 60 × 60 points and a grid spacing of 0.375 Å.

## 3. Results and Discussion

### 3.1. Synthesis and Spectroscopic Characterization of New 1,2,3-Triazolium Salts

**Scheme 1:**
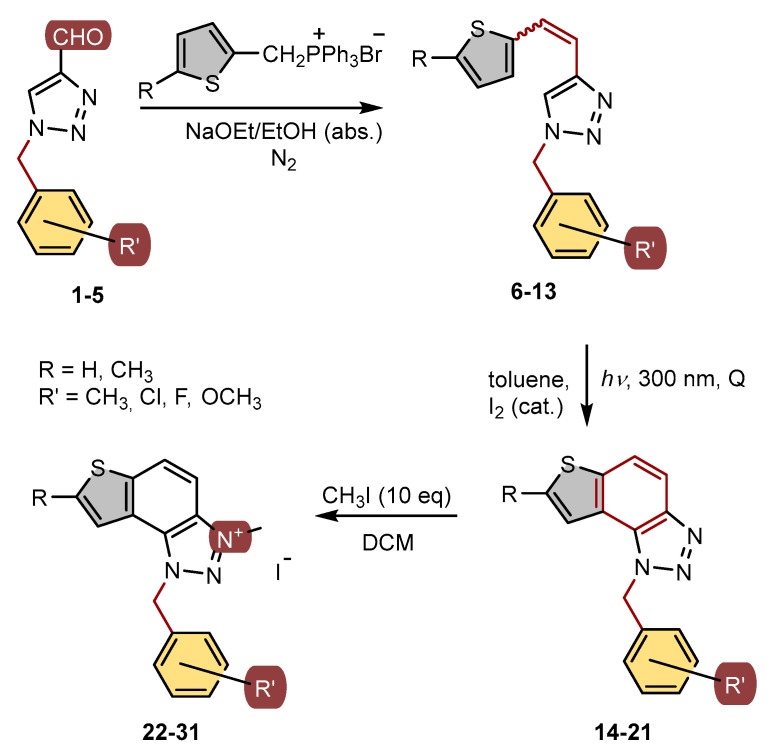
Reaction pathway for the synthesis of thienobenzo-triazoles **14**–**21** and their 1,2,3-triazolium salts **22**–**31**.

New targeted triazolium salts with the substituted benzyl group on the charged 1,2,3-triazole ring were synthesized to investigate their inhibition potential toward AChE and BChE based on the previous encouraging results. To obtain the same, a series of four consecutive reactions was carried out (Scheme 1). The 1,4-disubstituted triazole aldehydes **1**–**5** as reagents in this research were prepared according to the known procedure 1-(4-nitrophenyl)-1*H*-1,2,3-triazole-4-carbaldehyde and the corresponding amines (isolated yields 23–82%) [21].

The aldehydes **1**–**5** entered into the Wittig reaction, where they react with the corresponding phosphorus ylides containing the thiophene ring, thus obtaining triazole-thienostilbenes **6**–**13** as mixtures of *cis*- and *trans*-isomers (isolated yields for derivatives **6**–**8** 80–91% and isolated yields for derivatives **9**–**13** 13–32%, Scheme 1). Mixtures of the geometrical isomers of triazole-thienostilbenes **6**–**13** were not separated, and due to the presence of the resulting conjugated system, they were subjected to photochemical cyclization leading to thienobenzo-triazoles **14**–**21** (isolated yields 15–73%, Scheme 1). The photoproducts **14**–**21** were transferred to triazolium salts **22**–**29** as targeted products using methyl iodide (isolated yields 15–42%, except **29** obtained only in traces according to NMR analysis, not isolated and evaluated, Scheme 1). Two additional triazolium salts, **30** and **31**, were also prepared from the previously obtained and characterized neutral thienobenzo-triazoles (See Materials and Methods) [20].

**Figure 2:**
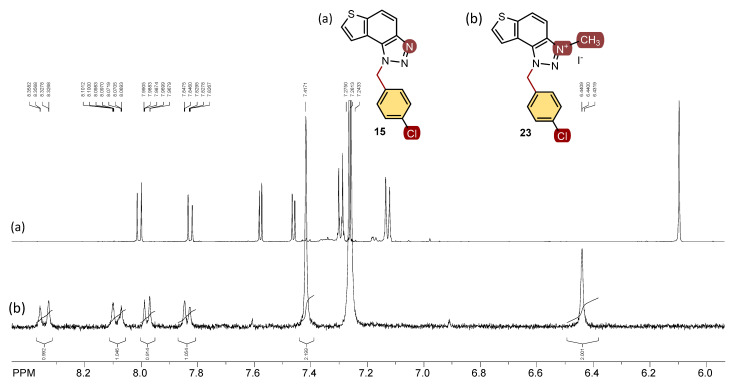
^1^H NMR spectra (CDCl_3_) of thienobenzo-triazole **16** (**a**) and its triazolium salt **24** (**b**).

All isolated triazole aldehydes **1**–**5**, triazole-thienostilbenes **6**–**13**, thienobenzo-triazoles **14**–**21**, and triazolium salts **22**–**31** were fully spectroscopically characterized (except **29** obtained only in traces, See Materials and Methods and Supplementary Materials). The ^1^H NMR spectra of **6**–**13** show the resolved patterns for ethylenic protons of both geometrical isomers between 6.5 and 7.0 ppm (*cis*-isomers) and between 6.8 and 7.7 ppm (*trans*-isomers) with the characteristic coupling constants, as well as signals for the protons on various substituents and singlets for the protons on the triazole rings (6.9 and 7.9 ppm). Preparative irradiations of **6**–**13** in toluene solutions under aerobic conditions gave the thienobenzo-triazoles **14**–**21**. They were isolated in high yields (Scheme 1) and characterized by NMR spectroscopy (See Materials and Methods and Supplementary Materials). The formation of the electrocyclization photoproducts **14**–**21** was generally accompanied by the formation of some high-molecular-weight products, which were not investigated. In their ^1^H NMR spectra, the disappearance of the ethylenic protons and the singlets for the protons on the 1,2,3-triazole rings can be seen in comparison with the starting **6**–**13** (Figure 2). In the ^1^H NMR spectra of triazolium salts **22**–**31**, a new signal of the methyl group on the triazole nitrogen is visible around 4.8 ppm. All signals in the triazolium salts **22**–**31** are shifted to a less shaded area compared to their neutral analogues **14**–**21**.

### 3.2. Inhibitory Activity of Thienobenzo-Triazoles ***14**–**21*** and Triazolium Salts ***22**–**31*** Toward Enzymes Cholinesterases

Newly prepared thienobenzo-triazoles **14**–**21** and triazolium salts **22**–**31** were tested for inhibitory activity toward AChE and BChE in various concentrations, depending on solubility. A spectrophotometric Ellman assay was applied [22], and the results obtained were compared to those of commercially available galantamine as the reference standard. The IC_50_ values of triazole derivatives **14**–**31** for AChE and BChE inhibition are presented in Table 1.

**Figure 3:**
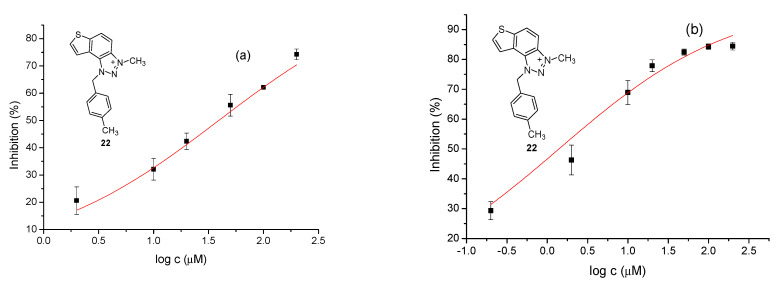
Dose–response curve for the inhibition of AChE (**a**) and BChE (**b**) by **22**.

**Figure 4:**
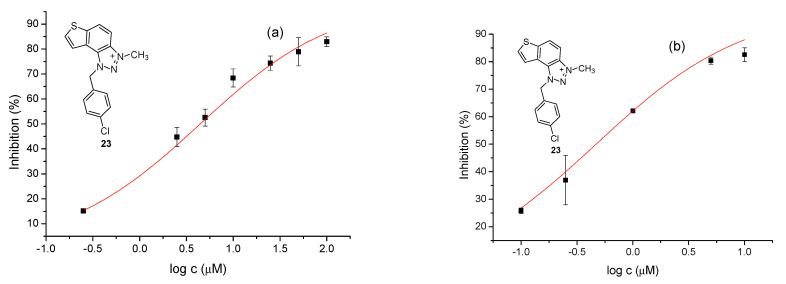
Dose–response curve for the inhibition of AChE (**a**) and BChE (**b**) by **23**.

The tested compounds were divided into two main groups: thienobenzo-triazoles, presented on the left side of Table 1, and triazolium salts, presented on the right. Analogs can be found between these two groups that differ only in charge, while the type and position of the substituents are the same. According to structural features, the following structure–activity relationship can be noted. The presence of a charge on the triazole ring favored more potent inhibition toward both enzymes, AChE and BChE. The leading compounds were **22**, **23**, **26**, **27**, and **30**, which inhibited both enzymes in the excellent to very good range of concentrations compared to the standard (Table 1, Figure 3 and Figure 4). The strongest inhibitor of both enzymes was derivative **23**, with a value for BChE in the nanomolar range that was better than the standard (Table 1) and a slightly higher but still excellent value for AChE. The exception among the salts was **25**, whose IC_50_ values were not in such a good range of concentrations. The presence of chlorine on the benzyl ring supports the activity since three salts with excellent results, **23**, **26**, and **30**, possess this substituent. Introduction of methyl substituent on thiophene ring, from **22** to **25**, significantly reduced activity toward enzymes. The same structural change from **23** to **26** resulted similarly. Derivatives **24** and **27** were an exception, but only toward AChE, because the methylated charged derivative **27** was a better inhibitor of this enzyme than the unmethylated **24**.

Uncharged thienobenzo-triazoles **14**–**21** prefer inhibition of BChE over AChE. This observation is consistent with the conclusions of our previous research, which indicated that neutral photoproducts tend to be weaker inhibitors and show selectivity toward BChE [20]. It is supported additionally by the fact that the uncharged analog of **30** analyzed in the previous study [20] showed weaker inhibition of both enzymes. For herein tested thienobenzo-triazoles, conversion to salt significantly increased inhibition of AChE and, to a good extent, also of BChE. Exceptions were **16** and **17**, which achieved better IC_50_ values for BChE than the corresponding salts **24** and **25**.

It can be concluded that the most important structural feature for thienobenzo-triazoles is the presence/absence of charge. Triazolium salts were shown to be dual inhibitors whose potency of inhibition varies depending on the substituent of thiophene and triazole heterocycles.

### 3.3. ADMET Properties of Biologically Active Thienobenzo-Triazoles and Triazolium Salts

Absorption, distribution, metabolism, excretion, and toxicity (ADME(T)) studies are critical in drug development. They give insights into whether a compound exhibits drug-like pharmacokinetic properties and whether it has properties that will cause safety concerns in people [27]. ADMET properties are investigated in silico, in vitro, and in vivo. In early drug discovery, in silico tools are indispensable. For this study, free in silico tools [28,29] were employed to screen the candidate compounds.

As compounds in the form of salts (**22**–**31**) are developed mainly to enhance the ADMET part of the properties, it is important to compare the neutral molecule against its salt counterpart. It can be seen (Table 2) that the water solubility, although higher, remains very low, and that all other properties are alike. To determine whether the in silico models could differentiate between the neutral molecule and the salt, multiple software programs were utilized, revealing variations in the absorption estimates. As other parts of the ADMET are concerned, the results are similar. To further test whether there is a deterioration or an improvement in the CNS bioavailability for the most active compounds **22** and **23**, in comparison to their neutral counterparts **14** and **15**, additional in silico analysis was performed. Additional marker PPB is tested using one additional in silico tool [30]. PPB is the plasma protein binding percentage, which indicates how much of the drug will be bound to the blood proteins. Interpretation of this indicator just on the basis of in silico preliminary results is complex. Drugs that are highlighted as potential CNS candidates are very sensitive to PPB; a maximal concentration in the plasma is one of the key factors with CNS candidates. Here the salts have a slightly lower percentage that is bound to the plasma proteins. Usually the bonds are reversible, but this would require further experimental results for the tested structures. If we consider that the unbound drug is the effective form, then the reduction of PPB can indicate a better bioavailability, and the drug can better distribute in the central nervous system (Table 3). All these calculated parameters will serve as input data for broader SAR or QSAR analyses of the most active molecules.

### 3.4. Molecular Docking of Biologically Active Thienobenzo-Triazoles and Triazolium Salts into Cholinesterases

To reveal the structure of non-covalent complexes formed between the tested compounds and enzymes and identify the interactions responsible for their stabilization, molecular docking of two thienobenzo-triazoles and their triazolium counterparts into the active sites of acetylcholinesterase (AChE) and butyrylcholinesterase (BChE) was performed.

**Figure 5:**
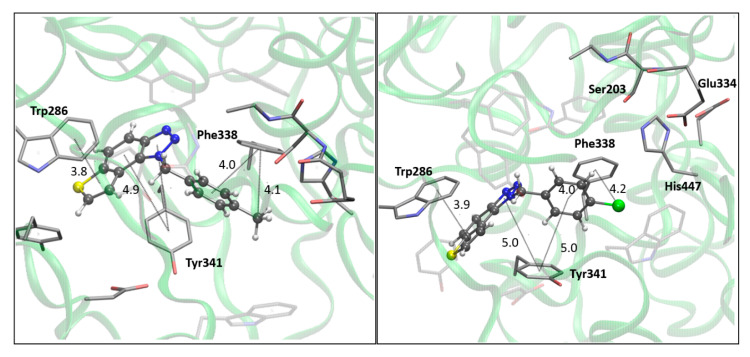
The AChE’s active site structure docked with compounds **14** (**left**) and **15** (**right** panel). Distances in angstroms; hydrogen atoms of the residues omitted for clarity.

**Figure 6:**
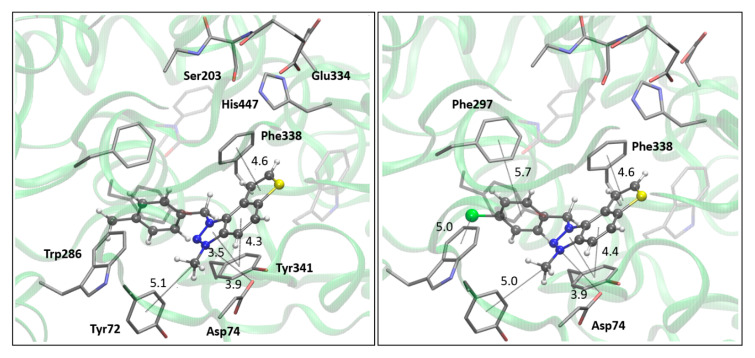
The AChE’s active site structure docked with triazolium salts **22** (**left**) and **23** (**right** panel). Distances in angstroms; hydrogen atoms of the residues omitted for clarity.

The most promising inhibitory activity was observed for triazolium salts **22** and **23**. Their corresponding neutral thienobenzo-triazoles **14** and **15**, which contain the same substituents, also performed very well (Table 1). Figure 5 presents the structures of the complexes resulting from the docking of compounds **14** (left panel) and **15** (right panel) into the active site of AChE, while the results of docking of cations **22** and **23** into the AChE are shown in Figure 6.

Both compounds occupy a similar position within the peripheral anionic subsite (PAS), allowing for π–π stacking between the thienobenzo moiety and Trp286 and similar interactions with Tyr341. The phenyl substituent in both cases is favorably oriented towards Phe338, also engaging in π–π stacking. In compound **14**, the methyl group at the para position of the phenyl substituent participates in an alkyl–π interaction with Phe338. In the protein–ligand complex with **15**, the chlorine atom is situated 4.2 Å from the centroid of Phe338, suggesting the presence of a halogen–π dispersive attraction. The attractive interactions between the chlorine of the ligand and the π-system of the aromatic protein residues can occur in two distinct geometries, depending on the positioning of the Cl atom relative to the aromatic ring: “edge-on” and “face-on” [31]. When the difference between the distance from the chlorine to the center of the aromatic ring and the distance from Cl to the closest carbon of the aromatic ring exceeds 0.3 Å, the “edge-on” geometry is observed. In this case, the distances are 4.2 Å and 3.8 Å, indicating that the interaction can be classified as “edge-on.”

The structures of the complexes formed between acetylcholinesterase and triazolium cations **22** and **23** (Figure 6) reveal that, in both cases, the quaternary nitrogen of the triazole engages in electrostatic interaction with the anionic residues of the active site, specifically aspartate in the peripheral anionic subdomain (Asp74). The distance between the quaternary nitrogen and the oxygen of Asp74 is 3.9 Å form a salt bridge that significantly contributes to the stability of the complex. The conformations of the docked cations **22** and **23** are nearly identical (Figure 6), facilitating alkyl–π interactions between the methyl group on the triazole and Tyr332, as well as parallel π–π stacking of the triazolo-benzo fragment with Tyr341 and the thiophene core with Phe338. For cation **23**, the preferred conformation of the ligand is unfavorable for establishing a chlorine-π interaction, as the Cl atom is positioned at distances exceeding 4.5 Å from the closest aromatic residues.

**Figure 7:**
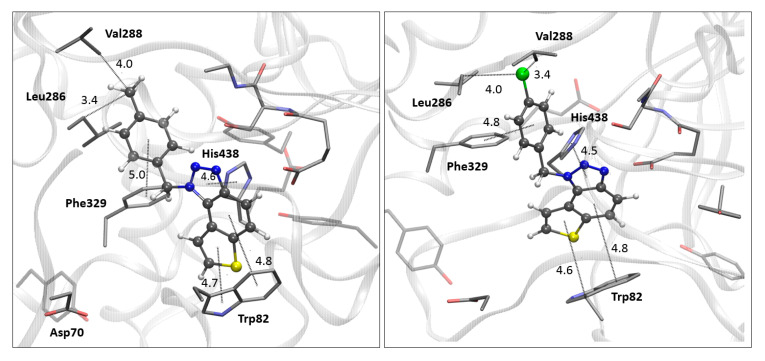
The BChE’s active site structure docked with compounds **14** (**left**) and **15** (**right**). Distances in angstroms; hydrogen atoms of the residues omitted for clarity.

The docking of compounds **14** and **15** into butyrylcholinesterase (BChE) resulted in the structures shown in Figure 7. In both cases, the ligands adopt similar orientations within the active site. The thienobenzo moiety engages in π–π stacking interactions with Trp82, which belongs to the anionic subdomain of the enzyme’s active site. Simultaneously, the triazole ring interacts similarly with the histidine residue in the esteratic site. The phenyl substituent is oriented to facilitate π–π stacking with residue Phe329.

In compound **14**, the methyl group on the phenyl substituent is conveniently positioned within the acyl pocket of BChE, interacting with residues Leu286 and Val288 to achieve an alkyl–alkyl dispersive interaction. In molecule **15**, the chlorine atom behaves similarly, establishing hydrophobic contacts with the aliphatic carbons of the residues in the acyl pocket.

**Figure 8:**
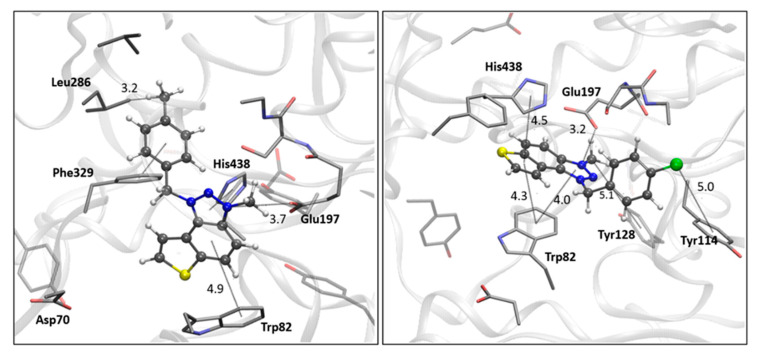
The BChE’s active site structure docked with triazolium salts **22** (**left**) and **23** (**right** panel). Distances in angstroms; hydrogen atoms of the residues omitted for clarity.

Finally, the structures of the complexes between **22** and **23** and BChE, shown in Figure 8, again reveal the presence of the electrostatic attraction between the positively charged ligands’ region and the anionic residue of the enzyme.

As noted previously, the anion involved in AChE is aspartate from the peripheral anionic subsite (PAS); however, in BChE, the anionic residue engaged in this charge–charge interaction is Glu197, which is part of the active site’s anionic subdomain. Similar to the structures of the complexes with ligands **14** and **15**, the thienobenzo moiety establishes parallel π–π stacking with the tryptophan in the anionic subsite, Trp82. In both cations, the phenyl substituent participates in stacking with Phe329. The methyl group on the phenyl of **22** is engaged in alkyl–alkyl dispersion with the acyl pocket. The chlorine atom in **23** is positioned too far from the nearest aromatic residue to form halogen–π dispersion effectively; however, it achieves hydrophobic interaction with residues of the acyl pocket.

### 3.5. Potential Genotoxicity of Thienobenzo-Triazoles ***14**–**21*** and Triazolium Salts ***22**–**31***

The pharmaceutical development process necessitates the evaluation of all involved compounds (including drug substances, impurities, intermediates, etc.) for their potential mutagenic or carcinogenic effects. The ICH M7 guideline governs the regulation of such compounds, requiring that levels present in the drug substance or drug product be calculated based on their acceptable daily intake (ADI) and the drug’s maximum daily dose (MDD). Experimental data for new drug substances and products are often unavailable in the literature databases. The quantitative structure–activity relationship (QSAR) approach is essential in these cases. QSAR models predict biological activity based on structural components [32]. The most commonly utilized tool for this purpose is Lhasa software (Nexus v.2.5.2 (Build 5, July 2022), Derek Nexus v.6.2.1, Meteor Nexus v.3.1.0, Sarah Nexus v.3.2.1, Vitic Link v.2.4.2), which incorporates two complementary models—one rule-based and the other statistical. Predictions from these models are subsequently reviewed by an expert to ensure accuracy.

In the case of compounds **14***–***31**, which are investigated for their potential biological activity with an emphasis on the inhibition of cholinesterase enzymes, the software, as it only recognizes specific functionalities, cannot determine the potential for salts, making the results inconclusive. It does not treat salts as it would their neutral counterparts; therefore, Derek Nexus will give them a negative result, but with lower confidence. All data is presented in the Supplementary Materials (Table S3).

## 4. Conclusions

In this research, pairs of uncharged thienobenzo-triazoles and their charged salts were prepared in order to examine further the role of the positive charge on the nitrogen of the triazole ring in interactions within the active site of the enzymes, and to compare the selectivity of 1,2,3-triazolium salts in relation to their uncharged analogs obtained by photochemical cyclization. Uncharged thienobenzo-triazoles **14**–**21** prefer inhibition of BChE over AChE. This is consistent with our previous findings that neutral photoproducts are generally weaker inhibitors than their charged salts and show selectivity toward BChE. For the thienobenzo-triazoles tested herein, conversion to the salt form (compounds **22**–**31**) significantly increased inhibition of AChE (the most active **23**: IC_50_ 4.4 μM), and, to a good extent, also of BChE (the most active **23**: IC_50_ 0.47 μM). It can be concluded that the most important structural feature for thienobenzo-triazoles is the presence/absence of charge. Triazolium salts **22**–**31** were shown to be dual inhibitors whose potency of inhibition varies depending on the substituent on thiophene and triazole heterocycles. These compounds present a novel scaffold for drug development, as current therapies for Alzheimer’s disease (AD) primarily use selective AChE inhibitors, while non-selective inhibitors of cholinesterases (ChEs) are preferred in Parkinson’s and all stages of AD. Molecular docking of the two most promising salts and their neutral counterparts revealed that, in addition to π–π stacking and hydrophobic interactions, positively charged ligands can form salt bridges with anionic residues in the active sites of ChEs, strongly stabilizing the non-covalent complexes. Since genotoxicity studies are critical in the development of new active substances and final drug forms, in silico assessments of all synthesized compounds were conducted. These studies indicated that compound **18** is the most promising candidate concerning genotoxic safety.

## Figures and Tables

**Table 1:** Calculated IC_50_ values for the inhibition of AChE and BChE by compounds **14**–**31**.

Compound	IC_50_ (AChE/BChE)/μM	Compound	IC_50_ (AChE/BChE)/μM
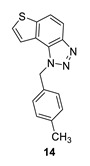	**117.2/24.3**	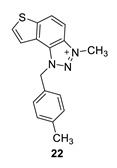	**39.2/1.0**
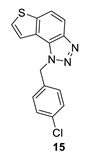	**77.0/34.6**	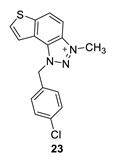	**4.4/0.5**
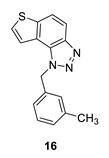	**77.1/2.9**	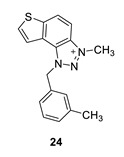	**55.1/7.0**
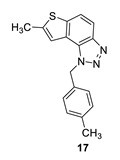	>250/**57.6**	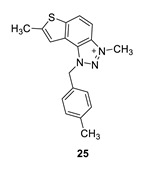	**211.1/102.3**
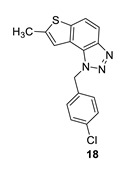	>100/**59.0**	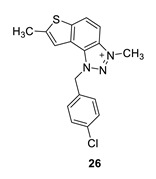	**8.1/13.4**
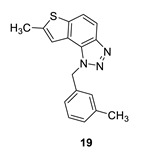	>250/**70.3**	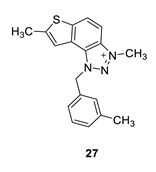	**8.3/9.7**
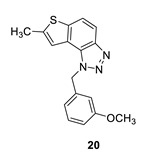	**185.8/90.2**	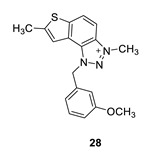	**54.8/10.6**
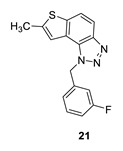	**464.7/171.9**	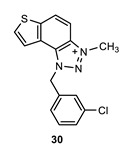	**12.2/2.7**
**galantamine**	**0.15/7.9**	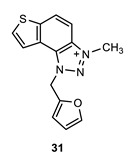	**157.0/16.1**

**Table 2:** In silico study for more indicators of absorption, distribution, metabolism, excretion, and toxicity (ADME(T)) in the human body of thienobenzo-triazoles pairs **14**, **15**, and **17** and their biologically active charged triazolium salts **22**, **23**, **25** and **30**.

Property	Model Name				Compound				Unit
		14	22	15	23	17	25	30	
Absorption	Water solubility	−6.53	−4.78	−6.30	−5.18	−6.20	−4.62	−4.69	log mol/L
Absorption	Caco2 permeability	0.820	0.852	0.892	1.209	0.794	0.780	0.761	log Papp in 10^−6^ cm/s
Absorption	Intestinal absorption (human)	87.7	95.8	76.1	95.9	76.1	76.2	76.1	% Absorbed
Distribution	BBB permeability	0.638	0.484	0.563	0.457	0.630	0.453	0.396	log BB
Metabolism	CYP2D6 substrate	No	No	No	No	No	No	No	
Metabolism	CYP3A4 substrate	Yes	Yes	Yes	Yes	Yes	Yes	Yes	
Metabolism	CYP2C9 inhibitior	No	No	No	No	No	No	No	
Metabolism	CYP2D6 inhibitior	Yes	Yes	Yes	No	No	No	No	
Metabolism	CYP3A4 inhibitior	No	No	No	No	No	No	No	
Excretion	Total clearance	0.960	0.874	0.901	0.855	0.873	0.882	0.925	log mL/min/kg
Excretion	Renal OCT2 substrate	No	No	No	No	No	No	No	
Toxicity	AMES toxicity	Yes	Yes	Yes	Yes	Yes	Yes	Yes	
Toxicity	hERG I inhibitor	No	No	No	No	No	No	No	
Toxicity	hERG II inhibitor	Yes	Yes	Yes	Yes	Yes	Yes	Yes	
Toxicity	Oral rat acute toxicity (LD50)	2.310	2.251	2.470	2.322	2.250	2.250	2.240	mol/kg
Toxicity	Oral rat chronic toxicity (LOAEL)	-	0.286	-	1.444	-	-	-	log mg/kg_bw/day
Toxicity	Hepatotoxicity	No	No	No	Yes	Yes	Yes	Yes	
Toxicity	Skin sensitisation	No	No	No	No	No	No	No	

**Table 3:** In silico PPB of the primary tested pairs.

Compound	14/22	15/23
PPB	98.2/96.9%	98.5/97.9%

## Data Availability

The data presented in this study are available on request from the corresponding authors. The data are not publicly available due to privacy.

## Supplementary Materials

The following supporting information can be downloaded at: https://www.mdpi.com/article/10.3390/biom14111391/s1, Figures S1–S87: ^1^H and ^13^C NMR spectra of compounds **1**–**31**, Figures S88–S108: Mass spectra and HRMS analyses of compounds **1**–**31**; Cartesian coordinates of docked ligands; coordinates of AChE and BChE; Tables S1 and S2, free energies of binding obtained by docking; Table S3, mutagenic potential of thienobenzo-triazoles **14–21** and triazolium salts **22–31**.
