# Supplementary material for: Efficient Access to New Thienobenzo-1,2,3-Triazolium Salts as Preferred Dual Cholinesterase Inhibitors
Source: Biomolecules. 2024 Oct 31;14(11):1391. doi: 10.3390/biom14111391 (PMC11591901; doi:10.3390/biom14111391)
Supplement: Supplementary file 1 [file biomolecules-14-01391-s001.zip › biomolecules-3262546-supplementary.pdf]

Electronic Supporting Information

Efficient access to new thienobenzo-1,2,3-triazolium salts as preferred dual cholinesterase inhibitors

Milena Mlakić,[1] Maja Sviben,[1] Ana Ratković,[2] Anamarija Raspudić,[3] Danijela Barić,[4] Ivana Šagud,[5] Zlata Lasić,[6] Ilijana Odak[3,]* and Irena Škorić[1,]*

[1] Department of Organic Chemistry, Faculty of Chemical Engineering and Technology, University of Zagreb, Trg Marka Marulića 19, HR-10 000 Zagreb, Croatia

[2] Chemistry, Selvita Ltd., Prilaz Baruna Filipovića 29, HR-10 000 Zagreb, Croatia

[3] Department of Chemistry, Faculty of Science and Education, University of Mostar, Matice hrvatske bb, 88 000 Mostar, Bosnia and Herzegovina

[4] Group for Computational Life Sciences, Division of Physical Chemistry, Ruđer Bošković Institute, Bijenička cesta 54, HR-10 000 Zagreb, Croatia

[5] Croatian Agency for Medicinal Products and Medical Devices, Ksaverska Cesta 4, HR-10 000 Zagreb, Croatia

[6 6] TEVA Global R&D, E&L R&D, Pliva Hrvatska d.o.o., Prilaz baruna Filipovića 25, HR-10 000 Zagreb, Croatia

* Correspondence: ilijana.odak@fpmoz.sum.ba (I.O.); iskoric@fkit.unizg.hr (I.Š.)

Contents

1. ^{1}H and ^{13}C NMR spectra of compounds **1–31**…………………………………….....................2
2. Mass spectra and HRMS analyses of compounds **1–31**…………………………........................89
3. Cartesian coordinates of docked ligands…………………………………………………..110
4. Coordinates of AChE and BChE...117
5. Tables S1 and S2, free energies of binding obtained by docking……………….....................307
6. Table S3, mutagenic potential of thienobenzo-triazoles **14–21** and triazolium salts **22–31**…309

1. ^{1}H and ^{13}C NMR spectra of compounds 1–31

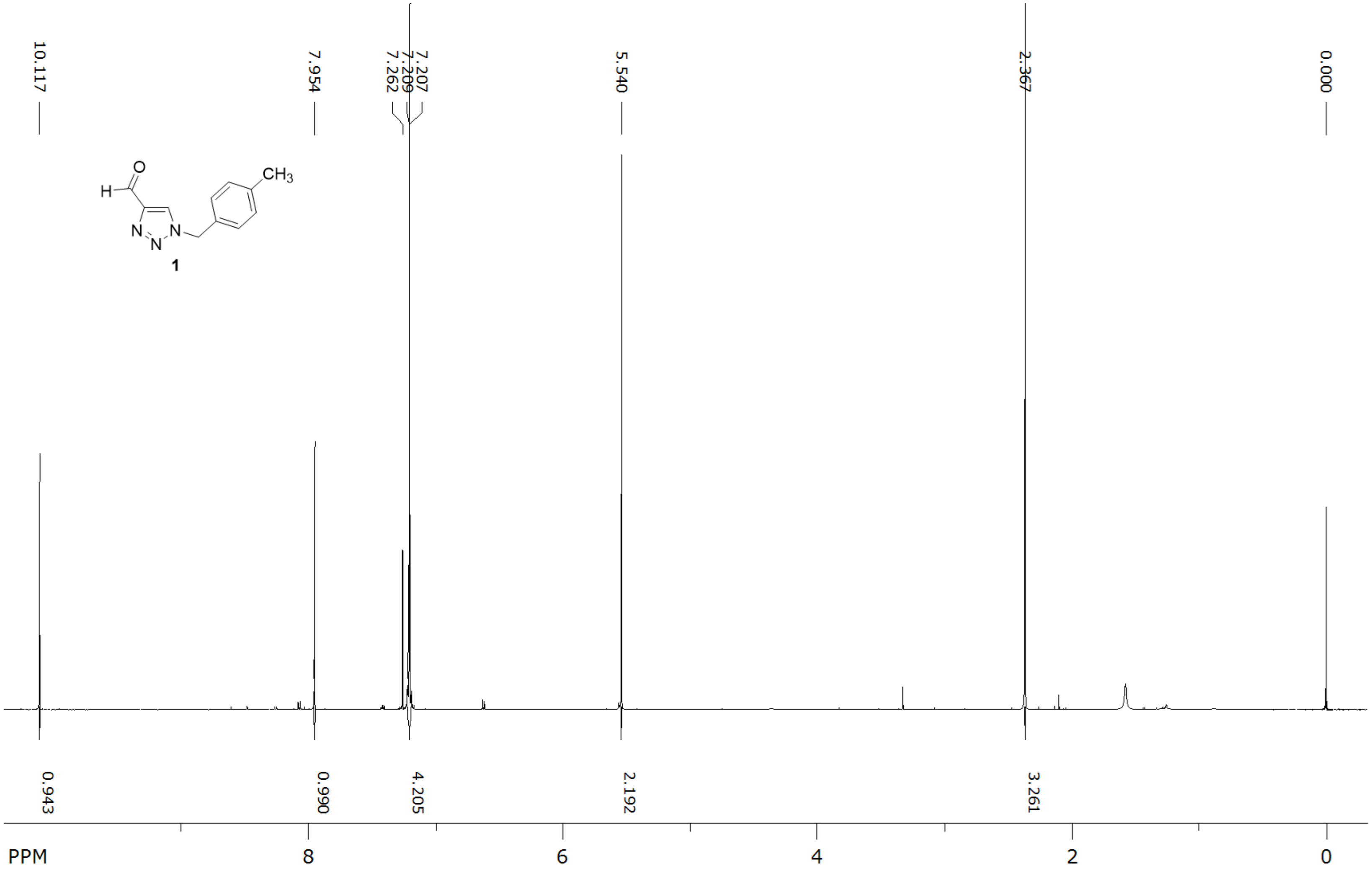

Figure S1. ^{1}H NMR ($CDCl_3$) spectrum of **1**.

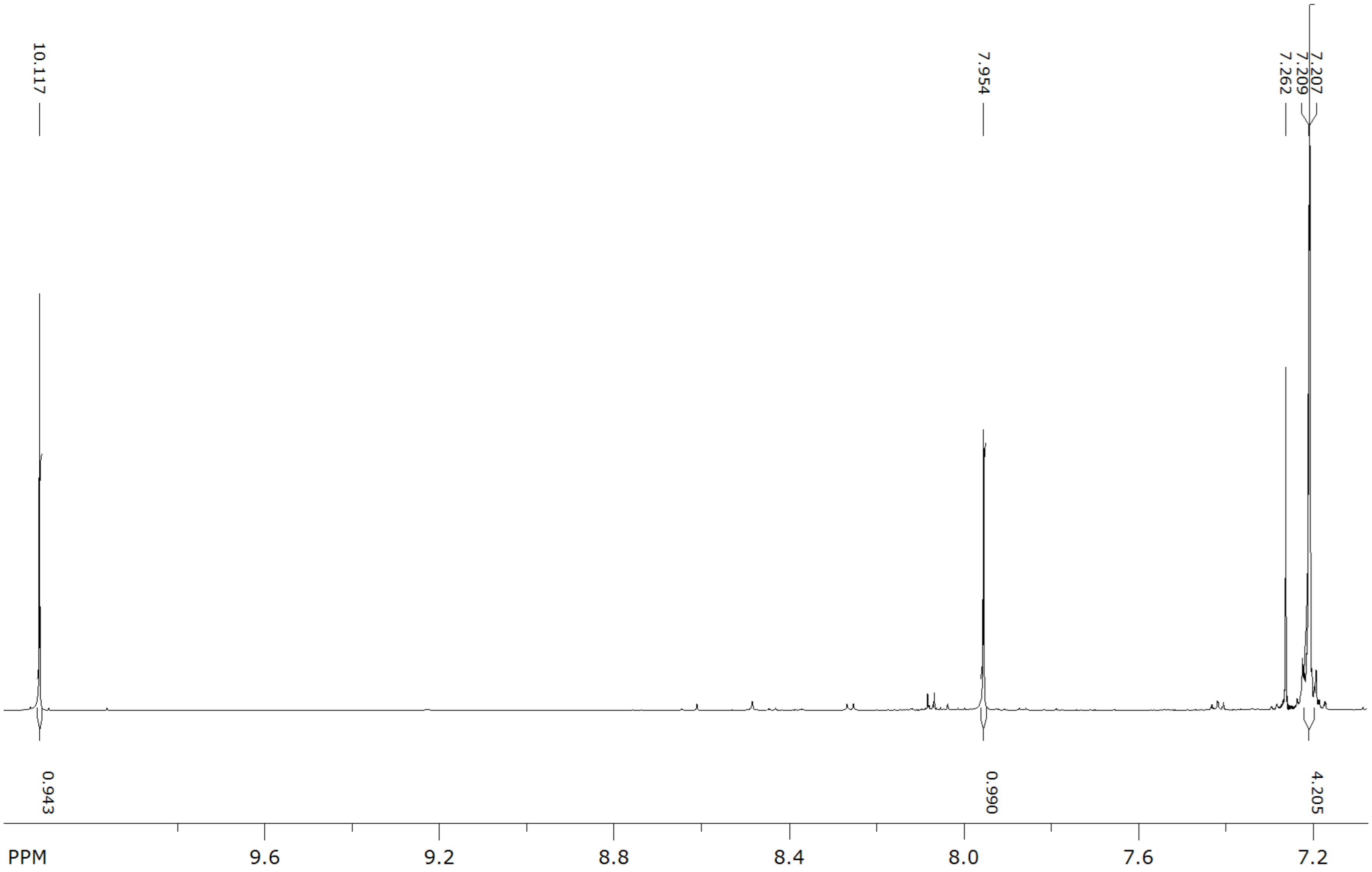

Figure S2. Part of the ^{1}H NMR ($CDCl_3$) spectrum of **1**.

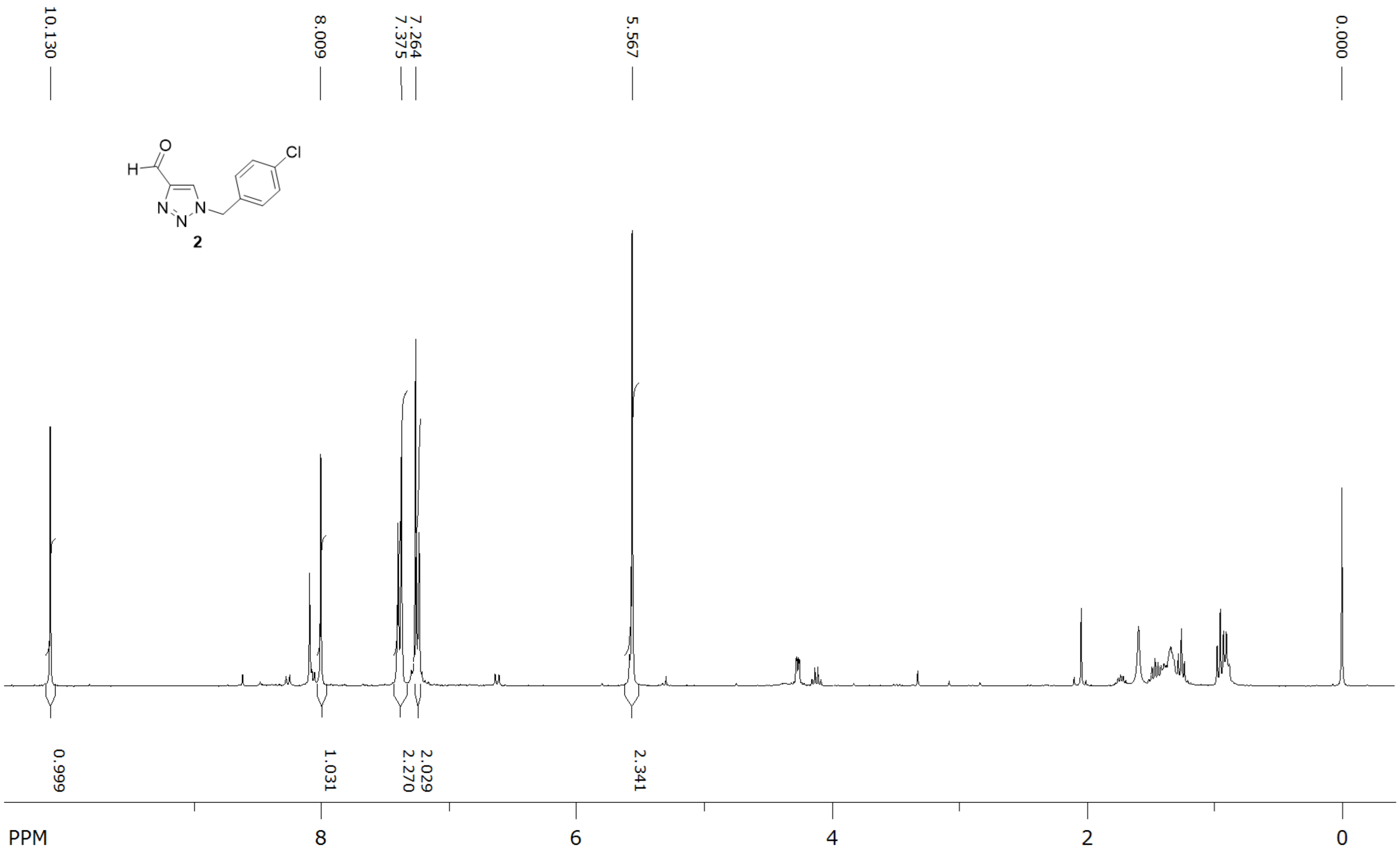

Figure S3. ^{1}H NMR ($CDCl_3$) spectrum of **2**.

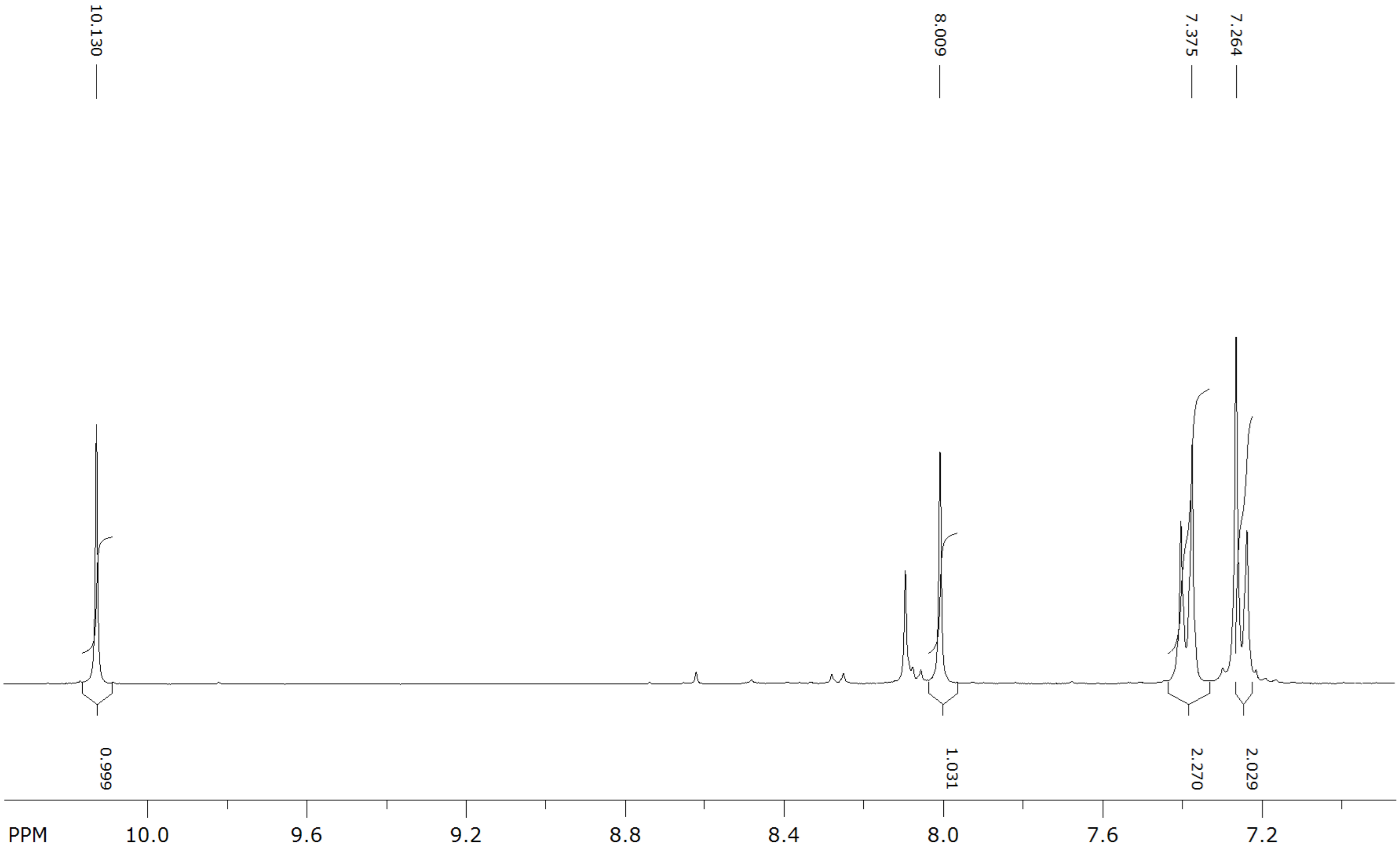

Figure S4. Part of the ^{1}H NMR ($CDCl_3$) spectrum of **2**.

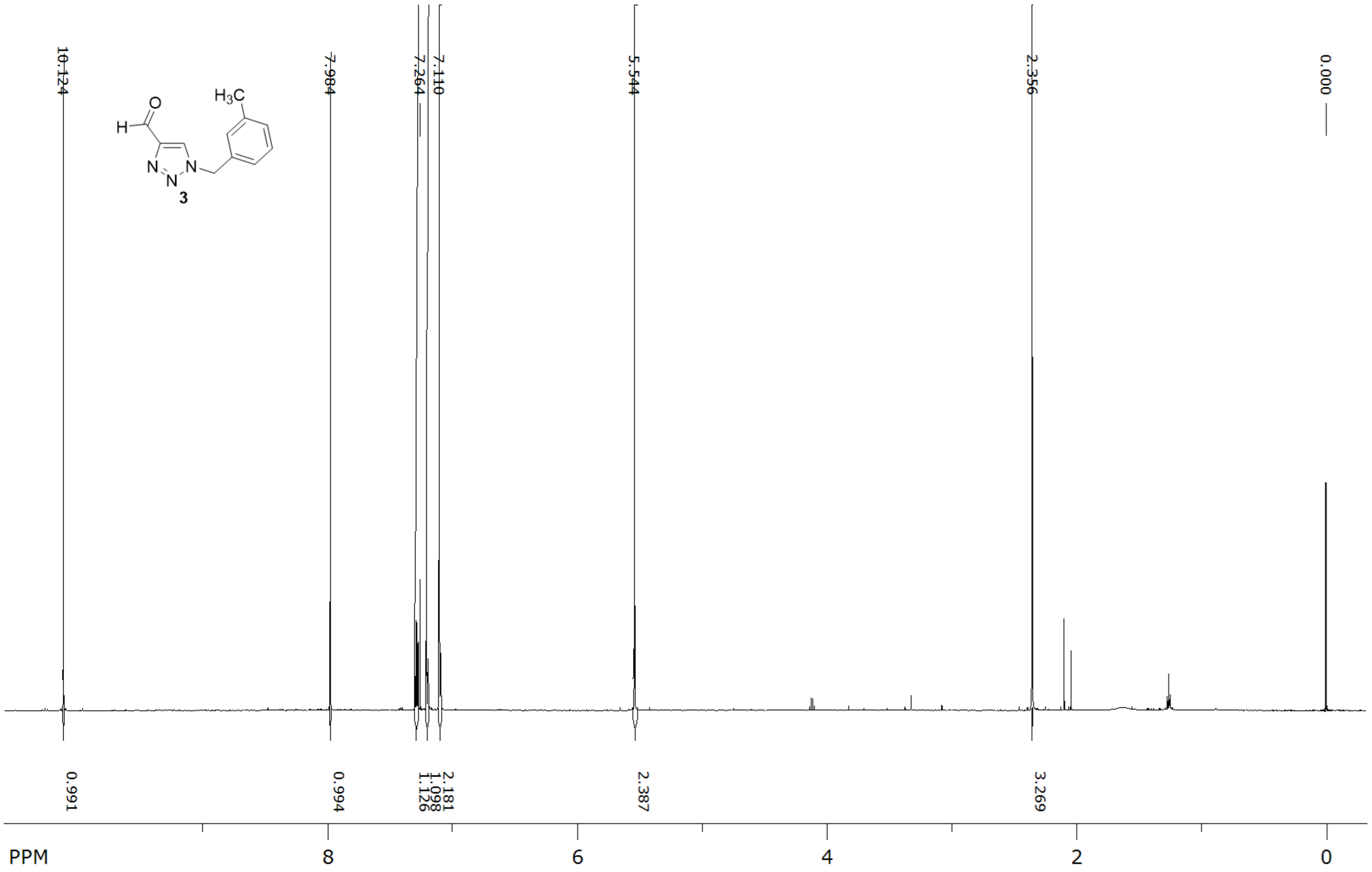

Figure S5. ^{1}H NMR ($CDCl_3$) spectrum of **3**.

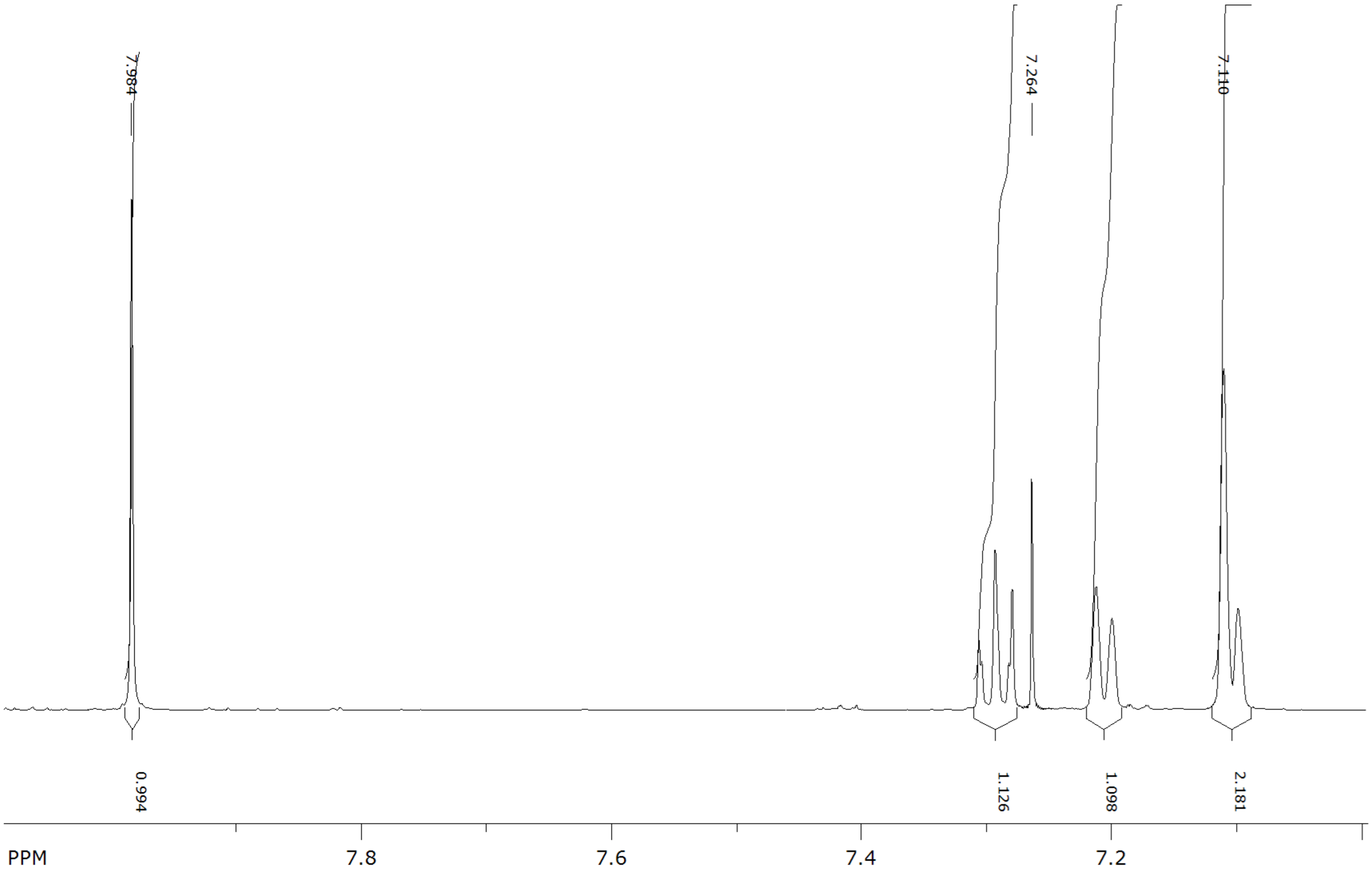

Figure S6. Part of the ^{1}H NMR ($CDCl_3$) spectrum of **3**.

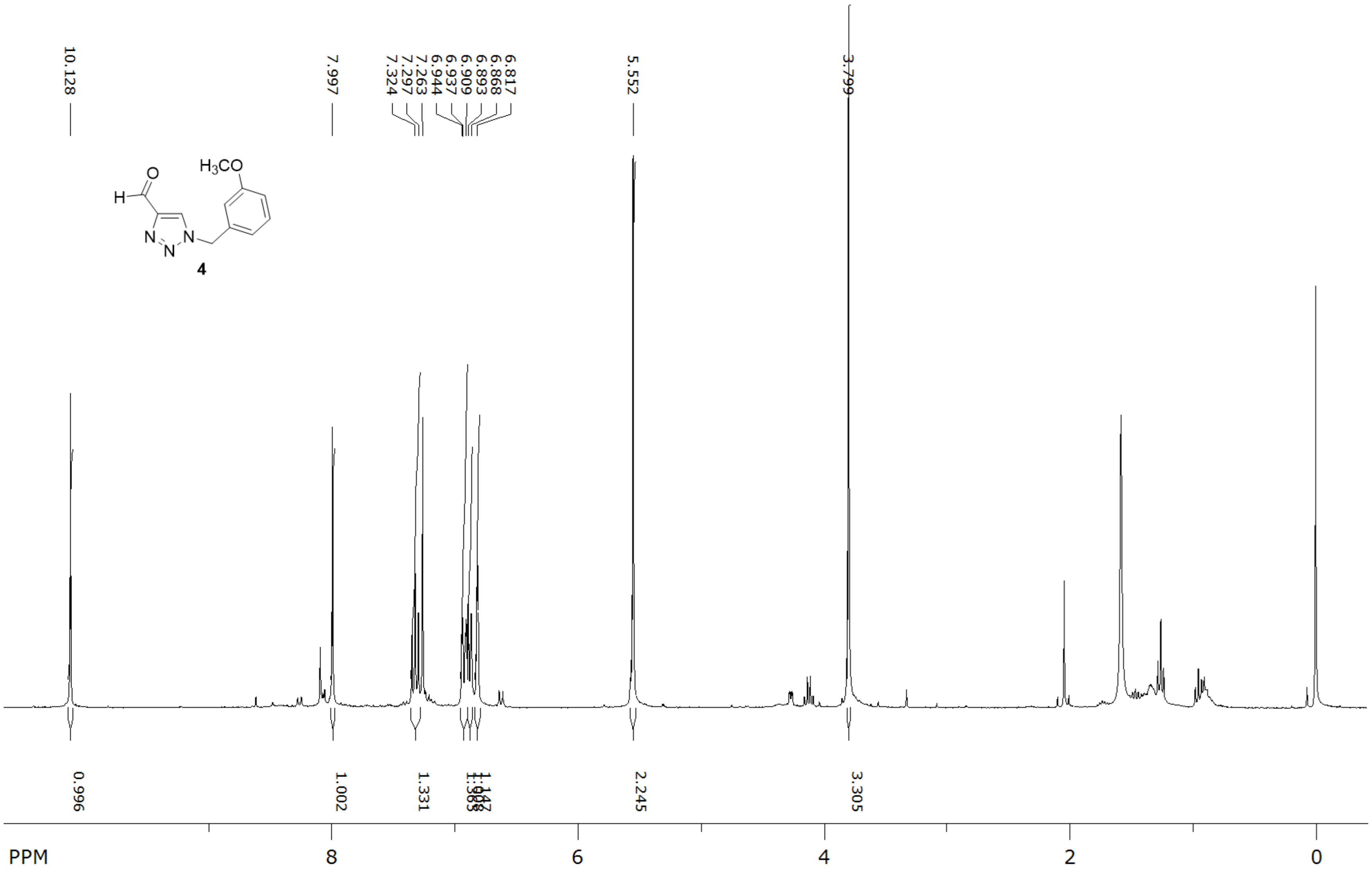

Figure S7. ^{1}H NMR ($CDCl_3$) spectrum of **4**.

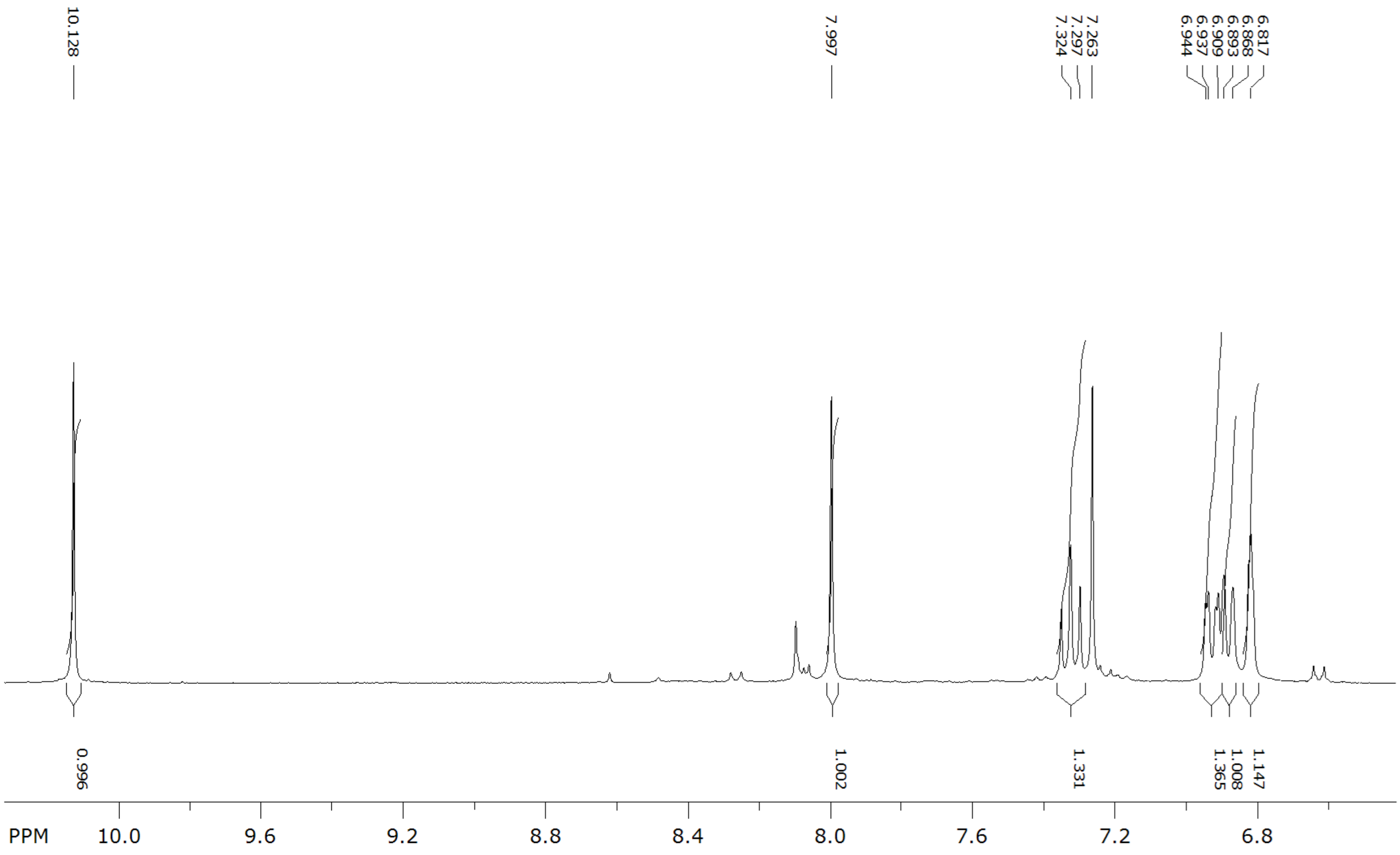

Figure S8. Part of the ^{1}H NMR ($CDCl_3$) spectrum of **4**.

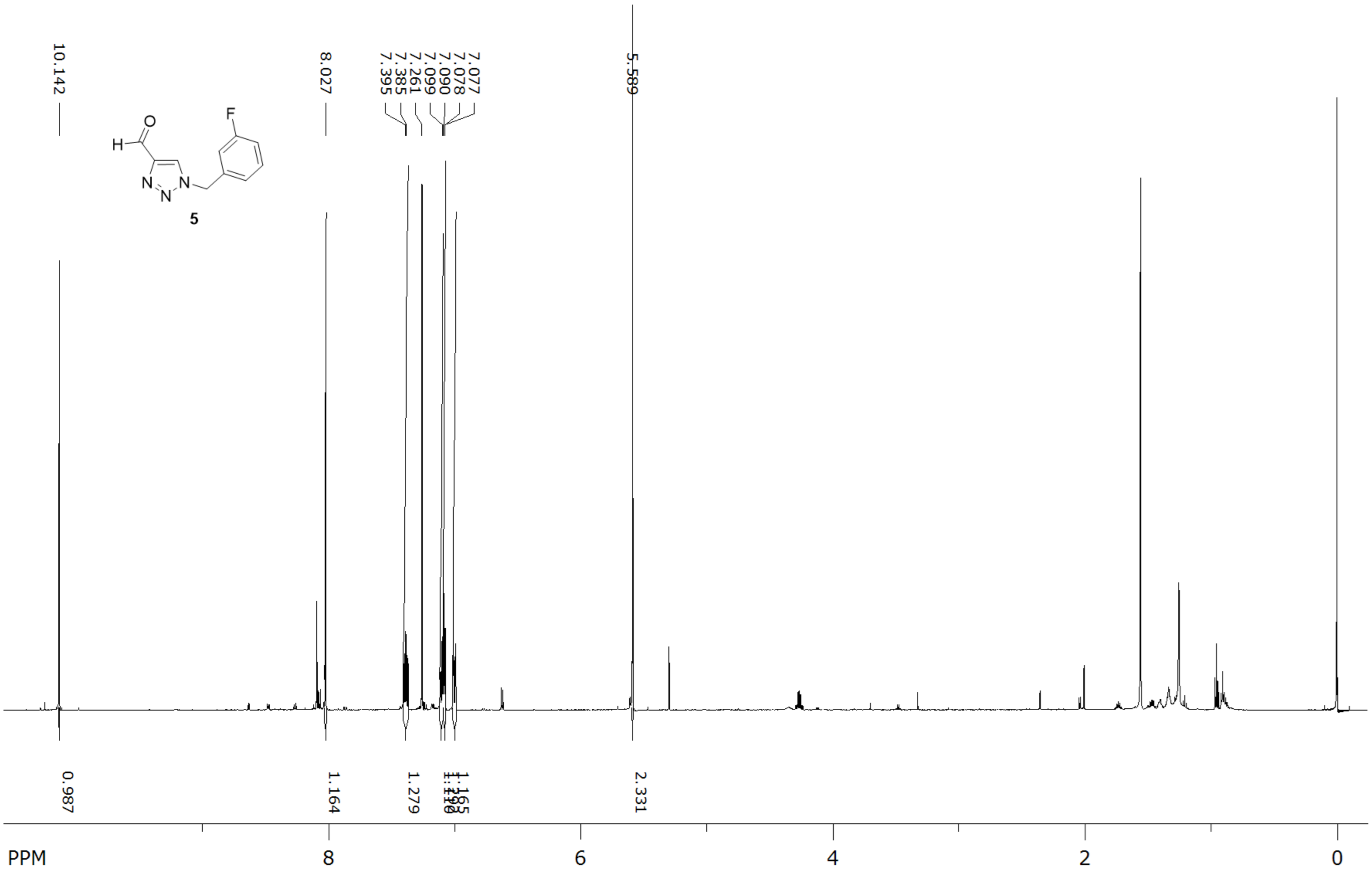

Figure S9. ^{1}H NMR ($CDCl_3$) spectrum of **5**.

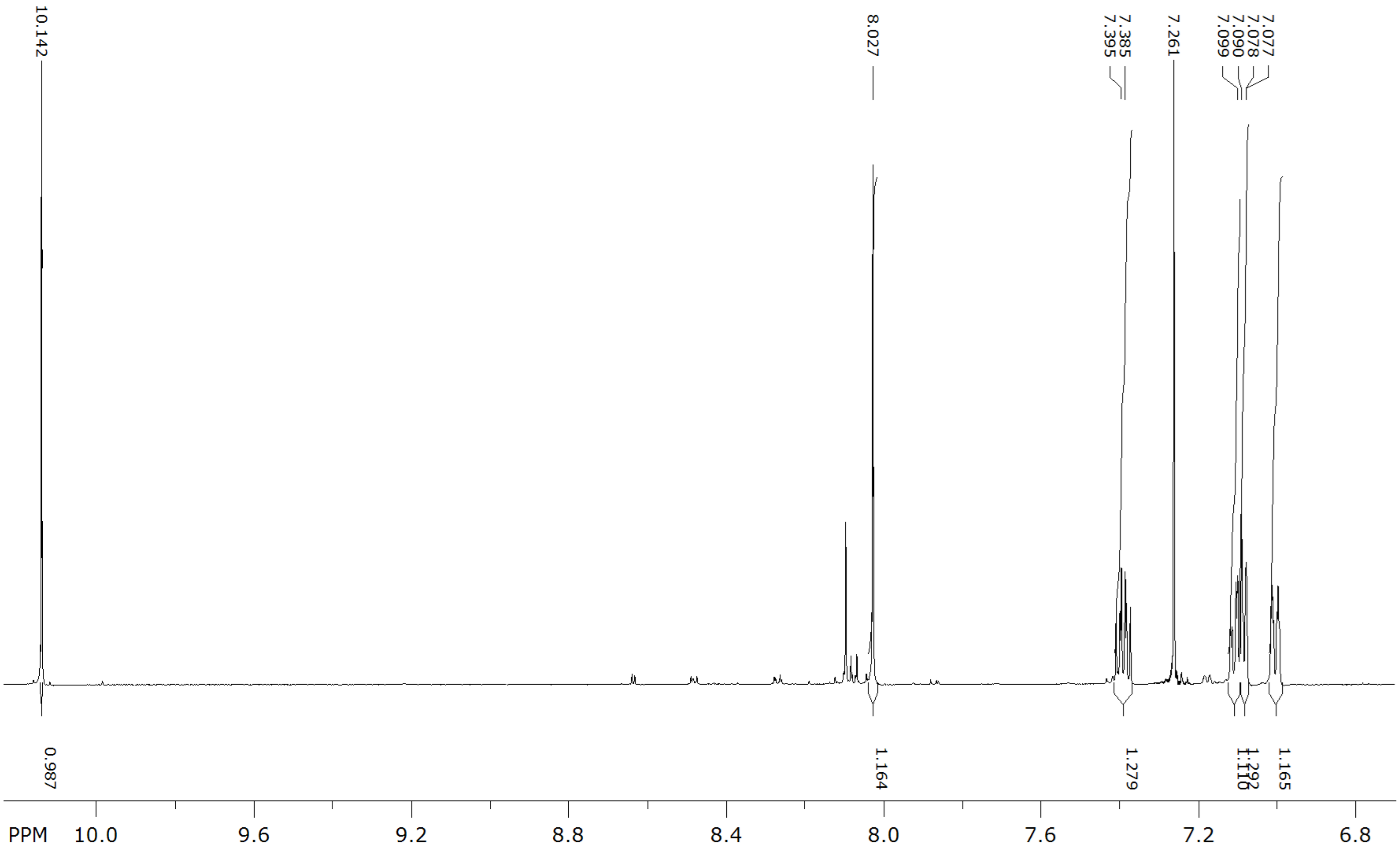

Figure S10. Part of the ^1H NMR ($CDCl_3$) spectrum of **5**.

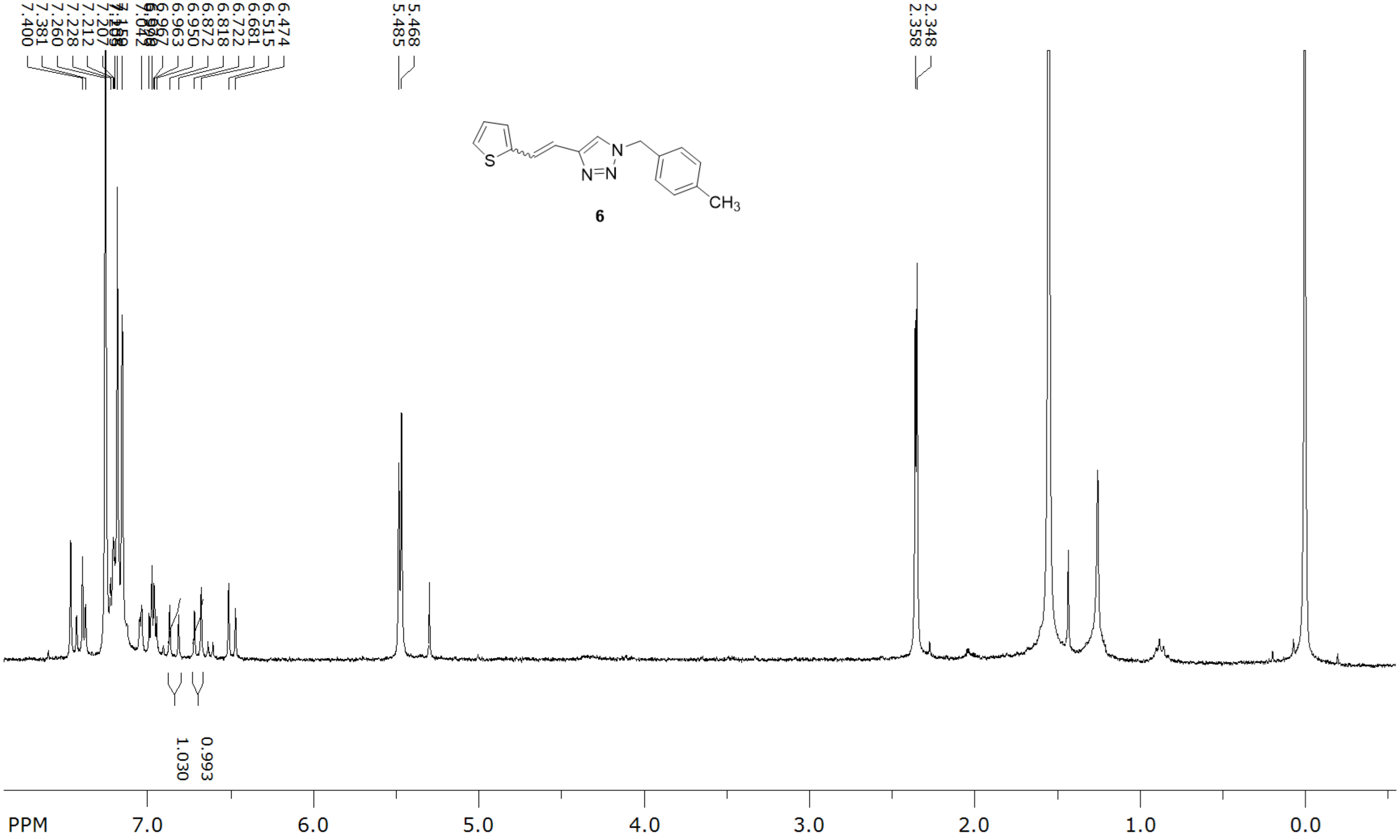

Figure S11. ^{1}H NMR ($CDCl_3$) spectrum of a mixture of isomers of **6**.

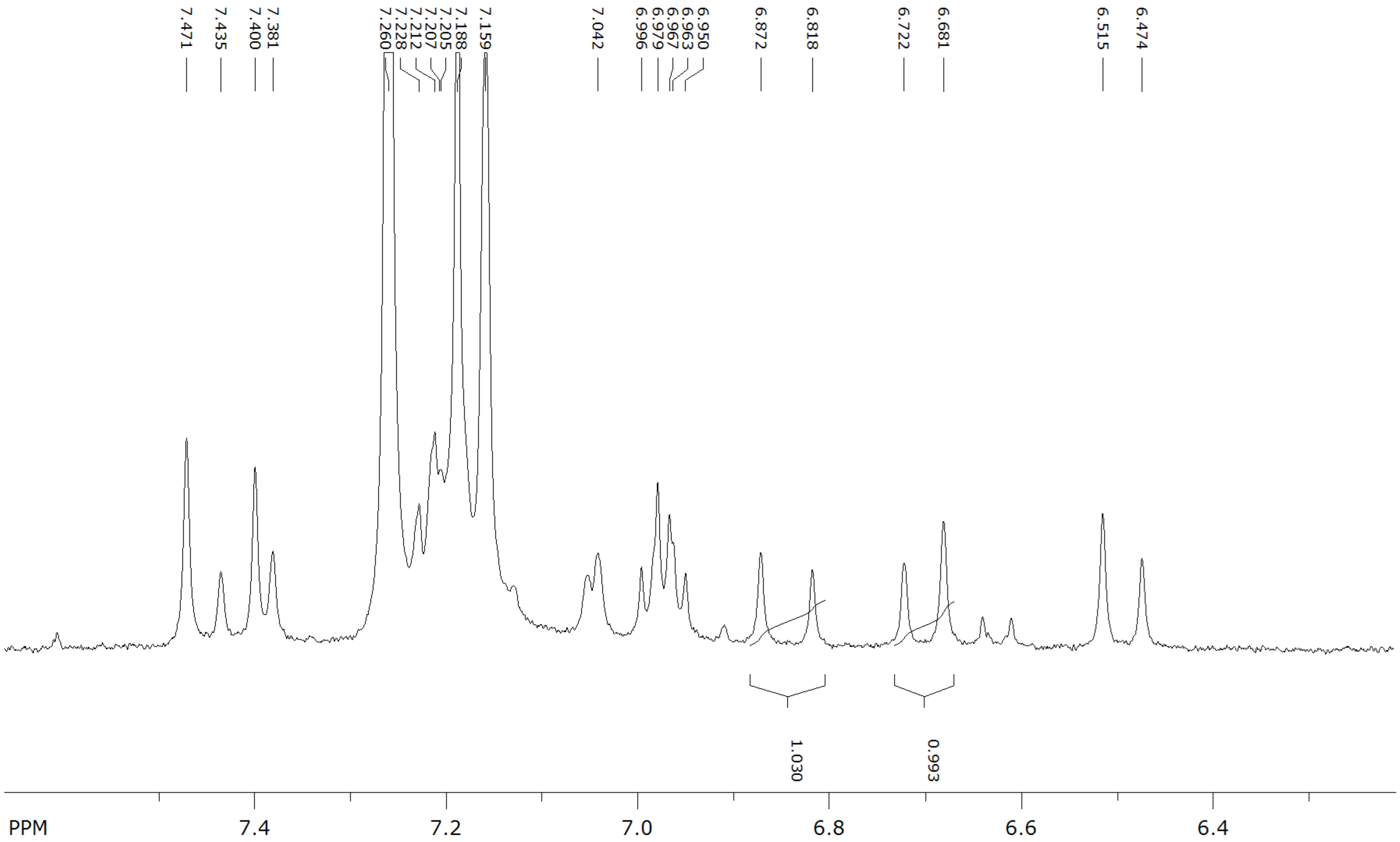

Figure S12. Unsaturated part of the ^{1}H NMR ($CDCl_3$) spectrum of a mixture of isomers of **6**.

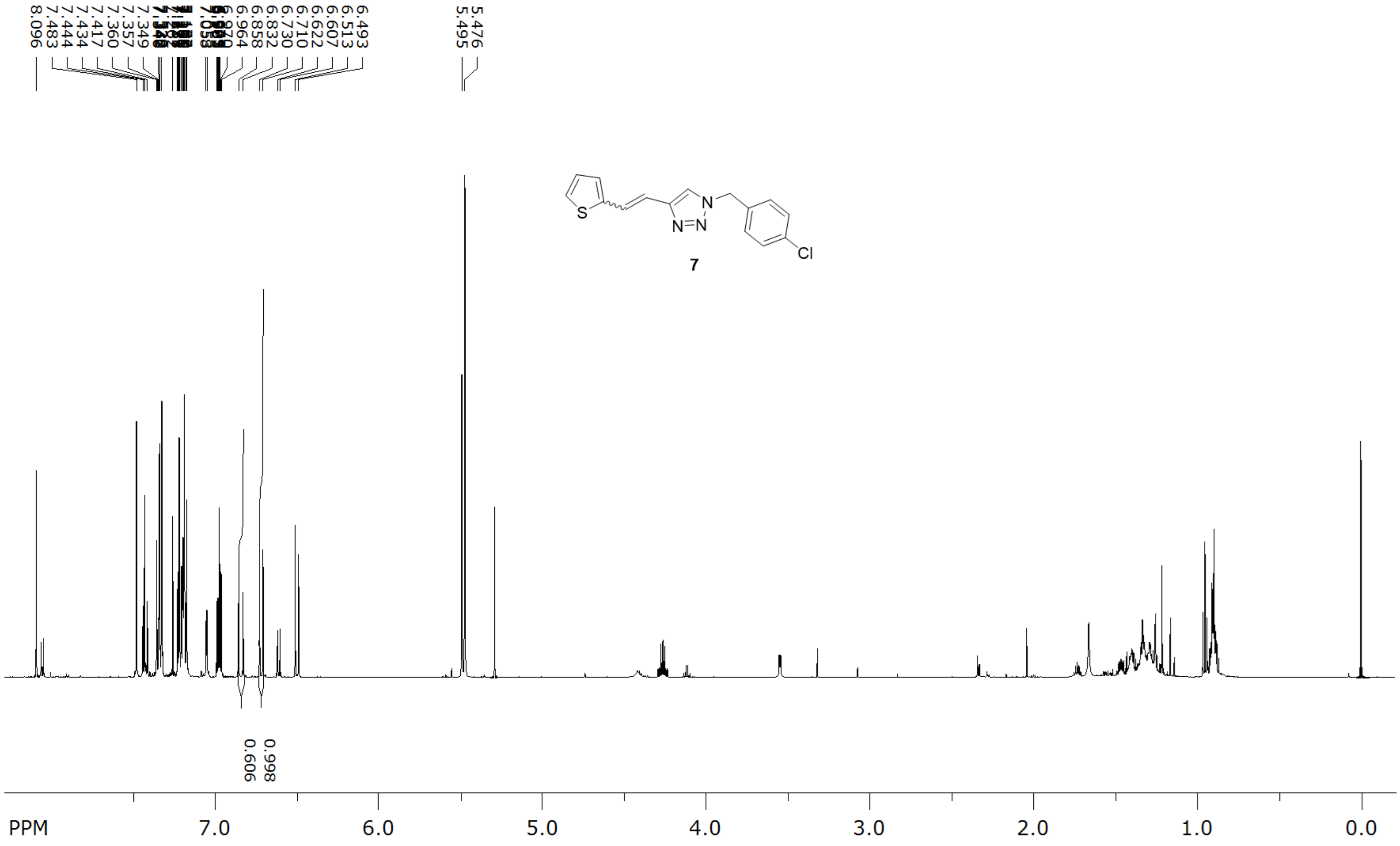

Figure S13. ^{1}H NMR ($CDCl_3$) spectrum of a mixture of isomers of **7**.

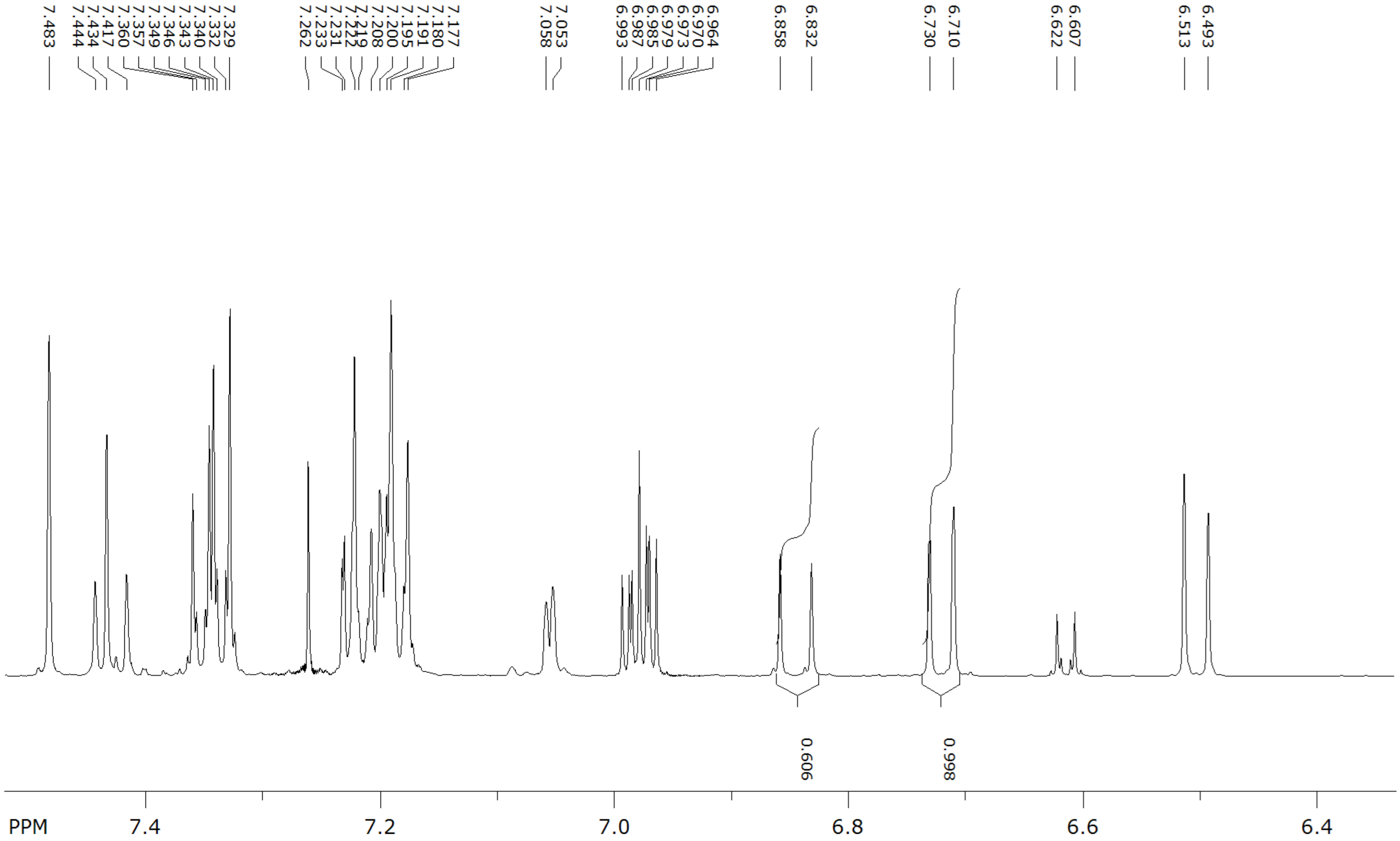

Figure S14. Unsaturated part of the ^1H NMR ($CDCl_3$) spectrum of a mixture of isomers of **7**.

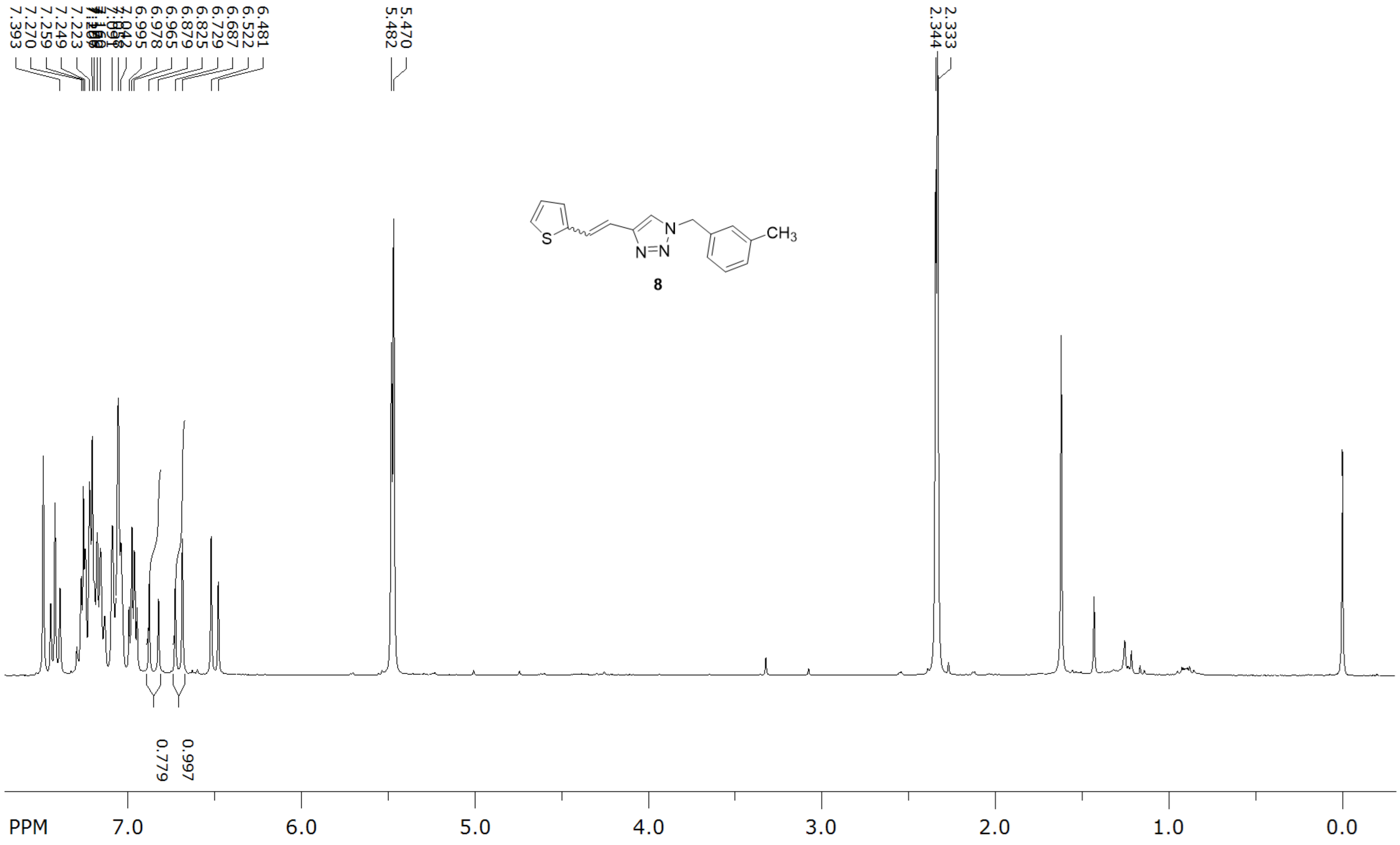

Figure S15. ^{1}H NMR ($CDCl_3$) spectrum of a mixture of isomers of **8**.

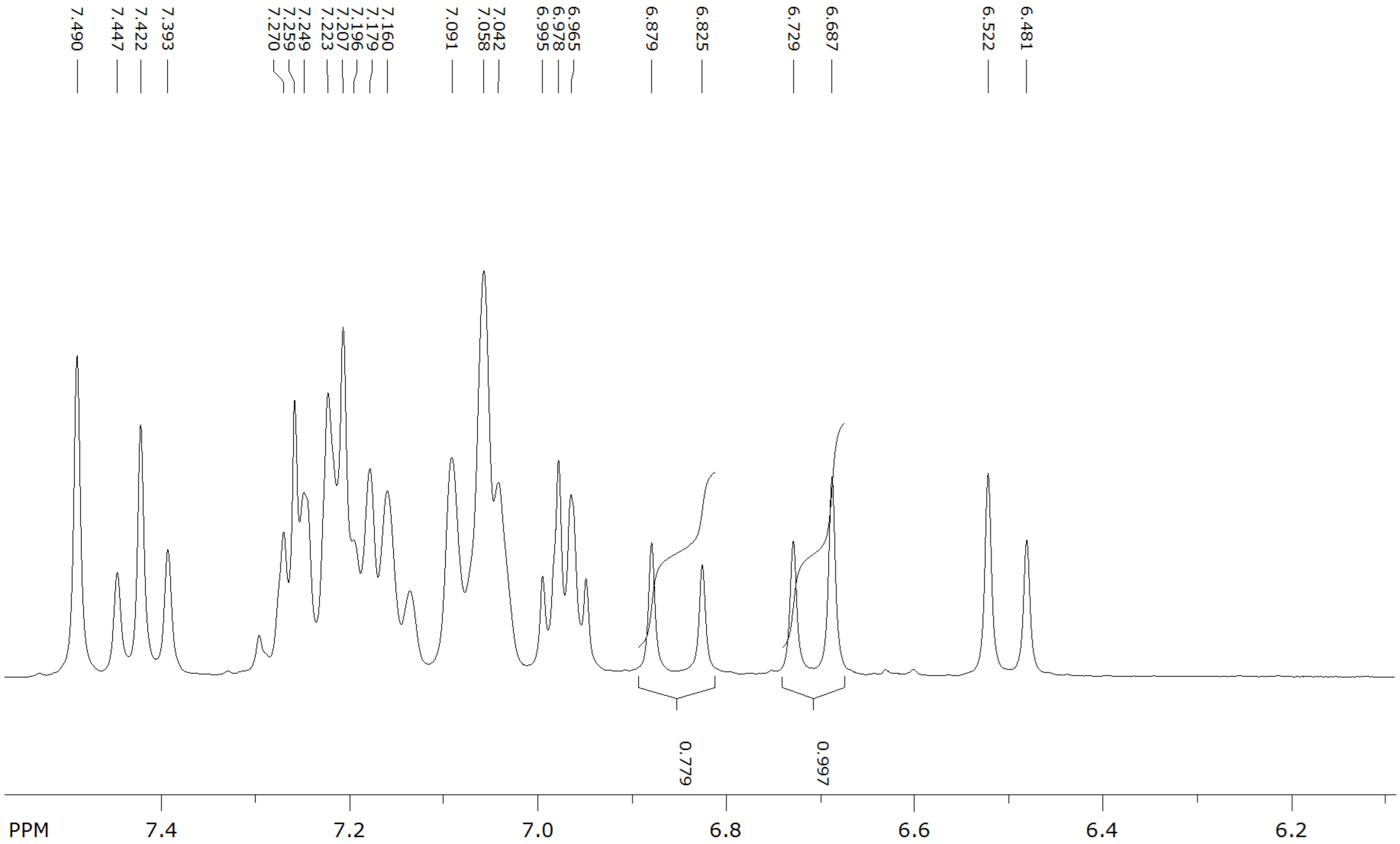

Figure S16. Unsaturated part of the ^{1}H NMR ($CDCl_3$) spectrum of a mixture of isomers of **8**.

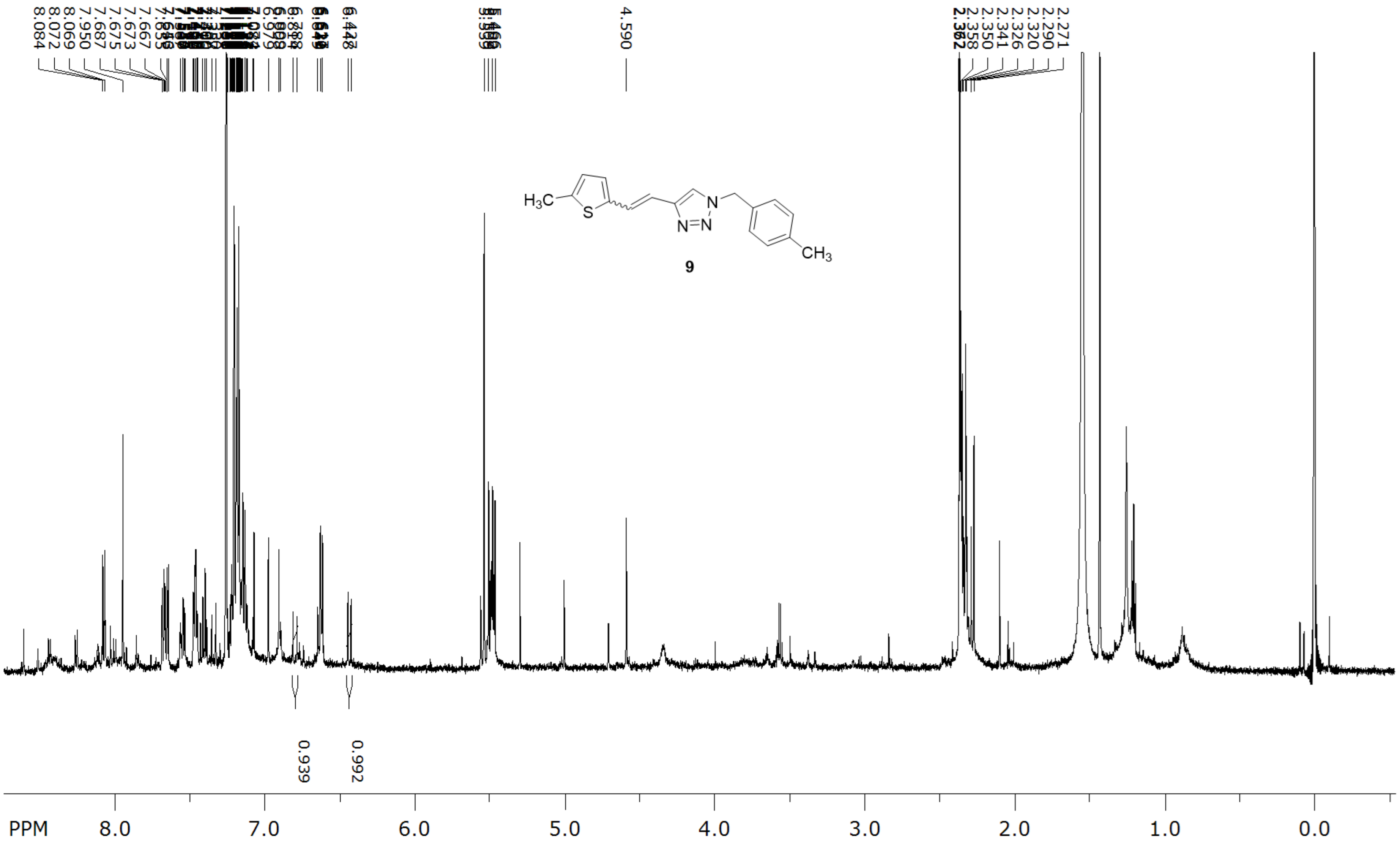

Figure S17. ^{1}H NMR ($CDCl_3$) spectrum of a mixture of isomers of **9**.

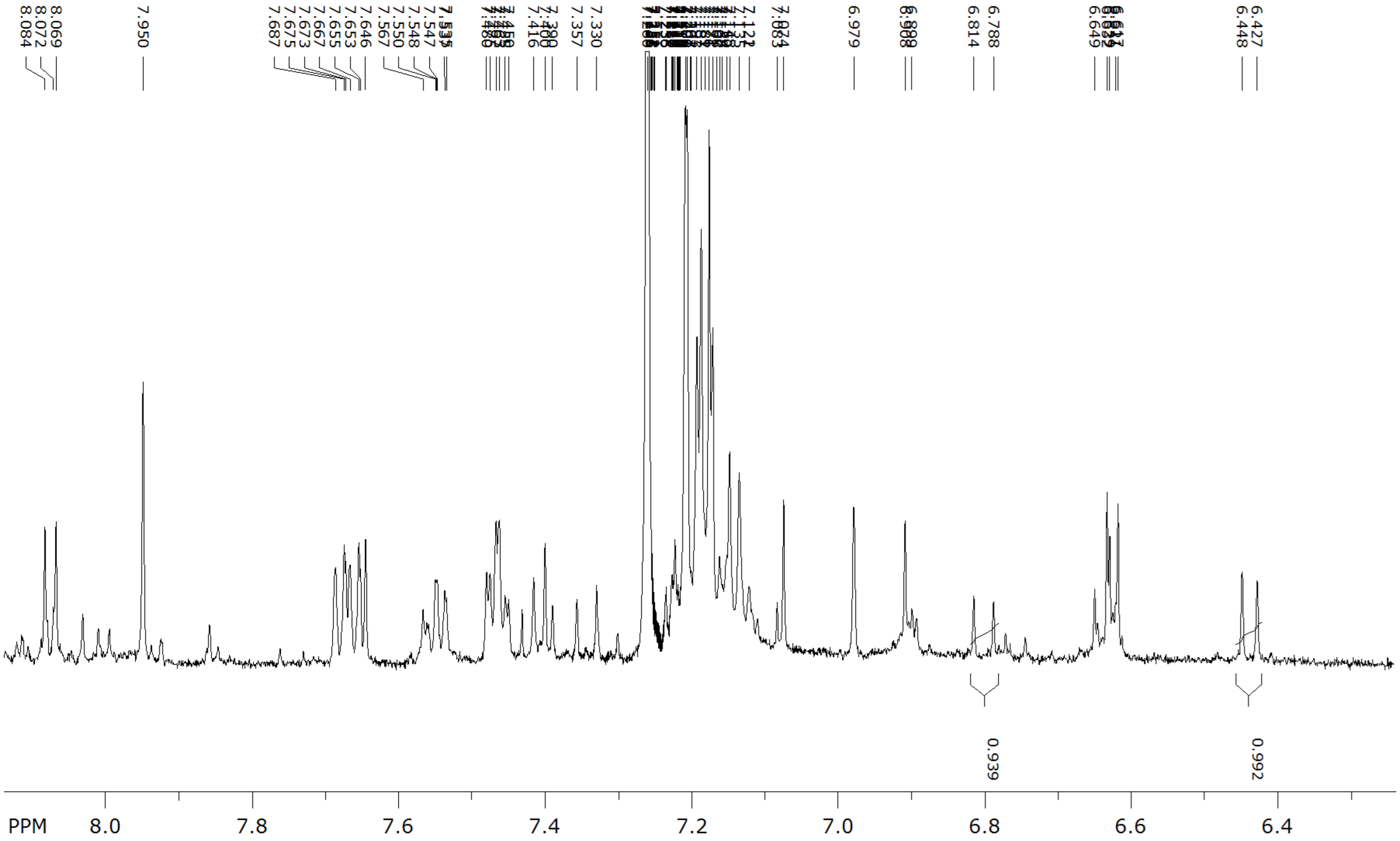

Figure S18. Unsaturated part of the ^{1}H NMR ($CDCl_3$) spectrum of a mixture of isomers of **9**.

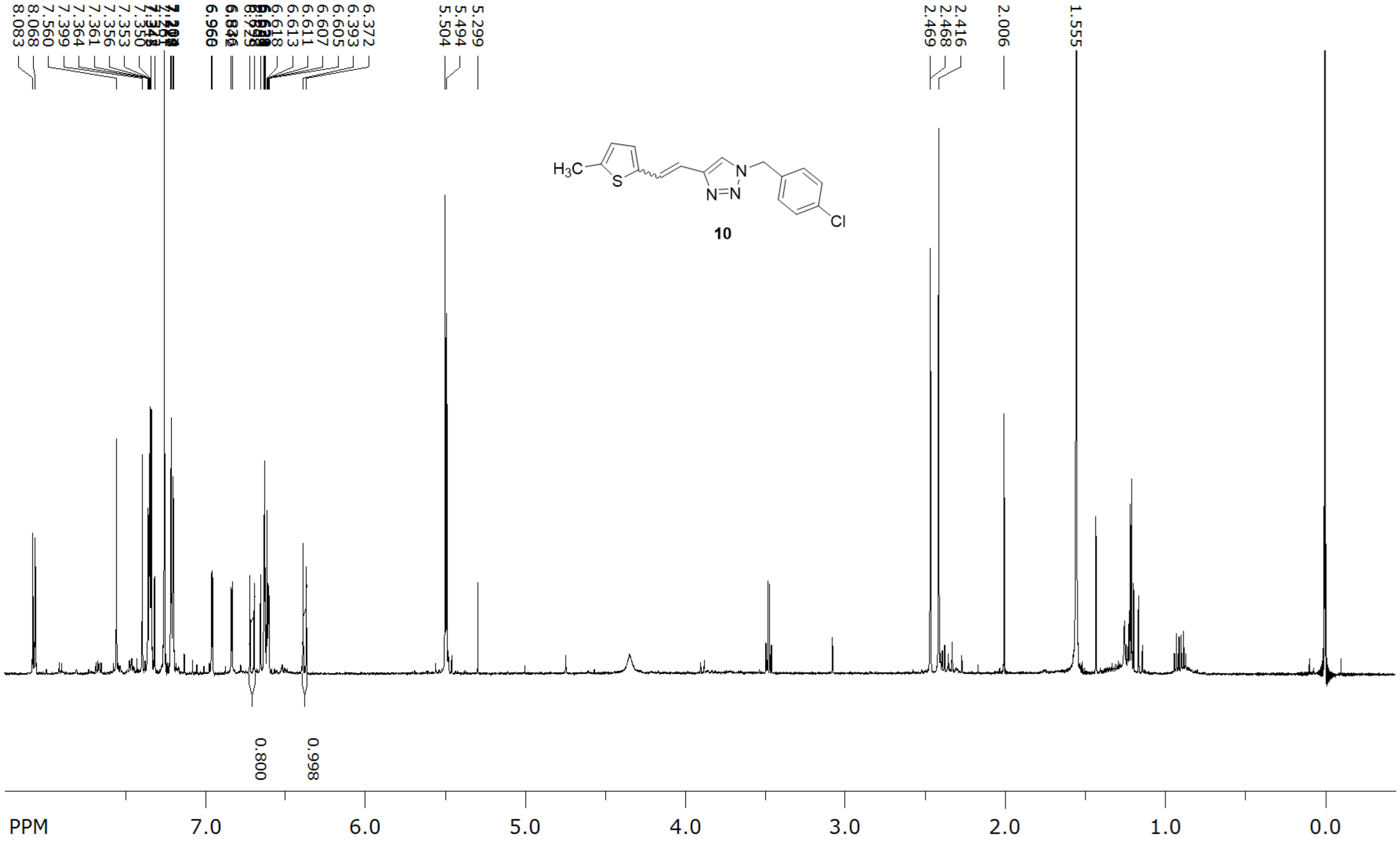

Figure S19. ^{1}H NMR ($CDCl_3$) spectrum of a mixture of isomers of **10**.

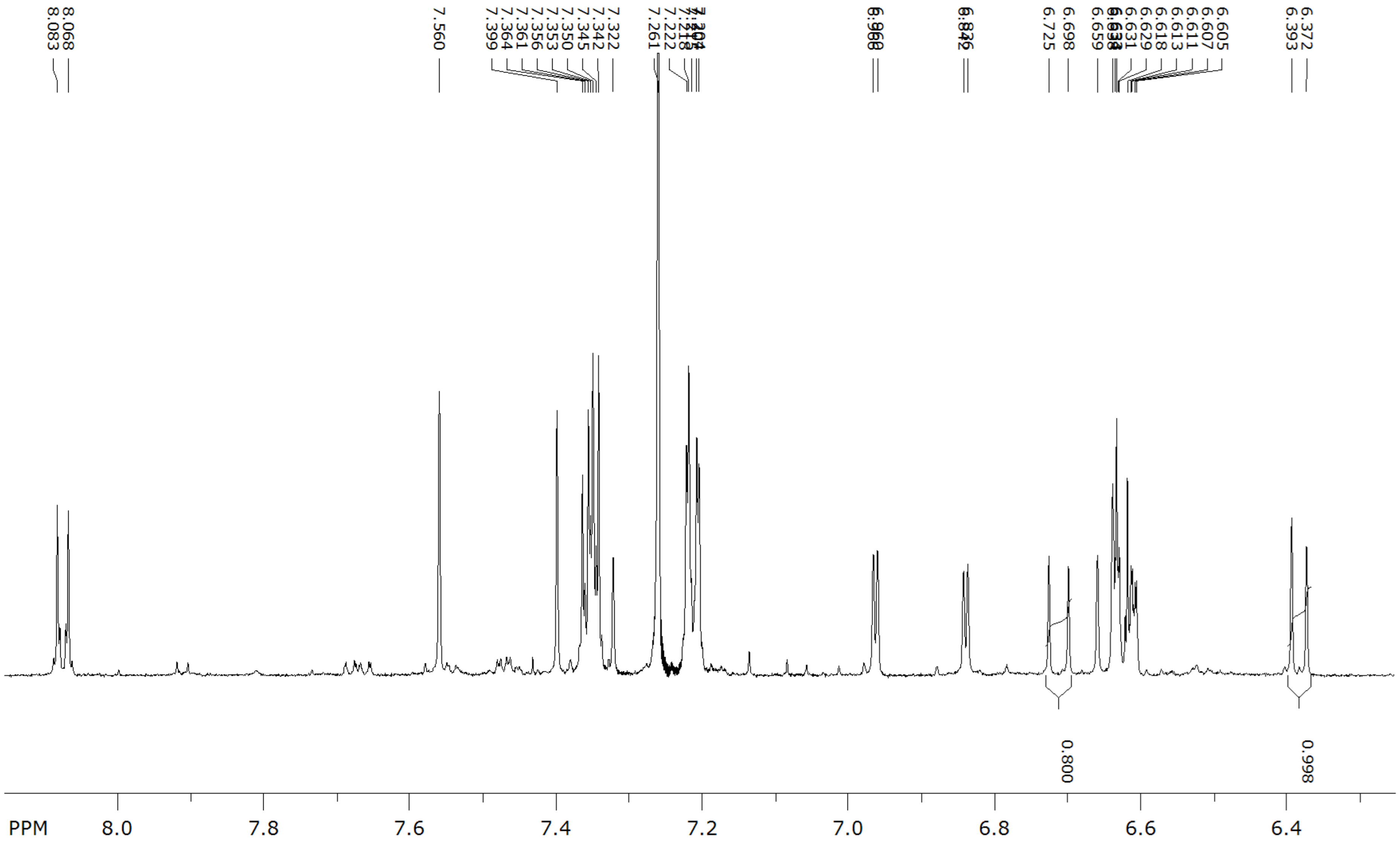

Figure S20. Unsaturated part of the ^1H NMR ($CDCl_3$) spectrum of a mixture of isomers of **10**.

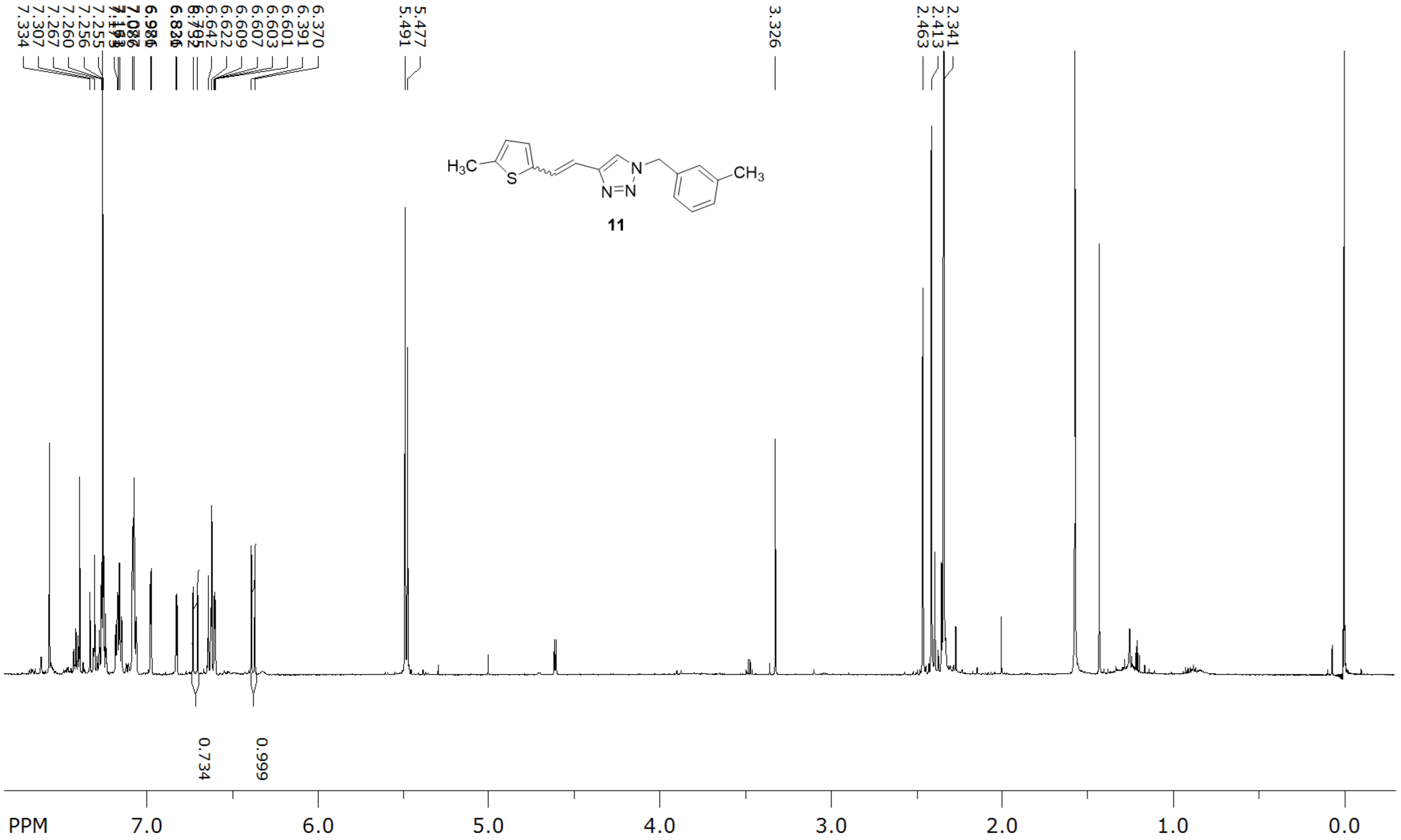

Figure S21. ¹H NMR ($CDCl_3$) spectrum of a mixture of isomers of **11**.

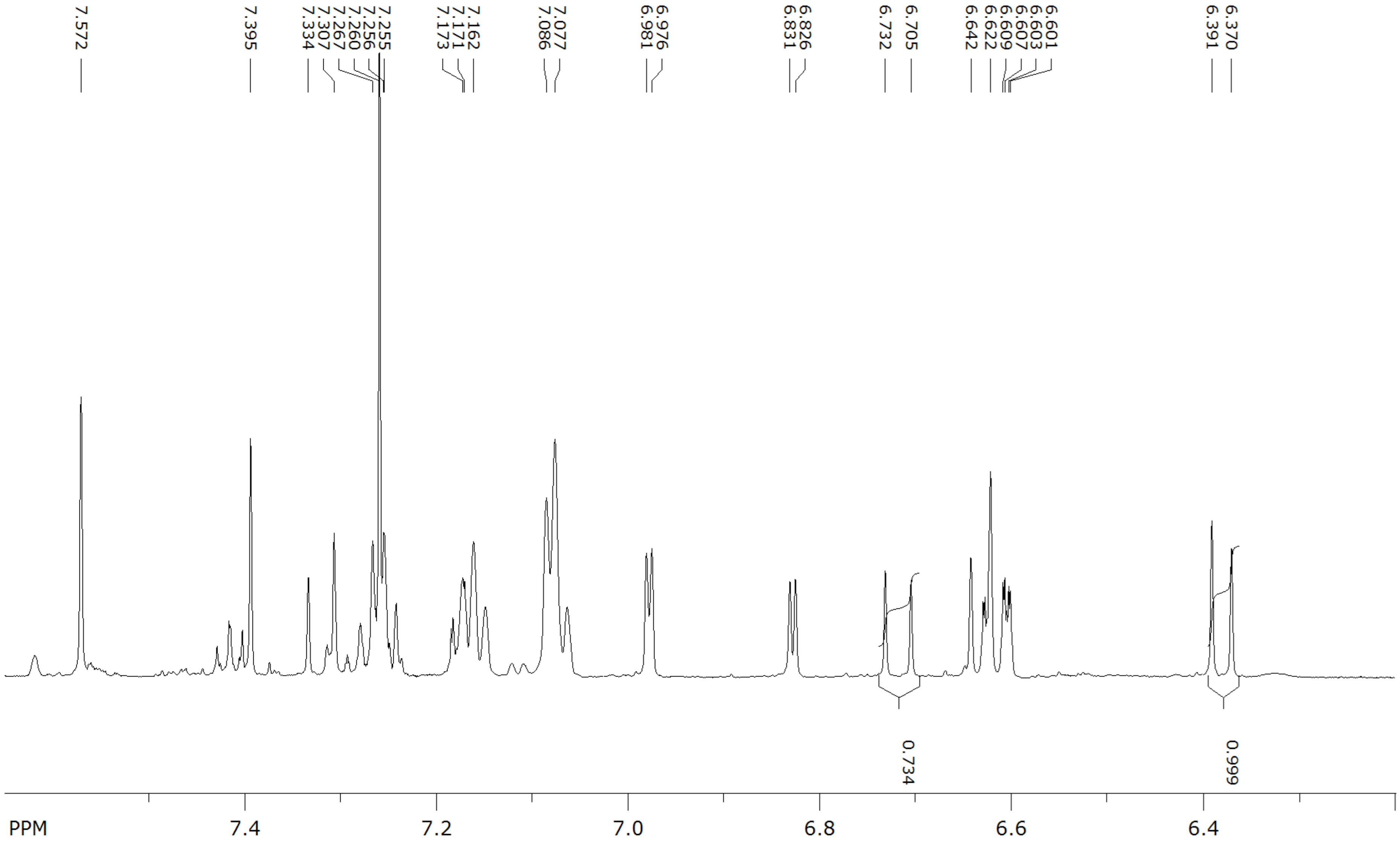

Figure S22. Unsaturated part of the ^{1}H NMR ($CDCl_3$) spectrum of a mixture of isomers of **11**.

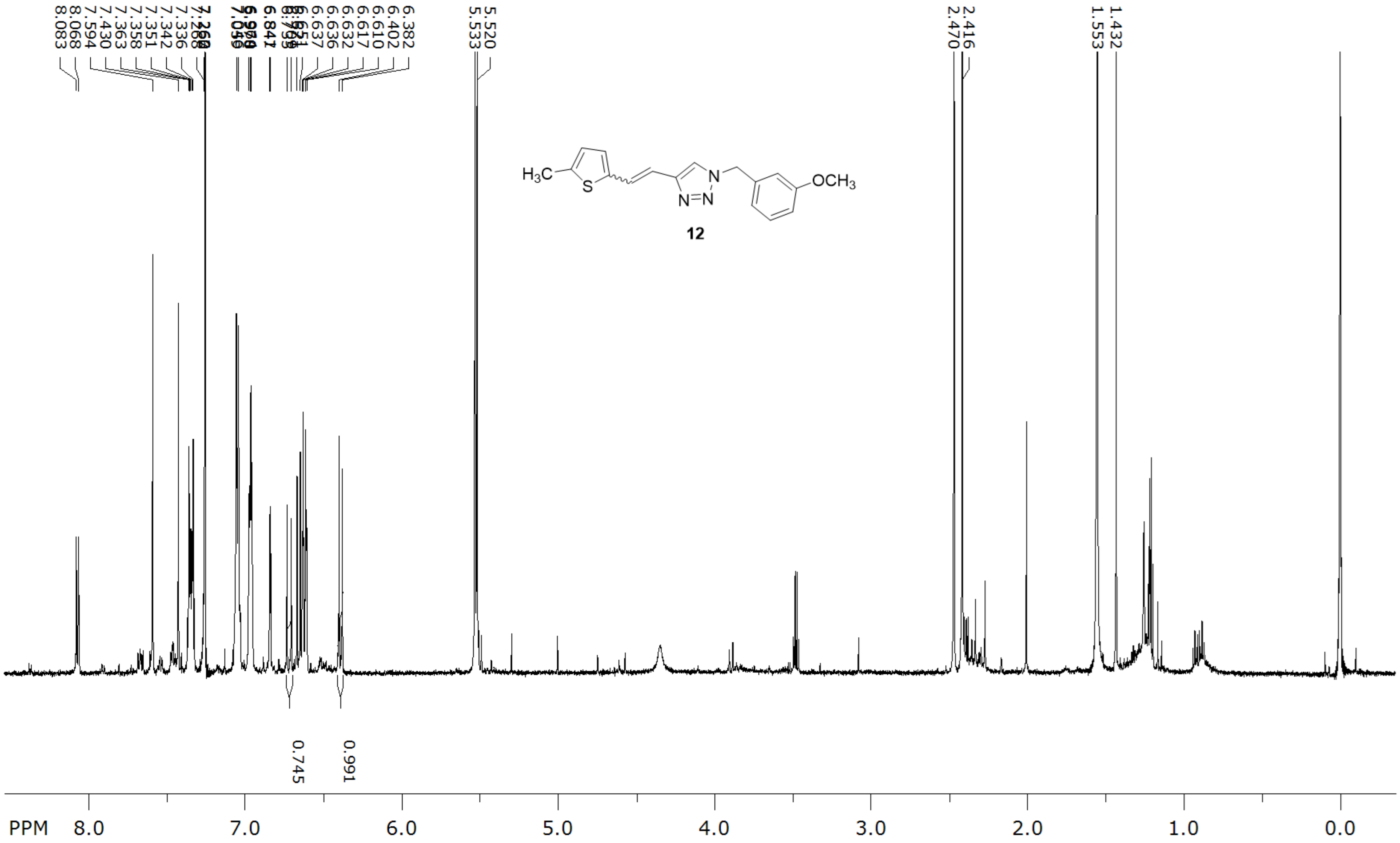

Figure S23. ^{1}H NMR ($CDCl_3$) spectrum of a mixture of isomers of **12**.

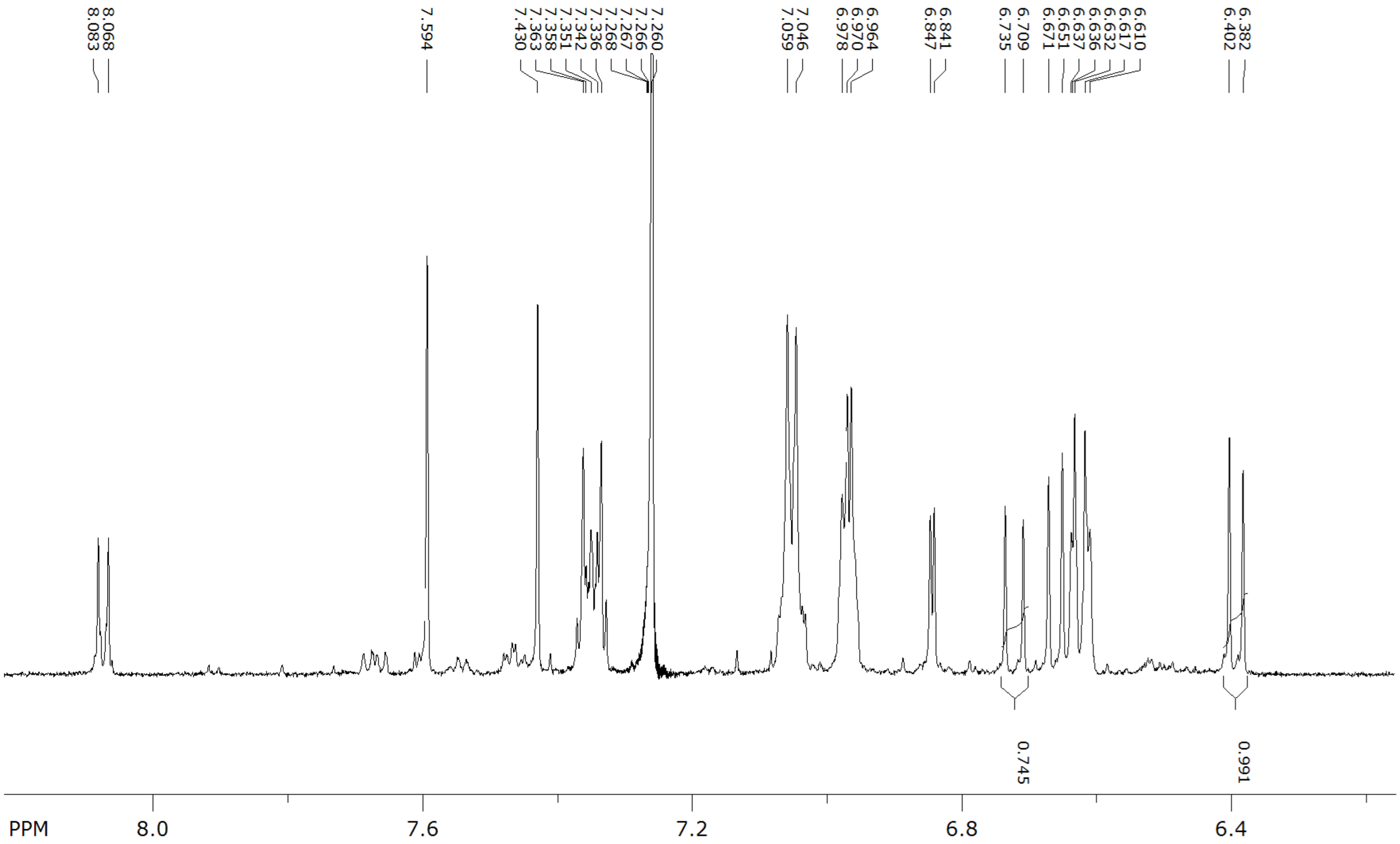

Figure S24. Unsaturated part of the ^{1}H NMR ($CDCl_3$) spectrum of a mixture of isomers of **12**.

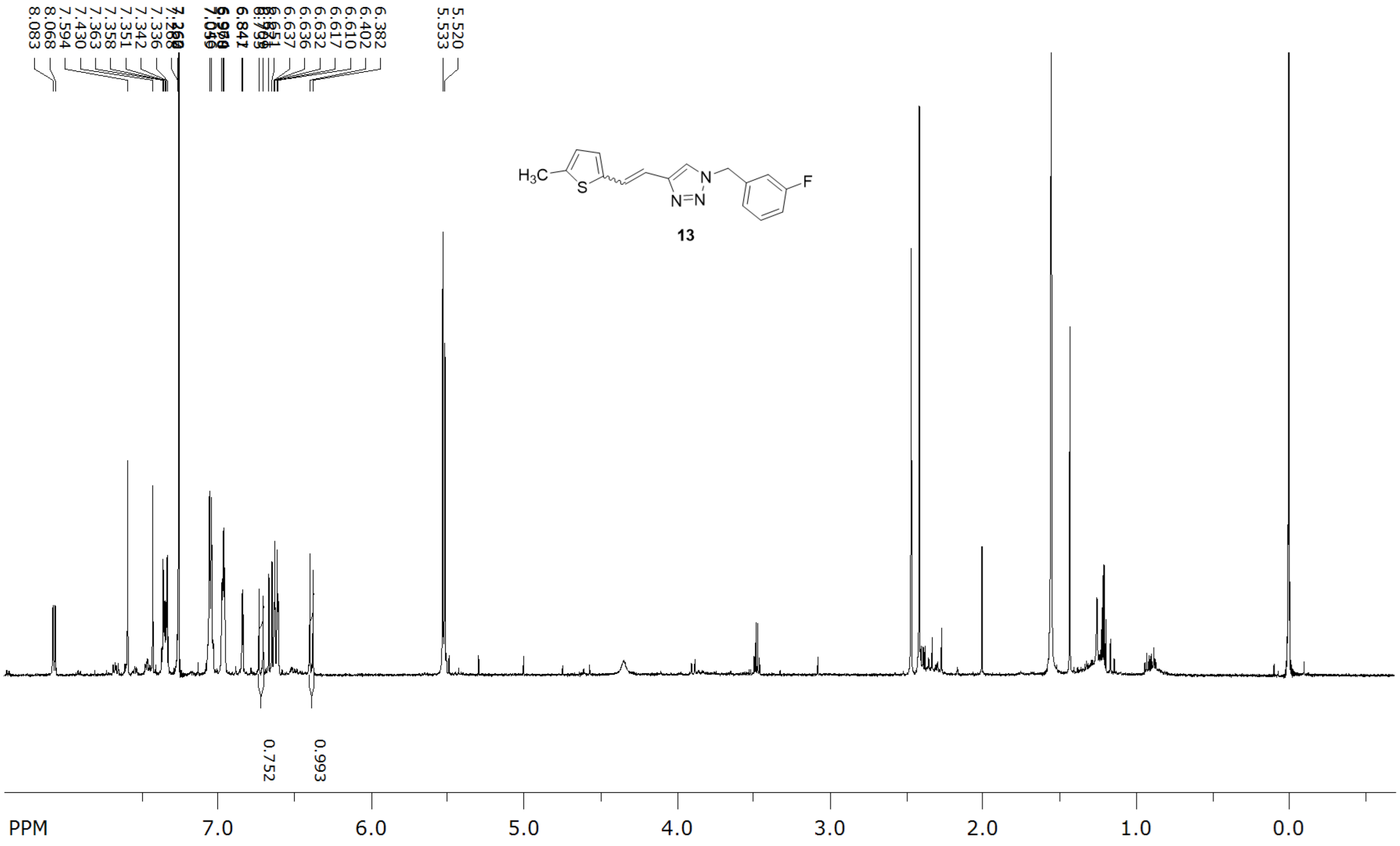

Figure S25. ^{1}H NMR ($CDCl_3$) spectrum of a mixture of isomers of **13**.

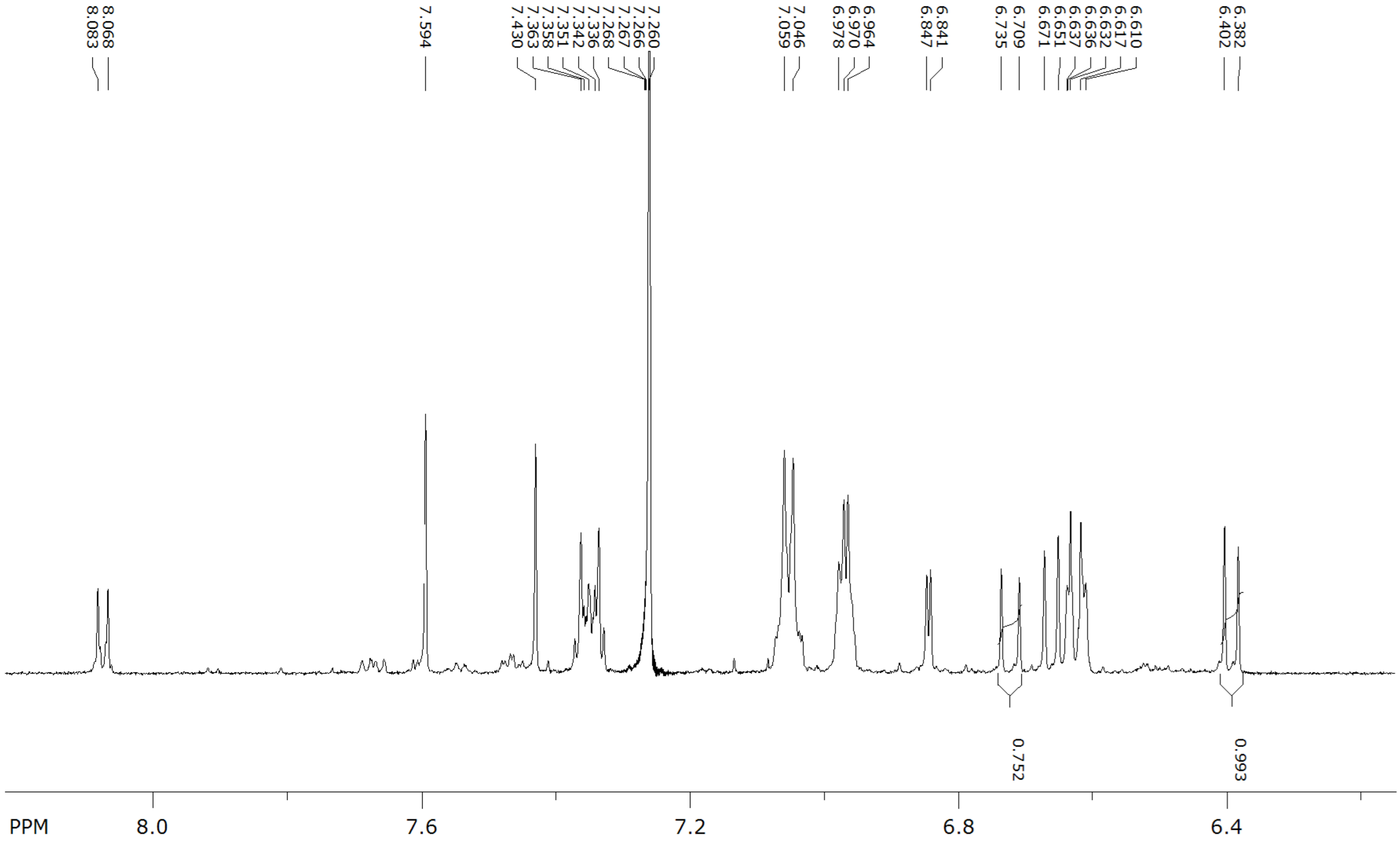

Figure S26. Unsaturated part of the ^{1}H NMR ($CDCl_3$) spectrum of a mixture of isomers of **13**.

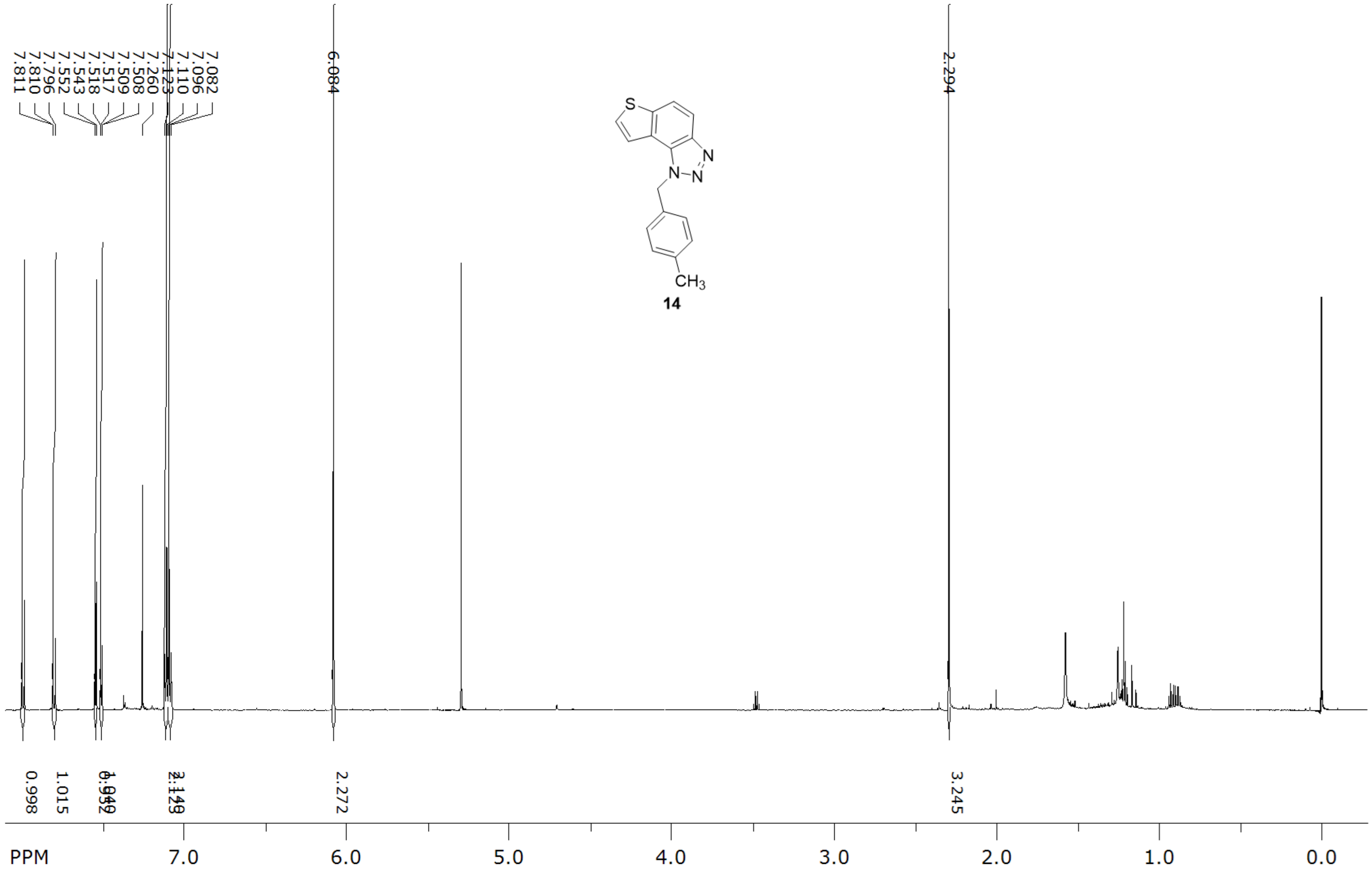

Figure S27. ^{1}H NMR ($CDCl_3$) spectrum of **14**.

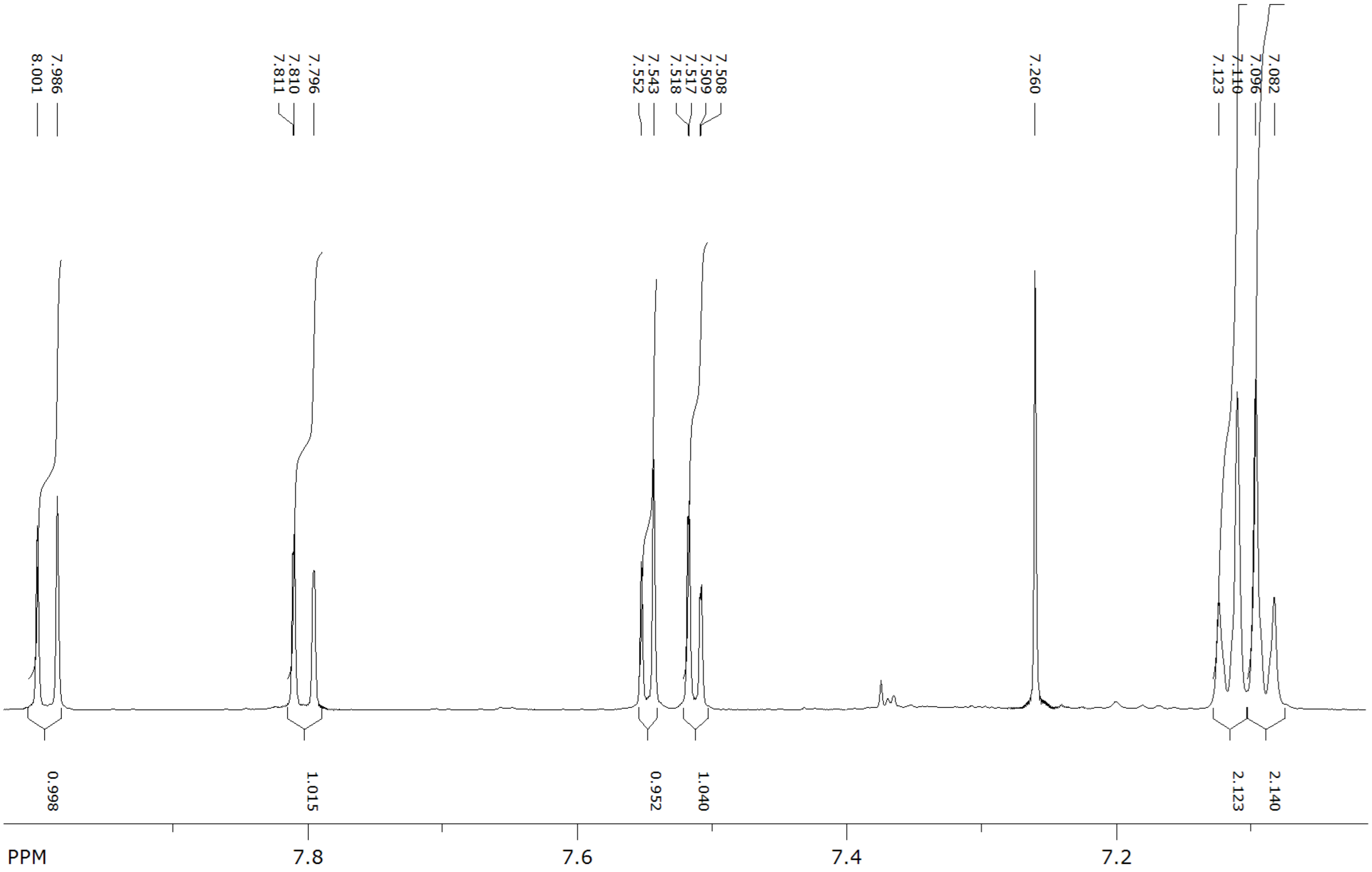

Figure S28. Aromatic part of the ^{1}H NMR ($CDCl_3$) spectrum of **14**.

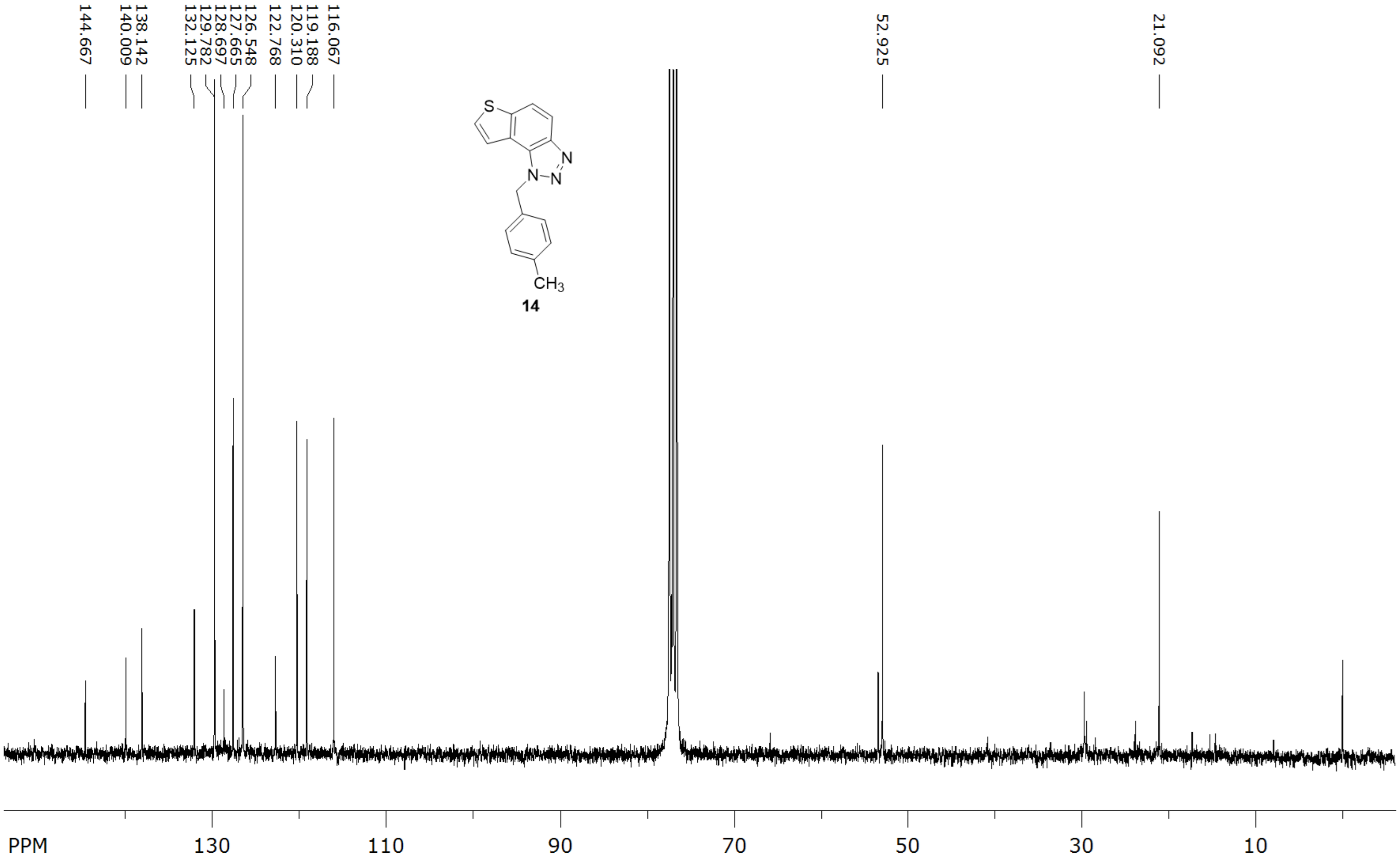

Figure S29. ¹³C NMR ($CDCl_3$) spectrum of **14**.

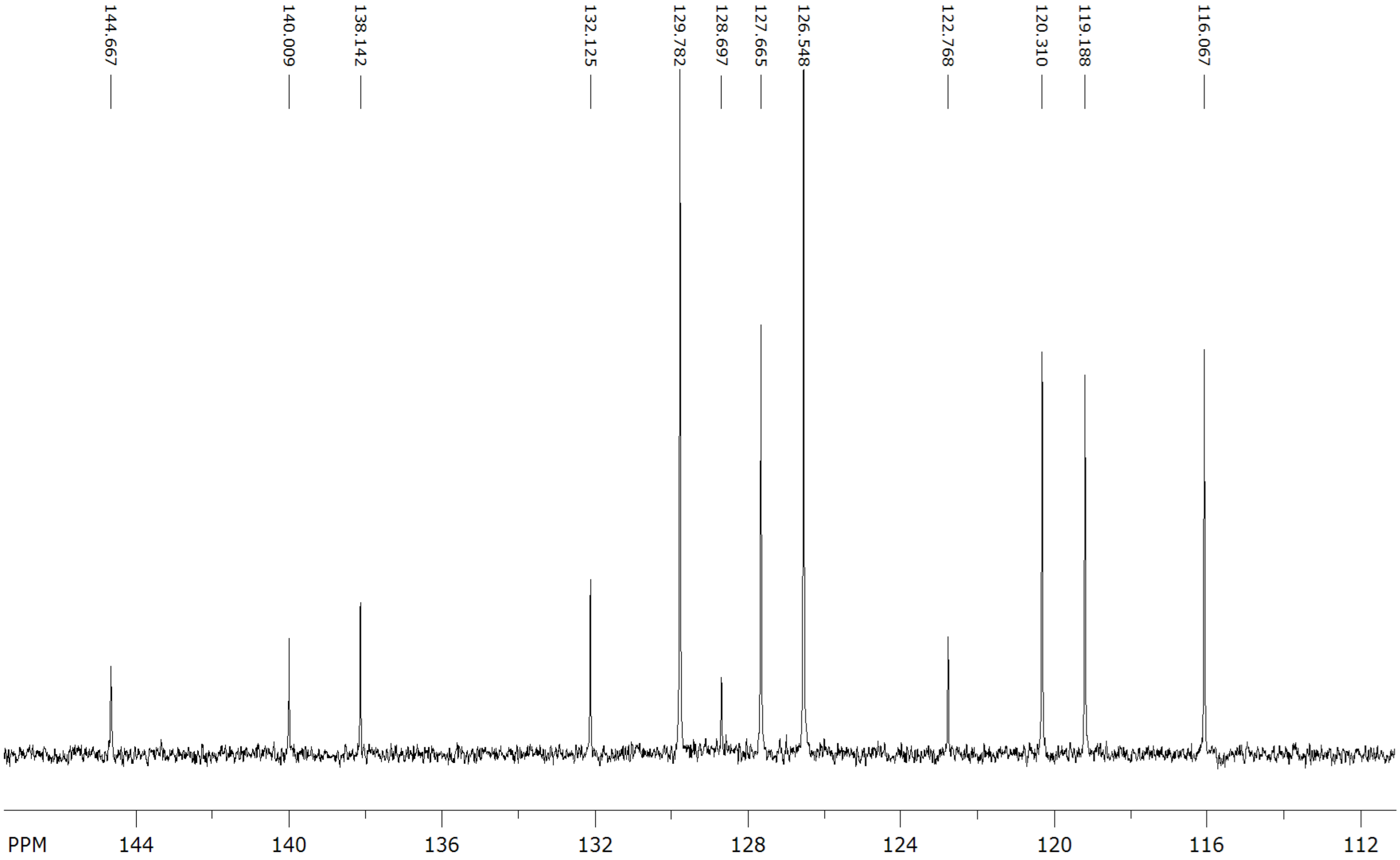

Figure S30. Aromatic part of the ^{13}C NMR ($CDCl_3$) spectrum of **14**.

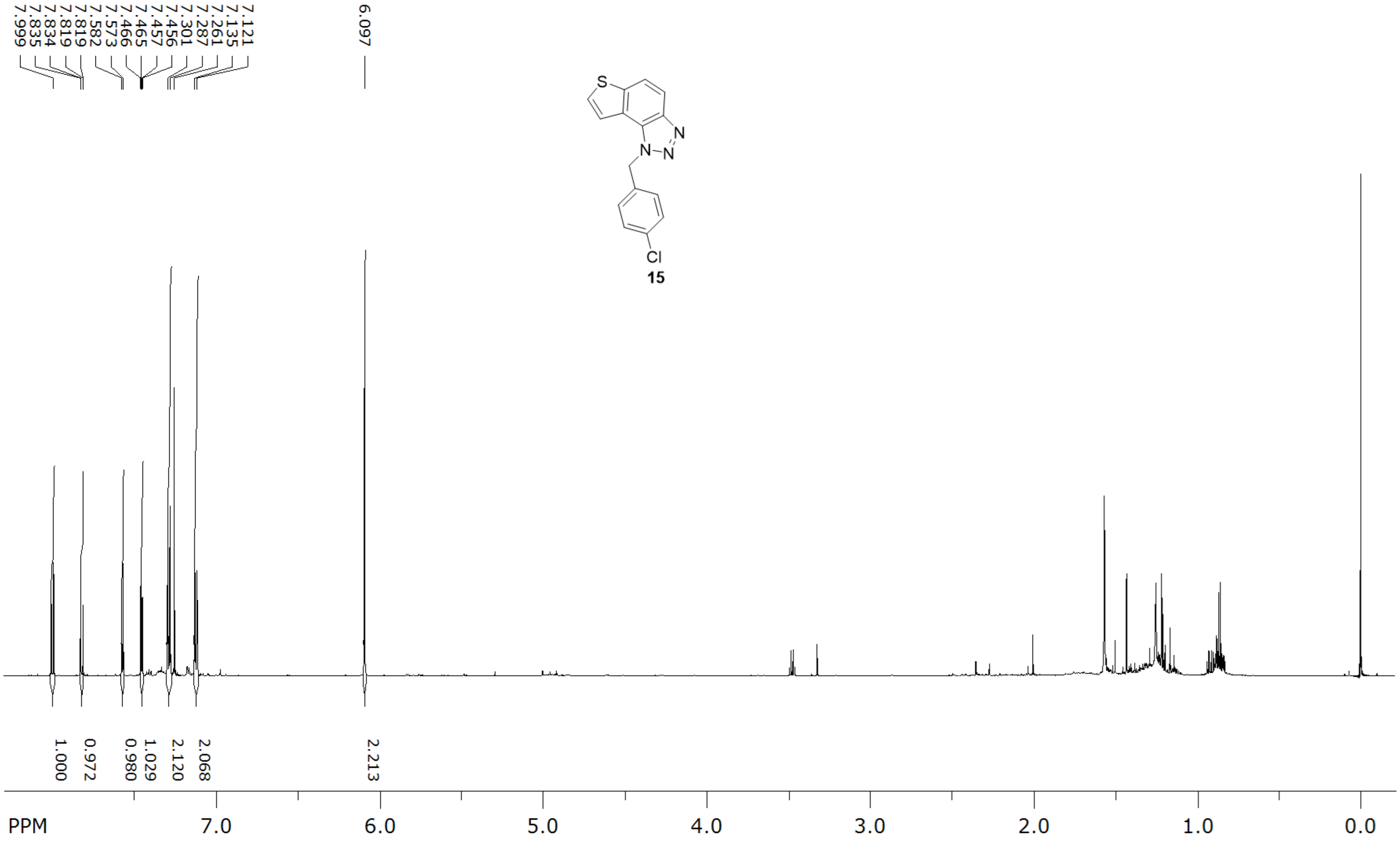

Figure S31. ^{1}H NMR ($CDCl_3$) spectrum of **15**.

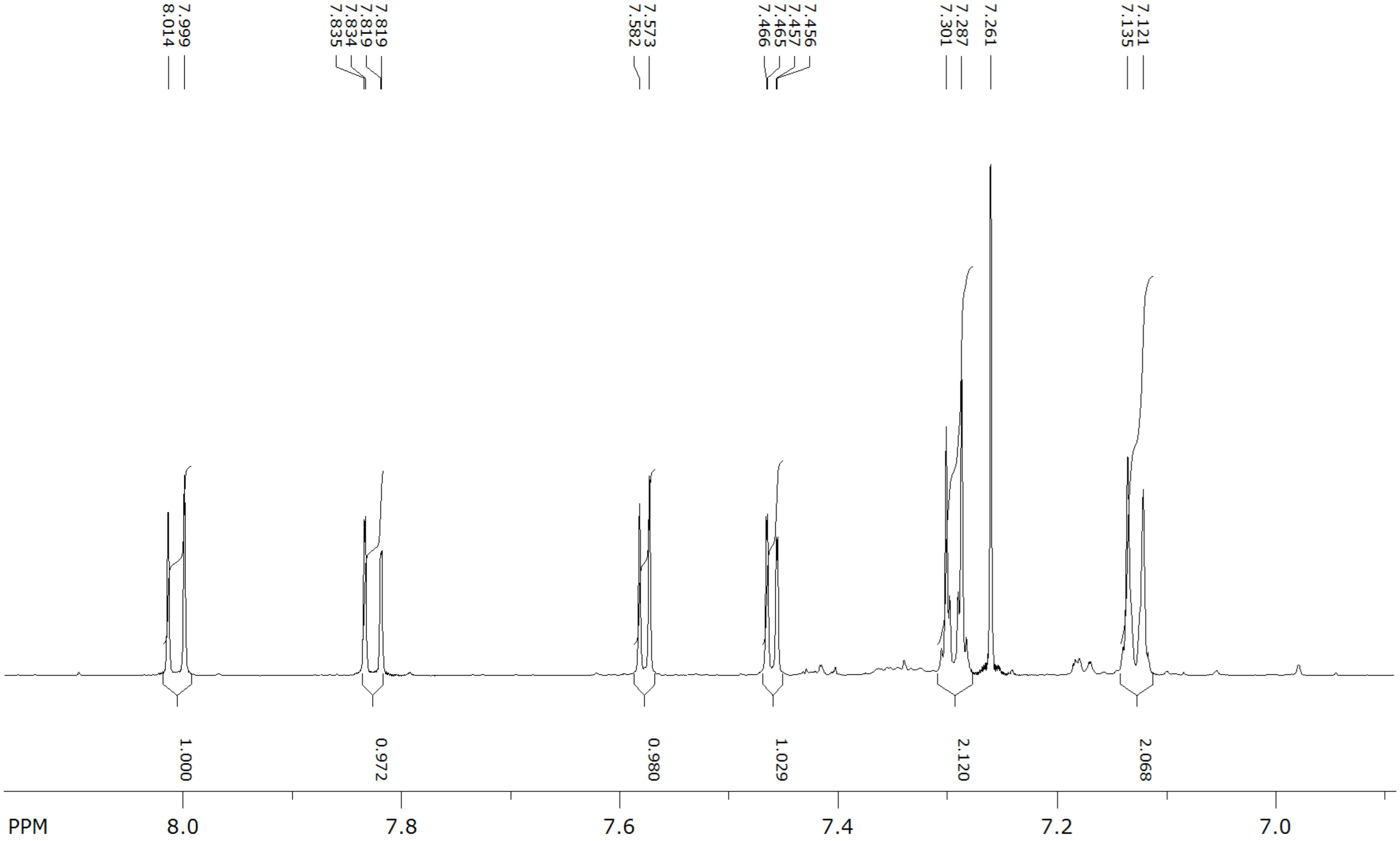

Figure S32. Aromatic part of the ^{1}H NMR ($CDCl_3$) spectrum of **15**.

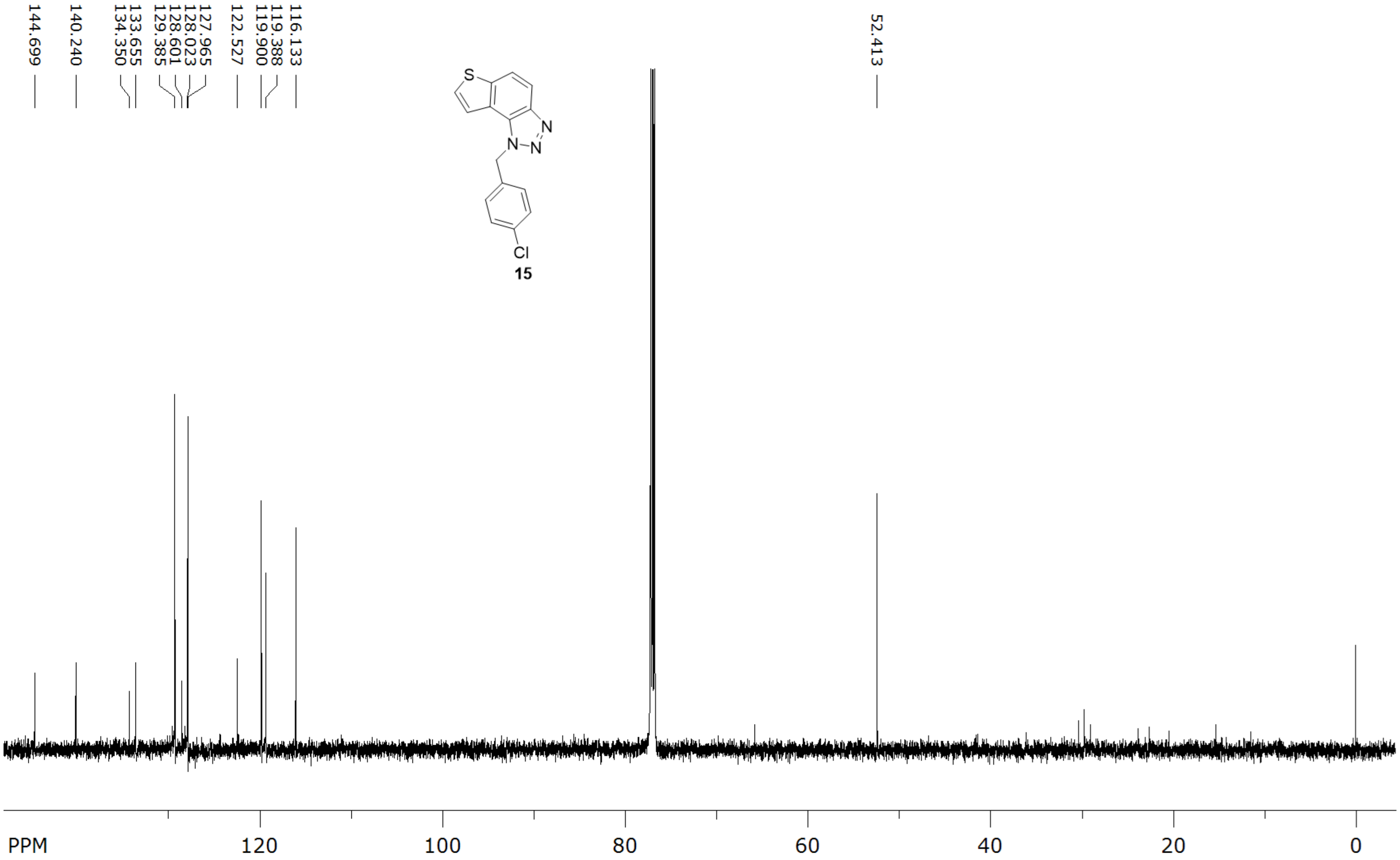

Figure S33. ^{13}C NMR ($CDCl_3$) spectrum of **15**.

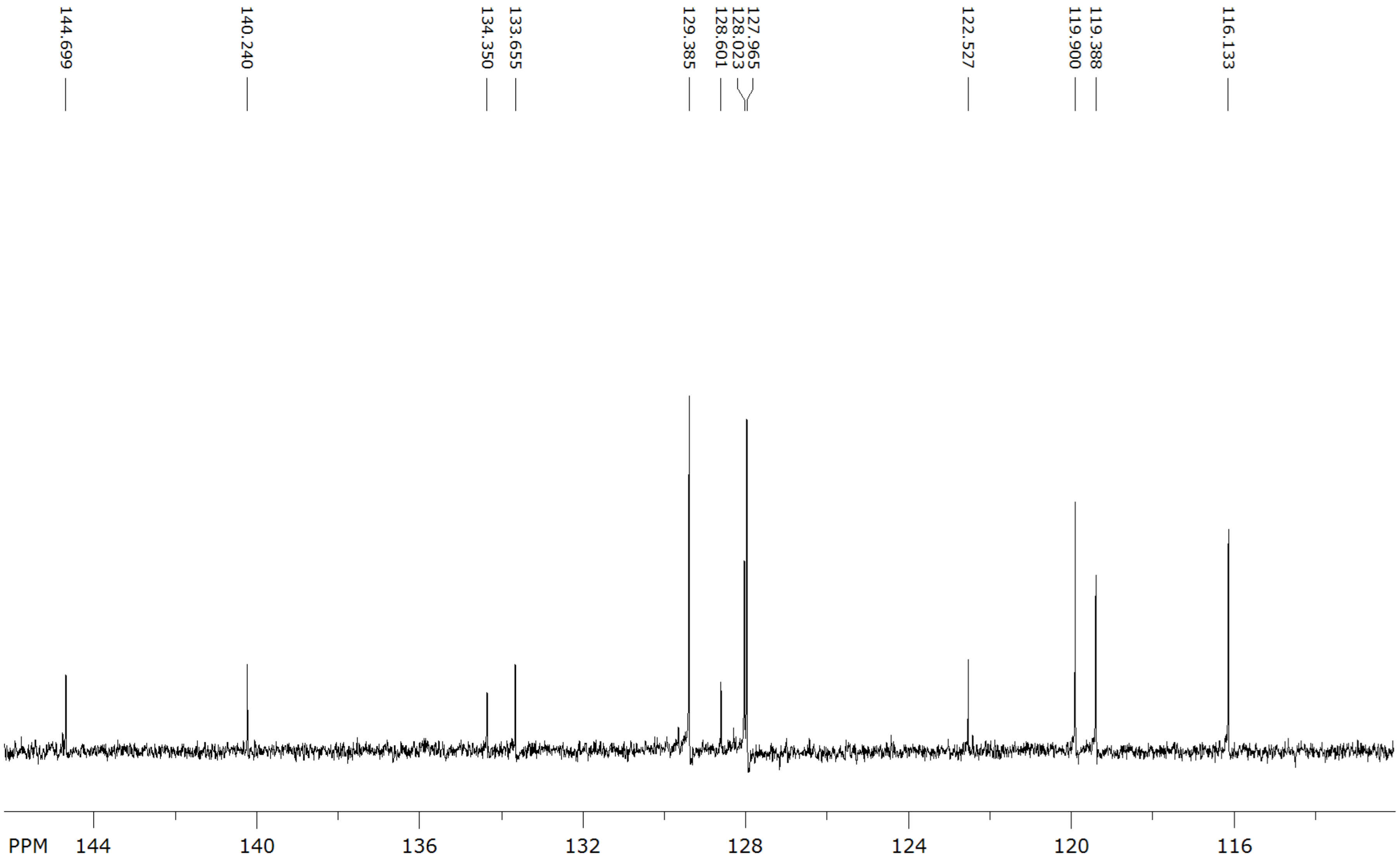

Figure S34. Aromatic part of the ^{13}C NMR ($CDCl_3$) spectrum of **15**.

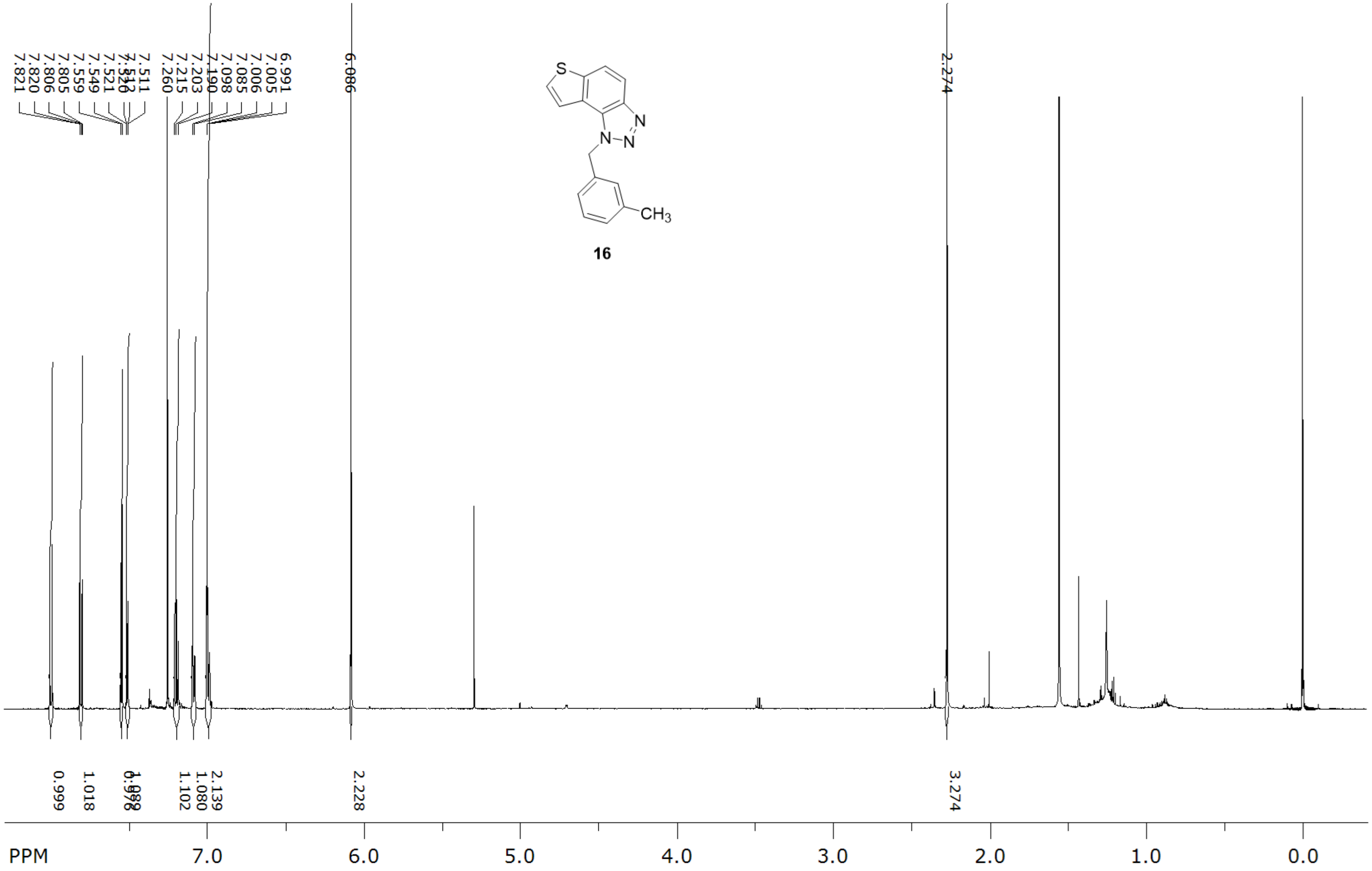

Figure S35. ^{1}H NMR ($CDCl_3$) spectrum of **16**.

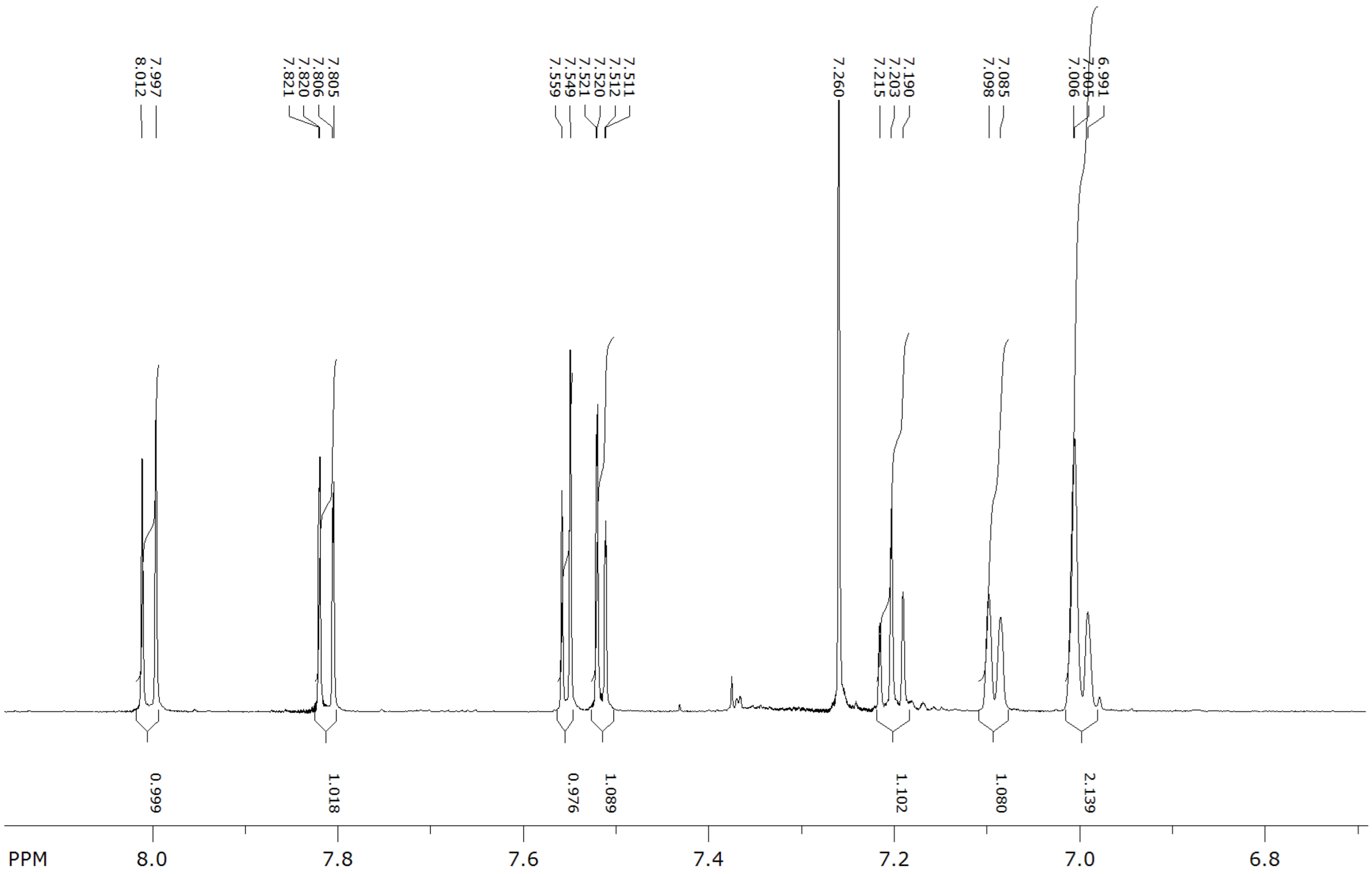

Figure S36. Aromatic part of the ^{1}H NMR ($CDCl_3$) spectrum of **16**.

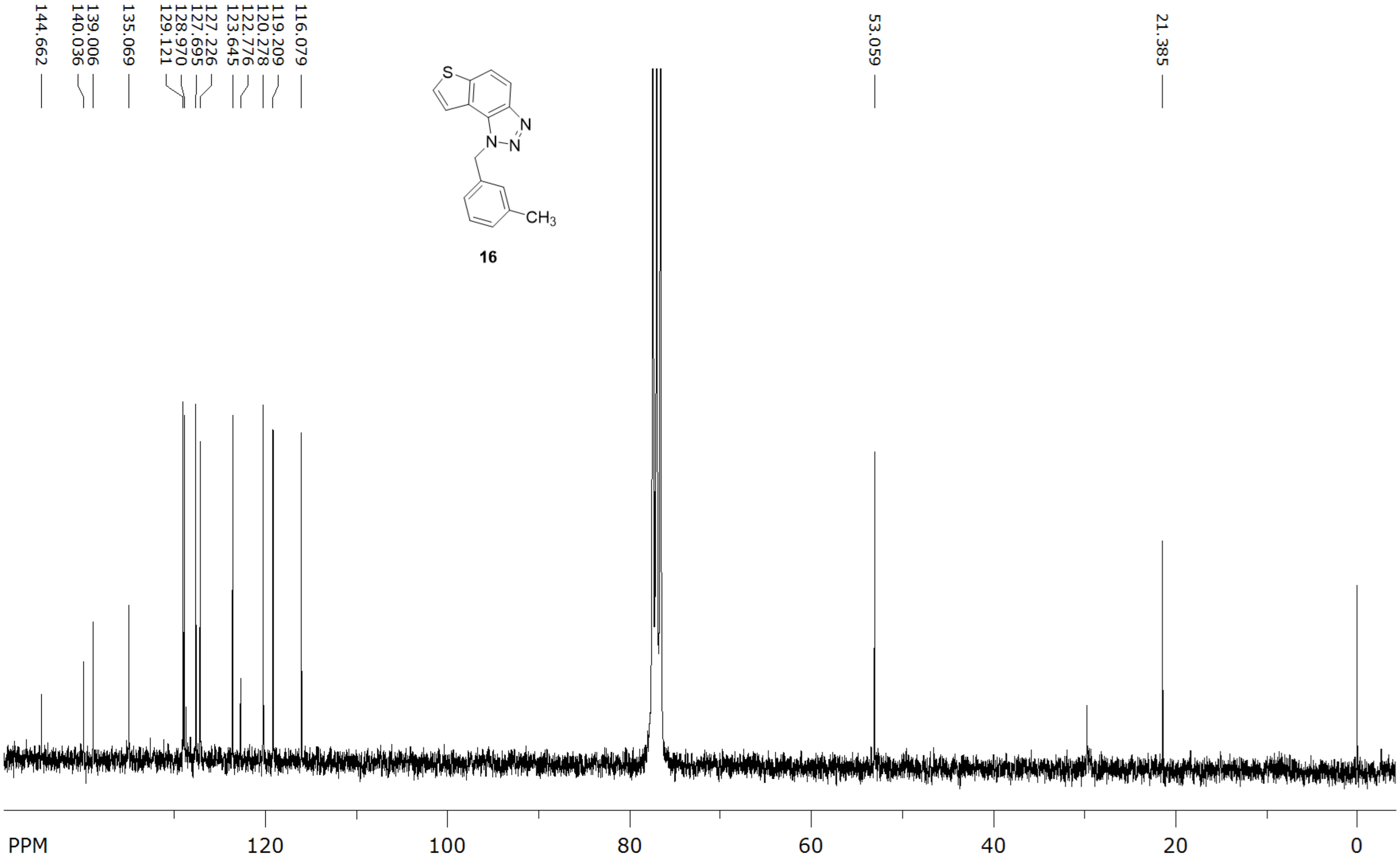

Figure S37. ^{13}C NMR ($CDCl_3$) spectrum of **16**.

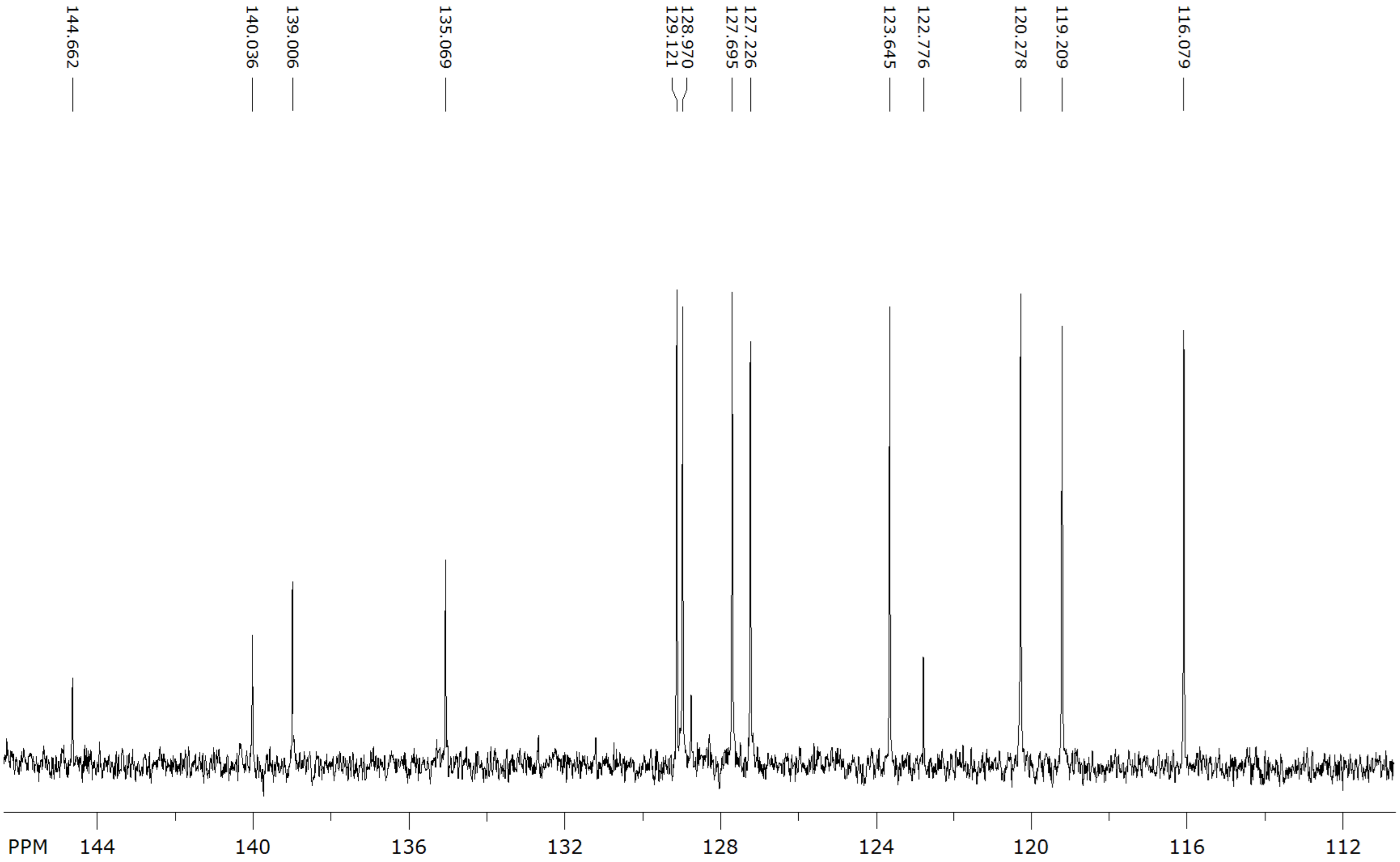

Figure S38. Aromatic part of the ^{13}C NMR ($CDCl_3$) spectrum of **16**.

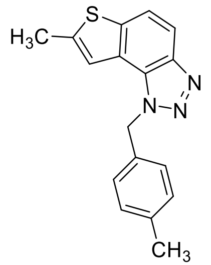

17

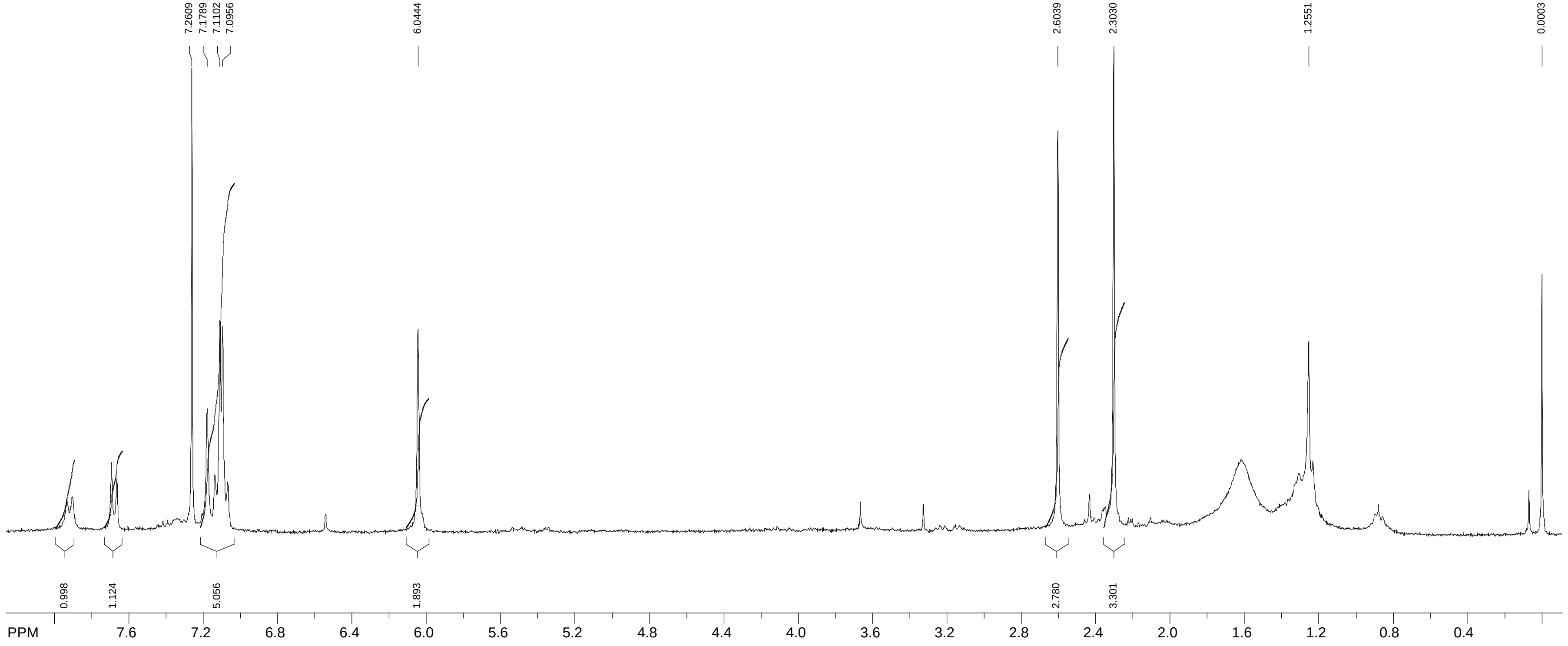

Figure S39. ^{1}H NMR ($CDCl_3$) spectrum of **17**.

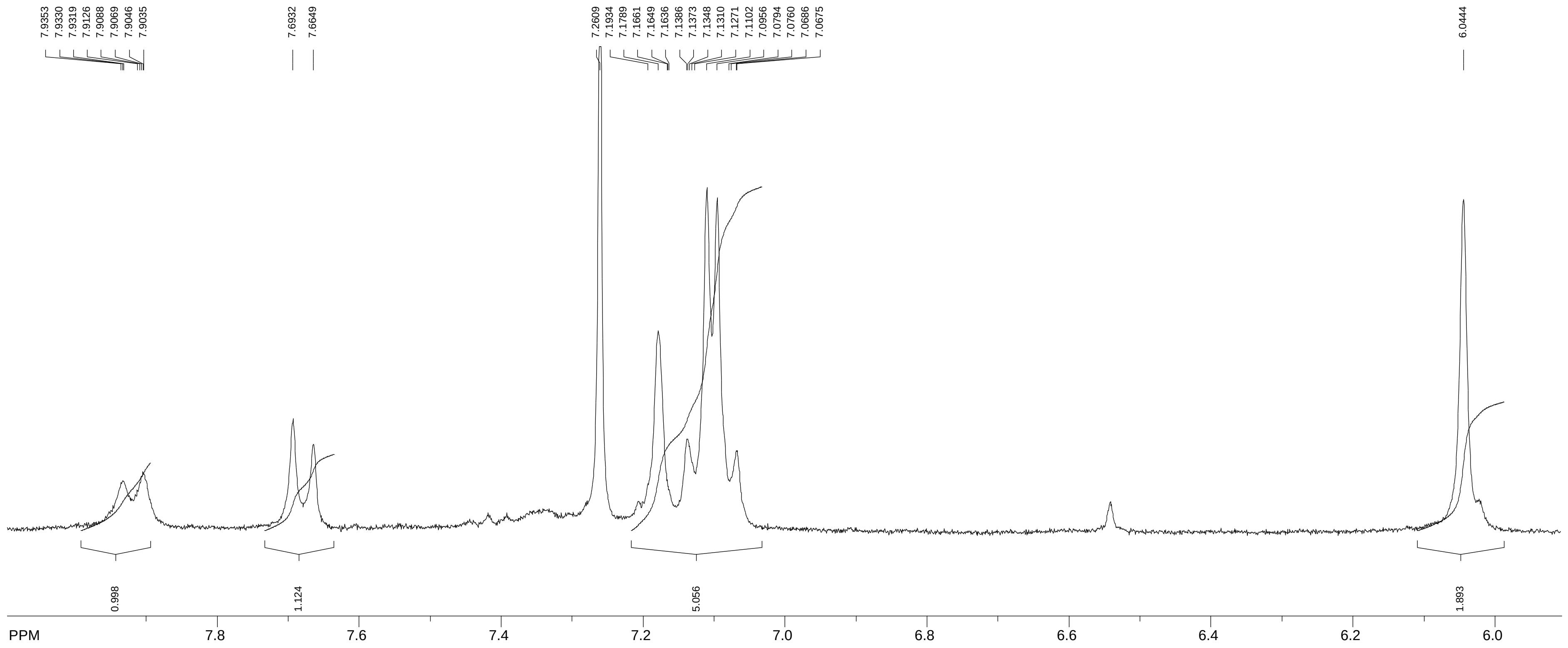

Figure S40. Aromatic part of the ^{1}H NMR ($CDCl_3$) spectrum of **17**.

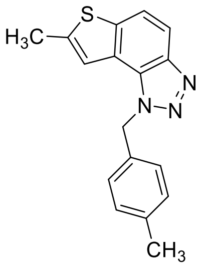

17

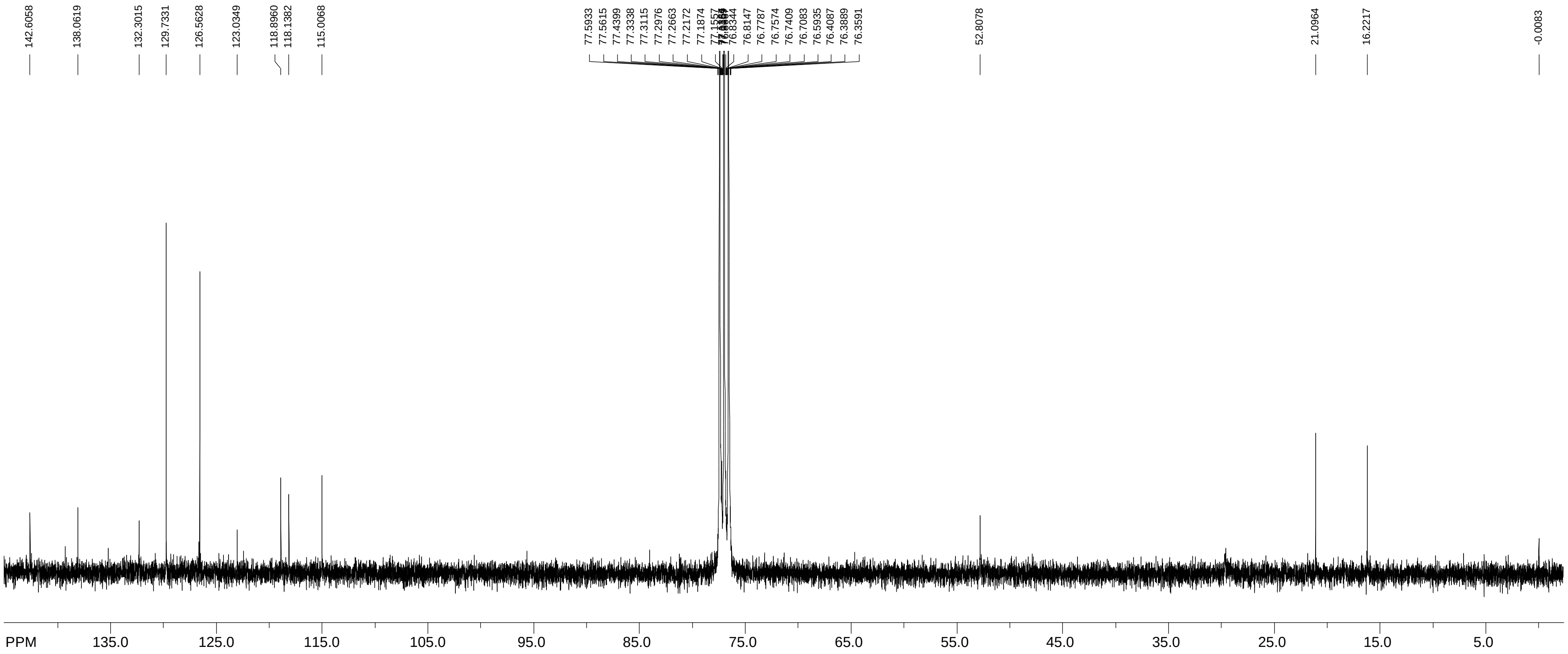

Figure S41. ^{13}C NMR ($CDCl_3$) spectrum of **17**.

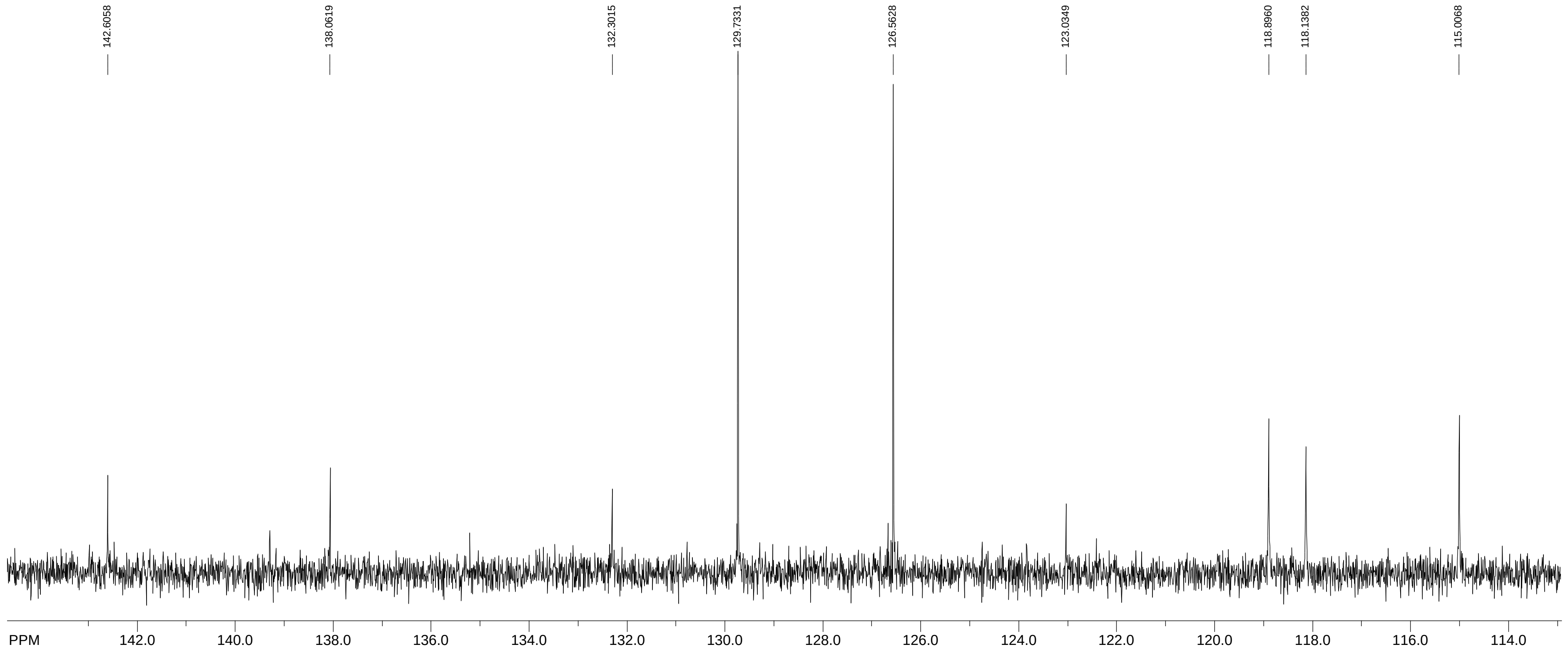

Figure S42. Aromatic part of the ^{13}C NMR ($CDCl_3$) spectrum of **17**.

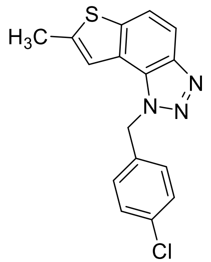

18

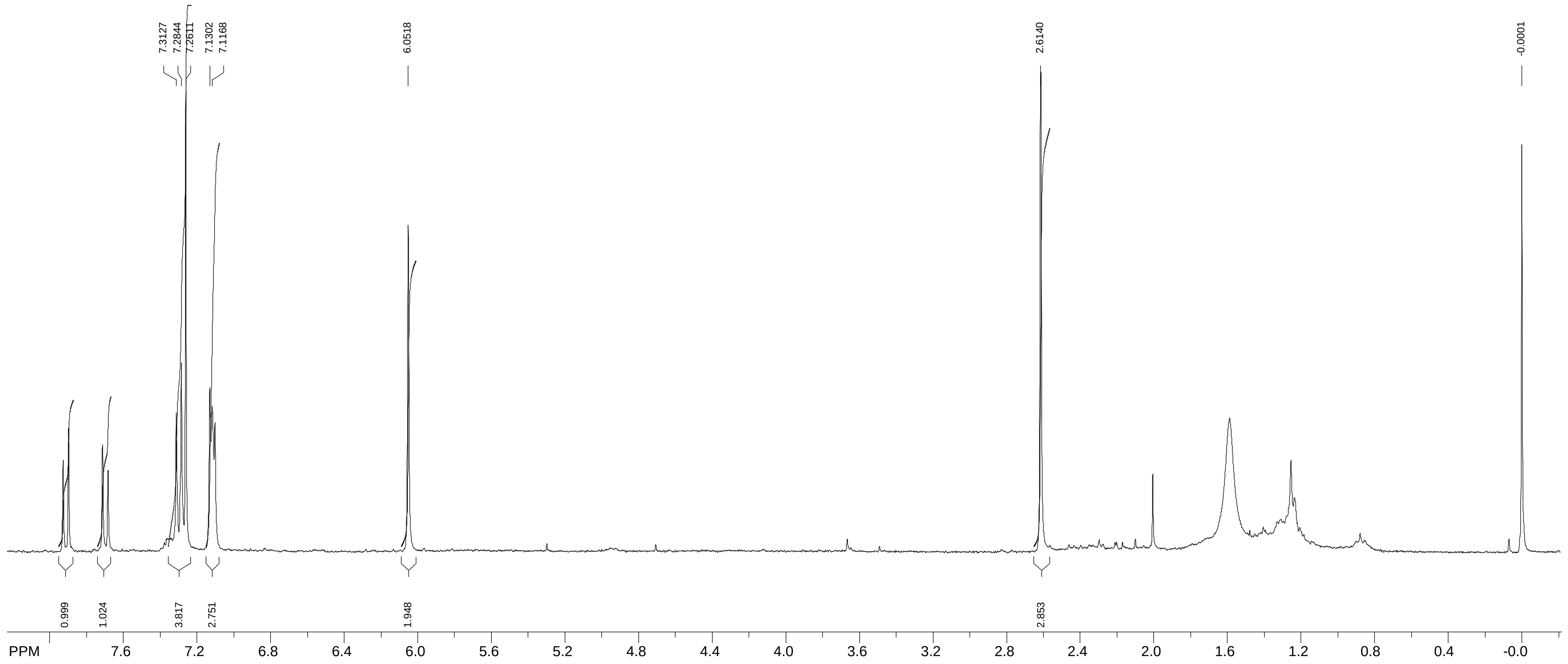

Figure S43. ^{1}H NMR ($CDCl_3$) spectrum of **18**.

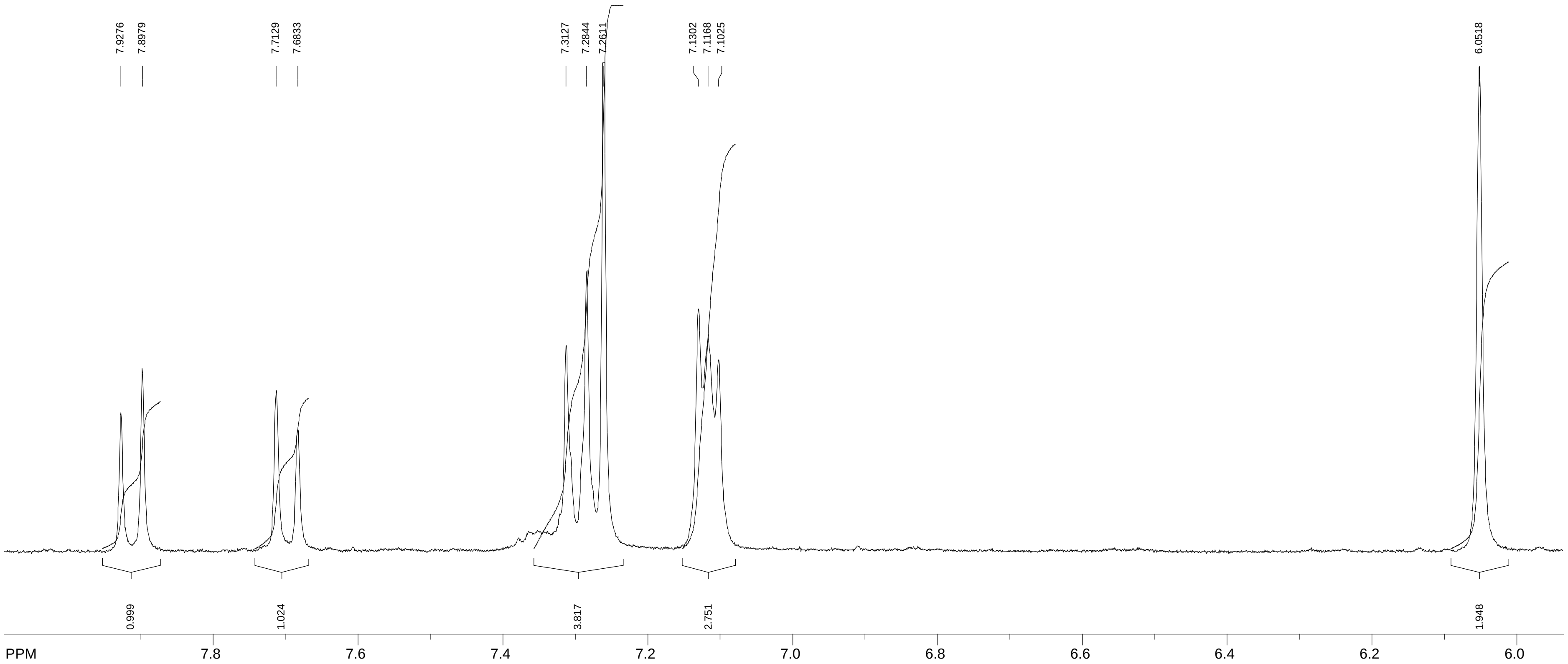

Figure S44. Aromatic part of the ^{1}H NMR ($CDCl_3$) spectrum of **18**.

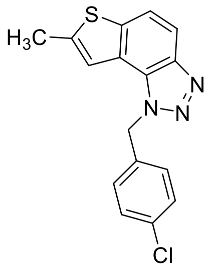

18

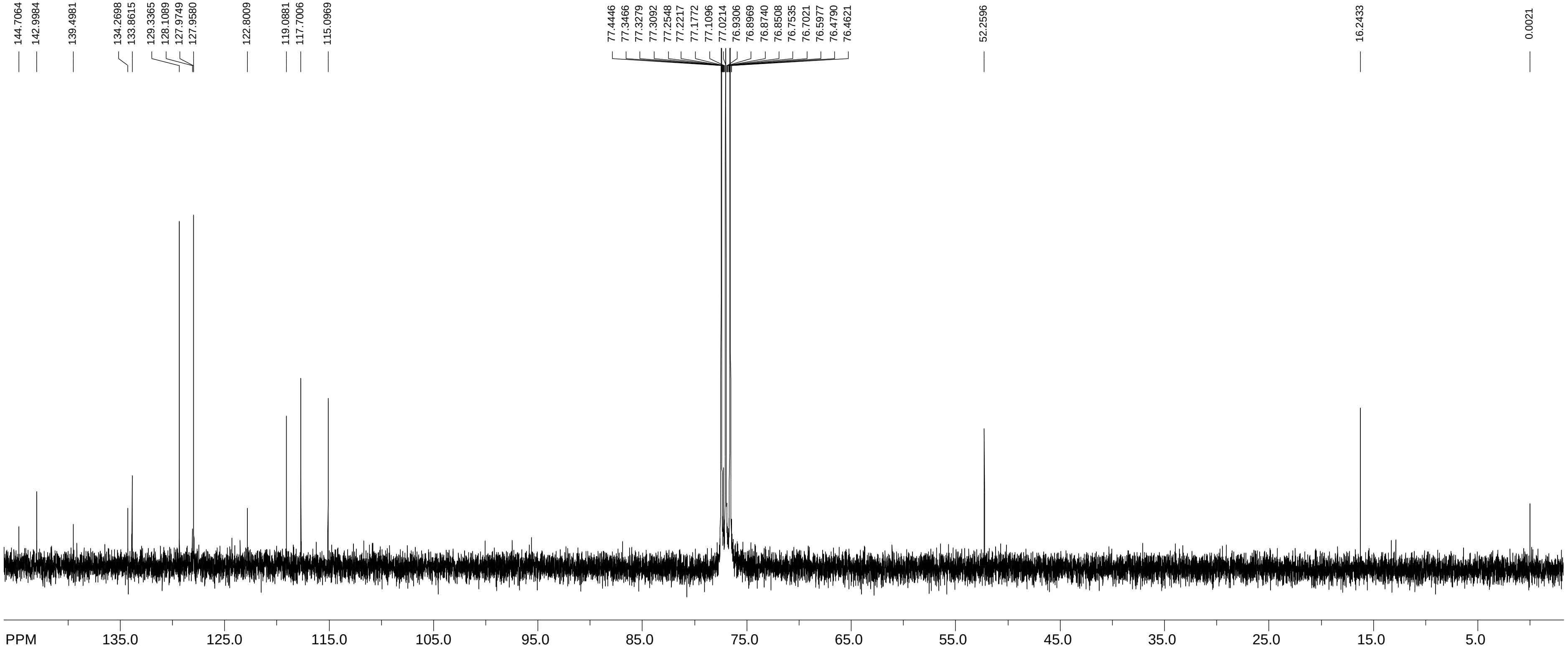

Figure S45. ^{13}C NMR ($CDCl_3$) spectrum of **18**.

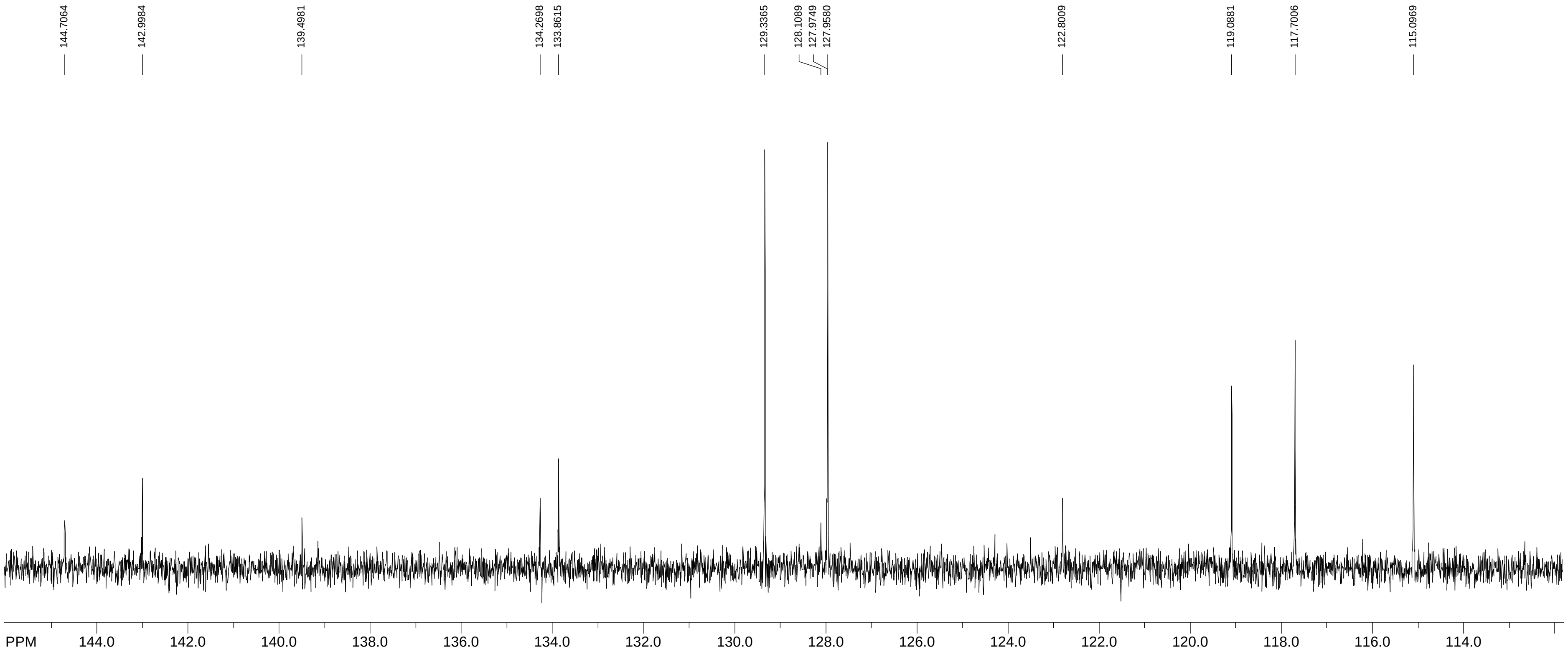

Figure S46. Aromatic part of the ^{13}C NMR ($CDCl_3$) spectrum of **18**.

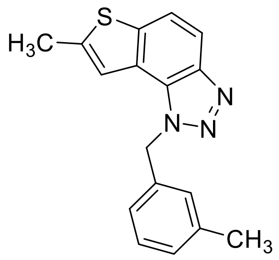

19

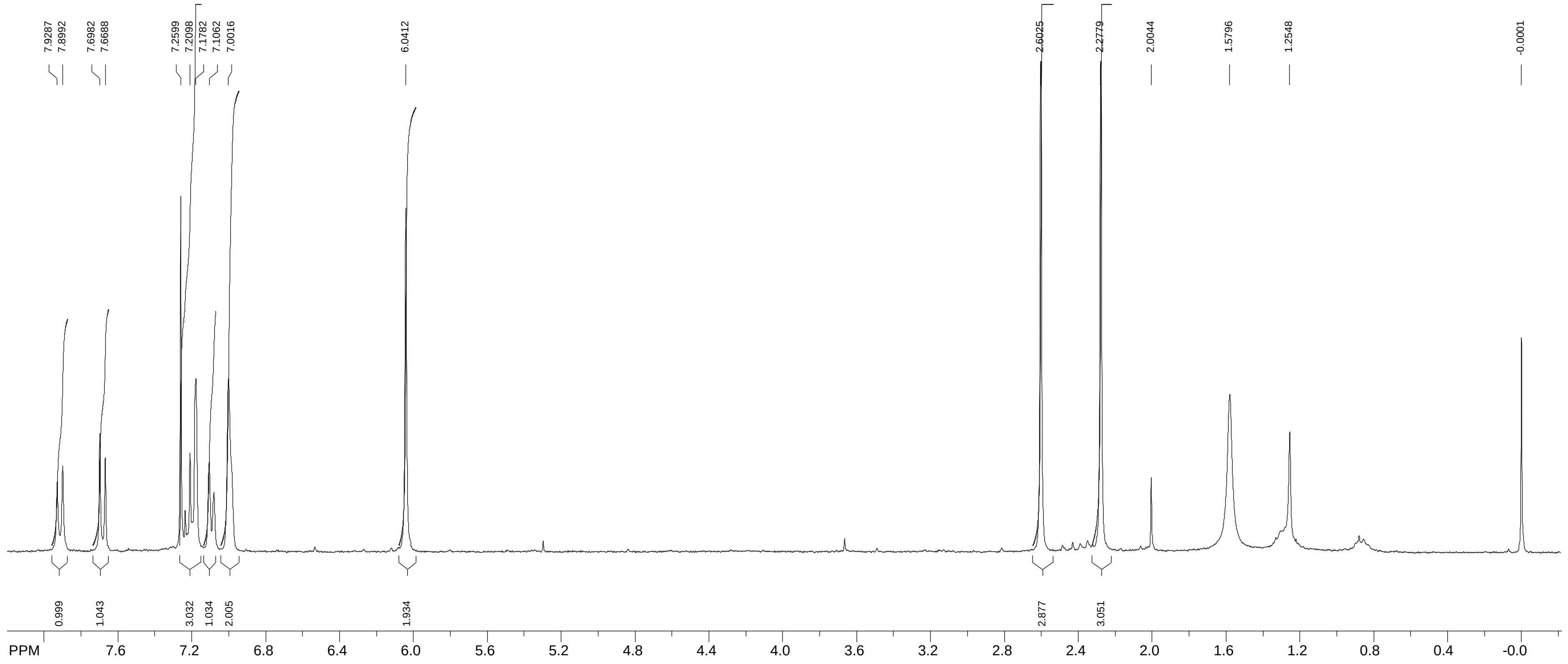

Figure S47. ^1H NMR ($CDCl_3$) spectrum of **19**.

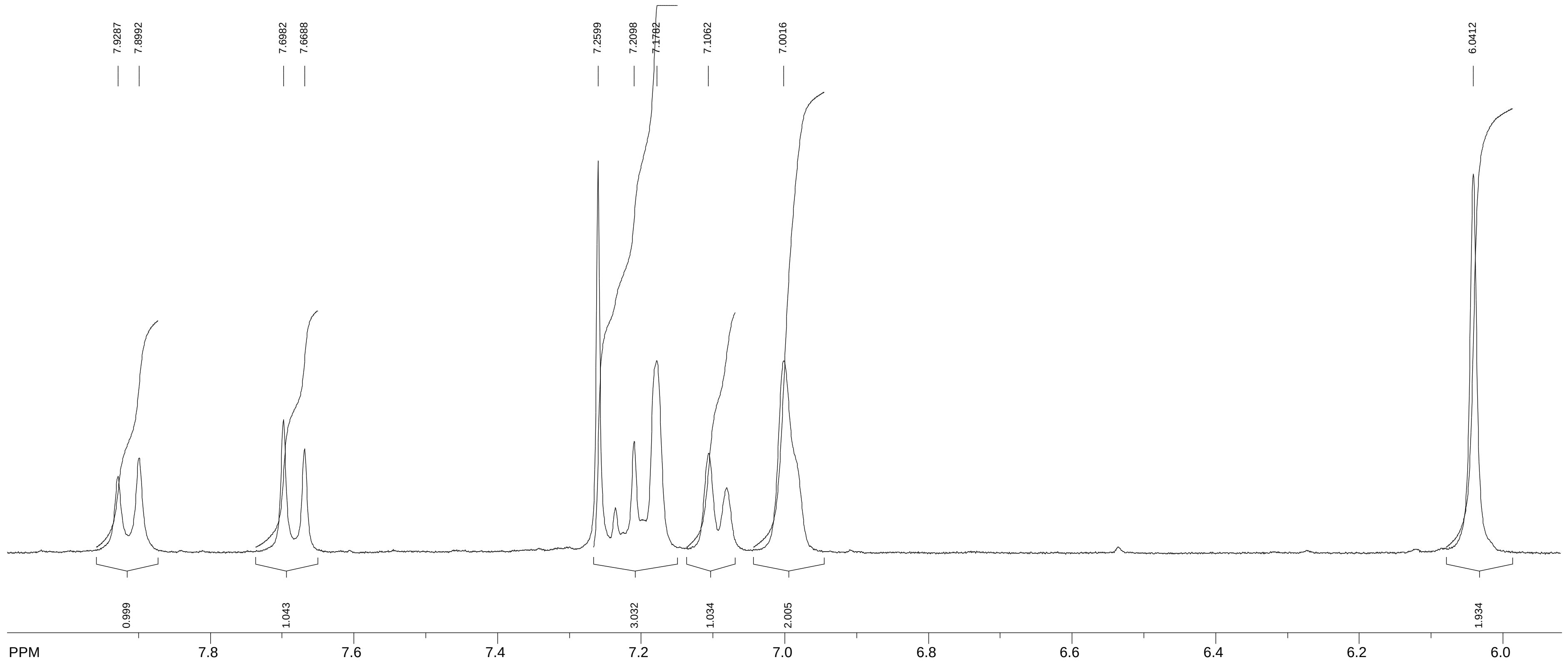

Figure S48. Aromatic part of the ^{1}H NMR ($CDCl_3$) spectrum of **19**.

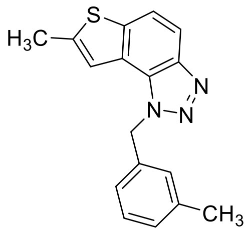

19

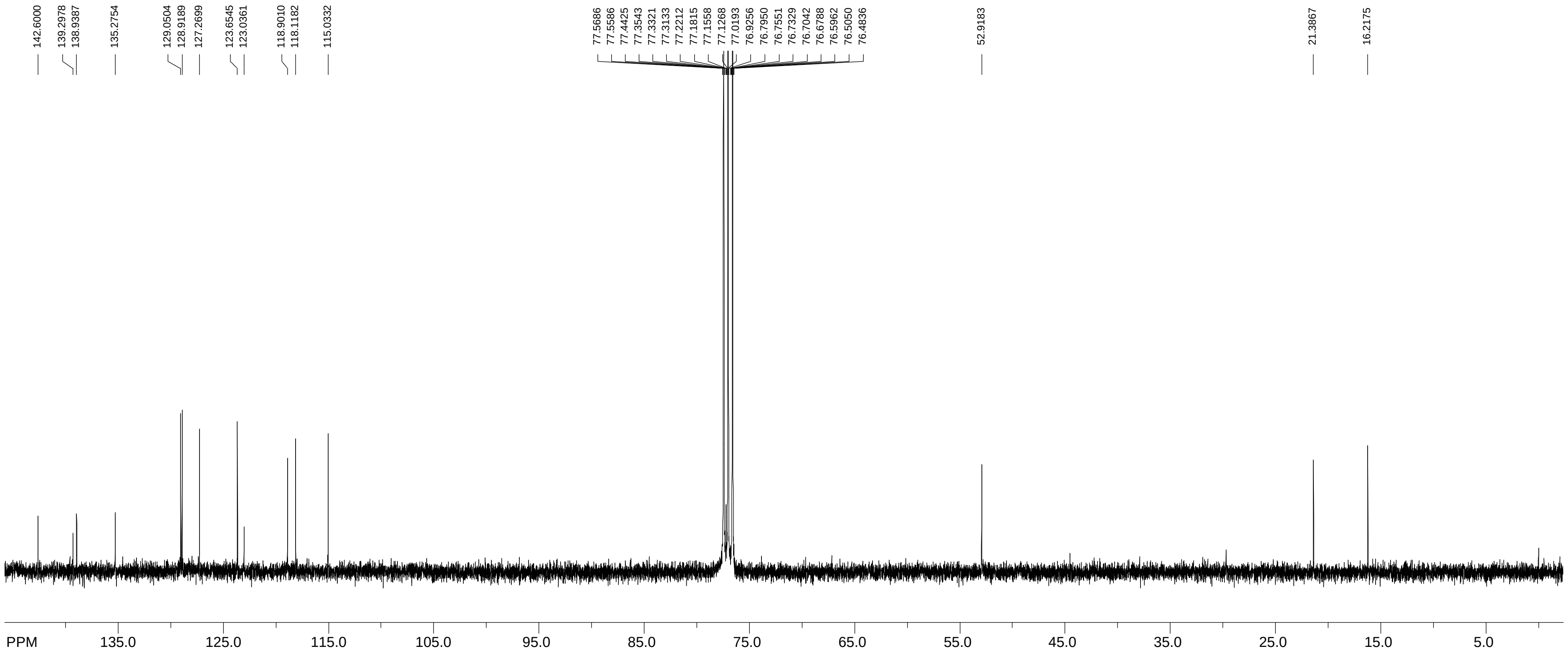

Figure S49. ^{13}C NMR ($CDCl_3$) spectrum of **19**.

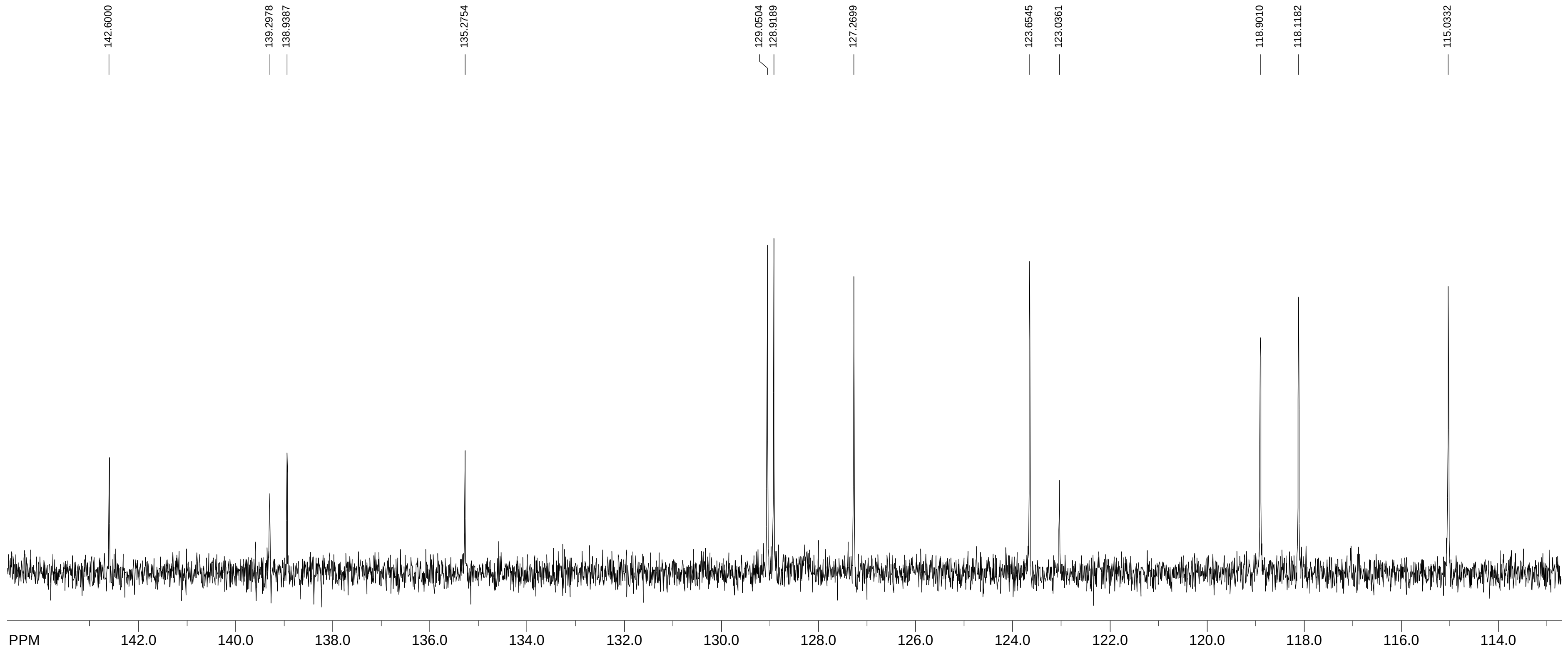

Figure S50. Aromatic part of the ^{13}C NMR ($CDCl_3$) spectrum of **19**.

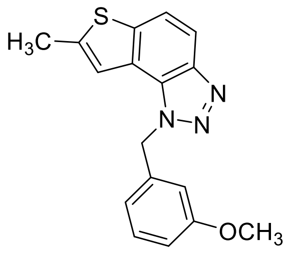

20

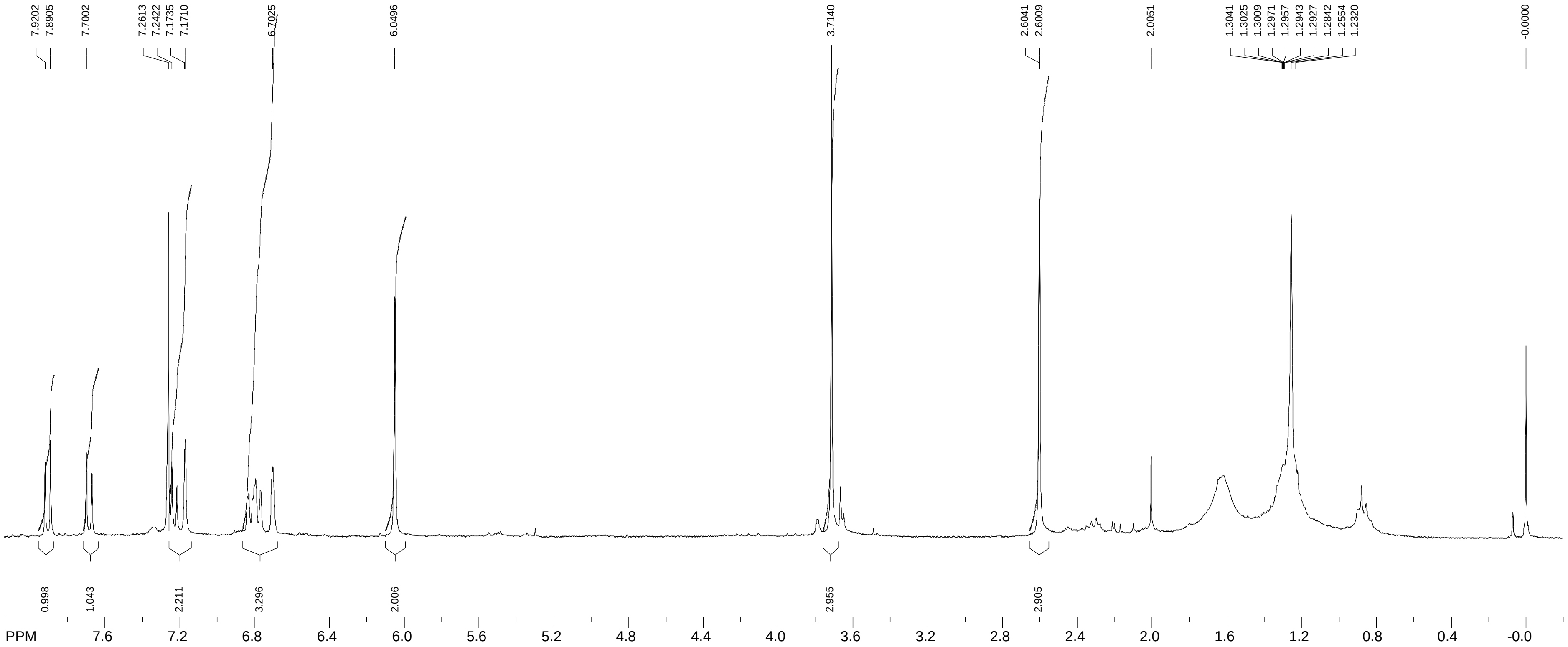

Figure S51. ^{1}H NMR (CDCl3) spectrum of **20**.

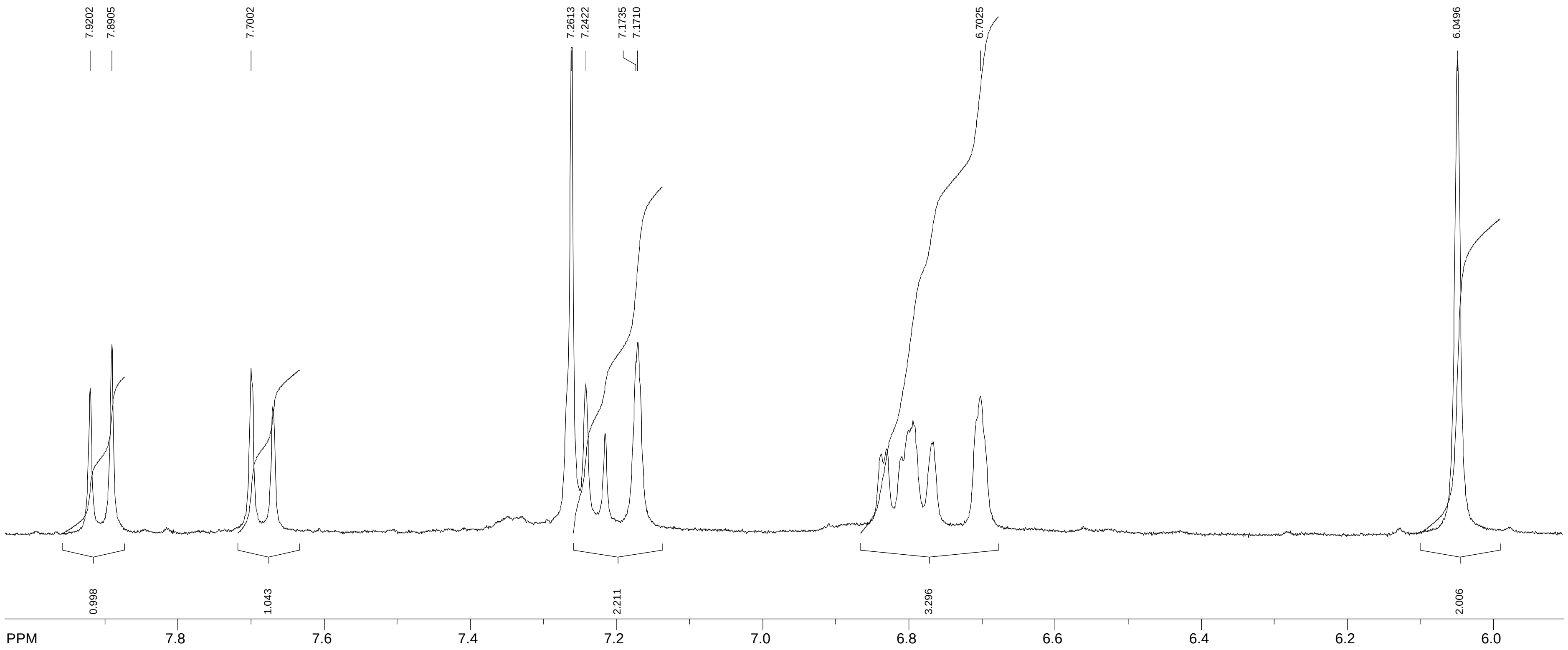

Figure S52. Aromatic part of the ^{1}H NMR ($CDCl_3$) spectrum of **20**.

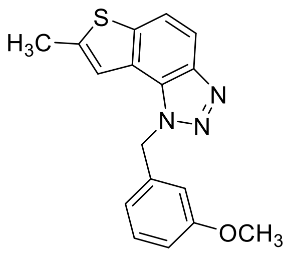
S
H3C
N
N
N
OCH3

20

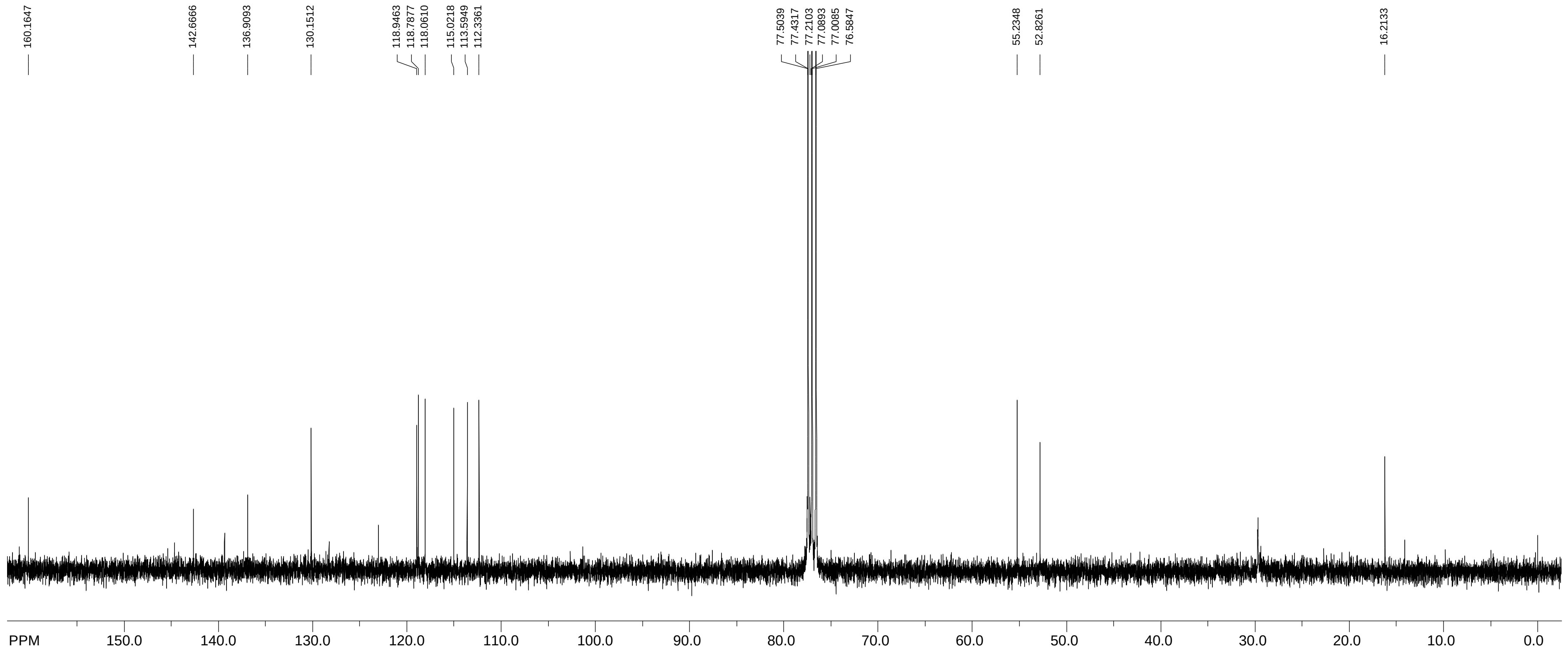
160.1647
142.6666
136.9093
130.1512
118.9463
118.7877
118.0610
115.0218
113.5949
112.3361
77.5039
77.4317
77.2103
77.0893
77.0085
76.5847
55.2348
52.8261
16.2133
PPM
150.0
140.0
130.0
120.0
110.0
100.0
90.0
80.0
70.0
60.0
50.0
40.0
30.0
20.0
10.0
0.0

Figure S53. ^{13}C NMR (CDCl$_3$) spectrum of **20**.

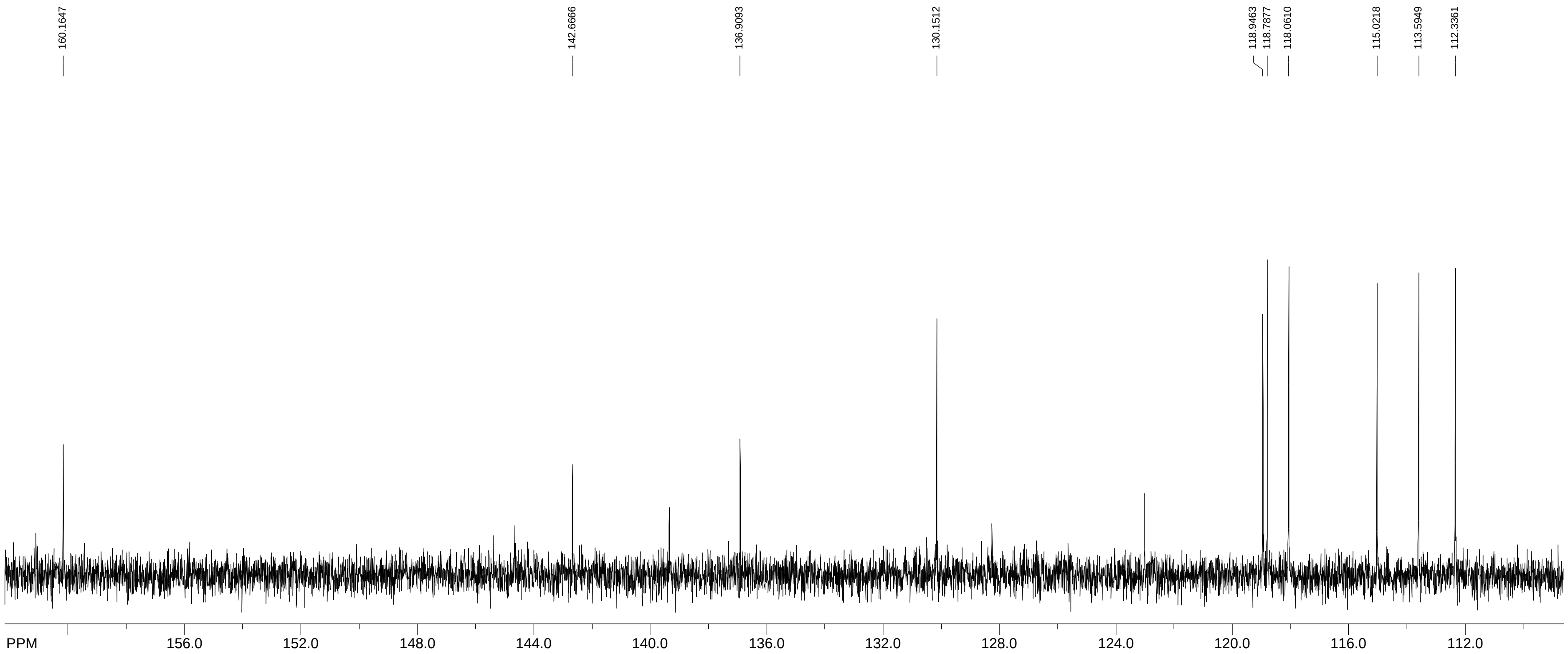

Figure S54. Aromatic part of the ^{13}C NMR (CDCl$_3$) spectrum of **20**.

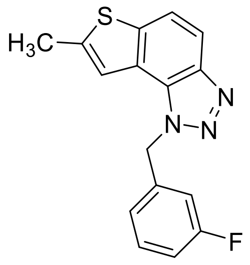
S
H3C
N
N
N
F

21

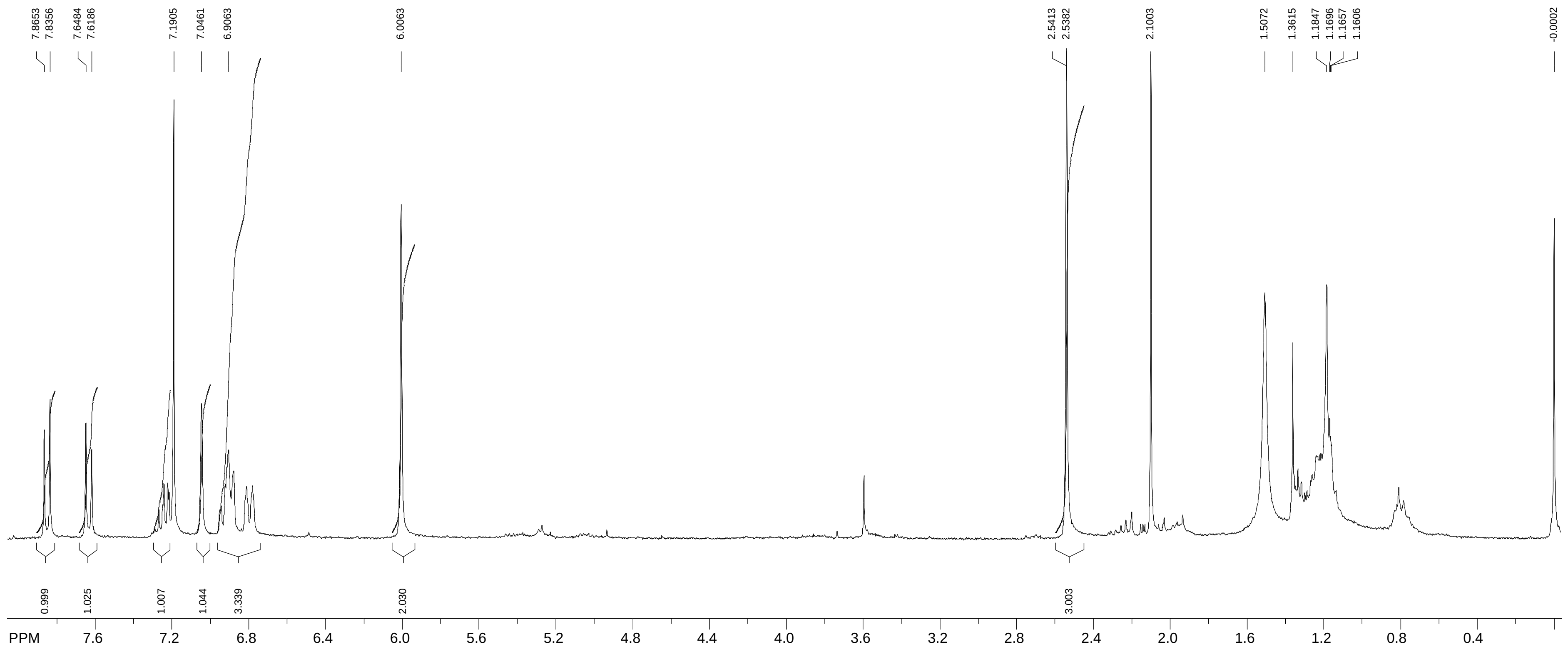
7.8653
7.8356
7.6484
7.6186
7.1905
7.0461
6.9063
6.0063
2.5413
2.5382
2.1003
1.5072
1.3615
1.1847
1.1696
1.1657
1.1606
-0.0002
0.999
1.025
1.007
1.044
3.339
2.030
3.003
PPM
7.6
7.2
6.8
6.4
6.0
5.6
5.2
4.8
4.4
4.0
3.6
3.2
2.8
2.4
2.0
1.6
1.2
0.8
0.4

Figure S55. ^{1}H NMR ($CDCl_3$) spectrum of **21**.

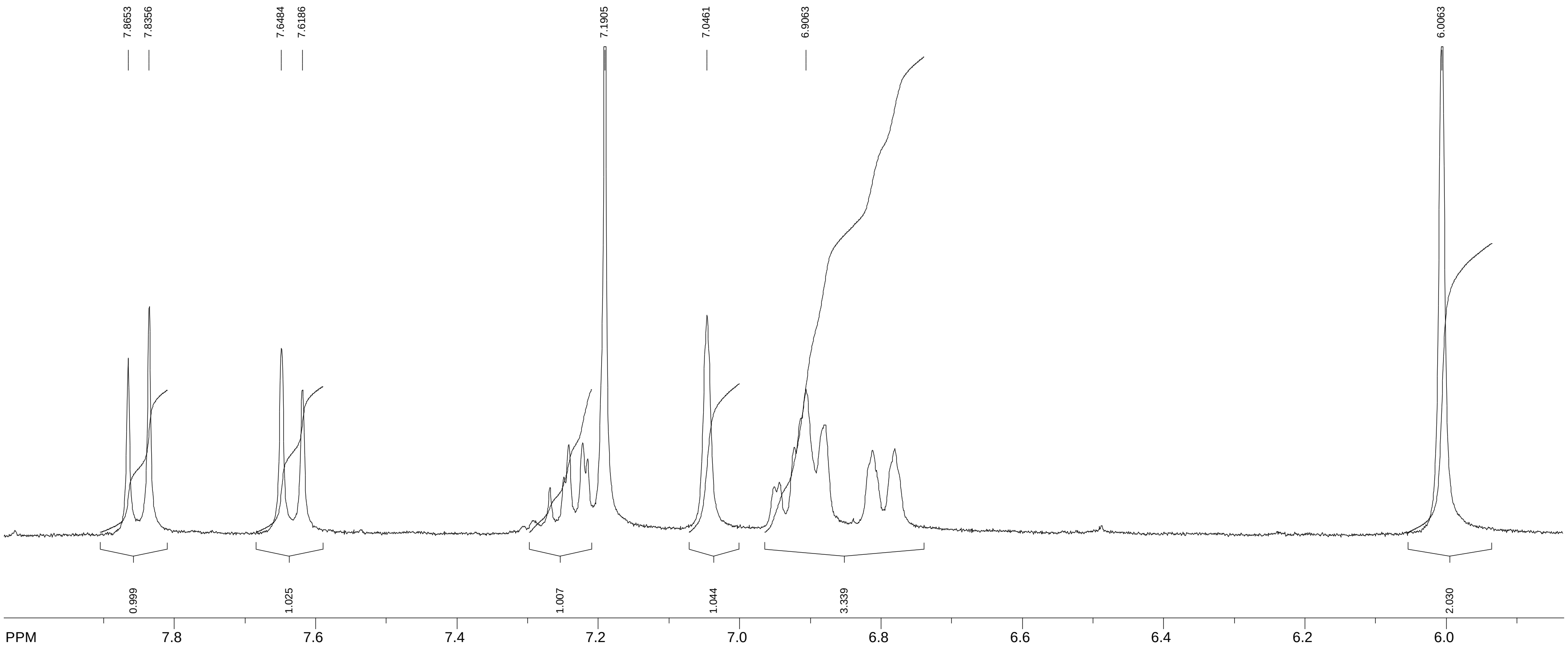

Figure S56. Aromatic part of the ^{1}H NMR ($CDCl_3$) spectrum of **21**.

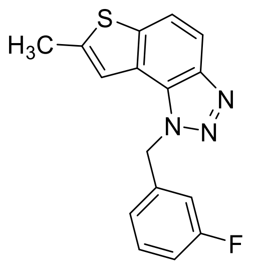

S
H3C
N
N
N
F

21

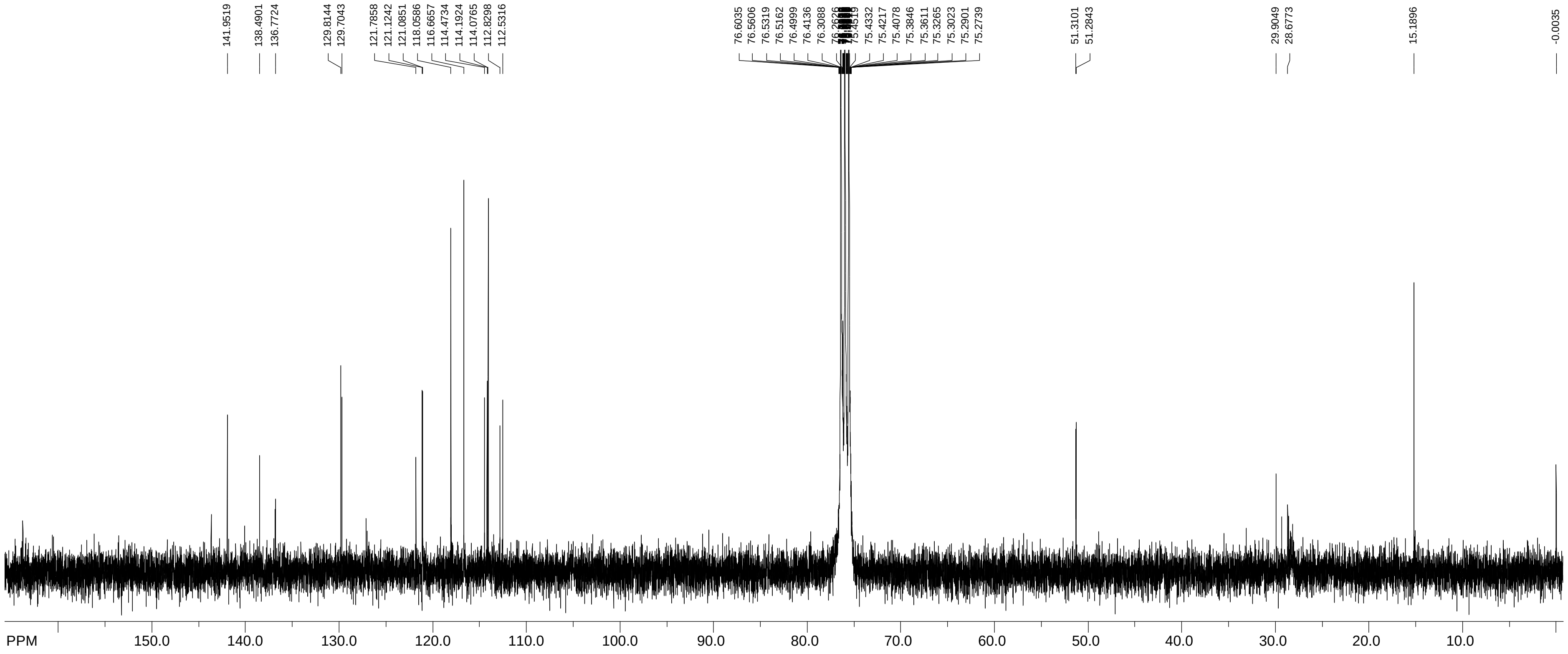

141.9519
138.4901
136.7724
129.8144
129.7043
121.7858
121.1242
121.0851
118.0586
116.6657
114.4734
114.1924
114.0765
112.8298
112.5316
76.6035
76.5606
76.5319
76.5162
76.4999
76.4136
76.3088
75.4519
75.4332
75.4217
75.4078
75.3846
75.3611
75.3265
75.3023
75.2901
75.2739
51.3101
51.2843
29.9049
28.6773
15.1896
-0.0035
PPM
150.0
140.0
130.0
120.0
110.0
100.0
90.0
80.0
70.0
60.0
50.0
40.0
30.0
20.0
10.0

Figure S57. ^{13}C NMR ($CDCl_3$) spectrum of **21**.

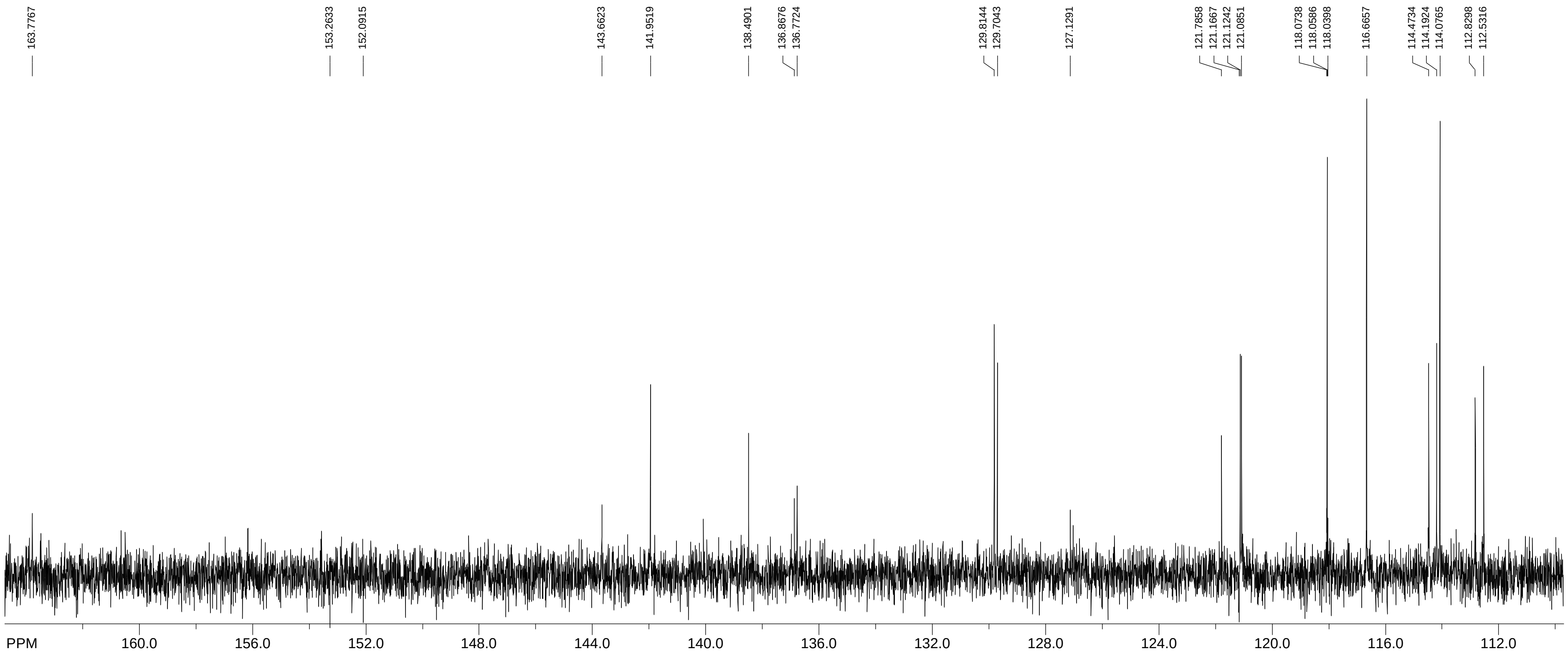

Figure S58. Aromatic part of the ^{13}C NMR ($CDCl_3$) spectrum of **21**.

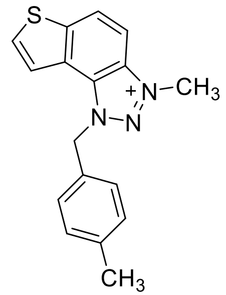
S
N
N
N
CH3
CH3

22

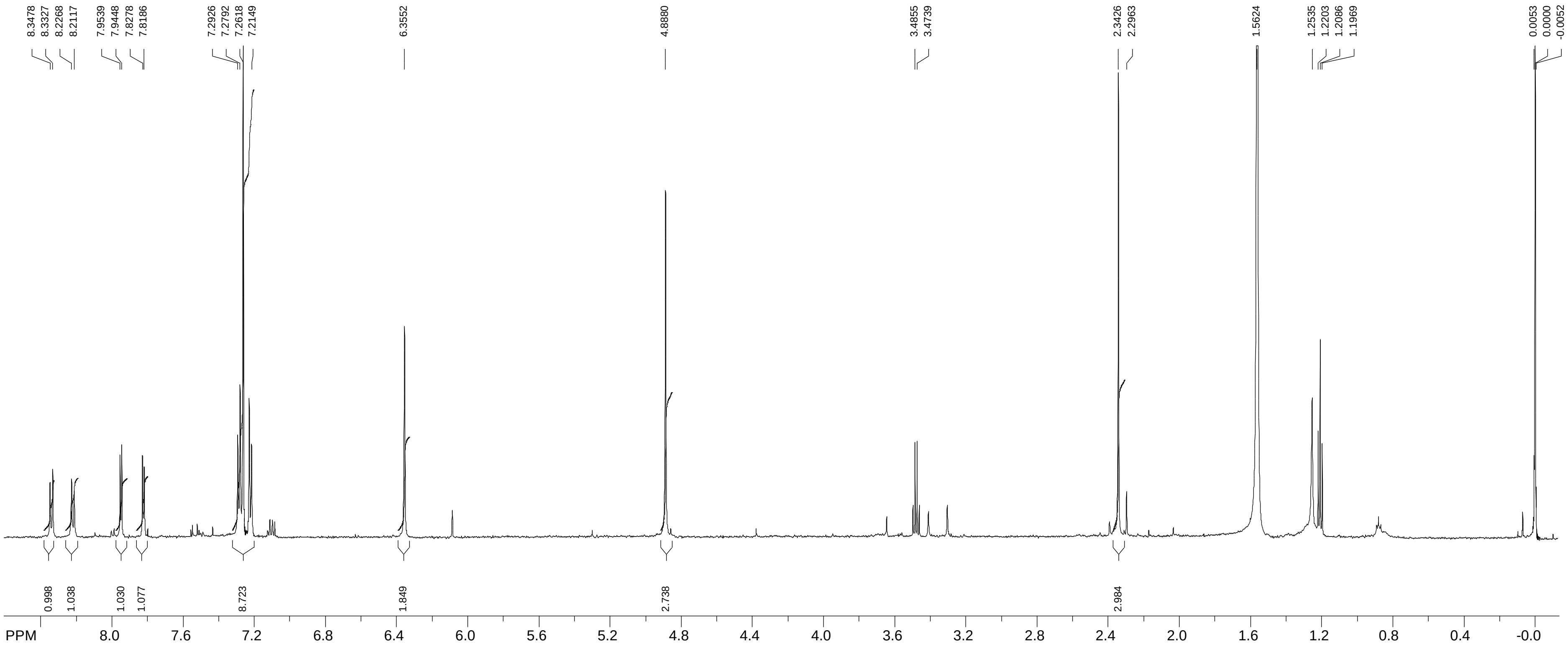
8.3478
8.3327
8.2268
8.2117
7.9539
7.9448
7.8278
7.8186
7.2926
7.2792
7.2618
7.2149
6.3552
4.8880
3.4855
3.4739
2.3426
2.2963
1.5624
1.2535
1.2203
1.2086
1.1969
0.0053
0.0000
-0.0052
0.998
1.038
1.030
1.077
8.723
1.849
2.738
2.984
PPM
8.0
7.6
7.2
6.8
6.4
6.0
5.6
5.2
4.8
4.4
4.0
3.6
3.2
2.8
2.4
2.0
1.6
1.2
0.8
0.4
-0.0

Figure S59. ¹H NMR ($CDCl_3$) spectrum of **22**.

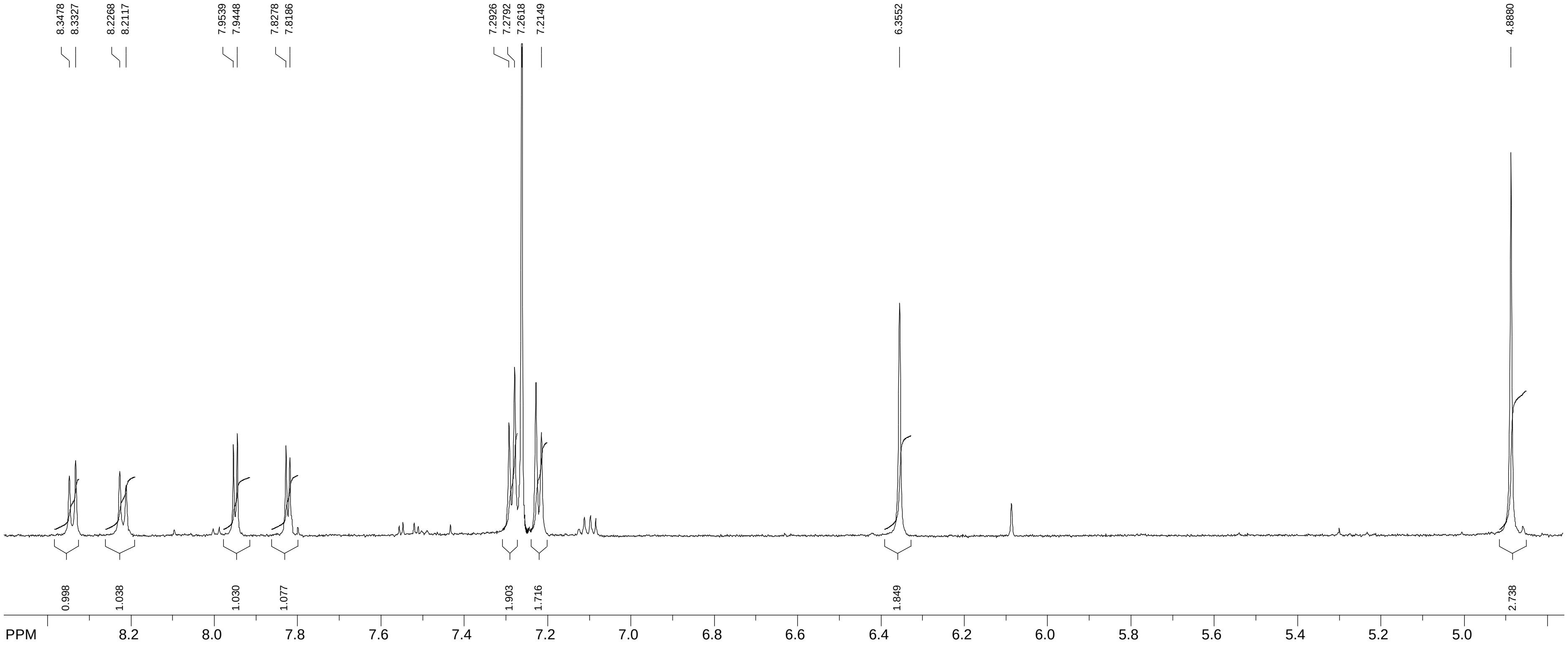

Figure S60. Aromatic part of the ¹H NMR ($CDCl_3$) spectrum of **22**.

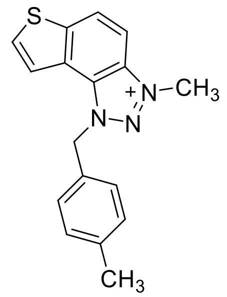

22

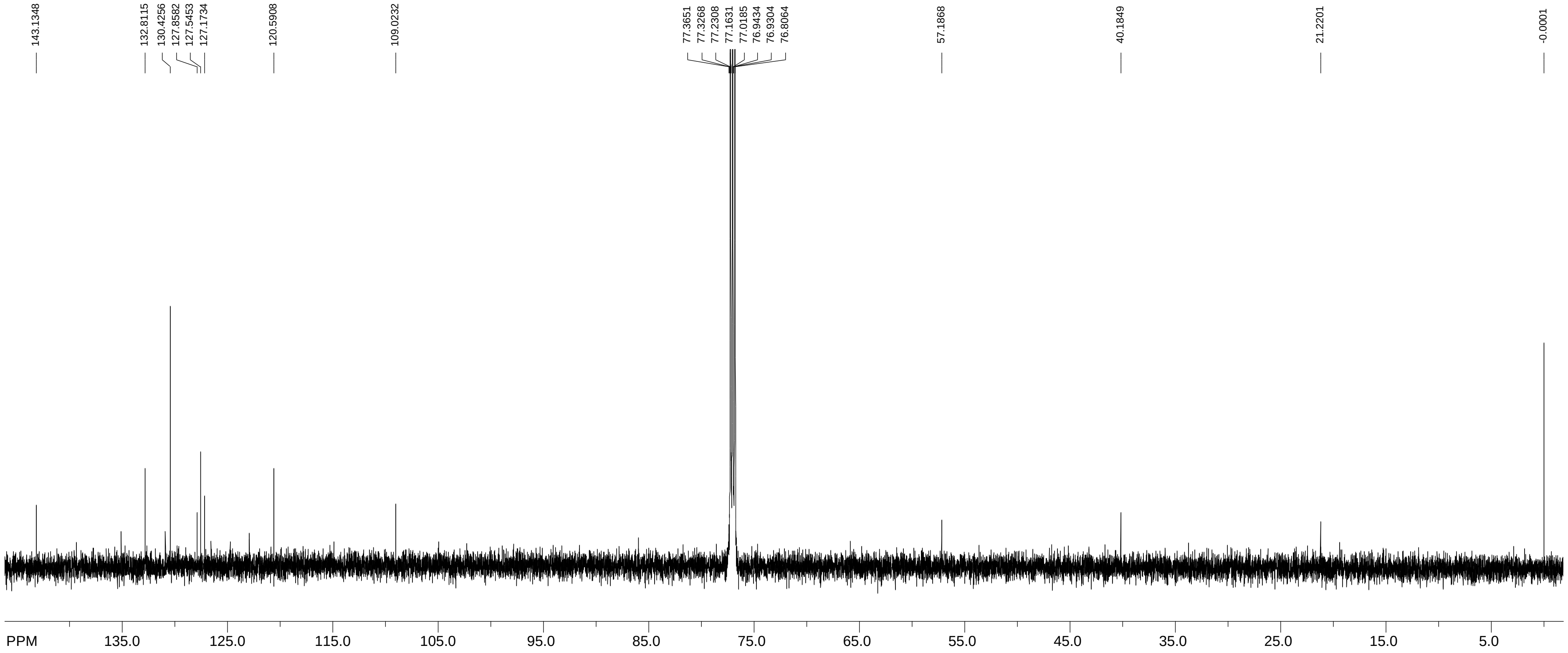

Figure S61. ^{13}C NMR ($CDCl_3$) spectrum of **22**.

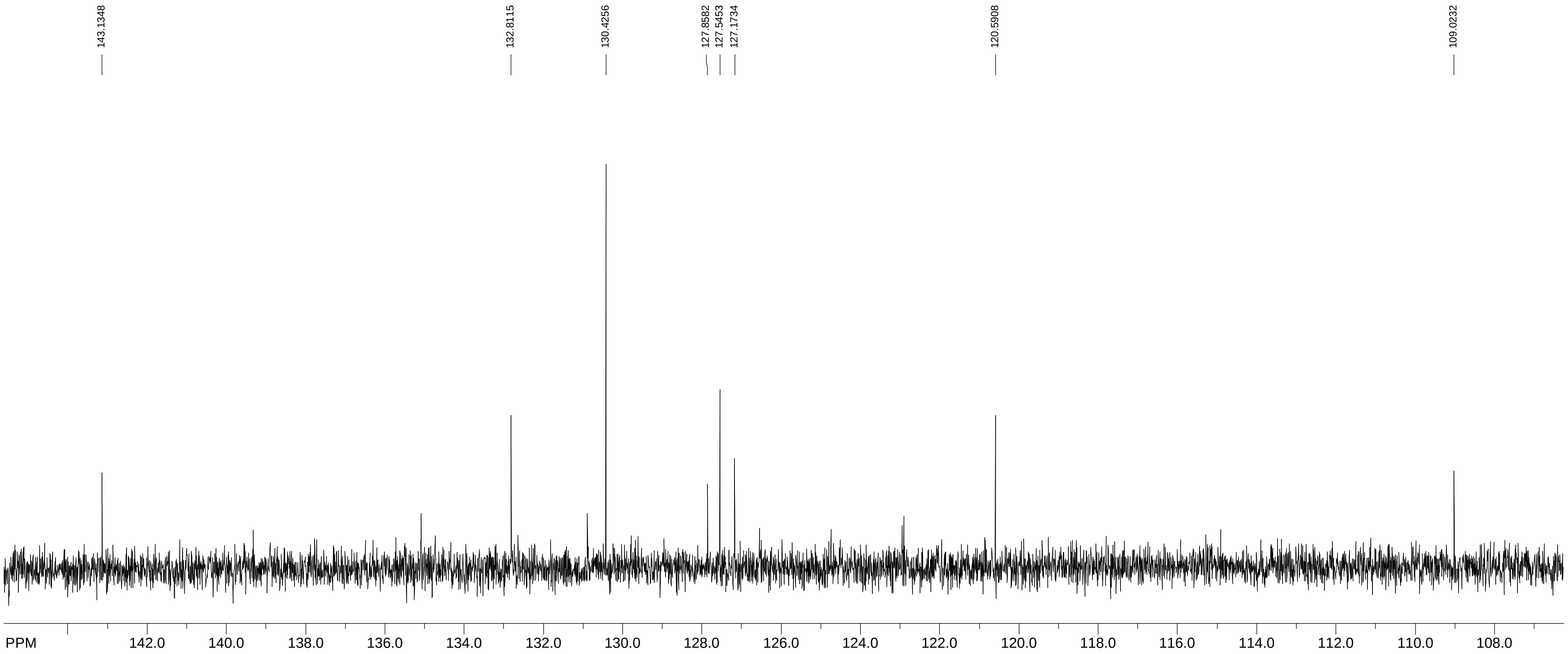

Figure S62. Aromatic part of the ^{13}C NMR ($CDCl_3$) spectrum of **22**.

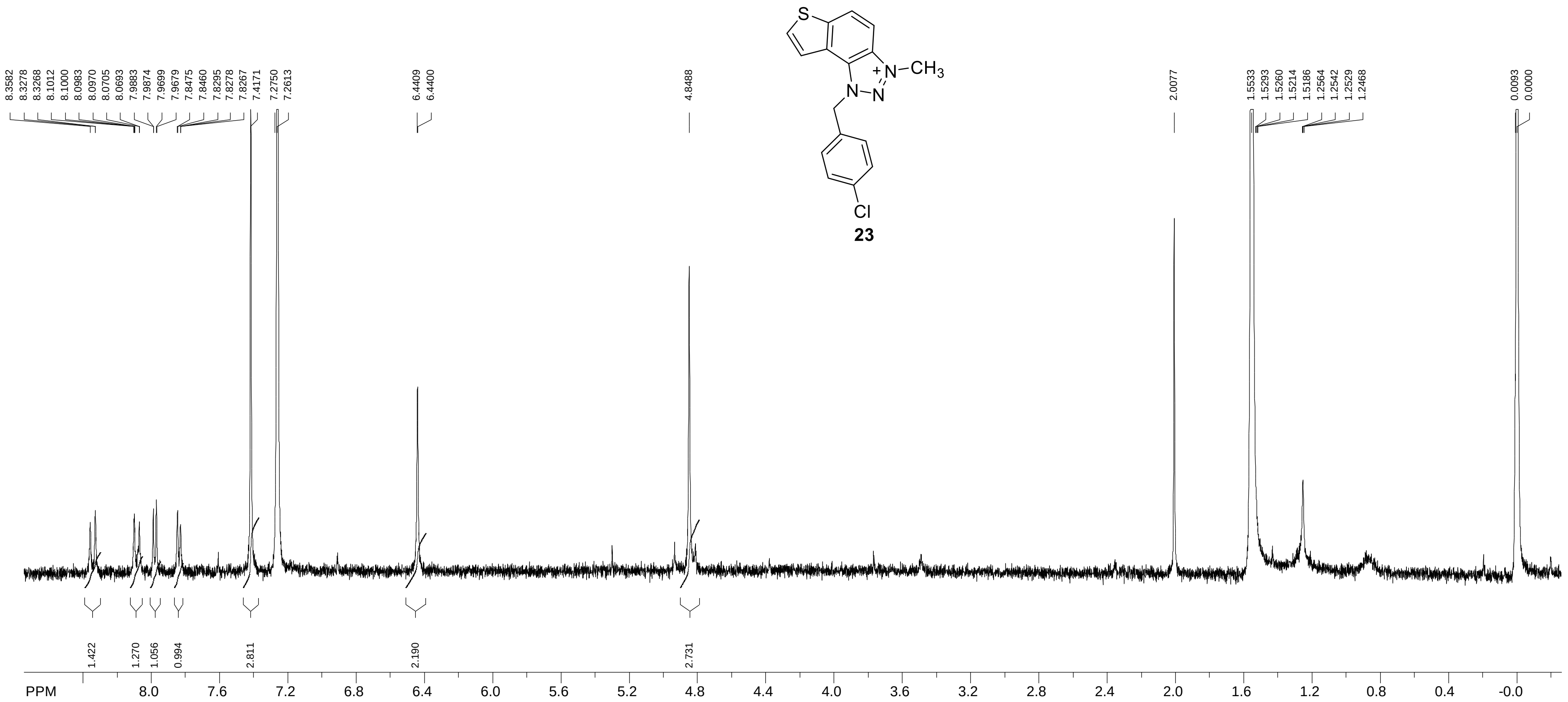

Figure S63. ^{1}H NMR ($CDCl_3$) spectrum of **23**.

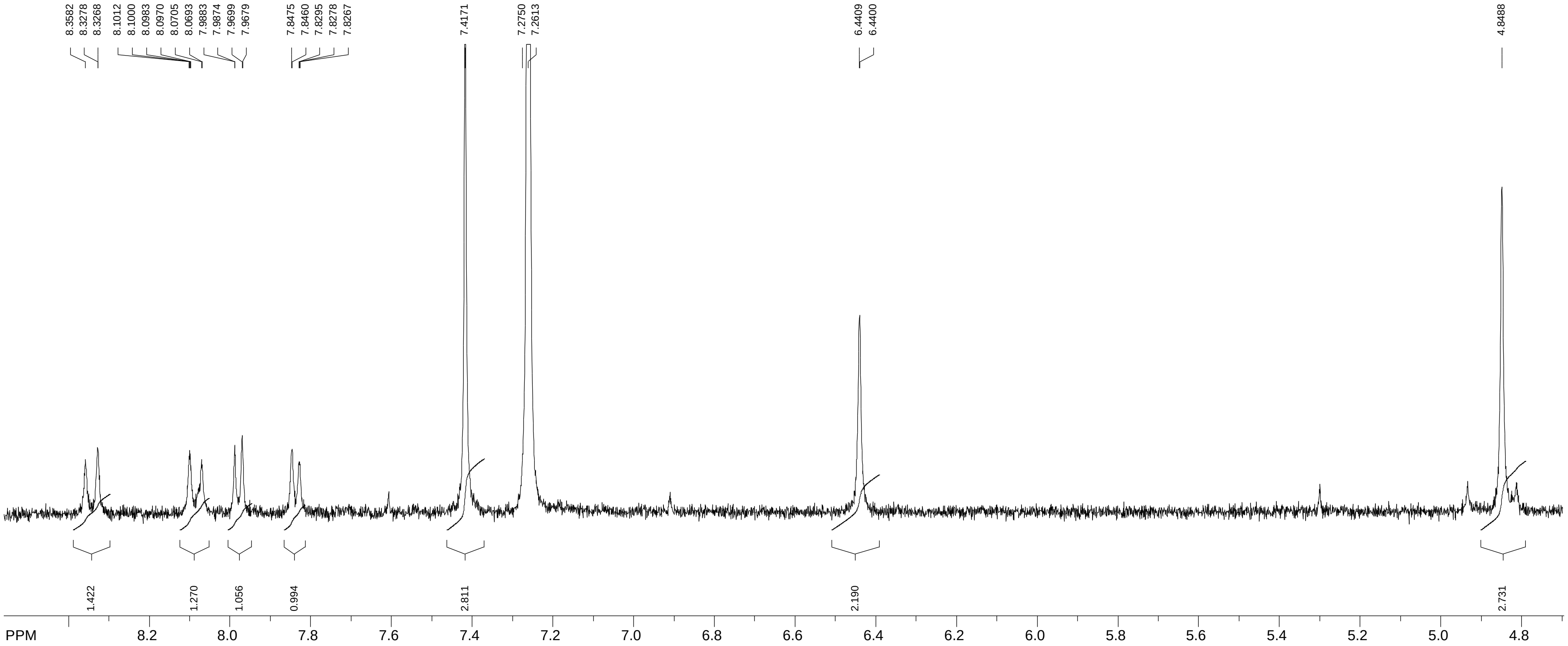

Figure S64. Part of the ^{1}H NMR ($CDCl_3$) spectrum of **23**.

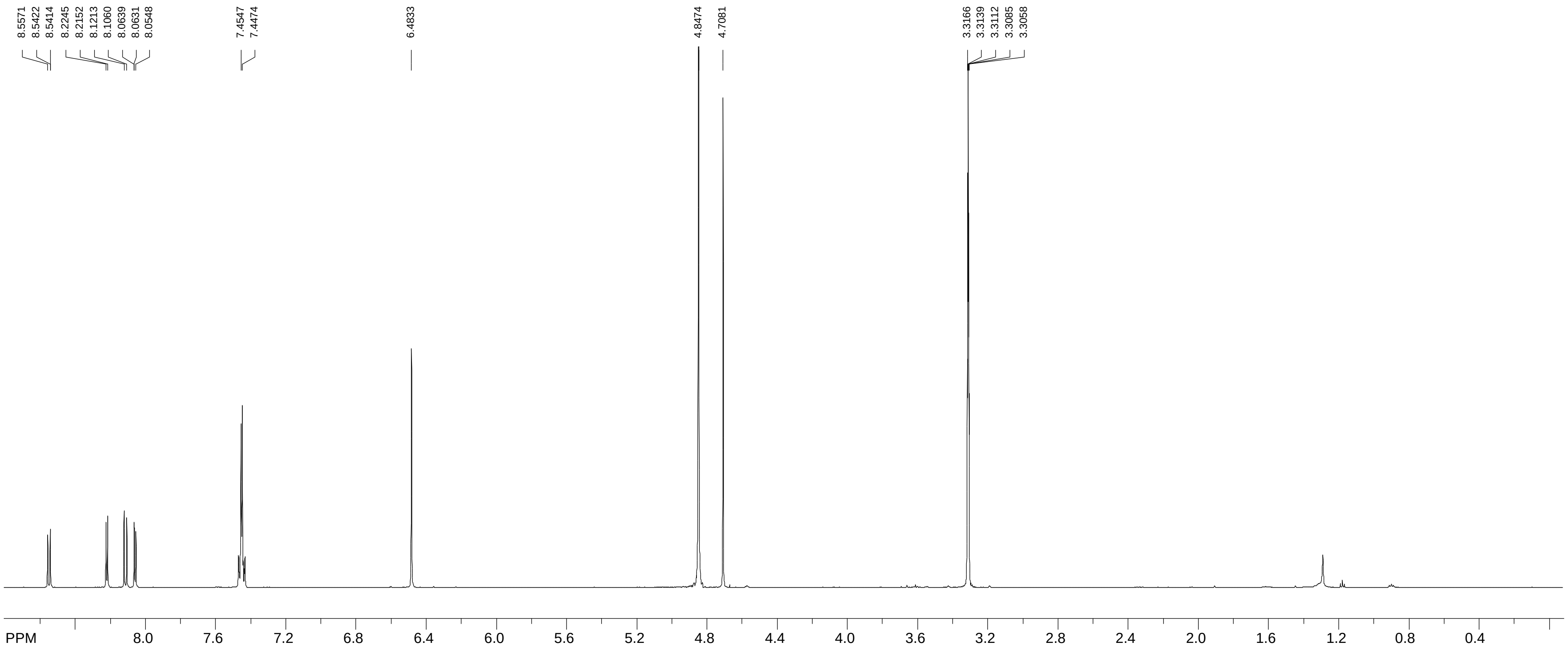

Figure S65. ^{1}H NMR (CD_3OD) spectrum of **23**.

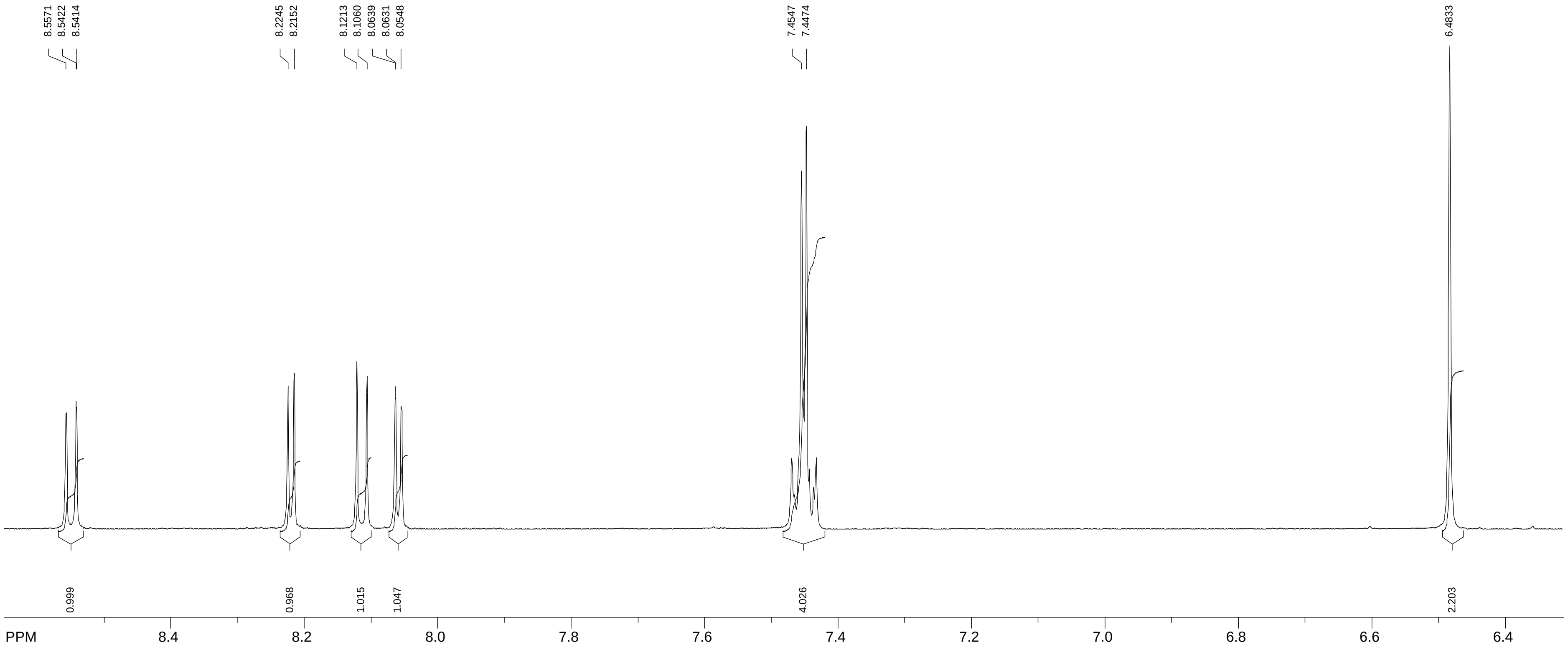

Figure S66. Part of the ^{1}H NMR (CD_3OD) spectrum of **23**.

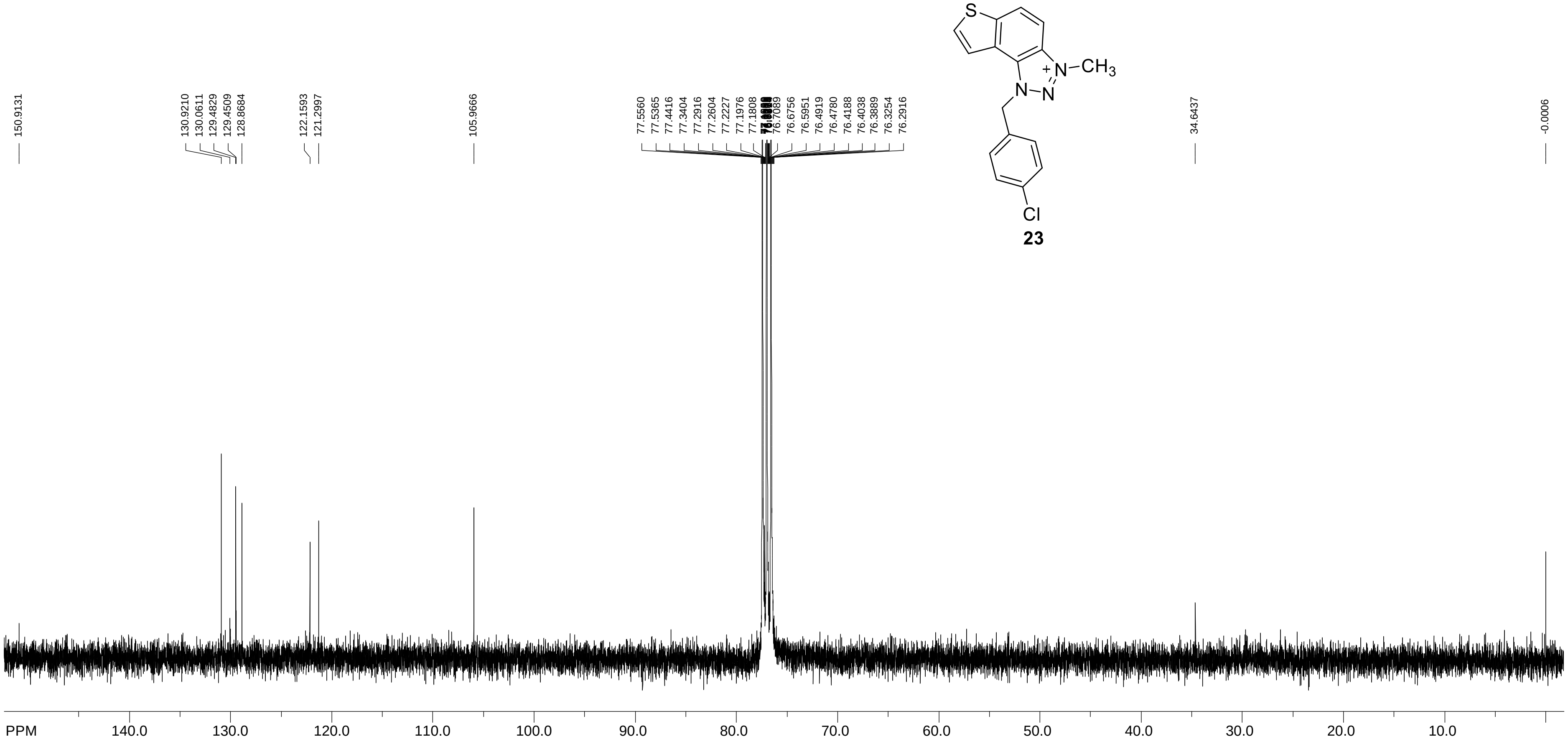

Figure S67. ^{13}C NMR ($CDCl_3$) spectrum of **23**.

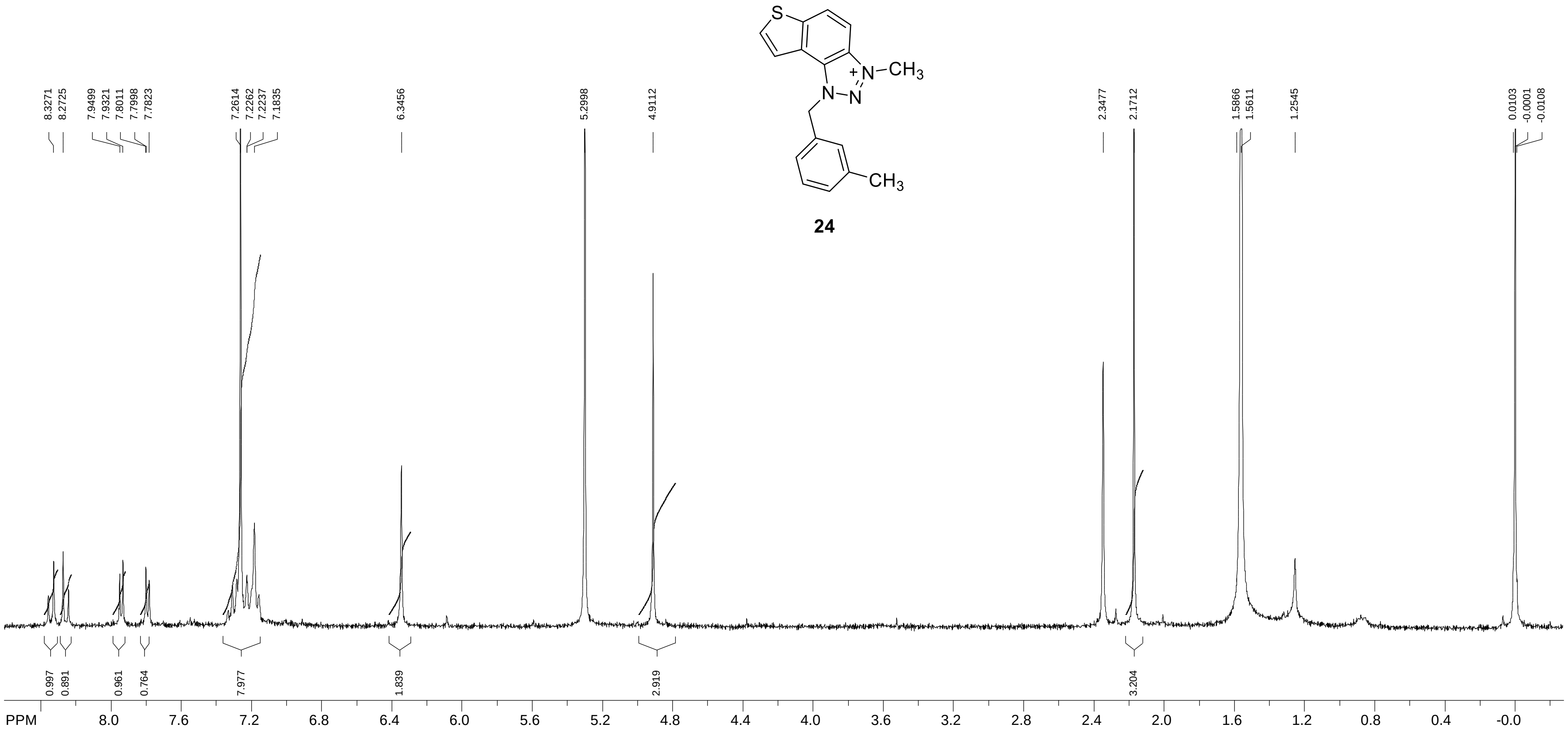

Figure S68. ^1H NMR ($CDCl_3$) spectrum of **24**.

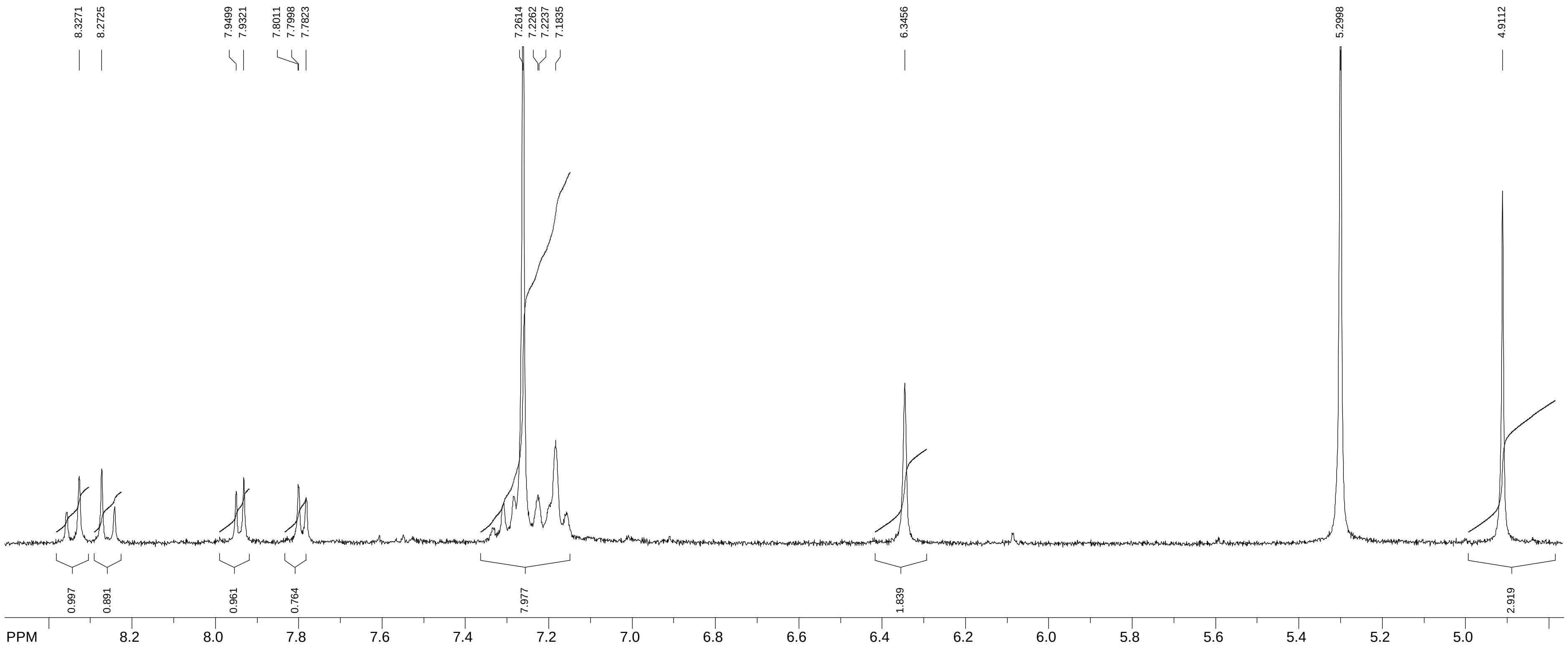

Figure S69. Part of the ^{1}H NMR ($CDCl_3$) spectrum of **24**.

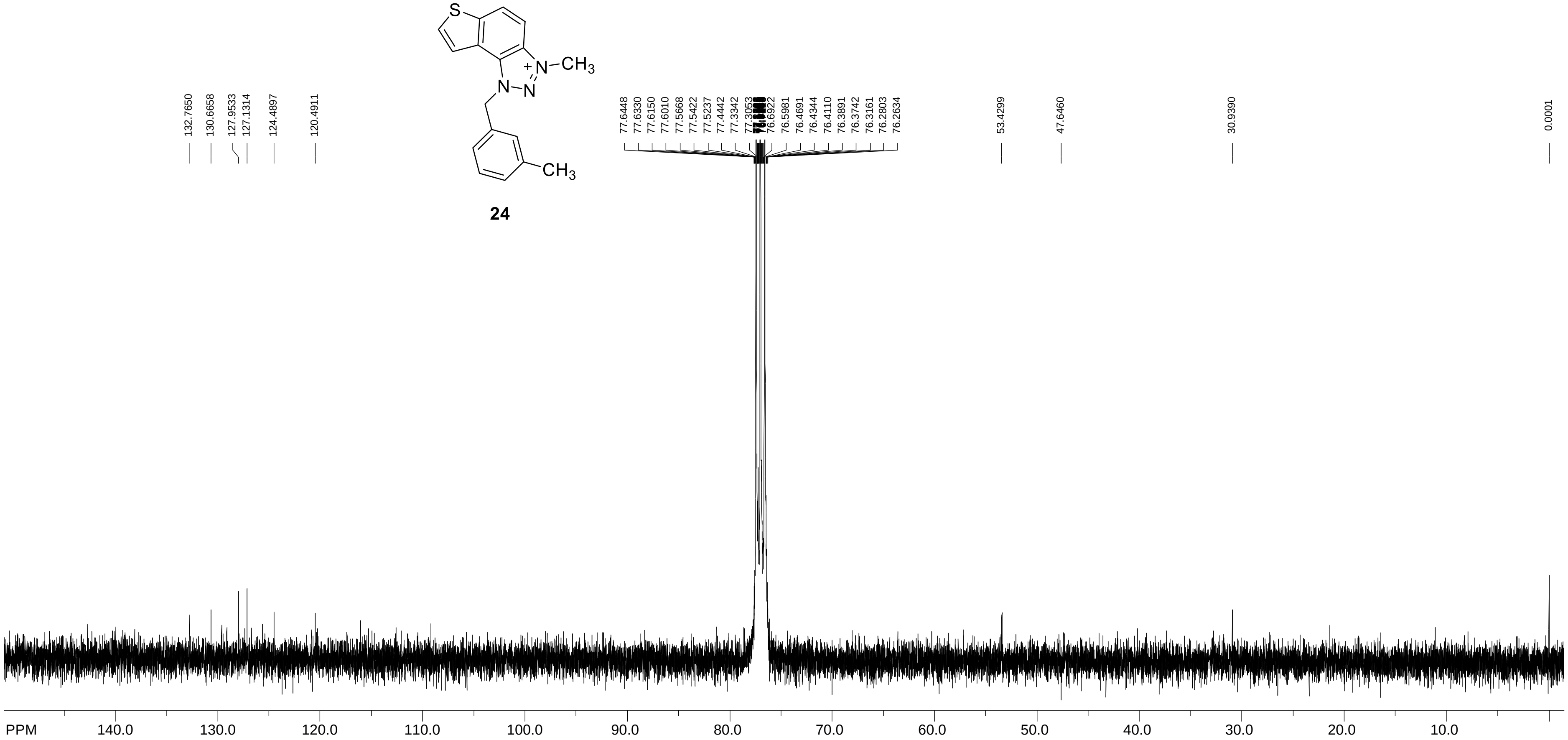

Figure S70. ^{13}C NMR ($CDCl_3$) spectrum of **24**.

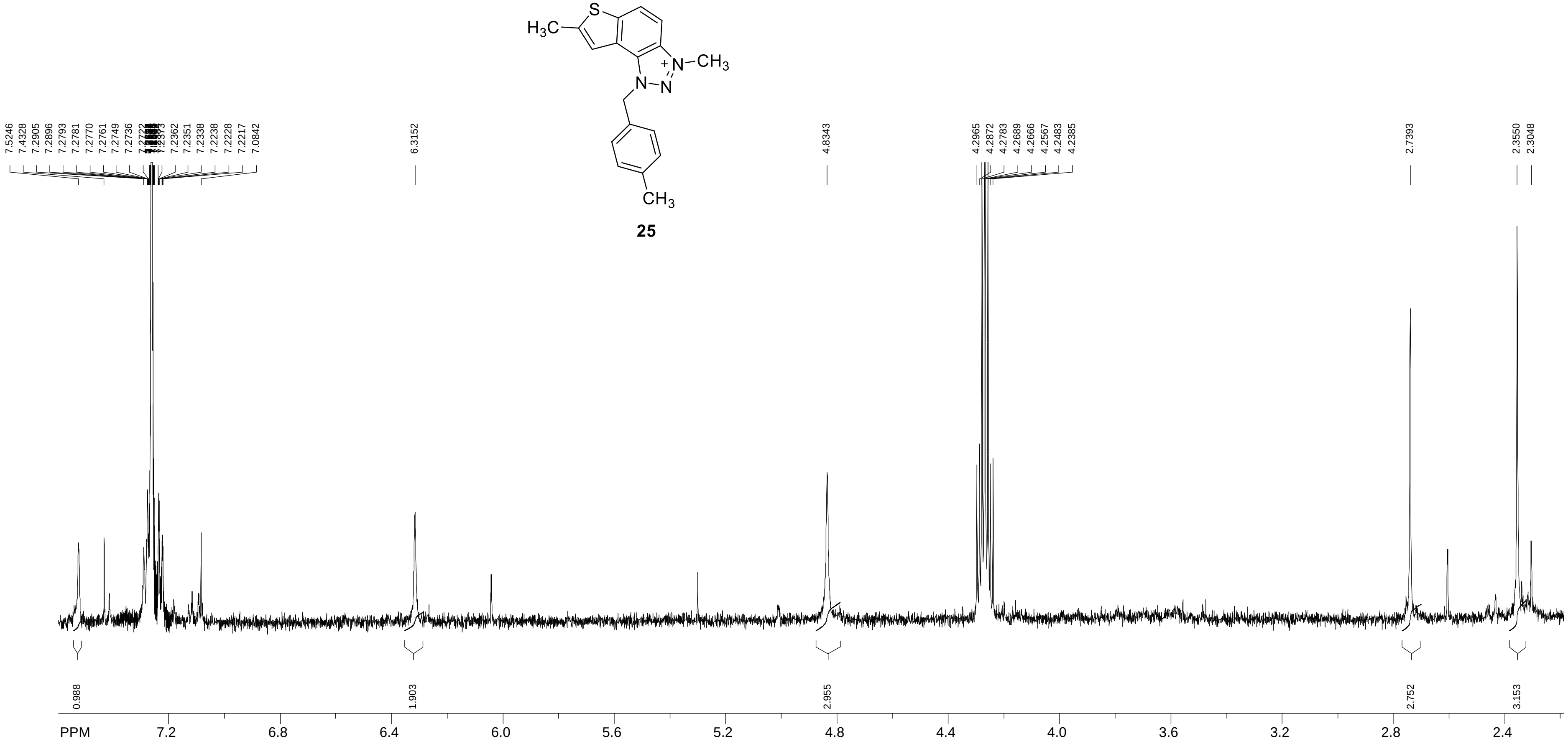

Figure S71. ^{1}H NMR ($CDCl_3$) spectrum of **25** (with traces of non-charged analogue **17**).

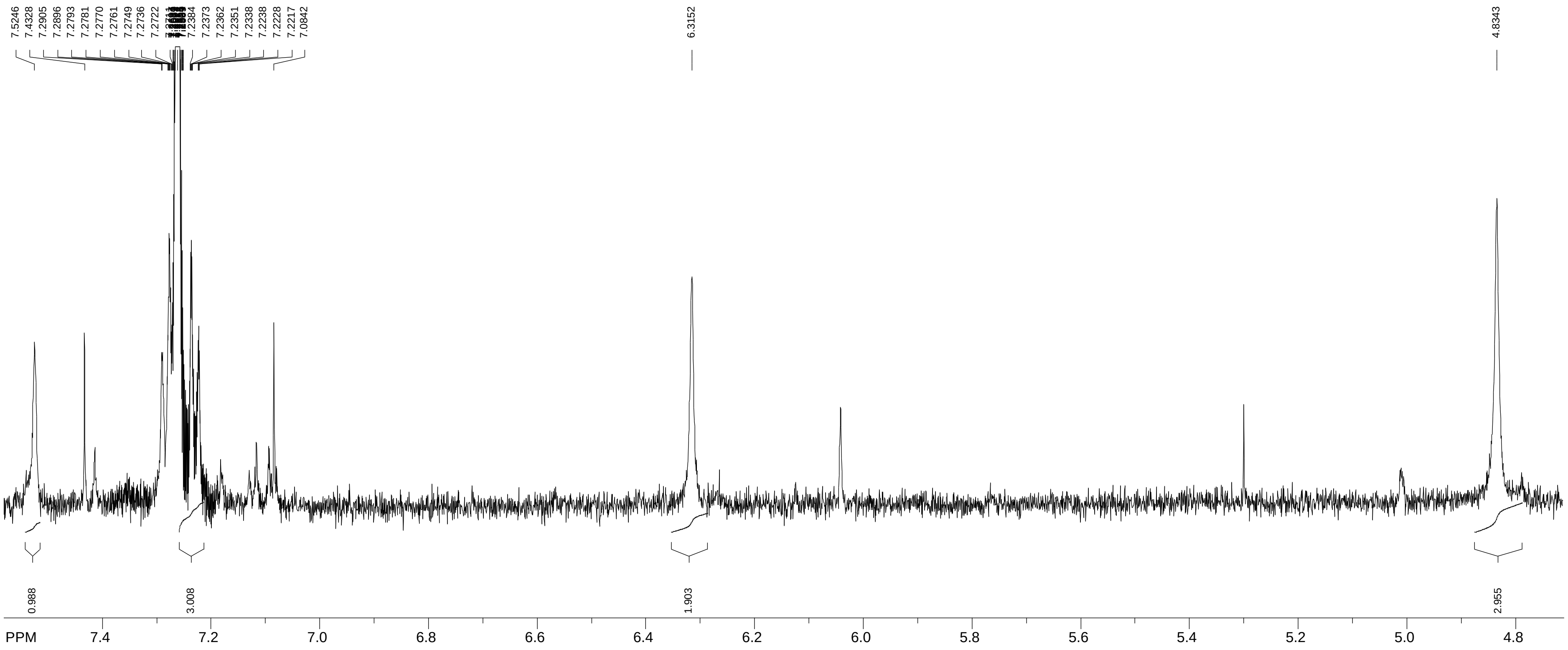

Figure S72. Part of the ^{1}H NMR ($CDCl_3$) spectrum of **25** (with traces of non-charged analogue **17**).

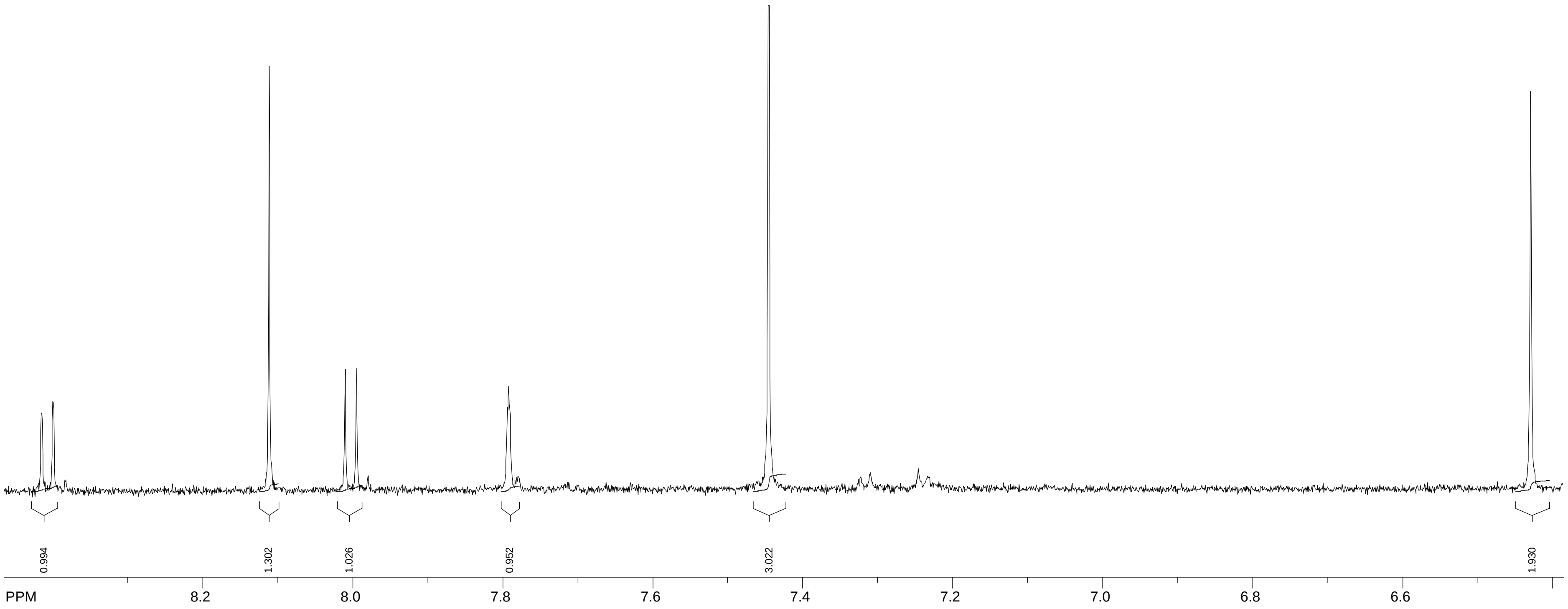

Figure S73. Part of the ^{1}H NMR (CD_3OD) spectrum of **25**.

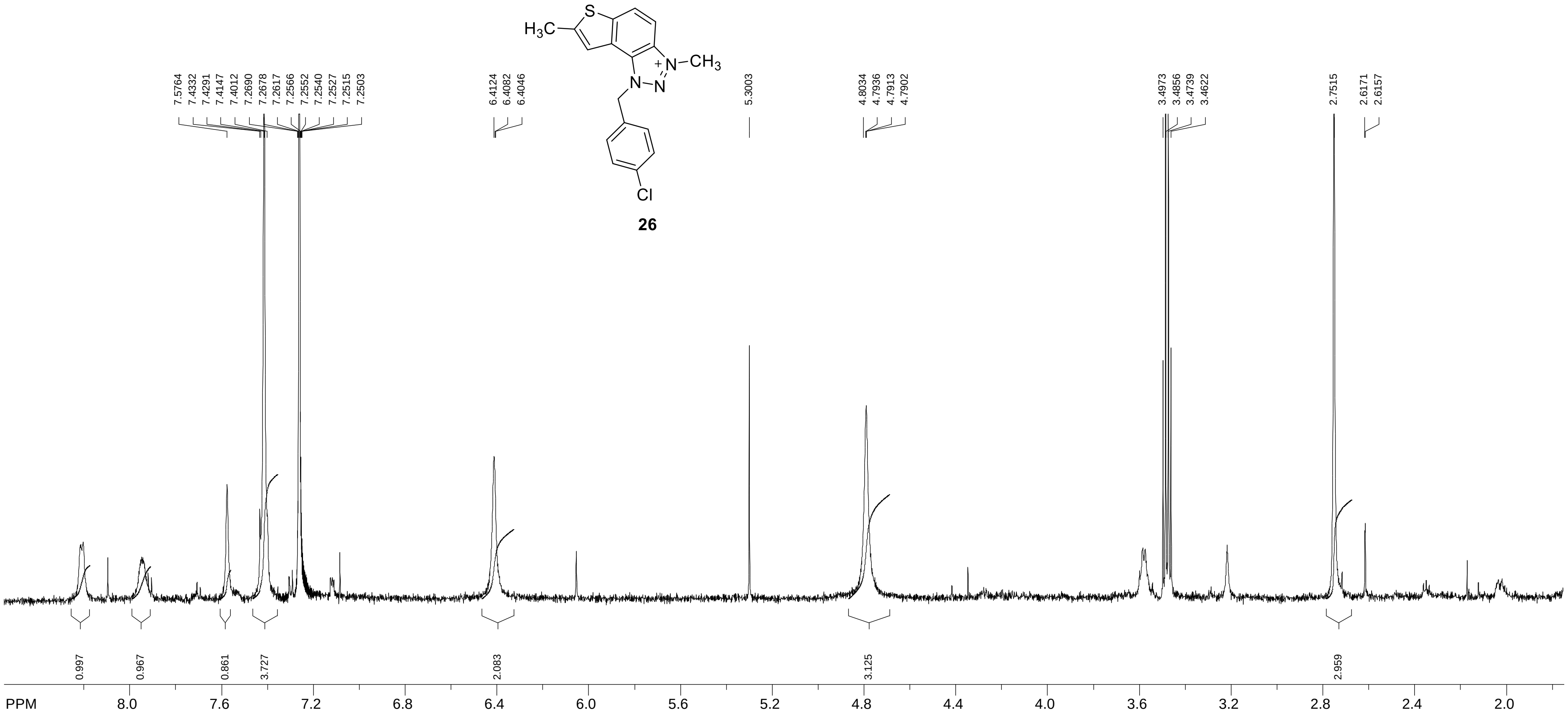

Figure S74. ^{1}H NMR ($CDCl_3$) spectrum of **26** (with traces of non-charged analogue **18**).

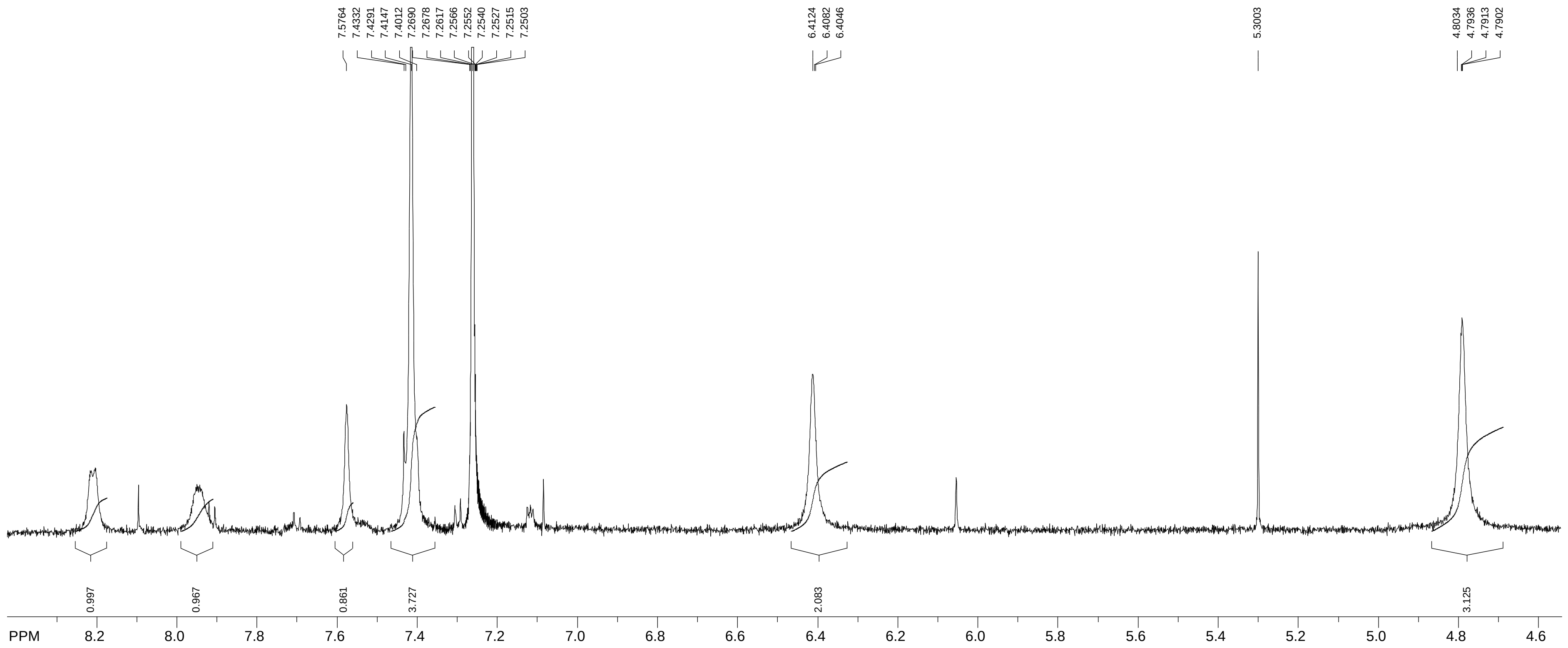

Figure S75. Part of the ^{1}H NMR ($CDCl_3$) spectrum of **26** (with traces of non-charged analogue **18**).

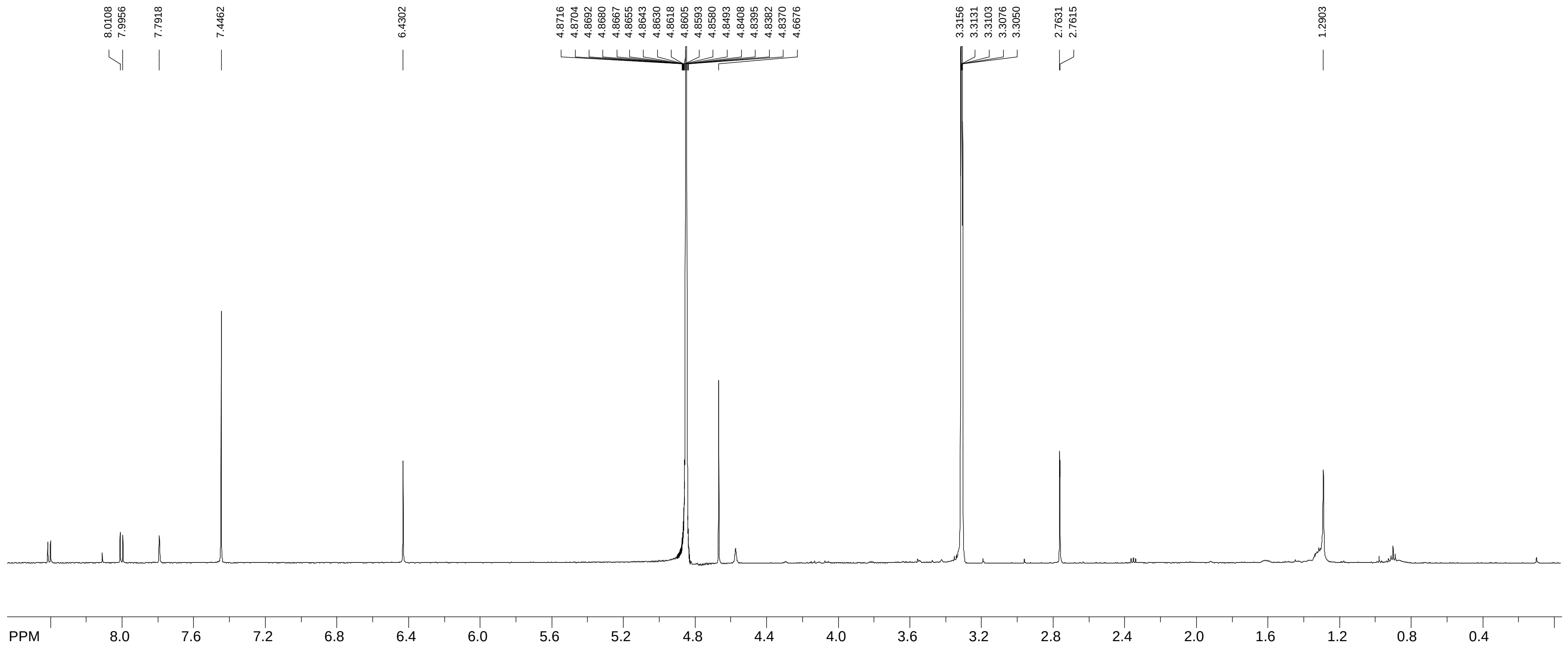

Figure S76. ^{1}H NMR (CD_3OD) spectrum of **26**.

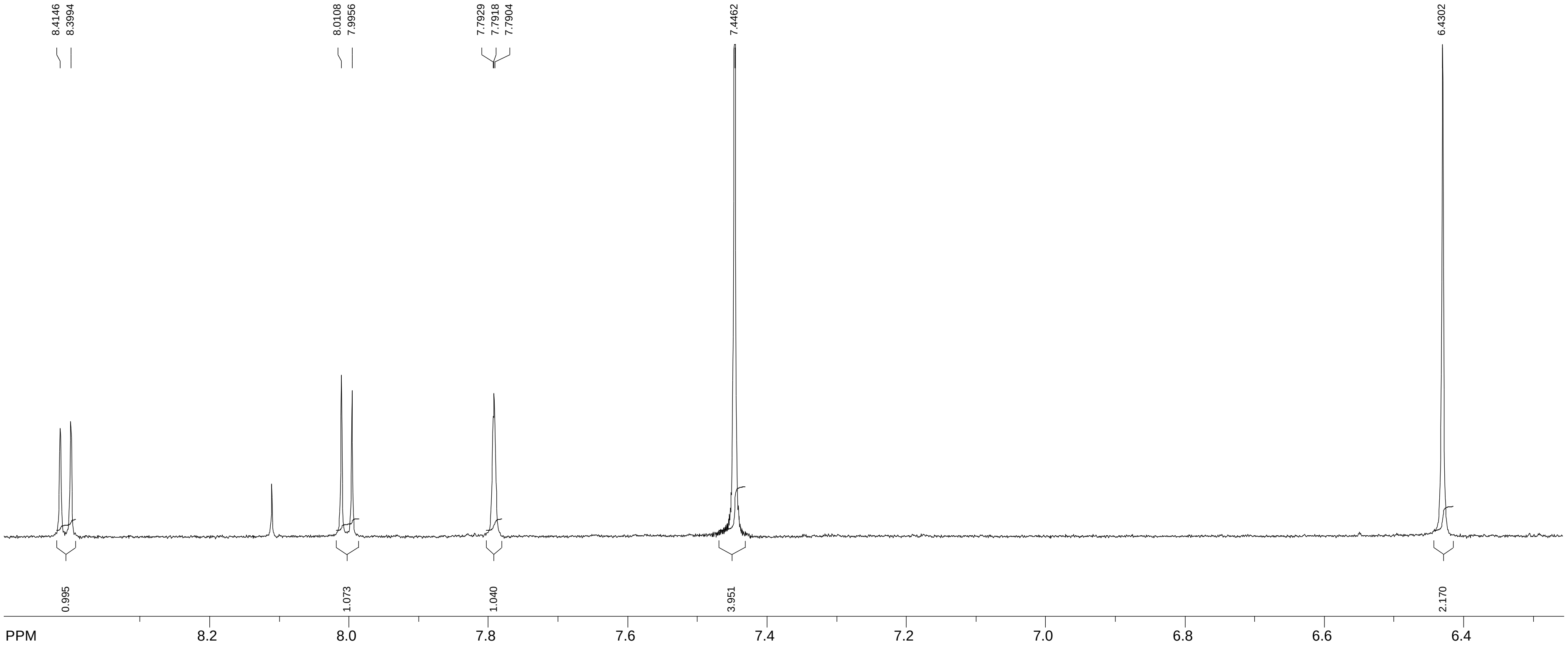

Figure S77. Part of the ^1H NMR (CD_3OD) spectrum of **26**.

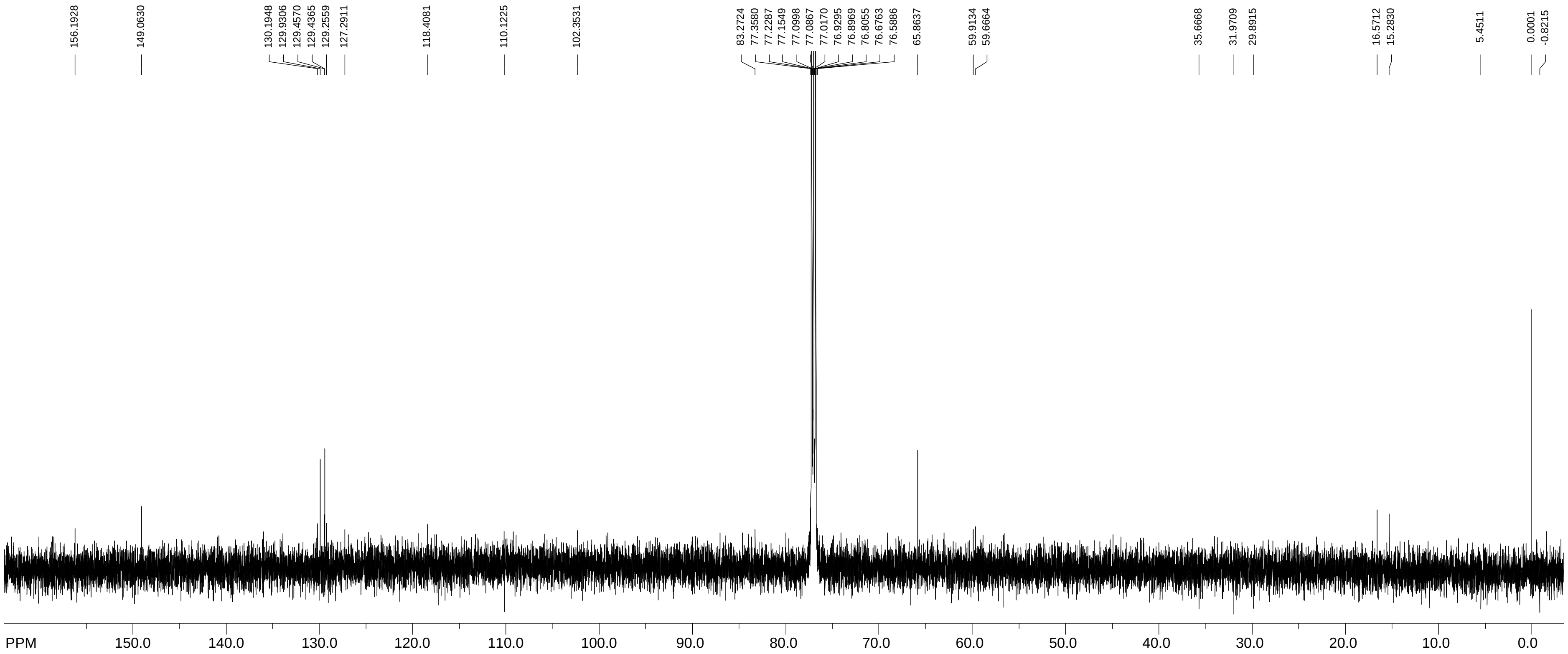

Figure S78. ^{13}C NMR ($CDCl_3$) spectrum of **26**.

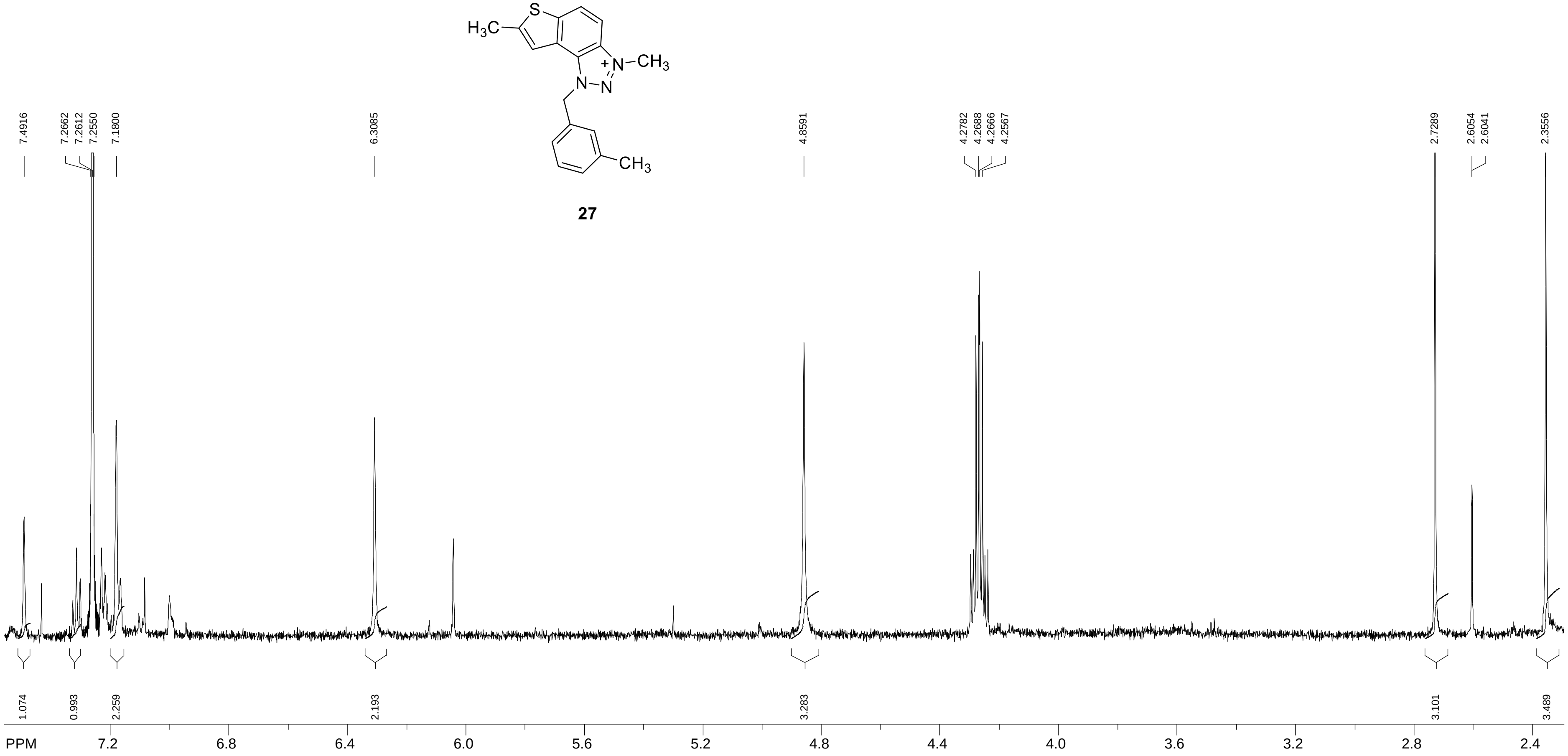

Figure S79. ^{1}H NMR ($CDCl_3$) spectrum of **27** (with traces of non-charged analogue **19**).

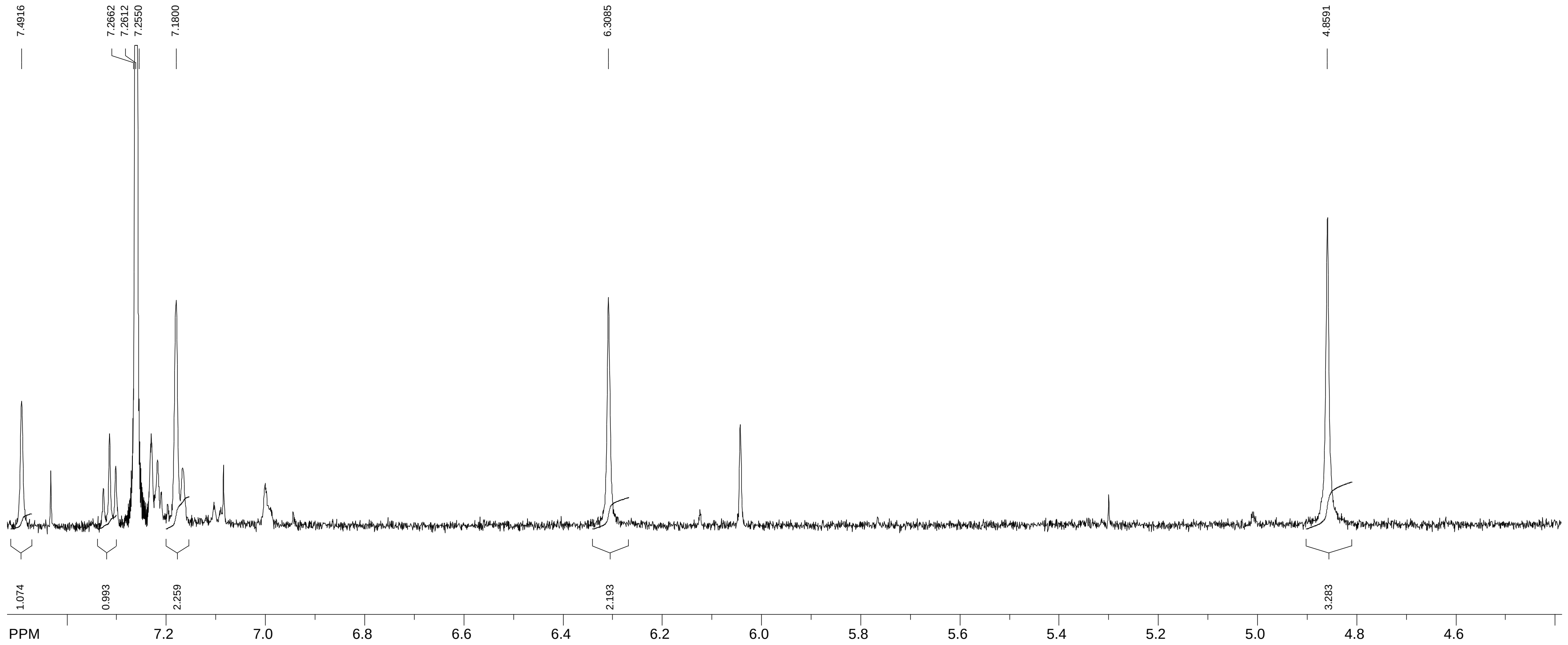

Figure S80. Part of the ^{1}H NMR ($CDCl_3$) spectrum of **27** (with traces of non-charged analogue **19**).

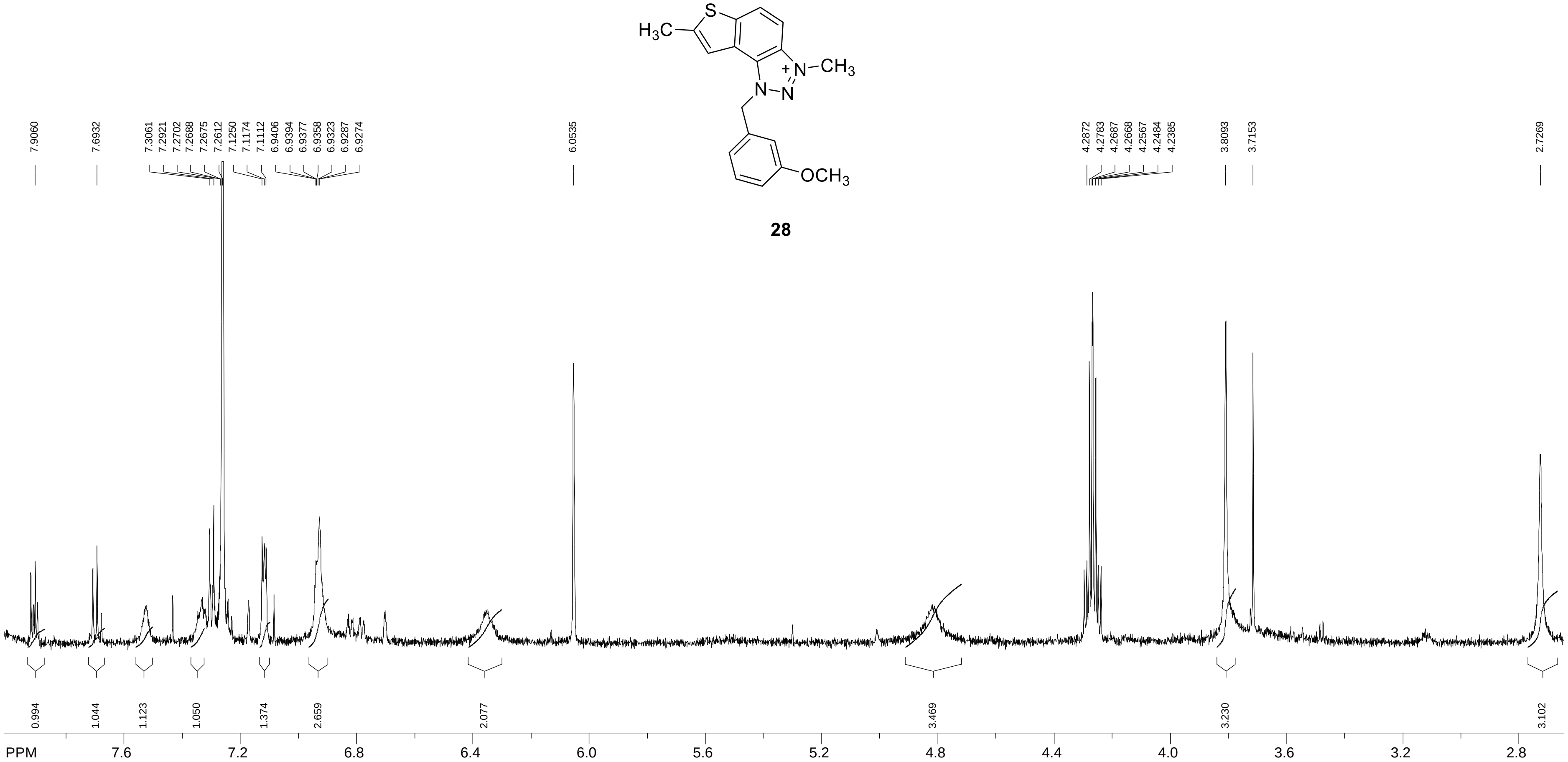

Figure S81. ^{1}H NMR ($CDCl_3$) spectrum of **28** (with certain proportion of non-charged analogue **20**).

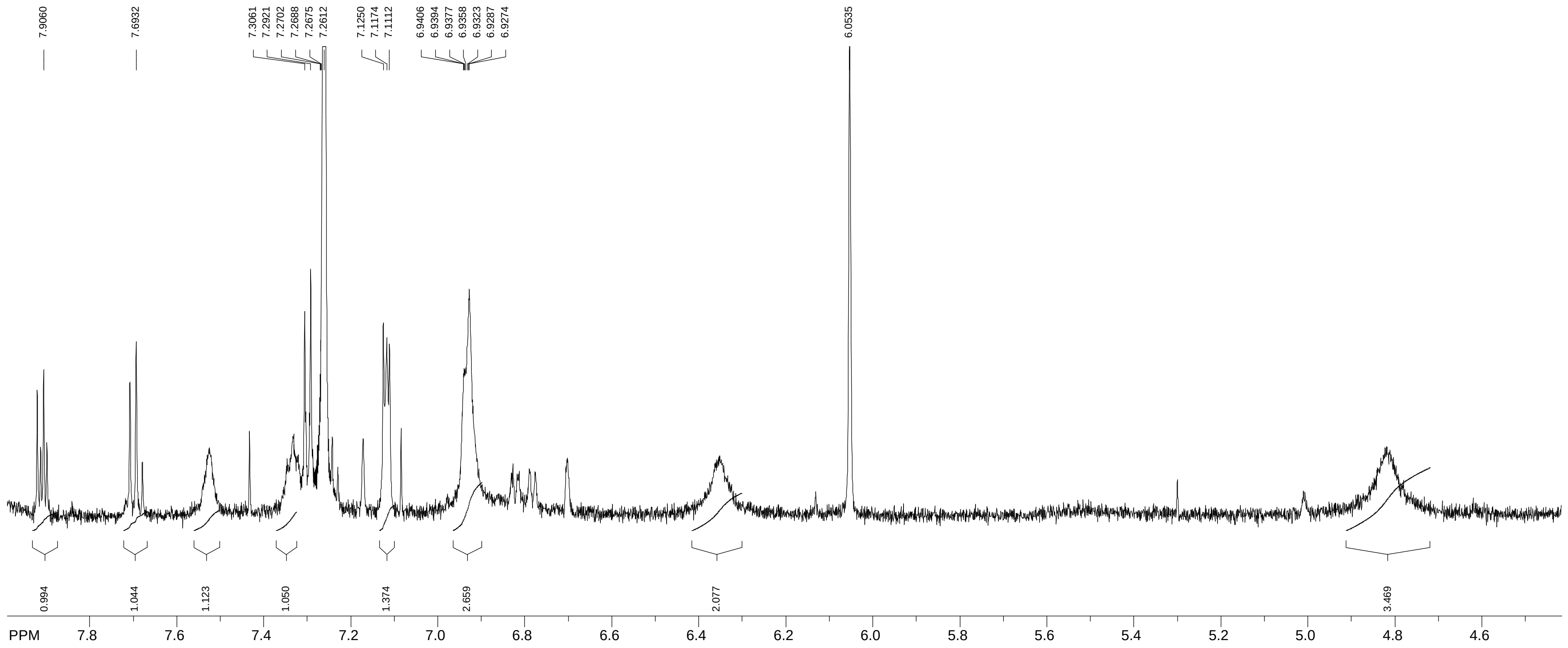

Figure S82. Part of the ^{1}H NMR ($CDCl_3$) spectrum of **28** (with certain proportion of non-charged analogue **20**).

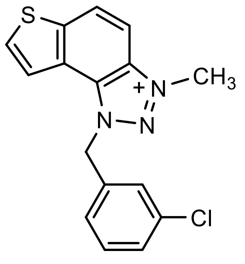

30

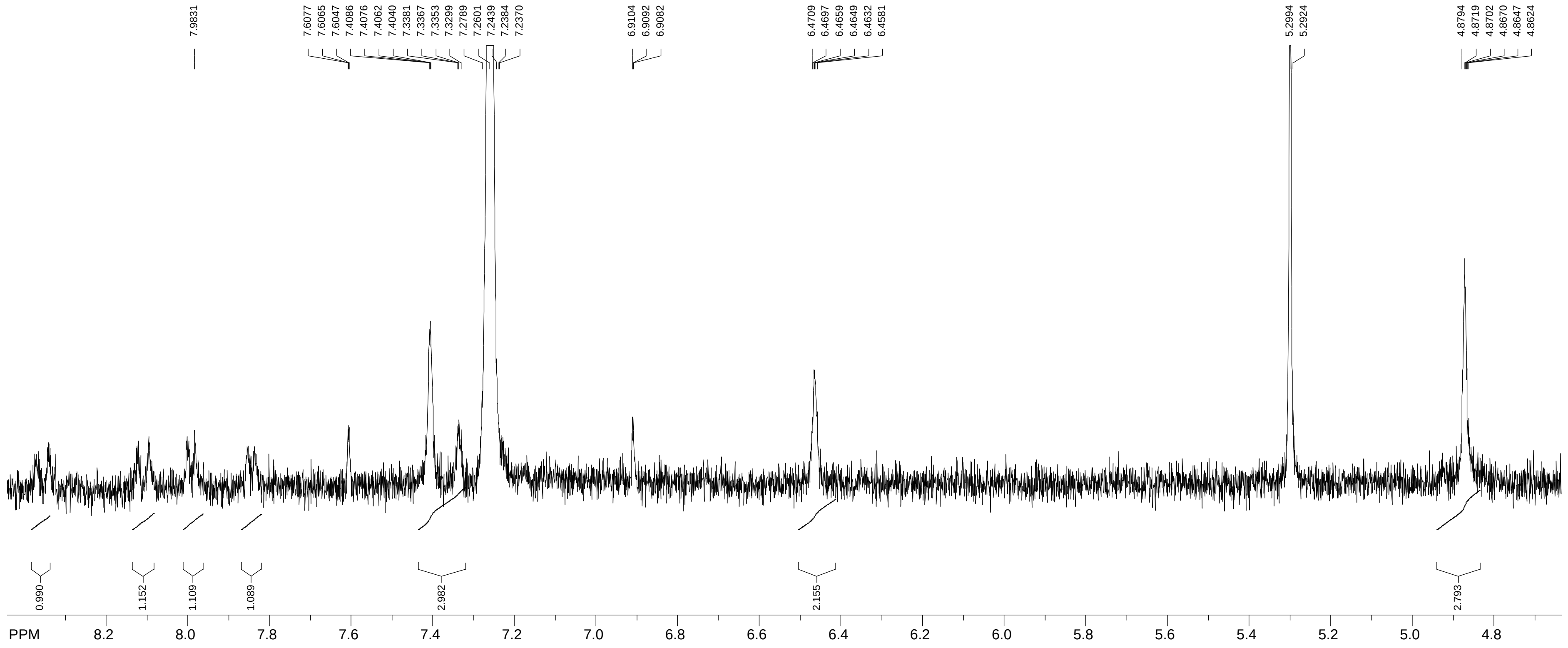

Figure S83. ^{1}H NMR ($CDCl_3$) spectrum of **30**.

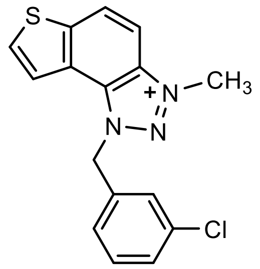

30

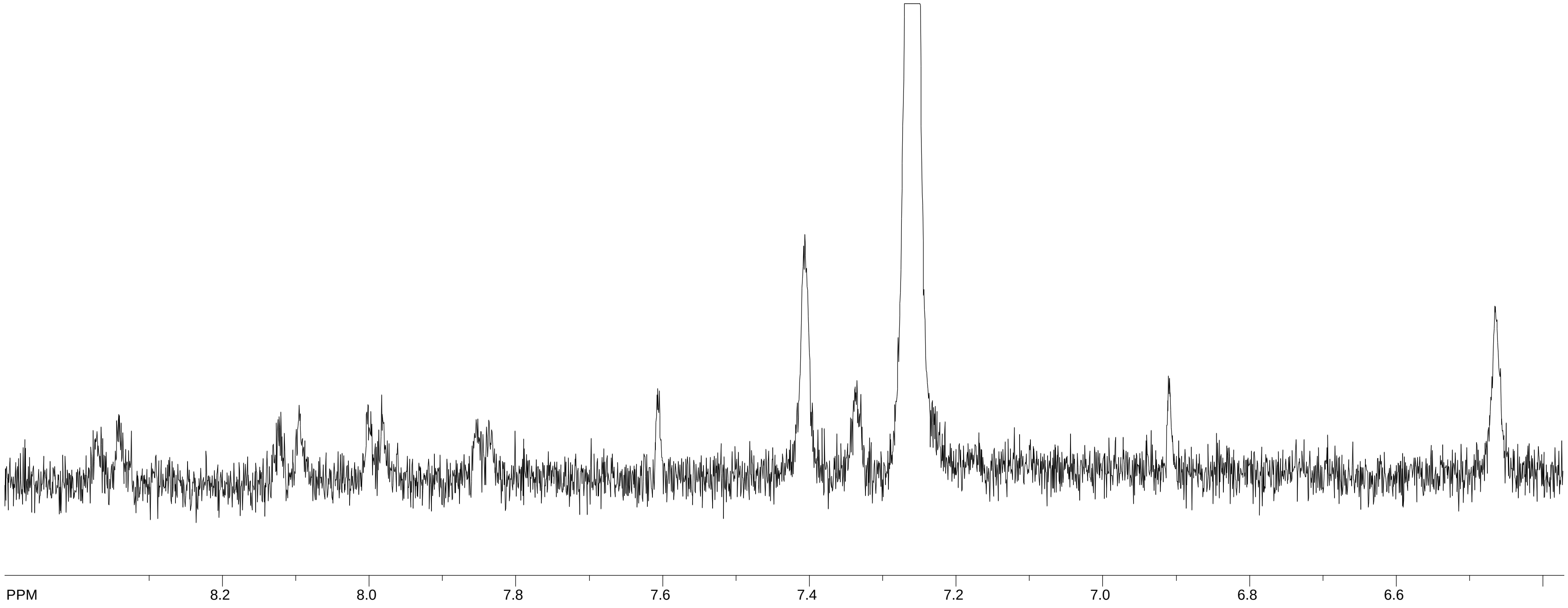

Figure S84. Aromatic part of the ^{1}H NMR ($CDCl_3$) spectrum of **30.**

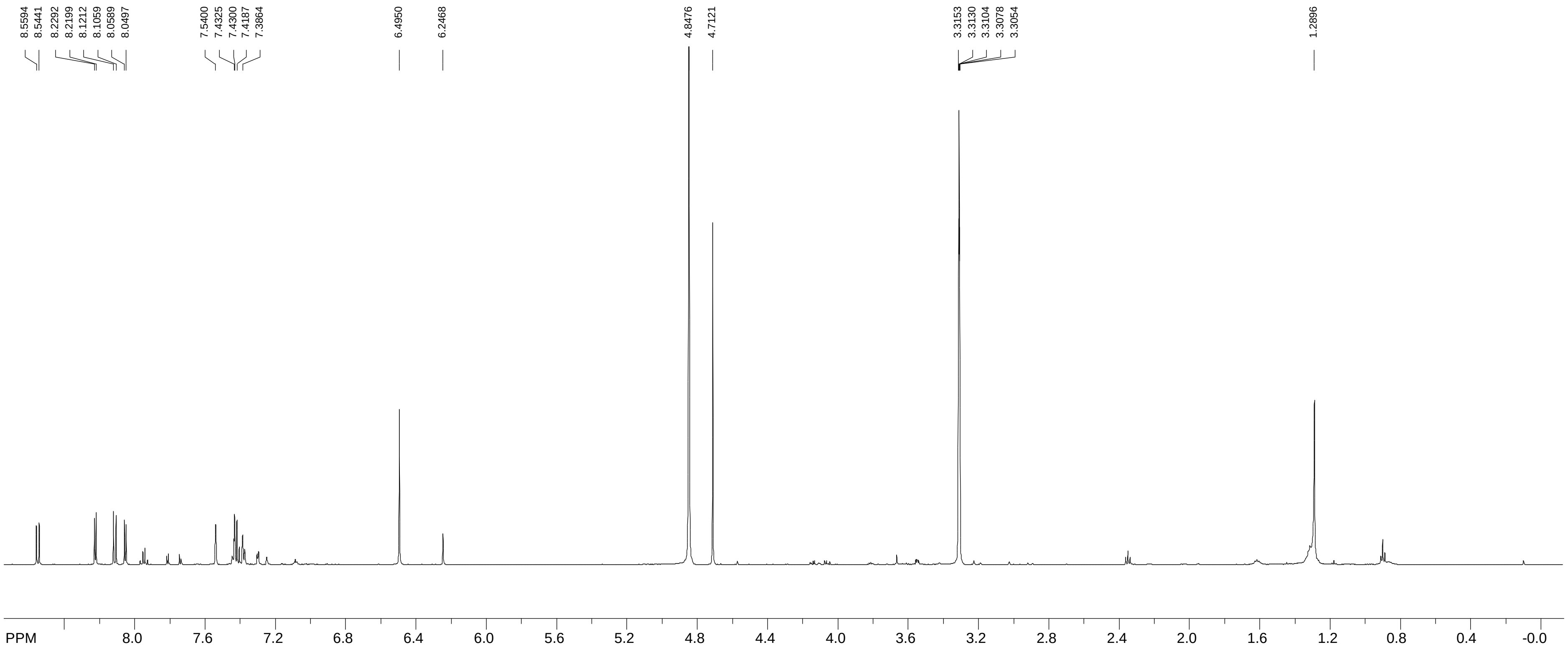

Figure S85. ^{1}H NMR (CD_3OD) spectrum of **30**.

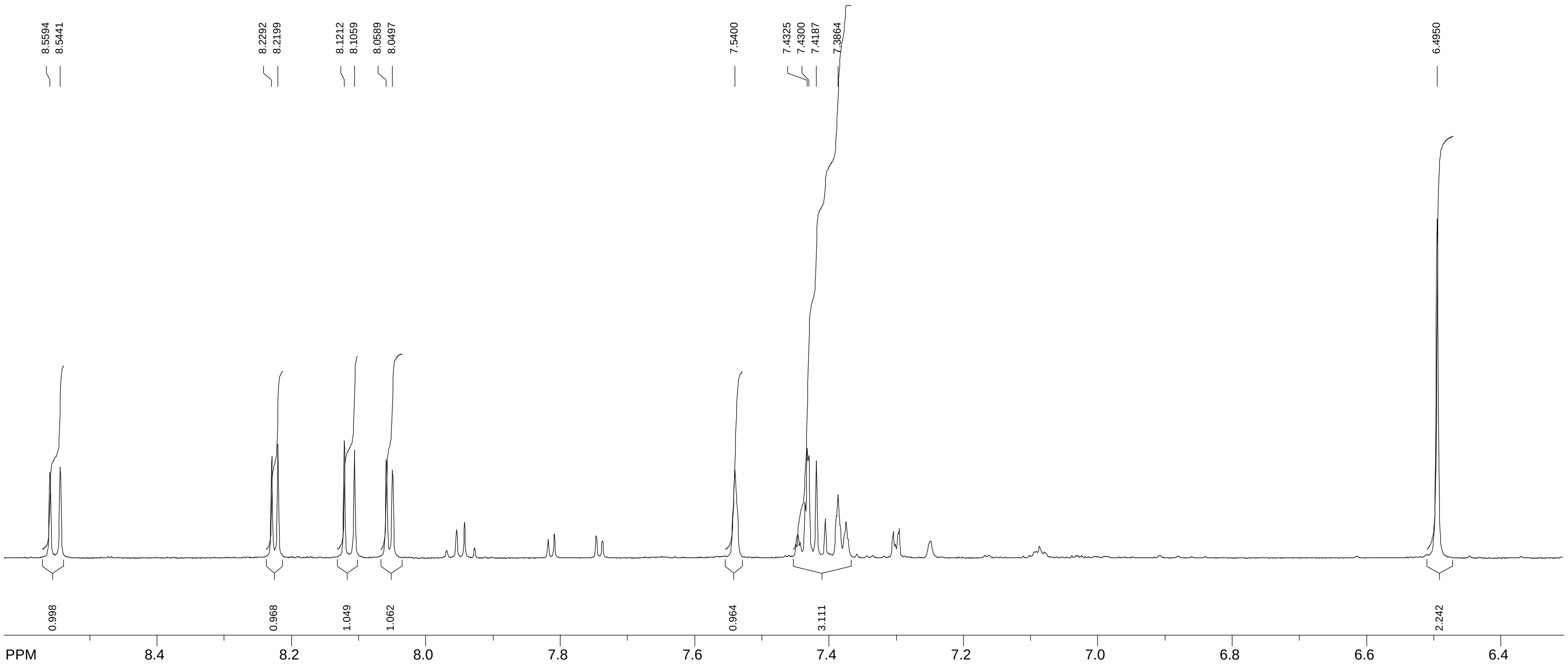

Figure S86. Part of the ^{1}H NMR (CD_3OD) spectrum of **30**.

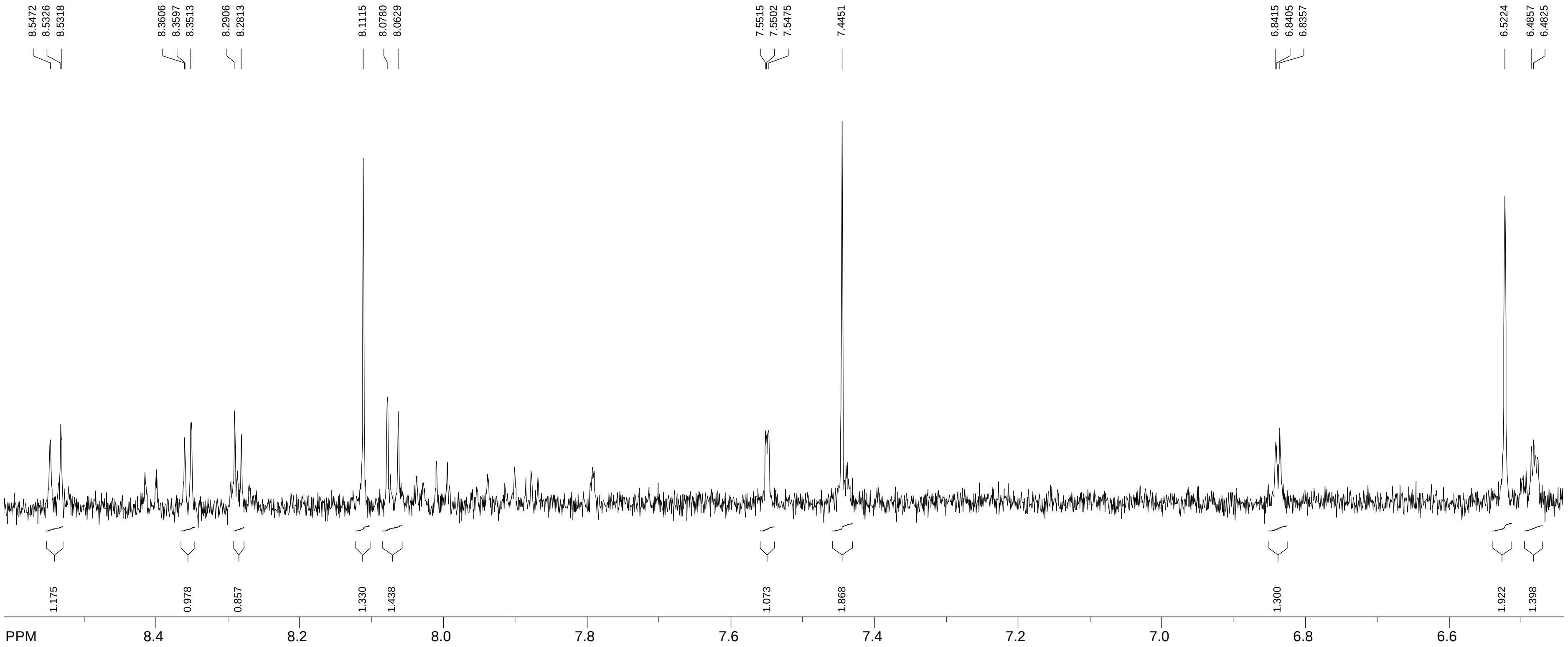

Figure S87. Part of the ^{1}H NMR (CD_3OD) spectrum of **31**.

2. Mass spectra and HRMS analyses of compounds 1–31

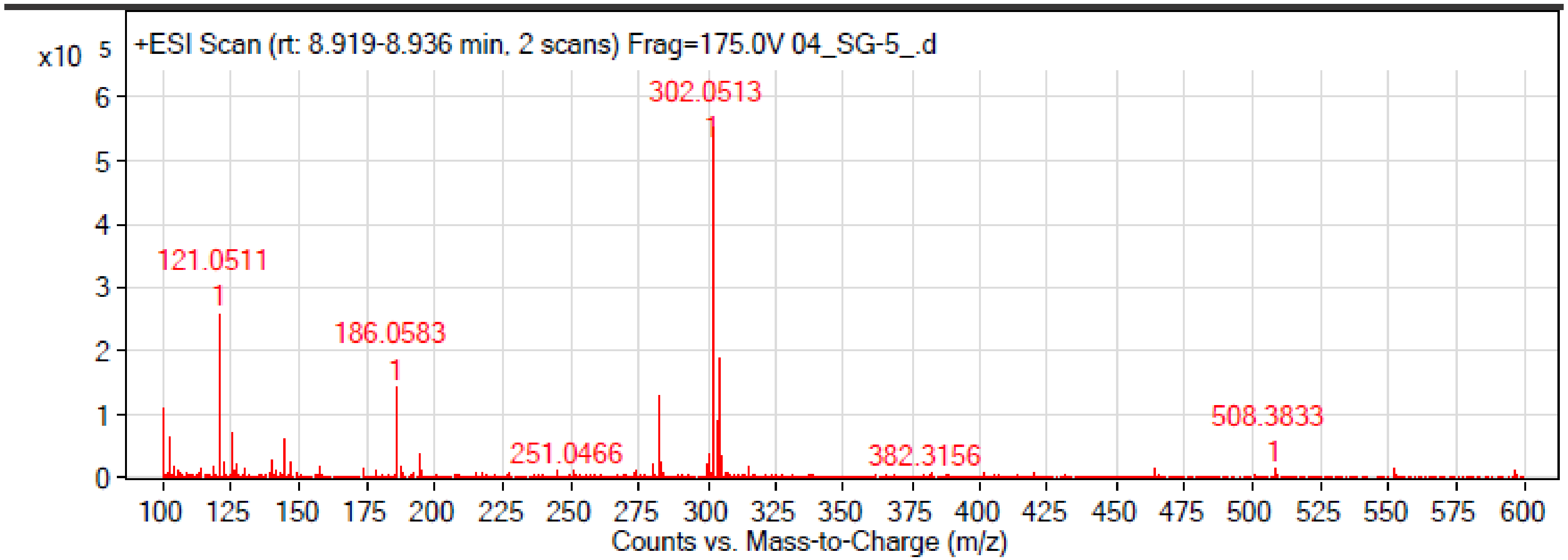

Formula Calculator Results

Formula	Best	Mass	Tgt Mass	Diff (ppm)	Ion Species	Score
C15 H12 Cl N3 S	True	301.0441	301.044	-0.21	C15 H13 Cl N3 S	98.35

Figure S88. Mass spectrum and HRMS analysis of compound **7**.

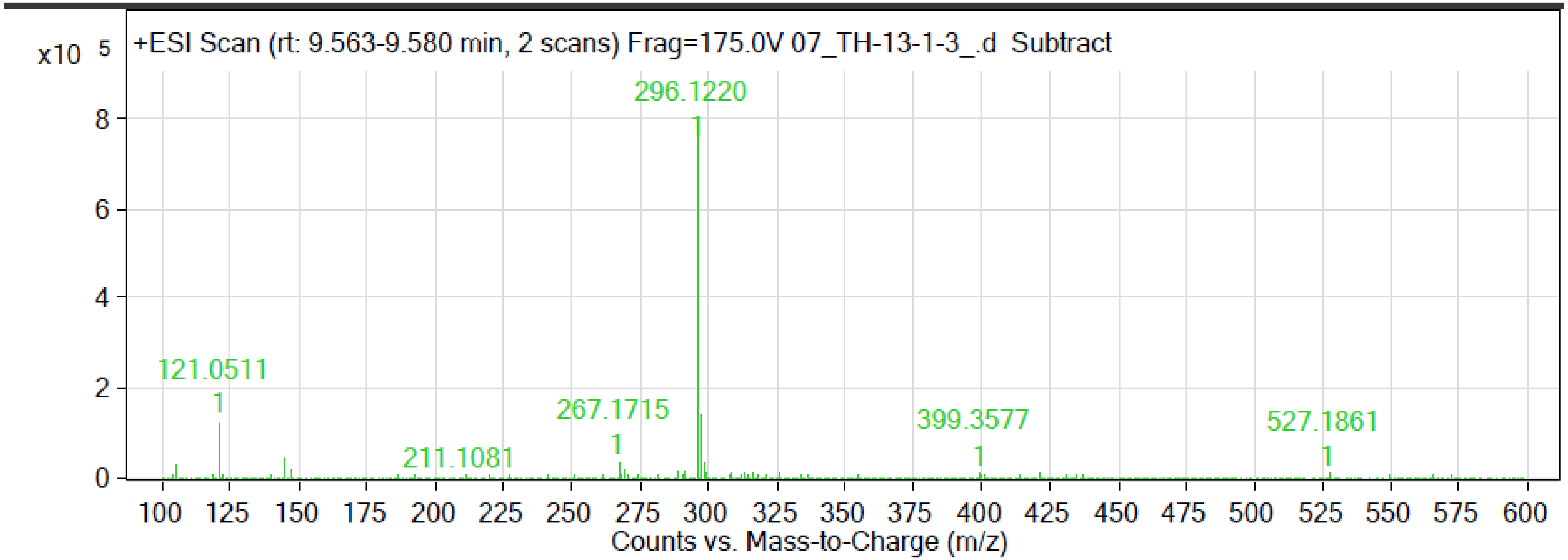

Formula Calculator Results

Formula	Best	Mass	Tgt Mass	Diff (ppm)	Ion Species	Score
C17 H17 N3 S	True	295.1147	295.1143	-1.3	C17 H18 N3 S	96.91

Figure S89. Mass spectrum and HRMS analysis of compound **9**.

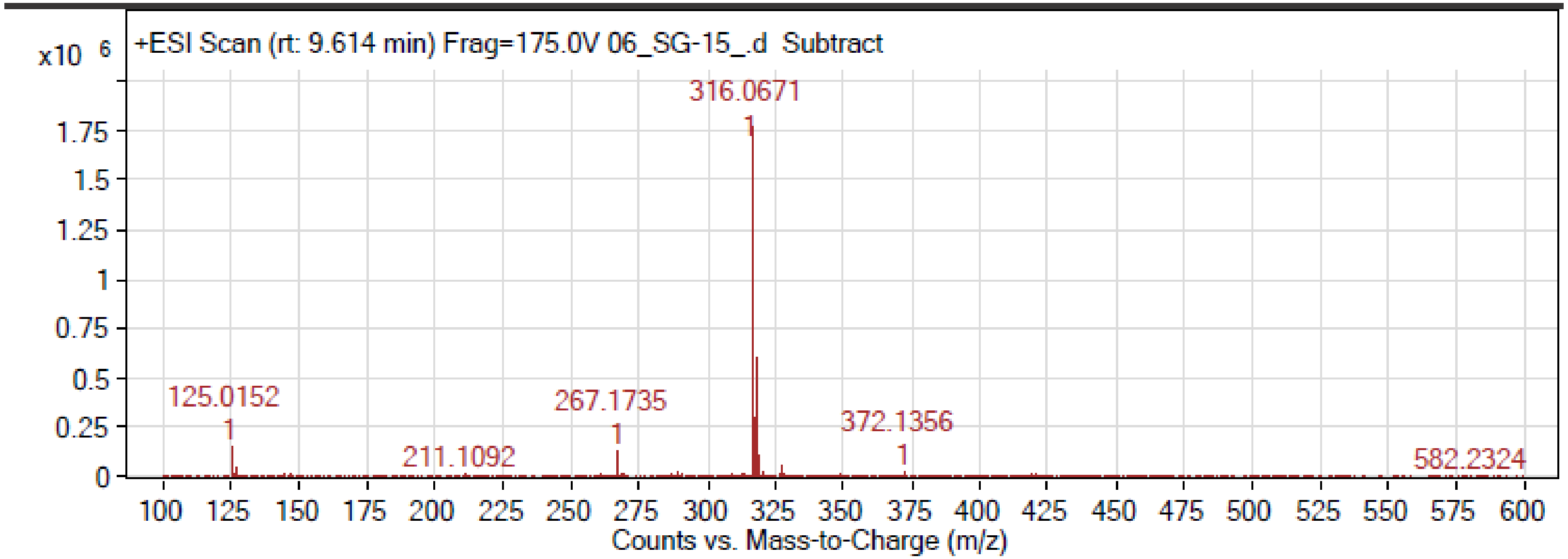

Formula Calculator Results

Formula	Best	Mass	Tgt Mass	Diff (ppm)	Ion Species	Score
C16 H14 Cl N3 S	True	315.0599	315.0597	-0.66	C16 H15 Cl N3 S	97.81

Figure S90. Mass spectrum and HRMS analysis of compound **10**.

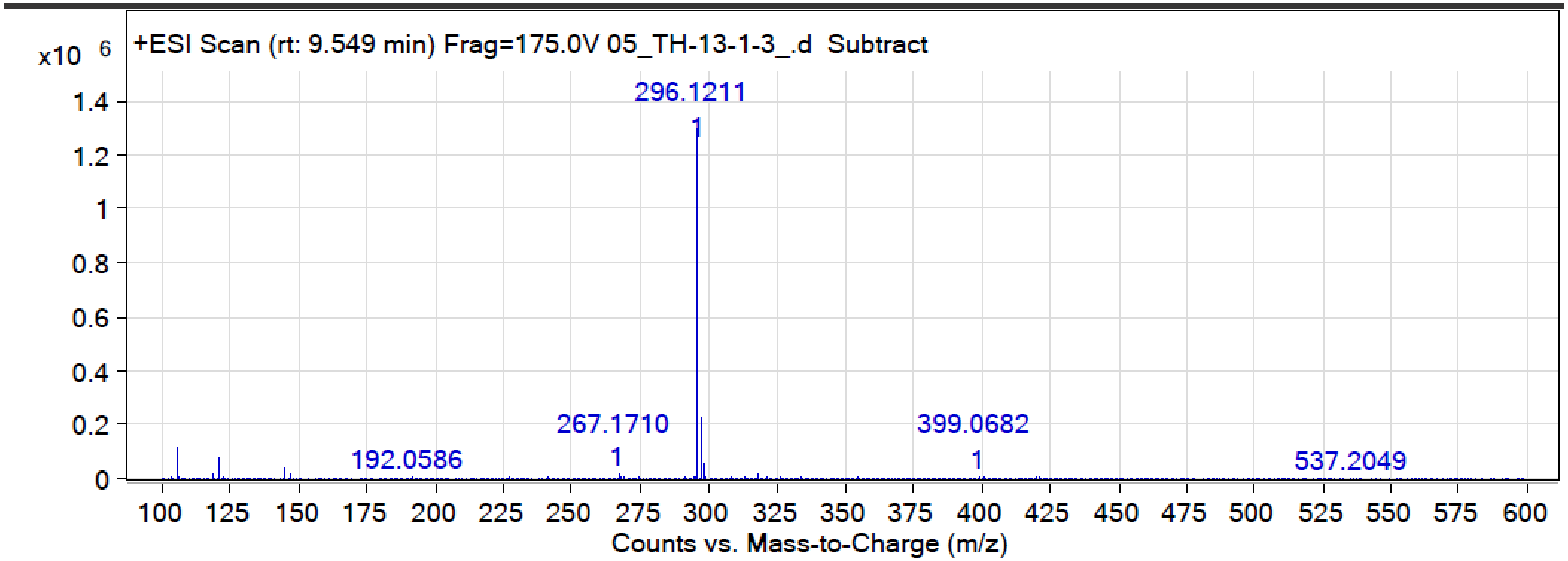

Formula Calculator Results

Formula	Best	Mass	Tgt Mass	Diff (ppm)	Ion Species	Score
C17 H17 N3 S	True	295.1139	295.1143	1.55	C17 H18 N3 S	96.33

Figure S81. Mass spectrum and HRMS analysis of compound **11**.

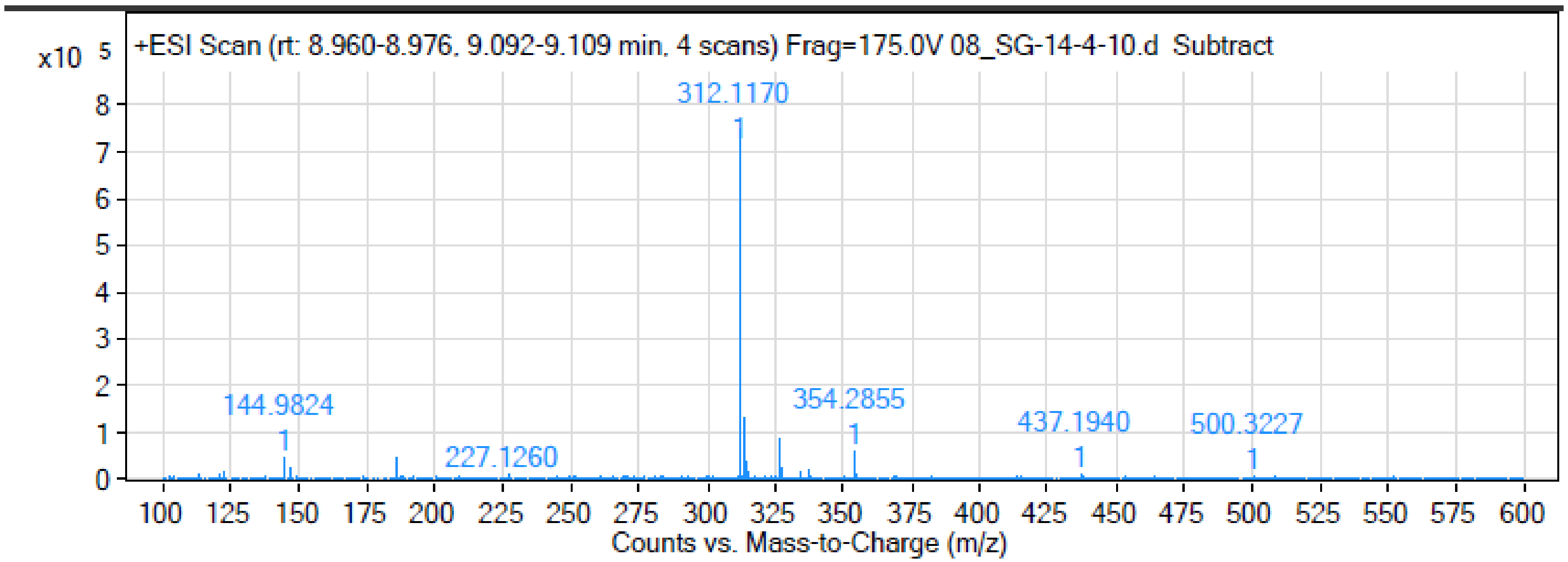

Formula Calculator Results

Formula	Best	Mass	Tgt Mass	Diff (ppm)	Ion Species	Score
C17 H17 N3 O S	True	311.1098	311.1092	-1.75	C17 H18 N3 O S	96.36

Figure S92. Mass spectrum and HRMS analysis of compound **12**.

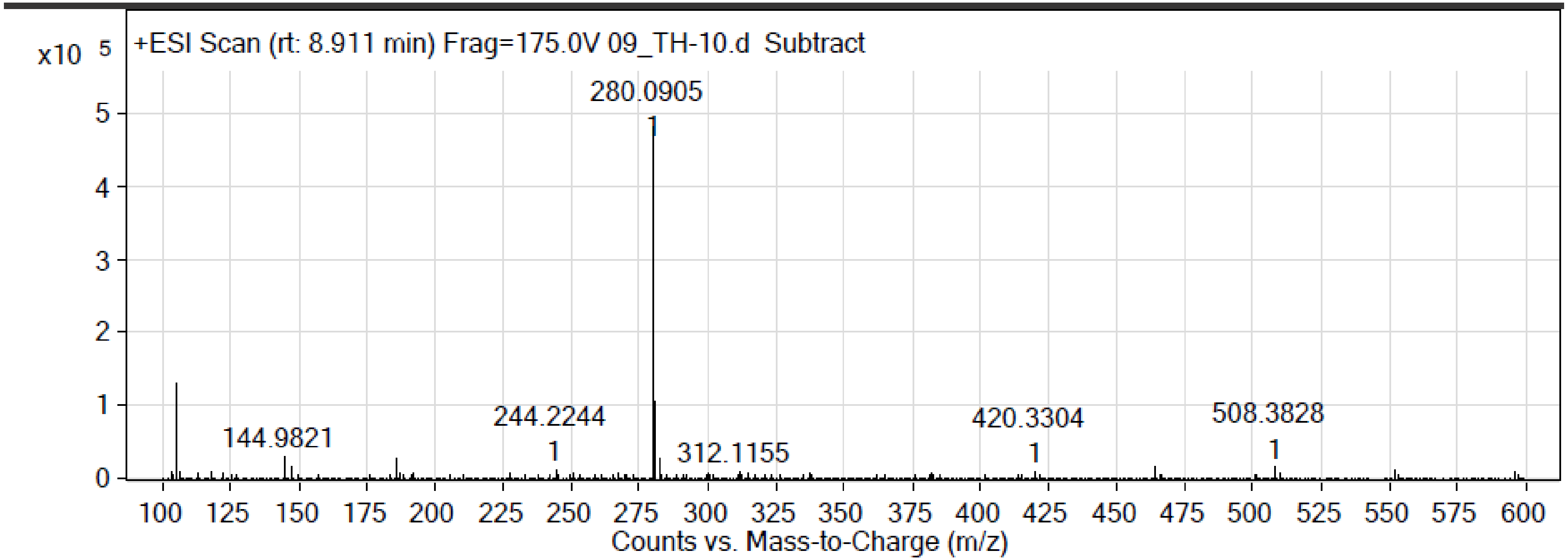

Formula Calculator Results

Formula	Best	Mass	Tgt Mass	Diff (ppm)	Ion Species	Score
C16 H13 N3 S	True	279.0832	279.083	-0.5	C16 H14 N3 S	97.87

Figure S93. Mass spectrum and HRMS analysis of compound **14**.

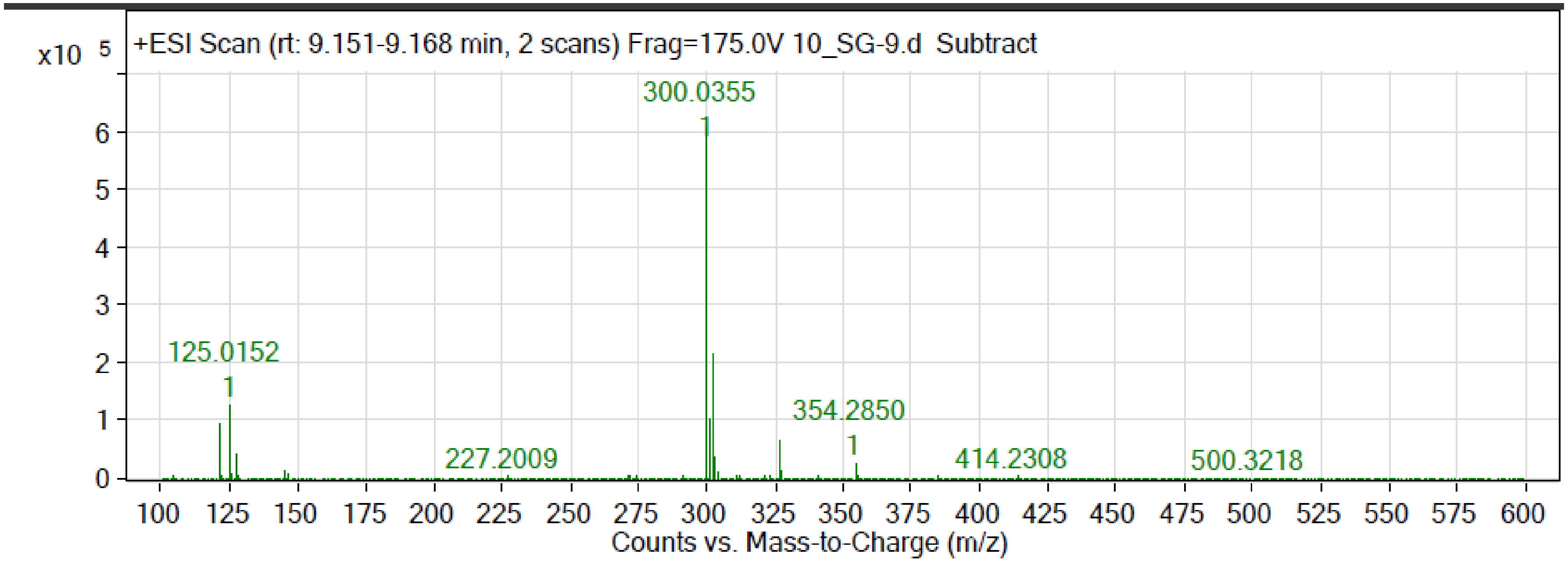

Formula Calculator Results

Formula	Best	Mass	Tgt Mass	Diff (ppm)	Ion Species	Score
C15 H10 Cl N3 S	True	299.0283	299.0284	0.48	C15 H11 Cl N3 S	99.22

Figure S94. Mass spectrum and HRMS analysis of compound **15**.

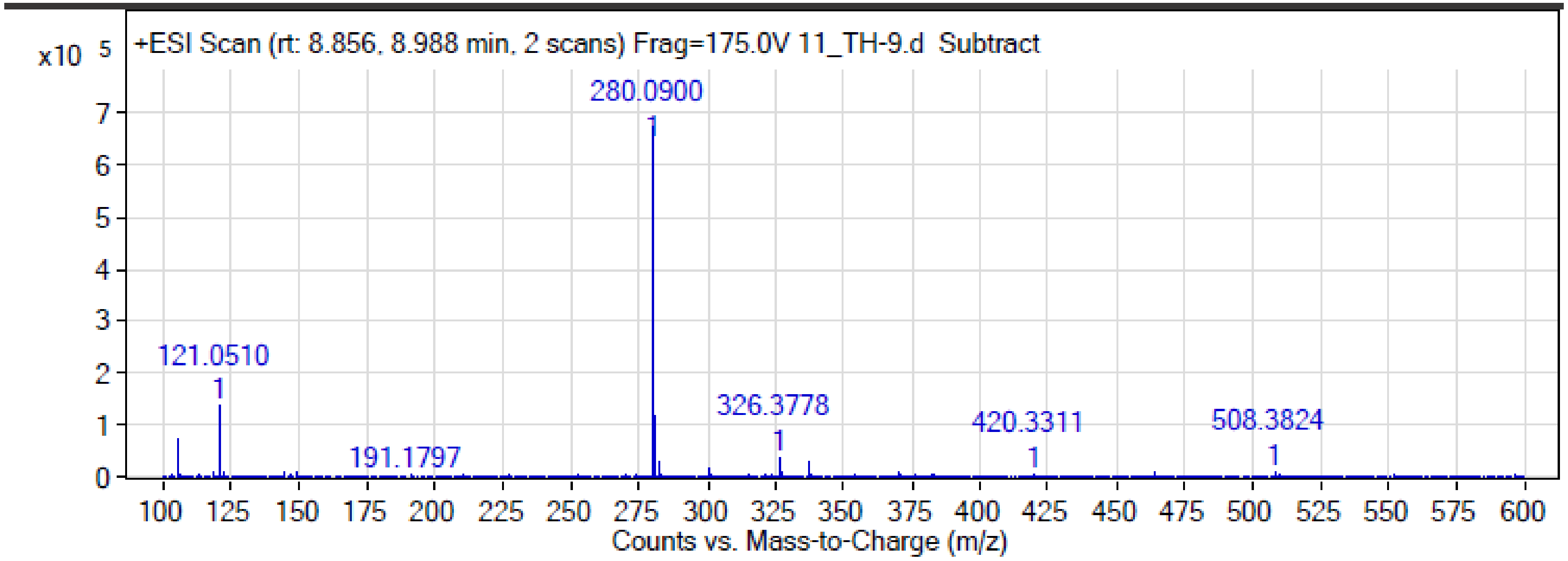

Formula Calculator Results

Formula	Best	Mass	Tgt Mass	Diff (ppm)	Ion Species	Score
C16 H13 N3 S	True	279.0828	279.083	0.76	C16 H14 N3 S	97.63

Figure S95. Mass spectrum and HRMS analysis of compound **16**.

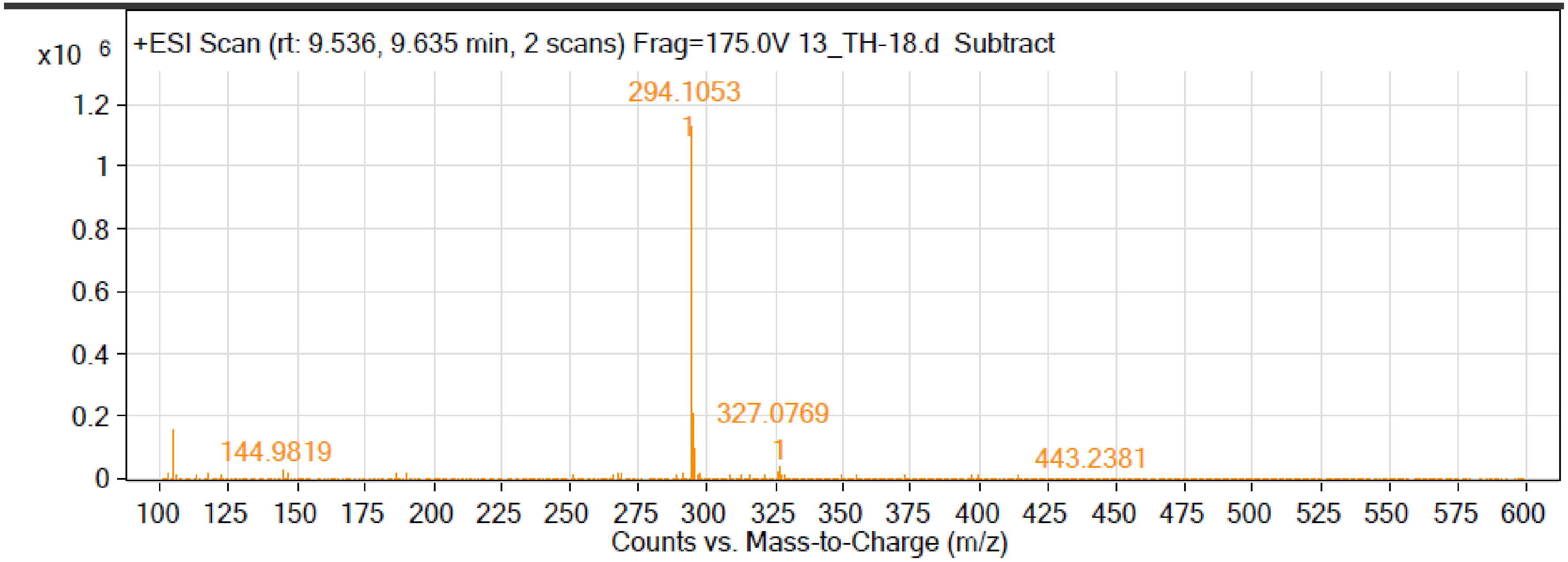

Peak List

Formula Calculator Results

Formula	Best	Mass	Tgt Mass	Diff (ppm)	Ion Species	Score
C17 H15 N3 S	True	293.0988	293.0987	-0.56	C17 H16 N3 S	87.86

Figure S96. Mass spectrum and HRMS analysis of compound **17**.

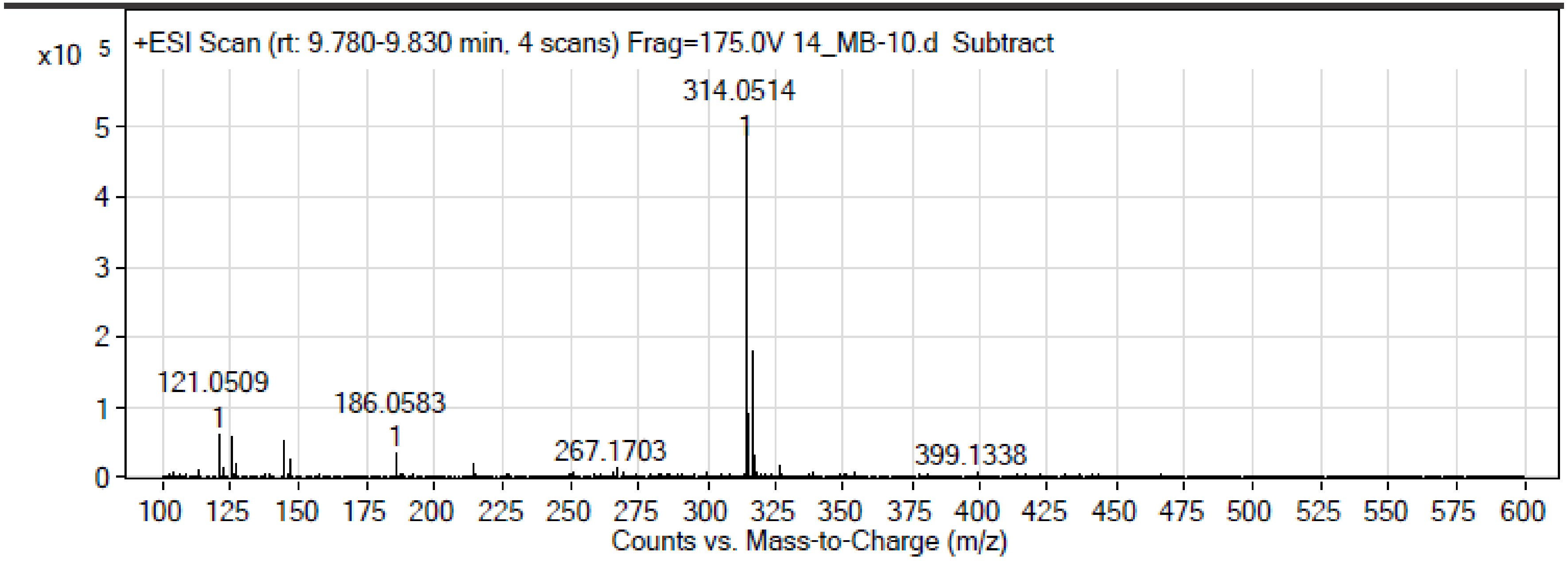

Formula Calculator Results

Formula	Best	Mass	Tgt Mass	Diff (ppm)	Ion Species	Score
C16 H12 Cl N3 S	True	313.0441	313.044	-0.21	C16 H13 Cl N3 S	99.38

Figure S97. Mass spectrum and HRMS analysis of compound **18**.

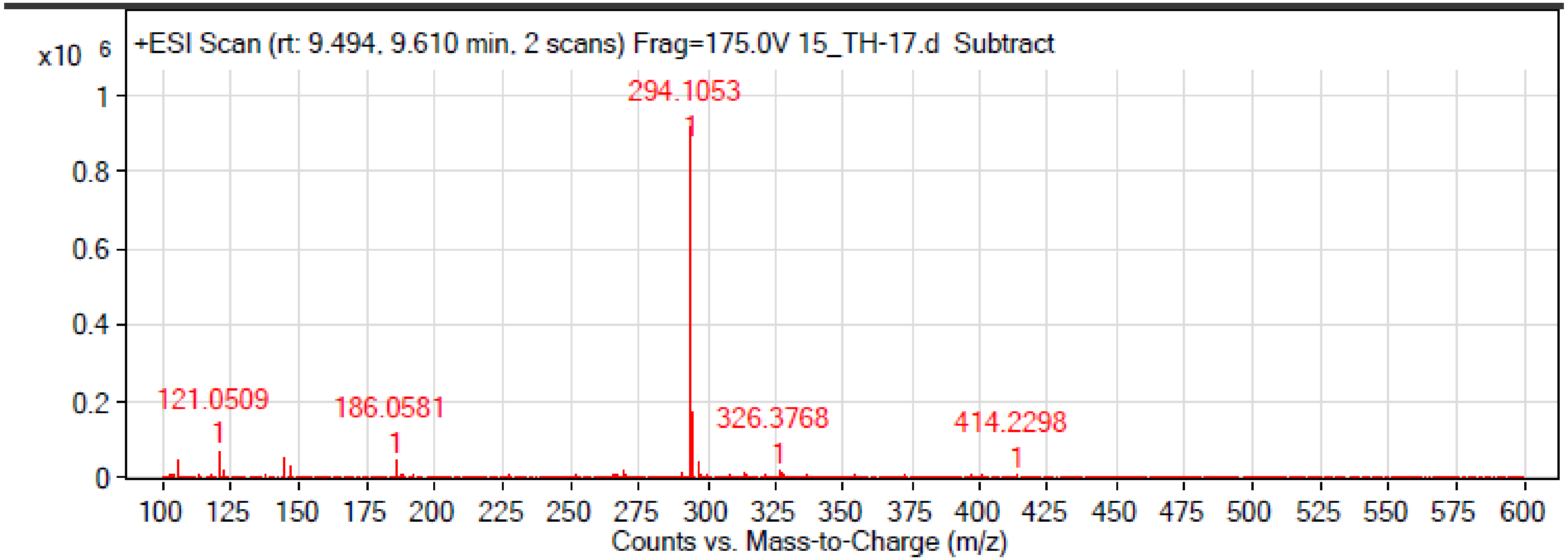

Formula Calculator Results

Formula	Best	Mass	Tgt Mass	Diff (ppm)	Ion Species	Score
C17 H15 N3 S	True	293.0981	293.0987	1.85	C17 H16 N3 S	96.41

Figure S98. Mass spectrum and HRMS analysis of compound **19**.

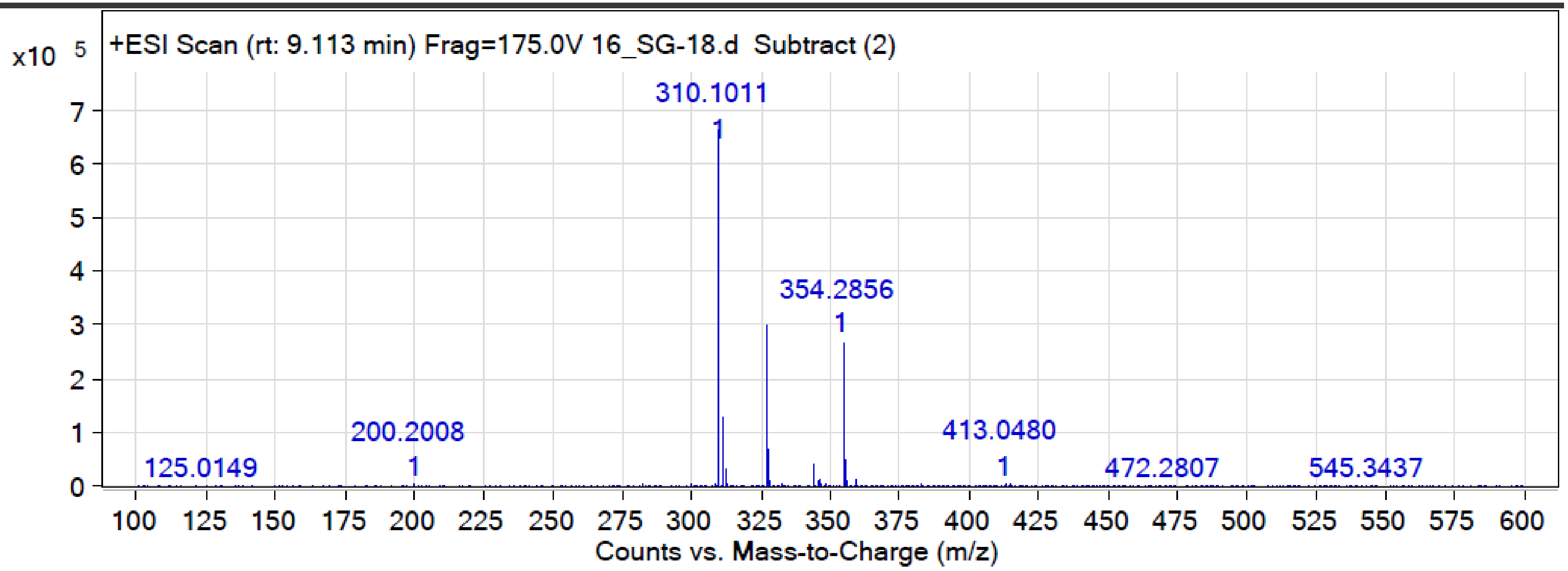

Formula Calculator Results

Formula	Best	Mass	Tgt Mass	Diff (ppm)	Ion Species	Score
C17 H15 N3 O S	True	309.0939	309.0936	-1.08	C17 H16 N3 O S	98

Figure S99. Mass spectrum and HRMS analysis of compound **20**.

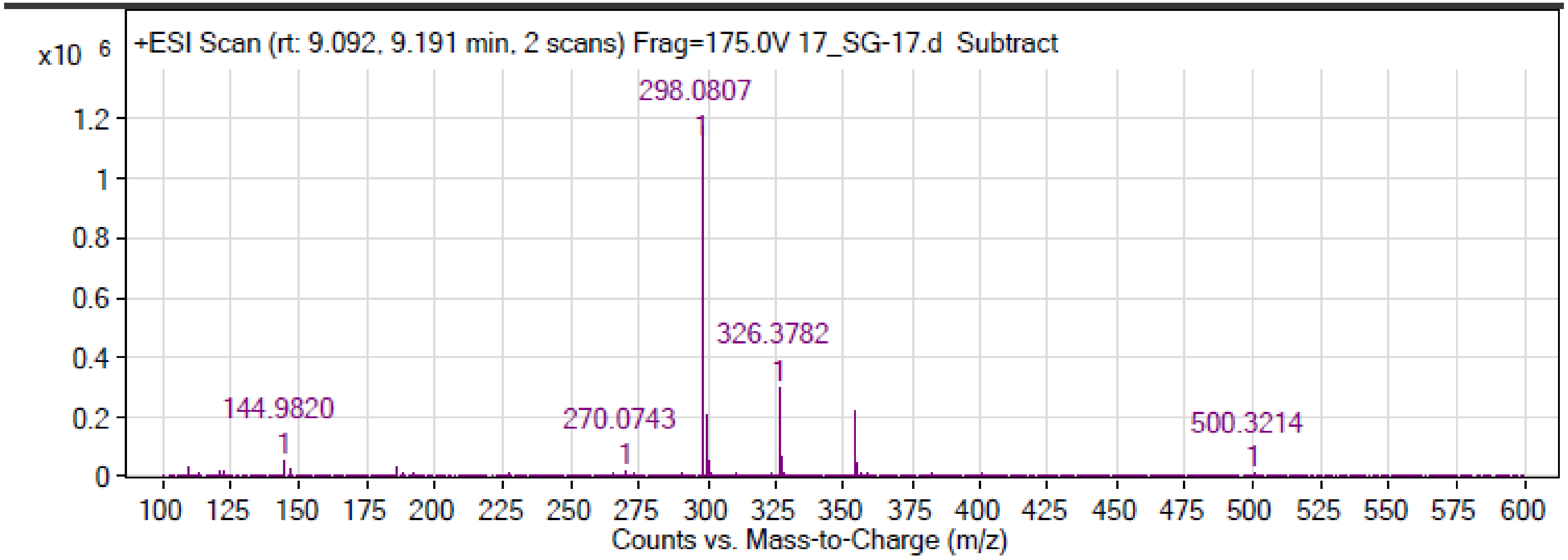

Formula Calculator Results

Formula	Best	Mass	Tgt Mass	Diff (ppm)	Ion Species	Score
C16 H12 F N3 S	True	297.0734	297.0736	0.53	C16 H13 F N3 S	97.94

Figure S100. Mass spectrum and HRMS analysis of compound **21**.

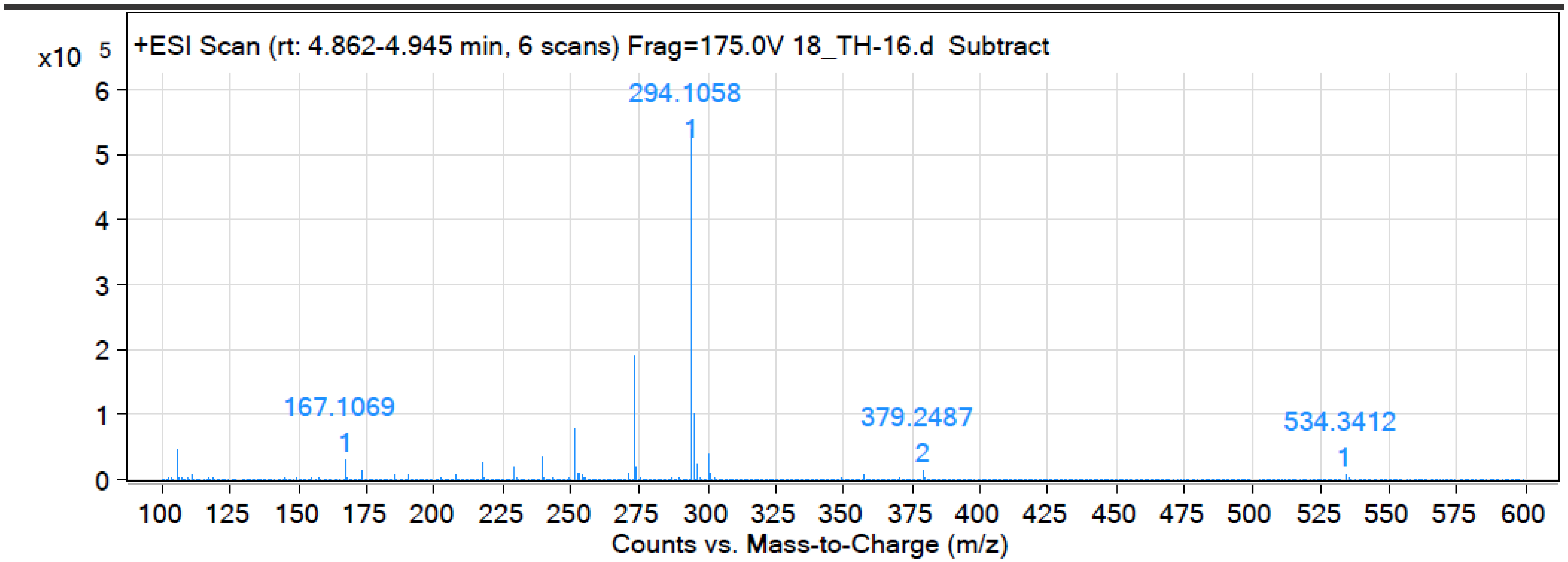

Formula Calculator Results

Formula	Best	Mass	Tgt Mass	Diff (ppm)	Ion Species	Score
C17 H15 N3 S	True	293.0985	293.0987	0.4	C17 H16 N3 S	98.1

Figure S101. Mass spectrum and HRMS analysis of compound **22**.

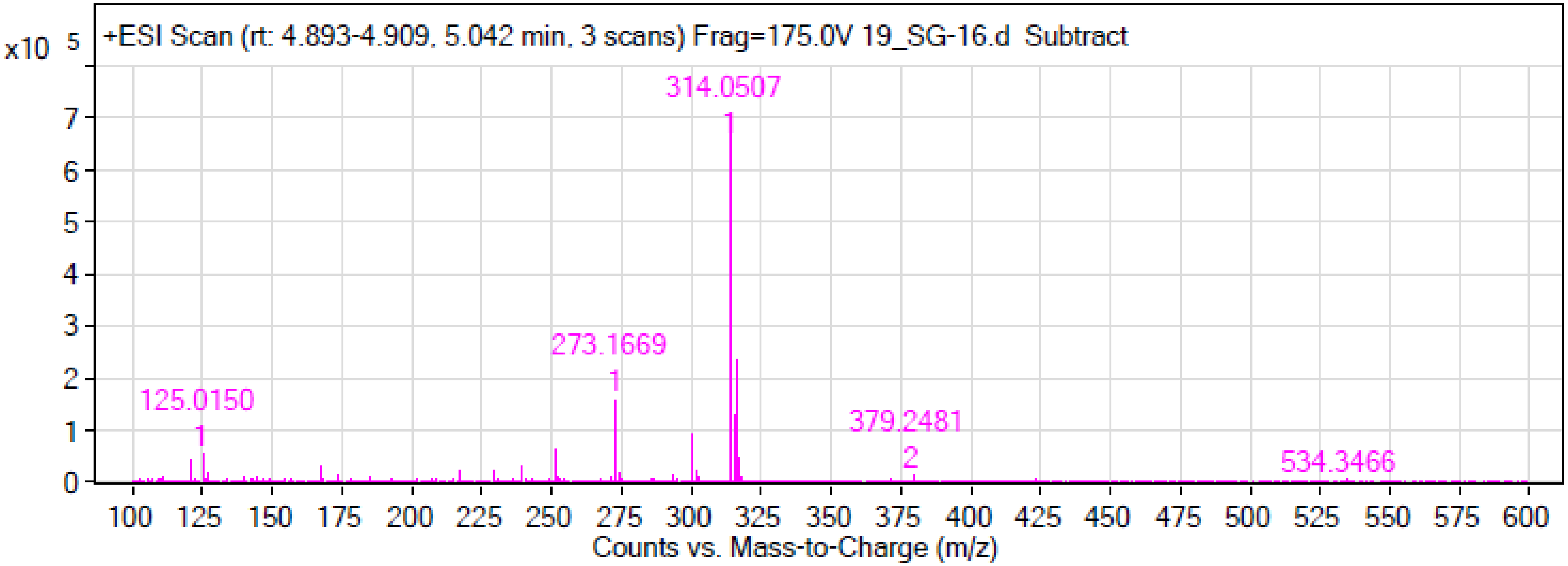

Formula Calculator Results

Formula	Best	Mass	Tgt Mass	Diff (ppm)	Ion Species	Score
C16 H12 Cl N3 S	True	313.0436	313.044	1.4	C16 H13 Cl N3 S	97.24

Figure S102. Mass spectrum and HRMS analysis of compound **23**.

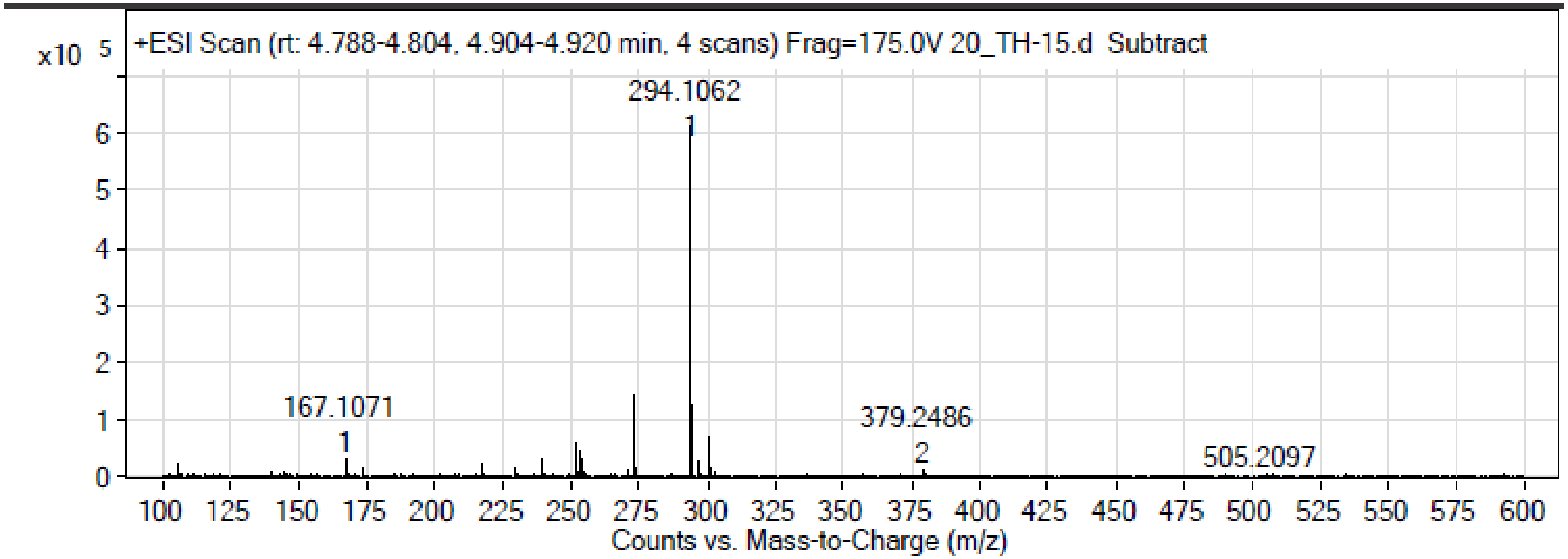

Peak List

Formula Calculator Results

Formula	Best	Mass	Tgt Mass	Diff (ppm)	Ion Species	Score
C17 H15 N3 S	True	293.0991	293.0987	-1.38	C17 H16 N3 S	97.07

Figure S103. Mass spectrum and HRMS analysis of compound **24**.

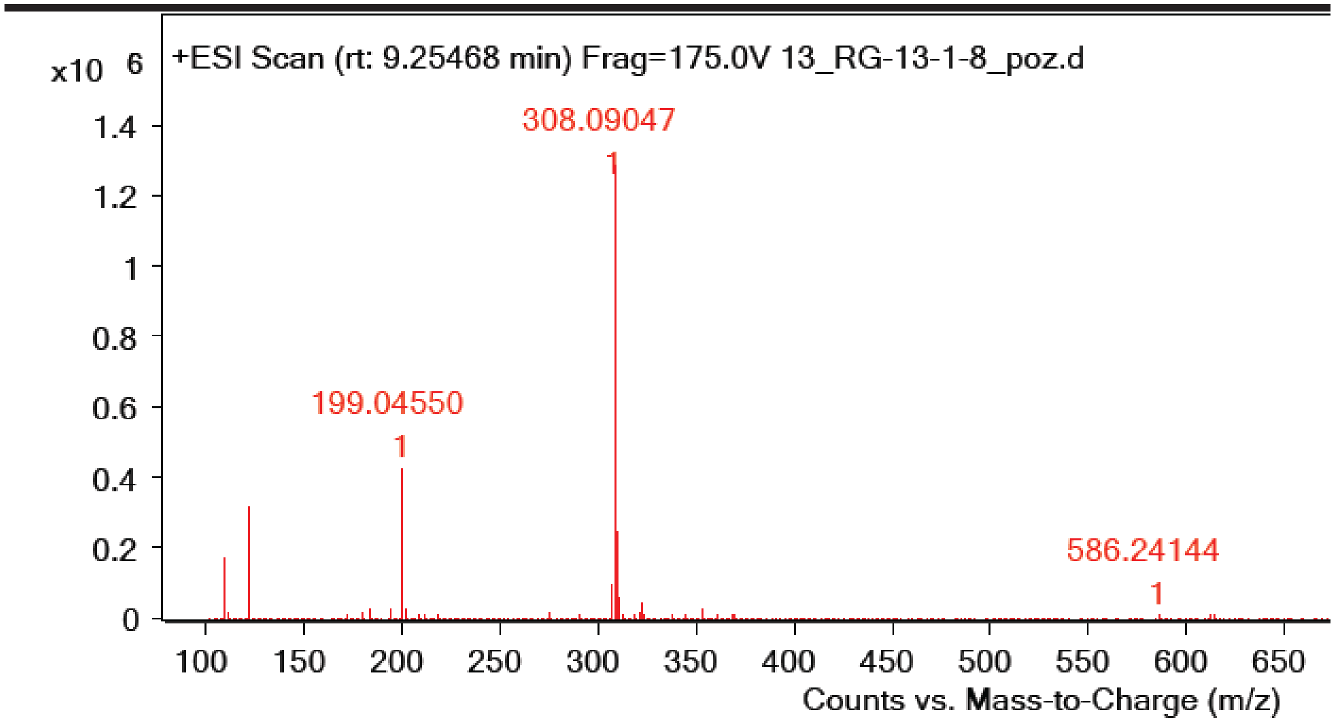

Figure S104. Mass spectrum of compound **25**.

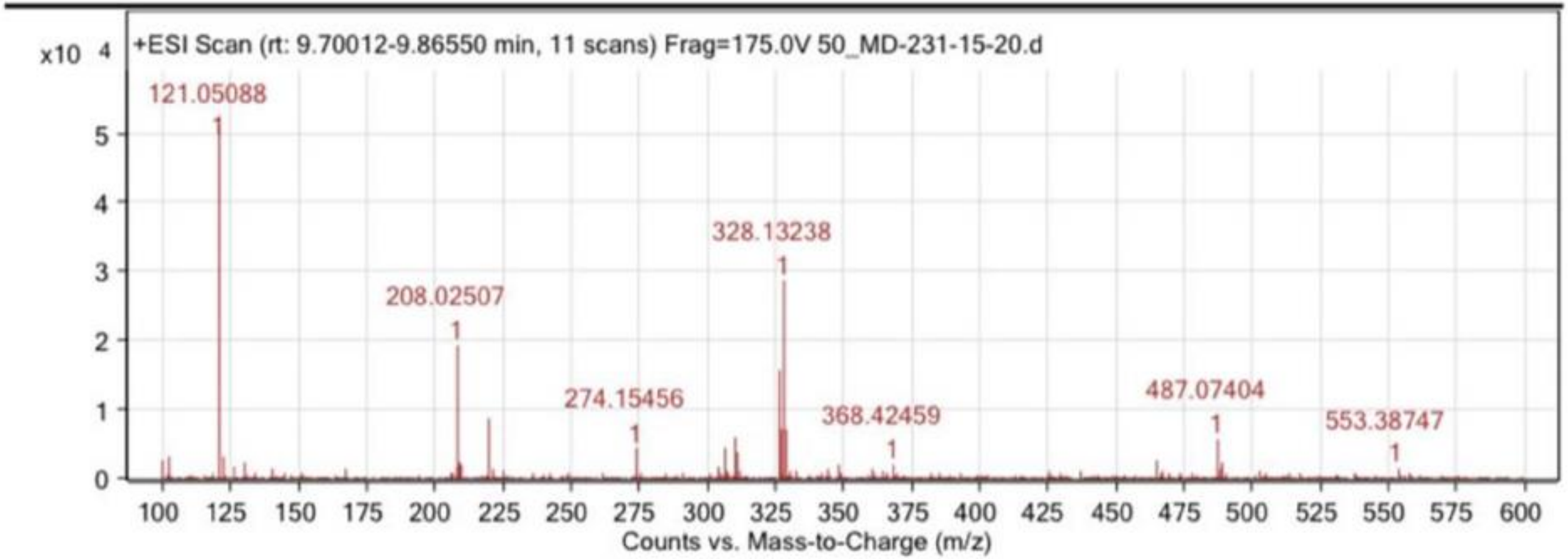

Figure S105. Mass spectrum of compound **26**.

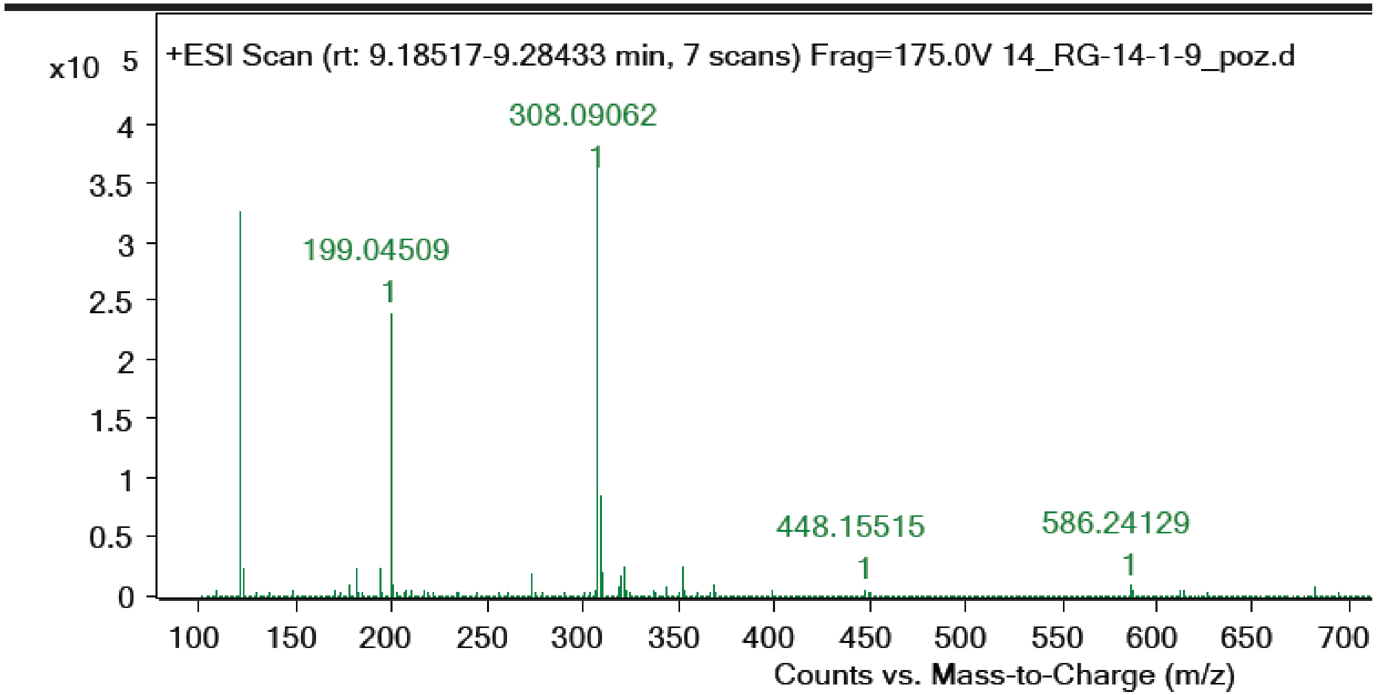

Figure S106. Mass spectrum of compound **27**.

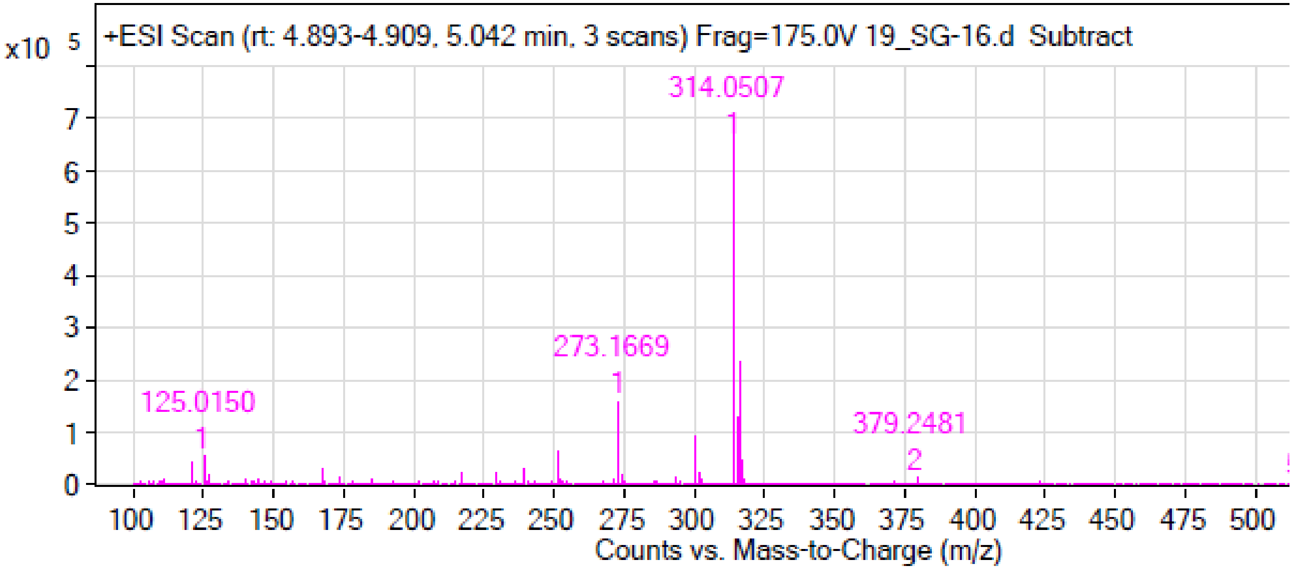

Formula Calculator Results

Formula	Best	Mass	Tgt Mass	Diff (ppm)	Ion Species	Score
C16 H12 Cl N3 S	True	313.0436	313.044	1.4	C16 H13 Cl N3 S	97.24

Figure S107. Mass spectrum and HRMS analysis of compound **30**.

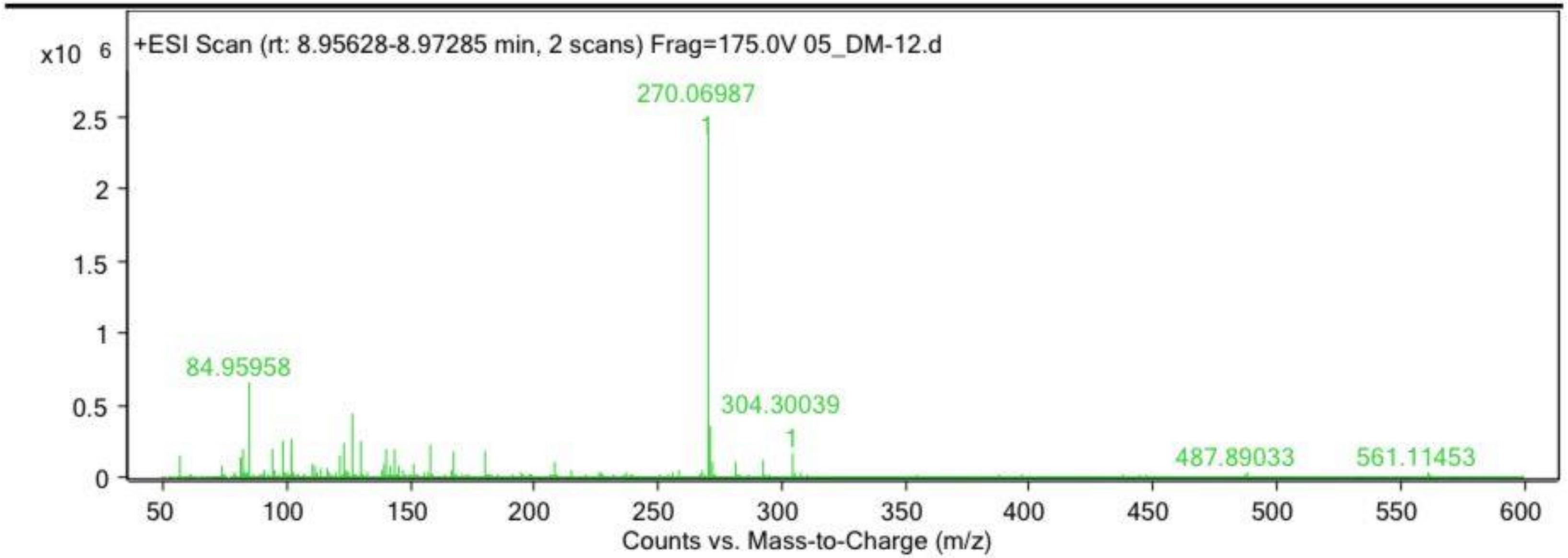

Formula Calculator Results

Formula	Best	Mass	Tgt Mass	Diff (ppm)	Ion Species	Score
C14 H11 N3 O S	True	269.06269	269.06228	-1.5	C14 H12 N3 O S	96.45

Figure S108. Mass spectrum and HRMS analysis of compound **31**.

3. Cartesian coordinates of docked ligands

Molecule 14 docked into the AChE

C	-13.573000	-45.130000	26.839000
H	-14.545000	-45.370000	26.376000
C	-13.199000	-43.793000	27.016000
C	-11.965000	-43.515000	27.610000
H	-11.654000	-42.467000	27.758000
C	-11.121000	-44.550000	28.018000
H	-10.154000	-44.308000	28.489000
C	-11.484000	-45.889000	27.838000
C	-12.726000	-46.159000	27.243000
H	-13.037000	-47.206000	27.092000
C	-10.562000	-47.013000	28.248000
H	-9.575000	-46.798000	28.721000
H	-10.387000	-47.661000	27.358000
H	-11.128000	-47.699000	28.921000
C	-14.133000	-42.659000	26.630000
H	-14.816000	-42.413000	27.476000
H	-13.570000	-41.700000	26.555000
N	-14.897000	-42.897000	25.412000
N	-14.227000	-43.254000	24.271000
N	-15.079000	-43.426000	23.314000
C	-16.342000	-43.187000	23.805000
C	-17.590000	-43.269000	23.149000
H	-17.652000	-43.545000	22.083000
C	-16.239000	-42.837000	25.165000
C	-18.728000	-42.995000	23.877000
H	-19.720000	-43.055000	23.399000
C	-18.616000	-42.635000	25.244000
S	-19.955000	-42.248000	26.307000
C	-17.387000	-42.534000	25.937000
C	-18.866000	-41.953000	27.638000
H	-19.202000	-41.648000	28.643000
C	-17.559000	-42.138000	27.307000
H	-16.723000	-41.999000	28.013000

Molecule 15 docked into the AChE

S	-19.956000	-42.346000	26.333000

C	-18.619000	-42.675000	25.250000
C	-17.385000	-42.529000	25.925000
C	-17.553000	-42.144000	27.300000
H	-16.713000	-41.974000	27.995000
C	-18.860000	-42.013000	27.650000
H	-19.193000	-41.726000	28.661000
C	-16.237000	-42.779000	25.134000
C	-16.345000	-43.129000	23.776000
C	-17.598000	-43.259000	23.137000
H	-17.665000	-43.534000	22.071000
C	-18.735000	-43.033000	23.883000
H	-19.731000	-43.130000	23.418000
N	-15.080000	-43.312000	23.265000
N	-14.221000	-43.107000	24.208000
N	-14.889000	-42.784000	25.363000
C	-14.117000	-42.510000	26.565000
H	-14.792000	-42.227000	27.406000
H	-13.545000	-41.560000	26.453000
C	-13.192000	-43.640000	26.984000
C	-11.962000	-43.354000	27.586000
H	-11.653000	-42.303000	27.716000
C	-11.120000	-44.376000	28.024000
H	-10.154000	-44.140000	28.500000
C	-11.518000	-45.700000	27.851000
Cl	-10.463000	-46.998000	28.391000
C	-12.740000	-46.010000	27.254000
H	-13.044000	-47.062000	27.122000
C	-13.572000	-44.978000	26.826000
H	-14.541000	-45.218000	26.357000

Cation 22 docked into the AChE

N	-14.696000	-44.324000	26.860000
C	-14.020000	-44.748000	27.983000
C	-14.775000	-44.261000	29.064000
N	-15.826000	-43.595000	28.483000
N	-15.775000	-43.635000	27.166000
C	-14.425000	-44.478000	30.413000
H	-15.036000	-44.085000	31.243000
C	-13.275000	-45.209000	30.638000
H	-12.950000	-45.410000	31.673000
C	-12.509000	-45.704000	29.554000

C	-12.839000	-45.501000	28.191000
S	-11.044000	-46.638000	29.699000
C	-10.894000	-46.742000	27.973000
H	-10.068000	-47.270000	27.468000
C	-11.897000	-46.107000	27.301000
H	-11.973000	-46.066000	26.201000
C	-16.928000	-42.889000	29.138000
H	-16.971000	-42.855000	30.252000
H	-17.892000	-43.300000	28.756000
H	-16.964000	-41.845000	28.748000
C	-14.325000	-44.569000	25.429000
H	-14.613000	-45.601000	25.121000
H	-13.218000	-44.642000	25.318000
C	-14.886000	-43.556000	24.472000
C	-14.344000	-42.267000	24.403000
H	-13.490000	-41.995000	25.046000
C	-14.878000	-41.326000	23.526000
H	-14.437000	-40.316000	23.480000
C	-15.967000	-41.641000	22.700000
C	-16.502000	-42.935000	22.777000
H	-17.355000	-43.208000	22.134000
C	-16.567000	-40.607000	21.780000
H	-16.142000	-39.578000	21.719000
H	-17.653000	-40.520000	22.018000
H	-16.593000	-41.033000	20.750000
C	-15.974000	-43.881000	23.652000
H	-16.415000	-44.891000	23.700000

Cation 23 docked into the AChE

N	-14.622000	-44.354000	26.920000
C	-13.945000	-44.767000	28.046000
C	-14.699000	-44.267000	29.122000
N	-15.750000	-43.606000	28.535000
N	-15.700000	-43.660000	27.218000
C	-14.348000	-44.469000	30.474000
H	-14.958000	-44.066000	31.300000
C	-13.200000	-45.199000	30.706000
H	-12.875000	-45.389000	31.743000
C	-12.434000	-45.707000	29.628000

C	-12.764000	-45.519000	28.262000
S	-10.970000	-46.640000	29.783000
C	-10.821000	-46.764000	28.058000
H	-9.996000	-47.300000	27.559000
C	-11.823000	-46.135000	27.378000
H	-11.898000	-46.105000	26.278000
C	-16.851000	-42.892000	29.183000
H	-16.893000	-42.846000	30.296000
H	-17.815000	-43.306000	28.806000
H	-16.887000	-41.852000	28.782000
C	-14.253000	-44.613000	25.492000
H	-14.534000	-45.650000	25.196000
H	-13.146000	-44.680000	25.378000
C	-14.825000	-43.613000	24.527000
C	-14.346000	-42.297000	24.504000
H	-13.537000	-41.999000	25.192000
C	-14.880000	-41.362000	23.622000
H	-14.497000	-40.328000	23.605000
C	-15.907000	-41.750000	22.758000
C	-16.404000	-43.055000	22.771000
H	-17.217000	-43.350000	22.087000
Cl	-16.585000	-40.583000	21.650000
C	-15.860000	-43.980000	23.659000
H	-16.249000	-45.012000	23.678000

Molecule 14 docked into the BChE

C	-18.671000	-14.597000	44.662000
H	-19.250000	-15.073000	43.853000
C	-18.472000	-13.212000	44.651000
C	-17.741000	-12.631000	45.691000
H	-17.575000	-11.541000	45.699000
C	-17.218000	-13.418000	46.720000
H	-16.647000	-12.939000	47.534000
C	-17.407000	-14.804000	46.734000
C	-18.144000	-15.378000	45.687000
H	-18.309000	-16.469000	45.676000
C	-16.817000	-15.664000	47.827000
H	-16.968000	-16.769000	47.838000
H	-17.161000	-15.259000	48.807000
H	-15.721000	-15.465000	47.868000

C	-19.085000	-12.339000	43.569000
H	-19.983000	-11.807000	43.961000
H	-19.564000	-12.970000	42.785000
N	-18.158000	-11.392000	42.963000
N	-16.820000	-11.689000	42.957000
N	-16.175000	-10.733000	42.371000
C	-17.080000	-9.776000	41.973000
C	-16.859000	-8.566000	41.278000
H	-15.841000	-8.259000	40.984000
C	-18.371000	-10.187000	42.356000
C	-17.950000	-7.779000	40.977000
H	-17.815000	-6.830000	40.431000
C	-19.246000	-8.195000	41.373000
S	-20.732000	-7.314000	41.079000
C	-19.511000	-9.394000	42.075000
C	-21.669000	-8.540000	41.894000
H	-22.764000	-8.490000	42.015000
C	-20.907000	-9.565000	42.365000
H	-21.317000	-10.432000	42.910000

Molecule 15 docked into the BChE

S	-20.706000	-7.362000	41.075000
C	-19.226000	-8.238000	41.411000
C	-19.513000	-9.478000	42.029000
C	-20.922000	-9.682000	42.224000
H	-21.351000	-10.583000	42.695000
C	-21.669000	-8.643000	41.764000
H	-22.770000	-8.613000	41.821000
C	-18.378000	-10.267000	42.339000
C	-17.075000	-9.820000	42.056000
C	-16.832000	-8.571000	41.442000
H	-15.803000	-8.236000	41.227000
C	-17.916000	-7.783000	41.119000
H	-17.765000	-6.803000	40.636000
N	-16.180000	-10.785000	42.460000
N	-16.840000	-11.778000	42.955000
N	-18.183000	-11.501000	42.894000
C	-19.125000	-12.487000	43.399000
H	-20.097000	-12.003000	43.652000
H	-19.458000	-13.164000	42.578000
C	-18.611000	-13.295000	44.579000

C	-17.929000	-12.659000	45.621000
H	-17.733000	-11.575000	45.559000
C	-17.492000	-13.373000	46.738000
H	-16.957000	-12.862000	47.556000
C	-17.742000	-14.742000	46.803000
Cl	-17.191000	-15.656000	48.200000
C	-18.421000	-15.399000	45.776000
H	-18.613000	-16.483000	45.839000
C	-18.854000	-14.671000	44.670000
H	-19.394000	-15.184000	43.857000

Cation 22 docked into BChE

N	-18.171000	-11.585000	42.584000
C	-18.879000	-10.445000	42.275000
C	-17.902000	-9.496000	41.926000
N	-16.708000	-10.161000	42.062000
N	-16.873000	-11.409000	42.456000
C	-18.208000	-8.176000	41.537000
H	-17.421000	-7.451000	41.268000
C	-19.547000	-7.840000	41.511000
H	-19.848000	-6.821000	41.216000
C	-20.538000	-8.789000	41.859000
C	-20.257000	-10.121000	42.254000
S	-22.257000	-8.495000	41.867000
C	-22.571000	-10.122000	42.387000
H	-23.586000	-10.514000	42.567000
C	-21.441000	-10.868000	42.551000
H	-21.440000	-11.923000	42.874000
C	-15.354000	-9.655000	41.828000
H	-15.215000	-8.600000	41.495000
H	-14.842000	-10.329000	41.102000
H	-14.747000	-9.822000	42.748000
C	-18.724000	-12.907000	43.023000
H	-19.824000	-12.949000	42.848000
H	-18.393000	-13.719000	42.334000
C	-18.402000	-13.260000	44.448000
C	-18.693000	-14.535000	44.947000
H	-19.150000	-15.291000	44.287000

C	-18.408000	-14.853000	46.272000
H	-18.639000	-15.864000	46.649000
C	-17.832000	-13.909000	47.136000
C	-17.545000	-12.635000	46.625000
H	-17.088000	-11.877000	47.283000
C	-17.562000	-14.245000	48.582000
H	-17.104000	-13.495000	49.268000
H	-16.939000	-15.169000	48.613000
H	-18.515000	-14.598000	49.040000
C	-17.827000	-12.310000	45.301000
H	-17.597000	-11.300000	44.922000

Cation 23 docked into BChE

N	-18.070000	-11.470000	41.949000
C	-18.889000	-10.366000	41.868000
C	-18.045000	-9.322000	41.449000
N	-16.807000	-9.905000	41.323000
N	-16.827000	-11.188000	41.622000
C	-18.492000	-8.001000	41.239000
H	-17.808000	-7.200000	40.911000
C	-19.833000	-7.765000	41.467000
H	-20.241000	-6.751000	41.319000
C	-20.692000	-8.810000	41.889000
C	-20.268000	-10.146000	42.107000
S	-22.396000	-8.647000	42.211000
C	-22.519000	-10.329000	42.624000
H	-23.463000	-10.809000	42.933000
C	-21.335000	-11.000000	42.529000
H	-21.215000	-12.074000	42.751000
C	-15.548000	-9.281000	40.913000
H	-14.544000	-9.754000	40.811000
H	-15.404000	-8.415000	41.601000
H	-15.757000	-8.803000	39.928000
C	-18.456000	-12.860000	42.351000
H	-17.926000	-13.612000	41.721000
H	-19.509000	-13.076000	42.055000
C	-18.246000	-13.149000	43.811000
C	-17.637000	-12.199000	44.641000
H	-17.320000	-11.230000	44.220000
C	-17.427000	-12.465000	45.991000
H	-16.950000	-11.712000	46.641000
C	-17.828000	-13.698000	46.512000

C	-18.429000	-14.662000	45.700000
H	-18.738000	-15.633000	46.122000
Cl	-17.566000	-14.042000	48.203000
C	-18.633000	-14.382000	44.351000
H	-19.104000	-15.139000	43.701000

Coordinates of AChE (4ey7.pdb) used for the docking study.

The search region of the enzyme was determined by setting the grid box around the center of the active site, consisting of 5 subdomains: peripheral anionic site (Tyr72, Asp74, Tyr124, Trp286, Tyr341), anionic site (Trp86, Glu202, Phe338), oxyanion hole (Gly121, Gly122, Ala204), acyl pocket (Phe295, Phe297) and esteratic site (Ser203, Glu334, His447).

```
ATOM      1  N   GLU A   4       2.514 -35.401  69.696  1.00 63.34           N
ATOM      2  CA  GLU A   4       2.230 -35.193  68.280  1.00 69.93           C
ATOM      3  C   GLU A   4       3.436 -35.566  67.408  1.00 69.11           C
ATOM      4  O   GLU A   4       4.572 -35.606  67.889  1.00 68.26           O
ATOM      5  CB  GLU A   4       1.808 -33.745  68.027  1.00 75.30           C
ATOM      6  CG  GLU A   4       1.007 -33.544  66.749  1.00 72.83           C
ATOM      7  CD  GLU A   4       0.729 -32.081  66.462  1.00 85.00           C
ATOM      8  OE1 GLU A   4       1.240 -31.222  67.218  1.00 82.36           O
ATOM      9  OE2 GLU A   4       0.004 -31.791  65.480  1.00 82.44           O
ATOM     10  N   ASP A   5       3.181 -35.833  66.128  1.00 52.26           N
ATOM     11  CA  ASP A   5       4.201 -36.371  65.228  1.00 52.44           C
ATOM     12  C   ASP A   5       4.816 -35.290  64.335  1.00 54.45           C
ATOM     13  O   ASP A   5       4.158 -34.780  63.425  1.00 49.84           O
ATOM     14  CB  ASP A   5       3.594 -37.477  64.366  1.00 39.43           C
ATOM     15  CG  ASP A   5       4.644 -38.385  63.750  1.00 48.34           C
ATOM     16  OD1 ASP A   5       4.289 -39.534  63.394  1.00 48.09           O
ATOM     17  OD2 ASP A   5       5.819 -37.960  63.629  1.00 39.55           O
ATOM     18  N   ALA A   6       6.083 -34.963  64.591  1.00 49.41           N
ATOM     19  CA  ALA A   6       6.767 -33.874  63.893  1.00 47.25           C
ATOM     20  C   ALA A   6       6.999 -34.189  62.421  1.00 50.52           C
ATOM     21  O   ALA A   6       7.210 -33.285  61.609  1.00 49.69           O
ATOM     22  CB  ALA A   6       8.095 -33.552  64.569  1.00 39.15           C
ATOM     23  N   GLU A   7       6.976 -35.472  62.081  1.00 44.06           N
ATOM     24  CA  GLU A   7       7.172 -35.882  60.701  1.00 44.76           C
ATOM     25  C   GLU A   7       6.010 -35.441  59.803  1.00 43.05           C
ATOM     26  O   GLU A   7       6.171 -35.278  58.594  1.00 37.71           O
ATOM     27  CB  GLU A   7       7.350 -37.396  60.629  1.00 47.62           C
ATOM     28  CG  GLU A   7       8.570 -37.904  61.377  1.00 46.10           C
```

```
ATOM     29  CD  GLU A   7       9.875 -37.419  60.768  1.00 51.67           C
ATOM     30  OE1 GLU A   7       9.880 -37.016  59.580  1.00 46.41           O
ATOM     31  OE2 GLU A   7      10.901 -37.451  61.480  1.00 47.54           O
ATOM     32  N   LEU A   8       4.848 -35.237  60.411  1.00 37.36           N
ATOM     33  CA  LEU A   8       3.632 -34.935  59.678  1.00 37.81           C
ATOM     34  C   LEU A   8       3.298 -33.450  59.708  1.00 37.82           C
ATOM     35  O   LEU A   8       2.201 -33.047  59.321  1.00 34.33           O
ATOM     36  CB  LEU A   8       2.471 -35.737  60.268  1.00 37.14           C
ATOM     37  CG  LEU A   8       2.729 -37.240  60.358  1.00 40.81           C
ATOM     38  CD1 LEU A   8       1.502 -37.949  60.910  1.00 36.15           C
ATOM     39  CD2 LEU A   8       3.125 -37.797  58.983  1.00 31.05           C
ATOM     40  N   LEU A   9       4.237 -32.644  60.192  1.00 37.67           N
ATOM     41  CA  LEU A   9       4.085 -31.195  60.163  1.00 33.55           C
ATOM     42  C   LEU A   9       5.075 -30.632  59.166  1.00 34.56           C
ATOM     43  O   LEU A   9       6.267 -30.920  59.239  1.00 36.14           O
ATOM     44  CB  LEU A   9       4.334 -30.587  61.543  1.00 38.69           C
ATOM     45  CG  LEU A   9       3.406 -31.057  62.662  1.00 44.18           C
ATOM     46  CD1 LEU A   9       3.760 -30.381  63.980  1.00 36.23           C
ATOM     47  CD2 LEU A   9       1.948 -30.806  62.293  1.00 39.51           C
ATOM     48  N   VAL A  10       4.569 -29.852  58.215  1.00 32.80           N
ATOM     49  CA  VAL A  10       5.402 -29.249  57.181  1.00 28.75           C
ATOM     50  C   VAL A  10       4.956 -27.810  56.965  1.00 28.14           C
ATOM     51  O   VAL A  10       3.764 -27.495  57.022  1.00 25.42           O
ATOM     52  CB  VAL A  10       5.265 -29.985  55.813  1.00 33.53           C
ATOM     53  CG1 VAL A  10       6.195 -29.378  54.770  1.00 33.30           C
ATOM     54  CG2 VAL A  10       5.555 -31.447  55.956  1.00 37.85           C
ATOM     55  N   THR A  11       5.913 -26.936  56.701  1.00 32.95           N
ATOM     56  CA  THR A  11       5.588 -25.570  56.351  1.00 33.65           C
ATOM     57  C   THR A  11       5.986 -25.337  54.909  1.00 29.68           C
ATOM     58  O   THR A  11       7.135 -25.543  54.530  1.00 32.40           O
ATOM     59  CB  THR A  11       6.300 -24.564  57.280  1.00 34.32           C
ATOM     60  OG1 THR A  11       5.942 -24.844  58.642  1.00 35.79           O
ATOM     61  CG2 THR A  11       5.894 -23.136  56.933  1.00 27.62           C
ATOM     62  N   VAL A  12       5.016 -24.941  54.098  1.00 26.59           N
ATOM     63  CA  VAL A  12       5.291 -24.567  52.720  1.00 33.50           C
ATOM     64  C   VAL A  12       5.092 -23.064  52.633  1.00 30.59           C
ATOM     65  O   VAL A  12       4.693 -22.436  53.611  1.00 37.88           O
ATOM     66  CB  VAL A  12       4.378 -25.323  51.718  1.00 29.54           C
ATOM     67  CG1 VAL A  12       4.746 -26.792  51.688  1.00 28.07           C
ATOM     68  CG2 VAL A  12       2.895 -25.152  52.078  1.00 22.96           C
ATOM     69  N   ARG A  13       5.365 -22.487  51.476  1.00 29.75           N
ATOM     70  CA  ARG A  13       5.316 -21.039  51.322  1.00 31.99           C
ATOM     71  C   ARG A  13       3.998 -20.406  51.748  1.00 30.74           C
ATOM     72  O   ARG A  13       3.981 -19.295  52.263  1.00 39.48           O
```

```
ATOM     73  CB  ARG A  13       5.663 -20.643  49.883  1.00 27.11           C
ATOM     74  CG  ARG A  13       7.114 -20.909  49.557  1.00 34.49           C
ATOM     75  CD  ARG A  13       7.582 -20.190  48.308  1.00 46.28           C
ATOM     76  NE  ARG A  13       8.927 -20.623  47.931  1.00 55.78           N
ATOM     77  CZ  ARG A  13       9.582 -20.193  46.857  1.00 55.33           C
ATOM     78  NH1 ARG A  13       9.017 -19.309  46.046  1.00 55.82           N
ATOM     79  NH2 ARG A  13      10.802 -20.650  46.593  1.00 51.58           N
ATOM     80  N   GLY A  14       2.896 -21.112  51.541  1.00 33.97           N
ATOM     81  CA  GLY A  14       1.589 -20.553  51.824  1.00 28.80           C
ATOM     82  C   GLY A  14       1.136 -20.752  53.255  1.00 31.36           C
ATOM     83  O   GLY A  14       0.104 -20.219  53.662  1.00 35.36           O
ATOM     84  N   GLY A  15       1.900 -21.517  54.028  1.00 34.77           N
ATOM     85  CA  GLY A  15       1.525 -21.792  55.403  1.00 32.18           C
ATOM     86  C   GLY A  15       1.807 -23.214  55.846  1.00 29.53           C
ATOM     87  O   GLY A  15       2.558 -23.944  55.201  1.00 35.95           O
ATOM     88  N   ARG A  16       1.193 -23.602  56.957  1.00 34.28           N
ATOM     89  CA  ARG A  16       1.494 -24.870  57.609  1.00 36.24           C
ATOM     90  C   ARG A  16       0.532 -25.995  57.210  1.00 34.39           C
ATOM     91  O   ARG A  16      -0.656 -25.754  56.976  1.00 31.78           O
ATOM     92  CB  ARG A  16       1.481 -24.682  59.131  1.00 37.88           C
ATOM     93  CG  ARG A  16       2.704 -23.935  59.691  1.00 44.59           C
ATOM     94  CD  ARG A  16       2.591 -23.720  61.209  1.00 36.64           C
ATOM     95  NE  ARG A  16       1.441 -22.888  61.552  1.00 46.69           N
ATOM     96  CZ  ARG A  16       0.685 -23.055  62.635  1.00 52.46           C
ATOM     97  NH1 ARG A  16       0.949 -24.035  63.489  1.00 49.27           N
ATOM     98  NH2 ARG A  16      -0.344 -22.243  62.862  1.00 47.23           N
ATOM     99  N   LEU A  17       1.058 -27.222  57.162  1.00 28.61           N
ATOM    100  CA  LEU A  17       0.282 -28.410  56.796  1.00 32.47           C
ATOM    101  C   LEU A  17       0.421 -29.511  57.836  1.00 29.60           C
ATOM    102  O   LEU A  17       1.482 -29.678  58.442  1.00 28.51           O
ATOM    103  CB  LEU A  17       0.777 -28.993  55.462  1.00 29.75           C
ATOM    104  CG  LEU A  17       0.773 -28.112  54.223  1.00 27.85           C
ATOM    105  CD1 LEU A  17       1.429 -28.843  53.050  1.00 26.86           C
ATOM    106  CD2 LEU A  17      -0.655 -27.726  53.894  1.00 31.11           C
ATOM    107  N   ARG A  18      -0.645 -30.287  58.000  1.00 29.63           N
ATOM    108  CA  ARG A  18      -0.599 -31.514  58.787  1.00 35.47           C
ATOM    109  C   ARG A  18      -0.925 -32.725  57.902  1.00 39.58           C
ATOM    110  O   ARG A  18      -1.995 -32.798  57.288  1.00 30.43           O
ATOM    111  CB  ARG A  18      -1.578 -31.445  59.961  1.00 33.61           C
ATOM    112  CG  ARG A  18      -1.723 -32.753  60.736  1.00 43.66           C
ATOM    113  CD  ARG A  18      -2.697 -32.581  61.903  1.00 58.88           C
ATOM    114  NE  ARG A  18      -3.704 -31.551  61.620  1.00 65.15           N
ATOM    115  CZ  ARG A  18      -4.953 -31.796  61.222  1.00 61.20           C
ATOM    116  NH1 ARG A  18      -5.380 -33.048  61.071  1.00 56.33           N
```

ATOM	117	NH2	ARG	A	18	-5.785	-30.788	60.987	1.00	52.28	N
ATOM	118	N	GLY	A	19	0.001	-33.676	57.848	1.00	32.94	N
ATOM	119	CA	GLY	A	19	-0.203	-34.875	57.065	1.00	34.62	C
ATOM	120	C	GLY	A	19	-0.705	-36.052	57.870	1.00	37.12	C
ATOM	121	O	GLY	A	19	-1.248	-35.898	58.965	1.00	39.77	O
ATOM	122	N	ILE	A	20	-0.520	-37.242	57.312	1.00	33.27	N
ATOM	123	CA	ILE	A	20	-0.988	-38.460	57.942	1.00	27.99	C
ATOM	124	C	ILE	A	20	-0.004	-39.577	57.646	1.00	31.46	C
ATOM	125	O	ILE	A	20	0.595	-39.617	56.572	1.00	33.31	O
ATOM	126	CB	ILE	A	20	-2.413	-38.832	57.476	1.00	33.65	C
ATOM	127	CG1	ILE	A	20	-2.956	-40.016	58.282	1.00	33.66	C
ATOM	128	CG2	ILE	A	20	-2.453	-39.123	55.957	1.00	30.57	C
ATOM	129	CD1	ILE	A	20	-4.390	-40.384	57.929	1.00	29.74	C
ATOM	130	N	ARG	A	21	0.186	-40.461	58.621	1.00	34.29	N
ATOM	131	CA	ARG	A	21	1.048	-41.624	58.462	1.00	34.59	C
ATOM	132	C	ARG	A	21	0.220	-42.748	57.857	1.00	34.51	C
ATOM	133	O	ARG	A	21	-0.907	-42.999	58.289	1.00	29.00	O
ATOM	134	CB	ARG	A	21	1.621	-42.053	59.820	1.00	32.91	C
ATOM	135	CG	ARG	A	21	2.887	-42.897	59.731	1.00	35.53	C
ATOM	136	CD	ARG	A	21	3.487	-43.168	61.131	1.00	45.17	C
ATOM	137	NE	ARG	A	21	2.480	-43.667	62.065	1.00	42.00	N
ATOM	138	CZ	ARG	A	21	2.027	-42.983	63.111	1.00	49.68	C
ATOM	139	NH1	ARG	A	21	2.510	-41.771	63.388	1.00	41.69	N
ATOM	140	NH2	ARG	A	21	1.094	-43.516	63.887	1.00	51.93	N
ATOM	141	N	LEU	A	22	0.763	-43.407	56.839	1.00	30.03	N
ATOM	142	CA	LEU	A	22	0.057	-44.505	56.199	1.00	29.84	C
ATOM	143	C	LEU	A	22	0.880	-45.773	56.319	1.00	32.54	C
ATOM	144	O	LEU	A	22	2.106	-45.708	56.371	1.00	32.66	O
ATOM	145	CB	LEU	A	22	-0.186	-44.199	54.717	1.00	34.51	C
ATOM	146	CG	LEU	A	22	-1.079	-42.996	54.426	1.00	41.05	C
ATOM	147	CD1	LEU	A	22	-1.294	-42.827	52.928	1.00	32.75	C
ATOM	148	CD2	LEU	A	22	-2.402	-43.136	55.160	1.00	34.25	C
ATOM	149	N	LYS	A	23	0.206	-46.922	56.339	1.00	33.18	N
ATOM	150	CA	LYS	A	23	0.886	-48.212	56.369	1.00	29.77	C
ATOM	151	C	LYS	A	23	1.040	-48.798	54.968	1.00	28.39	C
ATOM	152	O	LYS	A	23	0.213	-48.556	54.091	1.00	31.50	O
ATOM	153	CB	LYS	A	23	0.095	-49.209	57.219	1.00	32.71	C
ATOM	154	CG	LYS	A	23	-0.265	-48.730	58.611	1.00	32.52	C
ATOM	155	CD	LYS	A	23	-1.009	-49.822	59.377	1.00	32.93	C
ATOM	156	CE	LYS	A	23	-2.388	-50.093	58.783	1.00	42.37	C
ATOM	157	NZ	LYS	A	23	-3.017	-51.331	59.345	1.00	45.50	N
ATOM	158	N	THR	A	24	2.115	-49.557	54.772	1.00	26.18	N
ATOM	159	CA	THR	A	24	2.275	-50.447	53.626	1.00	33.85	C
ATOM	160	C	THR	A	24	2.872	-51.734	54.181	1.00	35.23	C

ATOM	161	O	THR	A	24	3.370	-51.749	55.305	1.00	29.03	O
ATOM	162	CB	THR	A	24	3.274	-49.902	52.572	1.00	26.38	C
ATOM	163	OG1	THR	A	24	4.615	-50.193	52.993	1.00	27.27	O
ATOM	164	CG2	THR	A	24	3.098	-48.409	52.361	1.00	30.78	C
ATOM	165	N	PRO	A	25	2.846	-52.817	53.391	1.00	36.40	N
ATOM	166	CA	PRO	A	25	3.500	-54.059	53.813	1.00	31.77	C
ATOM	167	C	PRO	A	25	5.007	-53.919	54.023	1.00	33.56	C
ATOM	168	O	PRO	A	25	5.613	-54.793	54.642	1.00	44.91	O
ATOM	169	CB	PRO	A	25	3.238	-55.000	52.641	1.00	34.71	C
ATOM	170	CG	PRO	A	25	1.988	-54.492	52.033	1.00	38.21	C
ATOM	171	CD	PRO	A	25	2.044	-53.005	52.170	1.00	35.26	C
ATOM	172	N	GLY	A	26	5.603	-52.844	53.520	1.00	29.29	N
ATOM	173	CA	GLY	A	26	7.031	-52.637	53.669	1.00	24.77	C
ATOM	174	C	GLY	A	26	7.382	-51.623	54.743	1.00	30.64	C
ATOM	175	O	GLY	A	26	8.557	-51.299	54.944	1.00	31.70	O
ATOM	176	N	GLY	A	27	6.366	-51.118	55.439	1.00	28.81	N
ATOM	177	CA	GLY	A	27	6.578	-50.081	56.431	1.00	28.06	C
ATOM	178	C	GLY	A	27	5.834	-48.787	56.125	1.00	35.77	C
ATOM	179	O	GLY	A	27	5.211	-48.647	55.065	1.00	34.74	O
ATOM	180	N	PRO	A	28	5.894	-47.822	57.057	1.00	28.77	N
ATOM	181	CA	PRO	A	28	5.105	-46.598	56.925	1.00	29.10	C
ATOM	182	C	PRO	A	28	5.602	-45.621	55.859	1.00	26.94	C
ATOM	183	O	PRO	A	28	6.704	-45.704	55.313	1.00	27.73	O
ATOM	184	CB	PRO	A	28	5.231	-45.957	58.312	1.00	28.73	C
ATOM	185	CG	PRO	A	28	6.532	-46.420	58.801	1.00	28.40	C
ATOM	186	CD	PRO	A	28	6.694	-47.824	58.291	1.00	24.19	C
ATOM	187	N	VAL	A	29	4.754	-44.645	55.601	1.00	24.78	N
ATOM	188	CA	VAL	A	29	5.013	-43.664	54.584	1.00	25.61	C
ATOM	189	C	VAL	A	29	4.299	-42.404	55.056	1.00	29.05	C
ATOM	190	O	VAL	A	29	3.324	-42.498	55.820	1.00	29.22	O
ATOM	191	CB	VAL	A	29	4.472	-44.203	53.235	1.00	31.92	C
ATOM	192	CG1	VAL	A	29	3.634	-43.171	52.505	1.00	24.51	C
ATOM	193	CG2	VAL	A	29	5.614	-44.772	52.385	1.00	27.21	C
ATOM	194	N	SER	A	30	4.801	-41.232	54.657	1.00	27.29	N
ATOM	195	CA	SER	A	30	4.151	-39.965	55.003	1.00	28.01	C
ATOM	196	C	SER	A	30	3.345	-39.451	53.819	1.00	28.27	C
ATOM	197	O	SER	A	30	3.831	-39.428	52.688	1.00	29.39	O
ATOM	198	CB	SER	A	30	5.178	-38.903	55.405	1.00	32.65	C
ATOM	199	OG	SER	A	30	6.116	-39.397	56.347	1.00	37.71	O
ATOM	200	N	ALA	A	31	2.117	-39.029	54.092	1.00	27.82	N
ATOM	201	CA	ALA	A	31	1.220	-38.556	53.060	1.00	30.49	C
ATOM	202	C	ALA	A	31	0.639	-37.202	53.432	1.00	33.65	C
ATOM	203	O	ALA	A	31	0.223	-36.975	54.569	1.00	29.88	O
ATOM	204	CB	ALA	A	31	0.103	-39.559	52.819	1.00	26.08	C

```
ATOM    205  N   PHE A  32       0.625 -36.309  52.452  1.00 27.48           N
ATOM    206  CA  PHE A  32       0.027 -34.997  52.594  1.00 25.43           C
ATOM    207  C   PHE A  32      -0.941 -34.851  51.428  1.00 28.56           C
ATOM    208  O   PHE A  32      -0.537 -34.703  50.258  1.00 27.10           O
ATOM    209  CB  PHE A  32       1.118 -33.924  52.596  1.00 23.49           C
ATOM    210  CG  PHE A  32       2.130 -34.111  53.696  1.00 28.30           C
ATOM    211  CD1 PHE A  32       3.219 -34.953  53.523  1.00 28.07           C
ATOM    212  CD2 PHE A  32       1.980 -33.464  54.914  1.00 29.85           C
ATOM    213  CE1 PHE A  32       4.146 -35.140  54.545  1.00 35.26           C
ATOM    214  CE2 PHE A  32       2.902 -33.650  55.938  1.00 33.86           C
ATOM    215  CZ  PHE A  32       3.984 -34.495  55.751  1.00 27.54           C
ATOM    216  N   LEU A  33      -2.222 -34.947  51.755  1.00 24.65           N
ATOM    217  CA  LEU A  33      -3.268 -35.064  50.759  1.00 25.47           C
ATOM    218  C   LEU A  33      -4.169 -33.858  50.829  1.00 27.68           C
ATOM    219  O   LEU A  33      -4.550 -33.437  51.916  1.00 27.95           O
ATOM    220  CB  LEU A  33      -4.094 -36.326  51.023  1.00 24.75           C
ATOM    221  CG  LEU A  33      -3.287 -37.615  51.213  1.00 26.93           C
ATOM    222  CD1 LEU A  33      -4.200 -38.801  51.538  1.00 23.65           C
ATOM    223  CD2 LEU A  33      -2.443 -37.909  49.971  1.00 27.62           C
ATOM    224  N   GLY A  34      -4.525 -33.308  49.671  1.00 21.27           N
ATOM    225  CA  GLY A  34      -5.476 -32.211  49.633  1.00 21.36           C
ATOM    226  C   GLY A  34      -4.857 -30.864  49.967  1.00 24.57           C
ATOM    227  O   GLY A  34      -5.498 -30.014  50.576  1.00 30.16           O
ATOM    228  N   ILE A  35      -3.604 -30.674  49.564  1.00 25.33           N
ATOM    229  CA  ILE A  35      -2.928 -29.388  49.684  1.00 25.24           C
ATOM    230  C   ILE A  35      -3.395 -28.440  48.587  1.00 27.40           C
ATOM    231  O   ILE A  35      -3.229 -28.731  47.402  1.00 26.75           O
ATOM    232  CB  ILE A  35      -1.429 -29.553  49.473  1.00 24.33           C
ATOM    233  CG1 ILE A  35      -0.859 -30.637  50.398  1.00 22.51           C
ATOM    234  CG2 ILE A  35      -0.736 -28.214  49.637  1.00 26.56           C
ATOM    235  CD1 ILE A  35       0.598 -31.003  50.093  1.00 18.98           C
ATOM    236  N   PRO A  36      -3.974 -27.297  48.968  1.00 27.84           N
ATOM    237  CA  PRO A  36      -4.423 -26.329  47.952  1.00 21.12           C
ATOM    238  C   PRO A  36      -3.253 -25.679  47.206  1.00 27.67           C
ATOM    239  O   PRO A  36      -2.332 -25.163  47.842  1.00 28.79           O
ATOM    240  CB  PRO A  36      -5.186 -25.286  48.772  1.00 25.38           C
ATOM    241  CG  PRO A  36      -4.573 -25.376  50.163  1.00 30.08           C
ATOM    242  CD  PRO A  36      -4.194 -26.827  50.349  1.00 27.02           C
ATOM    243  N   PHE A  37      -3.275 -25.707  45.875  1.00 22.83           N
ATOM    244  CA  PHE A  37      -2.206 -25.062  45.118  1.00 26.61           C
ATOM    245  C   PHE A  37      -2.674 -23.881  44.262  1.00 26.03           C
ATOM    246  O   PHE A  37      -1.858 -23.139  43.722  1.00 29.33           O
ATOM    247  CB  PHE A  37      -1.386 -26.079  44.301  1.00 29.84           C
ATOM    248  CG  PHE A  37      -2.120 -26.684  43.129  1.00 21.80           C
```

ATOM	249	CD1	PHE	A	37	-2.121	-26.050	41.889	1.00	24.95	C
ATOM	250	CD2	PHE	A	37	-2.760	-27.915	43.253	1.00	21.93	C
ATOM	251	CE1	PHE	A	37	-2.782	-26.620	40.788	1.00	26.08	C
ATOM	252	CE2	PHE	A	37	-3.424	-28.500	42.160	1.00	21.82	C
ATOM	253	CZ	PHE	A	37	-3.437	-27.847	40.928	1.00	22.95	C
ATOM	254	N	ALA	A	38	-3.986	-23.703	44.155	1.00	22.72	N
ATOM	255	CA	ALA	A	38	-4.544	-22.561	43.453	1.00	22.93	C
ATOM	256	C	ALA	A	38	-5.753	-22.049	44.204	1.00	27.43	C
ATOM	257	O	ALA	A	38	-6.320	-22.756	45.037	1.00	21.52	O
ATOM	258	CB	ALA	A	38	-4.940	-22.941	42.019	1.00	21.83	C
ATOM	259	N	GLU	A	39	-6.149	-20.815	43.907	1.00	30.23	N
ATOM	260	CA	GLU	A	39	-7.433	-20.309	44.372	1.00	28.42	C
ATOM	261	C	GLU	A	39	-8.532	-21.126	43.688	1.00	33.53	C
ATOM	262	O	GLU	A	39	-8.376	-21.537	42.533	1.00	25.87	O
ATOM	263	CB	GLU	A	39	-7.569	-18.816	44.041	1.00	26.70	C
ATOM	264	CG	GLU	A	39	-6.706	-17.908	44.917	1.00	31.16	C
ATOM	265	CD	GLU	A	39	-7.094	-17.992	46.392	1.00	39.36	C
ATOM	266	OE1	GLU	A	39	-8.283	-17.771	46.707	1.00	44.15	O
ATOM	267	OE2	GLU	A	39	-6.221	-18.304	47.232	1.00	36.49	O
ATOM	268	N	PRO	A	40	-9.634	-21.394	44.402	1.00	30.29	N
ATOM	269	CA	PRO	A	40	-10.712	-22.158	43.759	1.00	25.69	C
ATOM	270	C	PRO	A	40	-11.230	-21.464	42.497	1.00	28.41	C
ATOM	271	O	PRO	A	40	-11.714	-20.340	42.567	1.00	34.41	O
ATOM	272	CB	PRO	A	40	-11.786	-22.216	44.844	1.00	28.73	C
ATOM	273	CG	PRO	A	40	-10.988	-22.179	46.147	1.00	22.70	C
ATOM	274	CD	PRO	A	40	-9.849	-21.225	45.850	1.00	27.33	C
ATOM	275	N	PRO	A	41	-11.106	-22.128	41.341	1.00	29.22	N
ATOM	276	CA	PRO	A	41	-11.497	-21.518	40.065	1.00	29.57	C
ATOM	277	C	PRO	A	41	-13.022	-21.519	39.879	1.00	36.71	C
ATOM	278	O	PRO	A	41	-13.547	-22.216	39.007	1.00	30.33	O
ATOM	279	CB	PRO	A	41	-10.812	-22.413	39.034	1.00	24.51	C
ATOM	280	CG	PRO	A	41	-10.733	-23.753	39.692	1.00	27.29	C
ATOM	281	CD	PRO	A	41	-10.609	-23.507	41.176	1.00	25.77	C
ATOM	282	N	MET	A	42	-13.710	-20.722	40.695	1.00	32.51	N
ATOM	283	CA	MET	A	42	-15.171	-20.715	40.756	1.00	36.67	C
ATOM	284	C	MET	A	42	-15.781	-19.361	40.374	1.00	38.40	C
ATOM	285	O	MET	A	42	-15.095	-18.332	40.358	1.00	34.05	O
ATOM	286	CB	MET	A	42	-15.630	-21.098	42.166	1.00	39.73	C
ATOM	287	CG	MET	A	42	-15.072	-22.427	42.641	1.00	36.68	C
ATOM	288	SD	MET	A	42	-16.137	-23.231	43.839	1.00	57.16	S
ATOM	289	CE	MET	A	42	-16.049	-22.070	45.209	1.00	49.99	C
ATOM	290	N	GLY	A	43	-17.080	-19.371	40.080	1.00	30.37	N
ATOM	291	CA	GLY	A	43	-17.784	-18.161	39.699	1.00	33.05	C
ATOM	292	C	GLY	A	43	-17.139	-17.463	38.516	1.00	34.49	C

ATOM	293	O	GLY	A	43	-17.038	-18.032	37.425	1.00	30.76	O
ATOM	294	N	PRO	A	44	-16.685	-16.223	38.728	1.00	35.11	N
ATOM	295	CA	PRO	A	44	-16.048	-15.464	37.643	1.00	31.66	C
ATOM	296	C	PRO	A	44	-14.722	-16.085	37.197	1.00	35.68	C
ATOM	297	O	PRO	A	44	-14.259	-15.773	36.103	1.00	33.51	O
ATOM	298	CB	PRO	A	44	-15.832	-14.078	38.256	1.00	31.49	C
ATOM	299	CG	PRO	A	44	-15.797	-14.326	39.755	1.00	35.04	C
ATOM	300	CD	PRO	A	44	-16.721	-15.481	40.004	1.00	31.47	C
ATOM	301	N	ARG	A	45	-14.139	-16.961	38.017	1.00	36.60	N
ATOM	302	CA	ARG	A	45	-12.887	-17.638	37.666	1.00	34.54	C
ATOM	303	C	ARG	A	45	-13.052	-18.905	36.811	1.00	32.23	C
ATOM	304	O	ARG	A	45	-12.057	-19.465	36.349	1.00	30.53	O
ATOM	305	CB	ARG	A	45	-12.069	-17.990	38.915	1.00	32.99	C
ATOM	306	CG	ARG	A	45	-11.555	-16.810	39.738	1.00	43.08	C
ATOM	307	CD	ARG	A	45	-10.804	-15.756	38.916	1.00	53.00	C
ATOM	308	NE	ARG	A	45	-9.636	-16.262	38.188	1.00	59.27	N
ATOM	309	CZ	ARG	A	45	-8.753	-15.481	37.561	1.00	65.17	C
ATOM	310	NH1	ARG	A	45	-8.898	-14.159	37.589	1.00	56.69	N
ATOM	311	NH2	ARG	A	45	-7.719	-16.013	36.910	1.00	53.00	N
ATOM	312	N	ARG	A	46	-14.282	-19.375	36.620	1.00	29.33	N
ATOM	313	CA	ARG	A	46	-14.516	-20.510	35.725	1.00	27.71	C
ATOM	314	C	ARG	A	46	-13.998	-20.198	34.315	1.00	27.01	C
ATOM	315	O	ARG	A	46	-14.216	-19.101	33.805	1.00	31.91	O
ATOM	316	CB	ARG	A	46	-16.003	-20.878	35.676	1.00	28.48	C
ATOM	317	CG	ARG	A	46	-16.322	-22.024	34.719	1.00	27.46	C
ATOM	318	CD	ARG	A	46	-17.818	-22.264	34.580	1.00	26.70	C
ATOM	319	NE	ARG	A	46	-18.384	-23.018	35.700	1.00	29.15	N
ATOM	320	CZ	ARG	A	46	-19.688	-23.224	35.866	1.00	31.56	C
ATOM	321	NH1	ARG	A	46	-20.541	-22.730	34.979	1.00	30.91	N
ATOM	322	NH2	ARG	A	46	-20.144	-23.919	36.907	1.00	23.30	N
ATOM	323	N	PHE	A	47	-13.301	-21.165	33.716	1.00	28.25	N
ATOM	324	CA	PHE	A	47	-12.699	-21.055	32.371	1.00	31.69	C
ATOM	325	C	PHE	A	47	-11.408	-20.228	32.329	1.00	29.70	C
ATOM	326	O	PHE	A	47	-10.770	-20.118	31.273	1.00	27.89	O
ATOM	327	CB	PHE	A	47	-13.672	-20.502	31.310	1.00	27.02	C
ATOM	328	CG	PHE	A	47	-14.994	-21.212	31.238	1.00	27.00	C
ATOM	329	CD1	PHE	A	47	-15.059	-22.575	31.007	1.00	24.51	C
ATOM	330	CD2	PHE	A	47	-16.182	-20.494	31.349	1.00	28.87	C
ATOM	331	CE1	PHE	A	47	-16.285	-23.220	30.916	1.00	27.39	C
ATOM	332	CE2	PHE	A	47	-17.413	-21.126	31.256	1.00	21.67	C
ATOM	333	CZ	PHE	A	47	-17.471	-22.490	31.044	1.00	24.31	C
ATOM	334	N	LEU	A	48	-11.031	-19.638	33.460	1.00	26.60	N
ATOM	335	CA	LEU	A	48	-9.857	-18.770	33.501	1.00	30.25	C
ATOM	336	C	LEU	A	48	-8.620	-19.518	33.986	1.00	31.08	C

ATOM	337	O	LEU	A	48	-8.743	-20.533	34.676	1.00	25.38	O
ATOM	338	CB	LEU	A	48	-10.112	-17.555	34.398	1.00	34.18	C
ATOM	339	CG	LEU	A	48	-11.130	-16.505	33.940	1.00	35.89	C
ATOM	340	CD1	LEU	A	48	-10.997	-15.238	34.787	1.00	34.69	C
ATOM	341	CD2	LEU	A	48	-10.979	-16.192	32.467	1.00	34.23	C
ATOM	342	N	PRO	A	49	-7.422	-19.023	33.617	1.00	29.00	N
ATOM	343	CA	PRO	A	49	-6.164	-19.563	34.148	1.00	25.22	C
ATOM	344	C	PRO	A	49	-6.186	-19.599	35.666	1.00	28.57	C
ATOM	345	O	PRO	A	49	-6.831	-18.763	36.295	1.00	31.57	O
ATOM	346	CB	PRO	A	49	-5.129	-18.537	33.691	1.00	23.67	C
ATOM	347	CG	PRO	A	49	-5.695	-17.999	32.410	1.00	31.86	C
ATOM	348	CD	PRO	A	49	-7.189	-17.964	32.616	1.00	26.41	C
ATOM	349	N	PRO	A	50	-5.478	-20.558	36.258	1.00	26.46	N
ATOM	350	CA	PRO	A	50	-5.453	-20.667	37.718	1.00	25.74	C
ATOM	351	C	PRO	A	50	-4.658	-19.521	38.356	1.00	31.16	C
ATOM	352	O	PRO	A	50	-3.694	-19.047	37.763	1.00	29.04	O
ATOM	353	CB	PRO	A	50	-4.732	-21.998	37.944	1.00	25.39	C
ATOM	354	CG	PRO	A	50	-3.815	-22.131	36.737	1.00	22.68	C
ATOM	355	CD	PRO	A	50	-4.629	-21.570	35.598	1.00	23.59	C
ATOM	356	N	GLU	A	51	-5.072	-19.078	39.540	1.00	29.94	N
ATOM	357	CA	GLU	A	51	-4.303	-18.115	40.322	1.00	33.02	C
ATOM	358	C	GLU	A	51	-3.632	-18.858	41.468	1.00	30.96	C
ATOM	359	O	GLU	A	51	-4.231	-19.758	42.063	1.00	30.72	O
ATOM	360	CB	GLU	A	51	-5.209	-17.032	40.920	1.00	32.89	C
ATOM	361	CG	GLU	A	51	-6.167	-16.358	39.946	1.00	50.33	C
ATOM	362	CD	GLU	A	51	-7.187	-15.475	40.663	1.00	65.18	C
ATOM	363	OE1	GLU	A	51	-8.256	-15.991	41.078	1.00	61.78	O
ATOM	364	OE2	GLU	A	51	-6.913	-14.263	40.820	1.00	71.56	O
ATOM	365	N	PRO	A	52	-2.389	-18.484	41.788	1.00	29.16	N
ATOM	366	CA	PRO	A	52	-1.691	-19.089	42.930	1.00	27.90	C
ATOM	367	C	PRO	A	52	-2.517	-19.002	44.213	1.00	30.01	C
ATOM	368	O	PRO	A	52	-3.206	-18.006	44.445	1.00	28.32	O
ATOM	369	CB	PRO	A	52	-0.423	-18.244	43.038	1.00	27.69	C
ATOM	370	CG	PRO	A	52	-0.161	-17.803	41.614	1.00	22.16	C
ATOM	371	CD	PRO	A	52	-1.526	-17.556	41.031	1.00	24.99	C
ATOM	372	N	LYS	A	53	-2.487	-20.060	45.013	1.00	27.89	N
ATOM	373	CA	LYS	A	53	-3.222	-20.070	46.273	1.00	30.42	C
ATOM	374	C	LYS	A	53	-2.661	-19.001	47.213	1.00	32.66	C
ATOM	375	O	LYS	A	53	-1.449	-18.917	47.409	1.00	30.34	O
ATOM	376	CB	LYS	A	53	-3.133	-21.453	46.930	1.00	26.75	C
ATOM	377	CG	LYS	A	53	-3.696	-21.525	48.348	1.00	30.16	C
ATOM	378	CD	LYS	A	53	-5.206	-21.372	48.350	1.00	31.12	C
ATOM	379	CE	LYS	A	53	-5.789	-21.655	49.719	1.00	26.92	C
ATOM	380	NZ	LYS	A	53	-7.259	-21.391	49.739	1.00	28.34	N

ATOM	381	N	GLN	A	54	-3.548	-18.196	47.787	0.43	31.53	N
ATOM	383	CA	GLN	A	54	-3.155	-17.179	48.758	0.43	35.29	C
ATOM	385	C	GLN	A	54	-2.765	-17.837	50.083	0.43	34.30	C
ATOM	387	O	GLN	A	54	-3.389	-18.817	50.494	0.43	33.25	O
ATOM	389	CB	GLN	A	54	-4.297	-16.172	48.966	0.43	34.67	C
ATOM	391	CG	GLN	A	54	-4.604	-15.322	47.731	0.43	35.67	C
ATOM	393	CD	GLN	A	54	-6.033	-14.794	47.700	0.43	38.48	C
ATOM	395	OE1	GLN	A	54	-6.537	-14.406	46.643	0.43	38.77	O
ATOM	397	NE2	GLN	A	54	-6.691	-14.780	48.855	0.43	33.35	N
ATOM	399	N	PRO	A	55	-1.721	-17.312	50.747	1.00	33.15	N
ATOM	400	CA	PRO	A	55	-1.291	-17.843	52.052	1.00	34.19	C
ATOM	401	C	PRO	A	55	-2.418	-17.894	53.084	1.00	33.93	C
ATOM	402	O	PRO	A	55	-3.324	-17.061	53.061	1.00	31.90	O
ATOM	403	CB	PRO	A	55	-0.197	-16.866	52.492	1.00	30.69	C
ATOM	404	CG	PRO	A	55	0.360	-16.351	51.208	1.00	36.57	C
ATOM	405	CD	PRO	A	55	-0.825	-16.248	50.264	1.00	30.62	C
ATOM	406	N	TRP	A	56	-2.361	-18.897	53.956	1.00	30.06	N
ATOM	407	CA	TRP	A	56	-3.368	-19.114	54.985	1.00	31.65	C
ATOM	408	C	TRP	A	56	-2.706	-19.075	56.366	1.00	37.29	C
ATOM	409	O	TRP	A	56	-1.498	-19.319	56.505	1.00	29.83	O
ATOM	410	CB	TRP	A	56	-4.045	-20.478	54.787	1.00	29.54	C
ATOM	411	CG	TRP	A	56	-3.063	-21.622	54.828	1.00	30.99	C
ATOM	412	CD1	TRP	A	56	-2.635	-22.296	55.935	1.00	31.93	C
ATOM	413	CD2	TRP	A	56	-2.369	-22.200	53.715	1.00	31.44	C
ATOM	414	NE1	TRP	A	56	-1.722	-23.257	55.582	1.00	33.07	N
ATOM	415	CE2	TRP	A	56	-1.542	-23.221	54.224	1.00	30.32	C
ATOM	416	CE3	TRP	A	56	-2.371	-21.956	52.336	1.00	31.14	C
ATOM	417	CZ2	TRP	A	56	-0.727	-24.004	53.403	1.00	26.50	C
ATOM	418	CZ3	TRP	A	56	-1.562	-22.735	51.521	1.00	28.31	C
ATOM	419	CH2	TRP	A	56	-0.748	-23.744	52.060	1.00	28.66	C
ATOM	420	N	SER	A	57	-3.495	-18.762	57.384	1.00	33.45	N
ATOM	421	CA	SER	A	57	-3.008	-18.826	58.754	1.00	39.57	C
ATOM	422	C	SER	A	57	-3.492	-20.140	59.348	1.00	41.61	C
ATOM	423	O	SER	A	57	-4.399	-20.772	58.805	1.00	41.55	O
ATOM	424	CB	SER	A	57	-3.533	-17.646	59.564	1.00	41.88	C
ATOM	425	OG	SER	A	57	-4.948	-17.668	59.611	1.00	45.04	O
ATOM	426	N	GLY	A	58	-2.888	-20.560	60.453	1.00	40.86	N
ATOM	427	CA	GLY	A	58	-3.241	-21.838	61.043	1.00	37.53	C
ATOM	428	C	GLY	A	58	-2.747	-23.020	60.219	1.00	44.22	C
ATOM	429	O	GLY	A	58	-1.904	-22.871	59.331	1.00	41.44	O
ATOM	430	N	VAL	A	59	-3.283	-24.201	60.507	1.00	39.11	N
ATOM	431	CA	VAL	A	59	-2.763	-25.434	59.933	1.00	41.07	C
ATOM	432	C	VAL	A	59	-3.782	-26.101	59.022	1.00	40.03	C
ATOM	433	O	VAL	A	59	-4.849	-26.518	59.480	1.00	35.57	O

ATOM	434	CB	VAL	A	59	-2.387	-26.444	61.038	1.00	40.95	C
ATOM	435	CG1	VAL	A	59	-1.654	-27.644	60.440	1.00	36.13	C
ATOM	436	CG2	VAL	A	59	-1.544	-25.775	62.120	1.00	32.77	C
ATOM	437	N	VAL	A	60	-3.450	-26.208	57.736	1.00	35.85	N
ATOM	438	CA	VAL	A	60	-4.287	-26.952	56.792	1.00	32.06	C
ATOM	439	C	VAL	A	60	-4.243	-28.457	57.071	1.00	34.95	C
ATOM	440	O	VAL	A	60	-3.170	-29.067	57.178	1.00	32.51	O
ATOM	441	CB	VAL	A	60	-3.909	-26.664	55.316	1.00	31.60	C
ATOM	442	CG1	VAL	A	60	-4.473	-27.728	54.390	1.00	29.25	C
ATOM	443	CG2	VAL	A	60	-4.410	-25.292	54.898	1.00	31.86	C
ATOM	444	N	ASP	A	61	-5.428	-29.037	57.211	1.00	33.02	N
ATOM	445	CA	ASP	A	61	-5.593	-30.470	57.374	1.00	38.98	C
ATOM	446	C	ASP	A	61	-5.325	-31.192	56.052	1.00	41.18	C
ATOM	447	O	ASP	A	61	-6.156	-31.171	55.146	1.00	42.08	O
ATOM	448	CB	ASP	A	61	-7.020	-30.757	57.837	1.00	38.86	C
ATOM	449	CG	ASP	A	61	-7.355	-32.230	57.821	1.00	47.45	C
ATOM	450	OD1	ASP	A	61	-6.435	-33.057	57.981	1.00	52.46	O
ATOM	451	OD2	ASP	A	61	-8.546	-32.563	57.655	1.00	57.14	O
ATOM	452	N	ALA	A	62	-4.167	-31.829	55.940	1.00	33.88	N
ATOM	453	CA	ALA	A	62	-3.833	-32.544	54.718	1.00	33.27	C
ATOM	454	C	ALA	A	62	-3.819	-34.053	54.945	1.00	29.34	C
ATOM	455	O	ALA	A	62	-2.835	-34.719	54.636	1.00	26.52	O
ATOM	456	CB	ALA	A	62	-2.478	-32.074	54.169	1.00	28.50	C
ATOM	457	N	THR	A	63	-4.911	-34.589	55.480	1.00	29.97	N
ATOM	458	CA	THR	A	63	-4.940	-35.992	55.881	1.00	34.87	C
ATOM	459	C	THR	A	63	-5.886	-36.800	55.006	1.00	34.98	C
ATOM	460	O	THR	A	63	-6.021	-38.014	55.177	1.00	34.94	O
ATOM	461	CB	THR	A	63	-5.381	-36.158	57.362	1.00	35.26	C
ATOM	462	OG1	THR	A	63	-6.697	-35.620	57.530	1.00	32.20	O
ATOM	463	CG2	THR	A	63	-4.432	-35.438	58.289	1.00	30.47	C
ATOM	464	N	THR	A	64	-6.539	-36.127	54.067	1.00	32.28	N
ATOM	465	CA	THR	A	64	-7.572	-36.774	53.273	1.00	29.96	C
ATOM	466	C	THR	A	64	-7.635	-36.208	51.833	1.00	32.97	C
ATOM	467	O	THR	A	64	-7.289	-35.046	51.599	1.00	31.19	O
ATOM	468	CB	THR	A	64	-8.936	-36.683	54.021	1.00	31.00	C
ATOM	469	OG1	THR	A	64	-9.899	-37.536	53.398	1.00	46.09	O
ATOM	470	CG2	THR	A	64	-9.452	-35.252	54.050	1.00	25.37	C
ATOM	471	N	PHE	A	65	-8.037	-37.035	50.868	1.00	26.42	N
ATOM	472	CA	PHE	A	65	-8.167	-36.570	49.491	1.00	25.38	C
ATOM	473	C	PHE	A	65	-9.244	-35.504	49.384	1.00	26.15	C
ATOM	474	O	PHE	A	65	-10.265	-35.560	50.068	1.00	28.45	O
ATOM	475	CB	PHE	A	65	-8.491	-37.719	48.529	1.00	25.63	C
ATOM	476	CG	PHE	A	65	-7.315	-38.586	48.196	1.00	20.84	C
ATOM	477	CD1	PHE	A	65	-6.187	-38.047	47.605	1.00	20.39	C

ATOM	478	CD2	PHE	A	65	-7.348	-39.946	48.448	1.00	20.16	C
ATOM	479	CE1	PHE	A	65	-5.098	-38.849	47.292	1.00	21.43	C
ATOM	480	CE2	PHE	A	65	-6.264	-40.761	48.134	1.00	18.76	C
ATOM	481	CZ	PHE	A	65	-5.135	-40.205	47.556	1.00	22.11	C
ATOM	482	N	GLN	A	66	-9.013	-34.532	48.514	1.00	24.40	N
ATOM	483	CA	GLN	A	66	-9.978	-33.472	48.296	1.00	22.11	C
ATOM	484	C	GLN	A	66	-10.901	-33.816	47.126	1.00	26.84	C
ATOM	485	O	GLN	A	66	-10.762	-34.872	46.498	1.00	25.86	O
ATOM	486	CB	GLN	A	66	-9.247	-32.159	48.026	1.00	27.01	C
ATOM	487	CG	GLN	A	66	-9.063	-31.289	49.236	1.00	24.82	C
ATOM	488	CD	GLN	A	66	-10.328	-30.532	49.594	1.00	35.60	C
ATOM	489	OE1	GLN	A	66	-11.357	-30.645	48.914	1.00	35.85	O
ATOM	490	NE2	GLN	A	66	-10.256	-29.742	50.657	1.00	26.54	N
ATOM	491	N	SER	A	67	-11.824	-32.907	46.829	1.00	19.94	N
ATOM	492	CA	SER	A	67	-12.853	-33.130	45.828	1.00	26.24	C
ATOM	493	C	SER	A	67	-12.318	-33.430	44.418	1.00	29.68	C
ATOM	494	O	SER	A	67	-11.234	-32.987	44.030	1.00	24.61	O
ATOM	495	CB	SER	A	67	-13.773	-31.917	45.773	1.00	27.81	C
ATOM	496	OG	SER	A	67	-14.263	-31.605	47.063	1.00	34.46	O
ATOM	497	N	VAL	A	68	-13.099	-34.194	43.663	1.00	26.74	N
ATOM	498	CA	VAL	A	68	-12.799	-34.471	42.265	1.00	22.55	C
ATOM	499	C	VAL	A	68	-13.315	-33.322	41.400	1.00	23.76	C
ATOM	500	O	VAL	A	68	-14.427	-32.828	41.615	1.00	23.36	O
ATOM	501	CB	VAL	A	68	-13.425	-35.815	41.833	1.00	24.54	C
ATOM	502	CG1	VAL	A	68	-13.235	-36.067	40.335	1.00	22.63	C
ATOM	503	CG2	VAL	A	68	-12.828	-36.955	42.662	1.00	18.61	C
ATOM	504	N	CYS	A	69	-12.492	-32.869	40.457	1.00	20.75	N
ATOM	505	CA	CYS	A	69	-12.910	-31.827	39.515	1.00	24.58	C
ATOM	506	C	CYS	A	69	-14.135	-32.299	38.768	1.00	26.10	C
ATOM	507	O	CYS	A	69	-14.199	-33.463	38.367	1.00	24.20	O
ATOM	508	CB	CYS	A	69	-11.790	-31.501	38.518	1.00	22.99	C
ATOM	509	SG	CYS	A	69	-10.349	-30.673	39.265	1.00	27.26	S
ATOM	510	N	TYR	A	70	-15.100	-31.398	38.589	1.00	25.05	N
ATOM	511	CA	TYR	A	70	-16.350	-31.724	37.903	1.00	26.87	C
ATOM	512	C	TYR	A	70	-16.167	-32.441	36.568	1.00	27.98	C
ATOM	513	O	TYR	A	70	-15.441	-31.970	35.691	1.00	25.35	O
ATOM	514	CB	TYR	A	70	-17.196	-30.471	37.701	1.00	30.15	C
ATOM	515	CG	TYR	A	70	-18.461	-30.525	38.506	1.00	32.51	C
ATOM	516	CD1	TYR	A	70	-18.555	-29.869	39.728	1.00	26.53	C
ATOM	517	CD2	TYR	A	70	-19.557	-31.264	38.063	1.00	32.44	C
ATOM	518	CE1	TYR	A	70	-19.713	-29.922	40.482	1.00	29.37	C
ATOM	519	CE2	TYR	A	70	-20.725	-31.330	38.819	1.00	36.45	C
ATOM	520	CZ	TYR	A	70	-20.789	-30.657	40.029	1.00	33.93	C
ATOM	521	OH	TYR	A	70	-21.936	-30.706	40.787	1.00	52.19	O

```
ATOM    522  N   GLN A  71     -16.843 -33.578  36.421  1.00 27.96           N
ATOM    523  CA  GLN A  71     -16.658 -34.421  35.255  1.00 25.32           C
ATOM    524  C   GLN A  71     -17.798 -35.417  35.074  1.00 31.39           C
ATOM    525  O   GLN A  71     -18.510 -35.747  36.032  1.00 24.05           O
ATOM    526  CB  GLN A  71     -15.346 -35.185  35.372  1.00 21.80           C
ATOM    527  CG  GLN A  71     -15.315 -36.174  36.519  1.00 20.79           C
ATOM    528  CD  GLN A  71     -13.930 -36.738  36.749  1.00 23.12           C
ATOM    529  OE1 GLN A  71     -13.699 -37.937  36.582  1.00 20.38           O
ATOM    530  NE2 GLN A  71     -12.998 -35.878  37.153  1.00 22.86           N
ATOM    531  N   TYR A  72     -17.955 -35.865  33.827  1.00 23.07           N
ATOM    532  CA  TYR A  72     -18.878 -36.918  33.444  1.00 21.23           C
ATOM    533  C   TYR A  72     -18.592 -38.187  34.233  1.00 25.36           C
ATOM    534  O   TYR A  72     -17.429 -38.491  34.518  1.00 28.13           O
ATOM    535  CB  TYR A  72     -18.747 -37.186  31.928  1.00 26.46           C
ATOM    536  CG  TYR A  72     -19.487 -38.417  31.457  1.00 25.78           C
ATOM    537  CD1 TYR A  72     -20.864 -38.394  31.276  1.00 25.20           C
ATOM    538  CD2 TYR A  72     -18.811 -39.607  31.202  1.00 22.40           C
ATOM    539  CE1 TYR A  72     -21.553 -39.523  30.851  1.00 26.16           C
ATOM    540  CE2 TYR A  72     -19.492 -40.739  30.777  1.00 27.90           C
ATOM    541  CZ  TYR A  72     -20.865 -40.691  30.609  1.00 27.12           C
ATOM    542  OH  TYR A  72     -21.555 -41.812  30.201  1.00 26.34           O
ATOM    543  N   VAL A  73     -19.651 -38.910  34.606  1.00 26.54           N
ATOM    544  CA  VAL A  73     -19.520 -40.178  35.330  1.00 25.16           C
ATOM    545  C   VAL A  73     -19.941 -41.323  34.410  1.00 25.01           C
ATOM    546  O   VAL A  73     -21.075 -41.362  33.938  1.00 31.16           O
ATOM    547  CB  VAL A  73     -20.364 -40.206  36.652  1.00 30.05           C
ATOM    548  CG1 VAL A  73     -20.243 -41.549  37.356  1.00 26.01           C
ATOM    549  CG2 VAL A  73     -19.937 -39.099  37.601  1.00 22.33           C
ATOM    550  N   ASP A  74     -19.015 -42.237  34.143  1.00 27.32           N
ATOM    551  CA  ASP A  74     -19.239 -43.349  33.218  1.00 31.49           C
ATOM    552  C   ASP A  74     -20.332 -44.301  33.714  1.00 29.08           C
ATOM    553  O   ASP A  74     -20.139 -45.000  34.694  1.00 30.74           O
ATOM    554  CB  ASP A  74     -17.932 -44.128  33.032  1.00 29.75           C
ATOM    555  CG  ASP A  74     -17.992 -45.135  31.878  1.00 34.55           C
ATOM    556  OD1 ASP A  74     -19.077 -45.335  31.303  1.00 32.93           O
ATOM    557  OD2 ASP A  74     -16.946 -45.737  31.549  1.00 37.02           O
ATOM    558  N   THR A  75     -21.461 -44.352  33.012  1.00 32.23           N
ATOM    559  CA  THR A  75     -22.558 -45.249  33.393  1.00 34.66           C
ATOM    560  C   THR A  75     -22.797 -46.421  32.429  1.00 40.32           C
ATOM    561  O   THR A  75     -23.882 -47.002  32.428  1.00 36.31           O
ATOM    562  CB  THR A  75     -23.891 -44.487  33.549  1.00 28.12           C
ATOM    563  OG1 THR A  75     -24.119 -43.667  32.391  1.00 33.41           O
ATOM    564  CG2 THR A  75     -23.874 -43.615  34.792  1.00 25.24           C
ATOM    565  N   LEU A  76     -21.796 -46.775  31.626  1.00 38.02           N
```

```
ATOM    566  CA  LEU A  76     -21.956 -47.842  30.635  1.00 36.60           C
ATOM    567  C   LEU A  76     -22.394 -49.177  31.246  1.00 41.25           C
ATOM    568  O   LEU A  76     -23.337 -49.818  30.761  1.00 39.67           O
ATOM    569  CB  LEU A  76     -20.672 -48.048  29.828  1.00 33.09           C
ATOM    570  CG  LEU A  76     -20.762 -49.111  28.731  1.00 36.15           C
ATOM    571  CD1 LEU A  76     -21.778 -48.696  27.678  1.00 26.38           C
ATOM    572  CD2 LEU A  76     -19.404 -49.332  28.100  1.00 35.68           C
ATOM    573  N   TYR A  77     -21.700 -49.608  32.292  1.00 28.58           N
ATOM    574  CA  TYR A  77     -22.074 -50.846  32.959  1.00 31.33           C
ATOM    575  C   TYR A  77     -22.210 -50.600  34.460  1.00 33.33           C
ATOM    576  O   TYR A  77     -21.290 -50.885  35.217  1.00 31.60           O
ATOM    577  CB  TYR A  77     -21.044 -51.946  32.696  1.00 33.27           C
ATOM    578  CG  TYR A  77     -20.918 -52.391  31.245  1.00 42.31           C
ATOM    579  CD1 TYR A  77     -21.958 -53.057  30.601  1.00 40.46           C
ATOM    580  CD2 TYR A  77     -19.740 -52.171  30.530  1.00 37.43           C
ATOM    581  CE1 TYR A  77     -21.833 -53.473  29.276  1.00 41.00           C
ATOM    582  CE2 TYR A  77     -19.608 -52.580  29.213  1.00 34.21           C
ATOM    583  CZ  TYR A  77     -20.652 -53.229  28.587  1.00 41.56           C
ATOM    584  OH  TYR A  77     -20.507 -53.631  27.270  1.00 41.25           O
ATOM    585  N   PRO A  78     -23.362 -50.059  34.889  1.00 36.04           N
ATOM    586  CA  PRO A  78     -23.577 -49.661  36.292  1.00 34.13           C
ATOM    587  C   PRO A  78     -23.259 -50.776  37.272  1.00 32.92           C
ATOM    588  O   PRO A  78     -23.841 -51.854  37.185  1.00 37.25           O
ATOM    589  CB  PRO A  78     -25.069 -49.334  36.339  1.00 30.45           C
ATOM    590  CG  PRO A  78     -25.401 -48.916  34.934  1.00 39.15           C
ATOM    591  CD  PRO A  78     -24.541 -49.781  34.044  1.00 35.39           C
ATOM    592  N   GLY A  79     -22.323 -50.517  38.179  1.00 34.95           N
ATOM    593  CA  GLY A  79     -21.968 -51.476  39.207  1.00 34.30           C
ATOM    594  C   GLY A  79     -20.724 -52.271  38.873  1.00 35.32           C
ATOM    595  O   GLY A  79     -20.012 -52.739  39.755  1.00 36.39           O
ATOM    596  N   PHE A  80     -20.458 -52.418  37.585  1.00 37.05           N
ATOM    597  CA  PHE A  80     -19.299 -53.170  37.122  1.00 35.14           C
ATOM    598  C   PHE A  80     -17.994 -52.529  37.622  1.00 31.41           C
ATOM    599  O   PHE A  80     -17.756 -51.347  37.415  1.00 35.46           O
ATOM    600  CB  PHE A  80     -19.352 -53.251  35.594  1.00 29.41           C
ATOM    601  CG  PHE A  80     -18.248 -54.046  34.980  1.00 31.75           C
ATOM    602  CD1 PHE A  80     -17.980 -55.335  35.411  1.00 29.32           C
ATOM    603  CD2 PHE A  80     -17.495 -53.513  33.938  1.00 25.79           C
ATOM    604  CE1 PHE A  80     -16.965 -56.081  34.833  1.00 30.14           C
ATOM    605  CE2 PHE A  80     -16.477 -54.252  33.356  1.00 28.60           C
ATOM    606  CZ  PHE A  80     -16.211 -55.540  33.801  1.00 28.60           C
ATOM    607  N   GLU A  81     -17.166 -53.315  38.293  1.00 30.54           N
ATOM    608  CA  GLU A  81     -15.877 -52.852  38.793  1.00 32.97           C
ATOM    609  C   GLU A  81     -14.986 -52.267  37.688  1.00 39.81           C
```

```
ATOM    610  O   GLU A  81     -14.229 -51.315  37.919  1.00 30.76           O
ATOM    611  CB  GLU A  81     -15.154 -54.004  39.509  1.00 38.53           C
ATOM    612  CG  GLU A  81     -13.671 -53.774  39.822  1.00 56.65           C
ATOM    613  CD  GLU A  81     -13.427 -52.791  40.971  1.00 71.31           C
ATOM    614  OE1 GLU A  81     -14.401 -52.162  41.456  1.00 67.23           O
ATOM    615  OE2 GLU A  81     -12.251 -52.648  41.390  1.00 64.44           O
ATOM    616  N   GLY A  82     -15.094 -52.829  36.485  1.00 33.07           N
ATOM    617  CA  GLY A  82     -14.231 -52.449  35.382  1.00 28.36           C
ATOM    618  C   GLY A  82     -14.406 -51.019  34.911  1.00 27.54           C
ATOM    619  O   GLY A  82     -13.452 -50.396  34.430  1.00 27.99           O
ATOM    620  N   THR A  83     -15.624 -50.500  35.029  1.00 27.68           N
ATOM    621  CA  THR A  83     -15.876 -49.098  34.724  1.00 31.99           C
ATOM    622  C   THR A  83     -15.816 -48.226  35.986  1.00 34.89           C
ATOM    623  O   THR A  83     -15.403 -47.062  35.927  1.00 32.43           O
ATOM    624  CB  THR A  83     -17.249 -48.879  34.048  1.00 30.33           C
ATOM    625  OG1 THR A  83     -18.263 -49.542  34.807  1.00 37.88           O
ATOM    626  CG2 THR A  83     -17.253 -49.406  32.629  1.00 30.97           C
ATOM    627  N   GLU A  84     -16.231 -48.781  37.121  1.00 28.50           N
ATOM    628  CA  GLU A  84     -16.363 -47.977  38.335  1.00 32.61           C
ATOM    629  C   GLU A  84     -15.026 -47.553  38.938  1.00 30.40           C
ATOM    630  O   GLU A  84     -14.926 -46.484  39.528  1.00 29.64           O
ATOM    631  CB  GLU A  84     -17.199 -48.701  39.393  1.00 33.86           C
ATOM    632  CG  GLU A  84     -18.646 -48.959  39.000  1.00 40.69           C
ATOM    633  CD  GLU A  84     -19.504 -47.712  38.947  1.00 46.33           C
ATOM    634  OE1 GLU A  84     -20.707 -47.849  38.608  1.00 46.73           O
ATOM    635  OE2 GLU A  84     -18.989 -46.606  39.249  1.00 45.89           O
ATOM    636  N   MET A  85     -14.003 -48.386  38.785  1.00 30.72           N
ATOM    637  CA  MET A  85     -12.683 -48.082  39.332  1.00 25.48           C
ATOM    638  C   MET A  85     -12.091 -46.765  38.783  1.00 29.58           C
ATOM    639  O   MET A  85     -11.140 -46.238  39.351  1.00 30.73           O
ATOM    640  CB  MET A  85     -11.716 -49.246  39.079  1.00 27.96           C
ATOM    641  CG  MET A  85     -11.437 -49.524  37.581  1.00 27.82           C
ATOM    642  SD  MET A  85     -10.164 -50.784  37.292  1.00 33.83           S
ATOM    643  CE  MET A  85      -8.659 -49.888  37.663  1.00 27.28           C
ATOM    644  N   TRP A  86     -12.660 -46.244  37.692  1.00 26.62           N
ATOM    645  CA  TRP A  86     -12.176 -45.014  37.048  1.00 25.70           C
ATOM    646  C   TRP A  86     -13.030 -43.797  37.384  1.00 24.34           C
ATOM    647  O   TRP A  86     -12.640 -42.661  37.113  1.00 23.76           O
ATOM    648  CB  TRP A  86     -12.137 -45.171  35.518  1.00 24.84           C
ATOM    649  CG  TRP A  86     -11.376 -46.368  35.055  1.00 25.07           C
ATOM    650  CD1 TRP A  86     -11.896 -47.527  34.553  1.00 23.51           C
ATOM    651  CD2 TRP A  86      -9.959 -46.541  35.075  1.00 25.54           C
ATOM    652  NE1 TRP A  86     -10.889 -48.407  34.255  1.00 20.31           N
ATOM    653  CE2 TRP A  86      -9.688 -47.829  34.564  1.00 23.69           C
```

ATOM	654	CE3	TRP	A	86	-8.888	-45.730	35.466	1.00	25.38	C
ATOM	655	CZ2	TRP	A	86	-8.391	-48.321	34.422	1.00	22.04	C
ATOM	656	CZ3	TRP	A	86	-7.600	-46.227	35.343	1.00	26.45	C
ATOM	657	CH2	TRP	A	86	-7.364	-47.511	34.823	1.00	25.01	C
ATOM	658	N	ASN	A	87	-14.209	-44.046	37.940	1.00	24.25	N
ATOM	659	CA	ASN	A	87	-15.158	-42.989	38.269	1.00	22.72	C
ATOM	660	C	ASN	A	87	-14.755	-42.226	39.532	1.00	22.74	C
ATOM	661	O	ASN	A	87	-14.007	-42.748	40.370	1.00	20.93	O
ATOM	662	CB	ASN	A	87	-16.561	-43.585	38.432	1.00	22.74	C
ATOM	663	CG	ASN	A	87	-17.264	-43.825	37.085	1.00	32.22	C
ATOM	664	OD1	ASN	A	87	-16.924	-43.217	36.066	1.00	25.54	O
ATOM	665	ND2	ASN	A	87	-18.258	-44.706	37.090	1.00	29.46	N
ATOM	666	N	PRO	A	88	-15.246	-40.984	39.674	1.00	20.33	N
ATOM	667	CA	PRO	A	88	-14.957	-40.185	40.872	1.00	21.13	C
ATOM	668	C	PRO	A	88	-15.208	-40.980	42.150	1.00	28.46	C
ATOM	669	O	PRO	A	88	-16.250	-41.627	42.271	1.00	27.39	O
ATOM	670	CB	PRO	A	88	-15.954	-39.031	40.766	1.00	20.21	C
ATOM	671	CG	PRO	A	88	-16.189	-38.879	39.291	1.00	18.46	C
ATOM	672	CD	PRO	A	88	-16.079	-40.251	38.700	1.00	20.52	C
ATOM	673	N	ASN	A	89	-14.248	-40.964	43.069	1.00	27.58	N
ATOM	674	CA	ASN	A	89	-14.415	-41.619	44.364	1.00	25.82	C
ATOM	675	C	ASN	A	89	-14.413	-40.608	45.522	1.00	26.22	C
ATOM	676	O	ASN	A	89	-14.214	-40.972	46.679	1.00	28.49	O
ATOM	677	CB	ASN	A	89	-13.332	-42.683	44.577	1.00	22.93	C
ATOM	678	CG	ASN	A	89	-11.947	-42.077	44.753	1.00	28.32	C
ATOM	679	OD1	ASN	A	89	-11.722	-40.911	44.424	1.00	30.26	O
ATOM	680	ND2	ASN	A	89	-11.012	-42.867	45.263	1.00	25.28	N
ATOM	681	N	ARG	A	90	-14.596	-39.336	45.185	1.00	25.13	N
ATOM	682	CA	ARG	A	90	-14.881	-38.278	46.157	1.00	25.19	C
ATOM	683	C	ARG	A	90	-15.925	-37.377	45.521	1.00	25.05	C
ATOM	684	O	ARG	A	90	-16.217	-37.504	44.326	1.00	31.53	O
ATOM	685	CB	ARG	A	90	-13.626	-37.459	46.500	1.00	24.61	C
ATOM	686	CG	ARG	A	90	-12.536	-38.221	47.257	1.00	26.05	C
ATOM	687	CD	ARG	A	90	-13.006	-38.651	48.665	1.00	25.38	C
ATOM	688	NE	ARG	A	90	-11.924	-39.244	49.452	1.00	26.77	N
ATOM	689	CZ	ARG	A	90	-11.481	-40.495	49.307	1.00	33.02	C
ATOM	690	NH1	ARG	A	90	-12.021	-41.297	48.398	1.00	26.91	N
ATOM	691	NH2	ARG	A	90	-10.485	-40.950	50.064	1.00	24.76	N
ATOM	692	N	GLU	A	91	-16.477	-36.455	46.294	1.00	22.20	N
ATOM	693	CA	GLU	A	91	-17.515	-35.573	45.772	1.00	29.88	C
ATOM	694	C	GLU	A	91	-17.000	-34.669	44.646	1.00	32.61	C
ATOM	695	O	GLU	A	91	-15.830	-34.275	44.626	1.00	27.35	O
ATOM	696	CB	GLU	A	91	-18.146	-34.734	46.891	1.00	33.85	C
ATOM	697	CG	GLU	A	91	-17.294	-33.569	47.387	1.00	53.36	C

ATOM	698	CD	GLU	A	91	-18.077	-32.596	48.280	1.00	72.21	C
ATOM	699	OE1	GLU	A	91	-19.113	-33.009	48.853	1.00	69.65	O
ATOM	700	OE2	GLU	A	91	-17.657	-31.418	48.398	1.00	61.11	O
ATOM	701	N	LEU	A	92	-17.882	-34.353	43.704	1.00	30.19	N
ATOM	702	CA	LEU	A	92	-17.536	-33.464	42.608	1.00	27.87	C
ATOM	703	C	LEU	A	92	-17.559	-32.021	43.081	1.00	26.09	C
ATOM	704	O	LEU	A	92	-18.454	-31.614	43.816	1.00	32.14	O
ATOM	705	CB	LEU	A	92	-18.514	-33.626	41.437	1.00	26.29	C
ATOM	706	CG	LEU	A	92	-18.582	-34.960	40.699	1.00	27.83	C
ATOM	707	CD1	LEU	A	92	-19.508	-34.844	39.499	1.00	22.55	C
ATOM	708	CD2	LEU	A	92	-17.204	-35.397	40.264	1.00	27.36	C
ATOM	709	N	SER	A	93	-16.587	-31.236	42.634	1.00	28.50	N
ATOM	710	CA	SER	A	93	-16.592	-29.809	42.917	1.00	27.43	C
ATOM	711	C	SER	A	93	-15.708	-29.052	41.931	1.00	24.88	C
ATOM	712	O	SER	A	93	-14.727	-29.593	41.424	1.00	25.21	O
ATOM	713	CB	SER	A	93	-16.097	-29.556	44.345	1.00	19.41	C
ATOM	714	OG	SER	A	93	-16.065	-28.167	44.608	1.00	23.57	O
ATOM	715	N	GLU	A	94	-16.041	-27.795	41.668	1.00	22.25	N
ATOM	716	CA	GLU	A	94	-15.132	-26.944	40.909	1.00	24.77	C
ATOM	717	C	GLU	A	94	-13.966	-26.481	41.792	1.00	27.92	C
ATOM	718	O	GLU	A	94	-12.936	-26.034	41.296	1.00	28.79	O
ATOM	719	CB	GLU	A	94	-15.875	-25.747	40.324	1.00	20.54	C
ATOM	720	CG	GLU	A	94	-16.754	-26.107	39.129	1.00	27.26	C
ATOM	721	CD	GLU	A	94	-17.412	-24.886	38.521	1.00	30.30	C
ATOM	722	OE1	GLU	A	94	-16.768	-24.231	37.672	1.00	25.85	O
ATOM	723	OE2	GLU	A	94	-18.564	-24.571	38.907	1.00	26.50	O
ATOM	724	N	ASP	A	95	-14.147	-26.591	43.104	1.00	20.63	N
ATOM	725	CA	ASP	A	95	-13.098	-26.291	44.063	1.00	24.68	C
ATOM	726	C	ASP	A	95	-12.327	-27.582	44.178	1.00	25.21	C
ATOM	727	O	ASP	A	95	-12.586	-28.392	45.074	1.00	22.07	O
ATOM	728	CB	ASP	A	95	-13.734	-25.909	45.407	1.00	21.33	C
ATOM	729	CG	ASP	A	95	-12.712	-25.551	46.489	1.00	28.16	C
ATOM	730	OD1	ASP	A	95	-11.490	-25.728	46.302	1.00	27.58	O
ATOM	731	OD2	ASP	A	95	-13.153	-25.086	47.560	1.00	35.93	O
ATOM	732	N	CYS	A	96	-11.393	-27.796	43.256	1.00	23.66	N
ATOM	733	CA	CYS	A	96	-10.767	-29.113	43.135	1.00	22.30	C
ATOM	734	C	CYS	A	96	-9.269	-29.049	42.922	1.00	19.65	C
ATOM	735	O	CYS	A	96	-8.622	-30.075	42.758	1.00	23.97	O
ATOM	736	CB	CYS	A	96	-11.392	-29.885	41.976	1.00	20.41	C
ATOM	737	SG	CYS	A	96	-11.112	-29.104	40.352	1.00	24.70	S
ATOM	738	N	LEU	A	97	-8.711	-27.848	42.921	1.00	22.14	N
ATOM	739	CA	LEU	A	97	-7.297	-27.700	42.619	1.00	21.12	C
ATOM	740	C	LEU	A	97	-6.427	-27.946	43.854	1.00	24.73	C
ATOM	741	O	LEU	A	97	-5.961	-27.016	44.513	1.00	23.74	O

ATOM	742	CB	LEU	A	97	-7.020	-26.348	41.958	1.00	19.83	C
ATOM	743	CG	LEU	A	97	-7.642	-26.211	40.560	1.00	26.04	C
ATOM	744	CD1	LEU	A	97	-7.196	-24.914	39.872	1.00	20.81	C
ATOM	745	CD2	LEU	A	97	-7.341	-27.435	39.655	1.00	17.53	C
ATOM	746	N	TYR	A	98	-6.223	-29.227	44.152	1.00	22.12	N
ATOM	747	CA	TYR	A	98	-5.383	-29.656	45.251	1.00	22.08	C
ATOM	748	C	TYR	A	98	-4.386	-30.662	44.717	1.00	22.84	C
ATOM	749	O	TYR	A	98	-4.616	-31.267	43.671	1.00	22.52	O
ATOM	750	CB	TYR	A	98	-6.239	-30.267	46.374	1.00	23.83	C
ATOM	751	CG	TYR	A	98	-7.255	-29.279	46.895	1.00	26.59	C
ATOM	752	CD1	TYR	A	98	-7.014	-28.550	48.055	1.00	26.95	C
ATOM	753	CD2	TYR	A	98	-8.450	-29.051	46.208	1.00	27.25	C
ATOM	754	CE1	TYR	A	98	-7.939	-27.624	48.527	1.00	22.26	C
ATOM	755	CE2	TYR	A	98	-9.372	-28.125	46.659	1.00	24.41	C
ATOM	756	CZ	TYR	A	98	-9.110	-27.416	47.824	1.00	27.13	C
ATOM	757	OH	TYR	A	98	-10.025	-26.499	48.275	1.00	26.26	O
ATOM	758	N	LEU	A	99	-3.267	-30.824	45.416	1.00	20.46	N
ATOM	759	CA	LEU	A	99	-2.276	-31.823	45.034	1.00	20.90	C
ATOM	760	C	LEU	A	99	-1.889	-32.652	46.264	1.00	23.93	C
ATOM	761	O	LEU	A	99	-2.209	-32.285	47.398	1.00	25.12	O
ATOM	762	CB	LEU	A	99	-1.050	-31.178	44.351	1.00	19.30	C
ATOM	763	CG	LEU	A	99	-0.264	-30.036	45.026	1.00	25.48	C
ATOM	764	CD1	LEU	A	99	0.584	-30.518	46.213	1.00	16.78	C
ATOM	765	CD2	LEU	A	99	0.622	-29.301	44.026	1.00	22.19	C
ATOM	766	N	ASN	A	100	-1.210	-33.769	46.032	1.00	21.81	N
ATOM	767	CA	ASN	A	100	-0.840	-34.681	47.097	1.00	22.95	C
ATOM	768	C	ASN	A	100	0.646	-34.957	47.050	1.00	23.15	C
ATOM	769	O	ASN	A	100	1.223	-35.039	45.974	1.00	24.62	O
ATOM	770	CB	ASN	A	100	-1.596	-35.990	46.943	1.00	18.27	C
ATOM	771	CG	ASN	A	100	-3.021	-35.776	46.531	1.00	25.49	C
ATOM	772	OD1	ASN	A	100	-3.840	-35.304	47.319	1.00	26.37	O
ATOM	773	ND2	ASN	A	100	-3.336	-36.118	45.285	1.00	25.62	N
ATOM	774	N	VAL	A	101	1.265	-35.078	48.220	1.00	24.66	N
ATOM	775	CA	VAL	A	101	2.673	-35.450	48.311	1.00	24.05	C
ATOM	776	C	VAL	A	101	2.805	-36.687	49.208	1.00	28.45	C
ATOM	777	O	VAL	A	101	2.291	-36.703	50.333	1.00	26.83	O
ATOM	778	CB	VAL	A	101	3.519	-34.312	48.930	1.00	23.98	C
ATOM	779	CG1	VAL	A	101	4.999	-34.680	48.944	1.00	20.85	C
ATOM	780	CG2	VAL	A	101	3.296	-33.012	48.194	1.00	21.09	C
ATOM	781	N	TRP	A	102	3.464	-37.721	48.692	1.00	24.56	N
ATOM	782	CA	TRP	A	102	3.835	-38.890	49.482	1.00	24.17	C
ATOM	783	C	TRP	A	102	5.343	-38.871	49.626	1.00	26.87	C
ATOM	784	O	TRP	A	102	6.058	-38.553	48.676	1.00	25.82	O
ATOM	785	CB	TRP	A	102	3.479	-40.196	48.772	1.00	21.44	C

ATOM	786	CG	TRP	A	102	2.033	-40.561	48.736	1.00	25.82	C
ATOM	787	CD1	TRP	A	102	1.366	-41.356	49.622	1.00	25.82	C
ATOM	788	CD2	TRP	A	102	1.080	-40.183	47.739	1.00	23.43	C
ATOM	789	NE1	TRP	A	102	0.054	-41.481	49.247	1.00	28.85	N
ATOM	790	CE2	TRP	A	102	-0.148	-40.770	48.093	1.00	27.92	C
ATOM	791	CE3	TRP	A	102	1.143	-39.395	46.587	1.00	26.59	C
ATOM	792	CZ2	TRP	A	102	-1.305	-40.596	47.333	1.00	25.40	C
ATOM	793	CZ3	TRP	A	102	-0.007	-39.228	45.831	1.00	22.89	C
ATOM	794	CH2	TRP	A	102	-1.211	-39.827	46.207	1.00	22.77	C
ATOM	795	N	THR	A	103	5.831	-39.237	50.804	1.00	27.75	N
ATOM	796	CA	THR	A	103	7.265	-39.237	51.043	1.00	32.55	C
ATOM	797	C	THR	A	103	7.603	-40.358	52.027	1.00	30.46	C
ATOM	798	O	THR	A	103	6.726	-40.814	52.763	1.00	31.90	O
ATOM	799	CB	THR	A	103	7.727	-37.842	51.545	1.00	34.98	C
ATOM	800	OG1	THR	A	103	9.125	-37.675	51.294	1.00	48.36	O
ATOM	801	CG2	THR	A	103	7.448	-37.669	53.018	1.00	28.61	C
ATOM	802	N	PRO	A	104	8.849	-40.855	51.997	1.00	28.45	N
ATOM	803	CA	PRO	A	104	9.242	-41.880	52.972	1.00	28.87	C
ATOM	804	C	PRO	A	104	9.040	-41.441	54.427	1.00	36.11	C
ATOM	805	O	PRO	A	104	8.895	-40.247	54.712	1.00	36.58	O
ATOM	806	CB	PRO	A	104	10.729	-42.080	52.680	1.00	33.70	C
ATOM	807	CG	PRO	A	104	10.855	-41.776	51.215	1.00	28.71	C
ATOM	808	CD	PRO	A	104	9.895	-40.634	50.977	1.00	27.90	C
ATOM	809	N	TYR	A	105	8.986	-42.416	55.330	1.00	33.82	N
ATOM	810	CA	TYR	A	105	8.867	-42.151	56.761	1.00	36.45	C
ATOM	811	C	TYR	A	105	10.060	-42.775	57.481	1.00	42.67	C
ATOM	812	O	TYR	A	105	10.351	-43.962	57.298	1.00	35.72	O
ATOM	813	CB	TYR	A	105	7.561	-42.707	57.324	1.00	34.13	C
ATOM	814	CG	TYR	A	105	7.257	-42.273	58.746	1.00	37.23	C
ATOM	815	CD1	TYR	A	105	7.881	-42.883	59.841	1.00	39.92	C
ATOM	816	CD2	TYR	A	105	6.329	-41.265	59.000	1.00	41.58	C
ATOM	817	CE1	TYR	A	105	7.591	-42.489	61.152	1.00	33.33	C
ATOM	818	CE2	TYR	A	105	6.034	-40.863	60.299	1.00	36.54	C
ATOM	819	CZ	TYR	A	105	6.668	-41.479	61.370	1.00	40.45	C
ATOM	820	OH	TYR	A	105	6.375	-41.075	62.656	1.00	42.50	O
ATOM	821	N	PRO	A	106	10.766	-41.973	58.292	1.00	45.43	N
ATOM	822	CA	PRO	A	106	10.477	-40.550	58.489	1.00	45.26	C
ATOM	823	C	PRO	A	106	10.917	-39.757	57.256	1.00	42.85	C
ATOM	824	O	PRO	A	106	11.648	-40.317	56.431	1.00	38.81	O
ATOM	825	CB	PRO	A	106	11.347	-40.194	59.701	1.00	40.79	C
ATOM	826	CG	PRO	A	106	12.524	-41.109	59.581	1.00	38.96	C
ATOM	827	CD	PRO	A	106	11.960	-42.405	59.046	1.00	39.69	C
ATOM	828	N	ARG	A	107	10.464	-38.509	57.124	1.00	44.03	N
ATOM	829	CA	ARG	A	107	10.788	-37.686	55.957	1.00	43.51	C

```
ATOM    830  C   ARG A 107      12.267 -37.737  55.631  1.00 38.29           C
ATOM    831  O   ARG A 107      13.100 -37.692  56.527  1.00 52.90           O
ATOM    832  CB  ARG A 107      10.368 -36.227  56.166  1.00 42.66           C
ATOM    833  CG  ARG A 107       8.866 -36.014  56.289  1.00 46.41           C
ATOM    834  CD  ARG A 107       8.449 -34.700  55.627  1.00 48.08           C
ATOM    835  NE  ARG A 107       9.174 -33.536  56.151  1.00 43.80           N
ATOM    836  CZ  ARG A 107       8.753 -32.790  57.171  1.00 44.36           C
ATOM    837  NH1 ARG A 107       7.619 -33.086  57.799  1.00 36.87           N
ATOM    838  NH2 ARG A 107       9.469 -31.748  57.575  1.00 54.45           N
ATOM    839  N   PRO A 108      12.595 -37.849  54.343  1.00 43.44           N
ATOM    840  CA  PRO A 108      13.998 -37.835  53.926  1.00 46.55           C
ATOM    841  C   PRO A 108      14.636 -36.540  54.394  1.00 53.32           C
ATOM    842  O   PRO A 108      14.001 -35.484  54.334  1.00 49.91           O
ATOM    843  CB  PRO A 108      13.914 -37.846  52.397  1.00 42.34           C
ATOM    844  CG  PRO A 108      12.569 -38.406  52.089  1.00 39.04           C
ATOM    845  CD  PRO A 108      11.668 -37.987  53.207  1.00 39.43           C
ATOM    846  N   THR A 109      15.868 -36.618  54.877  1.00 58.25           N
ATOM    847  CA  THR A 109      16.555 -35.419  55.332  1.00 58.20           C
ATOM    848  C   THR A 109      17.589 -34.994  54.309  1.00 55.07           C
ATOM    849  O   THR A 109      18.404 -34.121  54.579  1.00 61.22           O
ATOM    850  CB  THR A 109      17.214 -35.606  56.720  1.00 51.78           C
ATOM    851  OG1 THR A 109      18.173 -36.672  56.670  1.00 52.58           O
ATOM    852  CG2 THR A 109      16.166 -35.902  57.783  1.00 46.11           C
ATOM    853  N   SER A 110      17.549 -35.621  53.135  1.00 51.00           N
ATOM    854  CA  SER A 110      18.417 -35.241  52.020  1.00 50.89           C
ATOM    855  C   SER A 110      17.648 -35.340  50.695  1.00 45.74           C
ATOM    856  O   SER A 110      16.722 -36.142  50.584  1.00 42.20           O
ATOM    857  CB  SER A 110      19.679 -36.110  52.004  1.00 45.77           C
ATOM    858  OG  SER A 110      19.348 -37.484  52.032  1.00 45.96           O
ATOM    859  N   PRO A 111      18.018 -34.515  49.698  1.00 44.51           N
ATOM    860  CA  PRO A 111      17.301 -34.414  48.415  1.00 39.67           C
ATOM    861  C   PRO A 111      16.971 -35.763  47.777  1.00 45.46           C
ATOM    862  O   PRO A 111      17.866 -36.512  47.381  1.00 46.20           O
ATOM    863  CB  PRO A 111      18.275 -33.627  47.537  1.00 35.63           C
ATOM    864  CG  PRO A 111      18.974 -32.732  48.505  1.00 42.97           C
ATOM    865  CD  PRO A 111      19.127 -33.548  49.771  1.00 40.34           C
ATOM    866  N   THR A 112      15.675 -36.053  47.698  1.00 41.07           N
ATOM    867  CA  THR A 112      15.157 -37.289  47.122  1.00 39.85           C
ATOM    868  C   THR A 112      14.520 -37.009  45.747  1.00 39.96           C
ATOM    869  O   THR A 112      13.786 -36.029  45.597  1.00 32.73           O
ATOM    870  CB  THR A 112      14.096 -37.867  48.063  1.00 38.25           C
ATOM    871  OG1 THR A 112      14.661 -38.023  49.371  1.00 42.56           O
ATOM    872  CG2 THR A 112      13.581 -39.202  47.569  1.00 36.47           C
ATOM    873  N   PRO A 113      14.805 -37.862  44.741  1.00 42.47           N
```

ATOM	874	CA	PRO	A	113	14.216	-37.720	43.395	1.00	33.41	C
ATOM	875	C	PRO	A	113	12.693	-37.738	43.416	1.00	30.49	C
ATOM	876	O	PRO	A	113	12.092	-38.541	44.133	1.00	29.75	O
ATOM	877	CB	PRO	A	113	14.741	-38.948	42.637	1.00	31.72	C
ATOM	878	CG	PRO	A	113	15.209	-39.902	43.698	1.00	40.75	C
ATOM	879	CD	PRO	A	113	15.703	-39.027	44.819	1.00	46.30	C
ATOM	880	N	VAL	A	114	12.090	-36.853	42.629	1.00	28.08	N
ATOM	881	CA	VAL	A	114	10.646	-36.648	42.631	1.00	28.49	C
ATOM	882	C	VAL	A	114	9.967	-37.217	41.380	1.00	26.63	C
ATOM	883	O	VAL	A	114	10.411	-36.978	40.256	1.00	29.38	O
ATOM	884	CB	VAL	A	114	10.323	-35.142	42.709	1.00	29.32	C
ATOM	885	CG1	VAL	A	114	8.823	-34.909	42.703	1.00	20.87	C
ATOM	886	CG2	VAL	A	114	10.973	-34.525	43.950	1.00	27.84	C
ATOM	887	N	LEU	A	115	8.900	-37.979	41.590	1.00	28.70	N
ATOM	888	CA	LEU	A	115	8.016	-38.416	40.511	1.00	25.93	C
ATOM	889	C	LEU	A	115	6.714	-37.631	40.591	1.00	26.36	C
ATOM	890	O	LEU	A	115	6.109	-37.530	41.669	1.00	24.66	O
ATOM	891	CB	LEU	A	115	7.717	-39.911	40.631	1.00	24.33	C
ATOM	892	CG	LEU	A	115	8.777	-40.849	40.051	1.00	27.85	C
ATOM	893	CD1	LEU	A	115	8.684	-42.221	40.677	1.00	32.07	C
ATOM	894	CD2	LEU	A	115	8.606	-40.940	38.538	1.00	30.14	C
ATOM	895	N	VAL	A	116	6.290	-37.064	39.465	1.00	18.56	N
ATOM	896	CA	VAL	A	116	5.025	-36.338	39.425	1.00	22.75	C
ATOM	897	C	VAL	A	116	4.033	-37.030	38.505	1.00	22.84	C
ATOM	898	O	VAL	A	116	4.291	-37.192	37.315	1.00	22.85	O
ATOM	899	CB	VAL	A	116	5.203	-34.881	38.966	1.00	20.98	C
ATOM	900	CG1	VAL	A	116	3.868	-34.155	39.033	1.00	17.02	C
ATOM	901	CG2	VAL	A	116	6.238	-34.186	39.828	1.00	21.88	C
ATOM	902	N	TRP	A	117	2.906	-37.448	39.064	1.00	21.82	N
ATOM	903	CA	TRP	A	117	1.904	-38.192	38.309	1.00	22.55	C
ATOM	904	C	TRP	A	117	0.821	-37.271	37.735	1.00	21.27	C
ATOM	905	O	TRP	A	117	0.228	-36.470	38.459	1.00	20.97	O
ATOM	906	CB	TRP	A	117	1.253	-39.269	39.197	1.00	20.20	C
ATOM	907	CG	TRP	A	117	0.116	-39.996	38.505	1.00	24.63	C
ATOM	908	CD1	TRP	A	117	-1.218	-39.871	38.763	1.00	22.18	C
ATOM	909	CD2	TRP	A	117	0.226	-40.928	37.415	1.00	21.67	C
ATOM	910	NE1	TRP	A	117	-1.945	-40.687	37.922	1.00	19.48	N
ATOM	911	CE2	TRP	A	117	-1.084	-41.342	37.084	1.00	21.48	C
ATOM	912	CE3	TRP	A	117	1.305	-41.462	36.695	1.00	22.53	C
ATOM	913	CZ2	TRP	A	117	-1.345	-42.262	36.059	1.00	21.31	C
ATOM	914	CZ3	TRP	A	117	1.042	-42.378	35.677	1.00	20.30	C
ATOM	915	CH2	TRP	A	117	-0.271	-42.767	35.374	1.00	19.23	C
ATOM	916	N	ILE	A	118	0.559	-37.397	36.438	1.00	20.06	N
ATOM	917	CA	ILE	A	118	-0.572	-36.714	35.807	1.00	19.74	C

```
ATOM    918  C   ILE A 118      -1.555 -37.755  35.270  1.00 20.69           C
ATOM    919  O   ILE A 118      -1.217 -38.500  34.353  1.00 22.31           O
ATOM    920  CB  ILE A 118      -0.110 -35.815  34.647  1.00 17.47           C
ATOM    921  CG1 ILE A 118       0.950 -34.821  35.131  1.00 25.61           C
ATOM    922  CG2 ILE A 118      -1.282 -35.076  34.039  1.00 15.42           C
ATOM    923  CD1 ILE A 118       1.513 -33.943  34.015  1.00 19.12           C
ATOM    924  N   TYR A 119      -2.764 -37.813  35.825  1.00 17.61           N
ATOM    925  CA  TYR A 119      -3.713 -38.855  35.425  1.00 18.62           C
ATOM    926  C   TYR A 119      -4.248 -38.619  34.026  1.00 20.26           C
ATOM    927  O   TYR A 119      -4.233 -37.493  33.533  1.00 18.75           O
ATOM    928  CB  TYR A 119      -4.896 -38.974  36.406  1.00 18.16           C
ATOM    929  CG  TYR A 119      -5.661 -37.679  36.665  1.00 21.50           C
ATOM    930  CD1 TYR A 119      -6.560 -37.158  35.727  1.00 18.60           C
ATOM    931  CD2 TYR A 119      -5.496 -36.988  37.866  1.00 17.24           C
ATOM    932  CE1 TYR A 119      -7.256 -35.965  35.976  1.00 15.59           C
ATOM    933  CE2 TYR A 119      -6.179 -35.809  38.121  1.00 19.43           C
ATOM    934  CZ  TYR A 119      -7.055 -35.294  37.184  1.00 21.42           C
ATOM    935  OH  TYR A 119      -7.722 -34.112  37.481  1.00 18.69           O
ATOM    936  N   GLY A 120      -4.743 -39.690  33.407  1.00 21.49           N
ATOM    937  CA  GLY A 120      -5.453 -39.607  32.142  1.00 19.47           C
ATOM    938  C   GLY A 120      -6.956 -39.647  32.367  1.00 22.29           C
ATOM    939  O   GLY A 120      -7.442 -39.397  33.474  1.00 21.22           O
ATOM    940  N   GLY A 121      -7.696 -39.975  31.317  1.00 21.32           N
ATOM    941  CA  GLY A 121      -9.143 -39.882  31.346  1.00 17.84           C
ATOM    942  C   GLY A 121      -9.629 -38.975  30.230  1.00 20.78           C
ATOM    943  O   GLY A 121     -10.609 -38.242  30.404  1.00 21.02           O
ATOM    944  N   GLY A 122      -8.926 -39.020  29.092  1.00 21.45           N
ATOM    945  CA  GLY A 122      -9.303 -38.289  27.887  1.00 21.75           C
ATOM    946  C   GLY A 122      -9.485 -36.782  27.999  1.00 19.32           C
ATOM    947  O   GLY A 122     -10.146 -36.180  27.157  1.00 20.07           O
ATOM    948  N   PHE A 123      -8.903 -36.170  29.032  1.00 19.52           N
ATOM    949  CA  PHE A 123      -9.117 -34.742  29.335  1.00 21.64           C
ATOM    950  C   PHE A 123     -10.551 -34.398  29.771  1.00 21.47           C
ATOM    951  O   PHE A 123     -10.869 -33.221  29.967  1.00 21.65           O
ATOM    952  CB  PHE A 123      -8.702 -33.824  28.162  1.00 19.28           C
ATOM    953  CG  PHE A 123      -7.232 -33.847  27.854  1.00 22.10           C
ATOM    954  CD1 PHE A 123      -6.309 -33.322  28.755  1.00 18.30           C
ATOM    955  CD2 PHE A 123      -6.768 -34.381  26.652  1.00 19.91           C
ATOM    956  CE1 PHE A 123      -4.945 -33.338  28.468  1.00 24.91           C
ATOM    957  CE2 PHE A 123      -5.399 -34.395  26.353  1.00 19.95           C
ATOM    958  CZ  PHE A 123      -4.490 -33.872  27.259  1.00 19.79           C
ATOM    959  N   TYR A 124     -11.412 -35.407  29.910  1.00 18.10           N
ATOM    960  CA  TYR A 124     -12.791 -35.177  30.356  1.00 21.68           C
ATOM    961  C   TYR A 124     -13.015 -35.740  31.769  1.00 22.16           C
```

ATOM	962	O	TYR	A	124	-14.054	-35.510	32.387	1.00	25.93	O
ATOM	963	CB	TYR	A	124	-13.816	-35.787	29.374	1.00	19.93	C
ATOM	964	CG	TYR	A	124	-13.866	-37.304	29.401	1.00	18.22	C
ATOM	965	CD1	TYR	A	124	-14.653	-37.983	30.328	1.00	21.15	C
ATOM	966	CD2	TYR	A	124	-13.118	-38.058	28.508	1.00	21.37	C
ATOM	967	CE1	TYR	A	124	-14.677	-39.365	30.377	1.00	18.91	C
ATOM	968	CE2	TYR	A	124	-13.146	-39.439	28.542	1.00	20.86	C
ATOM	969	CZ	TYR	A	124	-13.927	-40.086	29.482	1.00	20.72	C
ATOM	970	OH	TYR	A	124	-13.949	-41.461	29.515	1.00	25.00	O
ATOM	971	N	SER	A	125	-12.057	-36.504	32.270	1.00	19.62	N
ATOM	972	CA	SER	A	125	-12.240	-37.150	33.560	1.00	21.00	C
ATOM	973	C	SER	A	125	-10.912	-37.485	34.223	1.00	19.97	C
ATOM	974	O	SER	A	125	-9.848	-37.298	33.636	1.00	22.76	O
ATOM	975	CB	SER	A	125	-13.029	-38.444	33.374	1.00	20.59	C
ATOM	976	OG	SER	A	125	-12.295	-39.369	32.583	1.00	19.56	O
ATOM	977	N	GLY	A	126	-10.998	-38.024	35.436	1.00	18.24	N
ATOM	978	CA	GLY	A	126	-9.846	-38.504	36.170	1.00	19.93	C
ATOM	979	C	GLY	A	126	-9.766	-37.803	37.506	1.00	20.03	C
ATOM	980	O	GLY	A	126	-10.450	-36.807	37.724	1.00	21.13	O
ATOM	981	N	ALA	A	127	-8.933	-38.328	38.395	1.00	21.21	N
ATOM	982	CA	ALA	A	127	-8.707	-37.731	39.714	1.00	24.49	C
ATOM	983	C	ALA	A	127	-7.443	-38.324	40.313	1.00	24.63	C
ATOM	984	O	ALA	A	127	-7.107	-39.478	40.031	1.00	23.57	O
ATOM	985	CB	ALA	A	127	-9.893	-37.986	40.641	1.00	20.04	C
ATOM	986	N	SER	A	128	-6.742	-37.542	41.136	1.00	23.24	N
ATOM	987	CA	SER	A	128	-5.556	-38.040	41.824	1.00	20.64	C
ATOM	988	C	SER	A	128	-5.915	-38.997	42.966	1.00	24.81	C
ATOM	989	O	SER	A	128	-5.056	-39.738	43.460	1.00	21.61	O
ATOM	990	CB	SER	A	128	-4.709	-36.882	42.358	1.00	25.61	C
ATOM	991	OG	SER	A	128	-5.448	-36.077	43.267	1.00	28.47	O
ATOM	992	N	SER	A	129	-7.183	-38.987	43.372	1.00	20.56	N
ATOM	993	CA	SER	A	129	-7.629	-39.772	44.526	1.00	23.61	C
ATOM	994	C	SER	A	129	-7.990	-41.220	44.202	1.00	26.44	C
ATOM	995	O	SER	A	129	-8.331	-41.986	45.101	1.00	24.53	O
ATOM	996	CB	SER	A	129	-8.833	-39.107	45.181	1.00	21.54	C
ATOM	997	OG	SER	A	129	-9.849	-38.847	44.232	1.00	22.74	O
ATOM	998	N	LEU	A	130	-7.925	-41.594	42.927	1.00	24.76	N
ATOM	999	CA	LEU	A	130	-8.286	-42.952	42.527	1.00	24.77	C
ATOM	1000	C	LEU	A	130	-7.377	-43.984	43.188	1.00	25.28	C
ATOM	1001	O	LEU	A	130	-6.176	-43.748	43.353	1.00	25.31	O
ATOM	1002	CB	LEU	A	130	-8.243	-43.105	41.001	1.00	22.42	C
ATOM	1003	CG	LEU	A	130	-9.219	-42.223	40.211	1.00	28.16	C
ATOM	1004	CD1	LEU	A	130	-9.338	-42.651	38.725	1.00	19.46	C
ATOM	1005	CD2	LEU	A	130	-10.589	-42.201	40.895	1.00	24.15	C

```
ATOM   1006  N   ASP A 131      -7.959 -45.123  43.556  1.00 20.78           N
ATOM   1007  CA  ASP A 131      -7.232 -46.208  44.216  1.00 24.01           C
ATOM   1008  C   ASP A 131      -6.008 -46.645  43.425  1.00 25.55           C
ATOM   1009  O   ASP A 131      -4.954 -46.947  43.983  1.00 26.37           O
ATOM   1010  CB  ASP A 131      -8.147 -47.428  44.380  1.00 22.32           C
ATOM   1011  CG  ASP A 131      -9.060 -47.327  45.582  1.00 32.16           C
ATOM   1012  OD1 ASP A 131      -9.202 -46.221  46.161  1.00 27.06           O
ATOM   1013  OD2 ASP A 131      -9.643 -48.372  45.945  1.00 37.49           O
ATOM   1014  N   VAL A 132      -6.164 -46.706  42.112  1.00 23.20           N
ATOM   1015  CA  VAL A 132      -5.120 -47.242  41.263  1.00 23.96           C
ATOM   1016  C   VAL A 132      -3.898 -46.296  41.186  1.00 22.91           C
ATOM   1017  O   VAL A 132      -2.830 -46.696  40.737  1.00 23.80           O
ATOM   1018  CB  VAL A 132      -5.706 -47.610  39.875  1.00 27.08           C
ATOM   1019  CG1 VAL A 132      -6.107 -46.366  39.111  1.00 26.96           C
ATOM   1020  CG2 VAL A 132      -4.742 -48.440  39.080  1.00 30.55           C
ATOM   1021  N   TYR A 133      -4.054 -45.057  41.659  1.00 21.79           N
ATOM   1022  CA  TYR A 133      -2.953 -44.084  41.669  1.00 24.06           C
ATOM   1023  C   TYR A 133      -2.334 -43.870  43.064  1.00 26.48           C
ATOM   1024  O   TYR A 133      -1.618 -42.886  43.274  1.00 22.34           O
ATOM   1025  CB  TYR A 133      -3.408 -42.717  41.143  1.00 16.41           C
ATOM   1026  CG  TYR A 133      -4.092 -42.731  39.793  1.00 25.76           C
ATOM   1027  CD1 TYR A 133      -3.774 -43.689  38.840  1.00 18.04           C
ATOM   1028  CD2 TYR A 133      -5.062 -41.781  39.477  1.00 19.49           C
ATOM   1029  CE1 TYR A 133      -4.386 -43.696  37.622  1.00 22.10           C
ATOM   1030  CE2 TYR A 133      -5.686 -41.785  38.268  1.00 19.55           C
ATOM   1031  CZ  TYR A 133      -5.340 -42.747  37.333  1.00 24.09           C
ATOM   1032  OH  TYR A 133      -5.953 -42.767  36.107  1.00 17.97           O
ATOM   1033  N   ASP A 134      -2.615 -44.772  44.003  1.00 23.28           N
ATOM   1034  CA  ASP A 134      -2.073 -44.687  45.362  1.00 23.73           C
ATOM   1035  C   ASP A 134      -0.539 -44.685  45.371  1.00 23.54           C
ATOM   1036  O   ASP A 134       0.098 -45.678  45.029  1.00 21.34           O
ATOM   1037  CB  ASP A 134      -2.607 -45.847  46.205  1.00 25.23           C
ATOM   1038  CG  ASP A 134      -2.283 -45.705  47.693  1.00 33.25           C
ATOM   1039  OD1 ASP A 134      -1.488 -44.816  48.067  1.00 30.91           O
ATOM   1040  OD2 ASP A 134      -2.825 -46.498  48.494  1.00 35.15           O
ATOM   1041  N   GLY A 135       0.060 -43.574  45.788  1.00 22.67           N
ATOM   1042  CA  GLY A 135       1.511 -43.468  45.757  1.00 26.18           C
ATOM   1043  C   GLY A 135       2.289 -44.194  46.853  1.00 31.25           C
ATOM   1044  O   GLY A 135       3.527 -44.251  46.802  1.00 29.18           O
ATOM   1045  N   ARG A 136       1.593 -44.758  47.840  1.00 26.26           N
ATOM   1046  CA  ARG A 136       2.283 -45.274  49.029  1.00 28.24           C
ATOM   1047  C   ARG A 136       3.304 -46.387  48.739  1.00 28.73           C
ATOM   1048  O   ARG A 136       4.342 -46.457  49.396  1.00 29.29           O
ATOM   1049  CB  ARG A 136       1.282 -45.738  50.089  1.00 27.22           C
```

```
ATOM   1050  CG  ARG A 136       0.788 -47.160  49.871  1.00 27.24           C
ATOM   1051  CD  ARG A 136      -0.184 -47.541  50.948  1.00 29.02           C
ATOM   1052  NE  ARG A 136      -1.386 -46.728  50.861  1.00 24.65           N
ATOM   1053  CZ  ARG A 136      -2.252 -46.591  51.852  1.00 33.82           C
ATOM   1054  NH1 ARG A 136      -2.030 -47.203  53.018  1.00 25.93           N
ATOM   1055  NH2 ARG A 136      -3.323 -45.832  51.683  1.00 25.70           N
ATOM   1056  N   PHE A 137       3.036 -47.241  47.753  1.00 25.48           N
ATOM   1057  CA  PHE A 137       3.912 -48.397  47.512  1.00 22.79           C
ATOM   1058  C   PHE A 137       5.217 -48.020  46.804  1.00 30.12           C
ATOM   1059  O   PHE A 137       6.271 -48.601  47.085  1.00 26.81           O
ATOM   1060  CB  PHE A 137       3.176 -49.508  46.743  1.00 24.61           C
ATOM   1061  CG  PHE A 137       1.874 -49.916  47.376  1.00 28.63           C
ATOM   1062  CD1 PHE A 137       0.668 -49.452  46.877  1.00 22.95           C
ATOM   1063  CD2 PHE A 137       1.859 -50.733  48.500  1.00 29.02           C
ATOM   1064  CE1 PHE A 137      -0.535 -49.810  47.479  1.00 29.01           C
ATOM   1065  CE2 PHE A 137       0.661 -51.093  49.112  1.00 27.77           C
ATOM   1066  CZ  PHE A 137      -0.534 -50.633  48.605  1.00 23.62           C
ATOM   1067  N   LEU A 138       5.140 -47.052  45.890  1.00 29.14           N
ATOM   1068  CA  LEU A 138       6.307 -46.622  45.121  1.00 27.70           C
ATOM   1069  C   LEU A 138       7.262 -45.881  46.034  1.00 24.34           C
ATOM   1070  O   LEU A 138       8.483 -46.032  45.967  1.00 23.16           O
ATOM   1071  CB  LEU A 138       5.885 -45.688  43.980  1.00 22.29           C
ATOM   1072  CG  LEU A 138       5.911 -46.248  42.566  1.00 28.58           C
ATOM   1073  CD1 LEU A 138       5.152 -45.309  41.634  1.00 30.97           C
ATOM   1074  CD2 LEU A 138       7.345 -46.429  42.100  1.00 26.56           C
ATOM   1075  N   VAL A 139       6.685 -45.046  46.878  1.00 21.58           N
ATOM   1076  CA  VAL A 139       7.477 -44.249  47.778  1.00 25.63           C
ATOM   1077  C   VAL A 139       8.220 -45.146  48.764  1.00 28.74           C
ATOM   1078  O   VAL A 139       9.421 -44.982  48.968  1.00 29.24           O
ATOM   1079  CB  VAL A 139       6.598 -43.235  48.495  1.00 32.22           C
ATOM   1080  CG1 VAL A 139       7.354 -42.597  49.603  1.00 28.29           C
ATOM   1081  CG2 VAL A 139       6.123 -42.187  47.496  1.00 27.28           C
ATOM   1082  N   GLN A 140       7.519 -46.119  49.341  1.00 31.83           N
ATOM   1083  CA  GLN A 140       8.149 -47.049  50.275  1.00 29.83           C
ATOM   1084  C   GLN A 140       9.234 -47.856  49.575  1.00 29.82           C
ATOM   1085  O   GLN A 140      10.373 -47.885  50.029  1.00 32.02           O
ATOM   1086  CB  GLN A 140       7.112 -47.984  50.915  1.00 30.02           C
ATOM   1087  CG  GLN A 140       7.581 -48.693  52.191  1.00 29.43           C
ATOM   1088  CD  GLN A 140       8.521 -49.856  51.914  1.00 33.84           C
ATOM   1089  OE1 GLN A 140       8.305 -50.643  50.988  1.00 33.49           O
ATOM   1090  NE2 GLN A 140       9.578 -49.963  52.708  1.00 30.37           N
ATOM   1091  N   ALA A 141       8.880 -48.493  48.461  1.00 29.27           N
ATOM   1092  CA  ALA A 141       9.766 -49.464  47.822  1.00 30.81           C
ATOM   1093  C   ALA A 141      10.978 -48.841  47.145  1.00 30.50           C
```

```
ATOM   1094  O   ALA A 141      12.050 -49.446  47.085  1.00 30.33           O
ATOM   1095  CB  ALA A 141       8.984 -50.322  46.811  1.00 29.65           C
ATOM   1096  N   GLU A 142      10.810 -47.639  46.613  1.00 26.21           N
ATOM   1097  CA  GLU A 142      11.848 -47.079  45.772  1.00 25.36           C
ATOM   1098  C   GLU A 142      12.414 -45.791  46.333  1.00 28.97           C
ATOM   1099  O   GLU A 142      13.342 -45.212  45.758  1.00 24.98           O
ATOM   1100  CB  GLU A 142      11.338 -46.888  44.340  1.00 25.20           C
ATOM   1101  CG  GLU A 142      11.117 -48.200  43.613  1.00 27.04           C
ATOM   1102  CD  GLU A 142      12.392 -49.013  43.495  1.00 31.40           C
ATOM   1103  OE1 GLU A 142      13.473 -48.395  43.383  1.00 32.05           O
ATOM   1104  OE2 GLU A 142      12.320 -50.264  43.518  1.00 32.37           O
ATOM   1105  N   ARG A 143      11.857 -45.364  47.464  1.00 31.35           N
ATOM   1106  CA  ARG A 143      12.354 -44.204  48.202  1.00 31.83           C
ATOM   1107  C   ARG A 143      12.453 -42.952  47.353  1.00 29.37           C
ATOM   1108  O   ARG A 143      13.489 -42.312  47.273  1.00 35.35           O
ATOM   1109  CB  ARG A 143      13.660 -44.536  48.926  1.00 28.58           C
ATOM   1110  CG  ARG A 143      13.414 -45.552  50.039  1.00 35.03           C
ATOM   1111  CD  ARG A 143      14.567 -45.674  51.009  1.00 38.75           C
ATOM   1112  NE  ARG A 143      14.739 -44.482  51.836  1.00 46.42           N
ATOM   1113  CZ  ARG A 143      14.059 -44.239  52.954  1.00 45.47           C
ATOM   1114  NH1 ARG A 143      13.140 -45.098  53.381  1.00 32.13           N
ATOM   1115  NH2 ARG A 143      14.287 -43.127  53.639  1.00 46.82           N
ATOM   1116  N   THR A 144      11.344 -42.631  46.705  1.00 32.41           N
ATOM   1117  CA  THR A 144      11.202 -41.397  45.963  1.00 30.30           C
ATOM   1118  C   THR A 144      10.203 -40.522  46.698  1.00 27.99           C
ATOM   1119  O   THR A 144       9.533 -40.975  47.632  1.00 27.84           O
ATOM   1120  CB  THR A 144      10.597 -41.668  44.583  1.00 35.21           C
ATOM   1121  OG1 THR A 144       9.446 -42.511  44.744  1.00 38.23           O
ATOM   1122  CG2 THR A 144      11.603 -42.346  43.673  1.00 33.98           C
ATOM   1123  N   VAL A 145      10.100 -39.271  46.273  1.00 23.22           N
ATOM   1124  CA  VAL A 145       8.945 -38.454  46.618  1.00 29.04           C
ATOM   1125  C   VAL A 145       7.980 -38.472  45.423  1.00 32.40           C
ATOM   1126  O   VAL A 145       8.406 -38.376  44.263  1.00 26.72           O
ATOM   1127  CB  VAL A 145       9.366 -37.029  46.972  1.00 27.45           C
ATOM   1128  CG1 VAL A 145       8.156 -36.103  47.069  1.00 28.13           C
ATOM   1129  CG2 VAL A 145      10.157 -37.042  48.273  1.00 31.38           C
ATOM   1130  N   LEU A 146       6.690 -38.646  45.708  1.00 26.51           N
ATOM   1131  CA  LEU A 146       5.676 -38.760  44.666  1.00 26.15           C
ATOM   1132  C   LEU A 146       4.682 -37.642  44.858  1.00 26.71           C
ATOM   1133  O   LEU A 146       4.152 -37.457  45.959  1.00 29.11           O
ATOM   1134  CB  LEU A 146       4.959 -40.115  44.749  1.00 26.19           C
ATOM   1135  CG  LEU A 146       4.000 -40.630  43.655  1.00 32.46           C
ATOM   1136  CD1 LEU A 146       2.655 -39.907  43.594  1.00 31.30           C
ATOM   1137  CD2 LEU A 146       4.652 -40.560  42.320  1.00 28.97           C
```

```
ATOM   1138  N   VAL A 147       4.429 -36.901  43.784  1.00 25.66           N
ATOM   1139  CA  VAL A 147       3.442 -35.828  43.799  1.00 24.44           C
ATOM   1140  C   VAL A 147       2.400 -36.105  42.725  1.00 23.48           C
ATOM   1141  O   VAL A 147       2.738 -36.539  41.625  1.00 22.06           O
ATOM   1142  CB  VAL A 147       4.102 -34.453  43.513  1.00 23.14           C
ATOM   1143  CG1 VAL A 147       3.066 -33.324  43.556  1.00 18.11           C
ATOM   1144  CG2 VAL A 147       5.232 -34.186  44.500  1.00 22.93           C
ATOM   1145  N   SER A 148       1.132 -35.862  43.038  1.00 22.93           N
ATOM   1146  CA  SER A 148       0.098 -35.901  42.011  1.00 25.29           C
ATOM   1147  C   SER A 148      -0.871 -34.752  42.235  1.00 24.52           C
ATOM   1148  O   SER A 148      -1.132 -34.372  43.380  1.00 21.58           O
ATOM   1149  CB  SER A 148      -0.642 -37.239  42.032  1.00 19.94           C
ATOM   1150  OG  SER A 148      -1.368 -37.388  43.228  1.00 22.02           O
ATOM   1151  N   MET A 149      -1.405 -34.195  41.149  1.00 18.38           N
ATOM   1152  CA  MET A 149      -2.322 -33.072  41.277  1.00 23.28           C
ATOM   1153  C   MET A 149      -3.648 -33.360  40.593  1.00 25.41           C
ATOM   1154  O   MET A 149      -3.714 -34.201  39.705  1.00 20.70           O
ATOM   1155  CB  MET A 149      -1.707 -31.792  40.693  1.00 20.59           C
ATOM   1156  CG  MET A 149      -1.941 -31.610  39.178  1.00 19.86           C
ATOM   1157  SD  MET A 149      -1.074 -32.876  38.213  1.00 23.30           S
ATOM   1158  CE  MET A 149       0.637 -32.422  38.502  1.00 17.15           C
ATOM   1159  N   ASN A 150      -4.701 -32.668  41.027  1.00 20.00           N
ATOM   1160  CA  ASN A 150      -5.922 -32.599  40.250  1.00 23.24           C
ATOM   1161  C   ASN A 150      -5.824 -31.413  39.297  1.00 24.90           C
ATOM   1162  O   ASN A 150      -5.271 -30.377  39.646  1.00 26.11           O
ATOM   1163  CB  ASN A 150      -7.154 -32.453  41.151  1.00 20.18           C
ATOM   1164  CG  ASN A 150      -7.679 -33.794  41.656  1.00 28.81           C
ATOM   1165  OD1 ASN A 150      -7.022 -34.830  41.507  1.00 25.52           O
ATOM   1166  ND2 ASN A 150      -8.866 -33.777  42.268  1.00 27.97           N
ATOM   1167  N   TYR A 151      -6.346 -31.571  38.089  1.00 20.20           N
ATOM   1168  CA  TYR A 151      -6.426 -30.460  37.154  1.00 19.93           C
ATOM   1169  C   TYR A 151      -7.798 -30.473  36.510  1.00 19.16           C
ATOM   1170  O   TYR A 151      -8.416 -31.531  36.388  1.00 20.88           O
ATOM   1171  CB  TYR A 151      -5.328 -30.565  36.094  1.00 20.27           C
ATOM   1172  CG  TYR A 151      -5.405 -31.813  35.252  1.00 17.59           C
ATOM   1173  CD1 TYR A 151      -4.756 -32.978  35.641  1.00 16.30           C
ATOM   1174  CD2 TYR A 151      -6.116 -31.820  34.060  1.00 19.29           C
ATOM   1175  CE1 TYR A 151      -4.820 -34.119  34.870  1.00 18.96           C
ATOM   1176  CE2 TYR A 151      -6.184 -32.956  33.275  1.00 18.48           C
ATOM   1177  CZ  TYR A 151      -5.542 -34.100  33.684  1.00 20.22           C
ATOM   1178  OH  TYR A 151      -5.618 -35.222  32.901  1.00 18.68           O
ATOM   1179  N   ARG A 152      -8.274 -29.305  36.098  1.00 19.03           N
ATOM   1180  CA  ARG A 152      -9.610 -29.194  35.511  1.00 21.54           C
ATOM   1181  C   ARG A 152      -9.756 -29.973  34.212  1.00 24.15           C
```

ATOM	1182	O	ARG	A	152	-8.868	-29.949	33.350	1.00	22.83	O
ATOM	1183	CB	ARG	A	152	-9.984	-27.726	35.288	1.00	23.82	C
ATOM	1184	CG	ARG	A	152	-10.372	-26.985	36.565	1.00	19.56	C
ATOM	1185	CD	ARG	A	152	-10.517	-25.503	36.288	1.00	22.58	C
ATOM	1186	NE	ARG	A	152	-9.218	-24.840	36.177	1.00	22.21	N
ATOM	1187	CZ	ARG	A	152	-9.060	-23.549	35.899	1.00	23.75	C
ATOM	1188	NH1	ARG	A	152	-10.124	-22.781	35.689	1.00	24.78	N
ATOM	1189	NH2	ARG	A	152	-7.843	-23.025	35.828	1.00	20.31	N
ATOM	1190	N	VAL	A	153	-10.886	-30.659	34.073	1.00	22.48	N
ATOM	1191	CA	VAL	A	153	-11.145	-31.457	32.883	1.00	20.45	C
ATOM	1192	C	VAL	A	153	-12.435	-31.007	32.217	1.00	24.96	C
ATOM	1193	O	VAL	A	153	-13.138	-30.138	32.741	1.00	21.71	O
ATOM	1194	CB	VAL	A	153	-11.195	-32.963	33.199	1.00	25.37	C
ATOM	1195	CG1	VAL	A	153	-9.801	-33.473	33.577	1.00	19.61	C
ATOM	1196	CG2	VAL	A	153	-12.196	-33.249	34.324	1.00	17.61	C
ATOM	1197	N	GLY	A	154	-12.723	-31.572	31.045	1.00	22.57	N
ATOM	1198	CA	GLY	A	154	-13.919	-31.222	30.296	1.00	20.33	C
ATOM	1199	C	GLY	A	154	-13.950	-29.737	30.010	1.00	25.35	C
ATOM	1200	O	GLY	A	154	-12.901	-29.091	29.975	1.00	24.22	O
ATOM	1201	N	ALA	A	155	-15.147	-29.184	29.844	1.00	23.33	N
ATOM	1202	CA	ALA	A	155	-15.290	-27.763	29.550	1.00	25.46	C
ATOM	1203	C	ALA	A	155	-14.554	-26.872	30.556	1.00	27.25	C
ATOM	1204	O	ALA	A	155	-13.980	-25.848	30.190	1.00	25.72	O
ATOM	1205	CB	ALA	A	155	-16.773	-27.377	29.469	1.00	26.57	C
ATOM	1206	N	PHE	A	156	-14.559	-27.279	31.822	1.00	25.39	N
ATOM	1207	CA	PHE	A	156	-13.948	-26.479	32.880	1.00	23.14	C
ATOM	1208	C	PHE	A	156	-12.451	-26.274	32.683	1.00	24.00	C
ATOM	1209	O	PHE	A	156	-11.910	-25.232	33.051	1.00	27.09	O
ATOM	1210	CB	PHE	A	156	-14.253	-27.108	34.239	1.00	21.78	C
ATOM	1211	CG	PHE	A	156	-15.701	-27.388	34.424	1.00	24.35	C
ATOM	1212	CD1	PHE	A	156	-16.208	-28.658	34.190	1.00	22.82	C
ATOM	1213	CD2	PHE	A	156	-16.574	-26.363	34.768	1.00	20.78	C
ATOM	1214	CE1	PHE	A	156	-17.554	-28.911	34.329	1.00	23.46	C
ATOM	1215	CE2	PHE	A	156	-17.927	-26.611	34.927	1.00	24.75	C
ATOM	1216	CZ	PHE	A	156	-18.417	-27.885	34.697	1.00	24.97	C
ATOM	1217	N	GLY	A	157	-11.789	-27.257	32.083	1.00	22.51	N
ATOM	1218	CA	GLY	A	157	-10.366	-27.155	31.837	1.00	20.74	C
ATOM	1219	C	GLY	A	157	-9.983	-26.794	30.416	1.00	24.31	C
ATOM	1220	O	GLY	A	157	-8.869	-26.320	30.188	1.00	27.21	O
ATOM	1221	N	PHE	A	158	-10.891	-26.997	29.460	1.00	23.08	N
ATOM	1222	CA	PHE	A	158	-10.503	-26.955	28.051	1.00	23.82	C
ATOM	1223	C	PHE	A	158	-11.493	-26.327	27.072	1.00	26.67	C
ATOM	1224	O	PHE	A	158	-11.243	-26.323	25.869	1.00	25.88	O
ATOM	1225	CB	PHE	A	158	-10.061	-28.354	27.578	1.00	24.72	C

```
ATOM   1226  CG  PHE A 158      -8.890 -28.887  28.349  1.00 23.29           C
ATOM   1227  CD1 PHE A 158      -9.074 -29.783  29.390  1.00 23.35           C
ATOM   1228  CD2 PHE A 158      -7.611 -28.423  28.090  1.00 25.46           C
ATOM   1229  CE1 PHE A 158      -7.998 -30.239  30.135  1.00 19.80           C
ATOM   1230  CE2 PHE A 158      -6.529 -28.875  28.833  1.00 27.06           C
ATOM   1231  CZ  PHE A 158      -6.727 -29.786  29.861  1.00 21.72           C
ATOM   1232  N   LEU A 159     -12.600 -25.780  27.566  1.00 27.43           N
ATOM   1233  CA  LEU A 159     -13.477 -25.028  26.682  1.00 24.70           C
ATOM   1234  C   LEU A 159     -12.711 -23.803  26.195  1.00 30.82           C
ATOM   1235  O   LEU A 159     -12.099 -23.085  26.979  1.00 32.86           O
ATOM   1236  CB  LEU A 159     -14.787 -24.628  27.363  1.00 24.52           C
ATOM   1237  CG  LEU A 159     -15.782 -23.842  26.488  1.00 30.30           C
ATOM   1238  CD1 LEU A 159     -17.230 -24.297  26.691  1.00 24.74           C
ATOM   1239  CD2 LEU A 159     -15.673 -22.353  26.755  1.00 27.05           C
ATOM   1240  N   ALA A 160     -12.727 -23.578  24.891  1.00 30.16           N
ATOM   1241  CA  ALA A 160     -11.931 -22.505  24.320  1.00 32.03           C
ATOM   1242  C   ALA A 160     -12.731 -21.704  23.305  1.00 35.94           C
ATOM   1243  O   ALA A 160     -13.374 -22.272  22.420  1.00 26.91           O
ATOM   1244  CB  ALA A 160     -10.669 -23.072  23.664  1.00 23.68           C
ATOM   1245  N   LEU A 161     -12.709 -20.385  23.468  1.00 29.61           N
ATOM   1246  CA  LEU A 161     -13.074 -19.468  22.401  1.00 33.91           C
ATOM   1247  C   LEU A 161     -11.794 -18.743  22.049  1.00 37.16           C
ATOM   1248  O   LEU A 161     -11.461 -17.737  22.672  1.00 40.25           O
ATOM   1249  CB  LEU A 161     -14.137 -18.478  22.860  1.00 30.90           C
ATOM   1250  CG  LEU A 161     -15.503 -19.125  23.074  1.00 36.25           C
ATOM   1251  CD1 LEU A 161     -16.367 -18.255  23.954  1.00 38.39           C
ATOM   1252  CD2 LEU A 161     -16.181 -19.401  21.737  1.00 25.71           C
ATOM   1253  N   PRO A 162     -11.050 -19.281  21.069  1.00 39.19           N
ATOM   1254  CA  PRO A 162      -9.697 -18.813  20.740  1.00 42.68           C
ATOM   1255  C   PRO A 162      -9.655 -17.315  20.457  1.00 41.58           C
ATOM   1256  O   PRO A 162     -10.478 -16.798  19.700  1.00 40.96           O
ATOM   1257  CB  PRO A 162      -9.347 -19.617  19.489  1.00 38.81           C
ATOM   1258  CG  PRO A 162     -10.157 -20.873  19.628  1.00 38.57           C
ATOM   1259  CD  PRO A 162     -11.455 -20.426  20.236  1.00 31.70           C
ATOM   1260  N   GLY A 163      -8.713 -16.627  21.089  1.00 42.18           N
ATOM   1261  CA  GLY A 163      -8.615 -15.187  20.965  1.00 40.57           C
ATOM   1262  C   GLY A 163      -9.237 -14.461  22.145  1.00 44.92           C
ATOM   1263  O   GLY A 163      -8.787 -13.378  22.515  1.00 42.80           O
ATOM   1264  N   SER A 164     -10.268 -15.055  22.742  1.00 42.86           N
ATOM   1265  CA  SER A 164     -10.985 -14.409  23.840  1.00 41.59           C
ATOM   1266  C   SER A 164     -10.200 -14.452  25.142  1.00 42.81           C
ATOM   1267  O   SER A 164      -9.379 -15.337  25.363  1.00 45.41           O
ATOM   1268  CB  SER A 164     -12.339 -15.069  24.067  1.00 43.22           C
ATOM   1269  OG  SER A 164     -12.201 -16.179  24.939  1.00 47.27           O
```

```
ATOM   1270  N   ARG A 165     -10.472 -13.498  26.016  1.00 47.50           N
ATOM   1271  CA  ARG A 165      -9.806 -13.459  27.302  1.00 46.18           C
ATOM   1272  C   ARG A 165     -10.607 -14.254  28.319  1.00 41.15           C
ATOM   1273  O   ARG A 165     -10.052 -14.782  29.276  1.00 49.63           O
ATOM   1274  CB  ARG A 165      -9.632 -12.013  27.772  1.00 53.87           C
ATOM   1275  CG  ARG A 165      -8.841 -11.137  26.807  1.00 66.29           C
ATOM   1276  CD  ARG A 165      -8.257  -9.923  27.516  1.00 83.14           C
ATOM   1277  NE  ARG A 165      -7.479 -10.322  28.686  1.00102.11           N
ATOM   1278  CZ  ARG A 165      -6.827  -9.481  29.481  1.00102.00           C
ATOM   1279  NH1 ARG A 165      -6.851  -8.178  29.231  1.00101.62           N
ATOM   1280  NH2 ARG A 165      -6.148  -9.944  30.525  1.00 92.70           N
ATOM   1281  N   GLU A 166     -11.914 -14.348  28.094  1.00 45.29           N
ATOM   1282  CA  GLU A 166     -12.818 -14.986  29.049  1.00 43.35           C
ATOM   1283  C   GLU A 166     -12.761 -16.518  29.030  1.00 42.67           C
ATOM   1284  O   GLU A 166     -13.118 -17.162  30.015  1.00 44.50           O
ATOM   1285  CB  GLU A 166     -14.257 -14.502  28.840  1.00 42.48           C
ATOM   1286  CG  GLU A 166     -14.460 -13.003  29.051  1.00 45.32           C
ATOM   1287  CD  GLU A 166     -14.277 -12.190  27.766  1.00 58.15           C
ATOM   1288  OE1 GLU A 166     -13.923 -12.780  26.716  1.00 51.95           O
ATOM   1289  OE2 GLU A 166     -14.494 -10.956  27.808  1.00 60.65           O
ATOM   1290  N   ALA A 167     -12.315 -17.094  27.916  1.00 36.54           N
ATOM   1291  CA  ALA A 167     -12.119 -18.544  27.814  1.00 37.49           C
ATOM   1292  C   ALA A 167     -11.014 -18.879  26.812  1.00 35.29           C
ATOM   1293  O   ALA A 167     -11.290 -19.308  25.688  1.00 37.34           O
ATOM   1294  CB  ALA A 167     -13.418 -19.238  27.428  1.00 36.06           C
ATOM   1295  N   PRO A 168      -9.755 -18.684  27.220  1.00 31.87           N
ATOM   1296  CA  PRO A 168      -8.604 -18.764  26.313  1.00 30.40           C
ATOM   1297  C   PRO A 168      -8.223 -20.195  25.963  1.00 32.99           C
ATOM   1298  O   PRO A 168      -7.431 -20.402  25.048  1.00 33.75           O
ATOM   1299  CB  PRO A 168      -7.481 -18.118  27.125  1.00 28.22           C
ATOM   1300  CG  PRO A 168      -7.859 -18.392  28.546  1.00 29.58           C
ATOM   1301  CD  PRO A 168      -9.355 -18.359  28.600  1.00 28.38           C
ATOM   1302  N   GLY A 169      -8.774 -21.164  26.689  1.00 34.49           N
ATOM   1303  CA  GLY A 169      -8.441 -22.560  26.474  1.00 28.77           C
ATOM   1304  C   GLY A 169      -7.206 -22.994  27.246  1.00 29.61           C
ATOM   1305  O   GLY A 169      -6.448 -22.160  27.757  1.00 26.14           O
ATOM   1306  N   ASN A 170      -7.025 -24.310  27.334  1.00 23.30           N
ATOM   1307  CA  ASN A 170      -5.848 -24.922  27.939  1.00 23.17           C
ATOM   1308  C   ASN A 170      -5.658 -24.679  29.442  1.00 20.20           C
ATOM   1309  O   ASN A 170      -4.588 -24.960  29.977  1.00 19.47           O
ATOM   1310  CB  ASN A 170      -4.571 -24.525  27.179  1.00 23.14           C
ATOM   1311  CG  ASN A 170      -4.576 -25.000  25.741  1.00 28.93           C
ATOM   1312  OD1 ASN A 170      -5.194 -26.007  25.412  1.00 29.42           O
ATOM   1313  ND2 ASN A 170      -3.885 -24.272  24.872  1.00 26.40           N
```

```
ATOM   1314  N   VAL A 171      -6.681 -24.191  30.136  1.00 23.36           N
ATOM   1315  CA  VAL A 171      -6.470 -23.843  31.546  1.00 25.34           C
ATOM   1316  C   VAL A 171      -6.166 -25.067  32.435  1.00 24.82           C
ATOM   1317  O   VAL A 171      -5.452 -24.963  33.428  1.00 22.81           O
ATOM   1318  CB  VAL A 171      -7.580 -22.912  32.125  1.00 26.79           C
ATOM   1319  CG1 VAL A 171      -7.583 -21.571  31.377  1.00 20.81           C
ATOM   1320  CG2 VAL A 171      -8.966 -23.577  32.105  1.00 22.54           C
ATOM   1321  N   GLY A 172      -6.670 -26.232  32.048  1.00 24.47           N
ATOM   1322  CA  GLY A 172      -6.340 -27.452  32.761  1.00 21.32           C
ATOM   1323  C   GLY A 172      -4.860 -27.778  32.650  1.00 22.42           C
ATOM   1324  O   GLY A 172      -4.252 -28.318  33.591  1.00 25.51           O
ATOM   1325  N   LEU A 173      -4.280 -27.467  31.492  1.00 21.66           N
ATOM   1326  CA  LEU A 173      -2.841 -27.618  31.293  1.00 20.92           C
ATOM   1327  C   LEU A 173      -2.092 -26.643  32.191  1.00 21.56           C
ATOM   1328  O   LEU A 173      -1.064 -27.004  32.778  1.00 20.64           O
ATOM   1329  CB  LEU A 173      -2.457 -27.396  29.833  1.00 22.08           C
ATOM   1330  CG  LEU A 173      -2.644 -28.580  28.887  1.00 21.24           C
ATOM   1331  CD1 LEU A 173      -2.318 -28.166  27.471  1.00 16.67           C
ATOM   1332  CD2 LEU A 173      -1.756 -29.739  29.324  1.00 19.26           C
ATOM   1333  N   LEU A 174      -2.620 -25.422  32.315  1.00 18.37           N
ATOM   1334  CA  LEU A 174      -2.036 -24.433  33.232  1.00 23.66           C
ATOM   1335  C   LEU A 174      -2.185 -24.849  34.701  1.00 20.11           C
ATOM   1336  O   LEU A 174      -1.313 -24.564  35.511  1.00 22.41           O
ATOM   1337  CB  LEU A 174      -2.590 -23.018  32.986  1.00 19.19           C
ATOM   1338  CG  LEU A 174      -2.312 -22.416  31.599  1.00 30.59           C
ATOM   1339  CD1 LEU A 174      -2.970 -21.061  31.435  1.00 23.97           C
ATOM   1340  CD2 LEU A 174      -0.818 -22.302  31.345  1.00 26.40           C
ATOM   1341  N   ASP A 175      -3.277 -25.535  35.039  1.00 24.57           N
ATOM   1342  CA  ASP A 175      -3.422 -26.105  36.378  1.00 19.23           C
ATOM   1343  C   ASP A 175      -2.235 -27.018  36.655  1.00 26.36           C
ATOM   1344  O   ASP A 175      -1.667 -26.985  37.749  1.00 25.22           O
ATOM   1345  CB  ASP A 175      -4.706 -26.923  36.520  1.00 18.11           C
ATOM   1346  CG  ASP A 175      -5.957 -26.082  36.431  1.00 24.05           C
ATOM   1347  OD1 ASP A 175      -5.859 -24.842  36.578  1.00 22.04           O
ATOM   1348  OD2 ASP A 175      -7.044 -26.672  36.214  1.00 23.53           O
ATOM   1349  N   GLN A 176      -1.857 -27.825  35.662  1.00 21.63           N
ATOM   1350  CA  GLN A 176      -0.713 -28.726  35.813  1.00 21.59           C
ATOM   1351  C   GLN A 176       0.612 -27.962  35.967  1.00 23.98           C
ATOM   1352  O   GLN A 176       1.465 -28.320  36.800  1.00 25.19           O
ATOM   1353  CB  GLN A 176      -0.610 -29.683  34.622  1.00 25.77           C
ATOM   1354  CG  GLN A 176      -1.755 -30.680  34.475  1.00 20.49           C
ATOM   1355  CD  GLN A 176      -1.713 -31.394  33.129  1.00 23.90           C
ATOM   1356  OE1 GLN A 176      -0.695 -31.364  32.423  1.00 21.15           O
ATOM   1357  NE2 GLN A 176      -2.822 -32.028  32.761  1.00 20.77           N
```

```
ATOM   1358  N   ARG A 177       0.780 -26.909  35.168  1.00 21.47           N
ATOM   1359  CA  ARG A 177       2.002 -26.118  35.208  1.00 22.37           C
ATOM   1360  C   ARG A 177       2.157 -25.443  36.556  1.00 24.90           C
ATOM   1361  O   ARG A 177       3.255 -25.421  37.132  1.00 23.90           O
ATOM   1362  CB  ARG A 177       2.023 -25.062  34.102  1.00 24.55           C
ATOM   1363  CG  ARG A 177       3.244 -24.156  34.186  1.00 28.12           C
ATOM   1364  CD  ARG A 177       3.264 -23.126  33.088  1.00 25.82           C
ATOM   1365  NE  ARG A 177       3.475 -23.750  31.785  1.00 31.94           N
ATOM   1366  CZ  ARG A 177       3.263 -23.132  30.630  1.00 32.92           C
ATOM   1367  NH1 ARG A 177       2.834 -21.874  30.640  1.00 28.06           N
ATOM   1368  NH2 ARG A 177       3.474 -23.765  29.477  1.00 26.36           N
ATOM   1369  N   LEU A 178       1.053 -24.900  37.061  1.00 21.03           N
ATOM   1370  CA  LEU A 178       1.070 -24.249  38.363  1.00 24.07           C
ATOM   1371  C   LEU A 178       1.480 -25.259  39.437  1.00 23.99           C
ATOM   1372  O   LEU A 178       2.286 -24.939  40.315  1.00 26.85           O
ATOM   1373  CB  LEU A 178      -0.281 -23.593  38.680  1.00 22.34           C
ATOM   1374  CG  LEU A 178      -0.405 -22.944  40.064  1.00 26.94           C
ATOM   1375  CD1 LEU A 178       0.616 -21.827  40.244  1.00 22.05           C
ATOM   1376  CD2 LEU A 178      -1.812 -22.424  40.308  1.00 22.20           C
ATOM   1377  N   ALA A 179       0.964 -26.486  39.350  1.00 22.50           N
ATOM   1378  CA  ALA A 179       1.376 -27.535  40.295  1.00 24.01           C
ATOM   1379  C   ALA A 179       2.870 -27.854  40.175  1.00 22.18           C
ATOM   1380  O   ALA A 179       3.550 -28.065  41.180  1.00 24.04           O
ATOM   1381  CB  ALA A 179       0.534 -28.806  40.118  1.00 21.42           C
ATOM   1382  N   LEU A 180       3.381 -27.889  38.945  1.00 23.96           N
ATOM   1383  CA  LEU A 180       4.812 -28.114  38.740  1.00 24.42           C
ATOM   1384  C   LEU A 180       5.661 -26.967  39.319  1.00 26.59           C
ATOM   1385  O   LEU A 180       6.717 -27.202  39.907  1.00 28.51           O
ATOM   1386  CB  LEU A 180       5.124 -28.346  37.262  1.00 24.29           C
ATOM   1387  CG  LEU A 180       4.501 -29.602  36.639  1.00 23.58           C
ATOM   1388  CD1 LEU A 180       4.891 -29.726  35.171  1.00 23.58           C
ATOM   1389  CD2 LEU A 180       4.905 -30.835  37.404  1.00 24.75           C
ATOM   1390  N   GLN A 181       5.185 -25.735  39.165  1.00 24.40           N
ATOM   1391  CA  GLN A 181       5.803 -24.588  39.824  1.00 30.04           C
ATOM   1392  C   GLN A 181       5.723 -24.715  41.345  1.00 30.06           C
ATOM   1393  O   GLN A 181       6.678 -24.373  42.052  1.00 29.04           O
ATOM   1394  CB  GLN A 181       5.151 -23.279  39.372  1.00 25.71           C
ATOM   1395  CG  GLN A 181       5.426 -22.947  37.921  1.00 30.87           C
ATOM   1396  CD  GLN A 181       4.502 -21.876  37.386  1.00 35.16           C
ATOM   1397  OE1 GLN A 181       3.504 -21.532  38.018  1.00 36.01           O
ATOM   1398  NE2 GLN A 181       4.826 -21.343  36.209  1.00 35.85           N
ATOM   1399  N   TRP A 182       4.593 -25.210  41.846  1.00 27.52           N
ATOM   1400  CA  TRP A 182       4.445 -25.435  43.286  1.00 28.59           C
ATOM   1401  C   TRP A 182       5.521 -26.415  43.751  1.00 29.75           C
```

```
ATOM   1402  O   TRP A 182       6.120 -26.243  44.811  1.00 27.53           O
ATOM   1403  CB  TRP A 182       3.047 -25.971  43.619  1.00 19.63           C
ATOM   1404  CG  TRP A 182       2.758 -26.089  45.095  1.00 25.81           C
ATOM   1405  CD1 TRP A 182       2.094 -25.179  45.879  1.00 20.03           C
ATOM   1406  CD2 TRP A 182       3.099 -27.185  45.960  1.00 21.26           C
ATOM   1407  NE1 TRP A 182       2.012 -25.639  47.164  1.00 25.43           N
ATOM   1408  CE2 TRP A 182       2.622 -26.864  47.246  1.00 22.71           C
ATOM   1409  CE3 TRP A 182       3.767 -28.401  45.772  1.00 21.88           C
ATOM   1410  CZ2 TRP A 182       2.790 -27.714  48.342  1.00 21.35           C
ATOM   1411  CZ3 TRP A 182       3.934 -29.244  46.859  1.00 25.21           C
ATOM   1412  CH2 TRP A 182       3.442 -28.901  48.128  1.00 19.84           C
ATOM   1413  N   VAL A 183       5.772 -27.437  42.938  1.00 30.41           N
ATOM   1414  CA  VAL A 183       6.763 -28.445  43.289  1.00 28.57           C
ATOM   1415  C   VAL A 183       8.161 -27.836  43.366  1.00 30.28           C
ATOM   1416  O   VAL A 183       8.889 -28.062  44.340  1.00 32.75           O
ATOM   1417  CB  VAL A 183       6.723 -29.642  42.322  1.00 24.72           C
ATOM   1418  CG1 VAL A 183       7.979 -30.489  42.455  1.00 21.77           C
ATOM   1419  CG2 VAL A 183       5.481 -30.480  42.592  1.00 18.01           C
ATOM   1420  N   GLN A 184       8.516 -27.043  42.360  1.00 25.98           N
ATOM   1421  CA  GLN A 184       9.790 -26.326  42.353  1.00 31.20           C
ATOM   1422  C   GLN A 184      10.019 -25.537  43.633  1.00 30.44           C
ATOM   1423  O   GLN A 184      11.121 -25.552  44.198  1.00 27.22           O
ATOM   1424  CB  GLN A 184       9.876 -25.389  41.150  1.00 23.87           C
ATOM   1425  CG  GLN A 184      10.146 -26.123  39.860  1.00 28.49           C
ATOM   1426  CD  GLN A 184      11.394 -26.977  39.954  1.00 34.11           C
ATOM   1427  OE1 GLN A 184      12.504 -26.462  40.108  1.00 38.97           O
ATOM   1428  NE2 GLN A 184      11.219 -28.288  39.882  1.00 33.18           N
ATOM   1429  N   GLU A 185       8.967 -24.880  44.102  1.00 25.84           N
ATOM   1430  CA  GLU A 185       9.074 -23.990  45.251  1.00 30.03           C
ATOM   1431  C   GLU A 185       8.917 -24.669  46.611  1.00 38.05           C
ATOM   1432  O   GLU A 185       9.407 -24.155  47.614  1.00 39.83           O
ATOM   1433  CB  GLU A 185       8.077 -22.835  45.109  1.00 30.10           C
ATOM   1434  CG  GLU A 185       8.438 -21.903  43.949  1.00 40.79           C
ATOM   1435  CD  GLU A 185       7.364 -20.881  43.639  1.00 51.06           C
ATOM   1436  OE1 GLU A 185       7.330 -20.395  42.485  1.00 48.71           O
ATOM   1437  OE2 GLU A 185       6.560 -20.561  44.544  1.00 55.46           O
ATOM   1438  N   ASN A 186       8.254 -25.823  46.651  1.00 34.64           N
ATOM   1439  CA  ASN A 186       7.857 -26.392  47.934  1.00 29.15           C
ATOM   1440  C   ASN A 186       8.298 -27.821  48.217  1.00 31.70           C
ATOM   1441  O   ASN A 186       8.195 -28.285  49.356  1.00 36.34           O
ATOM   1442  CB  ASN A 186       6.341 -26.287  48.106  1.00 24.31           C
ATOM   1443  CG  ASN A 186       5.857 -24.855  48.090  1.00 28.03           C
ATOM   1444  OD1 ASN A 186       6.114 -24.101  49.020  1.00 34.74           O
ATOM   1445  ND2 ASN A 186       5.152 -24.471  47.031  1.00 31.10           N
```

```
ATOM   1446  N   VAL A 187       8.785 -28.530  47.206  1.00 28.96           N
ATOM   1447  CA  VAL A 187       8.974 -29.964  47.385  1.00 29.16           C
ATOM   1448  C   VAL A 187      10.131 -30.274  48.325  1.00 33.47           C
ATOM   1449  O   VAL A 187      10.126 -31.312  48.997  1.00 32.91           O
ATOM   1450  CB  VAL A 187       9.082 -30.740  46.038  1.00 32.40           C
ATOM   1451  CG1 VAL A 187      10.479 -30.622  45.434  1.00 21.52           C
ATOM   1452  CG2 VAL A 187       8.687 -32.211  46.240  1.00 25.93           C
ATOM   1453  N   ALA A 188      11.101 -29.361  48.394  1.00 33.21           N
ATOM   1454  CA  ALA A 188      12.245 -29.523  49.291  1.00 34.13           C
ATOM   1455  C   ALA A 188      11.784 -29.648  50.741  1.00 32.48           C
ATOM   1456  O   ALA A 188      12.394 -30.364  51.538  1.00 34.72           O
ATOM   1457  CB  ALA A 188      13.229 -28.363  49.137  1.00 25.47           C
ATOM   1458  N   ALA A 189      10.690 -28.971  51.078  1.00 29.35           N
ATOM   1459  CA  ALA A 189      10.181 -29.001  52.448  1.00 31.49           C
ATOM   1460  C   ALA A 189       9.713 -30.402  52.841  1.00 38.15           C
ATOM   1461  O   ALA A 189       9.433 -30.658  54.013  1.00 37.07           O
ATOM   1462  CB  ALA A 189       9.043 -27.995  52.625  1.00 26.13           C
ATOM   1463  N   PHE A 190       9.619 -31.298  51.857  1.00 33.91           N
ATOM   1464  CA  PHE A 190       9.188 -32.668  52.104  1.00 33.23           C
ATOM   1465  C   PHE A 190      10.342 -33.653  51.952  1.00 31.56           C
ATOM   1466  O   PHE A 190      10.177 -34.846  52.184  1.00 37.75           O
ATOM   1467  CB  PHE A 190       8.053 -33.058  51.150  1.00 30.12           C
ATOM   1468  CG  PHE A 190       6.829 -32.188  51.261  1.00 27.60           C
ATOM   1469  CD1 PHE A 190       6.756 -30.975  50.590  1.00 27.23           C
ATOM   1470  CD2 PHE A 190       5.741 -32.597  52.011  1.00 25.38           C
ATOM   1471  CE1 PHE A 190       5.632 -30.181  50.678  1.00 23.86           C
ATOM   1472  CE2 PHE A 190       4.606 -31.806  52.100  1.00 26.17           C
ATOM   1473  CZ  PHE A 190       4.554 -30.597  51.434  1.00 23.16           C
ATOM   1474  N   GLY A 191      11.509 -33.150  51.562  1.00 25.12           N
ATOM   1475  CA  GLY A 191      12.659 -34.002  51.327  1.00 31.32           C
ATOM   1476  C   GLY A 191      12.924 -34.229  49.851  1.00 35.18           C
ATOM   1477  O   GLY A 191      13.851 -34.947  49.475  1.00 36.31           O
ATOM   1478  N   GLY A 192      12.114 -33.603  49.004  1.00 31.45           N
ATOM   1479  CA  GLY A 192      12.225 -33.812  47.578  1.00 31.16           C
ATOM   1480  C   GLY A 192      13.257 -32.908  46.955  1.00 30.81           C
ATOM   1481  O   GLY A 192      13.529 -31.826  47.469  1.00 41.84           O
ATOM   1482  N   ASP A 193      13.822 -33.355  45.840  1.00 29.70           N
ATOM   1483  CA  ASP A 193      14.846 -32.611  45.120  1.00 30.22           C
ATOM   1484  C   ASP A 193      14.242 -31.998  43.858  1.00 35.83           C
ATOM   1485  O   ASP A 193      14.036 -32.694  42.866  1.00 37.04           O
ATOM   1486  CB  ASP A 193      16.003 -33.559  44.780  1.00 32.05           C
ATOM   1487  CG  ASP A 193      17.044 -32.943  43.858  1.00 31.24           C
ATOM   1488  OD1 ASP A 193      16.996 -31.734  43.560  1.00 35.77           O
ATOM   1489  OD2 ASP A 193      17.940 -33.690  43.425  1.00 39.32           O
```

```
ATOM   1490  N   PRO A 194      13.979 -30.681  43.888  1.00 40.39           N
ATOM   1491  CA  PRO A 194      13.401 -29.929  42.760  1.00 28.55           C
ATOM   1492  C   PRO A 194      14.256 -29.965  41.494  1.00 36.30           C
ATOM   1493  O   PRO A 194      13.759 -29.658  40.398  1.00 31.78           O
ATOM   1494  CB  PRO A 194      13.302 -28.494  43.297  1.00 34.98           C
ATOM   1495  CG  PRO A 194      14.241 -28.449  44.485  1.00 31.68           C
ATOM   1496  CD  PRO A 194      14.195 -29.830  45.073  1.00 27.86           C
ATOM   1497  N   THR A 195      15.522 -30.345  41.642  1.00 38.88           N
ATOM   1498  CA  THR A 195      16.428 -30.470  40.496  1.00 39.65           C
ATOM   1499  C   THR A 195      16.359 -31.869  39.873  1.00 38.04           C
ATOM   1500  O   THR A 195      17.034 -32.145  38.877  1.00 43.83           O
ATOM   1501  CB  THR A 195      17.900 -30.162  40.888  1.00 42.76           C
ATOM   1502  OG1 THR A 195      18.397 -31.195  41.755  1.00 40.81           O
ATOM   1503  CG2 THR A 195      18.009 -28.810  41.598  1.00 30.20           C
ATOM   1504  N   SER A 196      15.549 -32.750  40.458  1.00 31.70           N
ATOM   1505  CA  SER A 196      15.331 -34.083  39.879  1.00 37.92           C
ATOM   1506  C   SER A 196      13.843 -34.470  39.850  1.00 36.52           C
ATOM   1507  O   SER A 196      13.366 -35.254  40.680  1.00 32.26           O
ATOM   1508  CB  SER A 196      16.130 -35.143  40.634  1.00 34.48           C
ATOM   1509  OG  SER A 196      15.994 -36.408  40.005  1.00 47.57           O
ATOM   1510  N   VAL A 197      13.117 -33.915  38.888  1.00 30.36           N
ATOM   1511  CA  VAL A 197      11.680 -34.125  38.791  1.00 27.23           C
ATOM   1512  C   VAL A 197      11.342 -34.880  37.510  1.00 28.07           C
ATOM   1513  O   VAL A 197      11.629 -34.412  36.407  1.00 25.39           O
ATOM   1514  CB  VAL A 197      10.918 -32.781  38.817  1.00 28.35           C
ATOM   1515  CG1 VAL A 197       9.448 -32.977  38.406  1.00 25.43           C
ATOM   1516  CG2 VAL A 197      11.022 -32.137  40.194  1.00 24.24           C
ATOM   1517  N   THR A 198      10.745 -36.058  37.662  1.00 29.77           N
ATOM   1518  CA  THR A 198      10.326 -36.859  36.513  1.00 26.83           C
ATOM   1519  C   THR A 198       8.802 -36.861  36.407  1.00 24.98           C
ATOM   1520  O   THR A 198       8.106 -37.212  37.364  1.00 22.26           O
ATOM   1521  CB  THR A 198      10.843 -38.320  36.609  1.00 29.00           C
ATOM   1522  OG1 THR A 198      12.275 -38.332  36.717  1.00 27.34           O
ATOM   1523  CG2 THR A 198      10.430 -39.124  35.381  1.00 22.34           C
ATOM   1524  N   LEU A 199       8.288 -36.446  35.249  1.00 23.95           N
ATOM   1525  CA  LEU A 199       6.853 -36.498  34.977  1.00 21.52           C
ATOM   1526  C   LEU A 199       6.470 -37.862  34.417  1.00 27.34           C
ATOM   1527  O   LEU A 199       7.131 -38.377  33.509  1.00 23.45           O
ATOM   1528  CB  LEU A 199       6.462 -35.454  33.934  1.00 27.87           C
ATOM   1529  CG  LEU A 199       6.779 -33.989  34.185  1.00 24.15           C
ATOM   1530  CD1 LEU A 199       6.178 -33.142  33.068  1.00 23.75           C
ATOM   1531  CD2 LEU A 199       6.234 -33.583  35.533  1.00 27.88           C
ATOM   1532  N   PHE A 200       5.403 -38.446  34.953  1.00 21.41           N
ATOM   1533  CA  PHE A 200       4.821 -39.632  34.339  1.00 22.36           C
```

```
ATOM   1534  C   PHE A 200       3.306 -39.531  34.310  1.00 22.29           C
ATOM   1535  O   PHE A 200       2.690 -38.943  35.203  1.00 21.52           O
ATOM   1536  CB  PHE A 200       5.344 -40.970  34.926  1.00 19.85           C
ATOM   1537  CG  PHE A 200       4.913 -41.279  36.359  1.00 22.87           C
ATOM   1538  CD1 PHE A 200       4.960 -40.319  37.362  1.00 21.29           C
ATOM   1539  CD2 PHE A 200       4.522 -42.572  36.698  1.00 20.88           C
ATOM   1540  CE1 PHE A 200       4.596 -40.635  38.674  1.00 23.94           C
ATOM   1541  CE2 PHE A 200       4.161 -42.896  37.999  1.00 26.67           C
ATOM   1542  CZ  PHE A 200       4.196 -41.919  38.995  1.00 24.32           C
ATOM   1543  N   GLY A 201       2.717 -40.074  33.254  1.00 18.09           N
ATOM   1544  CA  GLY A 201       1.294 -39.931  33.037  1.00 18.76           C
ATOM   1545  C   GLY A 201       0.840 -40.993  32.067  1.00 22.17           C
ATOM   1546  O   GLY A 201       1.650 -41.569  31.344  1.00 19.77           O
ATOM   1547  N   GLU A 202      -0.460 -41.263  32.059  1.00 23.99           N
ATOM   1548  CA  GLU A 202      -1.006 -42.292  31.192  1.00 21.05           C
ATOM   1549  C   GLU A 202      -2.154 -41.708  30.386  1.00 21.75           C
ATOM   1550  O   GLU A 202      -2.861 -40.819  30.867  1.00 20.07           O
ATOM   1551  CB  GLU A 202      -1.439 -43.509  32.013  1.00 21.86           C
ATOM   1552  CG  GLU A 202      -1.869 -44.715  31.196  1.00 20.21           C
ATOM   1553  CD  GLU A 202      -3.337 -44.662  30.840  1.00 22.32           C
ATOM   1554  OE1 GLU A 202      -4.014 -43.731  31.319  1.00 21.71           O
ATOM   1555  OE2 GLU A 202      -3.809 -45.533  30.073  1.00 22.82           O
ATOM   1556  N   SER A 203      -2.317 -42.208  29.160  1.00 22.58           N
ATOM   1557  CA  SER A 203      -3.281 -41.676  28.192  1.00 21.40           C
ATOM   1558  C   SER A 203      -3.263 -40.140  28.139  1.00 19.08           C
ATOM   1559  O   SER A 203      -2.223 -39.555  27.830  1.00 20.56           O
ATOM   1560  CB  SER A 203      -4.691 -42.256  28.395  1.00 21.10           C
ATOM   1561  OG  SER A 203      -5.337 -41.735  29.543  1.00 34.01           O
ATOM   1562  N   ALA A 204      -4.380 -39.482  28.438  1.00 15.74           N
ATOM   1563  CA  ALA A 204      -4.387 -38.013  28.414  1.00 19.38           C
ATOM   1564  C   ALA A 204      -3.310 -37.401  29.332  1.00 23.32           C
ATOM   1565  O   ALA A 204      -2.815 -36.298  29.071  1.00 21.56           O
ATOM   1566  CB  ALA A 204      -5.772 -37.456  28.740  1.00 18.12           C
ATOM   1567  N   GLY A 205      -2.942 -38.133  30.388  1.00 18.85           N
ATOM   1568  CA  GLY A 205      -1.814 -37.768  31.224  1.00 17.67           C
ATOM   1569  C   GLY A 205      -0.475 -37.816  30.495  1.00 20.64           C
ATOM   1570  O   GLY A 205       0.366 -36.933  30.661  1.00 20.49           O
ATOM   1571  N   ALA A 206      -0.275 -38.866  29.704  1.00 20.64           N
ATOM   1572  CA  ALA A 206       0.900 -38.992  28.854  1.00 24.30           C
ATOM   1573  C   ALA A 206       0.931 -37.856  27.828  1.00 23.06           C
ATOM   1574  O   ALA A 206       1.968 -37.248  27.594  1.00 24.65           O
ATOM   1575  CB  ALA A 206       0.912 -40.366  28.150  1.00 18.92           C
ATOM   1576  N   ALA A 207      -0.220 -37.563  27.238  1.00 23.17           N
ATOM   1577  CA  ALA A 207      -0.316 -36.500  26.248  1.00 25.42           C
```

```
ATOM   1578  C   ALA A 207       0.049 -35.176  26.898  1.00 25.87           C
ATOM   1579  O   ALA A 207       0.704 -34.327  26.281  1.00 26.50           O
ATOM   1580  CB  ALA A 207      -1.732 -36.442  25.653  1.00 19.41           C
ATOM   1581  N   SER A 208      -0.375 -35.020  28.151  1.00 23.04           N
ATOM   1582  CA  SER A 208      -0.065 -33.838  28.945  1.00 20.93           C
ATOM   1583  C   SER A 208       1.435 -33.692  29.162  1.00 24.54           C
ATOM   1584  O   SER A 208       1.991 -32.612  28.944  1.00 22.45           O
ATOM   1585  CB  SER A 208      -0.779 -33.896  30.301  1.00 20.63           C
ATOM   1586  OG  SER A 208      -2.160 -33.591  30.172  1.00 22.78           O
ATOM   1587  N   VAL A 209       2.077 -34.771  29.617  1.00 23.12           N
ATOM   1588  CA  VAL A 209       3.531 -34.801  29.755  1.00 18.91           C
ATOM   1589  C   VAL A 209       4.188 -34.315  28.465  1.00 21.45           C
ATOM   1590  O   VAL A 209       5.019 -33.409  28.488  1.00 24.76           O
ATOM   1591  CB  VAL A 209       4.052 -36.218  30.081  1.00 19.87           C
ATOM   1592  CG1 VAL A 209       5.556 -36.276  29.927  1.00 19.06           C
ATOM   1593  CG2 VAL A 209       3.633 -36.659  31.495  1.00 19.18           C
ATOM   1594  N   GLY A 210       3.785 -34.899  27.340  1.00 20.80           N
ATOM   1595  CA  GLY A 210       4.320 -34.518  26.045  1.00 22.46           C
ATOM   1596  C   GLY A 210       4.125 -33.043  25.729  1.00 25.68           C
ATOM   1597  O   GLY A 210       4.988 -32.402  25.130  1.00 26.72           O
ATOM   1598  N   MET A 211       2.989 -32.494  26.138  1.00 25.06           N
ATOM   1599  CA  MET A 211       2.700 -31.097  25.851  1.00 22.88           C
ATOM   1600  C   MET A 211       3.578 -30.177  26.665  1.00 25.46           C
ATOM   1601  O   MET A 211       4.002 -29.127  26.174  1.00 23.84           O
ATOM   1602  CB  MET A 211       1.219 -30.785  26.061  1.00 25.67           C
ATOM   1603  CG  MET A 211       0.393 -31.141  24.831  1.00 29.35           C
ATOM   1604  SD  MET A 211      -1.360 -31.087  25.155  1.00 36.97           S
ATOM   1605  CE  MET A 211      -1.944 -32.463  24.149  1.00 23.81           C
ATOM   1606  N   HIS A 212       3.861 -30.577  27.902  1.00 21.18           N
ATOM   1607  CA  HIS A 212       4.811 -29.850  28.730  1.00 24.51           C
ATOM   1608  C   HIS A 212       6.221 -29.879  28.125  1.00 27.22           C
ATOM   1609  O   HIS A 212       6.968 -28.917  28.270  1.00 25.11           O
ATOM   1610  CB  HIS A 212       4.817 -30.380  30.169  1.00 20.21           C
ATOM   1611  CG  HIS A 212       3.569 -30.052  30.928  1.00 26.06           C
ATOM   1612  ND1 HIS A 212       3.224 -28.762  31.266  1.00 23.58           N
ATOM   1613  CD2 HIS A 212       2.579 -30.845  31.404  1.00 23.66           C
ATOM   1614  CE1 HIS A 212       2.074 -28.772  31.917  1.00 23.73           C
ATOM   1615  NE2 HIS A 212       1.663 -30.024  32.017  1.00 24.95           N
ATOM   1616  N   LEU A 213       6.573 -30.978  27.453  1.00 20.63           N
ATOM   1617  CA  LEU A 213       7.838 -31.070  26.731  1.00 28.26           C
ATOM   1618  C   LEU A 213       7.926 -30.039  25.611  1.00 32.08           C
ATOM   1619  O   LEU A 213       9.008 -29.540  25.305  1.00 29.91           O
ATOM   1620  CB  LEU A 213       8.008 -32.454  26.104  1.00 26.59           C
ATOM   1621  CG  LEU A 213       8.497 -33.568  27.015  1.00 28.06           C
```

```
ATOM   1622  CD1 LEU A 213       8.501 -34.869  26.245  1.00 27.31           C
ATOM   1623  CD2 LEU A 213       9.879 -33.238  27.537  1.00 33.82           C
ATOM   1624  N   LEU A 214       6.787 -29.730  24.998  1.00 25.69           N
ATOM   1625  CA  LEU A 214       6.775 -28.911  23.786  1.00 25.95           C
ATOM   1626  C   LEU A 214       6.418 -27.464  24.083  1.00 27.71           C
ATOM   1627  O   LEU A 214       6.328 -26.640  23.175  1.00 30.83           O
ATOM   1628  CB  LEU A 214       5.801 -29.499  22.766  1.00 20.80           C
ATOM   1629  CG  LEU A 214       6.011 -30.992  22.510  1.00 29.24           C
ATOM   1630  CD1 LEU A 214       4.891 -31.569  21.665  1.00 27.64           C
ATOM   1631  CD2 LEU A 214       7.371 -31.244  21.858  1.00 31.70           C
ATOM   1632  N   SER A 215       6.209 -27.157  25.360  1.00 26.20           N
ATOM   1633  CA  SER A 215       5.897 -25.793  25.765  1.00 28.91           C
ATOM   1634  C   SER A 215       7.016 -25.256  26.648  1.00 24.75           C
ATOM   1635  O   SER A 215       7.187 -25.721  27.767  1.00 33.26           O
ATOM   1636  CB  SER A 215       4.561 -25.756  26.506  1.00 27.70           C
ATOM   1637  OG  SER A 215       4.174 -24.428  26.796  1.00 28.17           O
ATOM   1638  N   PRO A 216       7.794 -24.282  26.141  1.00 32.91           N
ATOM   1639  CA  PRO A 216       8.973 -23.768  26.867  1.00 29.19           C
ATOM   1640  C   PRO A 216       8.752 -23.309  28.323  1.00 27.95           C
ATOM   1641  O   PRO A 216       9.626 -23.571  29.147  1.00 31.79           O
ATOM   1642  CB  PRO A 216       9.465 -22.615  25.977  1.00 30.22           C
ATOM   1643  CG  PRO A 216       8.999 -23.011  24.574  1.00 29.81           C
ATOM   1644  CD  PRO A 216       7.665 -23.687  24.792  1.00 30.86           C
ATOM   1645  N   PRO A 217       7.624 -22.644  28.648  1.00 28.03           N
ATOM   1646  CA  PRO A 217       7.472 -22.285  30.073  1.00 24.13           C
ATOM   1647  C   PRO A 217       7.367 -23.474  31.040  1.00 28.02           C
ATOM   1648  O   PRO A 217       7.576 -23.284  32.237  1.00 32.27           O
ATOM   1649  CB  PRO A 217       6.171 -21.479  30.097  1.00 22.50           C
ATOM   1650  CG  PRO A 217       6.063 -20.899  28.709  1.00 26.89           C
ATOM   1651  CD  PRO A 217       6.621 -21.971  27.798  1.00 25.03           C
ATOM   1652  N   SER A 218       7.035 -24.663  30.542  1.00 26.88           N
ATOM   1653  CA  SER A 218       6.979 -25.860  31.384  1.00 32.13           C
ATOM   1654  C   SER A 218       8.309 -26.586  31.418  1.00 30.74           C
ATOM   1655  O   SER A 218       8.635 -27.243  32.398  1.00 31.85           O
ATOM   1656  CB  SER A 218       5.914 -26.843  30.890  1.00 28.56           C
ATOM   1657  OG  SER A 218       4.613 -26.300  30.992  1.00 30.46           O
ATOM   1658  N   ARG A 219       9.067 -26.462  30.337  1.00 34.04           N
ATOM   1659  CA  ARG A 219      10.268 -27.261  30.126  1.00 38.21           C
ATOM   1660  C   ARG A 219      11.298 -27.079  31.253  1.00 35.64           C
ATOM   1661  O   ARG A 219      12.076 -27.987  31.552  1.00 36.49           O
ATOM   1662  CB  ARG A 219      10.849 -26.973  28.728  1.00 42.02           C
ATOM   1663  CG  ARG A 219      11.927 -27.940  28.247  1.00 49.52           C
ATOM   1664  CD  ARG A 219      11.431 -29.371  28.107  1.00 45.99           C
ATOM   1665  NE  ARG A 219      12.547 -30.316  27.990  1.00 57.13           N
```

```
ATOM   1666  CZ  ARG A 219      13.133 -30.939  29.019  1.00 58.51           C
ATOM   1667  NH1 ARG A 219      12.722 -30.741  30.277  1.00 37.69           N
ATOM   1668  NH2 ARG A 219      14.140 -31.775  28.790  1.00 50.40           N
ATOM   1669  N   GLY A 220      11.266 -25.931  31.917  1.00 36.05           N
ATOM   1670  CA  GLY A 220      12.149 -25.706  33.049  1.00 24.05           C
ATOM   1671  C   GLY A 220      11.633 -26.209  34.383  1.00 34.44           C
ATOM   1672  O   GLY A 220      12.254 -25.963  35.417  1.00 40.09           O
ATOM   1673  N   LEU A 221      10.507 -26.918  34.374  1.00 30.35           N
ATOM   1674  CA  LEU A 221       9.896 -27.385  35.616  1.00 27.02           C
ATOM   1675  C   LEU A 221      10.130 -28.865  35.894  1.00 32.58           C
ATOM   1676  O   LEU A 221       9.657 -29.396  36.902  1.00 35.22           O
ATOM   1677  CB  LEU A 221       8.399 -27.095  35.613  1.00 26.15           C
ATOM   1678  CG  LEU A 221       8.101 -25.615  35.399  1.00 29.41           C
ATOM   1679  CD1 LEU A 221       6.598 -25.378  35.348  1.00 26.24           C
ATOM   1680  CD2 LEU A 221       8.761 -24.796  36.511  1.00 27.01           C
ATOM   1681  N   PHE A 222      10.860 -29.532  35.007  1.00 27.81           N
ATOM   1682  CA  PHE A 222      11.101 -30.959  35.170  1.00 30.04           C
ATOM   1683  C   PHE A 222      12.283 -31.395  34.313  1.00 30.60           C
ATOM   1684  O   PHE A 222      12.796 -30.618  33.511  1.00 32.94           O
ATOM   1685  CB  PHE A 222       9.841 -31.771  34.834  1.00 27.84           C
ATOM   1686  CG  PHE A 222       9.457 -31.735  33.374  1.00 28.10           C
ATOM   1687  CD1 PHE A 222       8.780 -30.647  32.845  1.00 28.25           C
ATOM   1688  CD2 PHE A 222       9.776 -32.794  32.533  1.00 24.84           C
ATOM   1689  CE1 PHE A 222       8.419 -30.616  31.494  1.00 30.36           C
ATOM   1690  CE2 PHE A 222       9.426 -32.771  31.191  1.00 29.01           C
ATOM   1691  CZ  PHE A 222       8.742 -31.677  30.670  1.00 27.17           C
ATOM   1692  N   HIS A 223      12.700 -32.645  34.473  1.00 28.47           N
ATOM   1693  CA  HIS A 223      13.957 -33.090  33.894  1.00 31.69           C
ATOM   1694  C   HIS A 223      13.828 -34.330  33.010  1.00 29.60           C
ATOM   1695  O   HIS A 223      14.529 -34.454  32.007  1.00 37.05           O
ATOM   1696  CB  HIS A 223      14.990 -33.259  35.016  1.00 29.79           C
ATOM   1697  CG  HIS A 223      15.061 -32.062  35.912  1.00 45.46           C
ATOM   1698  ND1 HIS A 223      14.280 -31.930  37.044  1.00 40.67           N
ATOM   1699  CD2 HIS A 223      15.750 -30.900  35.793  1.00 35.56           C
ATOM   1700  CE1 HIS A 223      14.513 -30.755  37.601  1.00 40.14           C
ATOM   1701  NE2 HIS A 223      15.405 -30.113  36.864  1.00 43.12           N
ATOM   1702  N   ARG A 224      12.926 -35.236  33.370  1.00 29.77           N
ATOM   1703  CA  ARG A 224      12.688 -36.435  32.566  1.00 27.23           C
ATOM   1704  C   ARG A 224      11.204 -36.674  32.383  1.00 24.10           C
ATOM   1705  O   ARG A 224      10.386 -36.159  33.145  1.00 26.13           O
ATOM   1706  CB  ARG A 224      13.308 -37.660  33.223  1.00 30.12           C
ATOM   1707  CG  ARG A 224      14.802 -37.650  33.246  1.00 33.12           C
ATOM   1708  CD  ARG A 224      15.323 -38.643  34.271  1.00 36.19           C
ATOM   1709  NE  ARG A 224      16.742 -38.419  34.531  1.00 51.26           N
```

```
ATOM   1710  CZ  ARG A 224      17.205 -37.529  35.404  1.00 55.21           C
ATOM   1711  NH1 ARG A 224      16.359 -36.777  36.111  1.00 47.85           N
ATOM   1712  NH2 ARG A 224      18.517 -37.392  35.571  1.00 63.45           N
ATOM   1713  N   ALA A 225      10.865 -37.482  31.387  1.00 25.41           N
ATOM   1714  CA  ALA A 225       9.473 -37.697  31.033  1.00 24.82           C
ATOM   1715  C   ALA A 225       9.137 -39.168  30.781  1.00 24.15           C
ATOM   1716  O   ALA A 225       9.933 -39.910  30.205  1.00 25.74           O
ATOM   1717  CB  ALA A 225       9.111 -36.853  29.823  1.00 21.94           C
ATOM   1718  N   VAL A 226       7.951 -39.577  31.223  1.00 23.69           N
ATOM   1719  CA  VAL A 226       7.438 -40.913  30.963  1.00 21.01           C
ATOM   1720  C   VAL A 226       6.061 -40.776  30.347  1.00 25.43           C
ATOM   1721  O   VAL A 226       5.182 -40.152  30.945  1.00 24.61           O
ATOM   1722  CB  VAL A 226       7.295 -41.740  32.259  1.00 21.84           C
ATOM   1723  CG1 VAL A 226       6.736 -43.133  31.955  1.00 17.34           C
ATOM   1724  CG2 VAL A 226       8.623 -41.829  32.998  1.00 21.56           C
ATOM   1725  N   LEU A 227       5.876 -41.356  29.160  1.00 18.91           N
ATOM   1726  CA  LEU A 227       4.595 -41.301  28.471  1.00 20.51           C
ATOM   1727  C   LEU A 227       4.004 -42.692  28.289  1.00 24.42           C
ATOM   1728  O   LEU A 227       4.478 -43.478  27.456  1.00 26.58           O
ATOM   1729  CB  LEU A 227       4.737 -40.620  27.112  1.00 21.42           C
ATOM   1730  CG  LEU A 227       4.964 -39.112  27.120  1.00 23.98           C
ATOM   1731  CD1 LEU A 227       6.417 -38.782  27.344  1.00 23.66           C
ATOM   1732  CD2 LEU A 227       4.465 -38.510  25.816  1.00 27.24           C
ATOM   1733  N   GLN A 228       2.962 -42.990  29.059  1.00 22.02           N
ATOM   1734  CA  GLN A 228       2.325 -44.304  29.021  1.00 23.82           C
ATOM   1735  C   GLN A 228       1.033 -44.258  28.198  1.00 23.00           C
ATOM   1736  O   GLN A 228       0.072 -43.573  28.579  1.00 19.83           O
ATOM   1737  CB  GLN A 228       2.021 -44.791  30.451  1.00 19.01           C
ATOM   1738  CG  GLN A 228       3.251 -44.841  31.361  1.00 20.54           C
ATOM   1739  CD  GLN A 228       2.925 -45.164  32.813  1.00 23.77           C
ATOM   1740  OE1 GLN A 228       3.687 -44.821  33.719  1.00 26.24           O
ATOM   1741  NE2 GLN A 228       1.798 -45.828  33.042  1.00 20.98           N
ATOM   1742  N   SER A 229       1.015 -44.987  27.079  1.00 22.61           N
ATOM   1743  CA  SER A 229      -0.201 -45.156  26.266  1.00 19.15           C
ATOM   1744  C   SER A 229      -0.809 -43.825  25.850  1.00 18.53           C
ATOM   1745  O   SER A 229      -2.021 -43.637  25.922  1.00 23.59           O
ATOM   1746  CB  SER A 229      -1.261 -45.974  27.021  1.00 17.97           C
ATOM   1747  OG  SER A 229      -0.832 -47.306  27.249  1.00 19.68           O
ATOM   1748  N   GLY A 230       0.027 -42.885  25.440  1.00 15.93           N
ATOM   1749  CA  GLY A 230      -0.495 -41.587  25.064  1.00 20.78           C
ATOM   1750  C   GLY A 230       0.601 -40.679  24.566  1.00 23.83           C
ATOM   1751  O   GLY A 230       1.770 -40.873  24.894  1.00 22.17           O
ATOM   1752  N   ALA A 231       0.217 -39.697  23.761  1.00 17.39           N
ATOM   1753  CA  ALA A 231       1.165 -38.792  23.153  1.00 24.73           C
```

```
ATOM   1754  C   ALA A 231       0.425 -37.520  22.770  1.00 23.26           C
ATOM   1755  O   ALA A 231      -0.754 -37.568  22.447  1.00 24.81           O
ATOM   1756  CB  ALA A 231       1.814 -39.450  21.931  1.00 20.06           C
ATOM   1757  N   PRO A 232       1.113 -36.374  22.820  1.00 22.85           N
ATOM   1758  CA  PRO A 232       0.480 -35.093  22.486  1.00 22.54           C
ATOM   1759  C   PRO A 232       0.042 -35.016  21.026  1.00 26.55           C
ATOM   1760  O   PRO A 232      -0.850 -34.222  20.695  1.00 26.09           O
ATOM   1761  CB  PRO A 232       1.587 -34.060  22.777  1.00 23.04           C
ATOM   1762  CG  PRO A 232       2.876 -34.842  22.740  1.00 19.68           C
ATOM   1763  CD  PRO A 232       2.522 -36.218  23.226  1.00 20.79           C
ATOM   1764  N   ASN A 233       0.661 -35.825  20.169  1.00 27.72           N
ATOM   1765  CA  ASN A 233       0.337 -35.831  18.744  1.00 27.94           C
ATOM   1766  C   ASN A 233      -0.800 -36.793  18.391  1.00 29.49           C
ATOM   1767  O   ASN A 233      -1.177 -36.928  17.223  1.00 28.32           O
ATOM   1768  CB  ASN A 233       1.580 -36.127  17.893  1.00 23.40           C
ATOM   1769  CG  ASN A 233       2.361 -37.329  18.392  1.00 22.31           C
ATOM   1770  OD1 ASN A 233       2.768 -37.370  19.547  1.00 27.33           O
ATOM   1771  ND2 ASN A 233       2.548 -38.324  17.533  1.00 21.42           N
ATOM   1772  N   GLY A 234      -1.363 -37.457  19.395  1.00 24.31           N
ATOM   1773  CA  GLY A 234      -2.566 -38.250  19.154  1.00 39.31           C
ATOM   1774  C   GLY A 234      -3.712 -37.445  18.532  1.00 41.70           C
ATOM   1775  O   GLY A 234      -3.952 -36.296  18.932  1.00 40.00           O
ATOM   1776  N   PRO A 235      -4.438 -38.044  17.563  1.00 31.73           N
ATOM   1777  CA  PRO A 235      -5.580 -37.408  16.882  1.00 22.65           C
ATOM   1778  C   PRO A 235      -6.712 -36.988  17.835  1.00 25.71           C
ATOM   1779  O   PRO A 235      -7.572 -36.193  17.459  1.00 32.78           O
ATOM   1780  CB  PRO A 235      -6.081 -38.506  15.940  1.00 19.87           C
ATOM   1781  CG  PRO A 235      -5.653 -39.782  16.603  1.00 27.35           C
ATOM   1782  CD  PRO A 235      -4.313 -39.467  17.204  1.00 26.00           C
ATOM   1783  N   TRP A 236      -6.709 -37.506  19.055  1.00 23.29           N
ATOM   1784  CA  TRP A 236      -7.689 -37.098  20.059  1.00 24.88           C
ATOM   1785  C   TRP A 236      -7.192 -36.013  21.025  1.00 25.87           C
ATOM   1786  O   TRP A 236      -8.004 -35.375  21.705  1.00 25.72           O
ATOM   1787  CB  TRP A 236      -8.109 -38.313  20.876  1.00 23.50           C
ATOM   1788  CG  TRP A 236      -6.932 -39.054  21.405  1.00 25.15           C
ATOM   1789  CD1 TRP A 236      -6.260 -40.069  20.785  1.00 23.19           C
ATOM   1790  CD2 TRP A 236      -6.263 -38.830  22.654  1.00 20.13           C
ATOM   1791  NE1 TRP A 236      -5.221 -40.494  21.571  1.00 25.00           N
ATOM   1792  CE2 TRP A 236      -5.200 -39.753  22.726  1.00 22.86           C
ATOM   1793  CE3 TRP A 236      -6.462 -37.941  23.720  1.00 25.16           C
ATOM   1794  CZ2 TRP A 236      -4.332 -39.819  23.824  1.00 20.80           C
ATOM   1795  CZ3 TRP A 236      -5.604 -38.011  24.819  1.00 20.96           C
ATOM   1796  CH2 TRP A 236      -4.552 -38.946  24.859  1.00 24.81           C
ATOM   1797  N   ALA A 237      -5.875 -35.812  21.094  1.00 24.38           N
```

```
ATOM   1798  CA  ALA A 237      -5.271 -35.015  22.179  1.00 26.50           C
ATOM   1799  C   ALA A 237      -5.281 -33.500  21.963  1.00 25.74           C
ATOM   1800  O   ALA A 237      -5.074 -32.733  22.904  1.00 23.31           O
ATOM   1801  CB  ALA A 237      -3.847 -35.497  22.482  1.00 22.07           C
ATOM   1802  N   THR A 238      -5.496 -33.066  20.726  1.00 23.24           N
ATOM   1803  CA  THR A 238      -5.653 -31.640  20.457  1.00 23.58           C
ATOM   1804  C   THR A 238      -6.810 -31.386  19.504  1.00 24.76           C
ATOM   1805  O   THR A 238      -7.292 -32.292  18.825  1.00 21.78           O
ATOM   1806  CB  THR A 238      -4.373 -30.994  19.867  1.00 25.12           C
ATOM   1807  OG1 THR A 238      -4.022 -31.652  18.643  1.00 21.53           O
ATOM   1808  CG2 THR A 238      -3.214 -31.094  20.852  1.00 21.89           C
ATOM   1809  N   VAL A 239      -7.252 -30.141  19.462  1.00 23.54           N
ATOM   1810  CA  VAL A 239      -8.286 -29.741  18.524  1.00 26.73           C
ATOM   1811  C   VAL A 239      -7.891 -28.387  17.927  1.00 28.32           C
ATOM   1812  O   VAL A 239      -7.179 -27.602  18.573  1.00 26.17           O
ATOM   1813  CB  VAL A 239      -9.676 -29.686  19.212  1.00 26.08           C
ATOM   1814  CG1 VAL A 239      -9.764 -28.516  20.211  1.00 22.06           C
ATOM   1815  CG2 VAL A 239     -10.763 -29.588  18.192  1.00 30.33           C
ATOM   1816  N   GLY A 240      -8.318 -28.127  16.689  1.00 28.90           N
ATOM   1817  CA  GLY A 240      -8.033 -26.860  16.038  1.00 23.29           C
ATOM   1818  C   GLY A 240      -8.874 -25.728  16.607  1.00 32.93           C
ATOM   1819  O   GLY A 240      -9.912 -25.976  17.239  1.00 29.27           O
ATOM   1820  N   MET A 241      -8.424 -24.491  16.391  1.00 32.83           N
ATOM   1821  CA  MET A 241      -9.133 -23.309  16.883  1.00 32.56           C
ATOM   1822  C   MET A 241     -10.577 -23.249  16.385  1.00 34.99           C
ATOM   1823  O   MET A 241     -11.506 -23.018  17.169  1.00 33.14           O
ATOM   1824  CB  MET A 241      -8.405 -22.032  16.465  1.00 34.59           C
ATOM   1825  CG  MET A 241      -7.134 -21.754  17.227  1.00 38.47           C
ATOM   1826  SD  MET A 241      -6.380 -20.195  16.703  1.00 49.09           S
ATOM   1827  CE  MET A 241      -6.073 -20.555  14.974  1.00 40.60           C
ATOM   1828  N   GLY A 242     -10.755 -23.457  15.081  1.00 33.86           N
ATOM   1829  CA  GLY A 242     -12.071 -23.443  14.466  1.00 27.42           C
ATOM   1830  C   GLY A 242     -13.051 -24.407  15.121  1.00 33.65           C
ATOM   1831  O   GLY A 242     -14.164 -24.025  15.483  1.00 32.26           O
ATOM   1832  N   GLU A 243     -12.628 -25.657  15.286  1.00 30.49           N
ATOM   1833  CA  GLU A 243     -13.480 -26.686  15.863  1.00 27.82           C
ATOM   1834  C   GLU A 243     -13.697 -26.476  17.372  1.00 34.86           C
ATOM   1835  O   GLU A 243     -14.773 -26.777  17.890  1.00 33.61           O
ATOM   1836  CB  GLU A 243     -12.908 -28.078  15.560  1.00 26.00           C
ATOM   1837  CG  GLU A 243     -13.644 -29.261  16.200  1.00 27.00           C
ATOM   1838  CD  GLU A 243     -15.042 -29.464  15.650  1.00 33.40           C
ATOM   1839  OE1 GLU A 243     -15.393 -28.784  14.664  1.00 39.97           O
ATOM   1840  OE2 GLU A 243     -15.793 -30.304  16.201  1.00 31.18           O
ATOM   1841  N   ALA A 244     -12.691 -25.956  18.075  1.00 31.11           N
```

```
ATOM   1842  CA  ALA A 244     -12.866 -25.642  19.494  1.00 28.48           C
ATOM   1843  C   ALA A 244     -13.965 -24.605  19.654  1.00 31.19           C
ATOM   1844  O   ALA A 244     -14.820 -24.734  20.530  1.00 27.55           O
ATOM   1845  CB  ALA A 244     -11.577 -25.132  20.110  1.00 27.75           C
ATOM   1846  N   ARG A 245     -13.942 -23.585  18.794  1.00 31.51           N
ATOM   1847  CA  ARG A 245     -14.944 -22.525  18.828  1.00 30.37           C
ATOM   1848  C   ARG A 245     -16.325 -23.075  18.523  1.00 29.80           C
ATOM   1849  O   ARG A 245     -17.311 -22.694  19.150  1.00 32.55           O
ATOM   1850  CB  ARG A 245     -14.612 -21.399  17.842  1.00 30.24           C
ATOM   1851  CG  ARG A 245     -15.741 -20.378  17.694  1.00 28.78           C
ATOM   1852  CD  ARG A 245     -15.287 -19.123  16.952  1.00 25.30           C
ATOM   1853  NE  ARG A 245     -14.302 -18.372  17.720  1.00 29.68           N
ATOM   1854  CZ  ARG A 245     -14.612 -17.503  18.677  1.00 32.73           C
ATOM   1855  NH1 ARG A 245     -15.884 -17.283  18.990  1.00 34.23           N
ATOM   1856  NH2 ARG A 245     -13.654 -16.858  19.326  1.00 30.01           N
ATOM   1857  N   ARG A 246     -16.397 -23.987  17.566  1.00 28.84           N
ATOM   1858  CA  ARG A 246     -17.687 -24.547  17.215  1.00 38.52           C
ATOM   1859  C   ARG A 246     -18.268 -25.338  18.382  1.00 31.98           C
ATOM   1860  O   ARG A 246     -19.444 -25.199  18.705  1.00 27.93           O
ATOM   1861  CB  ARG A 246     -17.601 -25.432  15.977  1.00 30.00           C
ATOM   1862  CG  ARG A 246     -18.975 -25.795  15.449  1.00 33.64           C
ATOM   1863  CD  ARG A 246     -18.947 -27.029  14.578  1.00 37.66           C
ATOM   1864  NE  ARG A 246     -18.481 -28.215  15.293  1.00 37.55           N
ATOM   1865  CZ  ARG A 246     -19.240 -28.945  16.106  1.00 35.58           C
ATOM   1866  NH1 ARG A 246     -20.502 -28.599  16.338  1.00 38.99           N
ATOM   1867  NH2 ARG A 246     -18.733 -30.016  16.697  1.00 28.18           N
ATOM   1868  N   ARG A 247     -17.433 -26.169  18.999  1.00 36.20           N
ATOM   1869  CA  ARG A 247     -17.860 -27.009  20.111  1.00 32.90           C
ATOM   1870  C   ARG A 247     -18.295 -26.184  21.313  1.00 27.90           C
ATOM   1871  O   ARG A 247     -19.280 -26.517  21.975  1.00 29.59           O
ATOM   1872  CB  ARG A 247     -16.747 -27.965  20.514  1.00 33.98           C
ATOM   1873  CG  ARG A 247     -16.510 -29.064  19.496  1.00 33.61           C
ATOM   1874  CD  ARG A 247     -15.437 -30.005  19.985  1.00 29.17           C
ATOM   1875  NE  ARG A 247     -14.993 -30.889  18.921  1.00 26.50           N
ATOM   1876  CZ  ARG A 247     -14.099 -31.847  19.092  1.00 28.59           C
ATOM   1877  NH1 ARG A 247     -13.562 -32.034  20.294  1.00 24.41           N
ATOM   1878  NH2 ARG A 247     -13.746 -32.614  18.065  1.00 26.25           N
ATOM   1879  N   ALA A 248     -17.563 -25.106  21.579  1.00 29.19           N
ATOM   1880  CA  ALA A 248     -17.895 -24.202  22.676  1.00 31.39           C
ATOM   1881  C   ALA A 248     -19.220 -23.525  22.375  1.00 36.90           C
ATOM   1882  O   ALA A 248     -20.063 -23.353  23.261  1.00 35.05           O
ATOM   1883  CB  ALA A 248     -16.799 -23.154  22.863  1.00 23.03           C
ATOM   1884  N   THR A 249     -19.402 -23.149  21.111  1.00 28.81           N
ATOM   1885  CA  THR A 249     -20.601 -22.436  20.705  1.00 28.17           C
```

ATOM	1886	C	THR	A	249	-21.819	-23.349	20.757	1.00	28.49	C
ATOM	1887	O	THR	A	249	-22.890	-22.926	21.184	1.00	29.08	O
ATOM	1888	CB	THR	A	249	-20.437	-21.784	19.319	1.00	39.46	C
ATOM	1889	OG1	THR	A	249	-19.357	-20.843	19.372	1.00	38.83	O
ATOM	1890	CG2	THR	A	249	-21.716	-21.050	18.909	1.00	34.47	C
ATOM	1891	N	GLN	A	250	-21.648	-24.607	20.359	1.00	27.63	N
ATOM	1892	CA	GLN	A	250	-22.745	-25.562	20.439	1.00	28.63	C
ATOM	1893	C	GLN	A	250	-23.111	-25.898	21.880	1.00	31.94	C
ATOM	1894	O	GLN	A	250	-24.277	-26.164	22.182	1.00	32.14	O
ATOM	1895	CB	GLN	A	250	-22.440	-26.859	19.684	1.00	26.22	C
ATOM	1896	CG	GLN	A	250	-23.709	-27.687	19.470	1.00	33.51	C
ATOM	1897	CD	GLN	A	250	-23.455	-29.058	18.885	1.00	45.75	C
ATOM	1898	OE1	GLN	A	250	-22.408	-29.317	18.276	1.00	51.28	O
ATOM	1899	NE2	GLN	A	250	-24.420	-29.952	19.063	1.00	41.59	N
ATOM	1900	N	LEU	A	251	-22.119	-25.910	22.768	1.00	31.00	N
ATOM	1901	CA	LEU	A	251	-22.409	-26.165	24.174	1.00	35.41	C
ATOM	1902	C	LEU	A	251	-23.253	-25.028	24.725	1.00	30.24	C
ATOM	1903	O	LEU	A	251	-24.200	-25.264	25.466	1.00	28.35	O
ATOM	1904	CB	LEU	A	251	-21.139	-26.330	25.014	1.00	29.75	C
ATOM	1905	CG	LEU	A	251	-21.411	-26.693	26.480	1.00	30.41	C
ATOM	1906	CD1	LEU	A	251	-21.999	-28.104	26.598	1.00	22.37	C
ATOM	1907	CD2	LEU	A	251	-20.164	-26.548	27.351	1.00	29.82	C
ATOM	1908	N	ALA	A	252	-22.916	-23.798	24.351	1.00	31.40	N
ATOM	1909	CA	ALA	A	252	-23.669	-22.653	24.845	1.00	35.39	C
ATOM	1910	C	ALA	A	252	-25.115	-22.778	24.394	1.00	38.42	C
ATOM	1911	O	ALA	A	252	-26.037	-22.633	25.198	1.00	35.04	O
ATOM	1912	CB	ALA	A	252	-23.054	-21.336	24.381	1.00	26.33	C
ATOM	1913	N	HIS	A	253	-25.302	-23.090	23.113	1.00	36.18	N
ATOM	1914	CA	HIS	A	253	-26.640	-23.300	22.568	1.00	38.15	C
ATOM	1915	C	HIS	A	253	-27.430	-24.387	23.312	1.00	33.08	C
ATOM	1916	O	HIS	A	253	-28.600	-24.195	23.636	1.00	36.39	O
ATOM	1917	CB	HIS	A	253	-26.582	-23.625	21.067	1.00	37.06	C
ATOM	1918	CG	HIS	A	253	-27.920	-23.948	20.474	1.00	46.47	C
ATOM	1919	ND1	HIS	A	253	-28.380	-25.241	20.332	1.00	46.39	N
ATOM	1920	CD2	HIS	A	253	-28.908	-23.145	20.009	1.00	49.04	C
ATOM	1921	CE1	HIS	A	253	-29.588	-25.221	19.797	1.00	47.51	C
ATOM	1922	NE2	HIS	A	253	-29.931	-23.962	19.591	1.00	55.79	N
ATOM	1923	N	LEU	A	254	-26.787	-25.520	23.577	1.00	31.13	N
ATOM	1924	CA	LEU	A	254	-27.450	-26.653	24.220	1.00	28.49	C
ATOM	1925	C	LEU	A	254	-27.881	-26.390	25.662	1.00	34.16	C
ATOM	1926	O	LEU	A	254	-28.754	-27.085	26.186	1.00	35.71	O
ATOM	1927	CB	LEU	A	254	-26.558	-27.896	24.184	1.00	31.68	C
ATOM	1928	CG	LEU	A	254	-26.323	-28.573	22.832	1.00	39.90	C
ATOM	1929	CD1	LEU	A	254	-25.276	-29.674	22.974	1.00	33.30	C

ATOM	1930	CD2	LEU	A	254	-27.636	-29.132	22.289	1.00	29.11	C
ATOM	1931	N	VAL	A	255	-27.261	-25.408	26.309	1.00	32.47	N
ATOM	1932	CA	VAL	A	255	-27.612	-25.078	27.686	1.00	31.34	C
ATOM	1933	C	VAL	A	255	-28.302	-23.712	27.796	1.00	36.46	C
ATOM	1934	O	VAL	A	255	-28.388	-23.131	28.885	1.00	33.49	O
ATOM	1935	CB	VAL	A	255	-26.393	-25.149	28.632	1.00	29.29	C
ATOM	1936	CG1	VAL	A	255	-25.708	-26.496	28.495	1.00	27.72	C
ATOM	1937	CG2	VAL	A	255	-25.425	-24.004	28.364	1.00	26.70	C
ATOM	1938	N	GLY	A	256	-28.798	-23.213	26.663	1.00	29.86	N
ATOM	1939	CA	GLY	A	256	-29.640	-22.030	26.660	1.00	31.66	C
ATOM	1940	C	GLY	A	256	-28.937	-20.687	26.660	1.00	31.79	C
ATOM	1941	O	GLY	A	256	-29.554	-19.668	26.966	1.00	32.31	O
ATOM	1942	N	CYS	A	257	-27.659	-20.672	26.292	1.00	35.91	N
ATOM	1943	CA	CYS	A	257	-26.896	-19.427	26.221	1.00	37.16	C
ATOM	1944	C	CYS	A	257	-26.729	-18.942	24.782	1.00	41.86	C
ATOM	1945	O	CYS	A	257	-26.718	-19.747	23.857	1.00	44.43	O
ATOM	1946	CB	CYS	A	257	-25.532	-19.610	26.891	1.00	36.38	C
ATOM	1947	SG	CYS	A	257	-25.676	-19.935	28.655	1.00	36.74	S
ATOM	1948	N	PRO	A	258	-26.598	-17.619	24.593	1.00	49.86	N
ATOM	1949	CA	PRO	A	258	-26.408	-17.033	23.260	1.00	51.14	C
ATOM	1950	C	PRO	A	258	-25.035	-17.347	22.667	1.00	51.83	C
ATOM	1951	O	PRO	A	258	-24.894	-17.340	21.438	1.00	71.72	O
ATOM	1952	CB	PRO	A	258	-26.526	-15.531	23.525	1.00	51.45	C
ATOM	1953	CG	PRO	A	258	-26.113	-15.370	24.959	1.00	47.94	C
ATOM	1954	CD	PRO	A	258	-26.646	-16.587	25.647	1.00	47.82	C
ATOM	1955	N	ASN	A	265	-20.581	-12.339	20.298	1.00	55.41	N
ATOM	1956	CA	ASN	A	265	-19.863	-11.725	21.411	1.00	50.12	C
ATOM	1957	C	ASN	A	265	-19.414	-12.763	22.449	1.00	53.70	C
ATOM	1958	O	ASN	A	265	-20.218	-13.262	23.241	1.00	51.66	O
ATOM	1959	CB	ASN	A	265	-20.725	-10.637	22.056	1.00	58.06	C
ATOM	1960	CG	ASN	A	265	-19.933	-9.727	22.986	1.00	63.59	C
ATOM	1961	OD1	ASN	A	265	-18.993	-10.162	23.648	1.00	66.72	O
ATOM	1962	ND2	ASN	A	265	-20.321	-8.453	23.044	1.00	68.68	N
ATOM	1963	N	ASP	A	266	-18.121	-13.076	22.440	1.00	46.74	N
ATOM	1964	CA	ASP	A	266	-17.560	-14.072	23.345	1.00	44.11	C
ATOM	1965	C	ASP	A	266	-17.824	-13.743	24.824	1.00	49.38	C
ATOM	1966	O	ASP	A	266	-18.161	-14.633	25.616	1.00	43.46	O
ATOM	1967	CB	ASP	A	266	-16.052	-14.225	23.110	1.00	42.99	C
ATOM	1968	CG	ASP	A	266	-15.714	-14.788	21.732	1.00	39.96	C
ATOM	1969	OD1	ASP	A	266	-16.539	-15.515	21.147	1.00	39.30	O
ATOM	1970	OD2	ASP	A	266	-14.603	-14.508	21.240	1.00	49.48	O
ATOM	1971	N	THR	A	267	-17.666	-12.471	25.188	1.00	40.97	N
ATOM	1972	CA	THR	A	267	-17.893	-12.027	26.561	1.00	38.54	C
ATOM	1973	C	THR	A	267	-19.263	-12.457	27.096	1.00	42.88	C

ATOM	1974	O	THR	A	267	-19.360	-13.011	28.196	1.00	43.48	O
ATOM	1975	CB	THR	A	267	-17.765	-10.499	26.690	1.00	47.27	C
ATOM	1976	OG1	THR	A	267	-16.505	-10.074	26.157	1.00	50.74	O
ATOM	1977	CG2	THR	A	267	-17.865	-10.069	28.155	1.00	43.24	C
ATOM	1978	N	GLU	A	268	-20.312	-12.210	26.313	1.00	45.62	N
ATOM	1979	CA	GLU	A	268	-21.675	-12.562	26.707	1.00	43.71	C
ATOM	1980	C	GLU	A	268	-21.853	-14.068	26.791	1.00	42.01	C
ATOM	1981	O	GLU	A	268	-22.501	-14.586	27.709	1.00	42.76	O
ATOM	1982	CB	GLU	A	268	-22.689	-12.003	25.713	1.00	43.94	C
ATOM	1983	CG	GLU	A	268	-22.824	-10.492	25.719	1.00	64.17	C
ATOM	1984	CD	GLU	A	268	-23.896	-10.008	24.749	1.00	87.59	C
ATOM	1985	OE1	GLU	A	268	-24.858	-10.772	24.491	1.00	84.71	O
ATOM	1986	OE2	GLU	A	268	-23.769	-8.871	24.240	1.00	90.79	O
ATOM	1987	N	LEU	A	269	-21.282	-14.762	25.816	1.00	39.77	N
ATOM	1988	CA	LEU	A	269	-21.390	-16.210	25.732	1.00	39.41	C
ATOM	1989	C	LEU	A	269	-20.791	-16.857	26.985	1.00	34.90	C
ATOM	1990	O	LEU	A	269	-21.435	-17.677	27.635	1.00	39.60	O
ATOM	1991	CB	LEU	A	269	-20.717	-16.714	24.443	1.00	35.72	C
ATOM	1992	CG	LEU	A	269	-20.946	-18.150	23.964	1.00	39.66	C
ATOM	1993	CD1	LEU	A	269	-20.628	-18.279	22.482	1.00	40.94	C
ATOM	1994	CD2	LEU	A	269	-20.090	-19.132	24.750	1.00	40.14	C
ATOM	1995	N	VAL	A	270	-19.567	-16.475	27.333	1.00	34.23	N
ATOM	1996	CA	VAL	A	270	-18.921	-17.036	28.514	1.00	39.46	C
ATOM	1997	C	VAL	A	270	-19.651	-16.635	29.802	1.00	37.00	C
ATOM	1998	O	VAL	A	270	-19.777	-17.438	30.729	1.00	34.61	O
ATOM	1999	CB	VAL	A	270	-17.431	-16.642	28.592	1.00	42.61	C
ATOM	2000	CG1	VAL	A	270	-16.775	-17.258	29.817	1.00	35.28	C
ATOM	2001	CG2	VAL	A	270	-16.703	-17.087	27.330	1.00	42.78	C
ATOM	2002	N	ALA	A	271	-20.138	-15.400	29.849	1.00	33.48	N
ATOM	2003	CA	ALA	A	271	-20.826	-14.905	31.035	1.00	36.74	C
ATOM	2004	C	ALA	A	271	-22.095	-15.714	31.296	1.00	35.70	C
ATOM	2005	O	ALA	A	271	-22.411	-16.041	32.438	1.00	39.78	O
ATOM	2006	CB	ALA	A	271	-21.152	-13.424	30.892	1.00	31.07	C
ATOM	2007	N	CYS	A	272	-22.815	-16.045	30.232	1.00	37.01	N
ATOM	2008	CA	CYS	A	272	-23.983	-16.899	30.361	1.00	35.60	C
ATOM	2009	C	CYS	A	272	-23.590	-18.320	30.785	1.00	32.05	C
ATOM	2010	O	CYS	A	272	-24.254	-18.919	31.635	1.00	35.48	O
ATOM	2011	CB	CYS	A	272	-24.793	-16.919	29.059	1.00	37.02	C
ATOM	2012	SG	CYS	A	272	-26.238	-18.006	29.127	1.00	40.95	S
ATOM	2013	N	LEU	A	273	-22.521	-18.855	30.196	1.00	29.52	N
ATOM	2014	CA	LEU	A	273	-21.999	-20.166	30.598	1.00	31.53	C
ATOM	2015	C	LEU	A	273	-21.621	-20.194	32.081	1.00	34.00	C
ATOM	2016	O	LEU	A	273	-21.840	-21.195	32.772	1.00	31.46	O
ATOM	2017	CB	LEU	A	273	-20.776	-20.550	29.767	1.00	25.65	C

```
ATOM   2018  CG  LEU A 273     -20.996 -20.974  28.313  1.00 31.64           C
ATOM   2019  CD1 LEU A 273     -19.658 -21.246  27.648  1.00 28.58           C
ATOM   2020  CD2 LEU A 273     -21.890 -22.207  28.218  1.00 27.01           C
ATOM   2021  N   ARG A 274     -21.060 -19.091  32.570  1.00 31.47           N
ATOM   2022  CA  ARG A 274     -20.639 -19.025  33.966  1.00 36.88           C
ATOM   2023  C   ARG A 274     -21.810 -19.091  34.954  1.00 32.63           C
ATOM   2024  O   ARG A 274     -21.609 -19.405  36.119  1.00 27.59           O
ATOM   2025  CB  ARG A 274     -19.757 -17.796  34.231  1.00 32.84           C
ATOM   2026  CG  ARG A 274     -18.362 -17.910  33.632  1.00 35.67           C
ATOM   2027  CD  ARG A 274     -17.367 -16.976  34.304  1.00 34.38           C
ATOM   2028  NE  ARG A 274     -16.993 -15.844  33.458  1.00 50.29           N
ATOM   2029  CZ  ARG A 274     -15.815 -15.714  32.845  1.00 50.27           C
ATOM   2030  NH1 ARG A 274     -14.876 -16.647  32.973  1.00 40.60           N
ATOM   2031  NH2 ARG A 274     -15.570 -14.640  32.105  1.00 47.98           N
ATOM   2032  N   THR A 275     -23.027 -18.823  34.488  1.00 32.00           N
ATOM   2033  CA  THR A 275     -24.190 -18.851  35.378  1.00 30.23           C
ATOM   2034  C   THR A 275     -24.767 -20.256  35.501  1.00 34.45           C
ATOM   2035  O   THR A 275     -25.651 -20.505  36.315  1.00 38.79           O
ATOM   2036  CB  THR A 275     -25.322 -17.926  34.890  1.00 37.83           C
ATOM   2037  OG1 THR A 275     -25.919 -18.481  33.711  1.00 40.74           O
ATOM   2038  CG2 THR A 275     -24.803 -16.525  34.593  1.00 38.98           C
ATOM   2039  N   ARG A 276     -24.271 -21.180  34.691  1.00 26.87           N
ATOM   2040  CA  ARG A 276     -24.842 -22.513  34.667  1.00 24.97           C
ATOM   2041  C   ARG A 276     -24.202 -23.399  35.729  1.00 30.39           C
ATOM   2042  O   ARG A 276     -22.997 -23.314  35.967  1.00 29.98           O
ATOM   2043  CB  ARG A 276     -24.661 -23.130  33.278  1.00 29.51           C
ATOM   2044  CG  ARG A 276     -25.428 -22.412  32.177  1.00 28.92           C
ATOM   2045  CD  ARG A 276     -26.913 -22.594  32.371  1.00 33.03           C
ATOM   2046  NE  ARG A 276     -27.701 -22.127  31.235  1.00 32.87           N
ATOM   2047  CZ  ARG A 276     -28.236 -20.912  31.144  1.00 38.01           C
ATOM   2048  NH1 ARG A 276     -28.056 -20.027  32.117  1.00 38.64           N
ATOM   2049  NH2 ARG A 276     -28.947 -20.578  30.077  1.00 34.54           N
ATOM   2050  N   PRO A 277     -25.012 -24.232  36.395  1.00 30.00           N
ATOM   2051  CA  PRO A 277     -24.432 -25.218  37.308  1.00 28.67           C
ATOM   2052  C   PRO A 277     -23.463 -26.109  36.540  1.00 33.99           C
ATOM   2053  O   PRO A 277     -23.763 -26.480  35.403  1.00 31.93           O
ATOM   2054  CB  PRO A 277     -25.649 -26.038  37.757  1.00 23.85           C
ATOM   2055  CG  PRO A 277     -26.801 -25.093  37.625  1.00 29.45           C
ATOM   2056  CD  PRO A 277     -26.487 -24.243  36.420  1.00 28.32           C
ATOM   2057  N   ALA A 278     -22.327 -26.429  37.150  1.00 31.40           N
ATOM   2058  CA  ALA A 278     -21.328 -27.308  36.553  1.00 28.42           C
ATOM   2059  C   ALA A 278     -21.934 -28.547  35.908  1.00 28.93           C
ATOM   2060  O   ALA A 278     -21.587 -28.907  34.765  1.00 25.96           O
ATOM   2061  CB  ALA A 278     -20.298 -27.720  37.598  1.00 26.96           C
```

ATOM	2062	N	GLN	A	279	-22.840	-29.190	36.641	1.00	28.41	N
ATOM	2063	CA	GLN	A	279	-23.475	-30.427	36.186	1.00	30.33	C
ATOM	2064	C	GLN	A	279	-24.206	-30.255	34.847	1.00	33.06	C
ATOM	2065	O	GLN	A	279	-24.290	-31.191	34.046	1.00	29.64	O
ATOM	2066	CB	GLN	A	279	-24.437	-30.961	37.254	1.00	23.47	C
ATOM	2067	CG	GLN	A	279	-24.907	-32.399	37.007	1.00	23.74	C
ATOM	2068	CD	GLN	A	279	-23.750	-33.377	36.850	1.00	33.49	C
ATOM	2069	OE1	GLN	A	279	-22.853	-33.431	37.688	1.00	42.40	O
ATOM	2070	NE2	GLN	A	279	-23.768	-34.155	35.770	1.00	30.82	N
ATOM	2071	N	VAL	A	280	-24.735	-29.061	34.609	1.00	24.82	N
ATOM	2072	CA	VAL	A	280	-25.454	-28.803	33.376	1.00	33.54	C
ATOM	2073	C	VAL	A	280	-24.508	-28.851	32.173	1.00	31.38	C
ATOM	2074	O	VAL	A	280	-24.846	-29.428	31.140	1.00	33.93	O
ATOM	2075	CB	VAL	A	280	-26.234	-27.466	33.440	1.00	34.75	C
ATOM	2076	CG1	VAL	A	280	-26.623	-26.984	32.040	1.00	30.77	C
ATOM	2077	CG2	VAL	A	280	-27.462	-27.627	34.314	1.00	25.24	C
ATOM	2078	N	LEU	A	281	-23.318	-28.272	32.312	1.00	30.01	N
ATOM	2079	CA	LEU	A	281	-22.334	-28.346	31.234	1.00	30.05	C
ATOM	2080	C	LEU	A	281	-21.954	-29.801	30.986	1.00	27.76	C
ATOM	2081	O	LEU	A	281	-21.879	-30.251	29.842	1.00	33.36	O
ATOM	2082	CB	LEU	A	281	-21.087	-27.512	31.551	1.00	26.59	C
ATOM	2083	CG	LEU	A	281	-21.304	-26.053	31.966	1.00	28.04	C
ATOM	2084	CD1	LEU	A	281	-20.003	-25.268	31.900	1.00	27.45	C
ATOM	2085	CD2	LEU	A	281	-22.375	-25.376	31.132	1.00	24.18	C
ATOM	2086	N	VAL	A	282	-21.732	-30.539	32.065	1.00	24.60	N
ATOM	2087	CA	VAL	A	282	-21.397	-31.952	31.956	1.00	30.33	C
ATOM	2088	C	VAL	A	282	-22.473	-32.754	31.201	1.00	33.63	C
ATOM	2089	O	VAL	A	282	-22.152	-33.613	30.382	1.00	34.80	O
ATOM	2090	CB	VAL	A	282	-21.120	-32.575	33.348	1.00	23.95	C
ATOM	2091	CG1	VAL	A	282	-20.963	-34.077	33.250	1.00	24.10	C
ATOM	2092	CG2	VAL	A	282	-19.884	-31.961	33.951	1.00	23.86	C
ATOM	2093	N	ASN	A	283	-23.744	-32.457	31.459	1.00	32.75	N
ATOM	2094	CA	ASN	A	283	-24.842	-33.215	30.857	1.00	32.23	C
ATOM	2095	C	ASN	A	283	-24.951	-33.109	29.328	1.00	31.93	C
ATOM	2096	O	ASN	A	283	-25.569	-33.953	28.687	1.00	31.18	O
ATOM	2097	CB	ASN	A	283	-26.174	-32.838	31.510	1.00	30.33	C
ATOM	2098	CG	ASN	A	283	-26.283	-33.353	32.927	1.00	33.60	C
ATOM	2099	OD1	ASN	A	283	-25.520	-34.226	33.338	1.00	33.22	O
ATOM	2100	ND2	ASN	A	283	-27.230	-32.817	33.684	1.00	30.13	N
ATOM	2101	N	HIS	A	284	-24.357	-32.075	28.749	1.00	24.43	N
ATOM	2102	CA	HIS	A	284	-24.416	-31.905	27.308	1.00	33.00	C
ATOM	2103	C	HIS	A	284	-23.055	-32.094	26.632	1.00	34.33	C
ATOM	2104	O	HIS	A	284	-22.925	-31.848	25.441	1.00	34.18	O
ATOM	2105	CB	HIS	A	284	-24.994	-30.528	26.968	1.00	32.38	C

ATOM	2106	CG	HIS	A	284	-26.403	-30.347	27.432	1.00	42.07	C
ATOM	2107	ND1	HIS	A	284	-27.488	-30.745	26.680	1.00	44.72	N
ATOM	2108	CD2	HIS	A	284	-26.906	-29.843	28.584	1.00	31.89	C
ATOM	2109	CE1	HIS	A	284	-28.600	-30.480	27.342	1.00	38.32	C
ATOM	2110	NE2	HIS	A	284	-28.275	-29.931	28.499	1.00	40.01	N
ATOM	2111	N	GLU	A	285	-22.059	-32.542	27.394	1.00	34.17	N
ATOM	2112	CA	GLU	A	285	-20.674	-32.571	26.927	1.00	33.28	C
ATOM	2113	C	GLU	A	285	-20.484	-33.393	25.645	1.00	31.74	C
ATOM	2114	O	GLU	A	285	-19.929	-32.905	24.664	1.00	26.88	O
ATOM	2115	CB	GLU	A	285	-19.738	-33.102	28.025	1.00	28.94	C
ATOM	2116	CG	GLU	A	285	-18.272	-33.115	27.598	1.00	30.34	C
ATOM	2117	CD	GLU	A	285	-17.333	-33.711	28.646	1.00	34.90	C
ATOM	2118	OE1	GLU	A	285	-17.528	-33.466	29.854	1.00	33.45	O
ATOM	2119	OE2	GLU	A	285	-16.390	-34.425	28.255	1.00	30.99	O
ATOM	2120	N	TRP	A	286	-20.958	-34.634	25.668	1.00	26.72	N
ATOM	2121	CA	TRP	A	286	-20.742	-35.557	24.574	1.00	32.01	C
ATOM	2122	C	TRP	A	286	-21.473	-35.145	23.298	1.00	35.78	C
ATOM	2123	O	TRP	A	286	-21.116	-35.581	22.201	1.00	32.35	O
ATOM	2124	CB	TRP	A	286	-21.107	-36.977	25.009	1.00	26.15	C
ATOM	2125	CG	TRP	A	286	-20.213	-37.456	26.110	1.00	30.54	C
ATOM	2126	CD1	TRP	A	286	-20.468	-37.417	27.456	1.00	30.84	C
ATOM	2127	CD2	TRP	A	286	-18.899	-38.013	25.968	1.00	26.55	C
ATOM	2128	NE1	TRP	A	286	-19.401	-37.930	28.155	1.00	25.87	N
ATOM	2129	CE2	TRP	A	286	-18.423	-38.300	27.268	1.00	28.66	C
ATOM	2130	CE3	TRP	A	286	-18.079	-38.300	24.868	1.00	26.79	C
ATOM	2131	CZ2	TRP	A	286	-17.161	-38.859	27.499	1.00	23.83	C
ATOM	2132	CZ3	TRP	A	286	-16.829	-38.865	25.099	1.00	25.35	C
ATOM	2133	CH2	TRP	A	286	-16.382	-39.137	26.406	1.00	24.43	C
ATOM	2134	N	HIS	A	287	-22.454	-34.261	23.444	1.00	36.28	N
ATOM	2135	CA	HIS	A	287	-23.293	-33.846	22.326	1.00	34.94	C
ATOM	2136	C	HIS	A	287	-22.669	-32.815	21.396	1.00	34.99	C
ATOM	2137	O	HIS	A	287	-23.234	-32.526	20.349	1.00	44.11	O
ATOM	2138	CB	HIS	A	287	-24.658	-33.367	22.831	1.00	37.41	C
ATOM	2139	CG	HIS	A	287	-25.461	-34.449	23.488	1.00	49.46	C
ATOM	2140	ND1	HIS	A	287	-26.178	-34.249	24.652	1.00	54.02	N
ATOM	2141	CD2	HIS	A	287	-25.645	-35.748	23.150	1.00	46.20	C
ATOM	2142	CE1	HIS	A	287	-26.773	-35.377	24.999	1.00	52.51	C
ATOM	2143	NE2	HIS	A	287	-26.468	-36.302	24.103	1.00	59.58	N
ATOM	2144	N	VAL	A	288	-21.513	-32.258	21.746	1.00	36.90	N
ATOM	2145	CA	VAL	A	288	-20.858	-31.331	20.812	1.00	30.26	C
ATOM	2146	C	VAL	A	288	-19.875	-32.031	19.869	1.00	30.86	C
ATOM	2147	O	VAL	A	288	-19.295	-31.403	18.987	1.00	34.34	O
ATOM	2148	CB	VAL	A	288	-20.181	-30.128	21.508	1.00	31.90	C
ATOM	2149	CG1	VAL	A	288	-21.210	-29.341	22.318	1.00	34.01	C

```
ATOM   2150  CG2 VAL A 288     -19.009 -30.582  22.372  1.00 22.44           C
ATOM   2151  N   LEU A 289     -19.695 -33.333  20.046  1.00 28.61           N
ATOM   2152  CA  LEU A 289     -18.910 -34.092  19.090  1.00 31.47           C
ATOM   2153  C   LEU A 289     -19.657 -34.111  17.752  1.00 35.32           C
ATOM   2154  O   LEU A 289     -20.843 -34.431  17.707  1.00 34.79           O
ATOM   2155  CB  LEU A 289     -18.620 -35.501  19.609  1.00 22.19           C
ATOM   2156  CG  LEU A 289     -17.607 -35.556  20.769  1.00 26.54           C
ATOM   2157  CD1 LEU A 289     -17.383 -36.977  21.262  1.00 16.75           C
ATOM   2158  CD2 LEU A 289     -16.268 -34.906  20.391  1.00 25.36           C
ATOM   2159  N   PRO A 290     -18.966 -33.741  16.662  1.00 34.08           N
ATOM   2160  CA  PRO A 290     -19.576 -33.592  15.327  1.00 33.67           C
ATOM   2161  C   PRO A 290     -20.065 -34.902  14.712  1.00 34.58           C
ATOM   2162  O   PRO A 290     -21.027 -34.868  13.958  1.00 41.20           O
ATOM   2163  CB  PRO A 290     -18.445 -33.001  14.481  1.00 31.84           C
ATOM   2164  CG  PRO A 290     -17.184 -33.437  15.180  1.00 40.19           C
ATOM   2165  CD  PRO A 290     -17.518 -33.464  16.651  1.00 32.87           C
ATOM   2166  N   GLN A 291     -19.430 -36.031  15.017  1.00 37.51           N
ATOM   2167  CA  GLN A 291     -19.866 -37.309  14.442  1.00 34.40           C
ATOM   2168  C   GLN A 291     -19.796 -38.430  15.460  1.00 32.41           C
ATOM   2169  O   GLN A 291     -19.104 -38.323  16.473  1.00 36.76           O
ATOM   2170  CB  GLN A 291     -19.018 -37.693  13.215  1.00 41.43           C
ATOM   2171  CG  GLN A 291     -19.040 -36.692  12.049  1.00 43.40           C
ATOM   2172  CD  GLN A 291     -20.368 -36.667  11.292  1.00 53.77           C
ATOM   2173  OE1 GLN A 291     -21.100 -37.661  11.262  1.00 48.56           O
ATOM   2174  NE2 GLN A 291     -20.682 -35.523  10.679  1.00 42.95           N
ATOM   2175  N   GLU A 292     -20.518 -39.506  15.175  1.00 32.38           N
ATOM   2176  CA  GLU A 292     -20.495 -40.727  15.971  1.00 29.72           C
ATOM   2177  C   GLU A 292     -19.053 -41.230  16.041  1.00 35.49           C
ATOM   2178  O   GLU A 292     -18.367 -41.267  15.020  1.00 37.52           O
ATOM   2179  CB  GLU A 292     -21.397 -41.767  15.297  1.00 33.26           C
ATOM   2180  CG  GLU A 292     -21.564 -43.087  16.031  1.00 48.14           C
ATOM   2181  CD  GLU A 292     -22.407 -44.102  15.245  1.00 55.58           C
ATOM   2182  OE1 GLU A 292     -22.631 -43.894  14.029  1.00 50.51           O
ATOM   2183  OE2 GLU A 292     -22.841 -45.111  15.846  1.00 61.30           O
ATOM   2184  N   SER A 293     -18.589 -41.611  17.232  1.00 27.52           N
ATOM   2185  CA  SER A 293     -17.175 -41.946  17.415  1.00 25.58           C
ATOM   2186  C   SER A 293     -16.864 -42.749  18.683  1.00 27.39           C
ATOM   2187  O   SER A 293     -17.731 -42.980  19.522  1.00 32.07           O
ATOM   2188  CB  SER A 293     -16.330 -40.665  17.415  1.00 24.93           C
ATOM   2189  OG  SER A 293     -16.588 -39.881  18.573  1.00 27.41           O
ATOM   2190  N   VAL A 294     -15.624 -43.217  18.783  1.00 27.30           N
ATOM   2191  CA  VAL A 294     -15.042 -43.613  20.059  1.00 23.38           C
ATOM   2192  C   VAL A 294     -13.657 -42.982  20.117  1.00 24.64           C
ATOM   2193  O   VAL A 294     -13.134 -42.544  19.081  1.00 23.18           O
```

ATOM	2194	CB	VAL	A	294	-14.968	-45.150	20.261	1.00	24.79	C
ATOM	2195	CG1	VAL	A	294	-16.369	-45.755	20.300	1.00	26.24	C
ATOM	2196	CG2	VAL	A	294	-14.095	-45.829	19.198	1.00	23.02	C
ATOM	2197	N	PHE	A	295	-13.082	-42.905	21.317	1.00	24.25	N
ATOM	2198	CA	PHE	A	295	-11.747	-42.328	21.522	1.00	18.76	C
ATOM	2199	C	PHE	A	295	-11.675	-40.869	21.051	1.00	22.92	C
ATOM	2200	O	PHE	A	295	-10.644	-40.418	20.525	1.00	24.32	O
ATOM	2201	CB	PHE	A	295	-10.652	-43.189	20.849	1.00	17.57	C
ATOM	2202	CG	PHE	A	295	-9.384	-43.324	21.671	1.00	20.30	C
ATOM	2203	CD1	PHE	A	295	-8.837	-42.226	22.326	1.00	17.45	C
ATOM	2204	CD2	PHE	A	295	-8.749	-44.553	21.800	1.00	18.14	C
ATOM	2205	CE1	PHE	A	295	-7.681	-42.343	23.089	1.00	18.18	C
ATOM	2206	CE2	PHE	A	295	-7.585	-44.671	22.551	1.00	19.72	C
ATOM	2207	CZ	PHE	A	295	-7.059	-43.565	23.212	1.00	15.67	C
ATOM	2208	N	ARG	A	296	-12.780	-40.142	21.217	1.00	18.05	N
ATOM	2209	CA	ARG	A	296	-12.815	-38.712	20.905	1.00	21.16	C
ATOM	2210	C	ARG	A	296	-13.485	-37.991	22.052	1.00	23.12	C
ATOM	2211	O	ARG	A	296	-14.506	-38.440	22.563	1.00	20.28	O
ATOM	2212	CB	ARG	A	296	-13.585	-38.410	19.614	1.00	18.49	C
ATOM	2213	CG	ARG	A	296	-12.884	-38.822	18.327	1.00	23.57	C
ATOM	2214	CD	ARG	A	296	-11.617	-38.016	18.095	1.00	25.95	C
ATOM	2215	NE	ARG	A	296	-10.946	-38.439	16.875	1.00	28.30	N
ATOM	2216	CZ	ARG	A	296	-10.158	-39.506	16.775	1.00	27.16	C
ATOM	2217	NH1	ARG	A	296	-9.926	-40.272	17.837	1.00	21.89	N
ATOM	2218	NH2	ARG	A	296	-9.602	-39.807	15.606	1.00	22.49	N
ATOM	2219	N	PHE	A	297	-12.929	-36.854	22.443	1.00	21.88	N
ATOM	2220	CA	PHE	A	297	-13.447	-36.146	23.604	1.00	22.59	C
ATOM	2221	C	PHE	A	297	-13.655	-34.683	23.267	1.00	24.31	C
ATOM	2222	O	PHE	A	297	-12.880	-34.095	22.510	1.00	25.76	O
ATOM	2223	CB	PHE	A	297	-12.491	-36.340	24.775	1.00	18.27	C
ATOM	2224	CG	PHE	A	297	-11.853	-37.684	24.782	1.00	17.67	C
ATOM	2225	CD1	PHE	A	297	-12.599	-38.811	25.074	1.00	18.03	C
ATOM	2226	CD2	PHE	A	297	-10.518	-37.833	24.451	1.00	19.89	C
ATOM	2227	CE1	PHE	A	297	-12.019	-40.074	25.055	1.00	20.47	C
ATOM	2228	CE2	PHE	A	297	-9.926	-39.093	24.438	1.00	19.79	C
ATOM	2229	CZ	PHE	A	297	-10.678	-40.212	24.739	1.00	19.51	C
ATOM	2230	N	SER	A	298	-14.709	-34.105	23.829	1.00	24.71	N
ATOM	2231	CA	SER	A	298	-15.180	-32.792	23.404	1.00	27.45	C
ATOM	2232	C	SER	A	298	-14.209	-31.652	23.674	1.00	26.14	C
ATOM	2233	O	SER	A	298	-13.908	-30.873	22.777	1.00	28.28	O
ATOM	2234	CB	SER	A	298	-16.536	-32.487	24.040	1.00	23.34	C
ATOM	2235	OG	SER	A	298	-17.525	-33.332	23.482	1.00	30.43	O
ATOM	2236	N	PHE	A	299	-13.732	-31.547	24.911	1.00	23.00	N
ATOM	2237	CA	PHE	A	299	-12.884	-30.426	25.292	1.00	23.92	C

ATOM	2238	C	PHE	A	299	-11.469	-30.868	25.630	1.00	27.99	C
ATOM	2239	O	PHE	A	299	-11.217	-31.479	26.679	1.00	21.70	O
ATOM	2240	CB	PHE	A	299	-13.542	-29.610	26.410	1.00	23.64	C
ATOM	2241	CG	PHE	A	299	-14.891	-29.098	26.026	1.00	25.23	C
ATOM	2242	CD1	PHE	A	299	-15.009	-28.039	25.132	1.00	27.98	C
ATOM	2243	CD2	PHE	A	299	-16.045	-29.718	26.490	1.00	22.29	C
ATOM	2244	CE1	PHE	A	299	-16.264	-27.578	24.722	1.00	23.47	C
ATOM	2245	CE2	PHE	A	299	-17.301	-29.269	26.091	1.00	25.92	C
ATOM	2246	CZ	PHE	A	299	-17.410	-28.194	25.205	1.00	24.79	C
ATOM	2247	N	VAL	A	300	-10.557	-30.572	24.704	1.00	22.10	N
ATOM	2248	CA	VAL	A	300	-9.169	-31.009	24.807	1.00	22.42	C
ATOM	2249	C	VAL	A	300	-8.245	-29.814	24.563	1.00	20.22	C
ATOM	2250	O	VAL	A	300	-8.722	-28.744	24.198	1.00	21.24	O
ATOM	2251	CB	VAL	A	300	-8.880	-32.171	23.819	1.00	23.10	C
ATOM	2252	CG1	VAL	A	300	-9.770	-33.376	24.139	1.00	18.44	C
ATOM	2253	CG2	VAL	A	300	-9.051	-31.711	22.373	1.00	20.54	C
ATOM	2254	N	PRO	A	301	-6.930	-29.975	24.799	1.00	24.37	N
ATOM	2255	CA	PRO	A	301	-6.001	-28.882	24.476	1.00	25.07	C
ATOM	2256	C	PRO	A	301	-6.172	-28.327	23.056	1.00	30.22	C
ATOM	2257	O	PRO	A	301	-6.373	-29.073	22.094	1.00	25.83	O
ATOM	2258	CB	PRO	A	301	-4.633	-29.550	24.613	1.00	23.26	C
ATOM	2259	CG	PRO	A	301	-4.846	-30.567	25.681	1.00	26.00	C
ATOM	2260	CD	PRO	A	301	-6.234	-31.101	25.456	1.00	19.57	C
ATOM	2261	N	VAL	A	302	-6.110	-27.007	22.939	1.00	28.76	N
ATOM	2262	CA	VAL	A	302	-6.291	-26.349	21.661	1.00	28.19	C
ATOM	2263	C	VAL	A	302	-4.948	-25.784	21.180	1.00	34.31	C
ATOM	2264	O	VAL	A	302	-4.133	-25.318	21.977	1.00	32.40	O
ATOM	2265	CB	VAL	A	302	-7.398	-25.249	21.744	1.00	33.01	C
ATOM	2266	CG1	VAL	A	302	-6.977	-24.107	22.667	1.00	33.69	C
ATOM	2267	CG2	VAL	A	302	-7.748	-24.719	20.361	1.00	35.82	C
ATOM	2268	N	VAL	A	303	-4.693	-25.881	19.880	1.00	38.19	N
ATOM	2269	CA	VAL	A	303	-3.486	-25.296	19.306	1.00	37.52	C
ATOM	2270	C	VAL	A	303	-3.826	-23.879	18.893	1.00	38.56	C
ATOM	2271	O	VAL	A	303	-4.544	-23.668	17.914	1.00	42.58	O
ATOM	2272	CB	VAL	A	303	-3.012	-26.067	18.062	1.00	39.71	C
ATOM	2273	CG1	VAL	A	303	-1.689	-25.508	17.568	1.00	36.03	C
ATOM	2274	CG2	VAL	A	303	-2.895	-27.554	18.365	1.00	37.39	C
ATOM	2275	N	ASP	A	304	-3.334	-22.903	19.640	1.00	38.42	N
ATOM	2276	CA	ASP	A	304	-3.734	-21.524	19.396	1.00	42.73	C
ATOM	2277	C	ASP	A	304	-2.540	-20.575	19.307	1.00	44.58	C
ATOM	2278	O	ASP	A	304	-2.713	-19.360	19.237	1.00	50.09	O
ATOM	2279	CB	ASP	A	304	-4.710	-21.057	20.481	1.00	41.11	C
ATOM	2280	CG	ASP	A	304	-4.028	-20.813	21.819	1.00	40.98	C
ATOM	2281	OD1	ASP	A	304	-2.955	-21.396	22.071	1.00	40.45	O

ATOM	2282	OD2	ASP	A	304	-4.573	-20.033	22.626	1.00	50.49	O
ATOM	2283	N	GLY	A	305	-1.333	-21.132	19.326	1.00	39.12	N
ATOM	2284	CA	GLY	A	305	-0.131	-20.328	19.225	1.00	37.31	C
ATOM	2285	C	GLY	A	305	0.471	-19.929	20.559	1.00	44.74	C
ATOM	2286	O	GLY	A	305	1.592	-19.420	20.602	1.00	46.74	O
ATOM	2287	N	ASP	A	306	-0.259	-20.173	21.647	1.00	41.02	N
ATOM	2288	CA	ASP	A	306	0.175	-19.763	22.983	1.00	38.02	C
ATOM	2289	C	ASP	A	306	0.905	-20.882	23.754	1.00	43.86	C
ATOM	2290	O	ASP	A	306	2.128	-20.998	23.641	1.00	40.78	O
ATOM	2291	CB	ASP	A	306	-1.010	-19.205	23.781	1.00	37.18	C
ATOM	2292	CG	ASP	A	306	-0.587	-18.573	25.098	1.00	47.53	C
ATOM	2293	OD1	ASP	A	306	0.637	-18.480	25.358	1.00	39.59	O
ATOM	2294	OD2	ASP	A	306	-1.484	-18.151	25.866	1.00	52.97	O
ATOM	2295	N	PHE	A	307	0.175	-21.678	24.546	1.00	38.70	N
ATOM	2296	CA	PHE	A	307	0.764	-22.843	25.224	1.00	31.70	C
ATOM	2297	C	PHE	A	307	1.490	-23.724	24.205	1.00	37.55	C
ATOM	2298	O	PHE	A	307	2.623	-24.152	24.424	1.00	40.20	O
ATOM	2299	CB	PHE	A	307	-0.313	-23.669	25.934	1.00	35.00	C
ATOM	2300	CG	PHE	A	307	0.235	-24.683	26.915	1.00	30.59	C
ATOM	2301	CD1	PHE	A	307	0.795	-25.877	26.472	1.00	27.47	C
ATOM	2302	CD2	PHE	A	307	0.175	-24.444	28.281	1.00	30.22	C
ATOM	2303	CE1	PHE	A	307	1.299	-26.807	27.375	1.00	29.22	C
ATOM	2304	CE2	PHE	A	307	0.671	-25.372	29.199	1.00	26.08	C
ATOM	2305	CZ	PHE	A	307	1.232	-26.553	28.747	1.00	26.19	C
ATOM	2306	N	LEU	A	308	0.818	-23.992	23.091	1.00	33.65	N
ATOM	2307	CA	LEU	A	308	1.422	-24.691	21.972	1.00	39.58	C
ATOM	2308	C	LEU	A	308	1.545	-23.719	20.798	1.00	40.30	C
ATOM	2309	O	LEU	A	308	0.539	-23.333	20.202	1.00	44.59	O
ATOM	2310	CB	LEU	A	308	0.552	-25.881	21.575	1.00	35.62	C
ATOM	2311	CG	LEU	A	308	0.276	-26.911	22.669	1.00	41.43	C
ATOM	2312	CD1	LEU	A	308	-0.815	-27.867	22.213	1.00	33.84	C
ATOM	2313	CD2	LEU	A	308	1.556	-27.670	23.051	1.00	30.55	C
ATOM	2314	N	SER	A	309	2.769	-23.316	20.471	1.00	34.30	N
ATOM	2315	CA	SER	A	309	2.984	-22.352	19.393	1.00	44.40	C
ATOM	2316	C	SER	A	309	2.684	-22.954	18.016	1.00	43.27	C
ATOM	2317	O	SER	A	309	2.444	-22.233	17.058	1.00	50.15	O
ATOM	2318	CB	SER	A	309	4.402	-21.784	19.442	1.00	38.91	C
ATOM	2319	OG	SER	A	309	5.361	-22.812	19.320	1.00	45.78	O
ATOM	2320	N	ASP	A	310	2.684	-24.277	17.930	1.00	45.10	N
ATOM	2321	CA	ASP	A	310	2.293	-24.967	16.708	1.00	44.33	C
ATOM	2322	C	ASP	A	310	1.636	-26.284	17.110	1.00	40.79	C
ATOM	2323	O	ASP	A	310	1.514	-26.575	18.291	1.00	40.13	O
ATOM	2324	CB	ASP	A	310	3.524	-25.236	15.841	1.00	47.92	C
ATOM	2325	CG	ASP	A	310	3.201	-25.304	14.352	1.00	52.63	C

ATOM	2326	OD1	ASP	A	310	2.064	-25.683	13.986	1.00	45.33	O
ATOM	2327	OD2	ASP	A	310	4.100	-24.977	13.546	1.00	53.26	O
ATOM	2328	N	THR	A	311	1.204	-27.073	16.134	1.00	40.30	N
ATOM	2329	CA	THR	A	311	0.721	-28.417	16.418	1.00	39.03	C
ATOM	2330	C	THR	A	311	1.837	-29.230	17.080	1.00	39.52	C
ATOM	2331	O	THR	A	311	3.022	-28.968	16.855	1.00	41.00	O
ATOM	2332	CB	THR	A	311	0.263	-29.139	15.134	1.00	44.58	C
ATOM	2333	OG1	THR	A	311	1.405	-29.486	14.341	1.00	44.53	O
ATOM	2334	CG2	THR	A	311	-0.671	-28.251	14.326	1.00	34.09	C
ATOM	2335	N	PRO	A	312	1.469	-30.206	17.921	1.00	38.37	N
ATOM	2336	CA	PRO	A	312	2.517	-31.029	18.533	1.00	36.87	C
ATOM	2337	C	PRO	A	312	3.323	-31.781	17.473	1.00	37.32	C
ATOM	2338	O	PRO	A	312	4.529	-31.954	17.629	1.00	37.83	O
ATOM	2339	CB	PRO	A	312	1.724	-31.998	19.417	1.00	32.85	C
ATOM	2340	CG	PRO	A	312	0.467	-31.243	19.758	1.00	32.08	C
ATOM	2341	CD	PRO	A	312	0.140	-30.472	18.503	1.00	36.52	C
ATOM	2342	N	GLU	A	313	2.655	-32.216	16.408	0.45	40.47	N
ATOM	2344	CA	GLU	A	313	3.334	-32.884	15.301	0.45	41.44	C
ATOM	2346	C	GLU	A	313	4.456	-32.011	14.730	0.45	43.11	C
ATOM	2348	O	GLU	A	313	5.579	-32.479	14.542	0.45	44.39	O
ATOM	2350	CB	GLU	A	313	2.339	-33.281	14.202	0.45	40.97	C
ATOM	2352	CG	GLU	A	313	1.476	-34.508	14.527	0.45	40.40	C
ATOM	2354	CD	GLU	A	313	0.140	-34.159	15.185	0.45	41.13	C
ATOM	2356	OE1	GLU	A	313	-0.814	-34.955	15.036	0.45	41.54	O
ATOM	2358	OE2	GLU	A	313	0.040	-33.102	15.849	0.45	34.24	O
ATOM	2360	N	ALA	A	314	4.152	-30.742	14.474	1.00	39.34	N
ATOM	2361	CA	ALA	A	314	5.153	-29.801	13.988	1.00	40.75	C
ATOM	2362	C	ALA	A	314	6.260	-29.591	15.016	1.00	43.85	C
ATOM	2363	O	ALA	A	314	7.445	-29.706	14.692	1.00	43.87	O
ATOM	2364	CB	ALA	A	314	4.502	-28.463	13.629	1.00	41.36	C
ATOM	2365	N	LEU	A	315	5.864	-29.286	16.255	1.00	45.66	N
ATOM	2366	CA	LEU	A	315	6.813	-29.002	17.330	1.00	37.09	C
ATOM	2367	C	LEU	A	315	7.752	-30.178	17.567	1.00	38.04	C
ATOM	2368	O	LEU	A	315	8.937	-29.996	17.862	1.00	38.97	O
ATOM	2369	CB	LEU	A	315	6.077	-28.655	18.624	1.00	40.59	C
ATOM	2370	CG	LEU	A	315	5.332	-27.326	18.646	1.00	43.56	C
ATOM	2371	CD1	LEU	A	315	4.705	-27.097	20.012	1.00	36.99	C
ATOM	2372	CD2	LEU	A	315	6.274	-26.188	18.281	1.00	44.42	C
ATOM	2373	N	ILE	A	316	7.207	-31.381	17.439	1.00	36.19	N
ATOM	2374	CA	ILE	A	316	7.998	-32.597	17.537	1.00	39.95	C
ATOM	2375	C	ILE	A	316	9.003	-32.670	16.393	1.00	40.32	C
ATOM	2376	O	ILE	A	316	10.207	-32.723	16.629	1.00	44.96	O
ATOM	2377	CB	ILE	A	316	7.098	-33.857	17.579	1.00	37.42	C
ATOM	2378	CG1	ILE	A	316	6.490	-34.014	18.969	1.00	38.94	C

ATOM	2379	CG2	ILE	A	316	7.889	-35.106	17.281	1.00	34.48	C
ATOM	2380	CD1	ILE	A	316	5.203	-34.798	18.976	1.00	33.67	C
ATOM	2381	N	ASN	A	317	8.507	-32.641	15.157	1.00	51.63	N
ATOM	2382	CA	ASN	A	317	9.365	-32.749	13.973	1.00	48.94	C
ATOM	2383	C	ASN	A	317	10.510	-31.735	13.913	1.00	47.69	C
ATOM	2384	O	ASN	A	317	11.550	-32.015	13.323	1.00	56.86	O
ATOM	2385	CB	ASN	A	317	8.536	-32.675	12.690	1.00	46.15	C
ATOM	2386	CG	ASN	A	317	7.544	-33.812	12.572	1.00	52.93	C
ATOM	2387	OD1	ASN	A	317	7.688	-34.857	13.215	1.00	56.06	O
ATOM	2388	ND2	ASN	A	317	6.522	-33.615	11.746	1.00	55.78	N
ATOM	2389	N	ALA	A	318	10.328	-30.580	14.551	1.00	50.93	N
ATOM	2390	CA	ALA	A	318	11.292	-29.484	14.467	1.00	46.95	C
ATOM	2391	C	ALA	A	318	12.130	-29.262	15.730	1.00	51.65	C
ATOM	2392	O	ALA	A	318	12.915	-28.309	15.799	1.00	52.04	O
ATOM	2393	CB	ALA	A	318	10.569	-28.188	14.093	1.00	43.38	C
ATOM	2394	N	GLY	A	319	11.976	-30.128	16.726	1.00	49.62	N
ATOM	2395	CA	GLY	A	319	12.622	-29.905	18.012	1.00	37.36	C
ATOM	2396	C	GLY	A	319	14.026	-30.474	18.144	1.00	41.98	C
ATOM	2397	O	GLY	A	319	14.385	-31.442	17.470	1.00	45.58	O
ATOM	2398	N	ASP	A	320	14.821	-29.854	19.016	1.00	37.58	N
ATOM	2399	CA	ASP	A	320	16.143	-30.348	19.379	1.00	43.63	C
ATOM	2400	C	ASP	A	320	15.984	-30.960	20.767	1.00	44.73	C
ATOM	2401	O	ASP	A	320	15.701	-30.250	21.733	1.00	42.42	O
ATOM	2402	CB	ASP	A	320	17.158	-29.187	19.385	1.00	40.28	C
ATOM	2403	CG	ASP	A	320	18.551	-29.591	19.894	1.00	51.11	C
ATOM	2404	OD1	ASP	A	320	18.715	-30.688	20.473	1.00	52.23	O
ATOM	2405	OD2	ASP	A	320	19.500	-28.782	19.732	1.00	57.71	O
ATOM	2406	N	PHE	A	321	16.168	-32.275	20.866	1.00	40.53	N
ATOM	2407	CA	PHE	A	321	15.905	-32.976	22.121	1.00	34.15	C
ATOM	2408	C	PHE	A	321	17.144	-33.622	22.734	1.00	41.70	C
ATOM	2409	O	PHE	A	321	17.039	-34.611	23.472	1.00	38.14	O
ATOM	2410	CB	PHE	A	321	14.792	-34.012	21.937	1.00	31.78	C
ATOM	2411	CG	PHE	A	321	13.473	-33.412	21.531	1.00	37.41	C
ATOM	2412	CD1	PHE	A	321	12.650	-32.809	22.473	1.00	30.90	C
ATOM	2413	CD2	PHE	A	321	13.065	-33.435	20.206	1.00	34.59	C
ATOM	2414	CE1	PHE	A	321	11.441	-32.253	22.106	1.00	30.60	C
ATOM	2415	CE2	PHE	A	321	11.854	-32.871	19.826	1.00	37.44	C
ATOM	2416	CZ	PHE	A	321	11.041	-32.282	20.784	1.00	38.03	C
ATOM	2417	N	HIS	A	322	18.317	-33.073	22.426	1.00	39.99	N
ATOM	2418	CA	HIS	A	322	19.525	-33.477	23.137	1.00	45.55	C
ATOM	2419	C	HIS	A	322	19.363	-33.092	24.600	1.00	42.42	C
ATOM	2420	O	HIS	A	322	18.813	-32.030	24.912	1.00	41.22	O
ATOM	2421	CB	HIS	A	322	20.780	-32.827	22.535	1.00	43.13	C
ATOM	2422	CG	HIS	A	322	21.186	-33.419	21.222	1.00	40.16	C

```
ATOM   2423  ND1 HIS A 322      20.806 -32.879  20.012  1.00 37.83           N
ATOM   2424  CD2 HIS A 322      21.907 -34.529  20.930  1.00 35.53           C
ATOM   2425  CE1 HIS A 322      21.285 -33.623  19.030  1.00 40.44           C
ATOM   2426  NE2 HIS A 322      21.958 -34.629  19.562  1.00 38.10           N
ATOM   2427  N   GLY A 323      19.816 -33.968  25.492  1.00 38.52           N
ATOM   2428  CA  GLY A 323      19.698 -33.726  26.919  1.00 41.24           C
ATOM   2429  C   GLY A 323      18.464 -34.369  27.530  1.00 40.91           C
ATOM   2430  O   GLY A 323      18.336 -34.450  28.749  1.00 45.29           O
ATOM   2431  N   LEU A 324      17.557 -34.834  26.675  1.00 37.18           N
ATOM   2432  CA  LEU A 324      16.309 -35.438  27.124  1.00 34.07           C
ATOM   2433  C   LEU A 324      16.358 -36.977  27.181  1.00 26.56           C
ATOM   2434  O   LEU A 324      16.871 -37.641  26.274  1.00 30.78           O
ATOM   2435  CB  LEU A 324      15.152 -34.962  26.229  1.00 34.43           C
ATOM   2436  CG  LEU A 324      13.732 -35.476  26.505  1.00 31.32           C
ATOM   2437  CD1 LEU A 324      13.255 -35.053  27.890  1.00 27.23           C
ATOM   2438  CD2 LEU A 324      12.767 -34.983  25.441  1.00 26.28           C
ATOM   2439  N   GLN A 325      15.833 -37.537  28.263  1.00 26.91           N
ATOM   2440  CA  GLN A 325      15.576 -38.973  28.318  1.00 32.12           C
ATOM   2441  C   GLN A 325      14.080 -39.192  28.516  1.00 29.99           C
ATOM   2442  O   GLN A 325      13.443 -38.505  29.311  1.00 29.51           O
ATOM   2443  CB  GLN A 325      16.364 -39.658  29.440  1.00 33.24           C
ATOM   2444  CG  GLN A 325      17.874 -39.504  29.349  1.00 35.15           C
ATOM   2445  CD  GLN A 325      18.376 -38.316  30.136  1.00 42.38           C
ATOM   2446  OE1 GLN A 325      17.997 -38.113  31.296  1.00 44.08           O
ATOM   2447  NE2 GLN A 325      19.221 -37.509  29.506  1.00 48.06           N
ATOM   2448  N   VAL A 326      13.516 -40.136  27.774  1.00 29.09           N
ATOM   2449  CA  VAL A 326      12.082 -40.376  27.818  1.00 27.12           C
ATOM   2450  C   VAL A 326      11.825 -41.872  27.895  1.00 29.09           C
ATOM   2451  O   VAL A 326      12.536 -42.664  27.267  1.00 30.47           O
ATOM   2452  CB  VAL A 326      11.367 -39.840  26.545  1.00 30.99           C
ATOM   2453  CG1 VAL A 326       9.958 -39.413  26.878  1.00 31.05           C
ATOM   2454  CG2 VAL A 326      12.115 -38.675  25.947  1.00 36.39           C
ATOM   2455  N   LEU A 327      10.805 -42.254  28.659  1.00 26.83           N
ATOM   2456  CA  LEU A 327      10.331 -43.639  28.694  1.00 25.10           C
ATOM   2457  C   LEU A 327       8.918 -43.669  28.123  1.00 24.82           C
ATOM   2458  O   LEU A 327       8.028 -42.968  28.613  1.00 26.01           O
ATOM   2459  CB  LEU A 327      10.338 -44.170  30.127  1.00 23.82           C
ATOM   2460  CG  LEU A 327       9.793 -45.573  30.423  1.00 26.62           C
ATOM   2461  CD1 LEU A 327      10.617 -46.626  29.746  1.00 27.61           C
ATOM   2462  CD2 LEU A 327       9.789 -45.827  31.926  1.00 30.20           C
ATOM   2463  N   VAL A 328       8.711 -44.453  27.070  1.00 23.86           N
ATOM   2464  CA  VAL A 328       7.391 -44.523  26.441  1.00 24.94           C
ATOM   2465  C   VAL A 328       6.961 -45.969  26.226  1.00 24.06           C
ATOM   2466  O   VAL A 328       7.790 -46.871  26.137  1.00 21.42           O
```

ATOM	2467	CB	VAL	A	328	7.339	-43.779	25.081	1.00	23.74	C
ATOM	2468	CG1	VAL	A	328	7.850	-42.351	25.224	1.00	22.32	C
ATOM	2469	CG2	VAL	A	328	8.127	-44.532	24.028	1.00	21.83	C
ATOM	2470	N	GLY	A	329	5.659	-46.190	26.137	1.00	18.02	N
ATOM	2471	CA	GLY	A	329	5.168	-47.531	25.917	1.00	18.90	C
ATOM	2472	C	GLY	A	329	3.666	-47.604	25.796	1.00	22.66	C
ATOM	2473	O	GLY	A	329	2.954	-46.598	25.932	1.00	19.46	O
ATOM	2474	N	VAL	A	330	3.188	-48.815	25.540	1.00	17.93	N
ATOM	2475	CA	VAL	A	330	1.794	-49.051	25.241	1.00	18.46	C
ATOM	2476	C	VAL	A	330	1.434	-50.371	25.884	1.00	24.94	C
ATOM	2477	O	VAL	A	330	2.332	-51.153	26.220	1.00	21.09	O
ATOM	2478	CB	VAL	A	330	1.590	-49.197	23.714	1.00	21.64	C
ATOM	2479	CG1	VAL	A	330	1.900	-47.890	22.993	1.00	20.24	C
ATOM	2480	CG2	VAL	A	330	2.471	-50.312	23.162	1.00	19.18	C
ATOM	2481	N	VAL	A	331	0.135	-50.628	26.056	1.00	21.32	N
ATOM	2482	CA	VAL	A	331	-0.324	-51.980	26.393	1.00	23.09	C
ATOM	2483	C	VAL	A	331	-0.492	-52.833	25.129	1.00	26.05	C
ATOM	2484	O	VAL	A	331	-0.491	-52.312	24.011	1.00	23.57	O
ATOM	2485	CB	VAL	A	331	-1.639	-51.976	27.197	1.00	25.63	C
ATOM	2486	CG1	VAL	A	331	-1.446	-51.243	28.530	1.00	18.82	C
ATOM	2487	CG2	VAL	A	331	-2.775	-51.358	26.382	1.00	21.06	C
ATOM	2488	N	LYS	A	332	-0.631	-54.142	25.317	1.00	26.90	N
ATOM	2489	CA	LYS	A	332	-0.742	-55.082	24.208	1.00	29.99	C
ATOM	2490	C	LYS	A	332	-1.955	-54.817	23.316	1.00	29.15	C
ATOM	2491	O	LYS	A	332	-1.874	-54.967	22.099	1.00	27.12	O
ATOM	2492	CB	LYS	A	332	-0.779	-56.524	24.724	1.00	31.15	C
ATOM	2493	CG	LYS	A	332	-0.633	-57.589	23.628	1.00	27.70	C
ATOM	2494	CD	LYS	A	332	-0.439	-58.970	24.254	1.00	34.51	C
ATOM	2495	CE	LYS	A	332	-0.093	-60.025	23.220	1.00	35.15	C
ATOM	2496	NZ	LYS	A	332	-1.231	-60.356	22.302	1.00	46.13	N
ATOM	2497	N	ASP	A	333	-3.070	-54.413	23.918	1.00	23.08	N
ATOM	2498	CA	ASP	A	333	-4.297	-54.190	23.157	1.00	26.75	C
ATOM	2499	C	ASP	A	333	-4.918	-52.822	23.424	1.00	26.56	C
ATOM	2500	O	ASP	A	333	-5.945	-52.723	24.091	1.00	27.52	O
ATOM	2501	CB	ASP	A	333	-5.309	-55.308	23.453	1.00	25.08	C
ATOM	2502	CG	ASP	A	333	-4.737	-56.698	23.172	1.00	29.59	C
ATOM	2503	OD1	ASP	A	333	-4.718	-57.108	21.986	1.00	32.00	O
ATOM	2504	OD2	ASP	A	333	-4.283	-57.367	24.133	1.00	26.66	O
ATOM	2505	N	GLU	A	334	-4.300	-51.775	22.881	1.00	26.23	N
ATOM	2506	CA	GLU	A	334	-4.741	-50.397	23.106	1.00	24.52	C
ATOM	2507	C	GLU	A	334	-6.187	-50.119	22.706	1.00	26.78	C
ATOM	2508	O	GLU	A	334	-6.872	-49.321	23.358	1.00	25.34	O
ATOM	2509	CB	GLU	A	334	-3.815	-49.398	22.387	1.00	21.93	C
ATOM	2510	CG	GLU	A	334	-2.406	-49.284	22.993	1.00	19.96	C

```
ATOM   2511  CD  GLU A 334      -2.412 -48.616  24.371  1.00 26.36           C
ATOM   2512  OE1 GLU A 334      -3.469 -48.069  24.763  1.00 24.03           O
ATOM   2513  OE2 GLU A 334      -1.366 -48.632  25.063  1.00 23.90           O
ATOM   2514  N   GLY A 335      -6.660 -50.764  21.644  1.00 23.90           N
ATOM   2515  CA  GLY A 335      -7.946 -50.388  21.077  1.00 22.42           C
ATOM   2516  C   GLY A 335      -9.174 -51.090  21.634  1.00 23.54           C
ATOM   2517  O   GLY A 335     -10.283 -50.564  21.555  1.00 20.98           O
ATOM   2518  N   SER A 336      -8.978 -52.274  22.202  1.00 23.11           N
ATOM   2519  CA  SER A 336     -10.095 -53.149  22.543  1.00 26.70           C
ATOM   2520  C   SER A 336     -11.143 -52.500  23.458  1.00 27.34           C
ATOM   2521  O   SER A 336     -12.338 -52.562  23.183  1.00 29.04           O
ATOM   2522  CB  SER A 336      -9.577 -54.460  23.137  1.00 27.44           C
ATOM   2523  OG  SER A 336      -9.049 -54.263  24.429  1.00 28.54           O
ATOM   2524  N   TYR A 337     -10.674 -51.866  24.526  1.00 27.57           N
ATOM   2525  CA  TYR A 337     -11.517 -51.200  25.516  1.00 24.96           C
ATOM   2526  C   TYR A 337     -12.529 -50.254  24.877  1.00 25.09           C
ATOM   2527  O   TYR A 337     -13.702 -50.228  25.244  1.00 28.42           O
ATOM   2528  CB  TYR A 337     -10.600 -50.402  26.438  1.00 29.14           C
ATOM   2529  CG  TYR A 337     -11.082 -50.168  27.846  1.00 28.05           C
ATOM   2530  CD1 TYR A 337     -12.430 -49.969  28.136  1.00 28.74           C
ATOM   2531  CD2 TYR A 337     -10.172 -50.123  28.895  1.00 27.72           C
ATOM   2532  CE1 TYR A 337     -12.852 -49.747  29.439  1.00 26.86           C
ATOM   2533  CE2 TYR A 337     -10.584 -49.896  30.196  1.00 27.01           C
ATOM   2534  CZ  TYR A 337     -11.918 -49.705  30.464  1.00 25.53           C
ATOM   2535  OH  TYR A 337     -12.304 -49.478  31.766  1.00 26.03           O
ATOM   2536  N   PHE A 338     -12.075 -49.474  23.912  1.00 22.82           N
ATOM   2537  CA  PHE A 338     -12.919 -48.443  23.325  1.00 21.85           C
ATOM   2538  C   PHE A 338     -14.076 -48.966  22.462  1.00 26.20           C
ATOM   2539  O   PHE A 338     -15.075 -48.265  22.273  1.00 20.06           O
ATOM   2540  CB  PHE A 338     -12.050 -47.443  22.553  1.00 24.01           C
ATOM   2541  CG  PHE A 338     -11.095 -46.704  23.432  1.00 20.39           C
ATOM   2542  CD1 PHE A 338      -9.901 -47.288  23.815  1.00 21.01           C
ATOM   2543  CD2 PHE A 338     -11.414 -45.447  23.922  1.00 19.20           C
ATOM   2544  CE1 PHE A 338      -9.031 -46.624  24.675  1.00 22.38           C
ATOM   2545  CE2 PHE A 338     -10.542 -44.773  24.761  1.00 19.97           C
ATOM   2546  CZ  PHE A 338      -9.352 -45.367  25.140  1.00 17.92           C
ATOM   2547  N   LEU A 339     -13.961 -50.200  21.973  1.00 26.33           N
ATOM   2548  CA  LEU A 339     -14.969 -50.754  21.065  1.00 29.21           C
ATOM   2549  C   LEU A 339     -16.361 -50.880  21.693  1.00 32.14           C
ATOM   2550  O   LEU A 339     -17.372 -50.764  20.987  1.00 30.47           O
ATOM   2551  CB  LEU A 339     -14.507 -52.102  20.497  1.00 24.76           C
ATOM   2552  CG  LEU A 339     -13.170 -51.983  19.763  1.00 24.66           C
ATOM   2553  CD1 LEU A 339     -12.638 -53.315  19.253  1.00 25.37           C
ATOM   2554  CD2 LEU A 339     -13.305 -50.990  18.645  1.00 21.23           C
```

ATOM	2555	N	VAL	A	340	-16.421	-51.096	23.009	1.00	25.61	N
ATOM	2556	CA	VAL	A	340	-17.715	-51.247	23.671	1.00	24.07	C
ATOM	2557	C	VAL	A	340	-18.429	-49.922	23.928	1.00	30.99	C
ATOM	2558	O	VAL	A	340	-19.548	-49.916	24.438	1.00	33.06	O
ATOM	2559	CB	VAL	A	340	-17.623	-52.051	24.973	1.00	30.08	C
ATOM	2560	CG1	VAL	A	340	-17.162	-53.483	24.680	1.00	28.51	C
ATOM	2561	CG2	VAL	A	340	-16.701	-51.359	25.972	1.00	27.77	C
ATOM	2562	N	TYR	A	341	-17.801	-48.806	23.556	1.00	24.57	N
ATOM	2563	CA	TYR	A	341	-18.427	-47.492	23.731	1.00	20.56	C
ATOM	2564	C	TYR	A	341	-18.986	-46.895	22.441	1.00	24.89	C
ATOM	2565	O	TYR	A	341	-19.209	-45.680	22.384	1.00	23.54	O
ATOM	2566	CB	TYR	A	341	-17.440	-46.484	24.352	1.00	23.39	C
ATOM	2567	CG	TYR	A	341	-17.145	-46.710	25.820	1.00	23.42	C
ATOM	2568	CD1	TYR	A	341	-17.801	-45.975	26.804	1.00	23.97	C
ATOM	2569	CD2	TYR	A	341	-16.228	-47.668	26.220	1.00	21.18	C
ATOM	2570	CE1	TYR	A	341	-17.545	-46.184	28.145	1.00	22.86	C
ATOM	2571	CE2	TYR	A	341	-15.967	-47.889	27.555	1.00	25.11	C
ATOM	2572	CZ	TYR	A	341	-16.625	-47.140	28.514	1.00	23.75	C
ATOM	2573	OH	TYR	A	341	-16.354	-47.356	29.841	1.00	24.33	O
ATOM	2574	N	GLY	A	342	-19.203	-47.718	21.411	1.00	25.46	N
ATOM	2575	CA	GLY	A	342	-19.739	-47.204	20.158	1.00	25.99	C
ATOM	2576	C	GLY	A	342	-19.644	-48.051	18.893	1.00	28.24	C
ATOM	2577	O	GLY	A	342	-20.348	-47.795	17.918	1.00	29.98	O
ATOM	2578	N	ALA	A	343	-18.775	-49.050	18.883	1.00	31.23	N
ATOM	2579	CA	ALA	A	343	-18.662	-49.902	17.711	1.00	31.05	C
ATOM	2580	C	ALA	A	343	-19.840	-50.870	17.668	1.00	31.62	C
ATOM	2581	O	ALA	A	343	-20.101	-51.567	18.645	1.00	35.06	O
ATOM	2582	CB	ALA	A	343	-17.348	-50.659	17.725	1.00	28.45	C
ATOM	2583	N	PRO	A	344	-20.561	-50.907	16.534	1.00	29.97	N
ATOM	2584	CA	PRO	A	344	-21.691	-51.831	16.365	1.00	30.94	C
ATOM	2585	C	PRO	A	344	-21.275	-53.283	16.550	1.00	33.09	C
ATOM	2586	O	PRO	A	344	-20.244	-53.714	16.023	1.00	29.48	O
ATOM	2587	CB	PRO	A	344	-22.139	-51.590	14.915	1.00	30.57	C
ATOM	2588	CG	PRO	A	344	-20.971	-50.917	14.243	1.00	35.11	C
ATOM	2589	CD	PRO	A	344	-20.330	-50.091	15.328	1.00	30.43	C
ATOM	2590	N	GLY	A	345	-22.064	-54.020	17.323	1.00	32.04	N
ATOM	2591	CA	GLY	A	345	-21.825	-55.433	17.520	1.00	33.17	C
ATOM	2592	C	GLY	A	345	-20.965	-55.780	18.721	1.00	34.90	C
ATOM	2593	O	GLY	A	345	-20.758	-56.959	19.007	1.00	34.40	O
ATOM	2594	N	PHE	A	346	-20.468	-54.777	19.439	1.00	32.75	N
ATOM	2595	CA	PHE	A	346	-19.559	-55.072	20.554	1.00	31.69	C
ATOM	2596	C	PHE	A	346	-20.222	-55.103	21.925	1.00	30.22	C
ATOM	2597	O	PHE	A	346	-21.126	-54.323	22.214	1.00	37.20	O
ATOM	2598	CB	PHE	A	346	-18.372	-54.108	20.561	1.00	25.90	C

ATOM	2599	CG	PHE	A	346	-17.315	-54.457	19.557	1.00 30.40	C
ATOM	2600	CD1	PHE	A	346	-16.212	-55.204	19.926	1.00 26.14	C
ATOM	2601	CD2	PHE	A	346	-17.430	-54.043	18.238	1.00 27.66	C
ATOM	2602	CE1	PHE	A	346	-15.246	-55.530	19.002	1.00 26.44	C
ATOM	2603	CE2	PHE	A	346	-16.466	-54.363	17.315	1.00 29.07	C
ATOM	2604	CZ	PHE	A	346	-15.377	-55.108	17.694	1.00 25.30	C
ATOM	2605	N	SER	A	347	-19.751	-56.007	22.774	1.00 36.78	N
ATOM	2606	CA	SER	A	347	-20.181	-56.034	24.167	1.00 32.82	C
ATOM	2607	C	SER	A	347	-19.114	-56.690	25.025	1.00 30.94	C
ATOM	2608	O	SER	A	347	-18.376	-57.556	24.564	1.00 30.27	O
ATOM	2609	CB	SER	A	347	-21.512	-56.782	24.320	1.00 37.63	C
ATOM	2610	OG	SER	A	347	-21.906	-56.870	25.684	1.00 44.65	O
ATOM	2611	N	LYS	A	348	-19.029	-56.284	26.283	1.00 30.04	N
ATOM	2612	CA	LYS	A	348	-18.106	-56.942	27.185	1.00 31.61	C
ATOM	2613	C	LYS	A	348	-18.701	-58.289	27.569	1.00 30.55	C
ATOM	2614	O	LYS	A	348	-18.005	-59.162	28.086	1.00 32.59	O
ATOM	2615	CB	LYS	A	348	-17.876	-56.093	28.440	1.00 36.30	C
ATOM	2616	CG	LYS	A	348	-18.929	-56.293	29.533	1.00 26.56	C
ATOM	2617	CD	LYS	A	348	-18.602	-55.505	30.795	1.00 32.36	C
ATOM	2618	CE	LYS	A	348	-19.544	-55.881	31.936	1.00 28.29	C
ATOM	2619	NZ	LYS	A	348	-19.302	-57.290	32.403	1.00 24.87	N
ATOM	2620	N	ASP	A	349	-20.000	-58.443	27.317	1.00 32.19	N
ATOM	2621	CA	ASP	A	349	-20.740	-59.623	27.772	1.00 39.76	C
ATOM	2622	C	ASP	A	349	-20.886	-60.733	26.726	1.00 42.12	C
ATOM	2623	O	ASP	A	349	-21.442	-61.785	27.026	1.00 42.87	O
ATOM	2624	CB	ASP	A	349	-22.116	-59.229	28.318	1.00 32.87	C
ATOM	2625	CG	ASP	A	349	-22.028	-58.456	29.625	1.00 34.27	C
ATOM	2626	OD1	ASP	A	349	-21.254	-58.877	30.518	1.00 31.14	O
ATOM	2627	OD2	ASP	A	349	-22.733	-57.427	29.751	1.00 30.90	O
ATOM	2628	N	ASN	A	350	-20.410	-60.501	25.504	1.00 36.31	N
ATOM	2629	CA	ASN	A	350	-20.195	-61.614	24.575	1.00 40.40	C
ATOM	2630	C	ASN	A	350	-18.822	-61.593	23.912	1.00 39.83	C
ATOM	2631	O	ASN	A	350	-17.955	-60.783	24.273	1.00 34.61	O
ATOM	2632	CB	ASN	A	350	-21.328	-61.766	23.546	1.00 32.02	C
ATOM	2633	CG	ASN	A	350	-21.565	-60.517	22.736	1.00 36.99	C
ATOM	2634	OD1	ASN	A	350	-20.627	-59.890	22.245	1.00 41.10	O
ATOM	2635	ND2	ASN	A	350	-22.837	-60.144	22.598	1.00 32.34	N
ATOM	2636	N	GLU	A	351	-18.623	-62.499	22.959	1.00 31.05	N
ATOM	2637	CA	GLU	A	351	-17.339	-62.624	22.282	1.00 34.11	C
ATOM	2638	C	GLU	A	351	-17.119	-61.509	21.261	1.00 27.96	C
ATOM	2639	O	GLU	A	351	-16.048	-61.406	20.675	1.00 28.93	O
ATOM	2640	CB	GLU	A	351	-17.203	-63.998	21.608	1.00 37.14	C
ATOM	2641	CG	GLU	A	351	-16.947	-65.147	22.565	1.00 37.42	C
ATOM	2642	CD	GLU	A	351	-15.819	-64.838	23.542	1.00 54.91	C

ATOM	2643	OE1	GLU	A	351	-14.647	-65.142	23.223	1.00	53.57	O
ATOM	2644	OE2	GLU	A	351	-16.102	-64.286	24.634	1.00	58.71	O
ATOM	2645	N	SER	A	352	-18.137	-60.682	21.062	1.00	26.02	N
ATOM	2646	CA	SER	A	352	-18.083	-59.606	20.080	1.00	36.19	C
ATOM	2647	C	SER	A	352	-17.534	-60.081	18.728	1.00	36.49	C
ATOM	2648	O	SER	A	352	-16.680	-59.430	18.127	1.00	32.39	O
ATOM	2649	CB	SER	A	352	-17.258	-58.435	20.619	1.00	33.77	C
ATOM	2650	OG	SER	A	352	-17.900	-57.848	21.737	1.00	32.52	O
ATOM	2651	N	LEU	A	353	-18.016	-61.229	18.267	1.00	35.45	N
ATOM	2652	CA	LEU	A	353	-17.618	-61.758	16.969	1.00	33.17	C
ATOM	2653	C	LEU	A	353	-18.418	-61.063	15.885	1.00	32.82	C
ATOM	2654	O	LEU	A	353	-19.489	-61.528	15.516	1.00	41.45	O
ATOM	2655	CB	LEU	A	353	-17.880	-63.256	16.925	1.00	29.84	C
ATOM	2656	CG	LEU	A	353	-17.030	-64.044	17.915	1.00	31.06	C
ATOM	2657	CD1	LEU	A	353	-17.513	-65.483	18.006	1.00	27.72	C
ATOM	2658	CD2	LEU	A	353	-15.571	-63.985	17.499	1.00	29.65	C
ATOM	2659	N	ILE	A	354	-17.908	-59.942	15.384	1.00	32.32	N
ATOM	2660	CA	ILE	A	354	-18.671	-59.105	14.453	1.00	33.21	C
ATOM	2661	C	ILE	A	354	-18.651	-59.584	12.995	1.00	40.34	C
ATOM	2662	O	ILE	A	354	-17.822	-60.407	12.592	1.00	33.08	O
ATOM	2663	CB	ILE	A	354	-18.182	-57.649	14.475	1.00	32.94	C
ATOM	2664	CG1	ILE	A	354	-16.689	-57.588	14.117	1.00	31.25	C
ATOM	2665	CG2	ILE	A	354	-18.448	-57.022	15.833	1.00	35.62	C
ATOM	2666	CD1	ILE	A	354	-16.155	-56.188	13.984	1.00	25.38	C
ATOM	2667	N	SER	A	355	-19.581	-59.041	12.217	1.00	33.65	N
ATOM	2668	CA	SER	A	355	-19.701	-59.337	10.800	1.00	31.88	C
ATOM	2669	C	SER	A	355	-18.866	-58.337	10.015	1.00	35.78	C
ATOM	2670	O	SER	A	355	-18.407	-57.322	10.565	1.00	30.30	O
ATOM	2671	CB	SER	A	355	-21.164	-59.210	10.365	1.00	24.45	C
ATOM	2672	OG	SER	A	355	-21.570	-57.847	10.390	1.00	27.73	O
ATOM	2673	N	ARG	A	356	-18.691	-58.610	8.724	1.00	30.95	N
ATOM	2674	CA	ARG	A	356	-17.917	-57.729	7.864	1.00	26.47	C
ATOM	2675	C	ARG	A	356	-18.580	-56.363	7.787	1.00	26.84	C
ATOM	2676	O	ARG	A	356	-17.909	-55.330	7.876	1.00	32.00	O
ATOM	2677	CB	ARG	A	356	-17.739	-58.340	6.473	1.00	34.45	C
ATOM	2678	CG	ARG	A	356	-17.099	-57.404	5.445	1.00	35.75	C
ATOM	2679	CD	ARG	A	356	-15.615	-57.652	5.345	1.00	33.66	C
ATOM	2680	NE	ARG	A	356	-14.957	-56.715	4.445	1.00	33.44	N
ATOM	2681	CZ	ARG	A	356	-13.680	-56.362	4.554	1.00	34.14	C
ATOM	2682	NH1	ARG	A	356	-12.934	-56.875	5.530	1.00	30.29	N
ATOM	2683	NH2	ARG	A	356	-13.153	-55.488	3.701	1.00	24.27	N
ATOM	2684	N	ALA	A	357	-19.902	-56.353	7.656	1.00	28.18	N
ATOM	2685	CA	ALA	A	357	-20.644	-55.094	7.617	1.00	30.52	C
ATOM	2686	C	ALA	A	357	-20.457	-54.276	8.903	1.00	33.44	C

ATOM	2687	O	ALA	A	357	-20.321	-53.054	8.853	1.00	35.10	O
ATOM	2688	CB	ALA	A	357	-22.119	-55.352	7.350	1.00	23.37	C
ATOM	2689	N	GLU	A	358	-20.445	-54.946	10.053	1.00	32.97	N
ATOM	2690	CA	GLU	A	358	-20.228	-54.252	11.320	1.00	33.01	C
ATOM	2691	C	GLU	A	358	-18.795	-53.725	11.393	1.00	31.64	C
ATOM	2692	O	GLU	A	358	-18.557	-52.607	11.845	1.00	28.43	O
ATOM	2693	CB	GLU	A	358	-20.523	-55.170	12.507	1.00	30.45	C
ATOM	2694	CG	GLU	A	358	-21.997	-55.464	12.697	1.00	30.65	C
ATOM	2695	CD	GLU	A	358	-22.265	-56.532	13.752	1.00	34.65	C
ATOM	2696	OE1	GLU	A	358	-21.491	-57.510	13.854	1.00	35.45	O
ATOM	2697	OE2	GLU	A	358	-23.263	-56.388	14.484	1.00	42.45	O
ATOM	2698	N	PHE	A	359	-17.848	-54.531	10.929	1.00	28.58	N
ATOM	2699	CA	PHE	A	359	-16.465	-54.087	10.844	1.00	33.25	C
ATOM	2700	C	PHE	A	359	-16.362	-52.813	10.003	1.00	31.16	C
ATOM	2701	O	PHE	A	359	-15.742	-51.838	10.424	1.00	35.53	O
ATOM	2702	CB	PHE	A	359	-15.566	-55.205	10.295	1.00	32.11	C
ATOM	2703	CG	PHE	A	359	-14.183	-54.746	9.915	1.00	34.27	C
ATOM	2704	CD1	PHE	A	359	-13.169	-54.690	10.861	1.00	32.84	C
ATOM	2705	CD2	PHE	A	359	-13.894	-54.384	8.605	1.00	31.73	C
ATOM	2706	CE1	PHE	A	359	-11.884	-54.278	10.512	1.00	27.55	C
ATOM	2707	CE2	PHE	A	359	-12.612	-53.966	8.245	1.00	31.88	C
ATOM	2708	CZ	PHE	A	359	-11.607	-53.911	9.202	1.00	34.61	C
ATOM	2709	N	LEU	A	360	-16.991	-52.812	8.832	1.00	30.77	N
ATOM	2710	CA	LEU	A	360	-16.969	-51.641	7.956	1.00	27.06	C
ATOM	2711	C	LEU	A	360	-17.576	-50.396	8.612	1.00	28.39	C
ATOM	2712	O	LEU	A	360	-17.021	-49.303	8.519	1.00	29.80	O
ATOM	2713	CB	LEU	A	360	-17.668	-51.944	6.626	1.00	29.49	C
ATOM	2714	CG	LEU	A	360	-16.979	-52.942	5.686	1.00	36.32	C
ATOM	2715	CD1	LEU	A	360	-17.721	-53.020	4.349	1.00	25.45	C
ATOM	2716	CD2	LEU	A	360	-15.504	-52.589	5.469	1.00	25.35	C
ATOM	2717	N	ALA	A	361	-18.721	-50.555	9.265	1.00	28.87	N
ATOM	2718	CA	ALA	A	361	-19.323	-49.448	9.997	1.00	29.54	C
ATOM	2719	C	ALA	A	361	-18.423	-49.047	11.173	1.00	34.49	C
ATOM	2720	O	ALA	A	361	-18.246	-47.859	11.458	1.00	32.83	O
ATOM	2721	CB	ALA	A	361	-20.707	-49.837	10.500	1.00	31.49	C
ATOM	2722	N	GLY	A	362	-17.856	-50.044	11.848	1.00	23.72	N
ATOM	2723	CA	GLY	A	362	-16.938	-49.798	12.948	1.00	28.52	C
ATOM	2724	C	GLY	A	362	-15.755	-48.912	12.575	1.00	31.24	C
ATOM	2725	O	GLY	A	362	-15.322	-48.079	13.376	1.00	25.92	O
ATOM	2726	N	VAL	A	363	-15.241	-49.085	11.357	1.00	31.03	N
ATOM	2727	CA	VAL	A	363	-14.105	-48.303	10.870	1.00	25.40	C
ATOM	2728	C	VAL	A	363	-14.417	-46.805	10.811	1.00	25.29	C
ATOM	2729	O	VAL	A	363	-13.567	-45.986	11.148	1.00	29.50	O
ATOM	2730	CB	VAL	A	363	-13.608	-48.813	9.488	1.00	26.46	C

ATOM	2731	CG1	VAL	A	363	-12.699	-47.786	8.796	1.00	22.87	C
ATOM	2732	CG2	VAL	A	363	-12.897	-50.140	9.637	1.00	21.44	C
ATOM	2733	N	ARG	A	364	-15.626	-46.433	10.394	1.00	28.11	N
ATOM	2734	CA	ARG	A	364	-15.975	-45.011	10.354	1.00	24.72	C
ATOM	2735	C	ARG	A	364	-16.128	-44.451	11.768	1.00	29.34	C
ATOM	2736	O	ARG	A	364	-15.956	-43.254	11.984	1.00	29.49	O
ATOM	2737	CB	ARG	A	364	-17.262	-44.744	9.570	1.00	26.64	C
ATOM	2738	CG	ARG	A	364	-17.302	-45.278	8.136	1.00	34.70	C
ATOM	2739	CD	ARG	A	364	-16.034	-45.001	7.338	1.00	26.86	C
ATOM	2740	NE	ARG	A	364	-15.756	-43.586	7.089	1.00	32.05	N
ATOM	2741	CZ	ARG	A	364	-16.067	-42.946	5.964	1.00	34.02	C
ATOM	2742	NH1	ARG	A	364	-16.701	-43.582	4.991	1.00	29.08	N
ATOM	2743	NH2	ARG	A	364	-15.754	-41.665	5.814	1.00	28.57	N
ATOM	2744	N	VAL	A	365	-16.457	-45.315	12.726	1.00	26.09	N
ATOM	2745	CA	VAL	A	365	-16.607	-44.879	14.111	1.00	32.56	C
ATOM	2746	C	VAL	A	365	-15.237	-44.736	14.774	1.00	28.28	C
ATOM	2747	O	VAL	A	365	-14.977	-43.773	15.496	1.00	29.22	O
ATOM	2748	CB	VAL	A	365	-17.532	-45.819	14.918	1.00	26.99	C
ATOM	2749	CG1	VAL	A	365	-17.577	-45.410	16.386	1.00	25.61	C
ATOM	2750	CG2	VAL	A	365	-18.937	-45.796	14.332	1.00	27.11	C
ATOM	2751	N	GLY	A	366	-14.355	-45.687	14.498	1.00	26.78	N
ATOM	2752	CA	GLY	A	366	-13.009	-45.645	15.027	1.00	25.34	C
ATOM	2753	C	GLY	A	366	-12.141	-44.594	14.362	1.00	27.53	C
ATOM	2754	O	GLY	A	366	-11.157	-44.147	14.939	1.00	29.70	O
ATOM	2755	N	VAL	A	367	-12.485	-44.208	13.134	1.00	33.98	N
ATOM	2756	CA	VAL	A	367	-11.718	-43.184	12.422	1.00	27.43	C
ATOM	2757	C	VAL	A	367	-12.651	-42.101	11.907	1.00	27.81	C
ATOM	2758	O	VAL	A	367	-12.831	-41.949	10.690	1.00	29.82	O
ATOM	2759	CB	VAL	A	367	-10.900	-43.780	11.246	1.00	29.63	C
ATOM	2760	CG1	VAL	A	367	-9.855	-42.783	10.756	1.00	26.38	C
ATOM	2761	CG2	VAL	A	367	-10.225	-45.074	11.666	1.00	25.98	C
ATOM	2762	N	PRO	A	368	-13.235	-41.323	12.835	1.00	30.81	N
ATOM	2763	CA	PRO	A	368	-14.275	-40.358	12.468	1.00	26.43	C
ATOM	2764	C	PRO	A	368	-13.747	-39.202	11.613	1.00	29.52	C
ATOM	2765	O	PRO	A	368	-12.557	-38.898	11.630	1.00	33.13	O
ATOM	2766	CB	PRO	A	368	-14.767	-39.848	13.832	1.00	28.83	C
ATOM	2767	CG	PRO	A	368	-13.597	-40.019	14.744	1.00	26.00	C
ATOM	2768	CD	PRO	A	368	-12.931	-41.285	14.284	1.00	26.00	C
ATOM	2769	N	GLN	A	369	-14.645	-38.584	10.857	1.00	29.14	N
ATOM	2770	CA	GLN	A	369	-14.317	-37.416	10.035	1.00	37.95	C
ATOM	2771	C	GLN	A	369	-13.113	-37.516	9.082	1.00	37.72	C
ATOM	2772	O	GLN	A	369	-12.355	-36.555	8.928	1.00	35.60	O
ATOM	2773	CB	GLN	A	369	-14.185	-36.177	10.917	1.00	41.01	C
ATOM	2774	CG	GLN	A	369	-15.512	-35.680	11.475	1.00	39.17	C

```
ATOM   2775  CD  GLN A 369     -15.305 -34.529  12.412  1.00 43.50           C
ATOM   2776  OE1 GLN A 369     -14.681 -34.686  13.463  1.00 52.78           O
ATOM   2777  NE2 GLN A 369     -15.797 -33.352  12.035  1.00 35.79           N
ATOM   2778  N   VAL A 370     -12.941 -38.661   8.433  1.00 34.38           N
ATOM   2779  CA  VAL A 370     -12.026 -38.721   7.304  1.00 28.71           C
ATOM   2780  C   VAL A 370     -12.837 -39.050   6.068  1.00 32.20           C
ATOM   2781  O   VAL A 370     -13.960 -39.547   6.165  1.00 31.56           O
ATOM   2782  CB  VAL A 370     -10.885 -39.763   7.480  1.00 30.10           C
ATOM   2783  CG1 VAL A 370     -10.027 -39.438   8.703  1.00 22.84           C
ATOM   2784  CG2 VAL A 370     -11.439 -41.184   7.543  1.00 22.56           C
ATOM   2785  N   SER A 371     -12.260 -38.777   4.906  1.00 28.81           N
ATOM   2786  CA  SER A 371     -12.924 -39.048   3.640  1.00 32.80           C
ATOM   2787  C   SER A 371     -13.165 -40.535   3.448  1.00 35.63           C
ATOM   2788  O   SER A 371     -12.501 -41.377   4.076  1.00 32.38           O
ATOM   2789  CB  SER A 371     -12.065 -38.536   2.492  1.00 31.59           C
ATOM   2790  OG  SER A 371     -10.817 -39.210   2.483  1.00 39.99           O
ATOM   2791  N   ASP A 372     -14.121 -40.849   2.577  1.00 34.78           N
ATOM   2792  CA  ASP A 372     -14.367 -42.224   2.157  1.00 37.81           C
ATOM   2793  C   ASP A 372     -13.090 -42.885   1.635  1.00 35.69           C
ATOM   2794  O   ASP A 372     -12.801 -44.040   1.963  1.00 32.05           O
ATOM   2795  CB  ASP A 372     -15.458 -42.271   1.081  1.00 38.64           C
ATOM   2796  CG  ASP A 372     -16.815 -41.838   1.603  1.00 44.44           C
ATOM   2797  OD1 ASP A 372     -16.997 -41.790   2.844  1.00 44.99           O
ATOM   2798  OD2 ASP A 372     -17.701 -41.548   0.769  1.00 47.59           O
ATOM   2799  N   LEU A 373     -12.327 -42.155   0.824  1.00 33.87           N
ATOM   2800  CA  LEU A 373     -11.060 -42.688   0.338  1.00 36.02           C
ATOM   2801  C   LEU A 373     -10.138 -43.109   1.496  1.00 33.76           C
ATOM   2802  O   LEU A 373      -9.591 -44.213   1.489  1.00 31.73           O
ATOM   2803  CB  LEU A 373     -10.348 -41.687  -0.569  1.00 28.18           C
ATOM   2804  CG  LEU A 373      -9.114 -42.294  -1.239  1.00 32.22           C
ATOM   2805  CD1 LEU A 373      -9.514 -43.482  -2.118  1.00 31.60           C
ATOM   2806  CD2 LEU A 373      -8.356 -41.252  -2.041  1.00 34.94           C
ATOM   2807  N   ALA A 374      -9.983 -42.237   2.489  1.00 32.17           N
ATOM   2808  CA  ALA A 374      -9.138 -42.539   3.646  1.00 26.49           C
ATOM   2809  C   ALA A 374      -9.651 -43.768   4.382  1.00 27.42           C
ATOM   2810  O   ALA A 374      -8.868 -44.643   4.773  1.00 25.71           O
ATOM   2811  CB  ALA A 374      -9.063 -41.349   4.579  1.00 24.26           C
ATOM   2812  N   ALA A 375     -10.967 -43.842   4.555  1.00 25.32           N
ATOM   2813  CA  ALA A 375     -11.583 -45.009   5.185  1.00 25.32           C
ATOM   2814  C   ALA A 375     -11.312 -46.288   4.386  1.00 32.41           C
ATOM   2815  O   ALA A 375     -11.023 -47.341   4.968  1.00 32.15           O
ATOM   2816  CB  ALA A 375     -13.074 -44.796   5.355  1.00 23.99           C
ATOM   2817  N   GLU A 376     -11.411 -46.189   3.058  1.00 31.81           N
ATOM   2818  CA  GLU A 376     -11.112 -47.309   2.166  1.00 28.20           C
```

```
ATOM   2819  C   GLU A 376      -9.687 -47.798   2.371  1.00 25.95           C
ATOM   2820  O   GLU A 376      -9.428 -49.001   2.432  1.00 27.84           O
ATOM   2821  CB  GLU A 376     -11.289 -46.896   0.699  1.00 34.23           C
ATOM   2822  CG  GLU A 376     -12.732 -46.803   0.227  1.00 41.94           C
ATOM   2823  CD  GLU A 376     -13.493 -48.088   0.461  1.00 49.51           C
ATOM   2824  OE1 GLU A 376     -12.938 -49.174   0.186  1.00 52.84           O
ATOM   2825  OE2 GLU A 376     -14.646 -48.013   0.933  1.00 66.88           O
ATOM   2826  N   ALA A 377      -8.757 -46.855   2.468  1.00 24.20           N
ATOM   2827  CA  ALA A 377      -7.366 -47.201   2.720  1.00 25.54           C
ATOM   2828  C   ALA A 377      -7.208 -47.950   4.048  1.00 31.40           C
ATOM   2829  O   ALA A 377      -6.424 -48.900   4.132  1.00 32.08           O
ATOM   2830  CB  ALA A 377      -6.495 -45.960   2.693  1.00 26.18           C
ATOM   2831  N   VAL A 378      -7.950 -47.536   5.079  1.00 22.64           N
ATOM   2832  CA  VAL A 378      -7.901 -48.250   6.349  1.00 27.13           C
ATOM   2833  C   VAL A 378      -8.371 -49.696   6.172  1.00 26.04           C
ATOM   2834  O   VAL A 378      -7.703 -50.640   6.603  1.00 27.58           O
ATOM   2835  CB  VAL A 378      -8.736 -47.558   7.447  1.00 29.30           C
ATOM   2836  CG1 VAL A 378      -8.714 -48.396   8.725  1.00 23.62           C
ATOM   2837  CG2 VAL A 378      -8.217 -46.149   7.711  1.00 22.47           C
ATOM   2838  N   VAL A 379      -9.512 -49.859   5.515  1.00 26.25           N
ATOM   2839  CA  VAL A 379     -10.075 -51.180   5.259  1.00 27.00           C
ATOM   2840  C   VAL A 379      -9.117 -52.069   4.468  1.00 32.46           C
ATOM   2841  O   VAL A 379      -9.012 -53.274   4.730  1.00 31.44           O
ATOM   2842  CB  VAL A 379     -11.410 -51.064   4.507  1.00 31.31           C
ATOM   2843  CG1 VAL A 379     -11.891 -52.427   4.065  1.00 32.95           C
ATOM   2844  CG2 VAL A 379     -12.449 -50.397   5.390  1.00 27.37           C
ATOM   2845  N   LEU A 380      -8.410 -51.470   3.512  1.00 25.49           N
ATOM   2846  CA  LEU A 380      -7.421 -52.202   2.726  1.00 29.31           C
ATOM   2847  C   LEU A 380      -6.313 -52.773   3.600  1.00 29.31           C
ATOM   2848  O   LEU A 380      -6.029 -53.975   3.555  1.00 26.14           O
ATOM   2849  CB  LEU A 380      -6.796 -51.311   1.636  1.00 28.14           C
ATOM   2850  CG  LEU A 380      -5.532 -51.888   0.982  1.00 28.32           C
ATOM   2851  CD1 LEU A 380      -5.861 -53.039   0.016  1.00 25.95           C
ATOM   2852  CD2 LEU A 380      -4.702 -50.800   0.301  1.00 26.95           C
ATOM   2853  N   HIS A 381      -5.689 -51.901   4.386  0.54 27.06           N
ATOM   2855  CA  HIS A 381      -4.569 -52.295   5.229  0.54 29.28           C
ATOM   2857  C   HIS A 381      -4.988 -53.341   6.258  0.54 30.41           C
ATOM   2859  O   HIS A 381      -4.246 -54.287   6.539  0.54 30.39           O
ATOM   2861  CB  HIS A 381      -3.989 -51.068   5.940  0.54 29.71           C
ATOM   2863  CG  HIS A 381      -2.559 -51.225   6.354  0.54 29.59           C
ATOM   2865  ND1 HIS A 381      -1.511 -51.116   5.464  0.54 29.53           N
ATOM   2867  CD2 HIS A 381      -2.001 -51.478   7.563  0.54 30.53           C
ATOM   2869  CE1 HIS A 381      -0.370 -51.297   6.106  0.54 32.85           C
ATOM   2871  NE2 HIS A 381      -0.639 -51.523   7.380  0.54 32.75           N
```

```
ATOM   2873  N   TYR A 382      -6.190 -53.179   6.802  1.00 27.90           N
ATOM   2874  CA  TYR A 382      -6.626 -53.988   7.940  1.00 28.68           C
ATOM   2875  C   TYR A 382      -7.486 -55.204   7.607  1.00 29.15           C
ATOM   2876  O   TYR A 382      -7.926 -55.925   8.505  1.00 27.00           O
ATOM   2877  CB  TYR A 382      -7.295 -53.109   9.003  1.00 24.67           C
ATOM   2878  CG  TYR A 382      -6.291 -52.326   9.802  1.00 26.18           C
ATOM   2879  CD1 TYR A 382      -5.755 -52.841  10.980  1.00 20.42           C
ATOM   2880  CD2 TYR A 382      -5.856 -51.079   9.369  1.00 25.68           C
ATOM   2881  CE1 TYR A 382      -4.820 -52.127  11.707  1.00 21.55           C
ATOM   2882  CE2 TYR A 382      -4.922 -50.358  10.086  1.00 24.46           C
ATOM   2883  CZ  TYR A 382      -4.410 -50.884  11.260  1.00 26.20           C
ATOM   2884  OH  TYR A 382      -3.478 -50.161  11.973  1.00 21.98           O
ATOM   2885  N   THR A 383      -7.719 -55.443   6.323  1.00 30.28           N
ATOM   2886  CA  THR A 383      -8.404 -56.661   5.920  1.00 24.73           C
ATOM   2887  C   THR A 383      -7.377 -57.734   5.594  1.00 26.99           C
ATOM   2888  O   THR A 383      -6.368 -57.454   4.952  1.00 27.46           O
ATOM   2889  CB  THR A 383      -9.326 -56.413   4.712  1.00 27.08           C
ATOM   2890  OG1 THR A 383     -10.399 -55.543   5.100  1.00 26.64           O
ATOM   2891  CG2 THR A 383      -9.916 -57.717   4.189  1.00 25.21           C
ATOM   2892  N   ASP A 384      -7.618 -58.953   6.068  1.00 26.97           N
ATOM   2893  CA  ASP A 384      -6.903 -60.121   5.574  1.00 24.93           C
ATOM   2894  C   ASP A 384      -7.712 -60.638   4.387  1.00 31.27           C
ATOM   2895  O   ASP A 384      -8.774 -61.231   4.574  1.00 27.91           O
ATOM   2896  CB  ASP A 384      -6.817 -61.185   6.665  1.00 28.57           C
ATOM   2897  CG  ASP A 384      -6.093 -62.454   6.212  1.00 35.35           C
ATOM   2898  OD1 ASP A 384      -6.041 -62.742   4.998  1.00 31.44           O
ATOM   2899  OD2 ASP A 384      -5.580 -63.179   7.089  1.00 34.54           O
ATOM   2900  N   TRP A 385      -7.216 -60.424   3.169  1.00 26.91           N
ATOM   2901  CA  TRP A 385      -8.004 -60.757   1.974  1.00 30.08           C
ATOM   2902  C   TRP A 385      -8.095 -62.255   1.650  1.00 27.91           C
ATOM   2903  O   TRP A 385      -8.741 -62.652   0.677  1.00 34.23           O
ATOM   2904  CB  TRP A 385      -7.572 -59.918   0.762  1.00 25.55           C
ATOM   2905  CG  TRP A 385      -7.767 -58.457   1.015  1.00 24.41           C
ATOM   2906  CD1 TRP A 385      -6.837 -57.573   1.491  1.00 22.44           C
ATOM   2907  CD2 TRP A 385      -8.980 -57.711   0.848  1.00 22.18           C
ATOM   2908  NE1 TRP A 385      -7.395 -56.322   1.614  1.00 23.17           N
ATOM   2909  CE2 TRP A 385      -8.707 -56.380   1.223  1.00 20.76           C
ATOM   2910  CE3 TRP A 385     -10.266 -58.037   0.403  1.00 22.99           C
ATOM   2911  CZ2 TRP A 385      -9.675 -55.376   1.170  1.00 24.80           C
ATOM   2912  CZ3 TRP A 385     -11.222 -57.039   0.346  1.00 22.72           C
ATOM   2913  CH2 TRP A 385     -10.923 -55.724   0.731  1.00 24.41           C
ATOM   2914  N   LEU A 386      -7.468 -63.082   2.478  1.00 29.02           N
ATOM   2915  CA  LEU A 386      -7.731 -64.522   2.448  1.00 32.41           C
ATOM   2916  C   LEU A 386      -8.857 -64.926   3.424  1.00 37.64           C
```

ATOM	2917	O	LEU	A	386	-9.390	-66.036	3.348	1.00	33.76	O
ATOM	2918	CB	LEU	A	386	-6.452	-65.304	2.748	1.00	30.71	C
ATOM	2919	CG	LEU	A	386	-5.417	-65.281	1.617	1.00	38.59	C
ATOM	2920	CD1	LEU	A	386	-4.138	-65.965	2.030	1.00	36.26	C
ATOM	2921	CD2	LEU	A	386	-5.997	-65.946	0.402	1.00	31.53	C
ATOM	2922	N	HIS	A	387	-9.203	-64.021	4.340	1.00	35.19	N
ATOM	2923	CA	HIS	A	387	-10.253	-64.263	5.334	1.00	32.55	C
ATOM	2924	C	HIS	A	387	-11.030	-62.979	5.591	1.00	30.04	C
ATOM	2925	O	HIS	A	387	-11.087	-62.514	6.725	1.00	39.05	O
ATOM	2926	CB	HIS	A	387	-9.640	-64.735	6.661	1.00	23.66	C
ATOM	2927	CG	HIS	A	387	-8.718	-65.904	6.524	1.00	29.35	C
ATOM	2928	ND1	HIS	A	387	-9.170	-67.206	6.452	1.00	32.01	N
ATOM	2929	CD2	HIS	A	387	-7.368	-65.969	6.434	1.00	33.28	C
ATOM	2930	CE1	HIS	A	387	-8.138	-68.022	6.324	1.00	30.25	C
ATOM	2931	NE2	HIS	A	387	-7.033	-67.296	6.306	1.00	31.34	N
ATOM	2932	N	PRO	A	388	-11.629	-62.395	4.544	1.00	31.59	N
ATOM	2933	CA	PRO	A	388	-12.196	-61.049	4.697	1.00	32.84	C
ATOM	2934	C	PRO	A	388	-13.389	-60.983	5.645	1.00	36.58	C
ATOM	2935	O	PRO	A	388	-13.710	-59.894	6.126	1.00	36.04	O
ATOM	2936	CB	PRO	A	388	-12.651	-60.695	3.272	1.00	29.02	C
ATOM	2937	CG	PRO	A	388	-12.934	-62.014	2.631	1.00	25.80	C
ATOM	2938	CD	PRO	A	388	-11.900	-62.960	3.206	1.00	33.70	C
ATOM	2939	N	GLU	A	389	-14.020	-62.121	5.922	1.00	32.03	N
ATOM	2940	CA	GLU	A	389	-15.290	-62.117	6.646	1.00	36.97	C
ATOM	2941	C	GLU	A	389	-15.254	-62.881	7.967	1.00	33.58	C
ATOM	2942	O	GLU	A	389	-16.291	-63.072	8.600	1.00	35.49	O
ATOM	2943	CB	GLU	A	389	-16.406	-62.679	5.755	1.00	35.32	C
ATOM	2944	CG	GLU	A	389	-16.495	-62.017	4.387	1.00	38.11	C
ATOM	2945	CD	GLU	A	389	-17.889	-61.516	4.052	1.00	53.90	C
ATOM	2946	OE1	GLU	A	389	-18.863	-61.988	4.681	1.00	52.88	O
ATOM	2947	OE2	GLU	A	389	-18.010	-60.640	3.161	1.00	59.79	O
ATOM	2948	N	ASP	A	390	-14.069	-63.321	8.377	1.00	27.50	N
ATOM	2949	CA	ASP	A	390	-13.924	-64.078	9.611	1.00	31.12	C
ATOM	2950	C	ASP	A	390	-14.103	-63.182	10.845	1.00	34.26	C
ATOM	2951	O	ASP	A	390	-13.291	-62.291	11.098	1.00	32.21	O
ATOM	2952	CB	ASP	A	390	-12.568	-64.783	9.646	1.00	30.62	C
ATOM	2953	CG	ASP	A	390	-12.340	-65.525	10.947	1.00	40.77	C
ATOM	2954	OD1	ASP	A	390	-13.344	-65.885	11.595	1.00	47.65	O
ATOM	2955	OD2	ASP	A	390	-11.167	-65.750	11.328	1.00	44.78	O
ATOM	2956	N	PRO	A	391	-15.174	-63.424	11.616	1.00	39.33	N
ATOM	2957	CA	PRO	A	391	-15.560	-62.585	12.763	1.00	34.43	C
ATOM	2958	C	PRO	A	391	-14.439	-62.303	13.761	1.00	30.94	C
ATOM	2959	O	PRO	A	391	-14.198	-61.133	14.075	1.00	27.53	O
ATOM	2960	CB	PRO	A	391	-16.686	-63.392	13.413	1.00	36.25	C

ATOM	2961	CG	PRO	A	391	-17.340	-64.074	12.222	1.00	34.30	C
ATOM	2962	CD	PRO	A	391	-16.165	-64.486	11.361	1.00	33.40	C
ATOM	2963	N	ALA	A	392	-13.758	-63.339	14.240	1.00	27.80	N
ATOM	2964	CA	ALA	A	392	-12.677	-63.133	15.196	1.00	32.06	C
ATOM	2965	C	ALA	A	392	-11.577	-62.212	14.647	1.00	34.94	C
ATOM	2966	O	ALA	A	392	-11.106	-61.308	15.350	1.00	30.08	O
ATOM	2967	CB	ALA	A	392	-12.098	-64.465	15.659	1.00	33.37	C
ATOM	2968	N	ARG	A	393	-11.182	-62.431	13.394	1.00	29.19	N
ATOM	2969	CA	ARG	A	393	-10.152	-61.602	12.776	1.00	26.17	C
ATOM	2970	C	ARG	A	393	-10.628	-60.161	12.610	1.00	28.86	C
ATOM	2971	O	ARG	A	393	-9.851	-59.223	12.789	1.00	30.19	O
ATOM	2972	CB	ARG	A	393	-9.711	-62.186	11.433	1.00	28.84	C
ATOM	2973	CG	ARG	A	393	-8.782	-63.395	11.537	1.00	31.37	C
ATOM	2974	CD	ARG	A	393	-8.332	-63.781	10.130	1.00	34.11	C
ATOM	2975	NE	ARG	A	393	-7.380	-64.890	10.061	1.00	30.40	N
ATOM	2976	CZ	ARG	A	393	-7.740	-66.168	9.957	1.00	36.71	C
ATOM	2977	NH1	ARG	A	393	-9.027	-66.495	9.951	1.00	35.78	N
ATOM	2978	NH2	ARG	A	393	-6.819	-67.120	9.868	1.00	29.88	N
ATOM	2979	N	LEU	A	394	-11.909	-59.993	12.283	1.00	24.13	N
ATOM	2980	CA	LEU	A	394	-12.510	-58.668	12.178	1.00	24.22	C
ATOM	2981	C	LEU	A	394	-12.495	-57.947	13.522	1.00	29.21	C
ATOM	2982	O	LEU	A	394	-12.227	-56.743	13.588	1.00	26.61	O
ATOM	2983	CB	LEU	A	394	-13.945	-58.764	11.659	1.00	23.20	C
ATOM	2984	CG	LEU	A	394	-14.093	-59.263	10.219	1.00	33.56	C
ATOM	2985	CD1	LEU	A	394	-15.546	-59.581	9.895	1.00	28.58	C
ATOM	2986	CD2	LEU	A	394	-13.553	-58.230	9.256	1.00	23.66	C
ATOM	2987	N	ARG	A	395	-12.791	-58.688	14.588	1.00	29.48	N
ATOM	2988	CA	ARG	A	395	-12.766	-58.135	15.937	1.00	29.95	C
ATOM	2989	C	ARG	A	395	-11.362	-57.636	16.277	1.00	27.14	C
ATOM	2990	O	ARG	A	395	-11.179	-56.463	16.603	1.00	23.89	O
ATOM	2991	CB	ARG	A	395	-13.224	-59.180	16.952	1.00	26.84	C
ATOM	2992	CG	ARG	A	395	-13.375	-58.651	18.369	1.00	31.70	C
ATOM	2993	CD	ARG	A	395	-13.586	-59.801	19.326	1.00	27.33	C
ATOM	2994	NE	ARG	A	395	-12.485	-60.755	19.253	1.00	30.33	N
ATOM	2995	CZ	ARG	A	395	-12.579	-62.040	19.583	1.00	32.82	C
ATOM	2996	NH1	ARG	A	395	-13.734	-62.546	19.994	1.00	31.24	N
ATOM	2997	NH2	ARG	A	395	-11.514	-62.825	19.496	1.00	33.27	N
ATOM	2998	N	GLU	A	396	-10.377	-58.528	16.174	1.00	27.45	N
ATOM	2999	CA	GLU	A	396	-8.971	-58.175	16.366	1.00	28.72	C
ATOM	3000	C	GLU	A	396	-8.525	-57.009	15.479	1.00	28.62	C
ATOM	3001	O	GLU	A	396	-7.791	-56.126	15.925	1.00	30.19	O
ATOM	3002	CB	GLU	A	396	-8.065	-59.389	16.120	1.00	27.94	C
ATOM	3003	CG	GLU	A	396	-7.624	-60.154	17.368	1.00	36.37	C
ATOM	3004	CD	GLU	A	396	-8.782	-60.783	18.149	1.00	46.91	C

```
ATOM   3005  OE1 GLU A 396      -9.597 -60.033  18.738  1.00 49.83           O
ATOM   3006  OE2 GLU A 396      -8.869 -62.034  18.194  1.00 44.31           O
ATOM   3007  N   ALA A 397      -8.974 -56.995  14.230  1.00 29.47           N
ATOM   3008  CA  ALA A 397      -8.507 -55.972  13.300  1.00 27.17           C
ATOM   3009  C   ALA A 397      -9.067 -54.601  13.653  1.00 23.41           C
ATOM   3010  O   ALA A 397      -8.372 -53.597  13.532  1.00 23.74           O
ATOM   3011  CB  ALA A 397      -8.843 -56.346  11.842  1.00 22.82           C
ATOM   3012  N   LEU A 398     -10.323 -54.551  14.084  1.00 21.22           N
ATOM   3013  CA  LEU A 398     -10.900 -53.268  14.443  1.00 20.80           C
ATOM   3014  C   LEU A 398     -10.261 -52.745  15.740  1.00 27.40           C
ATOM   3015  O   LEU A 398     -10.060 -51.539  15.902  1.00 23.80           O
ATOM   3016  CB  LEU A 398     -12.413 -53.351  14.584  1.00 24.83           C
ATOM   3017  CG  LEU A 398     -13.073 -51.977  14.745  1.00 25.96           C
ATOM   3018  CD1 LEU A 398     -12.880 -51.154  13.479  1.00 24.09           C
ATOM   3019  CD2 LEU A 398     -14.540 -52.112  15.090  1.00 29.22           C
ATOM   3020  N   SER A 399      -9.934 -53.661  16.648  1.00 24.53           N
ATOM   3021  CA  SER A 399      -9.189 -53.312  17.846  1.00 27.36           C
ATOM   3022  C   SER A 399      -7.867 -52.660  17.442  1.00 28.12           C
ATOM   3023  O   SER A 399      -7.511 -51.589  17.950  1.00 25.55           O
ATOM   3024  CB  SER A 399      -8.931 -54.560  18.690  1.00 27.84           C
ATOM   3025  OG  SER A 399      -8.081 -54.273  19.791  1.00 30.97           O
ATOM   3026  N   ASP A 400      -7.160 -53.311  16.518  1.00 23.60           N
ATOM   3027  CA  ASP A 400      -5.918 -52.790  15.947  1.00 23.90           C
ATOM   3028  C   ASP A 400      -6.094 -51.414  15.311  1.00 24.80           C
ATOM   3029  O   ASP A 400      -5.243 -50.545  15.467  1.00 23.04           O
ATOM   3030  CB  ASP A 400      -5.375 -53.738  14.877  1.00 27.32           C
ATOM   3031  CG  ASP A 400      -4.716 -54.958  15.461  1.00 31.10           C
ATOM   3032  OD1 ASP A 400      -4.458 -54.963  16.682  1.00 34.65           O
ATOM   3033  OD2 ASP A 400      -4.434 -55.902  14.692  1.00 40.27           O
ATOM   3034  N   VAL A 401      -7.184 -51.217  14.578  1.00 22.86           N
ATOM   3035  CA  VAL A 401      -7.410 -49.920  13.945  1.00 25.06           C
ATOM   3036  C   VAL A 401      -7.458 -48.816  15.002  1.00 24.94           C
ATOM   3037  O   VAL A 401      -6.664 -47.881  14.964  1.00 24.27           O
ATOM   3038  CB  VAL A 401      -8.699 -49.894  13.091  1.00 20.50           C
ATOM   3039  CG1 VAL A 401      -9.069 -48.456  12.722  1.00 17.07           C
ATOM   3040  CG2 VAL A 401      -8.529 -50.746  11.849  1.00 20.91           C
ATOM   3041  N   VAL A 402      -8.380 -48.948  15.952  1.00 24.59           N
ATOM   3042  CA  VAL A 402      -8.568 -47.955  16.998  1.00 25.75           C
ATOM   3043  C   VAL A 402      -7.288 -47.745  17.811  1.00 25.08           C
ATOM   3044  O   VAL A 402      -6.901 -46.611  18.085  1.00 20.68           O
ATOM   3045  CB  VAL A 402      -9.737 -48.349  17.921  1.00 26.04           C
ATOM   3046  CG1 VAL A 402      -9.846 -47.383  19.095  1.00 18.44           C
ATOM   3047  CG2 VAL A 402     -11.040 -48.401  17.115  1.00 23.97           C
ATOM   3048  N   GLY A 403      -6.619 -48.842  18.152  1.00 24.74           N
```

```
ATOM   3049  CA  GLY A 403      -5.397 -48.788  18.937  1.00 26.13           C
ATOM   3050  C   GLY A 403      -4.174 -48.207  18.241  1.00 25.68           C
ATOM   3051  O   GLY A 403      -3.400 -47.459  18.862  1.00 21.02           O
ATOM   3052  N   ASP A 404      -3.992 -48.547  16.962  1.00 20.79           N
ATOM   3053  CA  ASP A 404      -2.836 -48.074  16.205  1.00 20.06           C
ATOM   3054  C   ASP A 404      -2.997 -46.591  15.923  1.00 24.12           C
ATOM   3055  O   ASP A 404      -2.082 -45.782  16.130  1.00 23.52           O
ATOM   3056  CB  ASP A 404      -2.690 -48.832  14.874  1.00 20.13           C
ATOM   3057  CG  ASP A 404      -2.412 -50.321  15.064  1.00 23.47           C
ATOM   3058  OD1 ASP A 404      -2.054 -50.742  16.190  1.00 25.85           O
ATOM   3059  OD2 ASP A 404      -2.566 -51.078  14.081  1.00 25.17           O
ATOM   3060  N   HIS A 405      -4.188 -46.254  15.456  1.00 19.39           N
ATOM   3061  CA  HIS A 405      -4.552 -44.895  15.112  1.00 22.74           C
ATOM   3062  C   HIS A 405      -4.479 -43.947  16.319  1.00 24.69           C
ATOM   3063  O   HIS A 405      -3.904 -42.861  16.239  1.00 26.36           O
ATOM   3064  CB  HIS A 405      -5.961 -44.913  14.510  1.00 19.16           C
ATOM   3065  CG  HIS A 405      -6.535 -43.558  14.258  1.00 26.56           C
ATOM   3066  ND1 HIS A 405      -5.855 -42.580  13.564  1.00 25.09           N
ATOM   3067  CD2 HIS A 405      -7.731 -43.019  14.604  1.00 26.60           C
ATOM   3068  CE1 HIS A 405      -6.611 -41.496  13.486  1.00 25.88           C
ATOM   3069  NE2 HIS A 405      -7.754 -41.737  14.107  1.00 29.98           N
ATOM   3070  N   ASN A 406      -5.052 -44.358  17.439  1.00 21.07           N
ATOM   3071  CA  ASN A 406      -5.116 -43.473  18.597  1.00 25.47           C
ATOM   3072  C   ASN A 406      -3.896 -43.493  19.517  1.00 21.80           C
ATOM   3073  O   ASN A 406      -3.554 -42.468  20.100  1.00 22.88           O
ATOM   3074  CB  ASN A 406      -6.394 -43.714  19.410  1.00 18.56           C
ATOM   3075  CG  ASN A 406      -7.644 -43.260  18.674  1.00 21.51           C
ATOM   3076  OD1 ASN A 406      -7.854 -42.065  18.457  1.00 18.76           O
ATOM   3077  ND2 ASN A 406      -8.482 -44.215  18.291  1.00 19.22           N
ATOM   3078  N   VAL A 407      -3.239 -44.640  19.653  1.00 20.47           N
ATOM   3079  CA  VAL A 407      -2.162 -44.737  20.637  1.00 19.47           C
ATOM   3080  C   VAL A 407      -0.805 -45.177  20.102  1.00 22.42           C
ATOM   3081  O   VAL A 407       0.176 -44.434  20.203  1.00 21.81           O
ATOM   3082  CB  VAL A 407      -2.536 -45.677  21.797  1.00 20.93           C
ATOM   3083  CG1 VAL A 407      -1.385 -45.751  22.799  1.00 19.76           C
ATOM   3084  CG2 VAL A 407      -3.820 -45.200  22.468  1.00 22.32           C
ATOM   3085  N   VAL A 408      -0.747 -46.393  19.562  1.00 17.97           N
ATOM   3086  CA  VAL A 408       0.528 -47.004  19.189  1.00 20.12           C
ATOM   3087  C   VAL A 408       1.324 -46.163  18.183  1.00 23.83           C
ATOM   3088  O   VAL A 408       2.511 -45.887  18.378  1.00 21.98           O
ATOM   3089  CB  VAL A 408       0.335 -48.430  18.616  1.00 21.63           C
ATOM   3090  CG1 VAL A 408       1.671 -49.010  18.211  1.00 20.13           C
ATOM   3091  CG2 VAL A 408      -0.363 -49.339  19.638  1.00 19.04           C
ATOM   3092  N   CYS A 409       0.671 -45.753  17.104  1.00 21.73           N
```

ATOM	3093	CA	CYS	A	409	1.383	-45.014	16.069	1.00	24.08	C
ATOM	3094	C	CYS	A	409	1.729	-43.578	16.463	1.00	24.52	C
ATOM	3095	O	CYS	A	409	2.785	-43.079	16.072	1.00	24.47	O
ATOM	3096	CB	CYS	A	409	0.651	-45.093	14.721	1.00	20.37	C
ATOM	3097	SG	CYS	A	409	0.455	-46.808	14.161	1.00	21.78	S
ATOM	3098	N	PRO	A	410	0.846	-42.897	17.221	1.00	25.26	N
ATOM	3099	CA	PRO	A	410	1.335	-41.609	17.730	1.00	23.93	C
ATOM	3100	C	PRO	A	410	2.529	-41.747	18.689	1.00	23.85	C
ATOM	3101	O	PRO	A	410	3.424	-40.898	18.651	1.00	23.22	O
ATOM	3102	CB	PRO	A	410	0.111	-41.028	18.439	1.00	24.24	C
ATOM	3103	CG	PRO	A	410	-1.039	-41.583	17.673	1.00	20.33	C
ATOM	3104	CD	PRO	A	410	-0.624	-42.993	17.317	1.00	19.27	C
ATOM	3105	N	VAL	A	411	2.557	-42.797	19.508	1.00	21.90	N
ATOM	3106	CA	VAL	A	411	3.678	-43.015	20.422	1.00	23.44	C
ATOM	3107	C	VAL	A	411	4.941	-43.378	19.634	1.00	25.90	C
ATOM	3108	O	VAL	A	411	6.028	-42.873	19.910	1.00	23.81	O
ATOM	3109	CB	VAL	A	411	3.359	-44.099	21.490	1.00	24.76	C
ATOM	3110	CG1	VAL	A	411	4.626	-44.562	22.212	1.00	22.20	C
ATOM	3111	CG2	VAL	A	411	2.347	-43.583	22.486	1.00	16.43	C
ATOM	3112	N	ALA	A	412	4.783	-44.236	18.635	1.00	24.47	N
ATOM	3113	CA	ALA	A	412	5.903	-44.631	17.783	1.00	26.37	C
ATOM	3114	C	ALA	A	412	6.522	-43.425	17.088	1.00	23.66	C
ATOM	3115	O	ALA	A	412	7.741	-43.255	17.066	1.00	28.37	O
ATOM	3116	CB	ALA	A	412	5.453	-45.661	16.757	1.00	24.17	C
ATOM	3117	N	GLN	A	413	5.676	-42.578	16.525	1.00	24.46	N
ATOM	3118	CA	GLN	A	413	6.150	-41.378	15.861	1.00	24.19	C
ATOM	3119	C	GLN	A	413	6.882	-40.453	16.830	1.00	27.61	C
ATOM	3120	O	GLN	A	413	7.942	-39.921	16.505	1.00	25.97	O
ATOM	3121	CB	GLN	A	413	4.985	-40.632	15.221	1.00	28.47	C
ATOM	3122	CG	GLN	A	413	5.304	-39.183	14.902	1.00	28.52	C
ATOM	3123	CD	GLN	A	413	5.042	-38.848	13.465	1.00	36.45	C
ATOM	3124	OE1	GLN	A	413	4.047	-38.201	13.145	1.00	53.07	O
ATOM	3125	NE2	GLN	A	413	5.930	-39.295	12.577	1.00	42.93	N
ATOM	3126	N	LEU	A	414	6.308	-40.253	18.016	1.00	26.71	N
ATOM	3127	CA	LEU	A	414	6.975	-39.469	19.050	1.00	23.78	C
ATOM	3128	C	LEU	A	414	8.352	-40.048	19.383	1.00	25.72	C
ATOM	3129	O	LEU	A	414	9.340	-39.321	19.456	1.00	25.03	O
ATOM	3130	CB	LEU	A	414	6.134	-39.416	20.322	1.00	24.33	C
ATOM	3131	CG	LEU	A	414	6.836	-38.645	21.442	1.00	28.17	C
ATOM	3132	CD1	LEU	A	414	6.942	-37.178	21.073	1.00	28.96	C
ATOM	3133	CD2	LEU	A	414	6.100	-38.792	22.719	1.00	30.66	C
ATOM	3134	N	ALA	A	415	8.410	-41.362	19.570	1.00	19.11	N
ATOM	3135	CA	ALA	A	415	9.648	-42.017	19.964	1.00	21.51	C
ATOM	3136	C	ALA	A	415	10.714	-41.909	18.869	1.00	30.91	C

ATOM	3137	O	ALA	A	415	11.889	-41.670	19.159	1.00	27.97	O
ATOM	3138	CB	ALA	A	415	9.384	-43.473	20.312	1.00	23.90	C
ATOM	3139	N	GLY	A	416	10.297	-42.071	17.615	1.00	27.27	N
ATOM	3140	CA	GLY	A	416	11.217	-42.012	16.494	1.00	22.95	C
ATOM	3141	C	GLY	A	416	11.812	-40.634	16.304	1.00	22.46	C
ATOM	3142	O	GLY	A	416	13.024	-40.470	16.176	1.00	30.69	O
ATOM	3143	N	ARG	A	417	10.959	-39.625	16.293	1.00	25.00	N
ATOM	3144	CA	ARG	A	417	11.434	-38.264	16.130	1.00	26.73	C
ATOM	3145	C	ARG	A	417	12.311	-37.822	17.296	1.00	34.63	C
ATOM	3146	O	ARG	A	417	13.356	-37.207	17.083	1.00	39.13	O
ATOM	3147	CB	ARG	A	417	10.263	-37.305	15.938	1.00	30.99	C
ATOM	3148	CG	ARG	A	417	9.570	-37.457	14.595	1.00	37.21	C
ATOM	3149	CD	ARG	A	417	10.598	-37.612	13.486	1.00	36.81	C
ATOM	3150	NE	ARG	A	417	10.101	-37.140	12.199	1.00	62.44	N
ATOM	3151	CZ	ARG	A	417	10.375	-35.942	11.693	1.00	56.80	C
ATOM	3152	NH1	ARG	A	417	11.145	-35.097	12.367	1.00	59.03	N
ATOM	3153	NH2	ARG	A	417	9.880	-35.590	10.514	1.00	49.35	N
ATOM	3154	N	LEU	A	418	11.897	-38.130	18.524	1.00	28.64	N
ATOM	3155	CA	LEU	A	418	12.686	-37.719	19.685	1.00	30.44	C
ATOM	3156	C	LEU	A	418	14.071	-38.364	19.632	1.00	26.99	C
ATOM	3157	O	LEU	A	418	15.077	-37.682	19.780	1.00	32.27	O
ATOM	3158	CB	LEU	A	418	11.983	-38.033	21.019	1.00	21.13	C
ATOM	3159	CG	LEU	A	418	10.696	-37.288	21.418	1.00	31.55	C
ATOM	3160	CD1	LEU	A	418	10.337	-37.511	22.886	1.00	24.60	C
ATOM	3161	CD2	LEU	A	418	10.769	-35.814	21.142	1.00	32.52	C
ATOM	3162	N	ALA	A	419	14.117	-39.675	19.414	1.00	25.08	N
ATOM	3163	CA	ALA	A	419	15.390	-40.392	19.302	1.00	28.50	C
ATOM	3164	C	ALA	A	419	16.276	-39.856	18.169	1.00	37.35	C
ATOM	3165	O	ALA	A	419	17.477	-39.650	18.359	1.00	38.50	O
ATOM	3166	CB	ALA	A	419	15.150	-41.892	19.119	1.00	22.50	C
ATOM	3167	N	ALA	A	420	15.686	-39.630	16.996	1.00	31.96	N
ATOM	3168	CA	ALA	A	420	16.450	-39.135	15.849	1.00	34.66	C
ATOM	3169	C	ALA	A	420	16.887	-37.686	16.020	1.00	35.33	C
ATOM	3170	O	ALA	A	420	17.733	-37.204	15.275	1.00	40.38	O
ATOM	3171	CB	ALA	A	420	15.651	-39.290	14.564	1.00	27.80	C
ATOM	3172	N	GLN	A	421	16.314	-36.991	16.999	1.00	35.89	N
ATOM	3173	CA	GLN	A	421	16.633	-35.582	17.204	1.00	25.77	C
ATOM	3174	C	GLN	A	421	17.297	-35.315	18.543	1.00	31.19	C
ATOM	3175	O	GLN	A	421	17.222	-34.205	19.089	1.00	31.62	O
ATOM	3176	CB	GLN	A	421	15.396	-34.717	16.991	1.00	31.03	C
ATOM	3177	CG	GLN	A	421	14.917	-34.821	15.551	1.00	39.90	C
ATOM	3178	CD	GLN	A	421	13.504	-34.331	15.343	1.00	44.93	C
ATOM	3179	OE1	GLN	A	421	12.762	-34.884	14.527	1.00	45.53	O
ATOM	3180	NE2	GLN	A	421	13.124	-33.284	16.070	1.00	42.40	N

ATOM	3181	N	GLY	A	422	17.951	-36.347	19.069	1.00	33.24	N
ATOM	3182	CA	GLY	A	422	18.866	-36.157	20.177	1.00	39.92	C
ATOM	3183	C	GLY	A	422	18.486	-36.726	21.527	1.00	38.93	C
ATOM	3184	O	GLY	A	422	19.282	-36.663	22.453	1.00	37.16	O
ATOM	3185	N	ALA	A	423	17.286	-37.278	21.656	1.00	35.51	N
ATOM	3186	CA	ALA	A	423	16.861	-37.795	22.949	1.00	32.75	C
ATOM	3187	C	ALA	A	423	17.227	-39.258	23.080	1.00	28.54	C
ATOM	3188	O	ALA	A	423	17.340	-39.958	22.082	1.00	32.18	O
ATOM	3189	CB	ALA	A	423	15.365	-37.609	23.132	1.00	32.19	C
ATOM	3190	N	ARG	A	424	17.411	-39.715	24.317	1.00	28.73	N
ATOM	3191	CA	ARG	A	424	17.542	-41.139	24.586	1.00	29.17	C
ATOM	3192	C	ARG	A	424	16.173	-41.682	24.968	1.00	29.84	C
ATOM	3193	O	ARG	A	424	15.577	-41.256	25.960	1.00	35.05	O
ATOM	3194	CB	ARG	A	424	18.551	-41.421	25.707	1.00	28.38	C
ATOM	3195	CG	ARG	A	424	18.873	-42.912	25.845	1.00	32.89	C
ATOM	3196	CD	ARG	A	424	20.250	-43.163	26.457	1.00	38.46	C
ATOM	3197	NE	ARG	A	424	20.187	-43.229	27.919	1.00	57.58	N
ATOM	3198	CZ	ARG	A	424	20.693	-42.316	28.744	1.00	50.14	C
ATOM	3199	NH1	ARG	A	424	21.319	-41.249	28.261	1.00	56.71	N
ATOM	3200	NH2	ARG	A	424	20.578	-42.475	30.056	1.00	46.64	N
ATOM	3201	N	VAL	A	425	15.680	-42.625	24.176	1.00	26.01	N
ATOM	3202	CA	VAL	A	425	14.344	-43.151	24.358	1.00	22.30	C
ATOM	3203	C	VAL	A	425	14.362	-44.624	24.769	1.00	30.22	C
ATOM	3204	O	VAL	A	425	15.113	-45.422	24.212	1.00	29.02	O
ATOM	3205	CB	VAL	A	425	13.535	-42.994	23.054	1.00	25.52	C
ATOM	3206	CG1	VAL	A	425	12.112	-43.527	23.225	1.00	19.82	C
ATOM	3207	CG2	VAL	A	425	13.540	-41.528	22.596	1.00	19.93	C
ATOM	3208	N	TYR	A	426	13.540	-44.982	25.752	1.00	24.33	N
ATOM	3209	CA	TYR	A	426	13.262	-46.388	26.030	1.00	25.70	C
ATOM	3210	C	TYR	A	426	11.791	-46.699	25.747	1.00	26.68	C
ATOM	3211	O	TYR	A	426	10.903	-45.916	26.104	1.00	27.55	O
ATOM	3212	CB	TYR	A	426	13.617	-46.738	27.478	1.00	24.33	C
ATOM	3213	CG	TYR	A	426	15.073	-46.513	27.786	1.00	28.82	C
ATOM	3214	CD1	TYR	A	426	16.006	-47.538	27.633	1.00	26.87	C
ATOM	3215	CD2	TYR	A	426	15.525	-45.266	28.212	1.00	23.25	C
ATOM	3216	CE1	TYR	A	426	17.350	-47.326	27.907	1.00	27.11	C
ATOM	3217	CE2	TYR	A	426	16.857	-45.048	28.487	1.00	26.98	C
ATOM	3218	CZ	TYR	A	426	17.766	-46.076	28.332	1.00	28.04	C
ATOM	3219	OH	TYR	A	426	19.092	-45.842	28.610	1.00	29.63	O
ATOM	3220	N	ALA	A	427	11.539	-47.843	25.117	1.00	23.25	N
ATOM	3221	CA	ALA	A	427	10.184	-48.237	24.737	1.00	22.32	C
ATOM	3222	C	ALA	A	427	9.814	-49.614	25.283	1.00	26.38	C
ATOM	3223	O	ALA	A	427	10.665	-50.503	25.372	1.00	28.09	O
ATOM	3224	CB	ALA	A	427	10.037	-48.216	23.209	1.00	23.11	C

```
ATOM   3225  N   TYR A 428       8.543 -49.797  25.630  1.00 19.79           N
ATOM   3226  CA  TYR A 428       8.066 -51.081  26.138  1.00 23.49           C
ATOM   3227  C   TYR A 428       6.696 -51.375  25.560  1.00 23.32           C
ATOM   3228  O   TYR A 428       5.969 -50.460  25.171  1.00 23.74           O
ATOM   3229  CB  TYR A 428       7.949 -51.078  27.687  1.00 19.45           C
ATOM   3230  CG  TYR A 428       6.884 -50.118  28.201  1.00 21.92           C
ATOM   3231  CD1 TYR A 428       5.538 -50.489  28.263  1.00 20.04           C
ATOM   3232  CD2 TYR A 428       7.222 -48.836  28.607  1.00 19.87           C
ATOM   3233  CE1 TYR A 428       4.563 -49.602  28.708  1.00 20.35           C
ATOM   3234  CE2 TYR A 428       6.256 -47.942  29.058  1.00 20.99           C
ATOM   3235  CZ  TYR A 428       4.930 -48.327  29.102  1.00 25.04           C
ATOM   3236  OH  TYR A 428       3.977 -47.424  29.540  1.00 24.46           O
ATOM   3237  N   VAL A 429       6.341 -52.652  25.533  1.00 22.88           N
ATOM   3238  CA  VAL A 429       4.954 -53.060  25.393  1.00 23.42           C
ATOM   3239  C   VAL A 429       4.617 -53.885  26.611  1.00 22.55           C
ATOM   3240  O   VAL A 429       5.364 -54.789  26.987  1.00 24.57           O
ATOM   3241  CB  VAL A 429       4.701 -53.905  24.126  1.00 25.90           C
ATOM   3242  CG1 VAL A 429       3.231 -54.269  24.035  1.00 24.83           C
ATOM   3243  CG2 VAL A 429       5.107 -53.125  22.888  1.00 26.71           C
ATOM   3244  N   PHE A 430       3.498 -53.561  27.239  1.00 26.81           N
ATOM   3245  CA  PHE A 430       3.118 -54.204  28.485  1.00 25.15           C
ATOM   3246  C   PHE A 430       2.136 -55.319  28.150  1.00 24.50           C
ATOM   3247  O   PHE A 430       1.063 -55.071  27.614  1.00 25.87           O
ATOM   3248  CB  PHE A 430       2.518 -53.164  29.426  1.00 20.68           C
ATOM   3249  CG  PHE A 430       2.097 -53.710  30.760  1.00 25.71           C
ATOM   3250  CD1 PHE A 430       2.961 -53.671  31.841  1.00 26.20           C
ATOM   3251  CD2 PHE A 430       0.829 -54.239  30.940  1.00 25.24           C
ATOM   3252  CE1 PHE A 430       2.568 -54.161  33.075  1.00 28.16           C
ATOM   3253  CE2 PHE A 430       0.434 -54.735  32.170  1.00 23.94           C
ATOM   3254  CZ  PHE A 430       1.304 -54.693  33.235  1.00 26.33           C
ATOM   3255  N   GLU A 431       2.515 -56.551  28.465  1.00 27.95           N
ATOM   3256  CA  GLU A 431       1.838 -57.715  27.899  1.00 30.59           C
ATOM   3257  C   GLU A 431       1.282 -58.664  28.943  1.00 33.80           C
ATOM   3258  O   GLU A 431       1.007 -59.825  28.644  1.00 34.48           O
ATOM   3259  CB  GLU A 431       2.790 -58.486  26.978  1.00 25.93           C
ATOM   3260  CG  GLU A 431       3.159 -57.743  25.707  1.00 25.92           C
ATOM   3261  CD  GLU A 431       3.789 -58.652  24.659  1.00 35.29           C
ATOM   3262  OE1 GLU A 431       3.304 -58.659  23.514  1.00 47.00           O
ATOM   3263  OE2 GLU A 431       4.765 -59.362  24.976  1.00 48.81           O
ATOM   3264  N   HIS A 432       1.131 -58.183  30.169  1.00 25.24           N
ATOM   3265  CA  HIS A 432       0.569 -59.020  31.207  1.00 29.29           C
ATOM   3266  C   HIS A 432      -0.885 -58.670  31.482  1.00 30.75           C
ATOM   3267  O   HIS A 432      -1.215 -57.515  31.748  1.00 29.88           O
ATOM   3268  CB  HIS A 432       1.374 -58.934  32.499  1.00 27.69           C
```

ATOM	3269	CG	HIS	A	432	0.780	-59.739	33.607	1.00	37.42	C
ATOM	3270	ND1	HIS	A	432	0.824	-61.117	33.626	1.00	42.63	N
ATOM	3271	CD2	HIS	A	432	0.078	-59.367	34.705	1.00	30.57	C
ATOM	3272	CE1	HIS	A	432	0.201	-61.557	34.706	1.00	41.80	C
ATOM	3273	NE2	HIS	A	432	-0.269	-60.515	35.371	1.00	33.49	N
ATOM	3274	N	ARG	A	433	-1.753	-59.673	31.400	1.00	29.51	N
ATOM	3275	CA	ARG	A	433	-3.156	-59.496	31.744	1.00	33.23	C
ATOM	3276	C	ARG	A	433	-3.329	-59.813	33.224	1.00	30.15	C
ATOM	3277	O	ARG	A	433	-3.005	-60.917	33.668	1.00	32.68	O
ATOM	3278	CB	ARG	A	433	-4.044	-60.411	30.890	1.00	31.06	C
ATOM	3279	CG	ARG	A	433	-5.525	-60.134	31.052	1.00	35.10	C
ATOM	3280	CD	ARG	A	433	-6.382	-61.102	30.249	1.00	32.15	C
ATOM	3281	NE	ARG	A	433	-7.730	-61.217	30.812	1.00	28.67	N
ATOM	3282	CZ	ARG	A	433	-8.790	-60.572	30.338	1.00	35.27	C
ATOM	3283	NH1	ARG	A	433	-8.661	-59.770	29.279	1.00	29.47	N
ATOM	3284	NH2	ARG	A	433	-9.979	-60.733	30.914	1.00	28.71	N
ATOM	3285	N	ALA	A	434	-3.811	-58.839	33.989	1.00	31.61	N
ATOM	3286	CA	ALA	A	434	-3.958	-58.996	35.436	1.00	33.89	C
ATOM	3287	C	ALA	A	434	-4.873	-60.167	35.762	1.00	38.84	C
ATOM	3288	O	ALA	A	434	-5.935	-60.322	35.147	1.00	33.93	O
ATOM	3289	CB	ALA	A	434	-4.506	-57.720	36.057	1.00	32.59	C
ATOM	3290	N	SER	A	435	-4.466	-60.987	36.729	1.00	30.25	N
ATOM	3291	CA	SER	A	435	-5.301	-62.104	37.174	1.00	31.51	C
ATOM	3292	C	SER	A	435	-6.645	-61.600	37.687	1.00	35.89	C
ATOM	3293	O	SER	A	435	-7.644	-62.313	37.652	1.00	35.40	O
ATOM	3294	CB	SER	A	435	-4.596	-62.909	38.266	1.00	35.53	C
ATOM	3295	OG	SER	A	435	-4.572	-62.189	39.486	1.00	44.45	O
ATOM	3296	N	THR	A	436	-6.663	-60.353	38.142	1.00	37.53	N
ATOM	3297	CA	THR	A	436	-7.854	-59.764	38.736	1.00	36.39	C
ATOM	3298	C	THR	A	436	-8.744	-59.061	37.719	1.00	35.08	C
ATOM	3299	O	THR	A	436	-9.791	-58.520	38.073	1.00	38.96	O
ATOM	3300	CB	THR	A	436	-7.468	-58.747	39.824	1.00	37.13	C
ATOM	3301	OG1	THR	A	436	-6.523	-57.806	39.287	1.00	36.28	O
ATOM	3302	CG2	THR	A	436	-6.844	-59.464	41.017	1.00	41.75	C
ATOM	3303	N	LEU	A	437	-8.329	-59.052	36.458	1.00	32.01	N
ATOM	3304	CA	LEU	A	437	-9.047	-58.275	35.452	1.00	31.37	C
ATOM	3305	C	LEU	A	437	-10.445	-58.835	35.207	1.00	34.16	C
ATOM	3306	O	LEU	A	437	-10.623	-60.044	35.042	1.00	37.61	O
ATOM	3307	CB	LEU	A	437	-8.248	-58.201	34.148	1.00	34.59	C
ATOM	3308	CG	LEU	A	437	-8.800	-57.277	33.058	1.00	36.68	C
ATOM	3309	CD1	LEU	A	437	-8.745	-55.841	33.511	1.00	29.03	C
ATOM	3310	CD2	LEU	A	437	-8.037	-57.463	31.746	1.00	28.90	C
ATOM	3311	N	SER	A	438	-11.434	-57.949	35.180	1.00	32.38	N
ATOM	3312	CA	SER	A	438	-12.823	-58.365	35.067	1.00	33.09	C

ATOM	3313	C	SER	A	438	-13.446	-58.062	33.707	1.00	37.59	C
ATOM	3314	O	SER	A	438	-14.601	-58.415	33.465	1.00	38.50	O
ATOM	3315	CB	SER	A	438	-13.655	-57.702	36.158	1.00	34.90	C
ATOM	3316	OG	SER	A	438	-13.671	-56.302	35.964	1.00	43.42	O
ATOM	3317	N	TRP	A	439	-12.704	-57.384	32.832	1.00	31.13	N
ATOM	3318	CA	TRP	A	439	-13.107	-57.283	31.434	1.00	29.80	C
ATOM	3319	C	TRP	A	439	-12.856	-58.642	30.772	1.00	28.87	C
ATOM	3320	O	TRP	A	439	-11.956	-59.379	31.196	1.00	28.19	O
ATOM	3321	CB	TRP	A	439	-12.341	-56.162	30.707	1.00	31.64	C
ATOM	3322	CG	TRP	A	439	-12.640	-54.799	31.252	1.00	26.49	C
ATOM	3323	CD1	TRP	A	439	-11.927	-54.120	32.202	1.00	23.10	C
ATOM	3324	CD2	TRP	A	439	-13.742	-53.956	30.901	1.00	22.21	C
ATOM	3325	NE1	TRP	A	439	-12.518	-52.908	32.458	1.00	23.88	N
ATOM	3326	CE2	TRP	A	439	-13.633	-52.781	31.674	1.00	24.58	C
ATOM	3327	CE3	TRP	A	439	-14.814	-54.083	30.022	1.00	23.78	C
ATOM	3328	CZ2	TRP	A	439	-14.553	-51.737	31.582	1.00	23.44	C
ATOM	3329	CZ3	TRP	A	439	-15.725	-53.042	29.927	1.00	24.91	C
ATOM	3330	CH2	TRP	A	439	-15.587	-51.886	30.700	1.00	25.50	C
ATOM	3331	N	PRO	A	440	-13.643	-58.977	29.730	1.00	26.32	N
ATOM	3332	CA	PRO	A	440	-13.551	-60.299	29.102	1.00	27.99	C
ATOM	3333	C	PRO	A	440	-12.177	-60.591	28.511	1.00	30.91	C
ATOM	3334	O	PRO	A	440	-11.389	-59.682	28.278	1.00	31.87	O
ATOM	3335	CB	PRO	A	440	-14.594	-60.227	27.977	1.00	29.76	C
ATOM	3336	CG	PRO	A	440	-14.756	-58.785	27.694	1.00	28.39	C
ATOM	3337	CD	PRO	A	440	-14.604	-58.109	29.027	1.00	28.82	C
ATOM	3338	N	LEU	A	441	-11.913	-61.866	28.263	1.00	33.53	N
ATOM	3339	CA	LEU	A	441	-10.656	-62.308	27.674	1.00	34.83	C
ATOM	3340	C	LEU	A	441	-10.390	-61.731	26.274	1.00	34.71	C
ATOM	3341	O	LEU	A	441	-9.232	-61.508	25.904	1.00	34.83	O
ATOM	3342	CB	LEU	A	441	-10.624	-63.836	27.625	1.00	39.37	C
ATOM	3343	CG	LEU	A	441	-9.255	-64.491	27.798	1.00	49.18	C
ATOM	3344	CD1	LEU	A	441	-8.790	-64.386	29.245	1.00	41.01	C
ATOM	3345	CD2	LEU	A	441	-9.299	-65.943	27.351	1.00	51.15	C
ATOM	3346	N	TRP	A	442	-11.441	-61.489	25.490	1.00	27.33	N
ATOM	3347	CA	TRP	A	442	-11.222	-61.007	24.125	1.00	29.30	C
ATOM	3348	C	TRP	A	442	-10.586	-59.619	24.090	1.00	31.09	C
ATOM	3349	O	TRP	A	442	-10.003	-59.219	23.081	1.00	30.58	O
ATOM	3350	CB	TRP	A	442	-12.493	-61.062	23.251	1.00	27.33	C
ATOM	3351	CG	TRP	A	442	-13.592	-60.077	23.595	1.00	26.60	C
ATOM	3352	CD1	TRP	A	442	-14.735	-60.338	24.295	1.00	28.60	C
ATOM	3353	CD2	TRP	A	442	-13.661	-58.691	23.222	1.00	26.83	C
ATOM	3354	NE1	TRP	A	442	-15.505	-59.202	24.392	1.00	26.49	N
ATOM	3355	CE2	TRP	A	442	-14.868	-58.178	23.743	1.00	23.68	C
ATOM	3356	CE3	TRP	A	442	-12.821	-57.835	22.496	1.00	26.62	C

ATOM	3357	CZ2	TRP	A	442	-15.249	-56.845	23.572	1.00	23.08	C
ATOM	3358	CZ3	TRP	A	442	-13.204	-56.515	22.326	1.00	23.66	C
ATOM	3359	CH2	TRP	A	442	-14.407	-56.032	22.864	1.00	25.81	C
ATOM	3360	N	MET	A	443	-10.689	-58.886	25.192	1.00	34.31	N
ATOM	3361	CA	MET	A	443	-10.089	-57.563	25.250	1.00	27.89	C
ATOM	3362	C	MET	A	443	-8.570	-57.610	25.422	1.00	27.47	C
ATOM	3363	O	MET	A	443	-7.892	-56.622	25.161	1.00	28.28	O
ATOM	3364	CB	MET	A	443	-10.746	-56.710	26.324	1.00	24.93	C
ATOM	3365	CG	MET	A	443	-12.050	-56.066	25.877	1.00	29.79	C
ATOM	3366	SD	MET	A	443	-12.603	-54.899	27.134	1.00	33.82	S
ATOM	3367	CE	MET	A	443	-14.163	-54.333	26.495	1.00	22.84	C
ATOM	3368	N	GLY	A	444	-8.038	-58.763	25.821	1.00	25.08	N
ATOM	3369	CA	GLY	A	444	-6.607	-58.912	26.019	1.00	22.89	C
ATOM	3370	C	GLY	A	444	-6.109	-58.037	27.163	1.00	31.72	C
ATOM	3371	O	GLY	A	444	-6.690	-58.046	28.253	1.00	28.49	O
ATOM	3372	N	VAL	A	445	-5.032	-57.289	26.923	1.00	27.33	N
ATOM	3373	CA	VAL	A	445	-4.538	-56.317	27.904	1.00	28.59	C
ATOM	3374	C	VAL	A	445	-5.016	-54.902	27.536	1.00	24.00	C
ATOM	3375	O	VAL	A	445	-4.408	-54.235	26.705	1.00	25.21	O
ATOM	3376	CB	VAL	A	445	-2.994	-56.323	27.987	1.00	26.29	C
ATOM	3377	CG1	VAL	A	445	-2.520	-55.452	29.145	1.00	24.82	C
ATOM	3378	CG2	VAL	A	445	-2.460	-57.746	28.120	1.00	21.36	C
ATOM	3379	N	PRO	A	446	-6.107	-54.436	28.158	1.00	24.45	N
ATOM	3380	CA	PRO	A	446	-6.711	-53.181	27.696	1.00	28.19	C
ATOM	3381	C	PRO	A	446	-6.008	-51.928	28.209	1.00	24.69	C
ATOM	3382	O	PRO	A	446	-5.273	-51.970	29.196	1.00	22.78	O
ATOM	3383	CB	PRO	A	446	-8.143	-53.238	28.265	1.00	22.28	C
ATOM	3384	CG	PRO	A	446	-8.317	-54.654	28.782	1.00	26.81	C
ATOM	3385	CD	PRO	A	446	-6.934	-55.088	29.182	1.00	22.91	C
ATOM	3386	N	HIS	A	447	-6.254	-50.831	27.499	1.00	22.67	N
ATOM	3387	CA	HIS	A	447	-5.836	-49.479	27.849	1.00	25.93	C
ATOM	3388	C	HIS	A	447	-6.039	-49.227	29.338	1.00	24.45	C
ATOM	3389	O	HIS	A	447	-7.115	-49.484	29.866	1.00	25.45	O
ATOM	3390	CB	HIS	A	447	-6.711	-48.524	27.046	1.00	18.26	C
ATOM	3391	CG	HIS	A	447	-6.275	-47.093	27.072	1.00	23.82	C
ATOM	3392	ND1	HIS	A	447	-5.134	-46.649	26.439	1.00	22.30	N
ATOM	3393	CD2	HIS	A	447	-6.871	-45.992	27.590	1.00	21.83	C
ATOM	3394	CE1	HIS	A	447	-5.034	-45.339	26.585	1.00	17.15	C
ATOM	3395	NE2	HIS	A	447	-6.075	-44.917	27.280	1.00	23.47	N
ATOM	3396	N	GLY	A	448	-5.001	-48.751	30.017	1.00	24.01	N
ATOM	3397	CA	GLY	A	448	-5.108	-48.415	31.431	1.00	21.59	C
ATOM	3398	C	GLY	A	448	-4.660	-49.484	32.420	1.00	25.57	C
ATOM	3399	O	GLY	A	448	-4.444	-49.180	33.585	1.00	26.49	O
ATOM	3400	N	TYR	A	449	-4.491	-50.728	31.977	1.00	23.96	N

ATOM	3401	CA	TYR	A	449	-4.279	-51.813	32.937	1.00	22.61	C
ATOM	3402	C	TYR	A	449	-2.835	-52.187	33.254	1.00	24.00	C
ATOM	3403	O	TYR	A	449	-2.586	-53.223	33.863	1.00	29.24	O
ATOM	3404	CB	TYR	A	449	-5.162	-53.033	32.623	1.00	19.78	C
ATOM	3405	CG	TYR	A	449	-6.584	-52.725	33.006	1.00	25.70	C
ATOM	3406	CD1	TYR	A	449	-6.970	-52.744	34.338	1.00	21.66	C
ATOM	3407	CD2	TYR	A	449	-7.527	-52.351	32.050	1.00	22.02	C
ATOM	3408	CE1	TYR	A	449	-8.256	-52.429	34.713	1.00	23.90	C
ATOM	3409	CE2	TYR	A	449	-8.821	-52.031	32.419	1.00	22.58	C
ATOM	3410	CZ	TYR	A	449	-9.177	-52.069	33.757	1.00	25.36	C
ATOM	3411	OH	TYR	A	449	-10.453	-51.748	34.153	1.00	24.77	O
ATOM	3412	N	GLU	A	450	-1.896	-51.325	32.872	1.00	25.52	N
ATOM	3413	CA	GLU	A	450	-0.521	-51.426	33.365	1.00	24.39	C
ATOM	3414	C	GLU	A	450	-0.306	-50.550	34.607	1.00	24.17	C
ATOM	3415	O	GLU	A	450	0.578	-50.823	35.409	1.00	25.41	O
ATOM	3416	CB	GLU	A	450	0.483	-51.024	32.277	1.00	22.35	C
ATOM	3417	CG	GLU	A	450	0.839	-49.552	32.246	1.00	14.42	C
ATOM	3418	CD	GLU	A	450	-0.260	-48.671	31.693	1.00	23.59	C
ATOM	3419	OE1	GLU	A	450	-1.420	-49.132	31.569	1.00	25.15	O
ATOM	3420	OE2	GLU	A	450	0.041	-47.502	31.366	1.00	24.11	O
ATOM	3421	N	ILE	A	451	-1.120	-49.502	34.751	1.00	23.20	N
ATOM	3422	CA	ILE	A	451	-0.949	-48.513	35.809	1.00	24.91	C
ATOM	3423	C	ILE	A	451	-0.863	-49.120	37.219	1.00	28.74	C
ATOM	3424	O	ILE	A	451	0.044	-48.784	37.982	1.00	27.00	O
ATOM	3425	CB	ILE	A	451	-2.076	-47.455	35.776	1.00	26.35	C
ATOM	3426	CG1	ILE	A	451	-2.164	-46.819	34.389	1.00	19.96	C
ATOM	3427	CG2	ILE	A	451	-1.853	-46.375	36.848	1.00	19.76	C
ATOM	3428	CD1	ILE	A	451	-3.311	-45.835	34.235	1.00	16.46	C
ATOM	3429	N	GLU	A	452	-1.790	-50.015	37.554	1.00	24.77	N
ATOM	3430	CA	GLU	A	452	-1.818	-50.631	38.881	1.00	27.00	C
ATOM	3431	C	GLU	A	452	-0.510	-51.364	39.197	1.00	28.81	C
ATOM	3432	O	GLU	A	452	-0.100	-51.452	40.360	1.00	27.33	O
ATOM	3433	CB	GLU	A	452	-3.022	-51.575	39.031	1.00	25.32	C
ATOM	3434	CG	GLU	A	452	-3.048	-52.720	38.010	1.00	30.83	C
ATOM	3435	CD	GLU	A	452	-4.454	-53.257	37.784	1.00	35.75	C
ATOM	3436	OE1	GLU	A	452	-4.697	-54.444	38.089	1.00	37.28	O
ATOM	3437	OE2	GLU	A	452	-5.322	-52.490	37.309	1.00	36.52	O
ATOM	3438	N	PHE	A	453	0.149	-51.866	38.158	1.00	24.75	N
ATOM	3439	CA	PHE	A	453	1.438	-52.526	38.329	1.00	26.89	C
ATOM	3440	C	PHE	A	453	2.587	-51.549	38.550	1.00	28.55	C
ATOM	3441	O	PHE	A	453	3.462	-51.797	39.386	1.00	28.27	O
ATOM	3442	CB	PHE	A	453	1.716	-53.472	37.157	1.00	24.87	C
ATOM	3443	CG	PHE	A	453	0.864	-54.696	37.190	1.00	28.57	C
ATOM	3444	CD1	PHE	A	453	-0.363	-54.717	36.547	1.00	28.16	C

ATOM	3445	CD2	PHE	A	453	1.253	-55.802	37.930	1.00	26.18	C
ATOM	3446	CE1	PHE	A	453	-1.175	-55.840	36.611	1.00	35.94	C
ATOM	3447	CE2	PHE	A	453	0.444	-56.931	37.999	1.00	31.42	C
ATOM	3448	CZ	PHE	A	453	-0.771	-56.948	37.341	1.00	30.45	C
ATOM	3449	N	ILE	A	454	2.579	-50.445	37.808	1.00	19.99	N
ATOM	3450	CA	ILE	A	454	3.572	-49.399	37.986	1.00	23.42	C
ATOM	3451	C	ILE	A	454	3.478	-48.841	39.418	1.00	27.75	C
ATOM	3452	O	ILE	A	454	4.499	-48.580	40.052	1.00	25.18	O
ATOM	3453	CB	ILE	A	454	3.383	-48.247	36.958	1.00	29.93	C
ATOM	3454	CG1	ILE	A	454	3.342	-48.783	35.515	1.00	23.48	C
ATOM	3455	CG2	ILE	A	454	4.454	-47.163	37.133	1.00	24.96	C
ATOM	3456	CD1	ILE	A	454	4.511	-49.665	35.141	1.00	25.38	C
ATOM	3457	N	PHE	A	455	2.257	-48.685	39.932	1.00	24.15	N
ATOM	3458	CA	PHE	A	455	2.062	-48.144	41.283	1.00	24.82	C
ATOM	3459	C	PHE	A	455	2.202	-49.187	42.379	1.00	27.72	C
ATOM	3460	O	PHE	A	455	2.072	-48.870	43.549	1.00	27.40	O
ATOM	3461	CB	PHE	A	455	0.709	-47.440	41.418	1.00	20.92	C
ATOM	3462	CG	PHE	A	455	0.715	-46.023	40.912	1.00	29.29	C
ATOM	3463	CD1	PHE	A	455	0.568	-45.752	39.560	1.00	20.10	C
ATOM	3464	CD2	PHE	A	455	0.892	-44.963	41.786	1.00	25.52	C
ATOM	3465	CE1	PHE	A	455	0.581	-44.446	39.087	1.00	22.52	C
ATOM	3466	CE2	PHE	A	455	0.905	-43.656	41.321	1.00	26.99	C
ATOM	3467	CZ	PHE	A	455	0.741	-43.395	39.969	1.00	24.45	C
ATOM	3468	N	GLY	A	456	2.455	-50.433	41.999	1.00	30.51	N
ATOM	3469	CA	GLY	A	456	2.703	-51.471	42.979	1.00	29.98	C
ATOM	3470	C	GLY	A	456	1.472	-51.850	43.783	1.00	29.41	C
ATOM	3471	O	GLY	A	456	1.590	-52.385	44.880	1.00	30.16	O
ATOM	3472	N	ILE	A	457	0.294	-51.569	43.234	1.00	29.62	N
ATOM	3473	CA	ILE	A	457	-0.970	-51.995	43.833	1.00	28.84	C
ATOM	3474	C	ILE	A	457	-1.044	-53.505	44.162	1.00	33.09	C
ATOM	3475	O	ILE	A	457	-1.663	-53.887	45.155	1.00	31.64	O
ATOM	3476	CB	ILE	A	457	-2.179	-51.584	42.956	1.00	30.31	C
ATOM	3477	CG1	ILE	A	457	-2.151	-50.080	42.675	1.00	28.30	C
ATOM	3478	CG2	ILE	A	457	-3.488	-51.977	43.617	1.00	27.50	C
ATOM	3479	CD1	ILE	A	457	-2.206	-49.211	43.930	1.00	23.56	C
ATOM	3480	N	PRO	A	458	-0.416	-54.372	43.340	1.00	34.06	N
ATOM	3481	CA	PRO	A	458	-0.442	-55.780	43.763	1.00	31.55	C
ATOM	3482	C	PRO	A	458	0.147	-56.067	45.147	1.00	35.51	C
ATOM	3483	O	PRO	A	458	-0.169	-57.113	45.710	1.00	37.58	O
ATOM	3484	CB	PRO	A	458	0.373	-56.481	42.675	1.00	29.43	C
ATOM	3485	CG	PRO	A	458	0.050	-55.688	41.441	1.00	30.52	C
ATOM	3486	CD	PRO	A	458	-0.026	-54.248	41.919	1.00	32.10	C
ATOM	3487	N	LEU	A	459	0.967	-55.170	45.687	1.00	32.98	N
ATOM	3488	CA	LEU	A	459	1.537	-55.380	47.019	1.00	34.67	C

ATOM	3489	C	LEU	A	459	0.494	-55.250	48.129	1.00	34.41	C
ATOM	3490	O	LEU	A	459	0.733	-55.662	49.260	1.00	36.84	O
ATOM	3491	CB	LEU	A	459	2.710	-54.428	47.279	1.00	33.18	C
ATOM	3492	CG	LEU	A	459	3.931	-54.671	46.389	1.00	41.36	C
ATOM	3493	CD1	LEU	A	459	5.082	-53.757	46.783	1.00	35.73	C
ATOM	3494	CD2	LEU	A	459	4.348	-56.130	46.462	1.00	40.34	C
ATOM	3495	N	ASP	A	460	-0.659	-54.677	47.800	1.00	34.30	N
ATOM	3496	CA	ASP	A	460	-1.735	-54.521	48.765	1.00	35.20	C
ATOM	3497	C	ASP	A	460	-2.271	-55.903	49.150	1.00	46.66	C
ATOM	3498	O	ASP	A	460	-2.867	-56.594	48.326	1.00	36.09	O
ATOM	3499	CB	ASP	A	460	-2.846	-53.644	48.188	1.00	32.48	C
ATOM	3500	CG	ASP	A	460	-3.900	-53.270	49.220	1.00	44.35	C
ATOM	3501	OD1	ASP	A	460	-3.980	-53.937	50.277	1.00	46.90	O
ATOM	3502	OD2	ASP	A	460	-4.658	-52.312	48.969	1.00	44.80	O
ATOM	3503	N	PRO	A	461	-2.065	-56.301	50.419	1.00	48.95	N
ATOM	3504	CA	PRO	A	461	-2.403	-57.646	50.906	1.00	49.71	C
ATOM	3505	C	PRO	A	461	-3.895	-57.947	50.793	1.00	49.28	C
ATOM	3506	O	PRO	A	461	-4.278	-59.100	50.585	1.00	53.09	O
ATOM	3507	CB	PRO	A	461	-1.985	-57.605	52.381	1.00	48.53	C
ATOM	3508	CG	PRO	A	461	-1.001	-56.489	52.470	1.00	55.07	C
ATOM	3509	CD	PRO	A	461	-1.473	-55.466	51.476	1.00	40.71	C
ATOM	3510	N	SER	A	462	-4.721	-56.914	50.911	1.00	39.53	N
ATOM	3511	CA	SER	A	462	-6.165	-57.080	50.838	1.00	43.50	C
ATOM	3512	C	SER	A	462	-6.692	-57.187	49.407	1.00	49.18	C
ATOM	3513	O	SER	A	462	-7.895	-57.089	49.181	1.00	48.60	O
ATOM	3514	CB	SER	A	462	-6.861	-55.921	51.544	1.00	50.23	C
ATOM	3515	OG	SER	A	462	-6.816	-54.757	50.744	1.00	50.68	O
ATOM	3516	N	ARG	A	463	-5.802	-57.375	48.440	1.00	47.02	N
ATOM	3517	CA	ARG	A	463	-6.235	-57.611	47.065	1.00	43.66	C
ATOM	3518	C	ARG	A	463	-5.742	-58.979	46.587	1.00	43.96	C
ATOM	3519	O	ARG	A	463	-4.924	-59.611	47.253	1.00	49.62	O
ATOM	3520	CB	ARG	A	463	-5.775	-56.487	46.137	1.00	44.77	C
ATOM	3521	CG	ARG	A	463	-6.397	-55.140	46.454	1.00	40.49	C
ATOM	3522	CD	ARG	A	463	-5.929	-54.074	45.474	1.00	38.83	C
ATOM	3523	NE	ARG	A	463	-6.307	-52.735	45.915	1.00	46.88	N
ATOM	3524	CZ	ARG	A	463	-7.382	-52.082	45.486	1.00	42.63	C
ATOM	3525	NH1	ARG	A	463	-8.188	-52.637	44.593	1.00	52.05	N
ATOM	3526	NH2	ARG	A	463	-7.649	-50.870	45.949	1.00	47.43	N
ATOM	3527	N	ASN	A	464	-6.233	-59.430	45.438	1.00	43.08	N
ATOM	3528	CA	ASN	A	464	-6.145	-60.850	45.102	1.00	50.05	C
ATOM	3529	C	ASN	A	464	-5.069	-61.223	44.091	1.00	47.44	C
ATOM	3530	O	ASN	A	464	-5.183	-62.244	43.406	1.00	50.70	O
ATOM	3531	CB	ASN	A	464	-7.512	-61.378	44.635	1.00	44.69	C
ATOM	3532	CG	ASN	A	464	-8.585	-61.250	45.705	1.00	57.54	C

```
ATOM   3533  OD1 ASN A 464      -8.280 -61.078  46.889  1.00 63.53           O
ATOM   3534  ND2 ASN A 464      -9.848 -61.340  45.295  1.00 62.13           N
ATOM   3535  N   TYR A 465      -4.019 -60.414  44.004  1.00 42.27           N
ATOM   3536  CA  TYR A 465      -2.960 -60.666  43.031  1.00 39.46           C
ATOM   3537  C   TYR A 465      -2.122 -61.876  43.447  1.00 35.90           C
ATOM   3538  O   TYR A 465      -2.090 -62.232  44.612  1.00 39.41           O
ATOM   3539  CB  TYR A 465      -2.072 -59.430  42.880  1.00 32.20           C
ATOM   3540  CG  TYR A 465      -2.747 -58.241  42.226  1.00 30.93           C
ATOM   3541  CD1 TYR A 465      -3.422 -57.295  42.984  1.00 26.64           C
ATOM   3542  CD2 TYR A 465      -2.693 -58.061  40.844  1.00 27.68           C
ATOM   3543  CE1 TYR A 465      -4.021 -56.204  42.392  1.00 29.55           C
ATOM   3544  CE2 TYR A 465      -3.294 -56.986  40.245  1.00 28.13           C
ATOM   3545  CZ  TYR A 465      -3.949 -56.053  41.018  1.00 31.24           C
ATOM   3546  OH  TYR A 465      -4.546 -54.976  40.406  1.00 26.63           O
ATOM   3547  N   THR A 466      -1.441 -62.497  42.493  1.00 37.96           N
ATOM   3548  CA  THR A 466      -0.609 -63.660  42.784  1.00 33.69           C
ATOM   3549  C   THR A 466       0.777 -63.244  43.240  1.00 35.24           C
ATOM   3550  O   THR A 466       1.176 -62.092  43.074  1.00 40.92           O
ATOM   3551  CB  THR A 466      -0.428 -64.551  41.537  1.00 37.32           C
ATOM   3552  OG1 THR A 466       0.375 -63.863  40.567  1.00 41.73           O
ATOM   3553  CG2 THR A 466      -1.773 -64.898  40.926  1.00 32.49           C
ATOM   3554  N   ALA A 467       1.513 -64.199  43.797  1.00 34.84           N
ATOM   3555  CA  ALA A 467       2.878 -63.966  44.249  1.00 41.85           C
ATOM   3556  C   ALA A 467       3.767 -63.480  43.107  1.00 38.26           C
ATOM   3557  O   ALA A 467       4.582 -62.578  43.292  1.00 39.48           O
ATOM   3558  CB  ALA A 467       3.456 -65.235  44.877  1.00 30.93           C
ATOM   3559  N   GLU A 468       3.601 -64.077  41.930  1.00 34.97           N
ATOM   3560  CA  GLU A 468       4.366 -63.674  40.755  1.00 42.81           C
ATOM   3561  C   GLU A 468       4.022 -62.246  40.346  1.00 38.69           C
ATOM   3562  O   GLU A 468       4.887 -61.491  39.895  1.00 32.63           O
ATOM   3563  CB  GLU A 468       4.093 -64.609  39.574  1.00 39.89           C
ATOM   3564  CG  GLU A 468       4.448 -66.066  39.804  1.00 53.65           C
ATOM   3565  CD  GLU A 468       4.070 -66.946  38.617  1.00 84.36           C
ATOM   3566  OE1 GLU A 468       3.056 -66.647  37.933  1.00 70.30           O
ATOM   3567  OE2 GLU A 468       4.792 -67.936  38.365  1.00 91.22           O
ATOM   3568  N   GLU A 469       2.750 -61.887  40.487  1.00 33.82           N
ATOM   3569  CA  GLU A 469       2.304 -60.558  40.119  1.00 31.00           C
ATOM   3570  C   GLU A 469       2.950 -59.510  41.024  1.00 41.01           C
ATOM   3571  O   GLU A 469       3.314 -58.416  40.563  1.00 31.83           O
ATOM   3572  CB  GLU A 469       0.775 -60.472  40.109  1.00 31.48           C
ATOM   3573  CG  GLU A 469       0.167 -61.024  38.810  1.00 34.20           C
ATOM   3574  CD  GLU A 469      -1.359 -61.027  38.799  1.00 37.65           C
ATOM   3575  OE1 GLU A 469      -1.969 -61.263  39.864  1.00 38.30           O
ATOM   3576  OE2 GLU A 469      -1.950 -60.804  37.719  1.00 36.38           O
```

```
ATOM   3577  N   LYS A 470       3.134 -59.861  42.296  1.00 35.28           N
ATOM   3578  CA  LYS A 470       3.796 -58.961  43.234  1.00 32.43           C
ATOM   3579  C   LYS A 470       5.246 -58.725  42.836  1.00 32.92           C
ATOM   3580  O   LYS A 470       5.716 -57.586  42.807  1.00 33.00           O
ATOM   3581  CB  LYS A 470       3.709 -59.492  44.669  1.00 32.31           C
ATOM   3582  CG  LYS A 470       2.290 -59.587  45.193  1.00 37.54           C
ATOM   3583  CD  LYS A 470       2.252 -60.150  46.608  1.00 46.37           C
ATOM   3584  CE  LYS A 470       0.824 -60.280  47.127  1.00 40.76           C
ATOM   3585  NZ  LYS A 470       0.792 -60.222  48.620  1.00 52.35           N
ATOM   3586  N   ILE A 471       5.955 -59.804  42.529  1.00 30.15           N
ATOM   3587  CA  ILE A 471       7.335 -59.694  42.067  1.00 33.02           C
ATOM   3588  C   ILE A 471       7.443 -58.811  40.810  1.00 31.76           C
ATOM   3589  O   ILE A 471       8.330 -57.959  40.721  1.00 31.86           O
ATOM   3590  CB  ILE A 471       7.949 -61.097  41.829  1.00 39.83           C
ATOM   3591  CG1 ILE A 471       8.188 -61.793  43.175  1.00 39.25           C
ATOM   3592  CG2 ILE A 471       9.248 -61.014  41.013  1.00 27.70           C
ATOM   3593  CD1 ILE A 471       8.386 -63.286  43.060  1.00 40.13           C
ATOM   3594  N   PHE A 472       6.520 -59.010  39.869  1.00 29.20           N
ATOM   3595  CA  PHE A 472       6.438 -58.234  38.628  1.00 32.71           C
ATOM   3596  C   PHE A 472       6.258 -56.744  38.916  1.00 30.00           C
ATOM   3597  O   PHE A 472       6.960 -55.898  38.350  1.00 26.98           O
ATOM   3598  CB  PHE A 472       5.278 -58.762  37.765  1.00 31.81           C
ATOM   3599  CG  PHE A 472       5.112 -58.063  36.431  1.00 33.80           C
ATOM   3600  CD1 PHE A 472       6.194 -57.878  35.576  1.00 31.63           C
ATOM   3601  CD2 PHE A 472       3.855 -57.635  36.013  1.00 29.77           C
ATOM   3602  CE1 PHE A 472       6.032 -57.249  34.342  1.00 30.14           C
ATOM   3603  CE2 PHE A 472       3.680 -57.003  34.776  1.00 25.32           C
ATOM   3604  CZ  PHE A 472       4.771 -56.816  33.938  1.00 25.93           C
ATOM   3605  N   ALA A 473       5.316 -56.426  39.798  1.00 27.67           N
ATOM   3606  CA  ALA A 473       5.088 -55.039  40.178  1.00 26.07           C
ATOM   3607  C   ALA A 473       6.384 -54.443  40.717  1.00 30.39           C
ATOM   3608  O   ALA A 473       6.756 -53.314  40.370  1.00 26.17           O
ATOM   3609  CB  ALA A 473       3.986 -54.940  41.202  1.00 25.01           C
ATOM   3610  N   GLN A 474       7.092 -55.230  41.522  1.00 25.20           N
ATOM   3611  CA  GLN A 474       8.337 -54.776  42.118  1.00 29.82           C
ATOM   3612  C   GLN A 474       9.398 -54.487  41.061  1.00 27.88           C
ATOM   3613  O   GLN A 474      10.139 -53.506  41.176  1.00 27.53           O
ATOM   3614  CB  GLN A 474       8.836 -55.768  43.185  1.00 29.59           C
ATOM   3615  CG  GLN A 474       7.972 -55.760  44.449  1.00 33.85           C
ATOM   3616  CD  GLN A 474       8.425 -56.758  45.511  1.00 40.20           C
ATOM   3617  OE1 GLN A 474       8.754 -57.911  45.216  1.00 42.75           O
ATOM   3618  NE2 GLN A 474       8.443 -56.310  46.758  1.00 36.28           N
ATOM   3619  N   ARG A 475       9.462 -55.332  40.033  1.00 26.86           N
ATOM   3620  CA  ARG A 475      10.363 -55.102  38.912  1.00 28.86           C
```

ATOM	3621	C	ARG	A	475	10.015	-53.798	38.203	1.00	29.12	C
ATOM	3622	O	ARG	A	475	10.901	-53.032	37.816	1.00	27.91	O
ATOM	3623	CB	ARG	A	475	10.250	-56.224	37.884	1.00	34.20	C
ATOM	3624	CG	ARG	A	475	10.961	-57.512	38.202	1.00	34.85	C
ATOM	3625	CD	ARG	A	475	10.714	-58.500	37.052	1.00	39.28	C
ATOM	3626	NE	ARG	A	475	11.138	-57.941	35.768	1.00	37.71	N
ATOM	3627	CZ	ARG	A	475	10.576	-58.226	34.596	1.00	40.54	C
ATOM	3628	NH1	ARG	A	475	9.548	-59.066	34.534	1.00	36.14	N
ATOM	3629	NH2	ARG	A	475	11.041	-57.665	33.482	1.00	29.23	N
ATOM	3630	N	LEU	A	476	8.718	-53.569	38.011	1.00	23.61	N
ATOM	3631	CA	LEU	A	476	8.263	-52.412	37.250	1.00	28.36	C
ATOM	3632	C	LEU	A	476	8.591	-51.131	37.991	1.00	27.63	C
ATOM	3633	O	LEU	A	476	9.069	-50.153	37.394	1.00	27.93	O
ATOM	3634	CB	LEU	A	476	6.761	-52.499	36.964	1.00	26.97	C
ATOM	3635	CG	LEU	A	476	6.377	-53.590	35.956	1.00	34.32	C
ATOM	3636	CD1	LEU	A	476	4.902	-53.551	35.649	1.00	32.76	C
ATOM	3637	CD2	LEU	A	476	7.174	-53.460	34.675	1.00	25.37	C
ATOM	3638	N	MET	A	477	8.346	-51.150	39.299	1.00	27.14	N
ATOM	3639	CA	MET	A	477	8.607	-49.996	40.142	1.00	24.45	C
ATOM	3640	C	MET	A	477	10.085	-49.668	40.094	1.00	24.66	C
ATOM	3641	O	MET	A	477	10.462	-48.502	40.034	1.00	27.59	O
ATOM	3642	CB	MET	A	477	8.161	-50.264	41.582	1.00	25.29	C
ATOM	3643	CG	MET	A	477	6.654	-50.283	41.768	1.00	25.66	C
ATOM	3644	SD	MET	A	477	6.149	-50.206	43.510	1.00	32.43	S
ATOM	3645	CE	MET	A	477	6.906	-51.699	44.156	1.00	24.62	C
ATOM	3646	N	ARG	A	478	10.918	-50.705	40.091	1.00	25.39	N
ATOM	3647	CA	ARG	A	478	12.368	-50.524	40.005	1.00	26.99	C
ATOM	3648	C	ARG	A	478	12.789	-49.931	38.650	1.00	29.83	C
ATOM	3649	O	ARG	A	478	13.630	-49.021	38.607	1.00	25.83	O
ATOM	3650	CB	ARG	A	478	13.099	-51.846	40.291	1.00	25.28	C
ATOM	3651	CG	ARG	A	478	14.621	-51.771	40.241	1.00	27.48	C
ATOM	3652	CD	ARG	A	478	15.194	-50.802	41.280	1.00	30.70	C
ATOM	3653	NE	ARG	A	478	14.768	-51.151	42.631	1.00	32.52	N
ATOM	3654	CZ	ARG	A	478	15.281	-52.156	43.337	1.00	38.35	C
ATOM	3655	NH1	ARG	A	478	16.237	-52.917	42.815	1.00	30.62	N
ATOM	3656	NH2	ARG	A	478	14.833	-52.405	44.561	1.00	35.06	N
ATOM	3657	N	TYR	A	479	12.208	-50.427	37.551	1.00	22.03	N
ATOM	3658	CA	TYR	A	479	12.502	-49.847	36.233	1.00	24.29	C
ATOM	3659	C	TYR	A	479	12.146	-48.363	36.229	1.00	27.25	C
ATOM	3660	O	TYR	A	479	12.948	-47.524	35.813	1.00	26.50	O
ATOM	3661	CB	TYR	A	479	11.728	-50.538	35.094	1.00	27.15	C
ATOM	3662	CG	TYR	A	479	12.195	-51.927	34.734	1.00	22.83	C
ATOM	3663	CD1	TYR	A	479	13.544	-52.232	34.652	1.00	23.39	C
ATOM	3664	CD2	TYR	A	479	11.277	-52.938	34.473	1.00	26.78	C

```
ATOM   3665  CE1 TYR A 479      13.968 -53.510  34.316  1.00 28.76           C
ATOM   3666  CE2 TYR A 479      11.689 -54.217  34.146  1.00 24.10           C
ATOM   3667  CZ  TYR A 479      13.034 -54.498  34.067  1.00 25.13           C
ATOM   3668  OH  TYR A 479      13.446 -55.771  33.741  1.00 34.94           O
ATOM   3669  N   TRP A 480      10.940 -48.045  36.699  1.00 24.54           N
ATOM   3670  CA  TRP A 480      10.443 -46.674  36.634  1.00 24.17           C
ATOM   3671  C   TRP A 480      11.271 -45.719  37.494  1.00 26.89           C
ATOM   3672  O   TRP A 480      11.618 -44.616  37.067  1.00 27.20           O
ATOM   3673  CB  TRP A 480       8.969 -46.623  37.044  1.00 22.42           C
ATOM   3674  CG  TRP A 480       8.006 -46.489  35.887  1.00 26.25           C
ATOM   3675  CD1 TRP A 480       7.135 -45.463  35.665  1.00 23.10           C
ATOM   3676  CD2 TRP A 480       7.821 -47.416  34.803  1.00 21.45           C
ATOM   3677  NE1 TRP A 480       6.417 -45.694  34.519  1.00 21.76           N
ATOM   3678  CE2 TRP A 480       6.816 -46.885  33.970  1.00 23.24           C
ATOM   3679  CE3 TRP A 480       8.400 -48.650  34.464  1.00 21.48           C
ATOM   3680  CZ2 TRP A 480       6.381 -47.537  32.803  1.00 19.76           C
ATOM   3681  CZ3 TRP A 480       7.966 -49.298  33.316  1.00 22.23           C
ATOM   3682  CH2 TRP A 480       6.966 -48.737  32.496  1.00 19.06           C
ATOM   3683  N   ALA A 481      11.600 -46.157  38.702  1.00 28.09           N
ATOM   3684  CA  ALA A 481      12.350 -45.321  39.633  1.00 27.33           C
ATOM   3685  C   ALA A 481      13.820 -45.198  39.227  1.00 27.15           C
ATOM   3686  O   ALA A 481      14.445 -44.152  39.446  1.00 23.91           O
ATOM   3687  CB  ALA A 481      12.216 -45.852  41.042  1.00 23.02           C
ATOM   3688  N   ASN A 482      14.364 -46.259  38.628  1.00 23.70           N
ATOM   3689  CA  ASN A 482      15.715 -46.193  38.077  1.00 25.11           C
ATOM   3690  C   ASN A 482      15.743 -45.107  37.033  1.00 29.68           C
ATOM   3691  O   ASN A 482      16.675 -44.297  36.978  1.00 28.76           O
ATOM   3692  CB  ASN A 482      16.141 -47.522  37.435  1.00 25.27           C
ATOM   3693  CG  ASN A 482      16.683 -48.522  38.447  1.00 27.86           C
ATOM   3694  OD1 ASN A 482      16.720 -48.250  39.646  1.00 33.86           O
ATOM   3695  ND2 ASN A 482      17.091 -49.692  37.968  1.00 29.67           N
ATOM   3696  N   PHE A 483      14.711 -45.098  36.193  1.00 25.96           N
ATOM   3697  CA  PHE A 483      14.640 -44.120  35.126  1.00 26.05           C
ATOM   3698  C   PHE A 483      14.540 -42.719  35.708  1.00 22.26           C
ATOM   3699  O   PHE A 483      15.206 -41.803  35.243  1.00 25.85           O
ATOM   3700  CB  PHE A 483      13.473 -44.393  34.169  1.00 26.10           C
ATOM   3701  CG  PHE A 483      13.280 -43.313  33.152  1.00 23.19           C
ATOM   3702  CD1 PHE A 483      14.090 -43.258  32.017  1.00 23.62           C
ATOM   3703  CD2 PHE A 483      12.320 -42.324  33.343  1.00 17.43           C
ATOM   3704  CE1 PHE A 483      13.929 -42.247  31.073  1.00 20.16           C
ATOM   3705  CE2 PHE A 483      12.155 -41.311  32.410  1.00 23.53           C
ATOM   3706  CZ  PHE A 483      12.963 -41.273  31.270  1.00 23.72           C
ATOM   3707  N   ALA A 484      13.710 -42.554  36.731  1.00 23.45           N
ATOM   3708  CA  ALA A 484      13.603 -41.263  37.399  1.00 29.16           C
```

```
ATOM   3709  C   ALA A 484      14.951 -40.789  37.968  1.00 29.25           C
ATOM   3710  O   ALA A 484      15.270 -39.598  37.940  1.00 32.60           O
ATOM   3711  CB  ALA A 484      12.547 -41.321  38.492  1.00 21.04           C
ATOM   3712  N   ARG A 485      15.739 -41.732  38.471  1.00 26.79           N
ATOM   3713  CA  ARG A 485      16.995 -41.409  39.145  1.00 28.99           C
ATOM   3714  C   ARG A 485      18.135 -41.140  38.183  1.00 31.97           C
ATOM   3715  O   ARG A 485      18.966 -40.281  38.455  1.00 33.66           O
ATOM   3716  CB  ARG A 485      17.406 -42.527  40.110  1.00 25.28           C
ATOM   3717  CG  ARG A 485      16.640 -42.521  41.427  1.00 28.48           C
ATOM   3718  CD  ARG A 485      17.159 -43.590  42.390  1.00 25.28           C
ATOM   3719  NE  ARG A 485      16.870 -44.939  41.907  1.00 30.49           N
ATOM   3720  CZ  ARG A 485      15.861 -45.684  42.344  1.00 25.28           C
ATOM   3721  NH1 ARG A 485      15.051 -45.209  43.275  1.00 22.07           N
ATOM   3722  NH2 ARG A 485      15.662 -46.898  41.850  1.00 23.96           N
ATOM   3723  N   THR A 486      18.173 -41.862  37.062  1.00 25.50           N
ATOM   3724  CA  THR A 486      19.358 -41.844  36.200  1.00 29.27           C
ATOM   3725  C   THR A 486      19.074 -41.582  34.729  1.00 32.71           C
ATOM   3726  O   THR A 486      20.002 -41.377  33.945  1.00 35.12           O
ATOM   3727  CB  THR A 486      20.110 -43.192  36.240  1.00 29.91           C
ATOM   3728  OG1 THR A 486      19.317 -44.199  35.598  1.00 31.78           O
ATOM   3729  CG2 THR A 486      20.426 -43.618  37.675  1.00 25.34           C
ATOM   3730  N   GLY A 487      17.804 -41.611  34.347  1.00 29.49           N
ATOM   3731  CA  GLY A 487      17.447 -41.552  32.943  1.00 32.73           C
ATOM   3732  C   GLY A 487      17.634 -42.891  32.251  1.00 30.26           C
ATOM   3733  O   GLY A 487      17.620 -42.973  31.025  1.00 32.67           O
ATOM   3734  N   ASP A 488      17.778 -43.952  33.037  1.00 30.84           N
ATOM   3735  CA  ASP A 488      18.014 -45.285  32.480  1.00 29.60           C
ATOM   3736  C   ASP A 488      17.330 -46.331  33.364  1.00 28.21           C
ATOM   3737  O   ASP A 488      17.704 -46.501  34.525  1.00 34.25           O
ATOM   3738  CB  ASP A 488      19.525 -45.529  32.427  1.00 28.42           C
ATOM   3739  CG  ASP A 488      19.905 -46.816  31.735  1.00 32.19           C
ATOM   3740  OD1 ASP A 488      19.074 -47.750  31.652  1.00 34.36           O
ATOM   3741  OD2 ASP A 488      21.070 -46.892  31.287  1.00 39.74           O
ATOM   3742  N   PRO A 489      16.339 -47.050  32.816  1.00 25.19           N
ATOM   3743  CA  PRO A 489      15.582 -48.004  33.636  1.00 27.20           C
ATOM   3744  C   PRO A 489      16.403 -49.220  34.072  1.00 26.45           C
ATOM   3745  O   PRO A 489      16.061 -49.853  35.070  1.00 30.78           O
ATOM   3746  CB  PRO A 489      14.450 -48.435  32.703  1.00 22.05           C
ATOM   3747  CG  PRO A 489      15.042 -48.301  31.332  1.00 24.29           C
ATOM   3748  CD  PRO A 489      15.927 -47.089  31.401  1.00 26.32           C
ATOM   3749  N   ASN A 490      17.478 -49.526  33.353  1.00 26.51           N
ATOM   3750  CA  ASN A 490      18.256 -50.735  33.617  1.00 28.38           C
ATOM   3751  C   ASN A 490      18.865 -50.876  35.013  1.00 38.06           C
ATOM   3752  O   ASN A 490      19.470 -49.939  35.545  1.00 35.96           O
```

```
ATOM   3753  CB  ASN A 490      19.372 -50.889  32.589  1.00 33.66           C
ATOM   3754  CG  ASN A 490      18.855 -51.239  31.227  1.00 29.57           C
ATOM   3755  OD1 ASN A 490      18.514 -52.391  30.955  1.00 30.73           O
ATOM   3756  ND2 ASN A 490      18.793 -50.251  30.355  1.00 26.79           N
ATOM   3757  N   GLU A 491      18.692 -52.070  35.579  1.00 36.91           N
ATOM   3758  CA  GLU A 491      19.430 -52.545  36.741  1.00 47.06           C
ATOM   3759  C   GLU A 491      20.857 -52.927  36.318  1.00 56.09           C
ATOM   3760  O   GLU A 491      21.156 -52.975  35.122  1.00 55.45           O
ATOM   3761  CB  GLU A 491      18.718 -53.781  37.302  1.00 54.15           C
ATOM   3762  CG  GLU A 491      17.444 -53.496  38.089  1.00 50.58           C
ATOM   3763  CD  GLU A 491      17.732 -53.137  39.534  1.00 47.29           C
ATOM   3764  OE1 GLU A 491      18.425 -52.127  39.768  1.00 48.14           O
ATOM   3765  OE2 GLU A 491      17.279 -53.873  40.437  1.00 55.32           O
ATOM   3766  N   PRO A 492      21.749 -53.190  37.295  1.00 64.54           N
ATOM   3767  CA  PRO A 492      23.075 -53.744  36.969  1.00 68.23           C
ATOM   3768  C   PRO A 492      23.011 -55.049  36.159  1.00 72.66           C
ATOM   3769  O   PRO A 492      22.128 -55.876  36.394  1.00 72.80           O
ATOM   3770  CB  PRO A 492      23.687 -54.002  38.346  1.00 54.34           C
ATOM   3771  CG  PRO A 492      23.086 -52.934  39.210  1.00 59.22           C
ATOM   3772  CD  PRO A 492      21.671 -52.748  38.703  1.00 61.59           C
ATOM   3773  N   ARG A 493      23.955 -55.221  35.232  1.00 71.22           N
ATOM   3774  CA  ARG A 493      23.938 -56.325  34.266  1.00 77.82           C
ATOM   3775  C   ARG A 493      23.827 -57.712  34.901  1.00 83.29           C
ATOM   3776  O   ARG A 493      24.351 -57.954  35.993  1.00 79.84           O
ATOM   3777  CB  ARG A 493      25.168 -56.265  33.353  1.00 66.68           C
ATOM   3778  CG  ARG A 493      25.714 -54.862  33.142  1.00 80.52           C
ATOM   3779  CD  ARG A 493      26.641 -54.798  31.939  1.00 84.61           C
ATOM   3780  NE  ARG A 493      25.890 -54.696  30.692  1.00 89.10           N
ATOM   3781  CZ  ARG A 493      26.441 -54.693  29.482  1.00102.39           C
ATOM   3782  NH1 ARG A 493      27.758 -54.794  29.350  1.00104.92           N
ATOM   3783  NH2 ARG A 493      25.673 -54.593  28.402  1.00 94.94           N
ATOM   3784  N   ASP A 494      23.148 -58.613  34.192  1.00 83.07           N
ATOM   3785  CA  ASP A 494      22.843 -59.951  34.688  1.00 87.34           C
ATOM   3786  C   ASP A 494      22.234 -60.800  33.571  1.00 86.15           C
ATOM   3787  O   ASP A 494      22.606 -61.959  33.375  1.00 83.72           O
ATOM   3788  CB  ASP A 494      21.873 -59.864  35.872  1.00 94.18           C
ATOM   3789  CG  ASP A 494      21.521 -61.224  36.451  1.00 94.94           C
ATOM   3790  OD1 ASP A 494      22.326 -62.166  36.299  1.00 94.47           O
ATOM   3791  OD2 ASP A 494      20.438 -61.347  37.066  1.00 90.21           O
ATOM   3792  N   PRO A 498      19.131 -59.506  31.313  1.00 63.12           N
ATOM   3793  CA  PRO A 498      19.066 -58.993  29.934  1.00 66.77           C
ATOM   3794  C   PRO A 498      18.794 -57.489  29.924  1.00 55.82           C
ATOM   3795  O   PRO A 498      17.949 -57.016  30.692  1.00 49.71           O
ATOM   3796  CB  PRO A 498      17.880 -59.756  29.318  1.00 64.10           C
```

ATOM	3797	CG	PRO	A	498	17.603	-60.897	30.261	1.00	67.85	C
ATOM	3798	CD	PRO	A	498	18.011	-60.410	31.620	1.00	63.57	C
ATOM	3799	N	GLN	A	499	19.502	-56.748	29.077	1.00	39.26	N
ATOM	3800	CA	GLN	A	499	19.427	-55.293	29.123	1.00	42.50	C
ATOM	3801	C	GLN	A	499	18.319	-54.703	28.241	1.00	42.99	C
ATOM	3802	O	GLN	A	499	17.989	-55.236	27.176	1.00	38.38	O
ATOM	3803	CB	GLN	A	499	20.780	-54.664	28.774	1.00	41.69	C
ATOM	3804	CG	GLN	A	499	21.881	-54.944	29.794	1.00	55.30	C
ATOM	3805	CD	GLN	A	499	21.565	-54.409	31.189	1.00	52.96	C
ATOM	3806	OE1	GLN	A	499	21.734	-53.218	31.467	1.00	50.39	O
ATOM	3807	NE2	GLN	A	499	21.115	-55.292	32.073	1.00	49.34	N
ATOM	3808	N	TRP	A	500	17.760	-53.594	28.713	1.00	33.96	N
ATOM	3809	CA	TRP	A	500	16.747	-52.842	27.999	1.00	27.88	C
ATOM	3810	C	TRP	A	500	17.475	-51.791	27.177	1.00	29.91	C
ATOM	3811	O	TRP	A	500	17.979	-50.818	27.725	1.00	31.03	O
ATOM	3812	CB	TRP	A	500	15.802	-52.195	29.019	1.00	31.86	C
ATOM	3813	CG	TRP	A	500	14.600	-51.482	28.464	1.00	31.86	C
ATOM	3814	CD1	TRP	A	500	14.399	-51.068	27.172	1.00	28.00	C
ATOM	3815	CD2	TRP	A	500	13.427	-51.091	29.195	1.00	30.66	C
ATOM	3816	NE1	TRP	A	500	13.178	-50.446	27.061	1.00	26.59	N
ATOM	3817	CE2	TRP	A	500	12.559	-50.450	28.283	1.00	28.29	C
ATOM	3818	CE3	TRP	A	500	13.025	-51.230	30.530	1.00	26.84	C
ATOM	3819	CZ2	TRP	A	500	11.313	-49.948	28.665	1.00	23.19	C
ATOM	3820	CZ3	TRP	A	500	11.796	-50.727	30.909	1.00	24.10	C
ATOM	3821	CH2	TRP	A	500	10.951	-50.093	29.980	1.00	25.67	C
ATOM	3822	N	PRO	A	501	17.539	-51.993	25.851	1.00	31.98	N
ATOM	3823	CA	PRO	A	501	18.247	-51.097	24.932	1.00	29.37	C
ATOM	3824	C	PRO	A	501	17.417	-49.880	24.564	1.00	27.98	C
ATOM	3825	O	PRO	A	501	16.193	-49.964	24.530	1.00	31.20	O
ATOM	3826	CB	PRO	A	501	18.441	-51.957	23.672	1.00	29.46	C
ATOM	3827	CG	PRO	A	501	17.643	-53.216	23.890	1.00	35.99	C
ATOM	3828	CD	PRO	A	501	16.817	-53.049	25.127	1.00	28.00	C
ATOM	3829	N	PRO	A	502	18.077	-48.759	24.266	1.00	27.69	N
ATOM	3830	CA	PRO	A	502	17.372	-47.566	23.784	1.00	25.13	C
ATOM	3831	C	PRO	A	502	16.678	-47.790	22.424	1.00	33.90	C
ATOM	3832	O	PRO	A	502	17.188	-48.499	21.549	1.00	27.98	O
ATOM	3833	CB	PRO	A	502	18.494	-46.530	23.641	1.00	30.60	C
ATOM	3834	CG	PRO	A	502	19.579	-47.012	24.579	1.00	32.65	C
ATOM	3835	CD	PRO	A	502	19.511	-48.511	24.492	1.00	28.87	C
ATOM	3836	N	TYR	A	503	15.504	-47.185	22.272	1.00	28.46	N
ATOM	3837	CA	TYR	A	503	14.759	-47.208	21.027	1.00	28.73	C
ATOM	3838	C	TYR	A	503	15.367	-46.205	20.063	1.00	24.32	C
ATOM	3839	O	TYR	A	503	15.677	-45.090	20.445	1.00	24.28	O
ATOM	3840	CB	TYR	A	503	13.297	-46.839	21.292	1.00	29.16	C

```
ATOM   3841  CG  TYR A 503      12.396 -46.838  20.070  1.00 26.35           C
ATOM   3842  CD1 TYR A 503      12.233 -45.688  19.297  1.00 28.54           C
ATOM   3843  CD2 TYR A 503      11.684 -47.976  19.710  1.00 24.26           C
ATOM   3844  CE1 TYR A 503      11.403 -45.683  18.186  1.00 25.71           C
ATOM   3845  CE2 TYR A 503      10.846 -47.980  18.608  1.00 24.90           C
ATOM   3846  CZ  TYR A 503      10.710 -46.835  17.851  1.00 25.92           C
ATOM   3847  OH  TYR A 503       9.878 -46.845  16.755  1.00 32.62           O
ATOM   3848  N   THR A 504      15.539 -46.605  18.812  1.00 23.47           N
ATOM   3849  CA  THR A 504      16.037 -45.696  17.778  1.00 30.76           C
ATOM   3850  C   THR A 504      15.178 -45.822  16.526  1.00 26.81           C
ATOM   3851  O   THR A 504      14.515 -46.846  16.325  1.00 25.17           O
ATOM   3852  CB  THR A 504      17.495 -46.010  17.407  1.00 27.90           C
ATOM   3853  OG1 THR A 504      17.595 -47.391  17.048  1.00 29.77           O
ATOM   3854  CG2 THR A 504      18.421 -45.744  18.591  1.00 28.51           C
ATOM   3855  N   ALA A 505      15.190 -44.787  15.689  1.00 32.27           N
ATOM   3856  CA  ALA A 505      14.382 -44.779  14.464  1.00 29.23           C
ATOM   3857  C   ALA A 505      14.706 -45.953  13.539  1.00 28.05           C
ATOM   3858  O   ALA A 505      13.829 -46.460  12.848  1.00 32.22           O
ATOM   3859  CB  ALA A 505      14.538 -43.462  13.725  1.00 25.35           C
ATOM   3860  N   GLY A 506      15.959 -46.398  13.546  1.00 27.54           N
ATOM   3861  CA  GLY A 506      16.358 -47.520  12.717  1.00 23.18           C
ATOM   3862  C   GLY A 506      16.062 -48.897  13.281  1.00 27.55           C
ATOM   3863  O   GLY A 506      15.261 -49.657  12.724  1.00 28.33           O
ATOM   3864  N   ALA A 507      16.727 -49.246  14.377  1.00 26.56           N
ATOM   3865  CA  ALA A 507      16.548 -50.578  14.956  1.00 27.12           C
ATOM   3866  C   ALA A 507      15.188 -50.745  15.640  1.00 23.57           C
ATOM   3867  O   ALA A 507      14.659 -51.853  15.700  1.00 26.83           O
ATOM   3868  CB  ALA A 507      17.693 -50.918  15.904  1.00 18.80           C
ATOM   3869  N   GLN A 508      14.634 -49.644  16.148  1.00 24.61           N
ATOM   3870  CA  GLN A 508      13.268 -49.634  16.684  1.00 27.10           C
ATOM   3871  C   GLN A 508      13.062 -50.643  17.813  1.00 27.62           C
ATOM   3872  O   GLN A 508      12.025 -51.305  17.894  1.00 27.55           O
ATOM   3873  CB  GLN A 508      12.254 -49.899  15.565  1.00 24.57           C
ATOM   3874  CG  GLN A 508      12.266 -48.861  14.446  1.00 28.27           C
ATOM   3875  CD  GLN A 508      11.436 -49.292  13.244  1.00 31.38           C
ATOM   3876  OE1 GLN A 508      10.214 -49.382  13.319  1.00 32.07           O
ATOM   3877  NE2 GLN A 508      12.102 -49.576  12.138  1.00 28.93           N
ATOM   3878  N   GLN A 509      14.057 -50.773  18.681  1.00 27.85           N
ATOM   3879  CA  GLN A 509      14.005 -51.796  19.718  1.00 29.36           C
ATOM   3880  C   GLN A 509      13.170 -51.376  20.925  1.00 25.85           C
ATOM   3881  O   GLN A 509      13.183 -50.217  21.339  1.00 22.12           O
ATOM   3882  CB  GLN A 509      15.416 -52.195  20.149  1.00 28.51           C
ATOM   3883  CG  GLN A 509      16.133 -53.106  19.159  1.00 29.19           C
ATOM   3884  CD  GLN A 509      17.601 -53.312  19.512  1.00 30.77           C
```

ATOM	3885	OE1	GLN	A	509	18.357	-52.351	19.649	1.00	36.45	O
ATOM	3886	NE2	GLN	A	509	18.005	-54.566	19.659	1.00	28.18	N
ATOM	3887	N	TYR	A	510	12.433	-52.333	21.472	1.00	25.86	N
ATOM	3888	CA	TYR	A	510	11.661	-52.112	22.686	1.00	27.98	C
ATOM	3889	C	TYR	A	510	11.641	-53.433	23.446	1.00	28.62	C
ATOM	3890	O	TYR	A	510	12.032	-54.458	22.888	1.00	28.55	O
ATOM	3891	CB	TYR	A	510	10.238	-51.689	22.330	1.00	27.05	C
ATOM	3892	CG	TYR	A	510	9.455	-52.770	21.627	1.00	29.50	C
ATOM	3893	CD1	TYR	A	510	9.493	-52.899	20.236	1.00	28.48	C
ATOM	3894	CD2	TYR	A	510	8.687	-53.676	22.350	1.00	24.20	C
ATOM	3895	CE1	TYR	A	510	8.773	-53.891	19.594	1.00	24.00	C
ATOM	3896	CE2	TYR	A	510	7.975	-54.672	21.714	1.00	28.06	C
ATOM	3897	CZ	TYR	A	510	8.018	-54.772	20.341	1.00	27.12	C
ATOM	3898	OH	TYR	A	510	7.303	-55.770	19.729	1.00	35.61	O
ATOM	3899	N	VAL	A	511	11.187	-53.424	24.699	1.00	24.76	N
ATOM	3900	CA	VAL	A	511	11.057	-54.673	25.458	1.00	22.25	C
ATOM	3901	C	VAL	A	511	9.611	-55.033	25.753	1.00	23.39	C
ATOM	3902	O	VAL	A	511	8.739	-54.168	25.803	1.00	24.42	O
ATOM	3903	CB	VAL	A	511	11.817	-54.644	26.807	1.00	28.67	C
ATOM	3904	CG1	VAL	A	511	13.320	-54.515	26.583	1.00	25.52	C
ATOM	3905	CG2	VAL	A	511	11.278	-53.532	27.701	1.00	20.79	C
ATOM	3906	N	SER	A	512	9.361	-56.321	25.952	1.00	22.83	N
ATOM	3907	CA	SER	A	512	8.063	-56.768	26.428	1.00	24.93	C
ATOM	3908	C	SER	A	512	8.090	-56.818	27.952	1.00	31.13	C
ATOM	3909	O	SER	A	512	9.027	-57.339	28.550	1.00	26.63	O
ATOM	3910	CB	SER	A	512	7.722	-58.143	25.853	1.00	29.55	C
ATOM	3911	OG	SER	A	512	8.750	-59.069	26.151	1.00	40.37	O
ATOM	3912	N	LEU	A	513	7.065	-56.255	28.576	1.00	31.52	N
ATOM	3913	CA	LEU	A	513	6.943	-56.295	30.020	1.00	25.58	C
ATOM	3914	C	LEU	A	513	5.878	-57.301	30.433	1.00	31.83	C
ATOM	3915	O	LEU	A	513	4.678	-57.064	30.235	1.00	29.08	O
ATOM	3916	CB	LEU	A	513	6.599	-54.905	30.567	1.00	22.76	C
ATOM	3917	CG	LEU	A	513	7.677	-53.833	30.392	1.00	29.66	C
ATOM	3918	CD1	LEU	A	513	7.238	-52.492	31.005	1.00	21.14	C
ATOM	3919	CD2	LEU	A	513	9.002	-54.315	30.984	1.00	23.57	C
ATOM	3920	N	ASP	A	514	6.320	-58.428	30.988	1.00	25.47	N
ATOM	3921	CA	ASP	A	514	5.407	-59.412	31.565	1.00	33.20	C
ATOM	3922	C	ASP	A	514	6.122	-60.273	32.604	1.00	34.83	C
ATOM	3923	O	ASP	A	514	7.216	-59.925	33.050	1.00	35.18	O
ATOM	3924	CB	ASP	A	514	4.743	-60.278	30.485	1.00	34.97	C
ATOM	3925	CG	ASP	A	514	5.746	-61.020	29.618	1.00	40.28	C
ATOM	3926	OD1	ASP	A	514	6.953	-61.034	29.951	1.00	38.65	O
ATOM	3927	OD2	ASP	A	514	5.313	-61.605	28.603	1.00	45.51	O
ATOM	3928	N	LEU	A	515	5.512	-61.394	32.978	1.00	35.40	N

ATOM	3929	CA	LEU	A	515	6.066	-62.252	34.030	1.00	36.59	C
ATOM	3930	C	LEU	A	515	7.409	-62.867	33.650	1.00	34.58	C
ATOM	3931	O	LEU	A	515	8.247	-63.104	34.514	1.00	45.52	O
ATOM	3932	CB	LEU	A	515	5.080	-63.351	34.403	1.00	30.84	C
ATOM	3933	CG	LEU	A	515	3.721	-62.889	34.918	1.00	33.74	C
ATOM	3934	CD1	LEU	A	515	2.898	-64.101	35.332	1.00	35.09	C
ATOM	3935	CD2	LEU	A	515	3.876	-61.912	36.078	1.00	35.24	C
ATOM	3936	N	ARG	A	516	7.606	-63.117	32.359	1.00	38.46	N
ATOM	3937	CA	ARG	A	516	8.869	-63.634	31.834	1.00	36.84	C
ATOM	3938	C	ARG	A	516	9.934	-62.550	31.883	1.00	34.70	C
ATOM	3939	O	ARG	A	516	9.610	-61.373	31.950	1.00	35.38	O
ATOM	3940	CB	ARG	A	516	8.693	-64.059	30.373	1.00	38.63	C
ATOM	3941	CG	ARG	A	516	7.643	-65.126	30.114	1.00	48.11	C
ATOM	3942	CD	ARG	A	516	7.464	-65.341	28.604	1.00	55.91	C
ATOM	3943	NE	ARG	A	516	7.071	-64.110	27.910	1.00	65.65	N
ATOM	3944	CZ	ARG	A	516	7.782	-63.509	26.954	1.00	60.23	C
ATOM	3945	NH1	ARG	A	516	8.939	-64.023	26.548	1.00	54.11	N
ATOM	3946	NH2	ARG	A	516	7.327	-62.392	26.394	1.00	54.91	N
ATOM	3947	N	PRO	A	517	11.216	-62.940	31.827	1.00	41.57	N
ATOM	3948	CA	PRO	A	517	12.293	-61.945	31.740	1.00	39.46	C
ATOM	3949	C	PRO	A	517	12.139	-61.051	30.508	1.00	40.76	C
ATOM	3950	O	PRO	A	517	11.484	-61.461	29.541	1.00	33.73	O
ATOM	3951	CB	PRO	A	517	13.547	-62.807	31.585	1.00	36.37	C
ATOM	3952	CG	PRO	A	517	13.188	-64.105	32.210	1.00	37.49	C
ATOM	3953	CD	PRO	A	517	11.738	-64.315	31.912	1.00	37.69	C
ATOM	3954	N	LEU	A	518	12.731	-59.856	30.553	1.00	36.34	N
ATOM	3955	CA	LEU	A	518	12.713	-58.928	29.425	1.00	34.47	C
ATOM	3956	C	LEU	A	518	13.071	-59.643	28.136	1.00	39.53	C
ATOM	3957	O	LEU	A	518	13.956	-60.494	28.107	1.00	40.55	O
ATOM	3958	CB	LEU	A	518	13.716	-57.791	29.621	1.00	34.68	C
ATOM	3959	CG	LEU	A	518	13.444	-56.626	30.564	1.00	38.57	C
ATOM	3960	CD1	LEU	A	518	14.541	-55.587	30.396	1.00	37.22	C
ATOM	3961	CD2	LEU	A	518	12.067	-56.014	30.324	1.00	35.34	C
ATOM	3962	N	GLU	A	519	12.369	-59.289	27.073	1.00	36.10	N
ATOM	3963	CA	GLU	A	519	12.646	-59.822	25.756	1.00	38.36	C
ATOM	3964	C	GLU	A	519	12.696	-58.634	24.809	1.00	33.00	C
ATOM	3965	O	GLU	A	519	11.774	-57.820	24.782	1.00	28.01	O
ATOM	3966	CB	GLU	A	519	11.549	-60.800	25.352	1.00	37.76	C
ATOM	3967	CG	GLU	A	519	11.774	-61.472	24.016	1.00	53.61	C
ATOM	3968	CD	GLU	A	519	10.568	-62.286	23.569	1.00	64.78	C
ATOM	3969	OE1	GLU	A	519	9.978	-62.997	24.419	1.00	64.60	O
ATOM	3970	OE2	GLU	A	519	10.208	-62.203	22.370	1.00	59.40	O
ATOM	3971	N	VAL	A	520	13.795	-58.506	24.072	1.00	33.56	N
ATOM	3972	CA	VAL	A	520	13.960	-57.395	23.144	1.00	29.57	C

```
ATOM   3973  C   VAL A 520      13.329 -57.734  21.803  1.00 33.97           C
ATOM   3974  O   VAL A 520      13.506 -58.835  21.297  1.00 36.71           O
ATOM   3975  CB  VAL A 520      15.436 -57.060  22.925  1.00 32.54           C
ATOM   3976  CG1 VAL A 520      15.583 -55.932  21.906  1.00 28.80           C
ATOM   3977  CG2 VAL A 520      16.079 -56.674  24.242  1.00 29.32           C
ATOM   3978  N   ARG A 521      12.574 -56.789  21.249  1.00 34.38           N
ATOM   3979  CA  ARG A 521      11.919 -56.966  19.961  1.00 29.48           C
ATOM   3980  C   ARG A 521      12.092 -55.720  19.117  1.00 30.63           C
ATOM   3981  O   ARG A 521      12.625 -54.715  19.591  1.00 37.34           O
ATOM   3982  CB  ARG A 521      10.441 -57.264  20.156  1.00 33.43           C
ATOM   3983  CG  ARG A 521      10.190 -58.522  20.942  1.00 36.19           C
ATOM   3984  CD  ARG A 521       8.721 -58.693  21.243  1.00 42.05           C
ATOM   3985  NE  ARG A 521       8.508 -59.858  22.093  1.00 47.35           N
ATOM   3986  CZ  ARG A 521       7.366 -60.134  22.707  1.00 53.69           C
ATOM   3987  NH1 ARG A 521       6.326 -59.320  22.564  1.00 51.75           N
ATOM   3988  NH2 ARG A 521       7.268 -61.221  23.466  1.00 51.57           N
ATOM   3989  N   ARG A 522      11.644 -55.791  17.868  1.00 31.29           N
ATOM   3990  CA  ARG A 522      11.818 -54.695  16.919  1.00 35.65           C
ATOM   3991  C   ARG A 522      10.484 -54.244  16.341  1.00 30.66           C
ATOM   3992  O   ARG A 522       9.683 -55.066  15.902  1.00 35.43           O
ATOM   3993  CB  ARG A 522      12.736 -55.109  15.758  1.00 31.85           C
ATOM   3994  CG  ARG A 522      14.173 -55.426  16.134  1.00 34.39           C
ATOM   3995  CD  ARG A 522      15.060 -55.463  14.889  1.00 27.26           C
ATOM   3996  NE  ARG A 522      15.037 -54.170  14.211  1.00 29.66           N
ATOM   3997  CZ  ARG A 522      14.858 -54.007  12.903  1.00 39.97           C
ATOM   3998  NH1 ARG A 522      14.705 -55.067  12.109  1.00 30.68           N
ATOM   3999  NH2 ARG A 522      14.842 -52.777  12.392  1.00 32.57           N
ATOM   4000  N   GLY A 523      10.258 -52.936  16.337  1.00 28.85           N
ATOM   4001  CA  GLY A 523       9.123 -52.362  15.639  1.00 29.20           C
ATOM   4002  C   GLY A 523       7.855 -52.220  16.451  1.00 29.85           C
ATOM   4003  O   GLY A 523       7.238 -53.207  16.853  1.00 27.16           O
ATOM   4004  N   LEU A 524       7.461 -50.976  16.695  1.00 26.31           N
ATOM   4005  CA  LEU A 524       6.166 -50.719  17.300  1.00 29.51           C
ATOM   4006  C   LEU A 524       5.114 -50.739  16.195  1.00 30.59           C
ATOM   4007  O   LEU A 524       4.674 -49.677  15.726  1.00 26.81           O
ATOM   4008  CB  LEU A 524       6.161 -49.369  18.019  1.00 21.81           C
ATOM   4009  CG  LEU A 524       7.065 -49.275  19.247  1.00 27.97           C
ATOM   4010  CD1 LEU A 524       7.188 -47.822  19.702  1.00 26.48           C
ATOM   4011  CD2 LEU A 524       6.517 -50.156  20.360  1.00 22.04           C
ATOM   4012  N   ARG A 525       4.719 -51.949  15.800  0.44 27.08           N
ATOM   4014  CA  ARG A 525       3.777 -52.159  14.706  0.44 30.14           C
ATOM   4016  C   ARG A 525       4.170 -51.315  13.504  0.44 29.07           C
ATOM   4018  O   ARG A 525       3.381 -50.519  12.988  0.44 28.48           O
ATOM   4020  CB  ARG A 525       2.344 -51.877  15.155  0.44 29.15           C
```

ATOM	4022	CG	ARG	A	525	1.892	-52.791	16.282	0.44	27.15	C
ATOM	4024	CD	ARG	A	525	0.393	-52.727	16.507	0.44	24.02	C
ATOM	4026	NE	ARG	A	525	-0.006	-53.576	17.625	0.44	26.68	N
ATOM	4028	CZ	ARG	A	525	-1.264	-53.845	17.952	0.44	28.56	C
ATOM	4030	NH1	ARG	A	525	-2.261	-53.328	17.244	0.44	24.69	N
ATOM	4032	NH2	ARG	A	525	-1.523	-54.635	18.990	0.44	26.19	N
ATOM	4034	N	ALA	A	526	5.417	-51.500	13.088	1.00	28.97	N
ATOM	4035	CA	ALA	A	526	6.027	-50.732	12.014	1.00	26.01	C
ATOM	4036	C	ALA	A	526	5.239	-50.714	10.698	1.00	25.97	C
ATOM	4037	O	ALA	A	526	5.096	-49.662	10.081	1.00	22.97	O
ATOM	4038	CB	ALA	A	526	7.460	-51.220	11.781	1.00	25.78	C
ATOM	4039	N	GLN	A	527	4.734	-51.869	10.276	1.00	25.99	N
ATOM	4040	CA	GLN	A	527	3.961	-51.931	9.041	1.00	28.75	C
ATOM	4041	C	GLN	A	527	2.699	-51.092	9.132	1.00	23.13	C
ATOM	4042	O	GLN	A	527	2.440	-50.275	8.261	1.00	31.77	O
ATOM	4043	CB	GLN	A	527	3.618	-53.372	8.655	1.00	21.52	C
ATOM	4044	CG	GLN	A	527	4.809	-54.324	8.650	1.00	31.69	C
ATOM	4045	CD	GLN	A	527	6.011	-53.840	7.818	1.00	36.82	C
ATOM	4046	OE1	GLN	A	527	7.166	-54.109	8.174	1.00	45.76	O
ATOM	4047	NE2	GLN	A	527	5.745	-53.162	6.703	1.00	24.42	N
ATOM	4048	N	ALA	A	528	1.924	-51.277	10.192	1.00	27.42	N
ATOM	4049	CA	ALA	A	528	0.703	-50.498	10.375	1.00	24.78	C
ATOM	4050	C	ALA	A	528	1.007	-49.014	10.589	1.00	25.74	C
ATOM	4051	O	ALA	A	528	0.308	-48.145	10.071	1.00	30.15	O
ATOM	4052	CB	ALA	A	528	-0.110	-51.048	11.540	1.00	26.45	C
ATOM	4053	N	CYS	A	529	2.053	-48.714	11.344	1.00	21.23	N
ATOM	4054	CA	CYS	A	529	2.338	-47.315	11.661	1.00	22.48	C
ATOM	4055	C	CYS	A	529	2.937	-46.528	10.486	1.00	26.74	C
ATOM	4056	O	CYS	A	529	2.814	-45.299	10.425	1.00	23.49	O
ATOM	4057	CB	CYS	A	529	3.186	-47.202	12.935	1.00	25.75	C
ATOM	4058	SG	CYS	A	529	2.258	-47.695	14.454	1.00	26.72	S
ATOM	4059	N	ALA	A	530	3.561	-47.233	9.543	1.00	22.48	N
ATOM	4060	CA	ALA	A	530	3.980	-46.601	8.295	1.00	24.24	C
ATOM	4061	C	ALA	A	530	2.763	-46.008	7.602	1.00	24.53	C
ATOM	4062	O	ALA	A	530	2.827	-44.914	7.036	1.00	24.08	O
ATOM	4063	CB	ALA	A	530	4.660	-47.603	7.385	1.00	21.84	C
ATOM	4064	N	PHE	A	531	1.650	-46.736	7.657	1.00	21.27	N
ATOM	4065	CA	PHE	A	531	0.407	-46.265	7.052	1.00	23.07	C
ATOM	4066	C	PHE	A	531	-0.102	-44.981	7.721	1.00	27.44	C
ATOM	4067	O	PHE	A	531	-0.340	-43.984	7.042	1.00	27.79	O
ATOM	4068	CB	PHE	A	531	-0.664	-47.365	7.078	1.00	25.82	C
ATOM	4069	CG	PHE	A	531	-2.056	-46.864	6.803	1.00	25.95	C
ATOM	4070	CD1	PHE	A	531	-2.458	-46.569	5.508	1.00	25.61	C
ATOM	4071	CD2	PHE	A	531	-2.963	-46.693	7.840	1.00	23.77	C

ATOM	4072	CE1	PHE	A	531	-3.741	-46.099	5.248	1.00	29.73	C
ATOM	4073	CE2	PHE	A	531	-4.243	-46.222	7.592	1.00	28.02	C
ATOM	4074	CZ	PHE	A	531	-4.636	-45.923	6.289	1.00	30.35	C
ATOM	4075	N	TRP	A	532	-0.242	-45.002	9.047	1.00	21.54	N
ATOM	4076	CA	TRP	A	532	-0.804	-43.863	9.776	1.00	23.16	C
ATOM	4077	C	TRP	A	532	0.125	-42.664	9.798	1.00	26.60	C
ATOM	4078	O	TRP	A	532	-0.333	-41.515	9.741	1.00	25.43	O
ATOM	4079	CB	TRP	A	532	-1.175	-44.248	11.223	1.00	19.76	C
ATOM	4080	CG	TRP	A	532	-2.256	-45.287	11.302	1.00	18.42	C
ATOM	4081	CD1	TRP	A	532	-2.101	-46.627	11.524	1.00	20.41	C
ATOM	4082	CD2	TRP	A	532	-3.660	-45.071	11.130	1.00	18.93	C
ATOM	4083	NE1	TRP	A	532	-3.322	-47.257	11.508	1.00	18.50	N
ATOM	4084	CE2	TRP	A	532	-4.296	-46.318	11.277	1.00	21.68	C
ATOM	4085	CE3	TRP	A	532	-4.442	-43.941	10.868	1.00	19.57	C
ATOM	4086	CZ2	TRP	A	532	-5.677	-46.465	11.167	1.00	23.43	C
ATOM	4087	CZ3	TRP	A	532	-5.813	-44.092	10.762	1.00	18.85	C
ATOM	4088	CH2	TRP	A	532	-6.414	-45.338	10.910	1.00	18.05	C
ATOM	4089	N	ASN	A	533	1.426	-42.930	9.882	1.00	26.46	N
ATOM	4090	CA	ASN	A	533	2.406	-41.866	10.085	1.00	23.38	C
ATOM	4091	C	ASN	A	533	3.015	-41.328	8.791	1.00	23.77	C
ATOM	4092	O	ASN	A	533	3.359	-40.153	8.710	1.00	24.32	O
ATOM	4093	CB	ASN	A	533	3.510	-42.301	11.070	1.00	22.14	C
ATOM	4094	CG	ASN	A	533	2.974	-42.568	12.487	1.00	27.69	C
ATOM	4095	OD1	ASN	A	533	1.935	-42.039	12.880	1.00	26.73	O
ATOM	4096	ND2	ASN	A	533	3.691	-43.392	13.252	1.00	21.16	N
ATOM	4097	N	ARG	A	534	3.149	-42.178	7.778	1.00	24.48	N
ATOM	4098	CA	ARG	A	534	3.812	-41.757	6.540	1.00	25.46	C
ATOM	4099	C	ARG	A	534	2.856	-41.520	5.382	1.00	24.93	C
ATOM	4100	O	ARG	A	534	2.909	-40.478	4.742	1.00	36.43	O
ATOM	4101	CB	ARG	A	534	4.875	-42.768	6.125	1.00	26.52	C
ATOM	4102	CG	ARG	A	534	5.956	-42.942	7.167	1.00	28.43	C
ATOM	4103	CD	ARG	A	534	6.888	-44.096	6.831	1.00	31.59	C
ATOM	4104	NE	ARG	A	534	7.785	-44.316	7.955	1.00	39.95	N
ATOM	4105	CZ	ARG	A	534	8.942	-43.685	8.117	1.00	40.83	C
ATOM	4106	NH1	ARG	A	534	9.359	-42.813	7.201	1.00	34.71	N
ATOM	4107	NH2	ARG	A	534	9.688	-43.940	9.186	1.00	40.23	N
ATOM	4108	N	PHE	A	535	1.983	-42.483	5.113	1.00	25.85	N
ATOM	4109	CA	PHE	A	535	1.089	-42.384	3.960	1.00	27.73	C
ATOM	4110	C	PHE	A	535	-0.180	-41.566	4.193	1.00	29.52	C
ATOM	4111	O	PHE	A	535	-0.463	-40.645	3.438	1.00	32.74	O
ATOM	4112	CB	PHE	A	535	0.696	-43.765	3.411	1.00	24.39	C
ATOM	4113	CG	PHE	A	535	-0.233	-43.685	2.238	1.00	26.16	C
ATOM	4114	CD1	PHE	A	535	0.227	-43.235	1.012	1.00	27.67	C
ATOM	4115	CD2	PHE	A	535	-1.571	-44.016	2.367	1.00	26.36	C

ATOM	4116	CE1	PHE	A	535	-0.633	-43.125	-0.075	1.00	29.19	C
ATOM	4117	CE2	PHE	A	535	-2.436	-43.917	1.289	1.00	32.17	C
ATOM	4118	CZ	PHE	A	535	-1.967	-43.471	0.065	1.00	31.02	C
ATOM	4119	N	LEU	A	536	-0.955	-41.918	5.216	1.00	32.97	N
ATOM	4120	CA	LEU	A	536	-2.271	-41.301	5.409	1.00	33.83	C
ATOM	4121	C	LEU	A	536	-2.303	-39.759	5.453	1.00	34.18	C
ATOM	4122	O	LEU	A	536	-3.211	-39.159	4.888	1.00	37.39	O
ATOM	4123	CB	LEU	A	536	-3.011	-41.909	6.600	1.00	27.61	C
ATOM	4124	CG	LEU	A	536	-4.518	-41.661	6.565	1.00	36.86	C
ATOM	4125	CD1	LEU	A	536	-5.148	-42.390	5.400	1.00	37.05	C
ATOM	4126	CD2	LEU	A	536	-5.172	-42.084	7.852	1.00	32.33	C
ATOM	4127	N	PRO	A	537	-1.322	-39.115	6.118	1.00	35.13	N
ATOM	4128	CA	PRO	A	537	-1.260	-37.649	6.016	1.00	32.33	C
ATOM	4129	C	PRO	A	537	-1.191	-37.139	4.576	1.00	39.65	C
ATOM	4130	O	PRO	A	537	-1.914	-36.198	4.243	1.00	43.30	O
ATOM	4131	CB	PRO	A	537	0.041	-37.306	6.739	1.00	31.23	C
ATOM	4132	CG	PRO	A	537	0.208	-38.397	7.738	1.00	34.87	C
ATOM	4133	CD	PRO	A	537	-0.346	-39.637	7.094	1.00	33.67	C
ATOM	4134	N	LYS	A	538	-0.335	-37.740	3.747	1.00	43.30	N
ATOM	4135	CA	LYS	A	538	-0.200	-37.342	2.338	1.00	38.29	C
ATOM	4136	C	LYS	A	538	-1.543	-37.403	1.631	1.00	38.99	C
ATOM	4137	O	LYS	A	538	-1.918	-36.486	0.906	1.00	49.85	O
ATOM	4138	CB	LYS	A	538	0.809	-38.233	1.607	1.00	32.88	C
ATOM	4139	CG	LYS	A	538	2.240	-38.121	2.139	1.00	37.25	C
ATOM	4140	CD	LYS	A	538	3.208	-38.962	1.328	1.00	34.07	C
ATOM	4141	CE	LYS	A	538	4.656	-38.625	1.672	1.00	40.29	C
ATOM	4142	NZ	LYS	A	538	5.029	-39.050	3.049	1.00	49.96	N
ATOM	4143	N	LEU	A	539	-2.265	-38.492	1.862	1.00	37.42	N
ATOM	4144	CA	LEU	A	539	-3.585	-38.679	1.289	1.00	44.09	C
ATOM	4145	C	LEU	A	539	-4.516	-37.539	1.685	1.00	43.65	C
ATOM	4146	O	LEU	A	539	-5.081	-36.868	0.824	1.00	49.22	O
ATOM	4147	CB	LEU	A	539	-4.158	-40.030	1.720	1.00	38.01	C
ATOM	4148	CG	LEU	A	539	-5.526	-40.403	1.158	1.00	44.98	C
ATOM	4149	CD1	LEU	A	539	-5.599	-41.889	0.905	1.00	37.82	C
ATOM	4150	CD2	LEU	A	539	-6.630	-39.979	2.108	1.00	41.41	C
ATOM	4151	N	LEU	A	540	-4.666	-37.322	2.988	1.00	53.11	N
ATOM	4152	CA	LEU	A	540	-5.513	-36.244	3.508	1.00	51.08	C
ATOM	4153	C	LEU	A	540	-5.178	-34.863	2.909	1.00	49.04	C
ATOM	4154	O	LEU	A	540	-6.078	-34.068	2.633	1.00	60.01	O
ATOM	4155	CB	LEU	A	540	-5.464	-36.203	5.042	1.00	42.63	C
ATOM	4156	CG	LEU	A	540	-5.946	-37.451	5.801	1.00	45.41	C
ATOM	4157	CD1	LEU	A	540	-5.959	-37.228	7.302	1.00	32.64	C
ATOM	4158	CD2	LEU	A	540	-7.317	-37.884	5.342	1.00	42.75	C
ATOM	4159	N	SER	A	541	-3.893	-34.598	2.682	1.00	46.80	N

```
ATOM   4160  CA  SER A 541      -3.448 -33.332   2.092  1.00 48.24           C
ATOM   4161  C   SER A 541      -3.767 -33.197   0.602  1.00 58.15           C
ATOM   4162  O   SER A 541      -3.442 -32.179  -0.008  1.00 60.94           O
ATOM   4163  CB  SER A 541      -1.944 -33.145   2.290  1.00 47.04           C
ATOM   4164  OG  SER A 541      -1.594 -33.272   3.655  1.00 58.30           O
ATOM   4165  N   ALA A 542      -4.384 -34.222   0.017  1.00 61.61           N
ATOM   4166  CA  ALA A 542      -4.797 -34.177  -1.385  1.00 61.36           C
ATOM   4167  C   ALA A 542      -6.292 -34.471  -1.547  1.00 62.64           C
ATOM   4168  O   ALA A 542      -7.111 -34.089  -0.702  1.00 61.06           O
ATOM   4169  CB  ALA A 542      -3.965 -35.142  -2.216  1.00 49.85           C
TER    4170      ALA A 542
```

Coordinates of BChE (7aiy.pdb) used for the docking study.

The search region of the enzyme was determined by setting the grid box around the center of the active site, consisting of 5 subdomains: peripheral anionic site (Asp70, Tyr114, Tyr128, Tyr332), anionic site (Trp82, Glu197, Phe329), oxyanion hole (Gly116, Gly117, Ala199), acyl pocket (Leu286, Val288) and esteratic site (Ser198, Glu325, His438).

```
ATOM      1  N   ASP A   3       2.890   3.256  13.791  1.00124.50           N
ATOM      2  CA  ASP A   3       3.333   1.907  14.138  1.00128.88           C
ATOM      3  C   ASP A   3       4.243   1.957  15.379  1.00127.25           C
ATOM      4  O   ASP A   3       5.350   2.510  15.322  1.00127.10           O
ATOM      5  CB  ASP A   3       4.064   1.267  12.948  1.00122.02           C
ATOM      6  CG  ASP A   3       3.855  -0.243  12.863  1.00117.02           C
ATOM      7  OD1 ASP A   3       3.209  -0.827  13.757  1.00118.85           O
ATOM      8  OD2 ASP A   3       4.345  -0.852  11.891  1.00126.34           O
ATOM      9  N   ILE A   4       3.781   1.397  16.500  1.00113.53           N
ATOM     10  CA  ILE A   4       4.507   1.463  17.766  1.00108.97           C
ATOM     11  C   ILE A   4       4.730   0.046  18.281  1.00102.77           C
ATOM     12  O   ILE A   4       3.780  -0.734  18.424  1.00102.95           O
ATOM     13  CB  ILE A   4       3.776   2.330  18.800  1.00105.31           C
ATOM     14  CG1 ILE A   4       4.586   2.397  20.106  1.00101.54           C
ATOM     15  CG2 ILE A   4       2.362   1.832  18.993  1.00103.96           C
ATOM     16  CD1 ILE A   4       5.606   3.531  20.139  1.00101.44           C
ATOM     17  N   ILE A   5       5.980  -0.276  18.587  1.00 98.65           N
ATOM     18  CA  ILE A   5       6.409  -1.663  18.710  1.00 92.73           C
ATOM     19  C   ILE A   5       7.633  -1.696  19.606  1.00 94.40           C
ATOM     20  O   ILE A   5       8.538  -0.872  19.450  1.00100.81           O
ATOM     21  CB  ILE A   5       6.702  -2.223  17.308  1.00 97.51           C
ATOM     22  CG1 ILE A   5       7.331  -3.597  17.369  1.00 94.68           C
ATOM     23  CG2 ILE A   5       7.615  -1.291  16.529  1.00102.37           C
ATOM     24  CD1 ILE A   5       7.129  -4.267  16.078  1.00 95.66           C
```

```
ATOM     25  N   ILE A   6       7.664  -2.595  20.580  1.00 84.52           N
ATOM     26  CA  ILE A   6       8.646  -2.418  21.637  1.00 93.00           C
ATOM     27  C   ILE A   6       9.186  -3.770  22.081  1.00101.11           C
ATOM     28  O   ILE A   6       8.477  -4.787  22.070  1.00100.84           O
ATOM     29  CB  ILE A   6       8.060  -1.573  22.796  1.00 86.92           C
ATOM     30  CG1 ILE A   6       9.188  -0.775  23.492  1.00 95.17           C
ATOM     31  CG2 ILE A   6       7.350  -2.455  23.780  1.00 91.09           C
ATOM     32  CD1 ILE A   6       8.764   0.419  24.292  1.00 86.70           C
ATOM     33  N   ALA A   7      10.480  -3.783  22.382  1.00 97.68           N
ATOM     34  CA  ALA A   7      11.245  -5.007  22.532  1.00100.09           C
ATOM     35  C   ALA A   7      11.159  -5.453  23.982  1.00101.44           C
ATOM     36  O   ALA A   7      11.610  -4.742  24.886  1.00104.67           O
ATOM     37  CB  ALA A   7      12.696  -4.783  22.101  1.00 90.13           C
ATOM     38  N   THR A   8      10.564  -6.614  24.205  1.00 98.55           N
ATOM     39  CA  THR A   8      10.584  -7.195  25.533  1.00 97.98           C
ATOM     40  C   THR A   8      11.833  -8.065  25.674  1.00105.74           C
ATOM     41  O   THR A   8      12.675  -8.146  24.772  1.00 99.60           O
ATOM     42  CB  THR A   8       9.316  -8.000  25.807  1.00101.34           C
ATOM     43  OG1 THR A   8       9.382  -8.517  27.138  1.00109.78           O
ATOM     44  CG2 THR A   8       9.217  -9.192  24.889  1.00 97.63           C
ATOM     45  N   LYS A   9      11.956  -8.729  26.824  1.00110.35           N
ATOM     46  CA  LYS A   9      13.022  -9.691  27.068  1.00105.30           C
ATOM     47  C   LYS A   9      12.850 -10.967  26.246  1.00101.88           C
ATOM     48  O   LYS A   9      13.795 -11.759  26.171  1.00110.71           O
ATOM     49  CB  LYS A   9      13.088  -9.985  28.585  1.00 95.57           C
ATOM     50  CG  LYS A   9      13.967 -11.159  29.056  1.00 96.30           C
ATOM     51  CD  LYS A   9      14.260 -11.073  30.569  1.00 96.56           C
ATOM     52  CE  LYS A   9      14.242 -12.448  31.258  1.00 98.62           C
ATOM     53  NZ  LYS A   9      12.959 -12.762  31.972  1.00 87.40           N
ATOM     54  N   ASN A  10      11.697 -11.170  25.586  1.00 99.14           N
ATOM     55  CA  ASN A  10      11.464 -12.420  24.861  1.00109.47           C
ATOM     56  C   ASN A  10      10.877 -12.193  23.471  1.00110.54           C
ATOM     57  O   ASN A  10      10.212 -13.078  22.917  1.00106.92           O
ATOM     58  CB  ASN A  10      10.546 -13.354  25.649  1.00103.55           C
ATOM     59  CG  ASN A  10      11.050 -13.615  27.035  1.00100.49           C
ATOM     60  OD1 ASN A  10      10.481 -13.125  28.007  1.00110.80           O
ATOM     61  ND2 ASN A  10      12.124 -14.393  27.144  1.00 95.58           N
ATOM     62  N   GLY A  11      11.126 -11.047  22.884  1.00106.02           N
ATOM     63  CA  GLY A  11      10.638 -10.786  21.554  1.00100.73           C
ATOM     64  C   GLY A  11      10.132  -9.373  21.485  1.00100.03           C
ATOM     65  O   GLY A  11      10.360  -8.571  22.385  1.00 98.97           O
ATOM     66  N   LYS A  12       9.500  -9.022  20.373  1.00106.48           N
ATOM     67  CA  LYS A  12       8.966  -7.676  20.236  1.00 98.88           C
ATOM     68  C   LYS A  12       7.489  -7.786  19.909  1.00 97.17           C
```

```
ATOM     69  O   LYS A  12       7.044  -8.772  19.324  1.00 95.00           O
ATOM     70  CB  LYS A  12       9.766  -6.792  19.238  1.00 89.59           C
ATOM     71  CG  LYS A  12      10.310  -7.220  17.898  1.00 92.19           C
ATOM     72  CD  LYS A  12       9.287  -7.002  16.717  1.00108.98           C
ATOM     73  CE  LYS A  12       9.920  -6.545  15.468  1.00117.98           C
ATOM     74  NZ  LYS A  12       8.768  -6.240  14.583  1.00127.65           N
ATOM     75  N   VAL A  13       6.718  -6.810  20.380  1.00 96.29           N
ATOM     76  CA  VAL A  13       5.259  -6.878  20.405  1.00 93.06           C
ATOM     77  C   VAL A  13       4.688  -5.597  19.796  1.00 78.85           C
ATOM     78  O   VAL A  13       5.168  -4.500  20.092  1.00 82.09           O
ATOM     79  CB  VAL A  13       4.746  -7.080  21.850  1.00 89.54           C
ATOM     80  CG1 VAL A  13       3.326  -6.584  22.034  1.00 93.02           C
ATOM     81  CG2 VAL A  13       4.853  -8.531  22.280  1.00 87.59           C
ATOM     82  N   ARG A  14       3.669  -5.724  18.949  1.00 76.04           N
ATOM     83  CA  ARG A  14       3.057  -4.569  18.295  1.00 95.11           C
ATOM     84  C   ARG A  14       1.752  -4.208  18.990  1.00 94.09           C
ATOM     85  O   ARG A  14       0.955  -5.096  19.314  1.00 90.02           O
ATOM     86  CB  ARG A  14       2.778  -4.844  16.816  1.00 95.36           C
ATOM     87  CG  ARG A  14       1.687  -3.964  16.234  1.00 95.51           C
ATOM     88  CD  ARG A  14       1.317  -4.410  14.838  1.00106.41           C
ATOM     89  NE  ARG A  14       2.482  -4.841  14.078  1.00111.87           N
ATOM     90  CZ  ARG A  14       2.444  -5.167  12.793  1.00109.84           C
ATOM     91  NH1 ARG A  14       1.293  -5.098  12.131  1.00 99.48           N
ATOM     92  NH2 ARG A  14       3.555  -5.553  12.174  1.00110.47           N
ATOM     93  N   GLY A  15       1.512  -2.906  19.178  1.00 99.24           N
ATOM     94  CA  GLY A  15       0.328  -2.429  19.842  1.00 88.62           C
ATOM     95  C   GLY A  15      -0.677  -1.725  18.952  1.00 88.60           C
ATOM     96  O   GLY A  15      -0.690  -1.872  17.727  1.00 94.53           O
ATOM     97  N   MET A  16      -1.559  -0.975  19.616  1.00 78.02           N
ATOM     98  CA  MET A  16      -2.632  -0.226  18.986  1.00 79.79           C
ATOM     99  C   MET A  16      -2.754   1.157  19.621  1.00 90.76           C
ATOM    100  O   MET A  16      -2.469   1.363  20.814  1.00 82.39           O
ATOM    101  CB  MET A  16      -3.991  -0.919  19.108  1.00 74.22           C
ATOM    102  CG  MET A  16      -4.020  -2.414  18.998  1.00 88.42           C
ATOM    103  SD  MET A  16      -5.516  -2.945  19.861  1.00100.81           S
ATOM    104  CE  MET A  16      -6.603  -1.541  19.529  1.00 76.56           C
ATOM    105  N   ASN A  17      -3.231   2.090  18.788  1.00 80.91           N
ATOM    106  CA  ASN A  17      -3.457   3.488  19.137  1.00 91.87           C
ATOM    107  C   ASN A  17      -4.948   3.753  19.357  1.00 89.22           C
ATOM    108  O   ASN A  17      -5.769   3.520  18.463  1.00 89.10           O
ATOM    109  CB  ASN A  17      -2.899   4.417  18.058  1.00 30.00           C
ATOM    110  CG  ASN A  17      -1.383   4.455  18.047  1.00 30.00           C
ATOM    111  OD1 ASN A  17      -0.736   4.154  19.050  1.00 30.00           O
ATOM    112  ND2 ASN A  17      -0.808   4.825  16.909  1.00 30.00           N
```

ATOM	113	N	LEU	A	18	-5.311	4.212	20.556	1.00	78.24	N
ATOM	114	CA	LEU	A	18	-6.715	4.414	20.913	1.00	77.90	C
ATOM	115	C	LEU	A	18	-7.032	5.887	21.127	1.00	88.85	C
ATOM	116	O	LEU	A	18	-6.317	6.584	21.854	1.00	91.44	O
ATOM	117	CB	LEU	A	18	-7.105	3.644	22.183	1.00	84.57	C
ATOM	118	CG	LEU	A	18	-6.822	2.144	22.330	1.00	96.00	C
ATOM	119	CD1	LEU	A	18	-5.366	1.878	22.677	1.00	91.46	C
ATOM	120	CD2	LEU	A	18	-7.743	1.559	23.401	1.00	93.92	C
ATOM	121	N	THR	A	19	-8.127	6.351	20.525	1.00	87.68	N
ATOM	122	CA	THR	A	19	-8.638	7.682	20.839	1.00	85.16	C
ATOM	123	C	THR	A	19	-9.338	7.647	22.201	1.00	95.78	C
ATOM	124	O	THR	A	19	-10.320	6.916	22.390	1.00	98.05	O
ATOM	125	CB	THR	A	19	-9.593	8.177	19.748	1.00	85.80	C
ATOM	126	OG1	THR	A	19	-10.450	7.108	19.320	1.00	94.24	O
ATOM	127	CG2	THR	A	19	-8.811	8.709	18.547	1.00	83.58	C
ATOM	128	N	VAL	A	20	-8.808	8.403	23.166	1.00	95.84	N
ATOM	129	CA	VAL	A	20	-9.428	8.522	24.484	1.00	85.07	C
ATOM	130	C	VAL	A	20	-9.473	9.991	24.879	1.00	81.91	C
ATOM	131	O	VAL	A	20	-8.435	10.573	25.218	1.00	77.53	O
ATOM	132	CB	VAL	A	20	-8.673	7.703	25.548	1.00	81.78	C
ATOM	133	CG1	VAL	A	20	-9.423	7.748	26.863	1.00	73.26	C
ATOM	134	CG2	VAL	A	20	-8.475	6.276	25.096	1.00	78.78	C
ATOM	135	N	PHE	A	21	-10.669	10.597	24.825	1.00	80.23	N
ATOM	136	CA	PHE	A	21	-10.894	11.973	25.290	1.00	86.18	C
ATOM	137	C	PHE	A	21	-10.050	12.981	24.520	1.00	90.05	C
ATOM	138	O	PHE	A	21	-9.394	13.847	25.113	1.00	88.76	O
ATOM	139	CB	PHE	A	21	-10.609	12.111	26.786	1.00	83.04	C
ATOM	140	CG	PHE	A	21	-11.451	11.239	27.641	1.00	85.78	C
ATOM	141	CD1	PHE	A	21	-10.925	10.669	28.786	1.00	84.51	C
ATOM	142	CD2	PHE	A	21	-12.771	10.994	27.312	1.00	80.47	C
ATOM	143	CE1	PHE	A	21	-11.697	9.867	29.588	1.00	79.62	C
ATOM	144	CE2	PHE	A	21	-13.547	10.199	28.109	1.00	81.07	C
ATOM	145	CZ	PHE	A	21	-13.012	9.633	29.250	1.00	81.06	C
ATOM	146	N	GLY	A	22	-10.066	12.870	23.193	1.00	77.03	N
ATOM	147	CA	GLY	A	22	-9.215	13.739	22.399	1.00	85.24	C
ATOM	148	C	GLY	A	22	-7.747	13.639	22.751	1.00	87.03	C
ATOM	149	O	GLY	A	22	-7.031	14.647	22.733	1.00	87.50	O
ATOM	150	N	GLY	A	23	-7.293	12.453	23.120	1.00	91.28	N
ATOM	151	CA	GLY	A	23	-5.890	12.162	23.299	1.00	88.43	C
ATOM	152	C	GLY	A	23	-5.697	10.767	22.759	1.00	97.30	C
ATOM	153	O	GLY	A	23	-6.649	10.168	22.245	1.00	99.86	O
ATOM	154	N	THR	A	24	-4.494	10.230	22.831	1.00	94.92	N
ATOM	155	CA	THR	A	24	-4.299	8.857	22.406	1.00	89.74	C
ATOM	156	C	THR	A	24	-3.600	8.102	23.519	1.00	99.56	C

```
ATOM    157  O   THR A  24      -2.861   8.678  24.330  1.00 97.81           O
ATOM    158  CB  THR A  24      -3.499   8.703  21.079  1.00 89.86           C
ATOM    159  OG1 THR A  24      -2.094   8.673  21.351  1.00 88.66           O
ATOM    160  CG2 THR A  24      -3.820   9.819  20.063  1.00 86.07           C
ATOM    161  N   VAL A  25      -3.870   6.800  23.552  1.00102.18           N
ATOM    162  CA  VAL A  25      -3.293   5.885  24.521  1.00 86.86           C
ATOM    163  C   VAL A  25      -2.871   4.633  23.753  1.00 86.99           C
ATOM    164  O   VAL A  25      -3.534   4.244  22.789  1.00 89.27           O
ATOM    165  CB  VAL A  25      -4.308   5.583  25.648  1.00 80.93           C
ATOM    166  CG1 VAL A  25      -5.286   4.478  25.255  1.00 92.62           C
ATOM    167  CG2 VAL A  25      -3.604   5.275  26.964  1.00 80.38           C
ATOM    168  N   THR A  26      -1.730   4.047  24.112  1.00 91.28           N
ATOM    169  CA  THR A  26      -1.214   2.826  23.487  1.00 92.18           C
ATOM    170  C   THR A  26      -1.684   1.664  24.345  1.00 85.48           C
ATOM    171  O   THR A  26      -1.411   1.645  25.553  1.00 79.82           O
ATOM    172  CB  THR A  26       0.324   2.800  23.458  1.00 92.28           C
ATOM    173  OG1 THR A  26       0.770   3.332  24.693  1.00100.39           O
ATOM    174  CG2 THR A  26       1.020   3.615  22.356  1.00 76.59           C
ATOM    175  N   ALA A  27      -2.284   0.657  23.716  1.00 85.75           N
ATOM    176  CA  ALA A  27      -2.623  -0.585  24.397  1.00 74.89           C
ATOM    177  C   ALA A  27      -1.955  -1.750  23.690  1.00 75.04           C
ATOM    178  O   ALA A  27      -1.905  -1.792  22.461  1.00 74.91           O
ATOM    179  CB  ALA A  27      -4.142  -0.839  24.456  1.00 72.62           C
ATOM    180  N   PHE A  28      -1.459  -2.704  24.472  1.00 86.10           N
ATOM    181  CA  PHE A  28      -0.911  -3.967  23.970  1.00 79.65           C
ATOM    182  C   PHE A  28      -1.704  -5.075  24.644  1.00 79.15           C
ATOM    183  O   PHE A  28      -1.386  -5.481  25.764  1.00 85.88           O
ATOM    184  CB  PHE A  28       0.578  -4.059  24.261  1.00 80.89           C
ATOM    185  CG  PHE A  28       1.303  -2.775  23.982  1.00 87.11           C
ATOM    186  CD1 PHE A  28       1.996  -2.122  24.961  1.00 87.45           C
ATOM    187  CD2 PHE A  28       1.287  -2.227  22.734  1.00 88.46           C
ATOM    188  CE1 PHE A  28       2.632  -0.940  24.703  1.00 88.13           C
ATOM    189  CE2 PHE A  28       1.935  -1.057  22.463  1.00 87.87           C
ATOM    190  CZ  PHE A  28       2.616  -0.414  23.450  1.00 87.00           C
ATOM    191  N   LEU A  29      -2.747  -5.554  23.970  1.00 76.82           N
ATOM    192  CA  LEU A  29      -3.671  -6.516  24.558  1.00 72.76           C
ATOM    193  C   LEU A  29      -3.215  -7.938  24.251  1.00 67.69           C
ATOM    194  O   LEU A  29      -2.987  -8.289  23.089  1.00 71.40           O
ATOM    195  CB  LEU A  29      -5.100  -6.269  24.051  1.00 70.76           C
ATOM    196  CG  LEU A  29      -5.601  -4.813  23.990  1.00 63.01           C
ATOM    197  CD1 LEU A  29      -6.993  -4.750  23.368  1.00 49.83           C
ATOM    198  CD2 LEU A  29      -5.583  -4.127  25.371  1.00 56.39           C
ATOM    199  N   GLY A  30      -3.060  -8.743  25.299  1.00 78.92           N
ATOM    200  CA  GLY A  30      -2.801 -10.165  25.134  1.00 73.44           C
```

```
ATOM    201  C   GLY A  30      -1.353 -10.563  24.932  1.00 63.37           C
ATOM    202  O   GLY A  30      -1.067 -11.480  24.157  1.00 67.10           O
ATOM    203  N   ILE A  31      -0.429  -9.900  25.606  1.00 71.62           N
ATOM    204  CA  ILE A  31       0.957 -10.383  25.627  1.00 70.47           C
ATOM    205  C   ILE A  31       1.025 -11.651  26.471  1.00 75.78           C
ATOM    206  O   ILE A  31       0.502 -11.664  27.602  1.00 67.56           O
ATOM    207  CB  ILE A  31       1.884  -9.320  26.203  1.00 72.02           C
ATOM    208  CG1 ILE A  31       1.500  -7.927  25.707  1.00 69.92           C
ATOM    209  CG2 ILE A  31       3.328  -9.677  25.885  1.00 62.02           C
ATOM    210  CD1 ILE A  31       2.242  -6.828  26.426  1.00 76.21           C
ATOM    211  N   PRO A  32       1.658 -12.720  25.990  1.00 76.74           N
ATOM    212  CA  PRO A  32       1.847 -13.914  26.827  1.00 70.71           C
ATOM    213  C   PRO A  32       2.973 -13.729  27.838  1.00 73.57           C
ATOM    214  O   PRO A  32       4.022 -13.158  27.520  1.00 72.43           O
ATOM    215  CB  PRO A  32       2.188 -14.999  25.799  1.00 69.36           C
ATOM    216  CG  PRO A  32       2.796 -14.243  24.645  1.00 61.85           C
ATOM    217  CD  PRO A  32       2.062 -12.946  24.591  1.00 70.64           C
ATOM    218  N   TYR A  33       2.742 -14.204  29.085  1.00 69.77           N
ATOM    219  CA  TYR A  33       3.760 -14.126  30.138  1.00 71.76           C
ATOM    220  C   TYR A  33       4.156 -15.482  30.724  1.00 78.46           C
ATOM    221  O   TYR A  33       5.170 -15.558  31.433  1.00 70.45           O
ATOM    222  CB  TYR A  33       3.313 -13.182  31.276  1.00 54.90           C
ATOM    223  CG  TYR A  33       2.155 -13.674  32.125  1.00 68.07           C
ATOM    224  CD1 TYR A  33       0.837 -13.314  31.835  1.00 62.46           C
ATOM    225  CD2 TYR A  33       2.384 -14.517  33.228  1.00 72.33           C
ATOM    226  CE1 TYR A  33      -0.227 -13.788  32.627  1.00 70.72           C
ATOM    227  CE2 TYR A  33       1.334 -14.996  34.012  1.00 67.16           C
ATOM    228  CZ  TYR A  33       0.034 -14.632  33.710  1.00 63.05           C
ATOM    229  OH  TYR A  33      -0.998 -15.106  34.483  1.00 52.16           O
ATOM    230  N   ALA A  34       3.412 -16.550  30.430  1.00 82.40           N
ATOM    231  CA  ALA A  34       3.680 -17.889  30.947  1.00 76.07           C
ATOM    232  C   ALA A  34       3.317 -18.896  29.866  1.00 72.88           C
ATOM    233  O   ALA A  34       2.393 -18.663  29.080  1.00 78.39           O
ATOM    234  CB  ALA A  34       2.893 -18.174  32.233  1.00 81.04           C
ATOM    235  N   GLN A  35       4.079 -19.992  29.810  0.59 77.41           N
ATOM    237  CA  GLN A  35       3.813 -21.044  28.830  0.59 73.56           C
ATOM    239  C   GLN A  35       2.374 -21.538  28.999  0.59 68.51           C
ATOM    241  O   GLN A  35       1.923 -21.718  30.129  0.59 71.39           O
ATOM    243  CB  GLN A  35       4.767 -22.230  29.001  0.59 76.88           C
ATOM    245  CG  GLN A  35       6.265 -21.939  28.909  0.59 79.71           C
ATOM    247  CD  GLN A  35       7.108 -23.215  28.928  0.59 80.25           C
ATOM    249  OE1 GLN A  35       6.623 -24.303  28.600  0.59 69.88           O
ATOM    251  NE2 GLN A  35       8.368 -23.087  29.342  0.59 83.62           N
ATOM    253  N   PRO A  36       1.648 -21.760  27.899  1.00 66.55           N
```

```
ATOM    254  CA  PRO A  36       0.256 -22.271  28.037  1.00 69.44           C
ATOM    255  C   PRO A  36       0.159 -23.453  28.968  1.00 73.94           C
ATOM    256  O   PRO A  36       0.859 -24.468  28.801  1.00 75.16           O
ATOM    257  CB  PRO A  36      -0.094 -22.670  26.590  1.00 61.61           C
ATOM    258  CG  PRO A  36       0.689 -21.700  25.770  1.00 65.79           C
ATOM    259  CD  PRO A  36       1.998 -21.456  26.518  1.00 72.26           C
ATOM    260  N   PRO A  37      -0.576 -23.353  29.918  1.00 75.69           N
ATOM    261  CA  PRO A  37      -0.626 -24.398  30.956  1.00 78.48           C
ATOM    262  C   PRO A  37      -1.446 -25.608  30.509  1.00 74.79           C
ATOM    263  O   PRO A  37      -2.503 -25.917  31.080  1.00 74.06           O
ATOM    264  CB  PRO A  37      -1.266 -23.659  32.141  1.00 67.81           C
ATOM    265  CG  PRO A  37      -1.991 -22.491  31.558  1.00 60.88           C
ATOM    266  CD  PRO A  37      -1.234 -22.100  30.334  1.00 65.86           C
ATOM    267  N   LEU A  38      -0.956 -26.298  29.465  1.00 65.26           N
ATOM    268  CA  LEU A  38      -1.646 -27.426  28.839  1.00 75.17           C
ATOM    269  C   LEU A  38      -0.843 -28.708  28.726  1.00 76.59           C
ATOM    270  O   LEU A  38       0.389 -28.707  28.625  1.00 82.95           O
ATOM    271  CB  LEU A  38      -2.153 -27.131  27.435  1.00 77.32           C
ATOM    272  CG  LEU A  38      -3.169 -26.029  27.530  1.00 82.35           C
ATOM    273  CD1 LEU A  38      -3.625 -25.682  26.075  1.00 82.73           C
ATOM    274  CD2 LEU A  38      -4.231 -26.578  28.489  1.00 76.24           C
ATOM    275  N   GLY A  39      -1.610 -29.801  28.678  1.00 62.92           N
ATOM    276  CA  GLY A  39      -1.067 -31.118  28.495  1.00 75.21           C
ATOM    277  C   GLY A  39      -0.430 -31.571  29.777  1.00 73.25           C
ATOM    278  O   GLY A  39      -1.100 -31.679  30.810  1.00 69.75           O
ATOM    279  N   ARG A  40       0.876 -31.835  29.700  1.00 81.23           N
ATOM    280  CA  ARG A  40       1.664 -32.132  30.885  1.00 76.50           C
ATOM    281  C   ARG A  40       1.520 -31.040  31.936  1.00 83.79           C
ATOM    282  O   ARG A  40       1.613 -31.321  33.137  1.00 84.01           O
ATOM    283  CB  ARG A  40       3.124 -32.314  30.462  1.00 79.32           C
ATOM    284  CG  ARG A  40       4.167 -32.318  31.556  1.00 87.83           C
ATOM    285  CD  ARG A  40       5.541 -32.170  30.933  0.60 79.83           C
ATOM    286  NE  ARG A  40       5.916 -30.770  30.810  0.69 76.15           N
ATOM    287  CZ  ARG A  40       6.807 -30.313  29.941  0.53 80.11           C
ATOM    288  NH1 ARG A  40       7.393 -31.158  29.102  0.87 74.09           N
ATOM    289  NH2 ARG A  40       7.093 -29.013  29.895  1.00 85.95           N
ATOM    290  N   LEU A  41       1.229 -29.805  31.514  1.00 82.96           N
ATOM    291  CA  LEU A  41       1.420 -28.649  32.377  1.00 77.19           C
ATOM    292  C   LEU A  41       0.175 -28.183  33.133  1.00 73.94           C
ATOM    293  O   LEU A  41       0.284 -27.202  33.876  1.00 74.03           O
ATOM    294  CB  LEU A  41       2.001 -27.470  31.578  1.00 64.70           C
ATOM    295  CG  LEU A  41       3.485 -27.631  31.195  1.00 67.61           C
ATOM    296  CD1 LEU A  41       4.124 -26.340  30.670  1.00 75.31           C
ATOM    297  CD2 LEU A  41       4.254 -28.128  32.398  1.00 79.71           C
```

ATOM	298	N	ARG	A	42	-0.993	-28.825	33.018	1.00	62.87	N
ATOM	299	CA	ARG	A	42	-2.104	-28.300	33.811	1.00	70.86	C
ATOM	300	C	ARG	A	42	-1.971	-28.739	35.262	1.00	74.99	C
ATOM	301	O	ARG	A	42	-1.388	-29.778	35.565	1.00	74.79	O
ATOM	302	CB	ARG	A	42	-3.481	-28.696	33.276	1.00	50.48	C
ATOM	303	CG	ARG	A	42	-3.632	-30.080	32.986	1.00	78.46	C
ATOM	304	CD	ARG	A	42	-4.755	-30.283	31.979	1.00	97.70	C
ATOM	305	NE	ARG	A	42	-6.010	-30.720	32.575	1.00	89.66	N
ATOM	306	CZ	ARG	A	42	-7.135	-30.867	31.883	1.00	78.58	C
ATOM	307	NH1	ARG	A	42	-7.171	-30.578	30.594	1.00	83.79	N
ATOM	308	NH2	ARG	A	42	-8.219	-31.289	32.480	1.00	89.43	N
ATOM	309	N	PHE	A	43	-2.504	-27.912	36.158	1.00	73.99	N
ATOM	310	CA	PHE	A	43	-2.415	-28.054	37.607	1.00	63.31	C
ATOM	311	C	PHE	A	43	-1.023	-27.736	38.144	1.00	66.62	C
ATOM	312	O	PHE	A	43	-0.837	-27.665	39.364	1.00	67.05	O
ATOM	313	CB	PHE	A	43	-2.823	-29.447	38.067	1.00	70.09	C
ATOM	314	CG	PHE	A	43	-4.193	-29.870	37.622	1.00	71.68	C
ATOM	315	CD1	PHE	A	43	-5.319	-29.170	38.029	1.00	67.13	C
ATOM	316	CD2	PHE	A	43	-4.367	-31.022	36.857	1.00	76.40	C
ATOM	317	CE1	PHE	A	43	-6.607	-29.582	37.637	1.00	67.96	C
ATOM	318	CE2	PHE	A	43	-5.648	-31.444	36.469	1.00	74.77	C
ATOM	319	CZ	PHE	A	43	-6.771	-30.723	36.859	1.00	58.05	C
ATOM	320	N	LYS	A	44	-0.037	-27.546	37.269	1.00	64.98	N
ATOM	321	CA	LYS	A	44	1.278	-27.159	37.762	1.00	68.24	C
ATOM	322	C	LYS	A	44	1.438	-25.643	37.785	1.00	67.90	C
ATOM	323	O	LYS	A	44	0.601	-24.889	37.286	1.00	62.43	O
ATOM	324	CB	LYS	A	44	2.396	-27.774	36.927	1.00	57.92	C
ATOM	325	CG	LYS	A	44	2.269	-29.239	36.731	1.00	71.31	C
ATOM	326	CD	LYS	A	44	3.537	-29.737	36.103	1.00	82.76	C
ATOM	327	CE	LYS	A	44	3.394	-31.163	35.647	1.00	95.29	C
ATOM	328	NZ	LYS	A	44	4.351	-31.397	34.539	1.00	96.94	N
ATOM	329	N	LYS	A	45	2.546	-25.204	38.376	1.00	66.20	N
ATOM	330	CA	LYS	A	45	2.854	-23.788	38.434	1.00	62.95	C
ATOM	331	C	LYS	A	45	3.274	-23.265	37.056	1.00	61.78	C
ATOM	332	O	LYS	A	45	3.912	-23.979	36.282	1.00	61.39	O
ATOM	333	CB	LYS	A	45	3.949	-23.555	39.466	1.00	66.30	C
ATOM	334	CG	LYS	A	45	3.475	-23.838	40.885	1.00	55.75	C
ATOM	335	CD	LYS	A	45	4.636	-23.970	41.853	1.00	68.17	C
ATOM	336	CE	LYS	A	45	5.305	-22.648	42.165	1.00	66.71	C
ATOM	337	NZ	LYS	A	45	6.348	-22.808	43.223	1.00	76.06	N
ATOM	338	N	PRO	A	46	2.937	-22.015	36.732	1.00	62.09	N
ATOM	339	CA	PRO	A	46	3.216	-21.507	35.388	1.00	64.86	C
ATOM	340	C	PRO	A	46	4.706	-21.487	35.094	1.00	81.02	C
ATOM	341	O	PRO	A	46	5.521	-21.094	35.935	1.00	76.72	O

```
ATOM    342  CB  PRO A  46       2.648 -20.090  35.428  1.00 59.07           C
ATOM    343  CG  PRO A  46       2.851 -19.686  36.824  1.00 60.35           C
ATOM    344  CD  PRO A  46       2.529 -20.921  37.630  1.00 65.49           C
ATOM    345  N   GLN A  47       5.052 -21.899  33.873  1.00 85.76           N
ATOM    346  CA  GLN A  47       6.436 -21.983  33.428  1.00 88.00           C
ATOM    347  C   GLN A  47       6.774 -20.782  32.551  1.00 83.06           C
ATOM    348  O   GLN A  47       5.994 -20.420  31.666  1.00 83.40           O
ATOM    349  CB  GLN A  47       6.677 -23.302  32.688  1.00 83.42           C
ATOM    350  CG  GLN A  47       6.646 -24.503  33.634  1.00 79.94           C
ATOM    351  CD  GLN A  47       7.962 -24.671  34.371  1.00 82.83           C
ATOM    352  OE1 GLN A  47       8.926 -23.946  34.120  1.00 92.49           O
ATOM    353  NE2 GLN A  47       8.000 -25.608  35.304  1.00 78.78           N
ATOM    354  N   SER A  48       7.930 -20.167  32.808  1.00 92.14           N
ATOM    355  CA  SER A  48       8.327 -18.937  32.127  1.00 98.21           C
ATOM    356  C   SER A  48       8.572 -19.186  30.629  1.00 91.80           C
ATOM    357  O   SER A  48       8.808 -20.318  30.189  1.00 77.71           O
ATOM    358  CB  SER A  48       9.572 -18.352  32.811  1.00 98.25           C
ATOM    359  OG  SER A  48      10.133 -19.259  33.766  1.00 89.25           O
ATOM    360  N   LEU A  49       8.520 -18.102  29.842  1.00 99.47           N
ATOM    361  CA  LEU A  49       8.594 -18.173  28.377  1.00107.29           C
ATOM    362  C   LEU A  49      10.010 -18.204  27.826  1.00101.59           C
ATOM    363  O   LEU A  49      10.983 -17.758  28.443  1.00 90.57           O
ATOM    364  CB  LEU A  49       7.910 -16.990  27.673  1.00 97.16           C
ATOM    365  CG  LEU A  49       6.443 -17.067  27.295  1.00 87.41           C
ATOM    366  CD1 LEU A  49       5.795 -17.744  28.389  1.00 88.57           C
ATOM    367  CD2 LEU A  49       5.837 -15.668  27.065  1.00 81.45           C
ATOM    368  N   THR A  50      10.070 -18.699  26.598  1.00 97.06           N
ATOM    369  CA  THR A  50      11.198 -18.644  25.694  1.00100.01           C
ATOM    370  C   THR A  50      10.846 -17.704  24.546  1.00108.52           C
ATOM    371  O   THR A  50       9.685 -17.617  24.135  1.00120.22           O
ATOM    372  CB  THR A  50      11.498 -20.046  25.170  1.00108.91           C
ATOM    373  OG1 THR A  50      12.400 -19.967  24.066  1.00111.41           O
ATOM    374  CG2 THR A  50      10.198 -20.735  24.725  1.00101.76           C
ATOM    375  N   LYS A  51      11.852 -17.000  24.029  1.00103.82           N
ATOM    376  CA  LYS A  51      11.618 -15.873  23.133  1.00 98.49           C
ATOM    377  C   LYS A  51      10.852 -16.287  21.869  1.00109.07           C
ATOM    378  O   LYS A  51      10.737 -17.468  21.522  1.00106.80           O
ATOM    379  CB  LYS A  51      12.954 -15.205  22.781  0.00101.53           C
ATOM    380  CG  LYS A  51      14.129 -16.173  22.539  0.00 98.94           C
ATOM    381  CD  LYS A  51      13.855 -17.128  21.383  0.00 96.80           C
ATOM    382  CE  LYS A  51      14.955 -18.147  21.127  0.00 91.59           C
ATOM    383  NZ  LYS A  51      14.458 -19.148  20.131  0.00 87.89           N
ATOM    384  N   TRP A  52      10.298 -15.278  21.198  1.00112.51           N
ATOM    385  CA  TRP A  52       9.818 -15.364  19.824  1.00109.42           C
```

```
ATOM    386  C   TRP A  52      10.468 -14.212  19.065  1.00117.58           C
ATOM    387  O   TRP A  52      10.871 -13.213  19.671  1.00112.53           O
ATOM    388  CB  TRP A  52       8.283 -15.284  19.751  1.00103.41           C
ATOM    389  CG  TRP A  52       7.763 -13.948  20.165  1.00 97.15           C
ATOM    390  CD1 TRP A  52       7.592 -12.853  19.367  1.00106.43           C
ATOM    391  CD2 TRP A  52       7.381 -13.544  21.483  1.00101.61           C
ATOM    392  NE1 TRP A  52       7.115 -11.796  20.102  1.00109.78           N
ATOM    393  CE2 TRP A  52       6.976 -12.195  21.406  1.00108.16           C
ATOM    394  CE3 TRP A  52       7.333 -14.191  22.719  1.00100.91           C
ATOM    395  CZ2 TRP A  52       6.530 -11.486  22.518  1.00 96.48           C
ATOM    396  CZ3 TRP A  52       6.889 -13.479  23.823  1.00 93.00           C
ATOM    397  CH2 TRP A  52       6.496 -12.144  23.714  1.00 91.86           C
ATOM    398  N   SER A  53      10.600 -14.345  17.738  1.00122.59           N
ATOM    399  CA  SER A  53      11.445 -13.389  16.986  1.00116.21           C
ATOM    400  C   SER A  53      11.252 -13.065  15.493  1.00115.83           C
ATOM    401  O   SER A  53      12.276 -12.754  14.879  1.00123.32           O
ATOM    402  CB  SER A  53      12.896 -13.848  17.147  1.00 30.00           C
ATOM    403  OG  SER A  53      13.129 -15.057  16.447  1.00 30.00           O
ATOM    404  N   ASP A  54      10.082 -13.067  14.841  1.00116.90           N
ATOM    405  CA  ASP A  54       8.663 -12.814  15.223  1.00110.08           C
ATOM    406  C   ASP A  54       8.257 -11.564  16.050  1.00 99.72           C
ATOM    407  O   ASP A  54       8.993 -11.129  16.926  1.00 95.09           O
ATOM    408  CB  ASP A  54       8.034 -14.031  15.904  1.00107.02           C
ATOM    409  CG  ASP A  54       6.531 -14.101  15.618  1.00112.51           C
ATOM    410  OD1 ASP A  54       5.726 -13.977  16.570  1.00104.45           O
ATOM    411  OD2 ASP A  54       6.152 -14.190  14.425  1.00116.08           O
ATOM    412  N   ILE A  55       7.090 -11.030  15.703  1.00 93.60           N
ATOM    413  CA  ILE A  55       6.449  -9.934  16.402  1.00103.35           C
ATOM    414  C   ILE A  55       5.103 -10.536  16.829  1.00108.45           C
ATOM    415  O   ILE A  55       4.396 -11.171  16.012  1.00102.06           O
ATOM    416  CB  ILE A  55       6.323  -8.667  15.510  1.00105.04           C
ATOM    417  CG1 ILE A  55       5.620  -8.771  14.131  1.00123.10           C
ATOM    418  CG2 ILE A  55       7.599  -8.629  14.653  1.00115.41           C
ATOM    419  CD1 ILE A  55       4.165  -8.670  14.077  1.00107.06           C
ATOM    420  N   TRP A  56       4.817 -10.454  18.136  1.00100.69           N
ATOM    421  CA  TRP A  56       3.520 -10.779  18.708  1.00 96.59           C
ATOM    422  C   TRP A  56       2.635  -9.545  18.537  1.00 91.54           C
ATOM    423  O   TRP A  56       2.940  -8.485  19.095  1.00 90.23           O
ATOM    424  CB  TRP A  56       3.727 -11.149  20.176  1.00 90.04           C
ATOM    425  CG  TRP A  56       2.567 -11.615  20.736  1.00 82.93           C
ATOM    426  CD1 TRP A  56       1.536 -10.881  21.179  1.00 81.72           C
ATOM    427  CD2 TRP A  56       2.208 -12.973  20.862  1.00 83.00           C
ATOM    428  NE1 TRP A  56       0.536 -11.702  21.609  1.00 87.69           N
ATOM    429  CE2 TRP A  56       0.927 -13.002  21.422  1.00 84.55           C
```

```
ATOM    430  CE3 TRP A  56       2.850 -14.177  20.559  1.00 78.94           C
ATOM    431  CZ2 TRP A  56       0.266 -14.183  21.697  1.00 84.49           C
ATOM    432  CZ3 TRP A  56       2.196 -15.352  20.830  1.00 79.73           C
ATOM    433  CH2 TRP A  56       0.912 -15.350  21.391  1.00 82.51           C
ATOM    434  N   ASN A  57       1.579  -9.648  17.728  1.00 87.63           N
ATOM    435  CA  ASN A  57       0.653  -8.524  17.572  1.00 89.85           C
ATOM    436  C   ASN A  57      -0.265  -8.542  18.784  1.00 86.78           C
ATOM    437  O   ASN A  57      -1.142  -9.398  18.900  1.00 91.42           O
ATOM    438  CB  ASN A  57      -0.113  -8.602  16.250  1.00 91.36           C
ATOM    439  CG  ASN A  57       0.772  -8.283  15.062  1.00 99.64           C
ATOM    440  OD1 ASN A  57       1.785  -7.616  15.232  1.00100.29           O
ATOM    441  ND2 ASN A  57       0.417  -8.772  13.867  1.00 99.34           N
ATOM    442  N   ALA A  58       0.010  -7.658  19.746  1.00 87.22           N
ATOM    443  CA  ALA A  58      -0.835  -7.514  20.932  1.00 84.92           C
ATOM    444  C   ALA A  58      -1.982  -6.559  20.604  1.00 78.65           C
ATOM    445  O   ALA A  58      -2.104  -5.451  21.127  1.00 76.85           O
ATOM    446  CB  ALA A  58      -0.022  -7.028  22.122  1.00 75.43           C
ATOM    447  N   THR A  59      -2.835  -7.020  19.707  1.00 70.59           N
ATOM    448  CA  THR A  59      -3.855  -6.169  19.143  1.00 73.30           C
ATOM    449  C   THR A  59      -5.262  -6.650  19.454  1.00 76.36           C
ATOM    450  O   THR A  59      -6.227  -6.007  19.018  1.00 68.83           O
ATOM    451  CB  THR A  59      -3.647  -6.050  17.626  1.00 83.31           C
ATOM    452  OG1 THR A  59      -4.402  -4.942  17.128  1.00 99.43           O
ATOM    453  CG2 THR A  59      -4.077  -7.323  16.904  1.00 70.23           C
ATOM    454  N   LYS A  60      -5.413  -7.745  20.203  1.00 71.73           N
ATOM    455  CA  LYS A  60      -6.726  -8.186  20.657  1.00 71.10           C
ATOM    456  C   LYS A  60      -6.606  -8.795  22.046  1.00 69.95           C
ATOM    457  O   LYS A  60      -5.577  -9.386  22.391  1.00 69.03           O
ATOM    458  CB  LYS A  60      -7.356  -9.196  19.682  1.00 75.47           C
ATOM    459  CG  LYS A  60      -8.823  -9.531  19.980  1.00 85.46           C
ATOM    460  CD  LYS A  60      -9.066 -11.046  20.050  1.00 89.79           C
ATOM    461  CE  LYS A  60     -10.411 -11.393  20.715  1.00 78.33           C
ATOM    462  NZ  LYS A  60     -10.482 -12.839  21.142  1.00 83.78           N
ATOM    463  N   TYR A  61      -7.664  -8.634  22.838  1.00 74.64           N
ATOM    464  CA  TYR A  61      -7.786  -9.357  24.102  1.00 72.19           C
ATOM    465  C   TYR A  61      -7.671 -10.862  23.881  1.00 57.15           C
ATOM    466  O   TYR A  61      -8.407 -11.432  23.077  1.00 52.89           O
ATOM    467  CB  TYR A  61      -9.135  -9.039  24.758  1.00 68.28           C
ATOM    468  CG  TYR A  61      -9.266  -7.677  25.450  1.00 70.11           C
ATOM    469  CD1 TYR A  61      -8.350  -7.264  26.417  1.00 62.71           C
ATOM    470  CD2 TYR A  61     -10.363  -6.853  25.199  1.00 59.31           C
ATOM    471  CE1 TYR A  61      -8.501  -6.051  27.070  1.00 63.95           C
ATOM    472  CE2 TYR A  61     -10.517  -5.647  25.850  1.00 53.96           C
ATOM    473  CZ  TYR A  61      -9.587  -5.250  26.783  1.00 61.98           C
```

ATOM	474	OH	TYR	A	61	-9.748	-4.052	27.444	1.00	59.98	O
ATOM	475	N	ALA	A	62	-6.773	-11.502	24.630	1.00	60.67	N
ATOM	476	CA	ALA	A	62	-6.520	-12.942	24.537	1.00	60.05	C
ATOM	477	C	ALA	A	62	-7.691	-13.740	25.121	1.00	53.69	C
ATOM	478	O	ALA	A	62	-8.703	-13.181	25.543	1.00	65.16	O
ATOM	479	CB	ALA	A	62	-5.211	-13.292	25.240	1.00	60.43	C
ATOM	480	N	ASN	A	63	-7.545	-15.069	25.181	1.00	54.89	N
ATOM	481	CA	ASN	A	63	-8.610	-15.928	25.697	1.00	63.00	C
ATOM	482	C	ASN	A	63	-8.727	-15.846	27.214	1.00	61.31	C
ATOM	483	O	ASN	A	63	-7.721	-15.801	27.925	1.00	64.54	O
ATOM	484	CB	ASN	A	63	-8.442	-17.387	25.250	1.00	73.77	C
ATOM	485	CG	ASN	A	63	-8.040	-17.518	23.780	1.00	69.16	C
ATOM	486	OD1	ASN	A	63	-8.848	-17.257	22.883	1.00	77.59	O
ATOM	487	ND2	ASN	A	63	-6.813	-17.958	23.531	1.00	77.46	N
ATOM	488	N	SER	A	64	-9.970	-15.796	27.707	1.00	60.97	N
ATOM	489	CA	SER	A	64	-10.214	-15.948	29.141	1.00	63.57	C
ATOM	490	C	SER	A	64	-9.952	-17.390	29.555	1.00	71.82	C
ATOM	491	O	SER	A	64	-10.250	-18.325	28.805	1.00	62.12	O
ATOM	492	CB	SER	A	64	-11.658	-15.591	29.497	1.00	61.48	C
ATOM	493	OG	SER	A	64	-12.156	-14.540	28.748	1.00	72.12	O
ATOM	494	N	CYS	A	65	-9.394	-17.570	30.760	1.00	68.19	N
ATOM	495	CA	CYS	A	65	-9.170	-18.918	31.274	1.00	58.46	C
ATOM	496	C	CYS	A	65	-10.493	-19.644	31.472	1.00	58.59	C
ATOM	497	O	CYS	A	65	-11.532	-19.023	31.741	1.00	52.59	O
ATOM	498	CB	CYS	A	65	-8.414	-18.896	32.601	1.00	60.71	C
ATOM	499	SG	CYS	A	65	-6.763	-18.191	32.547	1.00	60.60	S
ATOM	500	N	CYS	A	66	-10.429	-20.977	31.350	1.00	51.95	N
ATOM	501	CA	CYS	A	66	-11.602	-21.827	31.494	1.00	55.10	C
ATOM	502	C	CYS	A	66	-12.291	-21.609	32.841	1.00	46.38	C
ATOM	503	O	CYS	A	66	-11.646	-21.586	33.896	1.00	45.09	O
ATOM	504	CB	CYS	A	66	-11.195	-23.294	31.331	1.00	58.97	C
ATOM	505	SG	CYS	A	66	-10.928	-23.818	29.611	1.00	67.87	S
ATOM	506	N	GLN	A	67	-13.615	-21.474	32.797	1.00	43.81	N
ATOM	507	CA	GLN	A	67	-14.413	-21.101	33.952	1.00	50.15	C
ATOM	508	C	GLN	A	67	-15.877	-21.328	33.622	1.00	48.85	C
ATOM	509	O	GLN	A	67	-16.266	-21.262	32.456	1.00	55.08	O
ATOM	510	CB	GLN	A	67	-14.183	-19.630	34.309	1.00	58.43	C
ATOM	511	CG	GLN	A	67	-14.570	-18.695	33.180	1.00	52.33	C
ATOM	512	CD	GLN	A	67	-14.172	-17.262	33.443	1.00	52.35	C
ATOM	513	OE1	GLN	A	67	-14.960	-16.470	33.984	1.00	55.89	O
ATOM	514	NE2	GLN	A	67	-12.957	-16.902	33.028	1.00	50.02	N
ATOM	515	N	ASN	A	68	-16.689	-21.567	34.647	1.00	51.49	N
ATOM	516	CA	ASN	A	68	-18.126	-21.622	34.418	1.00	61.36	C
ATOM	517	C	ASN	A	68	-18.652	-20.227	34.089	1.00	47.95	C

ATOM	518	O	ASN	A	68	-17.952	-19.229	34.219	1.00	54.67	O
ATOM	519	CB	ASN	A	68	-18.845	-22.206	35.638	1.00	70.71	C
ATOM	520	CG	ASN	A	68	-18.083	-23.368	36.251	1.00	74.45	C
ATOM	521	OD1	ASN	A	68	-17.034	-23.175	36.888	1.00	80.59	O
ATOM	522	ND2	ASN	A	68	-18.598	-24.586	36.054	1.00	76.28	N
ATOM	523	N	ILE	A	69	-19.898	-20.164	33.632	1.00	51.07	N
ATOM	524	CA	ILE	A	69	-20.532	-18.906	33.262	1.00	59.67	C
ATOM	525	C	ILE	A	69	-21.882	-18.833	33.958	1.00	59.08	C
ATOM	526	O	ILE	A	69	-22.589	-19.838	34.052	1.00	84.21	O
ATOM	527	CB	ILE	A	69	-20.674	-18.760	31.724	1.00	57.80	C
ATOM	528	CG1	ILE	A	69	-19.304	-18.557	31.069	1.00	49.00	C
ATOM	529	CG2	ILE	A	69	-21.563	-17.585	31.353	1.00	63.61	C
ATOM	530	CD1	ILE	A	69	-18.621	-19.837	30.726	1.00	63.43	C
ATOM	531	N	ASP	A	70	-22.238	-17.651	34.456	1.00	64.02	N
ATOM	532	CA	ASP	A	70	-23.474	-17.457	35.214	1.00	67.21	C
ATOM	533	C	ASP	A	70	-24.629	-17.113	34.288	1.00	67.36	C
ATOM	534	O	ASP	A	70	-24.768	-15.965	33.855	1.00	79.33	O
ATOM	535	CB	ASP	A	70	-23.321	-16.341	36.233	1.00	66.23	C
ATOM	536	CG	ASP	A	70	-24.673	-15.883	36.789	1.00	74.75	C
ATOM	537	OD1	ASP	A	70	-24.773	-14.668	37.092	1.00	71.41	O
ATOM	538	OD2	ASP	A	70	-25.613	-16.721	36.937	1.00	65.21	O
ATOM	539	N	GLN	A	71	-25.502	-18.090	34.039	1.00	58.84	N
ATOM	540	CA	GLN	A	71	-26.772	-17.876	33.349	1.00	68.67	C
ATOM	541	C	GLN	A	71	-27.998	-17.962	34.258	1.00	63.07	C
ATOM	542	O	GLN	A	71	-29.072	-18.335	33.784	1.00	66.37	O
ATOM	543	CB	GLN	A	71	-26.980	-18.861	32.195	1.00	65.82	C
ATOM	544	CG	GLN	A	71	-26.077	-18.751	30.989	1.00	81.44	C
ATOM	545	CD	GLN	A	71	-24.689	-19.249	31.234	1.00	90.17	C
ATOM	546	OE1	GLN	A	71	-24.008	-18.760	32.115	0.49	96.27	O
ATOM	547	NE2	GLN	A	71	-24.255	-20.233	30.443	0.78	70.53	N
ATOM	548	N	SER	A	72	-27.897	-17.634	35.542	1.00	61.90	N
ATOM	549	CA	SER	A	72	-29.133	-17.605	36.314	1.00	64.93	C
ATOM	550	C	SER	A	72	-30.177	-16.724	35.633	1.00	73.06	C
ATOM	551	O	SER	A	72	-31.382	-16.999	35.718	1.00	72.13	O
ATOM	552	CB	SER	A	72	-28.842	-17.129	37.727	1.00	57.39	C
ATOM	553	OG	SER	A	72	-27.809	-17.926	38.262	1.00	65.23	O
ATOM	554	N	PHE	A	73	-29.725	-15.707	34.895	1.00	73.75	N
ATOM	555	CA	PHE	A	73	-30.599	-14.687	34.319	1.00	70.24	C
ATOM	556	C	PHE	A	73	-30.108	-14.406	32.911	1.00	67.94	C
ATOM	557	O	PHE	A	73	-29.288	-13.505	32.702	1.00	69.67	O
ATOM	558	CB	PHE	A	73	-30.597	-13.402	35.153	1.00	72.08	C
ATOM	559	CG	PHE	A	73	-30.822	-13.617	36.631	1.00	62.97	C
ATOM	560	CD1	PHE	A	73	-32.070	-13.997	37.109	1.00	62.96	C
ATOM	561	CD2	PHE	A	73	-29.797	-13.411	37.535	1.00	51.72	C

ATOM	562	CE1	PHE	A	73	-32.283	-14.177	38.460	1.00	65.32	C
ATOM	563	CE2	PHE	A	73	-30.010	-13.585	38.864	1.00	54.18	C
ATOM	564	CZ	PHE	A	73	-31.246	-13.977	39.344	1.00	58.05	C
ATOM	565	N	PRO	A	74	-30.582	-15.165	31.915	1.00	76.23	N
ATOM	566	CA	PRO	A	74	-30.073	-15.010	30.540	1.00	67.95	C
ATOM	567	C	PRO	A	74	-30.672	-13.791	29.855	1.00	66.34	C
ATOM	568	O	PRO	A	74	-31.891	-13.616	29.828	1.00	70.06	O
ATOM	569	CB	PRO	A	74	-30.520	-16.302	29.843	1.00	65.30	C
ATOM	570	CG	PRO	A	74	-30.932	-17.217	30.907	1.00	66.94	C
ATOM	571	CD	PRO	A	74	-31.430	-16.359	32.035	1.00	71.87	C
ATOM	572	N	GLY	A	75	-29.812	-12.964	29.268	1.00	64.57	N
ATOM	573	CA	GLY	A	75	-30.247	-11.736	28.647	1.00	66.82	C
ATOM	574	C	GLY	A	75	-30.332	-10.549	29.583	1.00	68.86	C
ATOM	575	O	GLY	A	75	-30.622	-9.438	29.124	1.00	71.47	O
ATOM	576	N	PHE	A	76	-30.092	-10.746	30.876	1.00	75.88	N
ATOM	577	CA	PHE	A	76	-30.087	-9.663	31.853	1.00	66.10	C
ATOM	578	C	PHE	A	76	-28.688	-9.076	31.928	1.00	62.08	C
ATOM	579	O	PHE	A	76	-27.741	-9.764	32.327	1.00	61.82	O
ATOM	580	CB	PHE	A	76	-30.531	-10.161	33.227	1.00	60.82	C
ATOM	581	CG	PHE	A	76	-30.447	-9.118	34.292	1.00	65.87	C
ATOM	582	CD1	PHE	A	76	-31.026	-7.879	34.108	1.00	72.49	C
ATOM	583	CD2	PHE	A	76	-29.767	-9.366	35.476	1.00	66.35	C
ATOM	584	CE1	PHE	A	76	-30.941	-6.901	35.094	1.00	73.64	C
ATOM	585	CE2	PHE	A	76	-29.665	-8.389	36.454	1.00	61.72	C
ATOM	586	CZ	PHE	A	76	-30.266	-7.159	36.264	1.00	63.44	C
ATOM	587	N	HIS	A	77	-28.564	-7.809	31.535	1.00	68.30	N
ATOM	588	CA	HIS	A	77	-27.261	-7.152	31.520	1.00	70.31	C
ATOM	589	C	HIS	A	77	-26.573	-7.266	32.876	1.00	65.46	C
ATOM	590	O	HIS	A	77	-25.359	-7.498	32.950	1.00	60.90	O
ATOM	591	CB	HIS	A	77	-27.436	-5.685	31.096	1.00	78.76	C
ATOM	592	CG	HIS	A	77	-26.149	-4.936	30.953	1.00	70.16	C
ATOM	593	ND1	HIS	A	77	-25.093	-5.416	30.210	1.00	62.80	N
ATOM	594	CD2	HIS	A	77	-25.752	-3.736	31.437	1.00	70.44	C
ATOM	595	CE1	HIS	A	77	-24.092	-4.555	30.258	1.00	65.52	C
ATOM	596	NE2	HIS	A	77	-24.466	-3.526	30.997	1.00	64.46	N
ATOM	597	N	GLY	A	78	-27.349	-7.172	33.958	1.00	63.65	N
ATOM	598	CA	GLY	A	78	-26.768	-7.029	35.282	1.00	64.98	C
ATOM	599	C	GLY	A	78	-25.865	-8.173	35.687	1.00	63.43	C
ATOM	600	O	GLY	A	78	-24.949	-7.987	36.492	1.00	63.17	O
ATOM	601	N	SER	A	79	-26.100	-9.364	35.134	1.00	71.03	N
ATOM	602	CA	SER	A	79	-25.276	-10.530	35.419	1.00	63.90	C
ATOM	603	C	SER	A	79	-24.345	-10.903	34.278	1.00	60.79	C
ATOM	604	O	SER	A	79	-23.202	-11.290	34.532	1.00	63.94	O
ATOM	605	CB	SER	A	79	-26.162	-11.729	35.776	1.00	51.85	C

```
ATOM    606  OG  SER A  79     -27.072 -12.006  34.734  1.00 57.31           O
ATOM    607  N   GLU A  80     -24.781 -10.753  33.030  1.00 61.52           N
ATOM    608  CA  GLU A  80     -23.934 -11.135  31.903  1.00 62.10           C
ATOM    609  C   GLU A  80     -22.774 -10.166  31.688  1.00 57.79           C
ATOM    610  O   GLU A  80     -21.791 -10.539  31.045  1.00 45.71           O
ATOM    611  CB  GLU A  80     -24.791 -11.250  30.646  1.00 56.14           C
ATOM    612  CG  GLU A  80     -25.708 -12.459  30.672  1.00 62.83           C
ATOM    613  CD  GLU A  80     -26.722 -12.473  29.543  1.00 72.25           C
ATOM    614  OE1 GLU A  80     -27.461 -13.477  29.432  1.00 74.57           O
ATOM    615  OE2 GLU A  80     -26.787 -11.488  28.772  1.00 79.45           O
ATOM    616  N   MET A  81     -22.858  -8.944  32.230  1.00 63.14           N
ATOM    617  CA  MET A  81     -21.753  -7.990  32.147  1.00 63.04           C
ATOM    618  C   MET A  81     -20.504  -8.468  32.873  1.00 67.77           C
ATOM    619  O   MET A  81     -19.436  -7.870  32.685  1.00 65.03           O
ATOM    620  CB  MET A  81     -22.161  -6.626  32.717  1.00 59.58           C
ATOM    621  CG  MET A  81     -22.533  -6.642  34.195  1.00 62.43           C
ATOM    622  SD  MET A  81     -22.849  -4.973  34.885  1.00 58.67           S
ATOM    623  CE  MET A  81     -21.134  -4.419  35.114  1.00 50.64           C
ATOM    624  N   TRP A  82     -20.617  -9.511  33.707  1.00 71.96           N
ATOM    625  CA  TRP A  82     -19.479 -10.103  34.397  1.00 60.45           C
ATOM    626  C   TRP A  82     -19.005 -11.395  33.748  1.00 58.71           C
ATOM    627  O   TRP A  82     -17.877 -11.825  34.015  1.00 59.68           O
ATOM    628  CB  TRP A  82     -19.840 -10.379  35.861  1.00 55.26           C
ATOM    629  CG  TRP A  82     -20.385  -9.171  36.562  1.00 49.70           C
ATOM    630  CD1 TRP A  82     -21.693  -8.927  36.884  1.00 50.20           C
ATOM    631  CD2 TRP A  82     -19.640  -8.025  37.006  1.00 54.29           C
ATOM    632  NE1 TRP A  82     -21.804  -7.701  37.503  1.00 64.13           N
ATOM    633  CE2 TRP A  82     -20.561  -7.128  37.589  1.00 60.30           C
ATOM    634  CE3 TRP A  82     -18.284  -7.667  36.956  1.00 58.82           C
ATOM    635  CZ2 TRP A  82     -20.169  -5.897  38.123  1.00 55.83           C
ATOM    636  CZ3 TRP A  82     -17.892  -6.449  37.489  1.00 55.41           C
ATOM    637  CH2 TRP A  82     -18.835  -5.579  38.067  1.00 57.44           C
ATOM    638  N   ASN A  83     -19.840 -12.024  32.918  1.00 58.67           N
ATOM    639  CA  ASN A  83     -19.430 -13.209  32.178  1.00 56.63           C
ATOM    640  C   ASN A  83     -18.344 -12.828  31.170  1.00 58.97           C
ATOM    641  O   ASN A  83     -18.319 -11.695  30.671  1.00 60.74           O
ATOM    642  CB  ASN A  83     -20.618 -13.833  31.441  1.00 53.94           C
ATOM    643  CG  ASN A  83     -21.597 -14.520  32.371  1.00 57.23           C
ATOM    644  OD1 ASN A  83     -21.225 -15.037  33.432  1.00 52.84           O
ATOM    645  ND2 ASN A  83     -22.866 -14.535  31.970  1.00 58.49           N
ATOM    646  N   PRO A  84     -17.448 -13.755  30.830  1.00 51.84           N
ATOM    647  CA  PRO A  84     -16.347 -13.399  29.931  1.00 57.84           C
ATOM    648  C   PRO A  84     -16.874 -12.951  28.570  1.00 68.06           C
ATOM    649  O   PRO A  84     -18.009 -13.235  28.177  1.00 70.26           O
```

ATOM	650	CB	PRO	A	84	-15.533	-14.698	29.827	1.00	51.24	C
ATOM	651	CG	PRO	A	84	-16.049	-15.579	30.936	1.00	61.73	C
ATOM	652	CD	PRO	A	84	-17.473	-15.196	31.116	1.00	59.67	C
ATOM	653	N	ASN	A	85	-16.032	-12.207	27.852	1.00	71.03	N
ATOM	654	CA	ASN	A	85	-16.349	-11.709	26.515	1.00	71.82	C
ATOM	655	C	ASN	A	85	-15.293	-12.130	25.506	1.00	66.80	C
ATOM	656	O	ASN	A	85	-15.037	-11.423	24.528	1.00	66.32	O
ATOM	657	CB	ASN	A	85	-16.481	-10.193	26.530	1.00	61.33	C
ATOM	658	CG	ASN	A	85	-15.222	-9.526	27.026	1.00	64.47	C
ATOM	659	OD1	ASN	A	85	-14.347	-10.185	27.593	1.00	77.53	O
ATOM	660	ND2	ASN	A	85	-15.132	-8.217	26.854	1.00	65.27	N
ATOM	661	N	THR	A	86	-14.653	-13.243	25.769	1.00	61.57	N
ATOM	662	CA	THR	A	86	-13.636	-13.804	24.938	1.00	58.16	C
ATOM	663	C	THR	A	86	-13.930	-15.294	24.878	1.00	70.20	C
ATOM	664	O	THR	A	86	-14.813	-15.788	25.585	1.00	74.44	O
ATOM	665	CB	THR	A	86	-12.270	-13.438	25.523	1.00	62.69	C
ATOM	666	OG1	THR	A	86	-11.931	-12.131	25.109	1.00	62.44	O
ATOM	667	CG2	THR	A	86	-11.255	-14.352	25.075	1.00	67.56	C
ATOM	668	N	ASP	A	87	-13.277	-15.995	23.956	1.00	81.52	N
ATOM	669	CA	ASP	A	87	-13.288	-17.448	23.959	1.00	77.48	C
ATOM	670	C	ASP	A	87	-12.634	-17.982	25.223	1.00	66.99	C
ATOM	671	O	ASP	A	87	-11.626	-17.446	25.693	1.00	64.52	O
ATOM	672	CB	ASP	A	87	-12.519	-17.974	22.759	1.00	72.04	C
ATOM	673	CG	ASP	A	87	-13.100	-17.507	21.465	1.00	81.42	C
ATOM	674	OD1	ASP	A	87	-13.958	-18.225	20.898	1.00	85.46	O
ATOM	675	OD2	ASP	A	87	-12.704	-16.399	21.039	1.00	91.43	O
ATOM	676	N	LEU	A	88	-13.205	-19.060	25.765	1.00	62.31	N
ATOM	677	CA	LEU	A	88	-12.564	-19.783	26.861	1.00	62.73	C
ATOM	678	C	LEU	A	88	-11.574	-20.805	26.319	1.00	57.53	C
ATOM	679	O	LEU	A	88	-11.810	-21.431	25.288	1.00	61.93	O
ATOM	680	CB	LEU	A	88	-13.580	-20.485	27.766	1.00	54.18	C
ATOM	681	CG	LEU	A	88	-14.871	-19.765	28.171	1.00	60.46	C
ATOM	682	CD1	LEU	A	88	-15.941	-20.738	28.715	1.00	54.29	C
ATOM	683	CD2	LEU	A	88	-14.562	-18.670	29.178	1.00	52.69	C
ATOM	684	N	SER	A	89	-10.486	-21.000	27.052	1.00	66.19	N
ATOM	685	CA	SER	A	89	-9.357	-21.825	26.646	1.00	58.82	C
ATOM	686	C	SER	A	89	-8.413	-21.992	27.830	1.00	59.39	C
ATOM	687	O	SER	A	89	-8.107	-21.011	28.517	1.00	66.79	O
ATOM	688	CB	SER	A	89	-8.611	-21.169	25.482	1.00	55.78	C
ATOM	689	OG	SER	A	89	-7.234	-21.484	25.563	1.00	65.95	O
ATOM	690	N	GLU	A	90	-7.915	-23.209	28.054	1.00	54.95	N
ATOM	691	CA	GLU	A	90	-6.895	-23.262	29.088	1.00	63.96	C
ATOM	692	C	GLU	A	90	-5.622	-22.537	28.654	1.00	62.70	C
ATOM	693	O	GLU	A	90	-4.754	-22.290	29.497	1.00	67.30	O

ATOM	694	CB	GLU	A	90	-6.556	-24.693	29.475	1.00	64.20	C
ATOM	695	CG	GLU	A	90	-7.633	-25.736	29.332	1.00	67.43	C
ATOM	696	CD	GLU	A	90	-7.244	-27.081	29.988	1.00	81.80	C
ATOM	697	OE1	GLU	A	90	-6.215	-27.134	30.706	1.00	81.00	O
ATOM	698	OE2	GLU	A	90	-7.890	-28.131	29.703	1.00	77.21	O
ATOM	699	N	ASP	A	91	-5.489	-22.186	27.371	1.00	63.41	N
ATOM	700	CA	ASP	A	91	-4.441	-21.271	26.914	1.00	65.56	C
ATOM	701	C	ASP	A	91	-4.933	-19.854	27.165	1.00	67.23	C
ATOM	702	O	ASP	A	91	-5.734	-19.314	26.393	1.00	57.70	O
ATOM	703	CB	ASP	A	91	-4.124	-21.485	25.439	1.00	65.25	C
ATOM	704	CG	ASP	A	91	-2.973	-20.605	24.949	1.00	75.50	C
ATOM	705	OD1	ASP	A	91	-2.235	-20.053	25.790	1.00	75.32	O
ATOM	706	OD2	ASP	A	91	-2.815	-20.446	23.719	1.00	80.08	O
ATOM	707	N	CYS	A	92	-4.442	-19.243	28.250	1.00	70.16	N
ATOM	708	CA	CYS	A	92	-4.988	-17.968	28.701	1.00	70.74	C
ATOM	709	C	CYS	A	92	-4.026	-17.133	29.534	1.00	63.35	C
ATOM	710	O	CYS	A	92	-4.450	-16.095	30.054	1.00	62.23	O
ATOM	711	CB	CYS	A	92	-6.265	-18.217	29.515	1.00	59.39	C
ATOM	712	SG	CYS	A	92	-5.910	-19.182	30.987	1.00	73.81	S
ATOM	713	N	LEU	A	93	-2.758	-17.515	29.678	1.00	58.15	N
ATOM	714	CA	LEU	A	93	-1.854	-16.778	30.564	1.00	65.42	C
ATOM	715	C	LEU	A	93	-1.285	-15.545	29.849	1.00	60.62	C
ATOM	716	O	LEU	A	93	-0.086	-15.431	29.573	1.00	64.11	O
ATOM	717	CB	LEU	A	93	-0.763	-17.707	31.083	1.00	65.92	C
ATOM	718	CG	LEU	A	93	-1.330	-18.709	32.096	1.00	59.88	C
ATOM	719	CD1	LEU	A	93	-0.241	-19.529	32.734	1.00	55.58	C
ATOM	720	CD2	LEU	A	93	-2.143	-17.990	33.163	1.00	60.59	C
ATOM	721	N	TYR	A	94	-2.176	-14.583	29.586	1.00	54.74	N
ATOM	722	CA	TYR	A	94	-1.840	-13.373	28.846	1.00	65.05	C
ATOM	723	C	TYR	A	94	-2.128	-12.140	29.699	1.00	67.00	C
ATOM	724	O	TYR	A	94	-2.945	-12.191	30.622	1.00	70.28	O
ATOM	725	CB	TYR	A	94	-2.617	-13.304	27.511	1.00	60.35	C
ATOM	726	CG	TYR	A	94	-2.351	-14.503	26.640	1.00	58.48	C
ATOM	727	CD1	TYR	A	94	-3.081	-15.674	26.799	1.00	60.37	C
ATOM	728	CD2	TYR	A	94	-1.334	-14.484	25.693	1.00	64.55	C
ATOM	729	CE1	TYR	A	94	-2.807	-16.787	26.038	1.00	70.42	C
ATOM	730	CE2	TYR	A	94	-1.049	-15.590	24.912	1.00	60.88	C
ATOM	731	CZ	TYR	A	94	-1.782	-16.743	25.086	1.00	74.41	C
ATOM	732	OH	TYR	A	94	-1.500	-17.858	24.310	1.00	71.75	O
ATOM	733	N	LEU	A	95	-1.447	-11.025	29.392	1.00	65.68	N
ATOM	734	CA	LEU	A	95	-1.680	-9.760	30.090	1.00	69.71	C
ATOM	735	C	LEU	A	95	-1.782	-8.589	29.102	1.00	78.61	C
ATOM	736	O	LEU	A	95	-1.433	-8.693	27.918	1.00	69.35	O
ATOM	737	CB	LEU	A	95	-0.596	-9.477	31.143	1.00	53.98	C

ATOM	738	CG	LEU	A	95	0.899	-9.489	30.822	1.00	59.14	C
ATOM	739	CD1	LEU	A	95	1.273	-8.443	29.799	1.00	72.55	C
ATOM	740	CD2	LEU	A	95	1.736	-9.287	32.068	1.00	55.72	C
ATOM	741	N	ASN	A	96	-2.251	-7.450	29.623	1.00	72.31	N
ATOM	742	CA	ASN	A	96	-2.510	-6.252	28.836	1.00	66.80	C
ATOM	743	C	ASN	A	96	-1.939	-5.047	29.563	1.00	73.87	C
ATOM	744	O	ASN	A	96	-2.144	-4.876	30.772	1.00	72.21	O
ATOM	745	CB	ASN	A	96	-4.007	-6.011	28.628	1.00	60.38	C
ATOM	746	CG	ASN	A	96	-4.802	-7.282	28.610	1.00	66.33	C
ATOM	747	OD1	ASN	A	96	-4.468	-8.245	27.904	1.00	71.98	O
ATOM	748	ND2	ASN	A	96	-5.865	-7.307	29.399	1.00	65.19	N
ATOM	749	N	VAL	A	97	-1.244	-4.192	28.829	1.00	79.79	N
ATOM	750	CA	VAL	A	97	-0.708	-2.964	29.399	1.00	79.89	C
ATOM	751	C	VAL	A	97	-1.271	-1.795	28.607	1.00	75.41	C
ATOM	752	O	VAL	A	97	-1.443	-1.889	27.386	1.00	81.16	O
ATOM	753	CB	VAL	A	97	0.829	-2.975	29.393	1.00	77.31	C
ATOM	754	CG1	VAL	A	97	1.318	-4.326	29.894	1.00	82.43	C
ATOM	755	CG2	VAL	A	97	1.346	-2.753	28.027	1.00	82.81	C
ATOM	756	N	TRP	A	98	-1.631	-0.730	29.304	1.00	65.50	N
ATOM	757	CA	TRP	A	98	-2.043	0.501	28.653	1.00	71.74	C
ATOM	758	C	TRP	A	98	-1.023	1.581	28.984	1.00	79.41	C
ATOM	759	O	TRP	A	98	-0.647	1.753	30.150	1.00	79.13	O
ATOM	760	CB	TRP	A	98	-3.445	0.935	29.091	1.00	69.11	C
ATOM	761	CG	TRP	A	98	-4.586	0.106	28.553	1.00	71.36	C
ATOM	762	CD1	TRP	A	98	-5.253	0.290	27.373	1.00	75.40	C
ATOM	763	CD2	TRP	A	98	-5.224	-1.003	29.202	1.00	73.70	C
ATOM	764	NE1	TRP	A	98	-6.252	-0.647	27.236	1.00	66.30	N
ATOM	765	CE2	TRP	A	98	-6.259	-1.449	28.348	1.00	68.20	C
ATOM	766	CE3	TRP	A	98	-5.014	-1.668	30.415	1.00	66.05	C
ATOM	767	CZ2	TRP	A	98	-7.080	-2.525	28.670	1.00	66.31	C
ATOM	768	CZ3	TRP	A	98	-5.831	-2.736	30.733	1.00	68.85	C
ATOM	769	CH2	TRP	A	98	-6.854	-3.155	29.861	1.00	70.86	C
ATOM	770	N	ILE	A	99	-0.555	2.287	27.956	1.00	76.90	N
ATOM	771	CA	ILE	A	99	0.538	3.247	28.113	1.00	78.84	C
ATOM	772	C	ILE	A	99	0.011	4.614	27.695	1.00	84.52	C
ATOM	773	O	ILE	A	99	-0.683	4.728	26.674	1.00	91.06	O
ATOM	774	CB	ILE	A	99	1.778	2.855	27.279	1.00	82.34	C
ATOM	775	CG1	ILE	A	99	2.751	1.947	28.025	1.00	81.64	C
ATOM	776	CG2	ILE	A	99	2.600	4.076	26.915	1.00	89.76	C
ATOM	777	CD1	ILE	A	99	2.184	0.637	28.430	1.00	92.21	C
ATOM	778	N	PRO	A	100	0.304	5.672	28.445	1.00	88.42	N
ATOM	779	CA	PRO	A	100	-0.070	7.022	28.000	1.00	94.37	C
ATOM	780	C	PRO	A	100	0.666	7.412	26.724	1.00	88.22	C
ATOM	781	O	PRO	A	100	1.745	6.897	26.421	1.00	84.47	O

```
ATOM    782  CB  PRO A 100       0.350   7.913  29.177  1.00 83.17           C
ATOM    783  CG  PRO A 100       0.414   6.998  30.336  1.00 79.09           C
ATOM    784  CD  PRO A 100       0.857   5.664  29.804  1.00 73.17           C
ATOM    785  N   ALA A 101       0.076   8.339  25.973  1.00 92.24           N
ATOM    786  CA  ALA A 101       0.781   8.916  24.818  1.00 97.87           C
ATOM    787  C   ALA A 101       0.823  10.446  24.887  1.00 88.77           C
ATOM    788  O   ALA A 101      -0.215  11.088  25.033  1.00 86.24           O
ATOM    789  CB  ALA A 101       0.151   8.462  23.521  1.00100.06           C
ATOM    790  N   PRO A 102       2.026  11.041  24.764  1.00101.15           N
ATOM    791  CA  PRO A 102       3.317  10.427  24.411  1.00 93.50           C
ATOM    792  C   PRO A 102       3.816   9.477  25.476  1.00 91.73           C
ATOM    793  O   PRO A 102       3.584   9.754  26.653  1.00 96.50           O
ATOM    794  CB  PRO A 102       4.279  11.626  24.312  1.00 95.89           C
ATOM    795  CG  PRO A 102       3.425  12.861  24.387  1.00 93.16           C
ATOM    796  CD  PRO A 102       2.189  12.460  25.132  1.00 99.27           C
ATOM    797  N   LYS A 103       4.481   8.401  25.063  1.00 91.53           N
ATOM    798  CA  LYS A 103       4.996   7.396  25.979  1.00 91.63           C
ATOM    799  C   LYS A 103       5.695   8.066  27.165  1.00 93.29           C
ATOM    800  O   LYS A 103       6.599   8.896  26.962  1.00 73.48           O
ATOM    801  CB  LYS A 103       5.951   6.449  25.243  1.00 93.16           C
ATOM    802  CG  LYS A 103       7.153   5.947  26.048  1.00 94.53           C
ATOM    803  CD  LYS A 103       7.742   4.678  25.420  1.00 95.35           C
ATOM    804  CE  LYS A 103       9.177   4.894  24.941  1.00 95.03           C
ATOM    805  NZ  LYS A 103       9.714   3.655  24.327  1.00 90.22           N
ATOM    806  N   PRO A 104       5.287   7.767  28.403  1.00 94.13           N
ATOM    807  CA  PRO A 104       5.915   8.404  29.563  1.00 91.80           C
ATOM    808  C   PRO A 104       7.348   7.940  29.745  1.00 94.24           C
ATOM    809  O   PRO A 104       7.825   7.087  28.986  1.00 94.25           O
ATOM    810  CB  PRO A 104       5.020   7.961  30.727  1.00 86.90           C
ATOM    811  CG  PRO A 104       4.511   6.629  30.292  1.00 96.61           C
ATOM    812  CD  PRO A 104       4.308   6.737  28.802  1.00 95.16           C
ATOM    813  N   LYS A 105       8.047   8.477  30.742  1.00 93.50           N
ATOM    814  CA  LYS A 105       9.425   8.072  30.932  1.00 86.99           C
ATOM    815  C   LYS A 105       9.677   7.342  32.241  1.00 93.16           C
ATOM    816  O   LYS A 105      10.494   6.420  32.252  1.00103.31           O
ATOM    817  CB  LYS A 105      10.373   9.278  30.808  1.00 92.75           C
ATOM    818  CG  LYS A 105      11.787   8.991  31.299  1.00 93.81           C
ATOM    819  CD  LYS A 105      12.861   9.740  30.509  1.00 96.13           C
ATOM    820  CE  LYS A 105      12.360  11.064  29.927  1.00108.52           C
ATOM    821  NZ  LYS A 105      12.327  12.173  30.935  1.00105.04           N
ATOM    822  N   ASN A 106       8.985   7.675  33.329  1.00 87.52           N
ATOM    823  CA  ASN A 106       9.177   6.899  34.568  1.00 94.01           C
ATOM    824  C   ASN A 106       7.855   6.779  35.328  1.00 94.68           C
ATOM    825  O   ASN A 106       7.745   7.084  36.519  1.00 79.87           O
```

ATOM	826	CB	ASN	A	106	10.274	7.522	35.432	1.00	92.43	C
ATOM	827	CG	ASN	A	106	11.157	6.476	36.099	1.00	84.77	C
ATOM	828	OD1	ASN	A	106	11.254	5.344	35.622	1.00	96.02	O
ATOM	829	ND2	ASN	A	106	11.811	6.851	37.200	1.00	74.52	N
ATOM	830	N	ALA	A	107	6.834	6.281	34.634	1.00	97.78	N
ATOM	831	CA	ALA	A	107	5.462	6.354	35.113	1.00	93.09	C
ATOM	832	C	ALA	A	107	5.182	5.314	36.194	1.00	88.04	C
ATOM	833	O	ALA	A	107	5.777	4.231	36.228	1.00	88.03	O
ATOM	834	CB	ALA	A	107	4.483	6.169	33.947	1.00	81.86	C
ATOM	835	N	THR	A	108	4.261	5.662	37.085	1.00	82.08	N
ATOM	836	CA	THR	A	108	3.740	4.694	38.037	1.00	84.37	C
ATOM	837	C	THR	A	108	2.921	3.641	37.296	1.00	92.50	C
ATOM	838	O	THR	A	108	2.236	3.941	36.308	1.00	86.76	O
ATOM	839	CB	THR	A	108	2.898	5.403	39.113	1.00	89.22	C
ATOM	840	OG1	THR	A	108	3.758	6.135	40.002	1.00	89.95	O
ATOM	841	CG2	THR	A	108	2.064	4.428	39.927	1.00	79.78	C
ATOM	842	N	VAL	A	109	3.027	2.394	37.762	1.00	91.79	N
ATOM	843	CA	VAL	A	109	2.338	1.247	37.182	1.00	74.35	C
ATOM	844	C	VAL	A	109	1.258	0.784	38.147	1.00	72.47	C
ATOM	845	O	VAL	A	109	1.530	0.573	39.336	1.00	73.67	O
ATOM	846	CB	VAL	A	109	3.321	0.108	36.889	1.00	74.42	C
ATOM	847	CG1	VAL	A	109	2.594	-1.054	36.241	1.00	73.81	C
ATOM	848	CG2	VAL	A	109	4.450	0.616	36.014	1.00	79.23	C
ATOM	849	N	LEU	A	110	0.038	0.630	37.635	1.00	74.51	N
ATOM	850	CA	LEU	A	110	-1.093	0.072	38.372	1.00	71.57	C
ATOM	851	C	LEU	A	110	-1.443	-1.288	37.786	1.00	72.20	C
ATOM	852	O	LEU	A	110	-1.636	-1.409	36.571	1.00	75.12	O
ATOM	853	CB	LEU	A	110	-2.319	0.977	38.282	1.00	70.01	C
ATOM	854	CG	LEU	A	110	-2.437	2.219	39.147	1.00	72.19	C
ATOM	855	CD1	LEU	A	110	-3.533	3.121	38.585	1.00	63.59	C
ATOM	856	CD2	LEU	A	110	-2.753	1.799	40.564	1.00	74.70	C
ATOM	857	N	ILE	A	111	-1.557	-2.301	38.640	1.00	68.57	N
ATOM	858	CA	ILE	A	111	-1.809	-3.661	38.190	1.00	62.52	C
ATOM	859	C	ILE	A	111	-3.152	-4.117	38.754	1.00	61.95	C
ATOM	860	O	ILE	A	111	-3.374	-4.074	39.970	1.00	58.30	O
ATOM	861	CB	ILE	A	111	-0.657	-4.600	38.594	1.00	71.24	C
ATOM	862	CG1	ILE	A	111	0.671	-4.016	38.093	1.00	65.20	C
ATOM	863	CG2	ILE	A	111	-0.877	-6.018	38.020	1.00	70.11	C
ATOM	864	CD1	ILE	A	111	1.891	-4.923	38.248	1.00	66.10	C
ATOM	865	N	TRP	A	112	-4.039	-4.564	37.869	1.00	60.68	N
ATOM	866	CA	TRP	A	112	-5.395	-4.957	38.244	1.00	67.79	C
ATOM	867	C	TRP	A	112	-5.549	-6.478	38.301	1.00	70.17	C
ATOM	868	O	TRP	A	112	-5.215	-7.185	37.344	1.00	70.51	O
ATOM	869	CB	TRP	A	112	-6.401	-4.373	37.259	1.00	55.40	C

ATOM	870	CG	TRP	A	112	-7.803	-4.788	37.480	1.00	52.12	C
ATOM	871	CD1	TRP	A	112	-8.569	-5.524	36.646	1.00	58.82	C
ATOM	872	CD2	TRP	A	112	-8.624	-4.480	38.607	1.00	51.84	C
ATOM	873	NE1	TRP	A	112	-9.821	-5.695	37.174	1.00	54.30	N
ATOM	874	CE2	TRP	A	112	-9.878	-5.054	38.379	1.00	50.06	C
ATOM	875	CE3	TRP	A	112	-8.424	-3.763	39.779	1.00	61.74	C
ATOM	876	CZ2	TRP	A	112	-10.923	-4.943	39.284	1.00	62.58	C
ATOM	877	CZ3	TRP	A	112	-9.472	-3.655	40.682	1.00	60.47	C
ATOM	878	CH2	TRP	A	112	-10.698	-4.245	40.430	1.00	55.17	C
ATOM	879	N	ILE	A	113	-6.077	-6.973	39.415	1.00	60.87	N
ATOM	880	CA	ILE	A	113	-6.369	-8.386	39.589	1.00	63.30	C
ATOM	881	C	ILE	A	113	-7.869	-8.517	39.830	1.00	67.15	C
ATOM	882	O	ILE	A	113	-8.381	-8.061	40.864	1.00	60.27	O
ATOM	883	CB	ILE	A	113	-5.560	-8.994	40.738	1.00	63.84	C
ATOM	884	CG1	ILE	A	113	-4.058	-8.896	40.435	1.00	62.95	C
ATOM	885	CG2	ILE	A	113	-6.001	-10.426	40.981	1.00	60.58	C
ATOM	886	CD1	ILE	A	113	-3.148	-9.259	41.637	1.00	59.73	C
ATOM	887	N	TYR	A	114	-8.570	-9.152	38.888	1.00	67.89	N
ATOM	888	CA	TYR	A	114	-10.028	-9.158	38.895	1.00	67.21	C
ATOM	889	C	TYR	A	114	-10.599	-10.154	39.906	1.00	57.70	C
ATOM	890	O	TYR	A	114	-9.990	-11.180	40.229	1.00	50.01	O
ATOM	891	CB	TYR	A	114	-10.564	-9.469	37.493	1.00	61.69	C
ATOM	892	CG	TYR	A	114	-10.035	-10.760	36.896	1.00	65.40	C
ATOM	893	CD1	TYR	A	114	-10.626	-11.987	37.191	1.00	58.19	C
ATOM	894	CD2	TYR	A	114	-8.951	-10.754	36.030	1.00	69.41	C
ATOM	895	CE1	TYR	A	114	-10.143	-13.159	36.659	1.00	55.92	C
ATOM	896	CE2	TYR	A	114	-8.470	-11.942	35.479	1.00	69.44	C
ATOM	897	CZ	TYR	A	114	-9.072	-13.132	35.801	1.00	61.05	C
ATOM	898	OH	TYR	A	114	-8.596	-14.299	35.262	1.00	66.70	O
ATOM	899	N	GLY	A	115	-11.800	-9.841	40.397	1.00	54.54	N
ATOM	900	CA	GLY	A	115	-12.485	-10.714	41.327	1.00	66.20	C
ATOM	901	C	GLY	A	115	-13.376	-11.722	40.624	1.00	66.69	C
ATOM	902	O	GLY	A	115	-13.626	-11.598	39.434	1.00	60.49	O
ATOM	903	N	GLY	A	116	-13.844	-12.717	41.392	1.00	76.30	N
ATOM	904	CA	GLY	A	116	-14.648	-13.805	40.848	1.00	77.09	C
ATOM	905	C	GLY	A	116	-14.817	-15.026	41.754	1.00	81.48	C
ATOM	906	O	GLY	A	116	-15.022	-16.162	41.266	1.00	63.87	O
ATOM	907	N	GLY	A	117	-14.752	-14.808	43.078	1.00	62.42	N
ATOM	908	CA	GLY	A	117	-14.757	-15.941	43.993	1.00	62.40	C
ATOM	909	C	GLY	A	117	-13.770	-17.034	43.606	1.00	49.75	C
ATOM	910	O	GLY	A	117	-13.965	-18.204	43.942	1.00	47.57	O
ATOM	911	N	PHE	A	118	-12.715	-16.650	42.883	1.00	47.70	N
ATOM	912	CA	PHE	A	118	-11.701	-17.534	42.317	1.00	55.81	C
ATOM	913	C	PHE	A	118	-12.284	-18.597	41.387	1.00	64.16	C

ATOM	914	O	PHE	A	118	-11.556	-19.518	40.975	1.00	55.78	O
ATOM	915	CB	PHE	A	118	-10.867	-18.224	43.405	1.00	55.48	C
ATOM	916	CG	PHE	A	118	-10.114	-17.285	44.292	1.00	48.45	C
ATOM	917	CD1	PHE	A	118	-8.929	-16.706	43.863	1.00	49.72	C
ATOM	918	CD2	PHE	A	118	-10.580	-17.001	45.568	1.00	50.18	C
ATOM	919	CE1	PHE	A	118	-8.229	-15.840	44.692	1.00	61.99	C
ATOM	920	CE2	PHE	A	118	-9.892	-16.138	46.399	1.00	49.95	C
ATOM	921	CZ	PHE	A	118	-8.718	-15.553	45.964	1.00	56.58	C
ATOM	922	N	GLN	A	119	-13.575	-18.512	41.048	1.00	57.04	N
ATOM	923	CA	GLN	A	119	-14.188	-19.488	40.167	1.00	48.08	C
ATOM	924	C	GLN	A	119	-14.444	-18.946	38.770	1.00	63.40	C
ATOM	925	O	GLN	A	119	-14.760	-19.732	37.863	1.00	66.30	O
ATOM	926	CB	GLN	A	119	-15.512	-19.988	40.758	1.00	47.60	C
ATOM	927	CG	GLN	A	119	-15.439	-20.525	42.186	1.00	57.17	C
ATOM	928	CD	GLN	A	119	-14.389	-21.603	42.370	1.00	66.55	C
ATOM	929	OE1	GLN	A	119	-14.615	-22.783	42.050	1.00	72.03	O
ATOM	930	NE2	GLN	A	119	-13.236	-21.210	42.901	1.00	57.59	N
ATOM	931	N	THR	A	120	-14.267	-17.643	38.559	1.00	57.56	N
ATOM	932	CA	THR	A	120	-14.708	-16.984	37.339	1.00	57.59	C
ATOM	933	C	THR	A	120	-13.861	-15.734	37.125	1.00	62.05	C
ATOM	934	O	THR	A	120	-13.078	-15.341	37.990	1.00	61.51	O
ATOM	935	CB	THR	A	120	-16.191	-16.607	37.434	1.00	64.43	C
ATOM	936	OG1	THR	A	120	-16.413	-15.877	38.655	1.00	65.97	O
ATOM	937	CG2	THR	A	120	-17.107	-17.850	37.388	1.00	47.02	C
ATOM	938	N	GLY	A	121	-14.040	-15.103	35.958	1.00	64.28	N
ATOM	939	CA	GLY	A	121	-13.495	-13.777	35.725	1.00	62.55	C
ATOM	940	C	GLY	A	121	-12.421	-13.679	34.659	1.00	64.23	C
ATOM	941	O	GLY	A	121	-11.820	-14.695	34.288	1.00	61.34	O
ATOM	942	N	THR	A	122	-12.142	-12.453	34.199	1.00	60.88	N
ATOM	943	CA	THR	A	122	-11.168	-12.197	33.142	1.00	64.31	C
ATOM	944	C	THR	A	122	-10.855	-10.703	33.029	1.00	61.54	C
ATOM	945	O	THR	A	122	-11.724	-9.849	33.225	1.00	52.36	O
ATOM	946	CB	THR	A	122	-11.666	-12.734	31.794	1.00	61.09	C
ATOM	947	OG1	THR	A	122	-10.669	-12.511	30.792	1.00	55.80	O
ATOM	948	CG2	THR	A	122	-12.963	-12.065	31.391	1.00	62.80	C
ATOM	949	N	SER	A	123	-9.603	-10.406	32.673	1.00	65.14	N
ATOM	950	CA	SER	A	123	-9.110	-9.036	32.554	1.00	71.14	C
ATOM	951	C	SER	A	123	-9.682	-8.295	31.353	1.00	74.11	C
ATOM	952	O	SER	A	123	-9.330	-7.130	31.119	1.00	74.34	O
ATOM	953	CB	SER	A	123	-7.590	-9.050	32.443	1.00	72.78	C
ATOM	954	OG	SER	A	123	-7.219	-9.583	31.185	1.00	55.83	O
ATOM	955	N	SER	A	124	-10.550	-8.941	30.597	1.00	73.78	N
ATOM	956	CA	SER	A	124	-11.014	-8.425	29.328	1.00	67.91	C
ATOM	957	C	SER	A	124	-12.319	-7.643	29.435	1.00	68.61	C

ATOM	958	O	SER	A	124	-12.761	-7.076	28.425	1.00	75.75	O
ATOM	959	CB	SER	A	124	-11.160	-9.599	28.345	1.00	68.26	C
ATOM	960	OG	SER	A	124	-12.114	-9.314	27.345	1.00	78.78	O
ATOM	961	N	LEU	A	125	-12.932	-7.580	30.621	1.00	65.37	N
ATOM	962	CA	LEU	A	125	-14.242	-6.945	30.757	1.00	73.43	C
ATOM	963	C	LEU	A	125	-14.180	-5.438	30.500	1.00	71.67	C
ATOM	964	O	LEU	A	125	-13.160	-4.773	30.740	1.00	60.82	O
ATOM	965	CB	LEU	A	125	-14.854	-7.202	32.147	1.00	72.55	C
ATOM	966	CG	LEU	A	125	-15.159	-8.632	32.637	1.00	62.20	C
ATOM	967	CD1	LEU	A	125	-15.882	-8.588	33.959	1.00	50.85	C
ATOM	968	CD2	LEU	A	125	-15.985	-9.397	31.623	1.00	69.27	C
ATOM	969	N	HIS	A	126	-15.304	-4.904	30.000	1.00	67.76	N
ATOM	970	CA	HIS	A	126	-15.380	-3.479	29.690	1.00	66.96	C
ATOM	971	C	HIS	A	126	-15.207	-2.617	30.944	1.00	62.08	C
ATOM	972	O	HIS	A	126	-14.649	-1.518	30.867	1.00	78.76	O
ATOM	973	CB	HIS	A	126	-16.702	-3.182	28.960	1.00	67.93	C
ATOM	974	CG	HIS	A	126	-16.955	-1.726	28.687	1.00	78.28	C
ATOM	975	ND1	HIS	A	126	-18.102	-1.079	29.102	1.00	73.52	N
ATOM	976	CD2	HIS	A	126	-16.208	-0.791	28.049	1.00	78.89	C
ATOM	977	CE1	HIS	A	126	-18.053	0.187	28.728	1.00	68.77	C
ATOM	978	NE2	HIS	A	126	-16.912	0.390	28.094	1.00	70.25	N
ATOM	979	N	VAL	A	127	-15.626	-3.100	32.113	1.00	51.04	N
ATOM	980	CA	VAL	A	127	-15.408	-2.315	33.321	1.00	61.30	C
ATOM	981	C	VAL	A	127	-13.959	-2.326	33.798	1.00	59.32	C
ATOM	982	O	VAL	A	127	-13.625	-1.578	34.718	1.00	61.55	O
ATOM	983	CB	VAL	A	127	-16.306	-2.791	34.483	1.00	62.02	C
ATOM	984	CG1	VAL	A	127	-17.749	-2.992	34.033	1.00	59.42	C
ATOM	985	CG2	VAL	A	127	-15.761	-4.060	35.070	1.00	64.95	C
ATOM	986	N	TYR	A	128	-13.088	-3.166	33.238	1.00	65.09	N
ATOM	987	CA	TYR	A	128	-11.677	-3.153	33.613	1.00	60.73	C
ATOM	988	C	TYR	A	128	-10.820	-2.465	32.574	1.00	69.02	C
ATOM	989	O	TYR	A	128	-9.606	-2.701	32.541	1.00	71.80	O
ATOM	990	CB	TYR	A	128	-11.135	-4.570	33.839	1.00	63.02	C
ATOM	991	CG	TYR	A	128	-11.962	-5.441	34.755	1.00	64.10	C
ATOM	992	CD1	TYR	A	128	-12.463	-4.946	35.953	1.00	63.09	C
ATOM	993	CD2	TYR	A	128	-12.231	-6.765	34.427	1.00	66.89	C
ATOM	994	CE1	TYR	A	128	-13.210	-5.732	36.788	1.00	60.98	C
ATOM	995	CE2	TYR	A	128	-12.977	-7.560	35.268	1.00	68.21	C
ATOM	996	CZ	TYR	A	128	-13.463	-7.032	36.443	1.00	61.47	C
ATOM	997	OH	TYR	A	128	-14.213	-7.805	37.275	1.00	56.93	O
ATOM	998	N	ASP	A	129	-11.427	-1.643	31.713	1.00	72.58	N
ATOM	999	CA	ASP	A	129	-10.726	-1.000	30.602	1.00	65.26	C
ATOM	1000	C	ASP	A	129	-9.803	0.074	31.166	1.00	77.91	C
ATOM	1001	O	ASP	A	129	-10.244	1.184	31.497	1.00	81.42	O

ATOM	1002	CB	ASP	A	129	-11.721	-0.399	29.617	1.00	67.67	C
ATOM	1003	CG	ASP	A	129	-11.075	-0.012	28.304	1.00	75.03	C
ATOM	1004	OD1	ASP	A	129	-11.794	-0.001	27.278	1.00	80.36	O
ATOM	1005	OD2	ASP	A	129	-9.844	0.238	28.290	1.00	72.62	O
ATOM	1006	N	GLY	A	130	-8.507	-0.244	31.252	1.00	69.57	N
ATOM	1007	CA	GLY	A	130	-7.553	0.669	31.853	1.00	75.28	C
ATOM	1008	C	GLY	A	130	-7.268	1.944	31.076	1.00	75.70	C
ATOM	1009	O	GLY	A	130	-6.557	2.803	31.605	1.00	75.91	O
ATOM	1010	N	LYS	A	131	-7.801	2.105	29.857	1.00	70.32	N
ATOM	1011	CA	LYS	A	131	-7.353	3.202	29.003	1.00	74.59	C
ATOM	1012	C	LYS	A	131	-7.623	4.568	29.646	1.00	80.86	C
ATOM	1013	O	LYS	A	131	-6.759	5.454	29.604	1.00	83.18	O
ATOM	1014	CB	LYS	A	131	-7.984	3.094	27.604	1.00	63.27	C
ATOM	1015	CG	LYS	A	131	-9.460	3.477	27.493	1.00	65.27	C
ATOM	1016	CD	LYS	A	131	-10.040	3.130	26.131	1.00	66.48	C
ATOM	1017	CE	LYS	A	131	-11.561	3.248	26.156	1.00	68.70	C
ATOM	1018	NZ	LYS	A	131	-12.252	2.667	24.952	1.00	55.27	N
ATOM	1019	N	PHE	A	132	-8.785	4.748	30.294	1.00	77.83	N
ATOM	1020	CA	PHE	A	132	-9.092	6.055	30.881	1.00	73.44	C
ATOM	1021	C	PHE	A	132	-8.088	6.430	31.975	1.00	72.71	C
ATOM	1022	O	PHE	A	132	-7.704	7.596	32.101	1.00	67.54	O
ATOM	1023	CB	PHE	A	132	-10.527	6.086	31.423	1.00	65.23	C
ATOM	1024	CG	PHE	A	132	-11.552	5.545	30.466	1.00	66.06	C
ATOM	1025	CD1	PHE	A	132	-11.908	6.247	29.325	1.00	80.27	C
ATOM	1026	CD2	PHE	A	132	-12.171	4.334	30.709	1.00	76.12	C
ATOM	1027	CE1	PHE	A	132	-12.866	5.742	28.422	1.00	78.33	C
ATOM	1028	CE2	PHE	A	132	-13.126	3.828	29.819	1.00	84.27	C
ATOM	1029	CZ	PHE	A	132	-13.475	4.541	28.673	1.00	75.09	C
ATOM	1030	N	LEU	A	133	-7.633	5.461	32.766	1.00	74.57	N
ATOM	1031	CA	LEU	A	133	-6.625	5.785	33.771	1.00	73.81	C
ATOM	1032	C	LEU	A	133	-5.323	6.265	33.140	1.00	75.70	C
ATOM	1033	O	LEU	A	133	-4.698	7.203	33.643	1.00	81.41	O
ATOM	1034	CB	LEU	A	133	-6.364	4.577	34.656	1.00	70.95	C
ATOM	1035	CG	LEU	A	133	-7.455	4.383	35.692	1.00	75.57	C
ATOM	1036	CD1	LEU	A	133	-7.452	2.934	36.156	1.00	69.32	C
ATOM	1037	CD2	LEU	A	133	-7.237	5.361	36.854	1.00	60.39	C
ATOM	1038	N	ALA	A	134	-4.883	5.627	32.057	1.00	76.81	N
ATOM	1039	CA	ALA	A	134	-3.641	6.050	31.422	1.00	77.28	C
ATOM	1040	C	ALA	A	134	-3.810	7.423	30.790	1.00	88.09	C
ATOM	1041	O	ALA	A	134	-2.948	8.306	30.919	1.00	82.31	O
ATOM	1042	CB	ALA	A	134	-3.212	5.022	30.378	1.00	75.35	C
ATOM	1043	N	ARG	A	135	-4.933	7.619	30.105	1.00	88.35	N
ATOM	1044	CA	ARG	A	135	-5.231	8.912	29.510	1.00	80.80	C
ATOM	1045	C	ARG	A	135	-5.265	10.002	30.572	1.00	79.71	C

```
ATOM   1046  O   ARG A 135      -4.500  10.968  30.508  1.00 90.73           O
ATOM   1047  CB  ARG A 135      -6.564   8.833  28.772  1.00 79.02           C
ATOM   1048  CG  ARG A 135      -7.044  10.147  28.248  1.00 81.43           C
ATOM   1049  CD  ARG A 135      -6.117  10.642  27.183  1.00 81.28           C
ATOM   1050  NE  ARG A 135      -6.678  11.826  26.556  1.00 86.71           N
ATOM   1051  CZ  ARG A 135      -6.337  13.062  26.890  1.00 85.41           C
ATOM   1052  NH1 ARG A 135      -5.421  13.257  27.835  1.00 76.27           N
ATOM   1053  NH2 ARG A 135      -6.904  14.092  26.272  1.00 75.89           N
ATOM   1054  N   VAL A 136      -6.124   9.837  31.582  1.00 76.50           N
ATOM   1055  CA  VAL A 136      -6.453  10.938  32.486  1.00 66.28           C
ATOM   1056  C   VAL A 136      -5.298  11.240  33.431  1.00 74.99           C
ATOM   1057  O   VAL A 136      -4.876  12.393  33.557  1.00 92.44           O
ATOM   1058  CB  VAL A 136      -7.741  10.627  33.264  1.00 65.97           C
ATOM   1059  CG1 VAL A 136      -7.913  11.607  34.376  1.00 75.43           C
ATOM   1060  CG2 VAL A 136      -8.929  10.674  32.342  1.00 64.14           C
ATOM   1061  N   GLU A 137      -4.785  10.227  34.124  1.00 70.11           N
ATOM   1062  CA  GLU A 137      -3.758  10.430  35.135  1.00 73.10           C
ATOM   1063  C   GLU A 137      -2.383   9.910  34.724  1.00 79.71           C
ATOM   1064  O   GLU A 137      -1.500   9.772  35.585  1.00 73.83           O
ATOM   1065  CB  GLU A 137      -4.201   9.799  36.450  1.00 85.20           C
ATOM   1066  CG  GLU A 137      -5.529  10.382  36.924  1.00 91.86           C
ATOM   1067  CD  GLU A 137      -5.351  11.543  37.890  1.00 97.02           C
ATOM   1068  OE1 GLU A 137      -6.300  12.370  37.999  1.00 92.53           O
ATOM   1069  OE2 GLU A 137      -4.265  11.639  38.539  1.00103.79           O
ATOM   1070  N   ARG A 138      -2.187   9.612  33.435  1.00 84.19           N
ATOM   1071  CA  ARG A 138      -0.861   9.353  32.864  1.00 83.97           C
ATOM   1072  C   ARG A 138      -0.138   8.245  33.626  1.00 75.72           C
ATOM   1073  O   ARG A 138       1.032   8.384  33.996  1.00 64.96           O
ATOM   1074  CB  ARG A 138      -0.024  10.638  32.818  1.00 97.88           C
ATOM   1075  CG  ARG A 138      -0.108  11.421  31.474  1.00 91.59           C
ATOM   1076  CD  ARG A 138       1.136  12.275  31.240  1.00 89.65           C
ATOM   1077  NE  ARG A 138       2.041  11.749  30.213  0.96 94.61           N
ATOM   1078  CZ  ARG A 138       3.343  11.528  30.411  0.75 96.74           C
ATOM   1079  NH1 ARG A 138       3.875  11.726  31.615  1.00 94.10           N
ATOM   1080  NH2 ARG A 138       4.103  11.081  29.419  1.00 91.95           N
ATOM   1081  N   VAL A 139      -0.862   7.155  33.885  1.00 82.50           N
ATOM   1082  CA  VAL A 139      -0.315   5.961  34.516  1.00 78.73           C
ATOM   1083  C   VAL A 139      -0.213   4.852  33.480  1.00 82.53           C
ATOM   1084  O   VAL A 139      -0.769   4.934  32.384  1.00 83.08           O
ATOM   1085  CB  VAL A 139      -1.174   5.484  35.693  1.00 74.21           C
ATOM   1086  CG1 VAL A 139      -1.078   6.436  36.818  1.00 71.13           C
ATOM   1087  CG2 VAL A 139      -2.630   5.331  35.222  1.00 70.35           C
ATOM   1088  N   ILE A 140       0.463   3.776  33.862  1.00 84.96           N
ATOM   1089  CA  ILE A 140       0.462   2.544  33.084  1.00 81.95           C
```

```
ATOM   1090  C   ILE A 140      -0.387   1.542  33.853  1.00 81.10           C
ATOM   1091  O   ILE A 140      -0.083   1.214  35.008  1.00 79.72           O
ATOM   1092  CB  ILE A 140       1.884   2.023  32.830  1.00 78.17           C
ATOM   1093  CG1 ILE A 140       2.655   3.035  31.993  1.00 73.57           C
ATOM   1094  CG2 ILE A 140       1.838   0.689  32.110  1.00 77.50           C
ATOM   1095  CD1 ILE A 140       4.072   2.677  31.777  1.00 73.83           C
ATOM   1096  N   VAL A 141      -1.486   1.117  33.238  1.00 73.44           N
ATOM   1097  CA  VAL A 141      -2.378   0.120  33.804  1.00 62.64           C
ATOM   1098  C   VAL A 141      -2.028  -1.208  33.166  1.00 68.93           C
ATOM   1099  O   VAL A 141      -1.928  -1.307  31.933  1.00 68.09           O
ATOM   1100  CB  VAL A 141      -3.854   0.469  33.550  1.00 68.73           C
ATOM   1101  CG1 VAL A 141      -4.768  -0.480  34.318  1.00 59.97           C
ATOM   1102  CG2 VAL A 141      -4.135   1.911  33.909  1.00 68.30           C
ATOM   1103  N   VAL A 142      -1.849  -2.228  34.001  1.00 77.75           N
ATOM   1104  CA  VAL A 142      -1.571  -3.591  33.564  1.00 68.74           C
ATOM   1105  C   VAL A 142      -2.624  -4.509  34.171  1.00 66.82           C
ATOM   1106  O   VAL A 142      -3.082  -4.287  35.301  1.00 65.66           O
ATOM   1107  CB  VAL A 142      -0.155  -4.046  33.975  1.00 63.12           C
ATOM   1108  CG1 VAL A 142       0.250  -5.253  33.178  1.00 65.09           C
ATOM   1109  CG2 VAL A 142       0.853  -2.909  33.813  1.00 74.42           C
ATOM   1110  N   SER A 143      -3.013  -5.536  33.420  1.00 54.85           N
ATOM   1111  CA  SER A 143      -3.942  -6.529  33.937  1.00 57.80           C
ATOM   1112  C   SER A 143      -3.601  -7.876  33.319  1.00 72.44           C
ATOM   1113  O   SER A 143      -3.174  -7.950  32.161  1.00 74.89           O
ATOM   1114  CB  SER A 143      -5.417  -6.153  33.672  1.00 66.82           C
ATOM   1115  OG  SER A 143      -5.716  -5.855  32.307  1.00 62.87           O
ATOM   1116  N   MET A 144      -3.789  -8.944  34.103  1.00 73.98           N
ATOM   1117  CA  MET A 144      -3.442 -10.294  33.674  1.00 68.28           C
ATOM   1118  C   MET A 144      -4.599 -11.256  33.917  1.00 66.78           C
ATOM   1119  O   MET A 144      -5.469 -11.025  34.761  1.00 73.47           O
ATOM   1120  CB  MET A 144      -2.184 -10.819  34.390  1.00 56.65           C
ATOM   1121  CG  MET A 144      -2.423 -11.452  35.749  1.00 55.93           C
ATOM   1122  SD  MET A 144      -3.006 -10.306  37.015  1.00 52.59           S
ATOM   1123  CE  MET A 144      -1.436  -9.752  37.718  1.00 56.45           C
ATOM   1124  N   ASN A 145      -4.599 -12.340  33.146  1.00 60.79           N
ATOM   1125  CA  ASN A 145      -5.460 -13.485  33.397  1.00 70.38           C
ATOM   1126  C   ASN A 145      -4.678 -14.532  34.174  1.00 69.25           C
ATOM   1127  O   ASN A 145      -3.530 -14.842  33.836  1.00 67.39           O
ATOM   1128  CB  ASN A 145      -5.998 -14.091  32.093  1.00 71.46           C
ATOM   1129  CG  ASN A 145      -6.929 -13.146  31.354  1.00 75.63           C
ATOM   1130  OD1 ASN A 145      -6.908 -11.933  31.587  1.00 73.71           O
ATOM   1131  ND2 ASN A 145      -7.768 -13.698  30.475  1.00 72.67           N
ATOM   1132  N   TYR A 146      -5.299 -15.057  35.227  1.00 64.52           N
ATOM   1133  CA  TYR A 146      -4.734 -16.116  36.045  1.00 60.95           C
```

ATOM	1134	C	TYR	A	146	-5.755	-17.240	36.118	1.00	58.84	C
ATOM	1135	O	TYR	A	146	-6.962	-16.994	36.098	1.00	58.94	O
ATOM	1136	CB	TYR	A	146	-4.385	-15.604	37.455	1.00	63.07	C
ATOM	1137	CG	TYR	A	146	-5.594	-15.100	38.229	1.00	60.54	C
ATOM	1138	CD1	TYR	A	146	-6.327	-15.948	39.039	1.00	62.49	C
ATOM	1139	CD2	TYR	A	146	-6.016	-13.791	38.121	1.00	54.58	C
ATOM	1140	CE1	TYR	A	146	-7.435	-15.500	39.721	1.00	63.97	C
ATOM	1141	CE2	TYR	A	146	-7.123	-13.335	38.806	1.00	54.17	C
ATOM	1142	CZ	TYR	A	146	-7.828	-14.188	39.605	1.00	60.51	C
ATOM	1143	OH	TYR	A	146	-8.942	-13.737	40.292	1.00	62.69	O
ATOM	1144	N	ARG	A	147	-5.271	-18.476	36.195	1.00	59.38	N
ATOM	1145	CA	ARG	A	147	-6.175	-19.618	36.227	1.00	52.55	C
ATOM	1146	C	ARG	A	147	-7.070	-19.596	37.469	1.00	56.80	C
ATOM	1147	O	ARG	A	147	-6.785	-18.947	38.476	1.00	60.69	O
ATOM	1148	CB	ARG	A	147	-5.381	-20.906	36.187	1.00	52.27	C
ATOM	1149	CG	ARG	A	147	-4.840	-21.218	34.840	1.00	59.82	C
ATOM	1150	CD	ARG	A	147	-3.842	-22.316	34.975	1.00	56.82	C
ATOM	1151	NE	ARG	A	147	-2.542	-21.815	35.396	1.00	49.99	N
ATOM	1152	CZ	ARG	A	147	-1.540	-22.620	35.716	1.00	55.74	C
ATOM	1153	NH1	ARG	A	147	-1.738	-23.944	35.674	1.00	62.84	N
ATOM	1154	NH2	ARG	A	147	-0.357	-22.114	36.064	1.00	48.34	N
ATOM	1155	N	VAL	A	148	-8.137	-20.383	37.404	1.00	58.92	N
ATOM	1156	CA	VAL	A	148	-9.365	-20.124	38.143	1.00	50.28	C
ATOM	1157	C	VAL	A	148	-10.142	-21.435	38.296	1.00	53.01	C
ATOM	1158	O	VAL	A	148	-10.024	-22.353	37.481	1.00	63.89	O
ATOM	1159	CB	VAL	A	148	-10.155	-19.034	37.373	1.00	54.90	C
ATOM	1160	CG1	VAL	A	148	-11.567	-19.119	37.598	1.00	53.91	C
ATOM	1161	CG2	VAL	A	148	-9.690	-17.669	37.736	1.00	60.65	C
ATOM	1162	N	GLY	A	149	-10.940	-21.531	39.354	1.00	57.54	N
ATOM	1163	CA	GLY	A	149	-11.560	-22.818	39.589	1.00	53.88	C
ATOM	1164	C	GLY	A	149	-10.485	-23.859	39.836	1.00	64.82	C
ATOM	1165	O	GLY	A	149	-9.353	-23.543	40.231	1.00	59.14	O
ATOM	1166	N	ALA	A	150	-10.825	-25.124	39.563	1.00	62.69	N
ATOM	1167	CA	ALA	A	150	-9.885	-26.195	39.864	1.00	56.49	C
ATOM	1168	C	ALA	A	150	-8.614	-26.107	39.014	1.00	53.24	C
ATOM	1169	O	ALA	A	150	-7.548	-26.535	39.468	1.00	52.45	O
ATOM	1170	CB	ALA	A	150	-10.561	-27.558	39.707	1.00	54.15	C
ATOM	1171	N	LEU	A	151	-8.658	-25.551	37.804	1.00	50.87	N
ATOM	1172	CA	LEU	A	151	-7.360	-25.542	37.149	1.00	51.20	C
ATOM	1173	C	LEU	A	151	-6.545	-24.357	37.614	1.00	47.43	C
ATOM	1174	O	LEU	A	151	-5.499	-24.037	37.053	1.00	55.73	O
ATOM	1175	CB	LEU	A	151	-7.498	-25.613	35.616	1.00	54.35	C
ATOM	1176	CG	LEU	A	151	-6.247	-25.386	34.738	1.00	68.04	C
ATOM	1177	CD1	LEU	A	151	-5.045	-26.228	35.216	1.00	59.27	C

```
ATOM   1178  CD2 LEU A 151      -6.515 -25.621  33.271  1.00 68.25           C
ATOM   1179  N   GLY A 152      -6.991 -23.701  38.667  1.00 52.36           N
ATOM   1180  CA  GLY A 152      -6.182 -22.651  39.238  1.00 53.14           C
ATOM   1181  C   GLY A 152      -5.785 -22.993  40.657  1.00 56.97           C
ATOM   1182  O   GLY A 152      -4.732 -22.570  41.146  1.00 58.07           O
ATOM   1183  N   PHE A 153      -6.618 -23.772  41.334  1.00 55.50           N
ATOM   1184  CA  PHE A 153      -6.445 -23.902  42.766  1.00 63.27           C
ATOM   1185  C   PHE A 153      -6.597 -25.308  43.320  1.00 64.39           C
ATOM   1186  O   PHE A 153      -6.492 -25.473  44.544  1.00 72.04           O
ATOM   1187  CB  PHE A 153      -7.403 -22.926  43.452  1.00 56.97           C
ATOM   1188  CG  PHE A 153      -6.999 -21.513  43.226  1.00 54.07           C
ATOM   1189  CD1 PHE A 153      -6.033 -20.934  44.019  1.00 59.54           C
ATOM   1190  CD2 PHE A 153      -7.498 -20.802  42.175  1.00 54.63           C
ATOM   1191  CE1 PHE A 153      -5.620 -19.661  43.789  1.00 57.38           C
ATOM   1192  CE2 PHE A 153      -7.085 -19.520  41.935  1.00 53.47           C
ATOM   1193  CZ  PHE A 153      -6.144 -18.952  42.740  1.00 59.09           C
ATOM   1194  N   LEU A 154      -6.797 -26.315  42.467  1.00 47.19           N
ATOM   1195  CA  LEU A 154      -6.812 -27.705  42.897  1.00 55.74           C
ATOM   1196  C   LEU A 154      -5.562 -27.994  43.705  1.00 71.20           C
ATOM   1197  O   LEU A 154      -4.458 -27.613  43.300  1.00 77.70           O
ATOM   1198  CB  LEU A 154      -6.872 -28.621  41.674  1.00 57.33           C
ATOM   1199  CG  LEU A 154      -7.085 -30.121  41.849  1.00 52.12           C
ATOM   1200  CD1 LEU A 154      -8.554 -30.480  41.616  1.00 46.42           C
ATOM   1201  CD2 LEU A 154      -6.180 -30.878  40.900  1.00 49.79           C
ATOM   1202  N   ALA A 155      -5.733 -28.652  44.856  1.00 64.12           N
ATOM   1203  CA  ALA A 155      -4.629 -28.856  45.785  1.00 66.23           C
ATOM   1204  C   ALA A 155      -4.553 -30.302  46.239  1.00 79.15           C
ATOM   1205  O   ALA A 155      -5.512 -30.820  46.825  1.00 79.99           O
ATOM   1206  CB  ALA A 155      -4.776 -27.954  47.007  1.00 71.05           C
ATOM   1207  N   LEU A 156      -3.412 -30.939  45.995  1.00 72.81           N
ATOM   1208  CA  LEU A 156      -2.932 -31.996  46.890  1.00 75.86           C
ATOM   1209  C   LEU A 156      -1.620 -31.495  47.466  1.00 78.26           C
ATOM   1210  O   LEU A 156      -0.620 -31.395  46.725  1.00 69.05           O
ATOM   1211  CB  LEU A 156      -2.738 -33.334  46.186  1.00 73.80           C
ATOM   1212  CG  LEU A 156      -3.946 -33.692  45.334  1.00 78.05           C
ATOM   1213  CD1 LEU A 156      -3.533 -33.474  43.932  1.00 75.10           C
ATOM   1214  CD2 LEU A 156      -4.391 -35.128  45.539  1.00 84.64           C
ATOM   1215  N   PRO A 157      -1.587 -31.161  48.760  1.00 72.99           N
ATOM   1216  CA  PRO A 157      -0.461 -30.390  49.305  1.00 67.51           C
ATOM   1217  C   PRO A 157       0.814 -31.211  49.409  1.00 67.02           C
ATOM   1218  O   PRO A 157       0.794 -32.389  49.770  1.00 80.76           O
ATOM   1219  CB  PRO A 157      -0.970 -29.957  50.684  1.00 68.62           C
ATOM   1220  CG  PRO A 157      -2.482 -30.054  50.571  1.00 70.70           C
ATOM   1221  CD  PRO A 157      -2.728 -31.219  49.686  1.00 73.56           C
```

```
ATOM   1222  N   GLY A 158       1.931 -30.564  49.084  1.00 66.52           N
ATOM   1223  CA  GLY A 158       3.217 -31.214  49.015  1.00 65.50           C
ATOM   1224  C   GLY A 158       3.516 -31.896  47.701  1.00 71.78           C
ATOM   1225  O   GLY A 158       4.628 -32.417  47.534  1.00 78.62           O
ATOM   1226  N   ASN A 159       2.572 -31.898  46.767  1.00 64.18           N
ATOM   1227  CA  ASN A 159       2.723 -32.553  45.475  1.00 68.53           C
ATOM   1228  C   ASN A 159       2.908 -31.535  44.354  1.00 76.79           C
ATOM   1229  O   ASN A 159       1.916 -30.945  43.897  1.00 76.83           O
ATOM   1230  CB  ASN A 159       1.495 -33.417  45.202  1.00 71.13           C
ATOM   1231  CG  ASN A 159       1.730 -34.418  44.104  1.00 79.52           C
ATOM   1232  OD1 ASN A 159       2.507 -34.169  43.182  1.00 80.95           O
ATOM   1233  ND2 ASN A 159       1.081 -35.575  44.206  1.00 79.35           N
ATOM   1234  N   PRO A 160       4.120 -31.333  43.827  1.00 75.15           N
ATOM   1235  CA  PRO A 160       4.295 -30.314  42.781  1.00 74.39           C
ATOM   1236  C   PRO A 160       3.468 -30.577  41.539  1.00 74.32           C
ATOM   1237  O   PRO A 160       3.285 -29.662  40.728  1.00 77.46           O
ATOM   1238  CB  PRO A 160       5.795 -30.383  42.472  1.00 74.01           C
ATOM   1239  CG  PRO A 160       6.125 -31.801  42.719  1.00 83.56           C
ATOM   1240  CD  PRO A 160       5.302 -32.199  43.933  1.00 84.58           C
ATOM   1241  N   GLU A 161       2.962 -31.794  41.349  1.00 76.70           N
ATOM   1242  CA  GLU A 161       2.075 -32.018  40.214  1.00 88.42           C
ATOM   1243  C   GLU A 161       0.778 -31.224  40.344  1.00 76.67           C
ATOM   1244  O   GLU A 161       0.158 -30.892  39.328  1.00 74.43           O
ATOM   1245  CB  GLU A 161       1.777 -33.522  40.041  1.00 90.07           C
ATOM   1246  CG  GLU A 161       2.680 -34.285  39.026  1.00 88.26           C
ATOM   1247  CD  GLU A 161       4.054 -34.704  39.581  1.00109.77           C
ATOM   1248  OE1 GLU A 161       4.132 -35.733  40.309  1.00 98.01           O
ATOM   1249  OE2 GLU A 161       5.058 -34.019  39.259  1.00106.35           O
ATOM   1250  N   ALA A 162       0.373 -30.891  41.553  1.00 71.10           N
ATOM   1251  CA  ALA A 162      -0.859 -30.154  41.759  1.00 64.14           C
ATOM   1252  C   ALA A 162      -0.790 -29.507  43.136  1.00 66.91           C
ATOM   1253  O   ALA A 162      -1.532 -29.895  44.055  1.00 65.40           O
ATOM   1254  CB  ALA A 162      -2.057 -31.090  41.621  1.00 58.17           C
ATOM   1255  N   PRO A 163       0.101 -28.527  43.319  1.00 61.64           N
ATOM   1256  CA  PRO A 163       0.432 -28.082  44.687  1.00 71.03           C
ATOM   1257  C   PRO A 163      -0.647 -27.252  45.357  1.00 65.30           C
ATOM   1258  O   PRO A 163      -0.669 -27.183  46.593  1.00 59.82           O
ATOM   1259  CB  PRO A 163       1.721 -27.268  44.495  1.00 68.57           C
ATOM   1260  CG  PRO A 163       1.762 -26.913  43.075  1.00 64.46           C
ATOM   1261  CD  PRO A 163       0.968 -27.917  42.300  1.00 60.15           C
ATOM   1262  N   GLY A 164      -1.556 -26.672  44.594  1.00 64.01           N
ATOM   1263  CA  GLY A 164      -2.411 -25.602  45.065  1.00 59.84           C
ATOM   1264  C   GLY A 164      -1.813 -24.251  44.739  1.00 66.05           C
ATOM   1265  O   GLY A 164      -0.598 -24.096  44.594  1.00 64.78           O
```

```
ATOM   1266  N   ASN A 165      -2.703 -23.260  44.575  1.00 66.73           N
ATOM   1267  CA  ASN A 165      -2.380 -21.843  44.377  1.00 57.88           C
ATOM   1268  C   ASN A 165      -1.774 -21.521  43.011  1.00 58.39           C
ATOM   1269  O   ASN A 165      -1.231 -20.422  42.828  1.00 56.09           O
ATOM   1270  CB  ASN A 165      -1.460 -21.315  45.483  1.00 47.48           C
ATOM   1271  CG  ASN A 165      -2.162 -21.234  46.812  1.00 57.42           C
ATOM   1272  OD1 ASN A 165      -3.389 -21.286  46.867  1.00 58.52           O
ATOM   1273  ND2 ASN A 165      -1.398 -21.116  47.895  1.00 59.11           N
ATOM   1274  N   MET A 166      -1.879 -22.416  42.023  1.00 54.24           N
ATOM   1275  CA  MET A 166      -1.323 -22.096  40.711  1.00 58.32           C
ATOM   1276  C   MET A 166      -1.871 -20.780  40.167  1.00 63.99           C
ATOM   1277  O   MET A 166      -1.233 -20.146  39.318  1.00 63.14           O
ATOM   1278  CB  MET A 166      -1.595 -23.219  39.709  1.00 58.41           C
ATOM   1279  CG  MET A 166      -1.146 -24.581  40.149  1.00 56.94           C
ATOM   1280  SD  MET A 166      -2.279 -25.219  41.370  1.00 72.25           S
ATOM   1281  CE  MET A 166      -3.486 -26.031  40.324  1.00 63.91           C
ATOM   1282  N   GLY A 167      -3.047 -20.357  40.637  1.00 63.78           N
ATOM   1283  CA  GLY A 167      -3.583 -19.078  40.208  1.00 53.07           C
ATOM   1284  C   GLY A 167      -2.749 -17.920  40.718  1.00 63.89           C
ATOM   1285  O   GLY A 167      -2.203 -17.138  39.934  1.00 55.63           O
ATOM   1286  N   LEU A 168      -2.614 -17.798  42.043  1.00 65.47           N
ATOM   1287  CA  LEU A 168      -1.792 -16.702  42.547  1.00 64.30           C
ATOM   1288  C   LEU A 168      -0.389 -16.764  41.964  1.00 65.97           C
ATOM   1289  O   LEU A 168       0.225 -15.723  41.699  1.00 69.89           O
ATOM   1290  CB  LEU A 168      -1.744 -16.664  44.079  1.00 51.28           C
ATOM   1291  CG  LEU A 168      -3.076 -16.628  44.855  1.00 54.40           C
ATOM   1292  CD1 LEU A 168      -3.242 -17.767  45.857  1.00 50.06           C
ATOM   1293  CD2 LEU A 168      -3.365 -15.273  45.482  1.00 53.97           C
ATOM   1294  N   PHE A 169       0.119 -17.971  41.711  1.00 65.25           N
ATOM   1295  CA  PHE A 169       1.429 -18.084  41.083  1.00 60.00           C
ATOM   1296  C   PHE A 169       1.421 -17.491  39.684  1.00 59.84           C
ATOM   1297  O   PHE A 169       2.406 -16.865  39.273  1.00 57.63           O
ATOM   1298  CB  PHE A 169       1.888 -19.539  41.056  1.00 55.82           C
ATOM   1299  CG  PHE A 169       2.618 -19.953  42.295  1.00 61.83           C
ATOM   1300  CD1 PHE A 169       2.146 -20.983  43.094  1.00 66.51           C
ATOM   1301  CD2 PHE A 169       3.771 -19.286  42.679  1.00 66.41           C
ATOM   1302  CE1 PHE A 169       2.823 -21.358  44.238  1.00 63.53           C
ATOM   1303  CE2 PHE A 169       4.452 -19.644  43.816  1.00 62.78           C
ATOM   1304  CZ  PHE A 169       3.982 -20.684  44.597  1.00 74.28           C
ATOM   1305  N   ASP A 170       0.325 -17.695  38.932  1.00 66.60           N
ATOM   1306  CA  ASP A 170       0.133 -16.999  37.657  1.00 61.81           C
ATOM   1307  C   ASP A 170       0.174 -15.490  37.857  1.00 67.37           C
ATOM   1308  O   ASP A 170       0.864 -14.773  37.116  1.00 66.60           O
ATOM   1309  CB  ASP A 170      -1.200 -17.390  36.999  1.00 60.05           C
```

ATOM	1310	CG	ASP	A	170	-1.253	-18.848	36.569	1.00	63.92	C
ATOM	1311	OD1	ASP	A	170	-0.179	-19.478	36.512	1.00	60.95	O
ATOM	1312	OD2	ASP	A	170	-2.368	-19.364	36.291	1.00	61.27	O
ATOM	1313	N	GLN	A	171	-0.573	-14.983	38.853	1.00	64.51	N
ATOM	1314	CA	GLN	A	171	-0.545	-13.547	39.118	1.00	57.04	C
ATOM	1315	C	GLN	A	171	0.879	-13.069	39.329	1.00	59.10	C
ATOM	1316	O	GLN	A	171	1.279	-12.031	38.795	1.00	67.51	O
ATOM	1317	CB	GLN	A	171	-1.395	-13.181	40.335	1.00	54.54	C
ATOM	1318	CG	GLN	A	171	-2.805	-13.704	40.325	1.00	61.66	C
ATOM	1319	CD	GLN	A	171	-3.560	-13.317	41.582	1.00	59.42	C
ATOM	1320	OE1	GLN	A	171	-4.781	-13.201	41.575	1.00	63.52	O
ATOM	1321	NE2	GLN	A	171	-2.829	-13.102	42.665	1.00	58.35	N
ATOM	1322	N	GLN	A	172	1.677	-13.838	40.064	1.00	63.46	N
ATOM	1323	CA	GLN	A	172	2.978	-13.345	40.490	1.00	64.09	C
ATOM	1324	C	GLN	A	172	4.005	-13.408	39.360	1.00	59.35	C
ATOM	1325	O	GLN	A	172	4.839	-12.504	39.244	1.00	62.74	O
ATOM	1326	CB	GLN	A	172	3.421	-14.110	41.750	1.00	64.55	C
ATOM	1327	CG	GLN	A	172	4.860	-13.864	42.242	1.00	70.94	C
ATOM	1328	CD	GLN	A	172	5.297	-14.856	43.329	1.00	71.39	C
ATOM	1329	OE1	GLN	A	172	5.186	-14.563	44.529	1.00	73.58	O
ATOM	1330	NE2	GLN	A	172	5.819	-16.015	42.914	1.00	62.46	N
ATOM	1331	N	LEU	A	173	3.939	-14.418	38.489	1.00	68.40	N
ATOM	1332	CA	LEU	A	173	4.796	-14.371	37.308	1.00	72.72	C
ATOM	1333	C	LEU	A	173	4.426	-13.186	36.413	1.00	72.49	C
ATOM	1334	O	LEU	A	173	5.314	-12.575	35.800	1.00	70.60	O
ATOM	1335	CB	LEU	A	173	4.741	-15.699	36.545	1.00	56.10	C
ATOM	1336	CG	LEU	A	173	5.931	-16.106	35.647	1.00	61.40	C
ATOM	1337	CD1	LEU	A	173	5.702	-17.486	35.052	1.00	79.00	C
ATOM	1338	CD2	LEU	A	173	6.227	-15.149	34.507	1.00	70.60	C
ATOM	1339	N	ALA	A	174	3.139	-12.819	36.354	1.00	65.41	N
ATOM	1340	CA	ALA	A	174	2.772	-11.547	35.727	1.00	73.10	C
ATOM	1341	C	ALA	A	174	3.486	-10.373	36.399	1.00	69.69	C
ATOM	1342	O	ALA	A	174	4.073	-9.522	35.719	1.00	59.73	O
ATOM	1343	CB	ALA	A	174	1.255	-11.348	35.759	1.00	70.13	C
ATOM	1344	N	LEU	A	175	3.449	-10.315	37.739	1.00	63.94	N
ATOM	1345	CA	LEU	A	175	4.204	-9.297	38.460	1.00	61.57	C
ATOM	1346	C	LEU	A	175	5.677	-9.355	38.111	1.00	70.25	C
ATOM	1347	O	LEU	A	175	6.323	-8.317	37.922	1.00	77.40	O
ATOM	1348	CB	LEU	A	175	4.027	-9.460	39.965	1.00	60.09	C
ATOM	1349	CG	LEU	A	175	2.593	-9.385	40.470	1.00	64.13	C
ATOM	1350	CD1	LEU	A	175	2.606	-9.114	41.946	1.00	67.27	C
ATOM	1351	CD2	LEU	A	175	1.799	-8.321	39.745	1.00	61.78	C
ATOM	1352	N	GLN	A	176	6.241	-10.559	38.037	0.66	74.73	N
ATOM	1354	CA	GLN	A	176	7.636	-10.678	37.628	0.66	78.54	C

```
ATOM   1356  C    GLN A 176       7.863 -10.039  36.264  0.66 79.76           C
ATOM   1358  O    GLN A 176       8.816  -9.268  36.081  0.66 81.39           O
ATOM   1360  CB   GLN A 176       8.072 -12.147  37.620  0.66 72.61           C
ATOM   1362  CG   GLN A 176       8.272 -12.726  39.012  0.66 71.70           C
ATOM   1364  CD   GLN A 176       8.996 -14.055  38.999  0.66 79.70           C
ATOM   1366  OE1  GLN A 176       9.405 -14.542  37.944  0.66 81.01           O
ATOM   1368  NE2  GLN A 176       9.169 -14.647  40.177  0.66 81.06           N
ATOM   1370  N    TRP A 177       6.981 -10.324  35.301  1.00 74.56           N
ATOM   1371  CA   TRP A 177       7.159  -9.784  33.964  1.00 72.85           C
ATOM   1372  C    TRP A 177       7.180  -8.261  33.992  1.00 77.91           C
ATOM   1373  O    TRP A 177       8.001  -7.634  33.314  1.00 83.81           O
ATOM   1374  CB   TRP A 177       6.055 -10.309  33.046  1.00 76.90           C
ATOM   1375  CG   TRP A 177       6.172  -9.873  31.611  1.00 74.04           C
ATOM   1376  CD1  TRP A 177       6.823 -10.533  30.614  1.00 78.72           C
ATOM   1377  CD2  TRP A 177       5.589  -8.711  31.009  1.00 74.50           C
ATOM   1378  NE1  TRP A 177       6.709  -9.845  29.436  1.00 88.84           N
ATOM   1379  CE2  TRP A 177       5.955  -8.721  29.647  1.00 74.53           C
ATOM   1380  CE3  TRP A 177       4.804  -7.656  31.490  1.00 81.25           C
ATOM   1381  CZ2  TRP A 177       5.566  -7.726  28.759  1.00 71.65           C
ATOM   1382  CZ3  TRP A 177       4.421  -6.659  30.601  1.00 89.87           C
ATOM   1383  CH2  TRP A 177       4.801  -6.710  29.245  1.00 78.12           C
ATOM   1384  N    VAL A 178       6.305  -7.652  34.799  1.00 75.22           N
ATOM   1385  CA   VAL A 178       6.287  -6.198  34.935  1.00 72.92           C
ATOM   1386  C    VAL A 178       7.613  -5.696  35.488  1.00 83.98           C
ATOM   1387  O    VAL A 178       8.143  -4.672  35.035  1.00 80.12           O
ATOM   1388  CB   VAL A 178       5.106  -5.767  35.821  1.00 75.86           C
ATOM   1389  CG1  VAL A 178       5.371  -4.393  36.450  1.00 79.04           C
ATOM   1390  CG2  VAL A 178       3.822  -5.755  35.001  1.00 73.12           C
ATOM   1391  N    GLN A 179       8.160  -6.400  36.485  1.00 84.92           N
ATOM   1392  CA   GLN A 179       9.447  -6.016  37.054  1.00 82.36           C
ATOM   1393  C    GLN A 179      10.531  -5.942  35.990  1.00 88.66           C
ATOM   1394  O    GLN A 179      11.361  -5.024  36.002  1.00 97.10           O
ATOM   1395  CB   GLN A 179       9.850  -6.999  38.150  1.00 86.82           C
ATOM   1396  CG   GLN A 179       9.229  -6.687  39.488  1.00 85.07           C
ATOM   1397  CD   GLN A 179       9.324  -5.216  39.823  1.00 91.17           C
ATOM   1398  OE1  GLN A 179      10.345  -4.743  40.326  1.00 97.70           O
ATOM   1399  NE2  GLN A 179       8.257  -4.476  39.530  1.00 87.57           N
ATOM   1400  N    LYS A 180      10.535  -6.885  35.052  1.00 92.58           N
ATOM   1401  CA   LYS A 180      11.639  -6.961  34.102  1.00 95.77           C
ATOM   1402  C    LYS A 180      11.392  -6.158  32.826  1.00 89.75           C
ATOM   1403  O    LYS A 180      12.353  -5.679  32.218  1.00 97.26           O
ATOM   1404  CB   LYS A 180      11.948  -8.433  33.770  1.00 83.26           C
ATOM   1405  CG   LYS A 180      12.401  -9.303  34.982  1.00 81.27           C
ATOM   1406  CD   LYS A 180      12.931  -8.457  36.182  1.00 89.01           C
```

```
ATOM   1407  CE  LYS A 180      13.765  -9.249  37.210  1.00 88.97           C
ATOM   1408  NZ  LYS A 180      13.071 -10.422  37.815  1.00 85.61           N
ATOM   1409  N   ASN A 181      10.133  -5.952  32.429  1.00 83.67           N
ATOM   1410  CA  ASN A 181       9.818  -5.425  31.107  1.00 81.50           C
ATOM   1411  C   ASN A 181       9.228  -4.027  31.103  1.00 86.63           C
ATOM   1412  O   ASN A 181       9.369  -3.327  30.098  1.00 88.11           O
ATOM   1413  CB  ASN A 181       8.835  -6.362  30.384  1.00 85.99           C
ATOM   1414  CG  ASN A 181       9.436  -7.730  30.098  1.00 93.83           C
ATOM   1415  OD1 ASN A 181      10.142  -7.914  29.106  1.00 96.40           O
ATOM   1416  ND2 ASN A 181       9.211  -8.679  31.002  1.00 85.40           N
ATOM   1417  N   ILE A 182       8.567  -3.604  32.181  1.00 89.60           N
ATOM   1418  CA  ILE A 182       7.766  -2.391  32.102  1.00 82.99           C
ATOM   1419  C   ILE A 182       8.610  -1.166  31.779  1.00 86.88           C
ATOM   1420  O   ILE A 182       8.096  -0.215  31.187  1.00 88.18           O
ATOM   1421  CB  ILE A 182       6.954  -2.170  33.398  1.00 85.86           C
ATOM   1422  CG1 ILE A 182       5.639  -1.463  33.058  1.00 77.93           C
ATOM   1423  CG2 ILE A 182       7.751  -1.348  34.423  1.00 79.95           C
ATOM   1424  CD1 ILE A 182       4.773  -2.239  32.099  1.00 74.02           C
ATOM   1425  N   ALA A 183       9.896  -1.160  32.142  1.00 92.55           N
ATOM   1426  CA  ALA A 183      10.753  -0.042  31.761  1.00 88.89           C
ATOM   1427  C   ALA A 183      10.701   0.195  30.255  1.00 79.49           C
ATOM   1428  O   ALA A 183      10.484   1.326  29.806  1.00 77.83           O
ATOM   1429  CB  ALA A 183      12.181  -0.285  32.246  1.00 80.00           C
ATOM   1430  N   ALA A 184      10.846  -0.875  29.460  1.00 86.67           N
ATOM   1431  CA  ALA A 184      10.683  -0.767  28.011  1.00 88.76           C
ATOM   1432  C   ALA A 184       9.491   0.110  27.658  1.00 93.91           C
ATOM   1433  O   ALA A 184       9.630   1.021  26.864  1.00105.76           O
ATOM   1434  CB  ALA A 184      10.502  -2.146  27.352  1.00 80.86           C
ATOM   1435  N   PHE A 185       8.353  -0.071  28.312  1.00 98.17           N
ATOM   1436  CA  PHE A 185       7.115   0.651  28.043  1.00 92.05           C
ATOM   1437  C   PHE A 185       7.085   2.046  28.643  1.00 89.84           C
ATOM   1438  O   PHE A 185       6.071   2.732  28.500  1.00 96.13           O
ATOM   1439  CB  PHE A 185       5.912  -0.150  28.577  1.00 93.46           C
ATOM   1440  CG  PHE A 185       5.759  -1.499  27.948  1.00 88.23           C
ATOM   1441  CD1 PHE A 185       6.525  -2.561  28.383  1.00 86.06           C
ATOM   1442  CD2 PHE A 185       4.913  -1.685  26.869  1.00 79.64           C
ATOM   1443  CE1 PHE A 185       6.417  -3.797  27.783  1.00 93.41           C
ATOM   1444  CE2 PHE A 185       4.811  -2.918  26.266  1.00 82.26           C
ATOM   1445  CZ  PHE A 185       5.551  -3.970  26.715  1.00 91.92           C
ATOM   1446  N   GLY A 186       8.154   2.490  29.296  1.00 85.55           N
ATOM   1447  CA  GLY A 186       8.113   3.758  29.987  1.00 82.98           C
ATOM   1448  C   GLY A 186       7.564   3.670  31.386  1.00 86.80           C
ATOM   1449  O   GLY A 186       7.101   4.683  31.924  1.00 97.57           O
ATOM   1450  N   GLY A 187       7.585   2.485  31.988  1.00 84.29           N
```

ATOM	1451	CA	GLY	A	187	7.104	2.288	33.333	1.00	79.94	C
ATOM	1452	C	GLY	A	187	8.253	2.218	34.314	1.00	90.03	C
ATOM	1453	O	GLY	A	187	9.404	2.006	33.931	1.00	87.99	O
ATOM	1454	N	ASN	A	188	7.917	2.406	35.595	1.00	91.14	N
ATOM	1455	CA	ASN	A	188	8.891	2.423	36.683	1.00	86.42	C
ATOM	1456	C	ASN	A	188	8.696	1.205	37.583	1.00	89.93	C
ATOM	1457	O	ASN	A	188	7.853	1.239	38.496	1.00	85.31	O
ATOM	1458	CB	ASN	A	188	8.758	3.721	37.490	1.00	89.44	C
ATOM	1459	CG	ASN	A	188	9.738	3.803	38.667	1.00	94.37	C
ATOM	1460	OD1	ASN	A	188	10.446	2.840	38.979	1.00	98.84	O
ATOM	1461	ND2	ASN	A	188	9.799	4.976	39.308	1.00	79.31	N
ATOM	1462	N	PRO	A	189	9.497	0.117	37.407	1.00	93.54	N
ATOM	1463	CA	PRO	A	189	9.317	-1.095	38.227	1.00	86.72	C
ATOM	1464	C	PRO	A	189	9.517	-0.869	39.717	1.00	93.89	C
ATOM	1465	O	PRO	A	189	9.511	-1.833	40.493	1.00	93.30	O
ATOM	1466	CB	PRO	A	189	10.380	-2.059	37.676	1.00	86.30	C
ATOM	1467	CG	PRO	A	189	11.406	-1.170	37.036	1.00	89.71	C
ATOM	1468	CD	PRO	A	189	10.607	-0.032	36.448	1.00	91.55	C
ATOM	1469	N	LYS	A	190	9.712	0.388	40.126	1.00	91.16	N
ATOM	1470	CA	LYS	A	190	9.787	0.752	41.535	1.00	88.94	C
ATOM	1471	C	LYS	A	190	8.660	1.701	41.946	1.00	89.73	C
ATOM	1472	O	LYS	A	190	8.696	2.269	43.041	1.00	80.28	O
ATOM	1473	CB	LYS	A	190	11.170	1.341	41.847	1.00	80.82	C
ATOM	1474	CG	LYS	A	190	11.519	1.537	43.335	1.00	90.58	C
ATOM	1475	CD	LYS	A	190	10.818	0.561	44.318	1.00	89.47	C
ATOM	1476	CE	LYS	A	190	11.232	-0.906	44.167	1.00100.04		C
ATOM	1477	NZ	LYS	A	190	10.157	-1.864	44.611	1.00	95.59	N
ATOM	1478	N	SER	A	191	7.645	1.882	41.104	1.00	92.02	N
ATOM	1479	CA	SER	A	191	6.386	2.485	41.550	1.00	88.65	C
ATOM	1480	C	SER	A	191	5.270	1.597	41.007	1.00	94.93	C
ATOM	1481	O	SER	A	191	4.691	1.870	39.948	1.00	88.81	O
ATOM	1482	CB	SER	A	191	6.244	3.921	41.106	1.00	87.11	C
ATOM	1483	OG	SER	A	191	5.164	4.521	41.795	1.00	88.43	O
ATOM	1484	N	VAL	A	192	4.998	0.519	41.744	1.00	92.10	N
ATOM	1485	CA	VAL	A	192	4.077	-0.537	41.348	1.00	77.57	C
ATOM	1486	C	VAL	A	192	3.127	-0.753	42.509	1.00	75.57	C
ATOM	1487	O	VAL	A	192	3.571	-1.078	43.614	1.00	79.95	O
ATOM	1488	CB	VAL	A	192	4.817	-1.842	41.025	1.00	76.04	C
ATOM	1489	CG1	VAL	A	192	3.910	-2.797	40.286	1.00	78.79	C
ATOM	1490	CG2	VAL	A	192	6.080	-1.558	40.237	1.00	78.62	C
ATOM	1491	N	THR	A	193	1.830	-0.548	42.282	1.00	75.13	N
ATOM	1492	CA	THR	A	193	0.839	-0.749	43.337	1.00	78.98	C
ATOM	1493	C	THR	A	193	-0.254	-1.652	42.798	1.00	73.94	C
ATOM	1494	O	THR	A	193	-0.850	-1.354	41.756	1.00	71.54	O

ATOM	1495	CB	THR	A	193	0.243	0.577	43.849	1.00	75.88	C
ATOM	1496	OG1	THR	A	193	-0.902	0.953	43.070	1.00	70.79	O
ATOM	1497	CG2	THR	A	193	1.271	1.690	43.773	1.00	84.26	C
ATOM	1498	N	LEU	A	194	-0.499	-2.759	43.492	1.00	57.59	N
ATOM	1499	CA	LEU	A	194	-1.552	-3.668	43.072	1.00	66.31	C
ATOM	1500	C	LEU	A	194	-2.902	-3.122	43.489	1.00	60.33	C
ATOM	1501	O	LEU	A	194	-3.015	-2.362	44.448	1.00	64.71	O
ATOM	1502	CB	LEU	A	194	-1.352	-5.068	43.658	1.00	71.00	C
ATOM	1503	CG	LEU	A	194	0.031	-5.717	43.489	1.00	67.20	C
ATOM	1504	CD1	LEU	A	194	0.128	-6.987	44.316	1.00	58.41	C
ATOM	1505	CD2	LEU	A	194	0.320	-5.996	42.010	1.00	73.81	C
ATOM	1506	N	PHE	A	195	-3.927	-3.492	42.739	1.00	62.84	N
ATOM	1507	CA	PHE	A	195	-5.282	-3.180	43.160	1.00	65.40	C
ATOM	1508	C	PHE	A	195	-6.192	-4.214	42.534	1.00	61.24	C
ATOM	1509	O	PHE	A	195	-6.039	-4.553	41.357	1.00	61.49	O
ATOM	1510	CB	PHE	A	195	-5.700	-1.729	42.815	1.00	51.46	C
ATOM	1511	CG	PHE	A	195	-5.927	-1.428	41.338	1.00	61.52	C
ATOM	1512	CD1	PHE	A	195	-4.932	-1.653	40.389	1.00	62.73	C
ATOM	1513	CD2	PHE	A	195	-7.116	-0.810	40.920	1.00	58.81	C
ATOM	1514	CE1	PHE	A	195	-5.142	-1.339	39.042	1.00	63.89	C
ATOM	1515	CE2	PHE	A	195	-7.336	-0.484	39.575	1.00	52.75	C
ATOM	1516	CZ	PHE	A	195	-6.350	-0.752	38.632	1.00	60.81	C
ATOM	1517	N	GLY	A	196	-7.076	-4.764	43.357	1.00	58.15	N
ATOM	1518	CA	GLY	A	196	-8.039	-5.747	42.927	1.00	52.71	C
ATOM	1519	C	GLY	A	196	-9.338	-5.495	43.653	1.00	60.88	C
ATOM	1520	O	GLY	A	196	-9.437	-4.633	44.527	1.00	66.41	O
ATOM	1521	N	GLU	A	197	-10.340	-6.269	43.293	1.00	64.32	N
ATOM	1522	CA	GLU	A	197	-11.609	-6.216	43.983	1.00	61.80	C
ATOM	1523	C	GLU	A	197	-12.041	-7.643	44.278	1.00	61.58	C
ATOM	1524	O	GLU	A	197	-11.806	-8.545	43.473	1.00	62.79	O
ATOM	1525	CB	GLU	A	197	-12.651	-5.492	43.143	1.00	61.63	C
ATOM	1526	CG	GLU	A	197	-13.926	-5.202	43.877	1.00	69.90	C
ATOM	1527	CD	GLU	A	197	-15.023	-6.203	43.581	1.00	76.65	C
ATOM	1528	OE1	GLU	A	197	-14.736	-7.189	42.853	1.00	64.79	O
ATOM	1529	OE2	GLU	A	197	-16.170	-5.984	44.062	1.00	78.36	O
ATOM	1530	N	SER	A	198	-12.659	-7.857	45.436	1.00	65.34	N
ATOM	1531	CA	SER	A	198	-13.280	-9.172	45.735	1.00	62.22	C
ATOM	1532	C	SER	A	198	-12.196	-10.237	45.823	1.00	56.27	C
ATOM	1533	O	SER	A	198	-11.238	-10.093	46.595	1.00	63.34	O
ATOM	1534	CB	SER	A	198	-14.344	-9.482	44.728	1.00	63.64	C
ATOM	1535	OG	SER	A	198	-14.717	-10.795	44.877	1.00	68.69	O
ATOM	1536	N	ALA	A	199	-12.298	-11.329	45.079	1.00	58.94	N
ATOM	1537	CA	ALA	A	199	-11.233	-12.315	45.157	1.00	57.65	C
ATOM	1538	C	ALA	A	199	-9.919	-11.682	44.737	1.00	55.39	C

ATOM	1539	O	ALA	A	199	-8.857	-12.046	45.257	1.00	48.73	O
ATOM	1540	CB	ALA	A	199	-11.579	-13.543	44.308	1.00	50.50	C
ATOM	1541	N	GLY	A	200	-9.994	-10.687	43.845	1.00	54.60	N
ATOM	1542	CA	GLY	A	200	-8.811	-9.930	43.479	1.00	55.69	C
ATOM	1543	C	GLY	A	200	-8.230	-9.175	44.658	1.00	60.07	C
ATOM	1544	O	GLY	A	200	-7.036	-9.286	44.960	1.00	55.01	O
ATOM	1545	N	ALA	A	201	-9.070	-8.395	45.346	1.00	60.80	N
ATOM	1546	CA	ALA	A	201	-8.661	-7.838	46.632	1.00	58.74	C
ATOM	1547	C	ALA	A	201	-8.029	-8.908	47.526	1.00	68.52	C
ATOM	1548	O	ALA	A	201	-6.919	-8.735	48.029	1.00	66.13	O
ATOM	1549	CB	ALA	A	201	-9.857	-7.201	47.329	1.00	55.59	C
ATOM	1550	N	ALA	A	202	-8.687	-10.061	47.682	1.00	68.31	N
ATOM	1551	CA	ALA	A	202	-8.104	-11.119	48.499	1.00	50.88	C
ATOM	1552	C	ALA	A	202	-6.789	-11.611	47.901	1.00	53.63	C
ATOM	1553	O	ALA	A	202	-5.872	-11.999	48.634	1.00	55.31	O
ATOM	1554	CB	ALA	A	202	-9.110	-12.253	48.673	1.00	55.31	C
ATOM	1555	N	SER	A	203	-6.655	-11.574	46.575	1.00	58.71	N
ATOM	1556	CA	SER	A	203	-5.377	-11.963	45.987	1.00	67.86	C
ATOM	1557	C	SER	A	203	-4.279	-10.978	46.372	1.00	65.35	C
ATOM	1558	O	SER	A	203	-3.174	-11.387	46.751	1.00	66.29	O
ATOM	1559	CB	SER	A	203	-5.486	-12.074	44.464	1.00	65.22	C
ATOM	1560	OG	SER	A	203	-6.182	-13.245	44.080	1.00	51.63	O
ATOM	1561	N	VAL	A	204	-4.564	-9.675	46.302	1.00	62.95	N
ATOM	1562	CA	VAL	A	204	-3.482	-8.726	46.537	1.00	68.33	C
ATOM	1563	C	VAL	A	204	-3.097	-8.725	48.004	1.00	72.12	C
ATOM	1564	O	VAL	A	204	-1.908	-8.673	48.324	1.00	76.99	O
ATOM	1565	CB	VAL	A	204	-3.806	-7.321	45.984	1.00	68.94	C
ATOM	1566	CG1	VAL	A	204	-4.060	-7.435	44.453	1.00	64.02	C
ATOM	1567	CG2	VAL	A	204	-4.956	-6.557	46.797	1.00	60.34	C
ATOM	1568	N	SER	A	205	-4.053	-8.962	48.909	1.00	67.43	N
ATOM	1569	CA	SER	A	205	-3.688	-9.129	50.317	1.00	61.57	C
ATOM	1570	C	SER	A	205	-2.776	-10.346	50.511	1.00	70.94	C
ATOM	1571	O	SER	A	205	-1.834	-10.317	51.320	1.00	64.80	O
ATOM	1572	CB	SER	A	205	-4.947	-9.258	51.182	1.00	66.14	C
ATOM	1573	OG	SER	A	205	-5.307	-10.615	51.421	1.00	54.95	O
ATOM	1574	N	LEU	A	206	-3.031	-11.425	49.758	1.00	71.51	N
ATOM	1575	CA	LEU	A	206	-2.137	-12.574	49.834	1.00	64.54	C
ATOM	1576	C	LEU	A	206	-0.771	-12.249	49.271	1.00	69.68	C
ATOM	1577	O	LEU	A	206	0.224	-12.835	49.702	1.00	73.50	O
ATOM	1578	CB	LEU	A	206	-2.710	-13.782	49.105	1.00	57.65	C
ATOM	1579	CG	LEU	A	206	-3.786	-14.524	49.873	1.00	56.05	C
ATOM	1580	CD1	LEU	A	206	-4.166	-15.787	49.146	1.00	57.83	C
ATOM	1581	CD2	LEU	A	206	-3.275	-14.840	51.252	1.00	57.21	C
ATOM	1582	N	HIS	A	207	-0.693	-11.332	48.311	1.00	68.05	N

```
ATOM   1583  CA  HIS A 207       0.624 -10.956  47.839  1.00 68.37           C
ATOM   1584  C   HIS A 207       1.415 -10.260  48.928  1.00 81.48           C
ATOM   1585  O   HIS A 207       2.633 -10.456  49.020  1.00 89.83           O
ATOM   1586  CB  HIS A 207       0.506 -10.098  46.592  1.00 65.80           C
ATOM   1587  CG  HIS A 207       0.280 -10.907  45.362  1.00 66.36           C
ATOM   1588  ND1 HIS A 207       1.186 -11.853  44.933  1.00 68.72           N
ATOM   1589  CD2 HIS A 207      -0.741 -10.929  44.475  1.00 59.66           C
ATOM   1590  CE1 HIS A 207       0.735 -12.421  43.830  1.00 67.42           C
ATOM   1591  NE2 HIS A 207      -0.428 -11.872  43.526  1.00 64.56           N
ATOM   1592  N   LEU A 208       0.740  -9.490  49.790  1.00 76.79           N
ATOM   1593  CA  LEU A 208       1.443  -8.830  50.887  1.00 73.88           C
ATOM   1594  C   LEU A 208       2.146  -9.841  51.793  1.00 75.80           C
ATOM   1595  O   LEU A 208       3.256  -9.579  52.270  1.00 82.56           O
ATOM   1596  CB  LEU A 208       0.480  -7.951  51.684  1.00 64.71           C
ATOM   1597  CG  LEU A 208      -0.040  -6.701  50.979  1.00 58.31           C
ATOM   1598  CD1 LEU A 208      -1.138  -6.041  51.779  1.00 65.22           C
ATOM   1599  CD2 LEU A 208       1.099  -5.740  50.789  1.00 62.09           C
ATOM   1600  N   LEU A 209       1.541 -11.013  52.025  1.00 71.32           N
ATOM   1601  CA  LEU A 209       2.167 -11.998  52.907  1.00 75.04           C
ATOM   1602  C   LEU A 209       3.172 -12.909  52.213  1.00 80.88           C
ATOM   1603  O   LEU A 209       4.003 -13.513  52.900  1.00 84.67           O
ATOM   1604  CB  LEU A 209       1.121 -12.888  53.592  1.00 60.10           C
ATOM   1605  CG  LEU A 209      -0.163 -12.221  54.091  1.00 69.72           C
ATOM   1606  CD1 LEU A 209      -1.239 -13.251  54.351  1.00 70.53           C
ATOM   1607  CD2 LEU A 209       0.123 -11.470  55.339  1.00 77.09           C
ATOM   1608  N   SER A 210       3.123 -13.040  50.891  1.00 82.22           N
ATOM   1609  CA  SER A 210       3.974 -14.017  50.216  1.00 76.51           C
ATOM   1610  C   SER A 210       5.385 -13.472  50.051  1.00 83.51           C
ATOM   1611  O   SER A 210       5.577 -12.491  49.320  1.00 77.72           O
ATOM   1612  CB  SER A 210       3.413 -14.384  48.851  1.00 86.12           C
ATOM   1613  OG  SER A 210       4.222 -15.371  48.206  1.00 90.06           O
ATOM   1614  N   PRO A 211       6.395 -14.084  50.679  1.00 84.95           N
ATOM   1615  CA  PRO A 211       7.783 -13.634  50.456  1.00 87.49           C
ATOM   1616  C   PRO A 211       8.172 -13.558  48.984  1.00 85.08           C
ATOM   1617  O   PRO A 211       8.916 -12.646  48.601  1.00 82.11           O
ATOM   1618  CB  PRO A 211       8.612 -14.684  51.216  1.00 83.02           C
ATOM   1619  CG  PRO A 211       7.700 -15.890  51.335  1.00 79.83           C
ATOM   1620  CD  PRO A 211       6.325 -15.307  51.497  1.00 77.62           C
ATOM   1621  N   GLY A 212       7.674 -14.482  48.149  1.00 79.86           N
ATOM   1622  CA  GLY A 212       7.984 -14.480  46.730  1.00 79.15           C
ATOM   1623  C   GLY A 212       7.555 -13.218  46.005  1.00 85.49           C
ATOM   1624  O   GLY A 212       8.130 -12.883  44.957  1.00 79.64           O
ATOM   1625  N   SER A 213       6.571 -12.500  46.548  1.00 82.83           N
ATOM   1626  CA  SER A 213       6.012 -11.318  45.907  1.00 72.65           C
```

ATOM	1627	C	SER	A	213	6.433	-10.013	46.568	1.00	76.12	C
ATOM	1628	O	SER	A	213	5.950	-8.952	46.158	1.00	78.73	O
ATOM	1629	CB	SER	A	213	4.476	-11.399	45.897	1.00	74.53	C
ATOM	1630	OG	SER	A	213	4.012	-12.658	45.429	1.00	77.99	O
ATOM	1631	N	HIS	A	214	7.313	-10.043	47.573	1.00	77.39	N
ATOM	1632	CA	HIS	A	214	7.642	-8.832	48.328	0.57	81.61	C
ATOM	1634	C	HIS	A	214	8.366	-7.808	47.460	1.00	74.27	C
ATOM	1635	O	HIS	A	214	8.176	-6.597	47.611	1.00	77.94	O
ATOM	1636	CB	HIS	A	214	8.487	-9.196	49.557	0.57	78.76	C
ATOM	1638	CG	HIS	A	214	8.867	-8.021	50.412	0.57	79.15	C
ATOM	1640	ND1	HIS	A	214	9.629	-6.968	49.949	0.57	79.04	N
ATOM	1642	CD2	HIS	A	214	8.587	-7.736	51.706	0.57	79.87	C
ATOM	1644	CE1	HIS	A	214	9.796	-6.084	50.916	0.57	74.10	C
ATOM	1646	NE2	HIS	A	214	9.175	-6.527	51.993	0.57	82.14	N
ATOM	1648	N	SER	A	215	9.206	-8.267	46.549	1.00	69.00	N
ATOM	1649	CA	SER	A	215	10.037	-7.356	45.783	1.00	74.44	C
ATOM	1650	C	SER	A	215	9.451	-6.987	44.413	1.00	89.52	C
ATOM	1651	O	SER	A	215	10.124	-6.306	43.626	1.00	93.94	O
ATOM	1652	CB	SER	A	215	11.431	-7.963	45.627	1.00	77.85	C
ATOM	1653	OG	SER	A	215	12.097	-7.457	44.481	1.00	92.57	O
ATOM	1654	N	LEU	A	216	8.214	-7.384	44.108	1.00	82.38	N
ATOM	1655	CA	LEU	A	216	7.647	-7.104	42.793	1.00	75.36	C
ATOM	1656	C	LEU	A	216	6.612	-5.992	42.795	1.00	79.50	C
ATOM	1657	O	LEU	A	216	6.061	-5.689	41.733	1.00	80.98	O
ATOM	1658	CB	LEU	A	216	7.031	-8.367	42.183	1.00	72.71	C
ATOM	1659	CG	LEU	A	216	7.798	-9.687	42.341	1.00	70.20	C
ATOM	1660	CD1	LEU	A	216	6.888	-10.879	42.024	1.00	71.10	C
ATOM	1661	CD2	LEU	A	216	9.041	-9.701	41.465	1.00	77.30	C
ATOM	1662	N	PHE	A	217	6.330	-5.388	43.947	1.00	78.41	N
ATOM	1663	CA	PHE	A	217	5.424	-4.248	44.020	1.00	76.78	C
ATOM	1664	C	PHE	A	217	5.798	-3.407	45.235	1.00	85.44	C
ATOM	1665	O	PHE	A	217	6.665	-3.778	46.033	1.00	84.61	O
ATOM	1666	CB	PHE	A	217	3.947	-4.690	44.057	1.00	71.38	C
ATOM	1667	CG	PHE	A	217	3.510	-5.346	45.353	1.00	71.24	C
ATOM	1668	CD1	PHE	A	217	4.096	-6.521	45.795	1.00	75.94	C
ATOM	1669	CD2	PHE	A	217	2.450	-4.822	46.091	1.00	74.34	C
ATOM	1670	CE1	PHE	A	217	3.664	-7.133	46.984	1.00	77.78	C
ATOM	1671	CE2	PHE	A	217	2.009	-5.427	47.270	1.00	61.28	C
ATOM	1672	CZ	PHE	A	217	2.619	-6.581	47.715	1.00	69.95	C
ATOM	1673	N	THR	A	218	5.152	-2.244	45.353	1.00	85.58	N
ATOM	1674	CA	THR	A	218	5.384	-1.347	46.480	1.00	80.61	C
ATOM	1675	C	THR	A	218	4.155	-1.171	47.361	1.00	77.76	C
ATOM	1676	O	THR	A	218	4.235	-1.461	48.551	1.00	74.01	O
ATOM	1677	CB	THR	A	218	5.899	0.010	45.979	1.00	79.90	C

ATOM	1678	OG1	THR	A	218	4.791	0.852	45.614	1.00	79.89	O
ATOM	1679	CG2	THR	A	218	6.824	-0.202	44.774	1.00	87.01	C
ATOM	1680	N	ARG	A	219	3.012	-0.752	46.813	1.00	76.41	N
ATOM	1681	CA	ARG	A	219	1.815	-0.444	47.591	1.00	72.66	C
ATOM	1682	C	ARG	A	219	0.650	-1.346	47.199	1.00	74.17	C
ATOM	1683	O	ARG	A	219	0.700	-2.077	46.207	1.00	80.39	O
ATOM	1684	CB	ARG	A	219	1.410	1.021	47.410	1.00	78.39	C
ATOM	1685	CG	ARG	A	219	2.123	1.955	48.349	1.00	78.84	C
ATOM	1686	CD	ARG	A	219	1.667	3.384	48.182	1.00	82.37	C
ATOM	1687	NE	ARG	A	219	2.387	4.088	47.122	1.00	83.44	N
ATOM	1688	CZ	ARG	A	219	3.625	4.557	47.252	1.00	79.12	C
ATOM	1689	NH1	ARG	A	219	4.215	5.192	46.246	1.00	71.84	N
ATOM	1690	NH2	ARG	A	219	4.283	4.366	48.387	1.00	78.22	N
ATOM	1691	N	ALA	A	220	-0.436	-1.258	47.966	1.00	66.51	N
ATOM	1692	CA	ALA	A	220	-1.537	-2.199	47.817	1.00	68.10	C
ATOM	1693	C	ALA	A	220	-2.881	-1.494	47.927	1.00	69.61	C
ATOM	1694	O	ALA	A	220	-3.093	-0.685	48.835	1.00	69.51	O
ATOM	1695	CB	ALA	A	220	-1.443	-3.313	48.879	1.00	69.63	C
ATOM	1696	N	ILE	A	221	-3.796	-1.820	47.017	1.00	68.41	N
ATOM	1697	CA	ILE	A	221	-5.182	-1.374	47.117	1.00	71.87	C
ATOM	1698	C	ILE	A	221	-6.097	-2.598	47.181	1.00	78.88	C
ATOM	1699	O	ILE	A	221	-5.963	-3.537	46.380	1.00	69.61	O
ATOM	1700	CB	ILE	A	221	-5.579	-0.457	45.951	1.00	58.36	C
ATOM	1701	CG1	ILE	A	221	-4.758	0.830	45.984	1.00	65.37	C
ATOM	1702	CG2	ILE	A	221	-7.058	-0.095	46.054	1.00	55.39	C
ATOM	1703	CD1	ILE	A	221	-4.700	1.524	44.631	1.00	68.15	C
ATOM	1704	N	LEU	A	222	-7.032	-2.556	48.134	1.00	72.76	N
ATOM	1705	CA	LEU	A	222	-7.942	-3.631	48.503	1.00	59.00	C
ATOM	1706	C	LEU	A	222	-9.377	-3.159	48.391	1.00	68.21	C
ATOM	1707	O	LEU	A	222	-9.846	-2.418	49.254	1.00	74.25	O
ATOM	1708	CB	LEU	A	222	-7.676	-4.087	49.925	1.00	60.80	C
ATOM	1709	CG	LEU	A	222	-6.413	-4.883	50.063	1.00	65.37	C
ATOM	1710	CD1	LEU	A	222	-5.954	-5.015	51.477	1.00	80.19	C
ATOM	1711	CD2	LEU	A	222	-7.001	-6.210	49.630	1.00	79.26	C
ATOM	1712	N	GLN	A	223	-10.100	-3.595	47.369	1.00	65.62	N
ATOM	1713	CA	GLN	A	223	-11.498	-3.195	47.230	1.00	68.50	C
ATOM	1714	C	GLN	A	223	-12.406	-4.389	47.502	1.00	61.88	C
ATOM	1715	O	GLN	A	223	-12.382	-5.377	46.762	1.00	73.59	O
ATOM	1716	CB	GLN	A	223	-11.739	-2.617	45.844	1.00	69.02	C
ATOM	1717	CG	GLN	A	223	-10.518	-1.879	45.336	1.00	72.12	C
ATOM	1718	CD	GLN	A	223	-10.754	-1.093	44.061	1.00	62.08	C
ATOM	1719	OE1	GLN	A	223	-11.889	-0.947	43.596	1.00	57.67	O
ATOM	1720	NE2	GLN	A	223	-9.662	-0.624	43.457	1.00	64.19	N
ATOM	1721	N	SER	A	224	-13.184	-4.313	48.578	1.00	63.36	N

```
ATOM   1722  CA  SER A 224     -14.238  -5.298  48.847  1.00 64.83           C
ATOM   1723  C   SER A 224     -13.701  -6.730  48.973  1.00 64.85           C
ATOM   1724  O   SER A 224     -14.379  -7.692  48.625  1.00 64.12           O
ATOM   1725  CB  SER A 224     -15.302  -5.223  47.758  1.00 63.20           C
ATOM   1726  OG  SER A 224     -15.482  -3.865  47.371  1.00 65.87           O
ATOM   1727  N   GLY A 225     -12.474  -6.886  49.468  1.00 62.68           N
ATOM   1728  CA  GLY A 225     -11.947  -8.213  49.728  1.00 53.58           C
ATOM   1729  C   GLY A 225     -10.654  -8.236  50.531  1.00 69.28           C
ATOM   1730  O   GLY A 225      -9.873  -7.279  50.485  1.00 71.42           O
ATOM   1731  N   SER A 226     -10.416  -9.334  51.256  1.00 66.61           N
ATOM   1732  CA  SER A 226      -9.238  -9.558  52.088  1.00 58.62           C
ATOM   1733  C   SER A 226      -9.167 -11.029  52.473  1.00 60.21           C
ATOM   1734  O   SER A 226     -10.198 -11.682  52.643  1.00 55.14           O
ATOM   1735  CB  SER A 226      -9.304  -8.696  53.333  1.00 60.72           C
ATOM   1736  OG  SER A 226     -10.610  -8.814  53.852  1.00 60.71           O
ATOM   1737  N   PHE A 227      -7.943 -11.535  52.647  1.00 57.58           N
ATOM   1738  CA  PHE A 227      -7.760 -12.977  52.805  1.00 64.49           C
ATOM   1739  C   PHE A 227      -8.456 -13.550  54.038  1.00 73.65           C
ATOM   1740  O   PHE A 227      -8.758 -14.755  54.069  1.00 67.28           O
ATOM   1741  CB  PHE A 227      -6.270 -13.326  52.866  1.00 69.44           C
ATOM   1742  CG  PHE A 227      -5.581 -12.913  54.150  1.00 75.67           C
ATOM   1743  CD1 PHE A 227      -4.759 -11.795  54.175  1.00 73.77           C
ATOM   1744  CD2 PHE A 227      -5.713 -13.668  55.317  1.00 75.27           C
ATOM   1745  CE1 PHE A 227      -4.107 -11.428  55.335  1.00 71.33           C
ATOM   1746  CE2 PHE A 227      -5.077 -13.297  56.475  1.00 69.07           C
ATOM   1747  CZ  PHE A 227      -4.267 -12.183  56.475  1.00 74.69           C
ATOM   1748  N   ASN A 228      -8.665 -12.737  55.072  1.00 64.52           N
ATOM   1749  CA  ASN A 228      -9.293 -13.215  56.292  1.00 51.68           C
ATOM   1750  C   ASN A 228     -10.803 -13.342  56.170  1.00 57.40           C
ATOM   1751  O   ASN A 228     -11.450 -13.740  57.143  1.00 71.89           O
ATOM   1752  CB  ASN A 228      -8.986 -12.269  57.436  1.00 57.33           C
ATOM   1753  CG  ASN A 228      -9.598 -10.911  57.205  1.00 75.20           C
ATOM   1754  OD1 ASN A 228      -9.615 -10.402  56.074  1.00 75.33           O
ATOM   1755  ND2 ASN A 228     -10.147 -10.334  58.252  1.00 71.32           N
ATOM   1756  N   ALA A 229     -11.390 -12.975  55.036  1.00 59.87           N
ATOM   1757  CA  ALA A 229     -12.817 -13.172  54.868  1.00 58.46           C
ATOM   1758  C   ALA A 229     -13.136 -14.665  54.907  1.00 51.21           C
ATOM   1759  O   ALA A 229     -12.335 -15.489  54.460  1.00 50.04           O
ATOM   1760  CB  ALA A 229     -13.296 -12.560  53.554  1.00 55.76           C
ATOM   1761  N   PRO A 230     -14.296 -15.038  55.437  1.00 48.52           N
ATOM   1762  CA  PRO A 230     -14.586 -16.468  55.631  1.00 55.09           C
ATOM   1763  C   PRO A 230     -14.477 -17.293  54.368  1.00 47.37           C
ATOM   1764  O   PRO A 230     -14.093 -18.464  54.459  1.00 54.11           O
ATOM   1765  CB  PRO A 230     -16.021 -16.465  56.186  1.00 58.48           C
```

ATOM	1766	CG	PRO	A	230	-16.608	-15.186	55.686	1.00	58.52	C
ATOM	1767	CD	PRO	A	230	-15.466	-14.196	55.730	1.00	54.70	C
ATOM	1768	N	TRP	A	231	-14.771	-16.714	53.200	1.00	43.15	N
ATOM	1769	CA	TRP	A	231	-14.781	-17.397	51.906	1.00	42.93	C
ATOM	1770	C	TRP	A	231	-13.418	-17.479	51.222	1.00	47.07	C
ATOM	1771	O	TRP	A	231	-13.352	-17.960	50.093	1.00	48.16	O
ATOM	1772	CB	TRP	A	231	-15.739	-16.689	50.919	1.00	51.80	C
ATOM	1773	CG	TRP	A	231	-15.529	-15.153	50.814	1.00	57.89	C
ATOM	1774	CD1	TRP	A	231	-16.183	-14.186	51.543	1.00	60.09	C
ATOM	1775	CD2	TRP	A	231	-14.610	-14.443	49.969	1.00	53.56	C
ATOM	1776	NE1	TRP	A	231	-15.730	-12.939	51.204	1.00	55.26	N
ATOM	1777	CE2	TRP	A	231	-14.775	-13.064	50.233	1.00	51.82	C
ATOM	1778	CE3	TRP	A	231	-13.678	-14.833	49.007	1.00	62.06	C
ATOM	1779	CZ2	TRP	A	231	-14.033	-12.078	49.582	1.00	48.64	C
ATOM	1780	CZ3	TRP	A	231	-12.945	-13.841	48.346	1.00	59.73	C
ATOM	1781	CH2	TRP	A	231	-13.124	-12.484	48.647	1.00	56.17	C
ATOM	1782	N	ALA	A	232	-12.339	-16.991	51.823	1.00	52.61	N
ATOM	1783	CA	ALA	A	232	-11.172	-16.709	50.992	1.00	43.94	C
ATOM	1784	C	ALA	A	232	-10.109	-17.790	51.024	1.00	52.51	C
ATOM	1785	O	ALA	A	232	-9.507	-18.068	49.982	1.00	54.36	O
ATOM	1786	CB	ALA	A	232	-10.535	-15.374	51.382	1.00	55.34	C
ATOM	1787	N	VAL	A	233	-9.836	-18.402	52.176	1.00	58.98	N
ATOM	1788	CA	VAL	A	233	-8.793	-19.422	52.269	1.00	61.76	C
ATOM	1789	C	VAL	A	233	-9.415	-20.755	52.671	1.00	61.34	C
ATOM	1790	O	VAL	A	233	-10.258	-20.810	53.575	1.00	54.43	O
ATOM	1791	CB	VAL	A	233	-7.676	-19.015	53.244	1.00	52.41	C
ATOM	1792	CG1	VAL	A	233	-6.666	-20.154	53.372	1.00	59.86	C
ATOM	1793	CG2	VAL	A	233	-7.006	-17.727	52.759	1.00	49.76	C
ATOM	1794	N	THR	A	234	-9.013	-21.821	51.975	1.00	71.08	N
ATOM	1795	CA	THR	A	234	-9.500	-23.173	52.221	1.00	69.32	C
ATOM	1796	C	THR	A	234	-8.439	-23.981	52.954	1.00	66.70	C
ATOM	1797	O	THR	A	234	-7.297	-24.074	52.496	1.00	56.91	O
ATOM	1798	CB	THR	A	234	-9.870	-23.879	50.916	1.00	56.36	C
ATOM	1799	OG1	THR	A	234	-10.771	-23.060	50.161	1.00	60.47	O
ATOM	1800	CG2	THR	A	234	-10.520	-25.221	51.221	1.00	69.73	C
ATOM	1801	N	SER	A	235	-8.819	-24.574	54.081	1.00	71.27	N
ATOM	1802	CA	SER	A	235	-7.855	-25.334	54.855	1.00	72.07	C
ATOM	1803	C	SER	A	235	-7.538	-26.654	54.166	1.00	71.94	C
ATOM	1804	O	SER	A	235	-8.343	-27.197	53.405	1.00	79.85	O
ATOM	1805	CB	SER	A	235	-8.377	-25.581	56.266	1.00	79.18	C
ATOM	1806	OG	SER	A	235	-9.386	-26.564	56.259	1.00	85.77	O
ATOM	1807	N	LEU	A	236	-6.346	-27.172	54.450	1.00	77.66	N
ATOM	1808	CA	LEU	A	236	-5.849	-28.381	53.801	1.00	77.28	C
ATOM	1809	C	LEU	A	236	-6.774	-29.580	53.918	1.00	74.74	C

ATOM	1810	O	LEU	A	236	-6.700	-30.472	53.076	1.00	71.08	O
ATOM	1811	CB	LEU	A	236	-4.499	-28.768	54.376	1.00	77.71	C
ATOM	1812	CG	LEU	A	236	-3.471	-27.654	54.177	1.00	89.84	C
ATOM	1813	CD1	LEU	A	236	-2.134	-28.106	54.745	1.00	95.22	C
ATOM	1814	CD2	LEU	A	236	-3.375	-27.301	52.701	1.00	91.18	C
ATOM	1815	N	TYR	A	237	-7.624	-29.653	54.932	1.00	85.40	N
ATOM	1816	CA	TYR	A	237	-8.497	-30.818	55.013	1.00	89.11	C
ATOM	1817	C	TYR	A	237	-9.560	-30.767	53.918	1.00	82.65	C
ATOM	1818	O	TYR	A	237	-9.773	-31.761	53.210	1.00	82.64	O
ATOM	1819	CB	TYR	A	237	-9.123	-30.944	56.411	1.00	83.84	C
ATOM	1820	CG	TYR	A	237	-10.068	-32.122	56.540	0.98	81.89	C
ATOM	1821	CD1	TYR	A	237	-9.584	-33.420	56.709	1.00	86.51	C
ATOM	1822	CD2	TYR	A	237	-11.447	-31.934	56.494	1.00	79.15	C
ATOM	1823	CE1	TYR	A	237	-10.457	-34.506	56.823	1.00	91.11	C
ATOM	1824	CE2	TYR	A	237	-12.324	-33.007	56.600	1.00	88.51	C
ATOM	1825	CZ	TYR	A	237	-11.827	-34.290	56.768	0.78	94.13	C
ATOM	1826	OH	TYR	A	237	-12.708	-35.349	56.877	1.00	92.91	O
ATOM	1827	N	GLU	A	238	-10.216	-29.608	53.738	1.00	71.57	N
ATOM	1828	CA	GLU	A	238	-11.255	-29.518	52.712	1.00	78.48	C
ATOM	1829	C	GLU	A	238	-10.640	-29.504	51.323	1.00	81.31	C
ATOM	1830	O	GLU	A	238	-11.119	-30.206	50.423	1.00	83.19	O
ATOM	1831	CB	GLU	A	238	-12.145	-28.288	52.914	1.00	76.26	C
ATOM	1832	CG	GLU	A	238	-12.836	-28.224	54.270	1.00	79.58	C
ATOM	1833	CD	GLU	A	238	-11.880	-27.774	55.358	1.00	97.71	C
ATOM	1834	OE1	GLU	A	238	-12.134	-28.013	56.570	1.00	95.96	O
ATOM	1835	OE2	GLU	A	238	-10.851	-27.166	54.979	1.00	96.12	O
ATOM	1836	N	ALA	A	239	-9.575	-28.722	51.138	1.00	69.02	N
ATOM	1837	CA	ALA	A	239	-8.769	-28.790	49.926	1.00	67.10	C
ATOM	1838	C	ALA	A	239	-8.581	-30.222	49.412	1.00	76.63	C
ATOM	1839	O	ALA	A	239	-8.898	-30.527	48.256	1.00	78.25	O
ATOM	1840	CB	ALA	A	239	-7.415	-28.140	50.198	1.00	68.40	C
ATOM	1841	N	ARG	A	240	-8.056	-31.116	50.256	1.00	73.44	N
ATOM	1842	CA	ARG	A	240	-7.945	-32.512	49.851	1.00	66.18	C
ATOM	1843	C	ARG	A	240	-9.315	-33.153	49.713	1.00	70.54	C
ATOM	1844	O	ARG	A	240	-9.524	-33.989	48.823	1.00	66.26	O
ATOM	1845	CB	ARG	A	240	-7.115	-33.305	50.855	1.00	83.62	C
ATOM	1846	CG	ARG	A	240	-5.671	-32.919	50.964	1.00	83.41	C
ATOM	1847	CD	ARG	A	240	-4.922	-34.050	51.635	1.00	88.63	C
ATOM	1848	NE	ARG	A	240	-5.425	-35.361	51.210	1.00	95.46	N
ATOM	1849	CZ	ARG	A	240	-4.817	-36.142	50.317	1.00	82.49	C
ATOM	1850	NH1	ARG	A	240	-3.680	-35.750	49.756	1.00	82.00	N
ATOM	1851	NH2	ARG	A	240	-5.331	-37.319	49.994	1.00	76.68	N
ATOM	1852	N	ASN	A	241	-10.259	-32.799	50.604	1.00	74.41	N
ATOM	1853	CA	ASN	A	241	-11.564	-33.449	50.557	1.00	73.22	C

ATOM	1854	C	ASN	A	241	-12.301	-33.069	49.295	1.00 69.87	C
ATOM	1855	O	ASN	A	241	-13.216	-33.785	48.893	1.00 76.06	O
ATOM	1856	CB	ASN	A	241	-12.429	-33.130	51.794	1.00 73.95	C
ATOM	1857	CG	ASN	A	241	-13.616	-34.120	51.975	1.00 75.41	C
ATOM	1858	OD1	ASN	A	241	-13.417	-35.333	52.017	1.00 70.85	O
ATOM	1859	ND2	ASN	A	241	-14.837	-33.594	52.111	1.00 78.45	N
ATOM	1860	N	ARG	A	242	-11.890	-32.000	48.624	1.00 72.16	N
ATOM	1861	CA	ARG	A	242	-12.530	-31.643	47.369	1.00 68.14	C
ATOM	1862	C	ARG	A	242	-11.704	-31.974	46.136	1.00 62.08	C
ATOM	1863	O	ARG	A	242	-12.281	-32.128	45.060	1.00 65.50	O
ATOM	1864	CB	ARG	A	242	-12.916	-30.166	47.386	1.00 60.52	C
ATOM	1865	CG	ARG	A	242	-13.691	-29.812	48.649	1.00 67.63	C
ATOM	1866	CD	ARG	A	242	-14.538	-28.562	48.515	1.00 61.59	C
ATOM	1867	NE	ARG	A	242	-13.793	-27.376	48.924	1.00 62.49	N
ATOM	1868	CZ	ARG	A	242	-14.207	-26.492	49.825	1.00 67.71	C
ATOM	1869	NH1	ARG	A	242	-15.381	-26.641	50.421	1.00 71.84	N
ATOM	1870	NH2	ARG	A	242	-13.452	-25.443	50.117	1.00 68.68	N
ATOM	1871	N	THR	A	243	-10.384	-32.121	46.248	1.00 67.03	N
ATOM	1872	CA	THR	A	243	-9.626	-32.704	45.141	1.00 68.82	C
ATOM	1873	C	THR	A	243	-9.997	-34.170	44.966	1.00 70.12	C
ATOM	1874	O	THR	A	243	-10.576	-34.561	43.945	1.00 67.66	O
ATOM	1875	CB	THR	A	243	-8.114	-32.568	45.369	1.00 73.63	C
ATOM	1876	OG1	THR	A	243	-7.726	-31.186	45.352	1.00 74.54	O
ATOM	1877	CG2	THR	A	243	-7.356	-33.316	44.292	1.00 60.09	C
ATOM	1878	N	LEU	A	244	-9.675	-34.999	45.971	1.00 71.48	N
ATOM	1879	CA	LEU	A	244	-10.136	-36.388	45.976	1.00 81.05	C
ATOM	1880	C	LEU	A	244	-11.622	-36.519	45.699	1.00 77.41	C
ATOM	1881	O	LEU	A	244	-12.054	-37.528	45.119	1.00 76.43	O
ATOM	1882	CB	LEU	A	244	-9.838	-37.073	47.302	1.00 78.63	C
ATOM	1883	CG	LEU	A	244	-8.426	-37.012	47.828	1.00 81.26	C
ATOM	1884	CD1	LEU	A	244	-8.459	-37.607	49.230	1.00 83.35	C
ATOM	1885	CD2	LEU	A	244	-7.542	-37.796	46.863	1.00 65.79	C
ATOM	1886	N	ASN	A	245	-12.425	-35.551	46.116	1.00 68.74	N
ATOM	1887	CA	ASN	A	245	-13.786	-35.569	45.630	1.00 68.44	C
ATOM	1888	C	ASN	A	245	-13.806	-35.436	44.114	1.00 69.65	C
ATOM	1889	O	ASN	A	245	-14.212	-36.352	43.408	1.00 77.17	O
ATOM	1890	CB	ASN	A	245	-14.610	-34.499	46.328	1.00 73.03	C
ATOM	1891	CG	ASN	A	245	-15.326	-35.060	47.539	1.00 68.94	C
ATOM	1892	OD1	ASN	A	245	-15.012	-36.163	47.971	1.00 70.98	O
ATOM	1893	ND2	ASN	A	245	-16.298	-34.327	48.072	1.00 73.47	N
ATOM	1894	N	LEU	A	246	-13.369	-34.307	43.588	1.00 73.56	N
ATOM	1895	CA	LEU	A	246	-13.476	-34.115	42.140	1.00 85.74	C
ATOM	1896	C	LEU	A	246	-13.086	-35.395	41.408	1.00 81.56	C
ATOM	1897	O	LEU	A	246	-13.769	-35.818	40.468	1.00 80.94	O

```
ATOM   1898  CB  LEU A 246     -12.611 -32.944  41.635  1.00 71.36           C
ATOM   1899  CG  LEU A 246     -13.242 -32.159  40.486  1.00 55.49           C
ATOM   1900  CD1 LEU A 246     -12.356 -31.028  40.182  1.00 56.48           C
ATOM   1901  CD2 LEU A 246     -13.427 -32.971  39.244  1.00 82.16           C
ATOM   1902  N   ALA A 247     -12.053 -36.069  41.917  1.00 76.24           N
ATOM   1903  CA  ALA A 247     -11.689 -37.384  41.405  1.00 78.11           C
ATOM   1904  C   ALA A 247     -12.872 -38.356  41.331  1.00 75.94           C
ATOM   1905  O   ALA A 247     -12.971 -39.126  40.369  1.00 84.59           O
ATOM   1906  CB  ALA A 247     -10.567 -37.978  42.254  1.00 80.01           C
ATOM   1907  N   LYS A 248     -13.777 -38.374  42.319  1.00 75.98           N
ATOM   1908  CA  LYS A 248     -14.773 -39.445  42.237  1.00 79.71           C
ATOM   1909  C   LYS A 248     -15.804 -39.143  41.143  1.00 75.47           C
ATOM   1910  O   LYS A 248     -16.275 -40.059  40.457  1.00 84.04           O
ATOM   1911  CB  LYS A 248     -15.431 -39.775  43.609  1.00 64.93           C
ATOM   1912  CG  LYS A 248     -16.630 -38.988  44.225  1.00 81.63           C
ATOM   1913  CD  LYS A 248     -16.460 -37.487  44.273  1.00 92.87           C
ATOM   1914  CE  LYS A 248     -17.528 -36.647  45.001  1.00 88.87           C
ATOM   1915  NZ  LYS A 248     -17.061 -35.207  44.886  1.00 84.86           N
ATOM   1916  N   LEU A 249     -16.121 -37.880  40.894  1.00 74.19           N
ATOM   1917  CA  LEU A 249     -17.174 -37.658  39.912  1.00 74.69           C
ATOM   1918  C   LEU A 249     -16.634 -37.557  38.494  1.00 77.14           C
ATOM   1919  O   LEU A 249     -17.420 -37.394  37.562  1.00 74.93           O
ATOM   1920  CB  LEU A 249     -17.960 -36.378  40.193  1.00 73.83           C
ATOM   1921  CG  LEU A 249     -18.520 -36.009  41.530  1.00 62.89           C
ATOM   1922  CD1 LEU A 249     -17.326 -35.483  42.209  1.00 63.92           C
ATOM   1923  CD2 LEU A 249     -19.662 -34.979  41.506  1.00 68.27           C
ATOM   1924  N   THR A 250     -15.321 -37.600  38.301  1.00 76.01           N
ATOM   1925  CA  THR A 250     -14.750 -37.700  36.967  1.00 73.71           C
ATOM   1926  C   THR A 250     -14.187 -39.090  36.693  1.00 82.95           C
ATOM   1927  O   THR A 250     -13.430 -39.261  35.727  1.00 87.75           O
ATOM   1928  CB  THR A 250     -13.683 -36.625  36.766  1.00 77.84           C
ATOM   1929  OG1 THR A 250     -12.681 -36.758  37.778  1.00 81.87           O
ATOM   1930  CG2 THR A 250     -14.325 -35.293  36.868  1.00 60.89           C
ATOM   1931  N   GLY A 251     -14.553 -40.080  37.506  1.00 78.47           N
ATOM   1932  CA  GLY A 251     -14.053 -41.432  37.337  1.00 75.43           C
ATOM   1933  C   GLY A 251     -12.548 -41.515  37.429  1.00 78.20           C
ATOM   1934  O   GLY A 251     -11.929 -42.281  36.686  1.00 89.89           O
ATOM   1935  N   CYS A 252     -11.936 -40.739  38.317  1.00 84.06           N
ATOM   1936  CA  CYS A 252     -10.487 -40.772  38.457  1.00 92.87           C
ATOM   1937  C   CYS A 252     -10.018 -41.377  39.774  1.00 92.73           C
ATOM   1938  O   CYS A 252      -8.839 -41.620  39.905  1.00100.53           O
ATOM   1939  CB  CYS A 252      -9.876 -39.383  38.268  1.00 83.90           C
ATOM   1940  SG  CYS A 252      -9.679 -38.907  36.492  1.00 81.18           S
ATOM   1941  N   SER A 253     -10.900 -41.702  40.719  1.00 78.17           N
```

```
ATOM   1942  CA  SER A 253     -10.520 -42.219  42.041  1.00 86.61           C
ATOM   1943  C   SER A 253      -9.506 -43.366  42.003  1.00 88.57           C
ATOM   1944  O   SER A 253      -9.890 -44.505  41.743  1.00 91.26           O
ATOM   1945  CB  SER A 253     -11.765 -42.681  42.814  1.00 86.61           C
ATOM   1946  OG  SER A 253     -12.566 -41.581  43.175  1.00 88.01           O
ATOM   1947  N   ARG A 254      -8.216 -43.093  42.280  1.00 85.55           N
ATOM   1948  CA  ARG A 254      -7.166 -44.117  42.394  1.00 88.07           C
ATOM   1949  C   ARG A 254      -6.496 -44.167  43.766  1.00 91.12           C
ATOM   1950  O   ARG A 254      -6.908 -43.498  44.721  1.00 94.32           O
ATOM   1951  CB  ARG A 254      -6.031 -43.972  41.346  1.00 90.98           C
ATOM   1952  CG  ARG A 254      -6.426 -43.526  39.949  1.00 85.55           C
ATOM   1953  CD  ARG A 254      -7.743 -44.180  39.551  1.00 91.10           C
ATOM   1954  NE  ARG A 254      -8.172 -43.911  38.176  1.00 83.01           N
ATOM   1955  CZ  ARG A 254      -9.350 -44.309  37.682  1.00 90.69           C
ATOM   1956  NH1 ARG A 254      -9.661 -44.016  36.425  1.00 86.03           N
ATOM   1957  NH2 ARG A 254     -10.254 -44.881  38.485  1.00 89.43           N
ATOM   1958  N   GLU A 255      -5.444 -44.980  43.845  1.00 91.56           N
ATOM   1959  CA  GLU A 255      -4.741 -45.347  45.064  1.00104.53           C
ATOM   1960  C   GLU A 255      -3.496 -44.512  45.276  1.00 97.78           C
ATOM   1961  O   GLU A 255      -3.045 -44.368  46.419  1.00107.64           O
ATOM   1962  CB  GLU A 255      -4.340 -46.820  44.995  1.00114.46           C
ATOM   1963  CG  GLU A 255      -5.494 -47.746  44.668  1.00112.25           C
ATOM   1964  CD  GLU A 255      -6.220 -48.225  45.896  1.00108.75           C
ATOM   1965  OE1 GLU A 255      -7.161 -47.521  46.340  1.00104.20           O
ATOM   1966  OE2 GLU A 255      -5.860 -49.315  46.408  1.00108.52           O
ATOM   1967  N   ASN A 256      -2.919 -44.007  44.183  1.00 90.92           N
ATOM   1968  CA  ASN A 256      -1.601 -43.390  44.202  1.00 89.64           C
ATOM   1969  C   ASN A 256      -1.799 -41.973  43.688  1.00 96.46           C
ATOM   1970  O   ASN A 256      -2.112 -41.786  42.509  1.00 98.38           O
ATOM   1971  CB  ASN A 256      -0.632 -44.163  43.313  1.00 98.63           C
ATOM   1972  CG  ASN A 256       0.819 -43.769  43.527  1.00107.55           C
ATOM   1973  OD1 ASN A 256       1.123 -42.848  44.283  1.00113.88           O
ATOM   1974  ND2 ASN A 256       1.730 -44.454  42.823  1.00 98.84           N
ATOM   1975  N   GLU A 257      -1.637 -40.979  44.569  1.00101.75           N
ATOM   1976  CA  GLU A 257      -1.822 -39.568  44.223  1.00 86.88           C
ATOM   1977  C   GLU A 257      -1.142 -39.254  42.900  1.00 89.99           C
ATOM   1978  O   GLU A 257      -1.702 -38.541  42.070  1.00 90.16           O
ATOM   1979  CB  GLU A 257      -1.290 -38.639  45.341  1.00 92.86           C
ATOM   1980  CG  GLU A 257      -2.356 -38.010  46.291  1.00 93.43           C
ATOM   1981  CD  GLU A 257      -1.761 -37.275  47.517  1.00 95.56           C
ATOM   1982  OE1 GLU A 257      -0.595 -36.824  47.429  1.00 97.43           O
ATOM   1983  OE2 GLU A 257      -2.448 -37.184  48.574  1.00 84.68           O
ATOM   1984  N   THR A 258       0.034 -39.819  42.654  1.00 96.33           N
ATOM   1985  CA  THR A 258       0.724 -39.488  41.412  1.00 91.55           C
```

```
ATOM   1986  C   THR A 258       0.095 -40.107  40.170  1.00 90.15           C
ATOM   1987  O   THR A 258       0.758 -40.181  39.128  1.00 88.37           O
ATOM   1988  CB  THR A 258       2.192 -39.917  41.441  0.44 98.19           C
ATOM   1989  OG1 THR A 258       2.761 -39.676  40.150  0.33 95.46           O
ATOM   1990  CG2 THR A 258       2.314 -41.433  41.733  0.84104.22           C
ATOM   1991  N   GLU A 259      -1.159 -40.562  40.255  1.00 95.16           N
ATOM   1992  CA  GLU A 259      -1.871 -40.957  39.044  1.00 94.06           C
ATOM   1993  C   GLU A 259      -3.351 -40.602  39.047  1.00 86.62           C
ATOM   1994  O   GLU A 259      -3.970 -40.675  37.978  1.00 84.51           O
ATOM   1995  CB  GLU A 259      -1.688 -42.466  38.763  0.80 99.33           C
ATOM   1996  CG  GLU A 259      -0.390 -42.715  38.000  0.61101.88           C
ATOM   1997  CD  GLU A 259      -0.463 -43.744  36.907  1.00116.96           C
ATOM   1998  OE1 GLU A 259       0.608 -44.034  36.325  1.00120.12           O
ATOM   1999  OE2 GLU A 259      -1.561 -44.266  36.630  1.00 92.58           O
ATOM   2000  N   ILE A 260      -3.946 -40.246  40.195  1.00 84.02           N
ATOM   2001  CA  ILE A 260      -5.150 -39.411  40.198  1.00 81.52           C
ATOM   2002  C   ILE A 260      -4.915 -38.217  39.294  1.00 77.14           C
ATOM   2003  O   ILE A 260      -5.785 -37.821  38.511  1.00 69.38           O
ATOM   2004  CB  ILE A 260      -5.492 -38.946  41.641  1.00 92.06           C
ATOM   2005  CG1 ILE A 260      -6.999 -38.877  41.953  1.00 86.86           C
ATOM   2006  CG2 ILE A 260      -4.953 -37.539  41.937  1.00 95.81           C
ATOM   2007  CD1 ILE A 260      -7.716 -40.119  42.013  1.00 98.53           C
ATOM   2008  N   ILE A 261      -3.708 -37.671  39.346  1.00 83.47           N
ATOM   2009  CA  ILE A 261      -3.432 -36.396  38.721  1.00 84.18           C
ATOM   2010  C   ILE A 261      -3.288 -36.578  37.226  1.00 86.31           C
ATOM   2011  O   ILE A 261      -3.833 -35.798  36.436  1.00 88.49           O
ATOM   2012  CB  ILE A 261      -2.191 -35.773  39.379  1.00 84.38           C
ATOM   2013  CG1 ILE A 261      -2.556 -35.452  40.816  1.00 79.13           C
ATOM   2014  CG2 ILE A 261      -1.830 -34.486  38.729  1.00 71.07           C
ATOM   2015  CD1 ILE A 261      -3.862 -34.637  40.829  1.00 87.49           C
ATOM   2016  N   LYS A 262      -2.619 -37.661  36.820  1.00 87.06           N
ATOM   2017  CA  LYS A 262      -2.491 -37.963  35.401  1.00 80.78           C
ATOM   2018  C   LYS A 262      -3.838 -38.342  34.796  1.00 75.39           C
ATOM   2019  O   LYS A 262      -4.148 -37.944  33.670  1.00 68.68           O
ATOM   2020  CB  LYS A 262      -1.441 -39.053  35.189  1.00 84.36           C
ATOM   2021  CG  LYS A 262      -0.113 -38.755  35.909  1.00 96.59           C
ATOM   2022  CD  LYS A 262       0.959 -39.821  35.637  1.00106.55           C
ATOM   2023  CE  LYS A 262       0.969 -40.259  34.167  1.00104.08           C
ATOM   2024  NZ  LYS A 262       2.336 -40.283  33.567  1.00105.30           N
ATOM   2025  N   CYS A 263      -4.674 -39.067  35.543  1.00 79.38           N
ATOM   2026  CA  CYS A 263      -6.048 -39.289  35.091  1.00 77.55           C
ATOM   2027  C   CYS A 263      -6.780 -37.971  34.875  1.00 70.62           C
ATOM   2028  O   CYS A 263      -7.438 -37.780  33.847  1.00 66.48           O
ATOM   2029  CB  CYS A 263      -6.819 -40.151  36.095  1.00 79.44           C
```

```
ATOM   2030  SG  CYS A 263      -8.595 -40.444  35.685  1.00 89.15           S
ATOM   2031  N   LEU A 264      -6.674 -37.047  35.835  1.00 82.27           N
ATOM   2032  CA  LEU A 264      -7.414 -35.793  35.743  1.00 76.02           C
ATOM   2033  C   LEU A 264      -6.978 -34.918  34.576  1.00 65.75           C
ATOM   2034  O   LEU A 264      -7.787 -34.109  34.123  1.00 76.54           O
ATOM   2035  CB  LEU A 264      -7.320 -34.984  37.043  1.00 75.06           C
ATOM   2036  CG  LEU A 264      -8.303 -35.274  38.186  1.00 68.92           C
ATOM   2037  CD1 LEU A 264      -7.972 -34.431  39.416  1.00 54.26           C
ATOM   2038  CD2 LEU A 264      -9.737 -35.026  37.747  1.00 65.11           C
ATOM   2039  N   ARG A 265      -5.746 -35.057  34.058  1.00 65.20           N
ATOM   2040  CA  ARG A 265      -5.336 -34.211  32.929  1.00 76.15           C
ATOM   2041  C   ARG A 265      -5.726 -34.809  31.565  1.00 79.41           C
ATOM   2042  O   ARG A 265      -5.742 -34.073  30.568  1.00 87.29           O
ATOM   2043  CB  ARG A 265      -3.836 -33.867  33.030  1.00 63.19           C
ATOM   2044  CG  ARG A 265      -2.884 -35.027  33.230  1.00 83.28           C
ATOM   2045  CD  ARG A 265      -1.816 -34.740  34.318  1.00 83.56           C
ATOM   2046  NE  ARG A 265      -1.095 -33.459  34.145  1.00 87.00           N
ATOM   2047  CZ  ARG A 265      -0.691 -32.669  35.156  1.00 92.82           C
ATOM   2048  NH1 ARG A 265      -0.027 -31.569  34.886  1.00 96.12           N
ATOM   2049  NH2 ARG A 265      -0.994 -32.948  36.420  1.00 89.40           N
ATOM   2050  N   ASN A 266      -6.095 -36.099  31.498  1.00 73.68           N
ATOM   2051  CA  ASN A 266      -6.723 -36.645  30.292  1.00 82.26           C
ATOM   2052  C   ASN A 266      -8.235 -36.438  30.250  1.00 83.44           C
ATOM   2053  O   ASN A 266      -8.870 -36.805  29.249  1.00 80.15           O
ATOM   2054  CB  ASN A 266      -6.452 -38.149  30.121  1.00 72.60           C
ATOM   2055  CG  ASN A 266      -4.977 -38.505  30.144  1.00 89.66           C
ATOM   2056  OD1 ASN A 266      -4.305 -38.397  31.167  1.00 96.00           O
ATOM   2057  ND2 ASN A 266      -4.451 -38.894  28.975  1.00100.52           N
ATOM   2058  N   LYS A 267      -8.836 -35.900  31.310  1.00 77.97           N
ATOM   2059  CA  LYS A 267     -10.233 -35.504  31.228  1.00 82.47           C
ATOM   2060  C   LYS A 267     -10.306 -34.169  30.503  1.00 82.17           C
ATOM   2061  O   LYS A 267      -9.493 -33.270  30.746  1.00 75.32           O
ATOM   2062  CB  LYS A 267     -10.885 -35.435  32.618  1.00 75.30           C
ATOM   2063  CG  LYS A 267     -11.515 -36.765  33.091  1.00 75.12           C
ATOM   2064  CD  LYS A 267     -12.696 -37.171  32.166  1.00 92.54           C
ATOM   2065  CE  LYS A 267     -12.287 -38.283  31.121  1.00103.28           C
ATOM   2066  NZ  LYS A 267     -13.184 -38.445  29.911  1.00 73.10           N
ATOM   2067  N   ASP A 268     -11.239 -34.057  29.560  1.00 81.28           N
ATOM   2068  CA  ASP A 268     -11.267 -32.838  28.777  1.00 84.02           C
ATOM   2069  C   ASP A 268     -11.702 -31.688  29.680  1.00 72.03           C
ATOM   2070  O   ASP A 268     -12.403 -31.906  30.671  1.00 72.18           O
ATOM   2071  CB  ASP A 268     -12.163 -32.971  27.526  1.00 89.92           C
ATOM   2072  CG  ASP A 268     -13.672 -32.898  27.815  1.00 95.89           C
ATOM   2073  OD1 ASP A 268     -14.092 -32.951  28.986  1.00 99.79           O
```

```
ATOM   2074  OD2 ASP A 268     -14.449 -32.752  26.834  1.00 85.45           O
ATOM   2075  N   PRO A 269     -11.263 -30.465  29.376  1.00 70.29           N
ATOM   2076  CA  PRO A 269     -11.386 -29.348  30.322  1.00 72.05           C
ATOM   2077  C   PRO A 269     -12.774 -29.146  30.894  1.00 64.95           C
ATOM   2078  O   PRO A 269     -12.922 -28.664  32.018  1.00 61.77           O
ATOM   2079  CB  PRO A 269     -10.976 -28.129  29.483  1.00 64.99           C
ATOM   2080  CG  PRO A 269     -10.638 -28.649  28.117  1.00 68.20           C
ATOM   2081  CD  PRO A 269     -10.399 -30.112  28.246  1.00 84.59           C
ATOM   2082  N   GLN A 270     -13.805 -29.504  30.150  1.00 70.14           N
ATOM   2083  CA  GLN A 270     -15.118 -29.105  30.614  1.00 65.79           C
ATOM   2084  C   GLN A 270     -15.771 -30.112  31.550  1.00 70.99           C
ATOM   2085  O   GLN A 270     -16.504 -29.680  32.441  1.00 75.03           O
ATOM   2086  CB  GLN A 270     -16.038 -28.778  29.429  1.00 77.18           C
ATOM   2087  CG  GLN A 270     -17.367 -28.301  29.845  1.00 69.45           C
ATOM   2088  CD  GLN A 270     -17.833 -27.055  29.165  1.00 88.98           C
ATOM   2089  OE1 GLN A 270     -18.158 -27.045  28.053  1.00102.57           O
ATOM   2090  NE2 GLN A 270     -17.864 -25.988  29.844  1.00 72.39           N
ATOM   2091  N   GLU A 271     -15.510 -31.429  31.452  1.00 67.98           N
ATOM   2092  CA  GLU A 271     -16.087 -32.281  32.494  1.00 65.98           C
ATOM   2093  C   GLU A 271     -15.417 -32.035  33.844  1.00 75.21           C
ATOM   2094  O   GLU A 271     -15.987 -32.374  34.892  1.00 68.33           O
ATOM   2095  CB  GLU A 271     -16.104 -33.778  32.104  1.00 68.85           C
ATOM   2096  CG  GLU A 271     -14.911 -34.428  31.423  1.00 92.19           C
ATOM   2097  CD  GLU A 271     -15.325 -35.614  30.501  1.00 95.93           C
ATOM   2098  OE1 GLU A 271     -16.511 -35.915  30.416  1.00 78.07           O
ATOM   2099  OE2 GLU A 271     -14.473 -36.232  29.859  1.00 88.54           O
ATOM   2100  N   ILE A 272     -14.266 -31.370  33.849  1.00 70.52           N
ATOM   2101  CA  ILE A 272     -13.751 -30.812  35.089  1.00 66.32           C
ATOM   2102  C   ILE A 272     -14.657 -29.695  35.595  1.00 68.26           C
ATOM   2103  O   ILE A 272     -15.064 -29.684  36.761  1.00 71.83           O
ATOM   2104  CB  ILE A 272     -12.309 -30.319  34.894  1.00 68.84           C
ATOM   2105  CG1 ILE A 272     -11.360 -31.487  35.094  1.00 69.35           C
ATOM   2106  CG2 ILE A 272     -11.978 -29.132  35.831  1.00 59.21           C
ATOM   2107  CD1 ILE A 272      -9.944 -31.112  34.933  1.00 70.46           C
ATOM   2108  N   LEU A 273     -14.994 -28.741  34.730  1.00 65.66           N
ATOM   2109  CA  LEU A 273     -15.618 -27.527  35.233  1.00 61.33           C
ATOM   2110  C   LEU A 273     -17.028 -27.751  35.752  1.00 65.38           C
ATOM   2111  O   LEU A 273     -17.452 -27.022  36.651  1.00 76.52           O
ATOM   2112  CB  LEU A 273     -15.630 -26.416  34.177  1.00 70.78           C
ATOM   2113  CG  LEU A 273     -14.303 -25.816  33.681  1.00 62.28           C
ATOM   2114  CD1 LEU A 273     -14.504 -25.001  32.418  1.00 55.50           C
ATOM   2115  CD2 LEU A 273     -13.624 -24.971  34.785  1.00 67.10           C
ATOM   2116  N   LEU A 274     -17.783 -28.718  35.229  1.00 68.43           N
ATOM   2117  CA  LEU A 274     -19.162 -28.825  35.705  1.00 75.78           C
```

ATOM	2118	C	LEU	A	274	-19.363	-29.783	36.866	1.00	71.53	C
ATOM	2119	O	LEU	A	274	-20.472	-29.826	37.407	1.00	79.51	O
ATOM	2120	CB	LEU	A	274	-20.139	-29.239	34.607	1.00	76.00	C
ATOM	2121	CG	LEU	A	274	-20.174	-28.447	33.326	1.00	69.51	C
ATOM	2122	CD1	LEU	A	274	-19.456	-29.256	32.289	1.00	68.97	C
ATOM	2123	CD2	LEU	A	274	-21.622	-28.179	32.978	1.00	70.94	C
ATOM	2124	N	ASN	A	275	-18.364	-30.575	37.239	1.00	73.13	N
ATOM	2125	CA	ASN	A	275	-18.425	-31.262	38.520	1.00	71.31	C
ATOM	2126	C	ASN	A	275	-17.777	-30.459	39.635	1.00	78.05	C
ATOM	2127	O	ASN	A	275	-17.823	-30.890	40.796	1.00	75.51	O
ATOM	2128	CB	ASN	A	275	-17.761	-32.634	38.453	1.00	69.05	C
ATOM	2129	CG	ASN	A	275	-18.274	-33.454	37.337	1.00	70.69	C
ATOM	2130	OD1	ASN	A	275	-19.365	-34.001	37.436	1.00	78.38	O
ATOM	2131	ND2	ASN	A	275	-17.480	-33.601	36.280	1.00	72.54	N
ATOM	2132	N	GLU	A	276	-17.159	-29.320	39.293	1.00	72.63	N
ATOM	2133	CA	GLU	A	276	-16.605	-28.401	40.282	1.00	63.84	C
ATOM	2134	C	GLU	A	276	-17.649	-27.968	41.288	1.00	59.97	C
ATOM	2135	O	GLU	A	276	-17.320	-27.692	42.440	1.00	59.97	O
ATOM	2136	CB	GLU	A	276	-16.038	-27.169	39.578	1.00	69.61	C
ATOM	2137	CG	GLU	A	276	-14.654	-27.332	38.959	1.00	74.17	C
ATOM	2138	CD	GLU	A	276	-14.042	-25.998	38.532	1.00	71.62	C
ATOM	2139	OE1	GLU	A	276	-14.814	-25.034	38.324	1.00	65.88	O
ATOM	2140	OE2	GLU	A	276	-12.795	-25.911	38.386	1.00	75.45	O
ATOM	2141	N	ALA	A	277	-18.908	-27.901	40.869	1.00	62.41	N
ATOM	2142	CA	ALA	A	277	-19.973	-27.392	41.716	1.00	63.40	C
ATOM	2143	C	ALA	A	277	-20.396	-28.355	42.806	1.00	70.25	C
ATOM	2144	O	ALA	A	277	-21.025	-27.914	43.774	1.00	77.96	O
ATOM	2145	CB	ALA	A	277	-21.194	-27.053	40.869	1.00	73.08	C
ATOM	2146	N	PHE	A	278	-20.088	-29.650	42.669	1.00	76.38	N
ATOM	2147	CA	PHE	A	278	-20.549	-30.679	43.595	1.00	69.47	C
ATOM	2148	C	PHE	A	278	-19.496	-31.135	44.600	1.00	76.14	C
ATOM	2149	O	PHE	A	278	-19.832	-31.910	45.497	1.00	94.33	O
ATOM	2150	CB	PHE	A	278	-21.039	-31.930	42.848	1.00	81.42	C
ATOM	2151	CG	PHE	A	278	-21.909	-31.666	41.654	1.00	72.14	C
ATOM	2152	CD1	PHE	A	278	-23.221	-31.262	41.807	1.00	68.73	C
ATOM	2153	CD2	PHE	A	278	-21.431	-31.917	40.378	1.00	75.49	C
ATOM	2154	CE1	PHE	A	278	-24.022	-31.060	40.717	1.00	69.66	C
ATOM	2155	CE2	PHE	A	278	-22.227	-31.719	39.280	1.00	77.97	C
ATOM	2156	CZ	PHE	A	278	-23.529	-31.287	39.450	1.00	78.13	C
ATOM	2157	N	VAL	A	279	-18.240	-30.698	44.482	1.00	69.01	N
ATOM	2158	CA	VAL	A	279	-17.150	-31.239	45.299	1.00	70.18	C
ATOM	2159	C	VAL	A	279	-17.423	-31.000	46.779	1.00	83.93	C
ATOM	2160	O	VAL	A	279	-16.662	-31.439	47.647	1.00	94.49	O
ATOM	2161	CB	VAL	A	279	-15.783	-30.634	44.916	1.00	70.06	C

```
ATOM   2162  CG1 VAL A 279     -15.389 -31.038  43.511  1.00 75.28           C
ATOM   2163  CG2 VAL A 279     -15.782 -29.117  45.078  1.00 69.42           C
ATOM   2164  N   VAL A 280     -18.505 -30.295  47.072  1.00 87.35           N
ATOM   2165  CA  VAL A 280     -18.868 -29.906  48.427  1.00 87.59           C
ATOM   2166  C   VAL A 280     -20.312 -30.351  48.632  1.00103.63           C
ATOM   2167  O   VAL A 280     -21.132 -30.215  47.708  1.00108.04           O
ATOM   2168  CB  VAL A 280     -18.683 -28.393  48.614  1.00 80.12           C
ATOM   2169  CG1 VAL A 280     -19.462 -27.642  47.552  1.00 81.96           C
ATOM   2170  CG2 VAL A 280     -19.112 -27.960  49.980  1.00 84.16           C
ATOM   2171  N   PRO A 281     -20.669 -30.920  49.792  1.00104.12           N
ATOM   2172  CA  PRO A 281     -22.070 -31.322  50.000  1.00100.75           C
ATOM   2173  C   PRO A 281     -23.014 -30.124  49.969  1.00101.72           C
ATOM   2174  O   PRO A 281     -23.751 -29.919  48.996  1.00105.98           O
ATOM   2175  CB  PRO A 281     -22.040 -32.003  51.374  1.00 95.60           C
ATOM   2176  CG  PRO A 281     -20.806 -31.435  52.049  1.00 96.93           C
ATOM   2177  CD  PRO A 281     -19.815 -31.220  50.956  1.00 90.51           C
ATOM   2178  N   TYR A 282     -22.987 -29.313  51.018  1.00 91.98           N
ATOM   2179  CA  TYR A 282     -23.792 -28.108  51.076  1.00 92.35           C
ATOM   2180  C   TYR A 282     -22.962 -26.933  50.589  1.00 93.91           C
ATOM   2181  O   TYR A 282     -21.773 -26.826  50.896  1.00 89.21           O
ATOM   2182  CB  TYR A 282     -24.294 -27.852  52.498  1.00100.07           C
ATOM   2183  N   GLY A 283     -23.591 -26.058  49.811  1.00 99.13           N
ATOM   2184  CA  GLY A 283     -22.960 -24.846  49.349  1.00 94.18           C
ATOM   2185  C   GLY A 283     -23.751 -23.624  49.781  1.00 91.02           C
ATOM   2186  O   GLY A 283     -24.780 -23.717  50.455  1.00 93.07           O
ATOM   2187  N   THR A 284     -23.224 -22.473  49.401  1.00 87.06           N
ATOM   2188  CA  THR A 284     -23.879 -21.178  49.521  1.00 79.37           C
ATOM   2189  C   THR A 284     -23.695 -20.469  48.197  1.00 76.61           C
ATOM   2190  O   THR A 284     -22.935 -20.926  47.338  1.00 83.06           O
ATOM   2191  CB  THR A 284     -23.260 -20.342  50.649  1.00 74.94           C
ATOM   2192  OG1 THR A 284     -22.047 -19.739  50.165  1.00 71.76           O
ATOM   2193  CG2 THR A 284     -22.971 -21.209  51.873  1.00 66.99           C
ATOM   2194  N   PRO A 285     -24.368 -19.338  47.989  1.00 74.53           N
ATOM   2195  CA  PRO A 285     -24.049 -18.545  46.791  1.00 68.66           C
ATOM   2196  C   PRO A 285     -22.623 -18.052  46.787  1.00 67.75           C
ATOM   2197  O   PRO A 285     -22.109 -17.693  45.721  1.00 75.69           O
ATOM   2198  CB  PRO A 285     -25.057 -17.397  46.844  1.00 66.23           C
ATOM   2199  CG  PRO A 285     -26.231 -17.991  47.583  1.00 71.75           C
ATOM   2200  CD  PRO A 285     -25.616 -18.886  48.633  1.00 63.60           C
ATOM   2201  N   LEU A 286     -21.954 -18.067  47.938  1.00 66.23           N
ATOM   2202  CA  LEU A 286     -20.582 -17.597  48.058  1.00 76.50           C
ATOM   2203  C   LEU A 286     -19.555 -18.711  48.024  1.00 81.37           C
ATOM   2204  O   LEU A 286     -18.383 -18.455  48.326  1.00 82.82           O
ATOM   2205  CB  LEU A 286     -20.397 -16.793  49.341  1.00 67.24           C
```

```
ATOM   2206  CG  LEU A 286     -21.217 -15.518  49.440  1.00 57.99           C
ATOM   2207  CD1 LEU A 286     -22.550 -15.710  50.139  1.00 55.54           C
ATOM   2208  CD2 LEU A 286     -20.349 -14.537  50.136  1.00 60.98           C
ATOM   2209  N   SER A 287     -19.954 -19.933  47.672  1.00 85.47           N
ATOM   2210  CA  SER A 287     -19.050 -21.068  47.830  1.00 84.29           C
ATOM   2211  C   SER A 287     -17.878 -20.943  46.871  1.00 69.97           C
ATOM   2212  O   SER A 287     -18.060 -20.969  45.643  1.00 67.26           O
ATOM   2213  CB  SER A 287     -19.772 -22.409  47.635  1.00 92.09           C
ATOM   2214  OG  SER A 287     -20.788 -22.627  48.606  1.00 91.45           O
ATOM   2215  N   VAL A 288     -16.687 -20.785  47.453  1.00 55.24           N
ATOM   2216  CA  VAL A 288     -15.416 -20.836  46.741  1.00 63.39           C
ATOM   2217  C   VAL A 288     -14.949 -22.290  46.823  1.00 68.91           C
ATOM   2218  O   VAL A 288     -14.337 -22.699  47.819  1.00 70.79           O
ATOM   2219  CB  VAL A 288     -14.385 -19.862  47.331  1.00 55.77           C
ATOM   2220  CG1 VAL A 288     -13.108 -19.857  46.521  1.00 57.76           C
ATOM   2221  CG2 VAL A 288     -14.938 -18.450  47.396  1.00 51.93           C
ATOM   2222  N   ASN A 289     -15.247 -23.079  45.774  1.00 67.34           N
ATOM   2223  CA  ASN A 289     -14.993 -24.518  45.818  1.00 52.73           C
ATOM   2224  C   ASN A 289     -13.501 -24.828  45.772  1.00 55.89           C
ATOM   2225  O   ASN A 289     -13.044 -25.797  46.394  1.00 56.88           O
ATOM   2226  CB  ASN A 289     -15.722 -25.207  44.669  1.00 55.01           C
ATOM   2227  CG  ASN A 289     -17.222 -24.970  44.698  1.00 66.77           C
ATOM   2228  OD1 ASN A 289     -17.764 -24.261  43.842  1.00 74.63           O
ATOM   2229  ND2 ASN A 289     -17.907 -25.595  45.652  1.00 61.86           N
ATOM   2230  N   PHE A 290     -12.721 -24.018  45.054  1.00 43.69           N
ATOM   2231  CA  PHE A 290     -11.269 -24.211  44.963  1.00 55.01           C
ATOM   2232  C   PHE A 290     -10.632 -22.825  45.024  1.00 47.78           C
ATOM   2233  O   PHE A 290     -10.585 -22.118  44.019  1.00 48.93           O
ATOM   2234  CB  PHE A 290     -10.868 -24.959  43.681  1.00 55.82           C
ATOM   2235  CG  PHE A 290     -11.422 -26.374  43.567  1.00 48.77           C
ATOM   2236  CD1 PHE A 290     -10.724 -27.462  44.066  1.00 55.39           C
ATOM   2237  CD2 PHE A 290     -12.661 -26.603  42.983  1.00 53.76           C
ATOM   2238  CE1 PHE A 290     -11.234 -28.747  43.954  1.00 53.87           C
ATOM   2239  CE2 PHE A 290     -13.179 -27.882  42.888  1.00 50.99           C
ATOM   2240  CZ  PHE A 290     -12.465 -28.951  43.377  1.00 50.29           C
ATOM   2241  N   GLY A 291     -10.142 -22.433  46.189  1.00 48.93           N
ATOM   2242  CA  GLY A 291      -9.421 -21.192  46.307  1.00 48.38           C
ATOM   2243  C   GLY A 291      -7.988 -21.339  46.787  1.00 58.24           C
ATOM   2244  O   GLY A 291      -7.360 -22.405  46.674  1.00 69.75           O
ATOM   2245  N   PRO A 292      -7.437 -20.245  47.319  1.00 59.14           N
ATOM   2246  CA  PRO A 292      -6.121 -20.315  47.957  1.00 59.09           C
ATOM   2247  C   PRO A 292      -6.107 -21.320  49.099  1.00 55.46           C
ATOM   2248  O   PRO A 292      -7.112 -21.538  49.783  1.00 48.79           O
ATOM   2249  CB  PRO A 292      -5.910 -18.888  48.472  1.00 47.95           C
```

ATOM	2250	CG	PRO	A	292	-6.746	-18.060	47.589	1.00	51.47	C
ATOM	2251	CD	PRO	A	292	-7.966	-18.871	47.320	1.00	50.52	C
ATOM	2252	N	THR	A	293	-4.941	-21.926	49.302	1.00	53.18	N
ATOM	2253	CA	THR	A	293	-4.716	-22.837	50.415	1.00	72.49	C
ATOM	2254	C	THR	A	293	-3.370	-22.504	51.049	1.00	72.11	C
ATOM	2255	O	THR	A	293	-2.582	-21.725	50.499	1.00	62.58	O
ATOM	2256	CB	THR	A	293	-4.767	-24.299	49.947	1.00	71.30	C
ATOM	2257	OG1	THR	A	293	-4.110	-24.409	48.676	1.00	70.26	O
ATOM	2258	CG2	THR	A	293	-6.207	-24.758	49.790	1.00	70.49	C
ATOM	2259	N	VAL	A	294	-3.103	-23.083	52.219	1.00	72.68	N
ATOM	2260	CA	VAL	A	294	-1.757	-22.997	52.779	1.00	76.75	C
ATOM	2261	C	VAL	A	294	-0.931	-24.089	52.107	1.00	71.24	C
ATOM	2262	O	VAL	A	294	-1.233	-25.277	52.218	1.00	75.30	O
ATOM	2263	CB	VAL	A	294	-1.745	-23.102	54.315	1.00	76.42	C
ATOM	2264	CG1	VAL	A	294	-2.756	-22.146	54.912	1.00	80.11	C
ATOM	2265	CG2	VAL	A	294	-2.034	-24.496	54.829	1.00	81.27	C
ATOM	2266	N	ASP	A	295	0.058	-23.687	51.321	1.00	68.80	N
ATOM	2267	CA	ASP	A	295	0.799	-24.665	50.549	1.00	68.00	C
ATOM	2268	C	ASP	A	295	2.242	-24.801	50.998	1.00	67.00	C
ATOM	2269	O	ASP	A	295	2.932	-25.720	50.548	1.00	66.28	O
ATOM	2270	CB	ASP	A	295	0.740	-24.332	49.041	1.00	66.07	C
ATOM	2271	CG	ASP	A	295	1.344	-22.974	48.691	1.00	69.63	C
ATOM	2272	OD1	ASP	A	295	1.331	-22.030	49.512	1.00	78.73	O
ATOM	2273	OD2	ASP	A	295	1.887	-22.867	47.581	1.00	73.42	O
ATOM	2274	N	GLY	A	296	2.709	-23.935	51.887	1.00	74.29	N
ATOM	2275	CA	GLY	A	296	4.118	-23.950	52.201	1.00	84.01	C
ATOM	2276	C	GLY	A	296	5.008	-23.451	51.087	1.00	83.99	C
ATOM	2277	O	GLY	A	296	6.231	-23.579	51.181	1.00	91.61	O
ATOM	2278	N	ASP	A	297	4.433	-22.894	50.020	1.00	81.61	N
ATOM	2279	CA	ASP	A	297	5.243	-22.249	48.999	1.00	72.44	C
ATOM	2280	C	ASP	A	297	4.859	-20.783	48.877	1.00	79.73	C
ATOM	2281	O	ASP	A	297	5.583	-19.906	49.363	1.00	87.48	O
ATOM	2282	CB	ASP	A	297	5.094	-22.957	47.661	1.00	77.41	C
ATOM	2283	CG	ASP	A	297	6.158	-22.532	46.659	1.00	84.55	C
ATOM	2284	OD1	ASP	A	297	6.718	-21.416	46.819	1.00	73.74	O
ATOM	2285	OD2	ASP	A	297	6.445	-23.324	45.723	1.00	79.10	O
ATOM	2286	N	PHE	A	298	3.729	-20.503	48.225	1.00	74.84	N
ATOM	2287	CA	PHE	A	298	3.275	-19.118	48.114	1.00	80.56	C
ATOM	2288	C	PHE	A	298	2.954	-18.556	49.491	1.00	79.46	C
ATOM	2289	O	PHE	A	298	3.427	-17.480	49.873	1.00	76.06	O
ATOM	2290	CB	PHE	A	298	2.061	-19.046	47.183	1.00	64.94	C
ATOM	2291	CG	PHE	A	298	1.578	-17.655	46.907	1.00	61.19	C
ATOM	2292	CD1	PHE	A	298	0.450	-17.161	47.533	1.00	65.42	C
ATOM	2293	CD2	PHE	A	298	2.264	-16.829	46.031	1.00	68.97	C

```
ATOM   2294  CE1 PHE A 298       0.000 -15.861  47.279  1.00 65.34           C
ATOM   2295  CE2 PHE A 298       1.827 -15.528  45.779  1.00 65.88           C
ATOM   2296  CZ  PHE A 298       0.692 -15.045  46.407  1.00 58.02           C
ATOM   2297  N   LEU A 299       2.180 -19.299  50.260  1.00 77.04           N
ATOM   2298  CA  LEU A 299       1.840 -18.955  51.629  1.00 76.68           C
ATOM   2299  C   LEU A 299       2.474 -20.020  52.512  1.00 87.71           C
ATOM   2300  O   LEU A 299       2.124 -21.204  52.404  1.00 85.43           O
ATOM   2301  CB  LEU A 299       0.327 -18.919  51.807  1.00 71.18           C
ATOM   2302  CG  LEU A 299      -0.207 -17.951  52.844  1.00 76.62           C
ATOM   2303  CD1 LEU A 299       0.637 -16.677  52.884  1.00 84.83           C
ATOM   2304  CD2 LEU A 299      -1.671 -17.668  52.578  1.00 75.65           C
ATOM   2305  N   THR A 300       3.398 -19.618  53.387  1.00 85.71           N
ATOM   2306  CA  THR A 300       4.152 -20.643  54.100  1.00 90.84           C
ATOM   2307  C   THR A 300       3.434 -21.181  55.340  1.00 86.01           C
ATOM   2308  O   THR A 300       3.839 -22.232  55.854  1.00 79.05           O
ATOM   2309  CB  THR A 300       5.538 -20.128  54.476  1.00 93.29           C
ATOM   2310  OG1 THR A 300       6.036 -19.285  53.423  1.00 92.31           O
ATOM   2311  CG2 THR A 300       6.491 -21.328  54.628  1.00 86.01           C
ATOM   2312  N   ASP A 301       2.390 -20.502  55.830  1.00 89.50           N
ATOM   2313  CA  ASP A 301       1.423 -21.122  56.737  1.00 95.40           C
ATOM   2314  C   ASP A 301       0.262 -20.144  56.899  1.00 89.07           C
ATOM   2315  O   ASP A 301       0.321 -19.011  56.432  1.00 86.24           O
ATOM   2316  CB  ASP A 301       2.045 -21.470  58.098  1.00 98.25           C
ATOM   2317  CG  ASP A 301       1.072 -22.187  59.048  1.00106.68           C
ATOM   2318  OD1 ASP A 301       1.564 -22.727  60.048  1.00125.35           O
ATOM   2319  OD2 ASP A 301      -0.146 -22.292  58.804  1.00105.98           O
ATOM   2320  N   MET A 302      -0.804 -20.614  57.538  1.00 90.66           N
ATOM   2321  CA  MET A 302      -2.075 -19.972  57.856  1.00 89.60           C
ATOM   2322  C   MET A 302      -1.988 -18.450  57.886  1.00 93.35           C
ATOM   2323  O   MET A 302      -1.371 -17.879  58.788  1.00 93.74           O
ATOM   2324  CB  MET A 302      -2.551 -20.504  59.206  1.00 87.55           C
ATOM   2325  CG  MET A 302      -4.021 -20.475  59.417  1.00 93.78           C
ATOM   2326  SD  MET A 302      -4.843 -21.879  58.652  1.00129.88           S
ATOM   2327  CE  MET A 302      -3.508 -23.067  58.573  1.00 91.21           C
ATOM   2328  N   PRO A 303      -2.630 -17.764  56.941  1.00 87.95           N
ATOM   2329  CA  PRO A 303      -2.404 -16.316  56.815  1.00 82.64           C
ATOM   2330  C   PRO A 303      -2.818 -15.504  58.041  1.00 89.07           C
ATOM   2331  O   PRO A 303      -2.109 -14.554  58.408  1.00 85.89           O
ATOM   2332  CB  PRO A 303      -3.216 -15.956  55.563  1.00 79.52           C
ATOM   2333  CG  PRO A 303      -4.195 -17.066  55.379  1.00 83.81           C
ATOM   2334  CD  PRO A 303      -3.541 -18.294  55.914  1.00 86.55           C
ATOM   2335  N   ASP A 304      -3.928 -15.850  58.706  1.00 95.56           N
ATOM   2336  CA  ASP A 304      -4.301 -15.116  59.918  1.00 90.68           C
ATOM   2337  C   ASP A 304      -3.176 -15.107  60.951  1.00 87.68           C
```

```
ATOM   2338  O   ASP A 304      -3.037 -14.127  61.691  1.00 89.48           O
ATOM   2339  CB  ASP A 304      -5.580 -15.682  60.552  1.00 80.22           C
ATOM   2340  CG  ASP A 304      -5.522 -17.181  60.750  1.00101.01           C
ATOM   2341  OD1 ASP A 304      -5.466 -17.904  59.724  1.00 93.57           O
ATOM   2342  OD2 ASP A 304      -5.537 -17.627  61.926  1.00 99.23           O
ATOM   2343  N   ILE A 305      -2.343 -16.156  61.004  1.00 92.72           N
ATOM   2344  CA  ILE A 305      -1.277 -16.175  62.008  1.00 93.96           C
ATOM   2345  C   ILE A 305      -0.164 -15.200  61.627  1.00100.29           C
ATOM   2346  O   ILE A 305       0.293 -14.419  62.472  1.00109.12           O
ATOM   2347  CB  ILE A 305      -0.737 -17.606  62.271  1.00 94.93           C
ATOM   2348  CG1 ILE A 305       0.385 -18.025  61.312  1.00 98.94           C
ATOM   2349  CG2 ILE A 305      -1.882 -18.664  62.326  1.00 90.51           C
ATOM   2350  CD1 ILE A 305       1.247 -19.176  61.806  1.00 91.80           C
ATOM   2351  N   LEU A 306       0.268 -15.190  60.349  1.00 90.01           N
ATOM   2352  CA  LEU A 306       1.442 -14.374  60.032  1.00 93.87           C
ATOM   2353  C   LEU A 306       1.144 -12.880  60.060  1.00 92.53           C
ATOM   2354  O   LEU A 306       2.030 -12.106  60.431  1.00 95.54           O
ATOM   2355  CB  LEU A 306       2.078 -14.720  58.680  1.00 94.62           C
ATOM   2356  CG  LEU A 306       2.456 -16.046  58.111  1.00 99.05           C
ATOM   2357  CD1 LEU A 306       1.203 -16.544  57.643  1.00 93.80           C
ATOM   2358  CD2 LEU A 306       3.405 -15.807  56.968  1.00 94.80           C
ATOM   2359  N   LEU A 307      -0.072 -12.449  59.731  1.00 84.95           N
ATOM   2360  CA  LEU A 307      -0.367 -11.046  59.881  1.00 93.99           C
ATOM   2361  C   LEU A 307      -0.611 -10.765  61.348  1.00100.47           C
ATOM   2362  O   LEU A 307      -0.037  -9.798  61.822  1.00 98.66           O
ATOM   2363  CB  LEU A 307      -1.427 -10.702  58.807  1.00102.25           C
ATOM   2364  CG  LEU A 307      -2.159  -9.420  58.559  1.00 99.35           C
ATOM   2365  CD1 LEU A 307      -1.943  -9.296  57.102  1.00 98.51           C
ATOM   2366  CD2 LEU A 307      -3.642  -9.636  58.623  1.00 90.49           C
ATOM   2367  N   GLU A 308      -1.223 -11.663  62.145  1.00101.99           N
ATOM   2368  CA  GLU A 308      -1.329 -11.385  63.586  1.00101.84           C
ATOM   2369  C   GLU A 308       0.036 -11.259  64.254  1.00101.12           C
ATOM   2370  O   GLU A 308       0.242 -10.364  65.076  1.00108.17           O
ATOM   2371  CB  GLU A 308      -2.176 -12.448  64.270  1.00 92.27           C
ATOM   2372  CG  GLU A 308      -3.472 -11.885  64.906  1.00101.22           C
ATOM   2373  CD  GLU A 308      -3.249 -11.082  66.206  1.00117.94           C
ATOM   2374  OE1 GLU A 308      -2.089 -10.793  66.602  1.00107.29           O
ATOM   2375  OE2 GLU A 308      -4.259 -10.747  66.877  1.00126.73           O
ATOM   2376  N   LEU A 309       0.994 -12.071  63.839  1.00 90.73           N
ATOM   2377  CA  LEU A 309       2.398 -11.970  64.180  1.00 87.82           C
ATOM   2378  C   LEU A 309       3.116 -10.914  63.346  1.00 97.70           C
ATOM   2379  O   LEU A 309       4.284 -10.620  63.619  1.00105.00           O
ATOM   2380  CB  LEU A 309       2.968 -13.372  63.980  1.00 85.44           C
ATOM   2381  CG  LEU A 309       4.324 -13.980  64.259  1.00 95.26           C
```

```
ATOM   2382  CD1 LEU A 309       4.115 -15.481  63.965  1.00 96.36           C
ATOM   2383  CD2 LEU A 309       5.275 -13.372  63.309  1.00 90.87           C
ATOM   2384  N   GLY A 310       2.445 -10.339  62.351  1.00103.25           N
ATOM   2385  CA  GLY A 310       3.024  -9.275  61.537  1.00 98.87           C
ATOM   2386  C   GLY A 310       4.239  -9.640  60.705  1.00 99.20           C
ATOM   2387  O   GLY A 310       5.237  -8.909  60.732  1.00 99.07           O
ATOM   2388  N   GLN A 311       4.178 -10.740  59.943  1.00 98.68           N
ATOM   2389  CA  GLN A 311       5.224 -11.066  58.966  1.00 97.24           C
ATOM   2390  C   GLN A 311       4.714 -10.747  57.564  1.00 96.39           C
ATOM   2391  O   GLN A 311       4.366 -11.626  56.775  1.00 97.97           O
ATOM   2392  CB  GLN A 311       5.650 -12.518  59.080  1.00 95.71           C
ATOM   2393  CG  GLN A 311       6.418 -12.826  60.328  1.00100.17           C
ATOM   2394  CD  GLN A 311       7.665 -13.648  60.077  1.00110.07           C
ATOM   2395  OE1 GLN A 311       7.602 -14.879  59.975  1.00108.90           O
ATOM   2396  NE2 GLN A 311       8.814 -12.978  60.014  1.00107.44           N
ATOM   2397  N   PHE A 312       4.689  -9.459  57.247  1.00 86.54           N
ATOM   2398  CA  PHE A 312       4.207  -9.059  55.942  1.00 85.33           C
ATOM   2399  C   PHE A 312       4.992  -7.855  55.454  1.00 87.02           C
ATOM   2400  O   PHE A 312       5.706  -7.192  56.212  1.00 81.38           O
ATOM   2401  CB  PHE A 312       2.703  -8.759  55.966  1.00 89.06           C
ATOM   2402  CG  PHE A 312       2.269  -7.834  57.079  1.00 90.52           C
ATOM   2403  CD1 PHE A 312       2.465  -6.474  56.979  1.00 81.37           C
ATOM   2404  CD2 PHE A 312       1.624  -8.329  58.203  1.00 90.18           C
ATOM   2405  CE1 PHE A 312       2.053  -5.625  57.981  1.00 85.42           C
ATOM   2406  CE2 PHE A 312       1.204  -7.480  59.206  1.00 93.76           C
ATOM   2407  CZ  PHE A 312       1.420  -6.126  59.094  1.00 89.82           C
ATOM   2408  N   LYS A 313       4.870  -7.610  54.156  1.00 87.18           N
ATOM   2409  CA  LYS A 313       5.409  -6.403  53.566  1.00 85.39           C
ATOM   2410  C   LYS A 313       4.901  -5.196  54.345  1.00 80.70           C
ATOM   2411  O   LYS A 313       3.721  -5.124  54.695  1.00 73.38           O
ATOM   2412  CB  LYS A 313       4.993  -6.327  52.089  1.00 76.19           C
ATOM   2413  CG  LYS A 313       5.778  -5.312  51.277  1.00 78.74           C
ATOM   2414  CD  LYS A 313       5.258  -5.182  49.866  1.00 78.43           C
ATOM   2415  CE  LYS A 313       5.925  -4.031  49.156  1.00 69.54           C
ATOM   2416  NZ  LYS A 313       7.382  -4.285  49.074  1.00 77.17           N
ATOM   2417  N   LYS A 314       5.807  -4.269  54.650  1.00 82.68           N
ATOM   2418  CA  LYS A 314       5.471  -3.044  55.361  1.00 81.41           C
ATOM   2419  C   LYS A 314       5.323  -1.914  54.356  1.00 77.99           C
ATOM   2420  O   LYS A 314       6.297  -1.539  53.698  1.00 83.68           O
ATOM   2421  CB  LYS A 314       6.540  -2.694  56.395  1.00 87.67           C
ATOM   2422  CG  LYS A 314       6.767  -3.775  57.433  1.00 77.88           C
ATOM   2423  CD  LYS A 314       5.549  -3.951  58.299  1.00 73.00           C
ATOM   2424  CE  LYS A 314       5.451  -5.383  58.735  1.00 79.54           C
ATOM   2425  NZ  LYS A 314       6.748  -5.804  59.323  1.00 80.12           N
```

ATOM	2426	N	THR	A	315	4.119	-1.356	54.265	1.00	80.01	N
ATOM	2427	CA	THR	A	315	3.783	-0.366	53.248	1.00	81.01	C
ATOM	2428	C	THR	A	315	2.365	0.154	53.496	1.00	81.43	C
ATOM	2429	O	THR	A	315	1.610	-0.401	54.300	1.00	78.53	O
ATOM	2430	CB	THR	A	315	3.950	-0.993	51.858	1.00	83.63	C
ATOM	2431	OG1	THR	A	315	5.348	-1.107	51.551	1.00	87.66	O
ATOM	2432	CG2	THR	A	315	3.242	-0.223	50.800	1.00	78.62	C
ATOM	2433	N	GLN	A	316	2.044	1.274	52.839	1.00	79.13	N
ATOM	2434	CA	GLN	A	316	0.712	1.868	52.836	1.00	76.40	C
ATOM	2435	C	GLN	A	316	-0.274	1.005	52.052	1.00	78.63	C
ATOM	2436	O	GLN	A	316	0.085	0.425	51.015	1.00	83.19	O
ATOM	2437	CB	GLN	A	316	0.760	3.268	52.207	1.00	79.09	C
ATOM	2438	CG	GLN	A	316	1.969	4.172	52.544	1.00	87.76	C
ATOM	2439	CD	GLN	A	316	3.318	3.756	51.917	1.00	93.23	C
ATOM	2440	OE1	GLN	A	316	3.649	2.570	51.795	1.00	89.96	O
ATOM	2441	NE2	GLN	A	316	4.072	4.754	51.464	1.00	97.24	N
ATOM	2442	N	ILE	A	317	-1.519	0.925	52.554	1.00	68.25	N
ATOM	2443	CA	ILE	A	317	-2.590	0.189	51.891	1.00	69.39	C
ATOM	2444	C	ILE	A	317	-3.841	1.050	51.873	1.00	71.60	C
ATOM	2445	O	ILE	A	317	-4.023	1.943	52.698	1.00	72.80	O
ATOM	2446	CB	ILE	A	317	-2.927	-1.178	52.545	1.00	75.83	C
ATOM	2447	CG1	ILE	A	317	-3.743	-0.977	53.831	1.00	78.38	C
ATOM	2448	CG2	ILE	A	317	-1.681	-1.976	52.810	1.00	67.93	C
ATOM	2449	CD1	ILE	A	317	-3.914	-2.230	54.692	1.00	76.40	C
ATOM	2450	N	LEU	A	318	-4.722	0.743	50.923	1.00	79.15	N
ATOM	2451	CA	LEU	A	318	-5.973	1.463	50.701	1.00	74.32	C
ATOM	2452	C	LEU	A	318	-7.072	0.416	50.584	1.00	69.49	C
ATOM	2453	O	LEU	A	318	-7.049	-0.394	49.652	1.00	69.01	O
ATOM	2454	CB	LEU	A	318	-5.870	2.332	49.435	1.00	69.21	C
ATOM	2455	CG	LEU	A	318	-7.137	3.054	48.995	1.00	71.01	C
ATOM	2456	CD1	LEU	A	318	-7.539	3.839	50.184	1.00	83.94	C
ATOM	2457	CD2	LEU	A	318	-6.868	4.003	47.829	1.00	77.96	C
ATOM	2458	N	VAL	A	319	-7.999	0.380	51.546	1.00	63.10	N
ATOM	2459	CA	VAL	A	319	-8.995	-0.694	51.597	1.00	64.32	C
ATOM	2460	C	VAL	A	319	-10.352	-0.136	51.988	1.00	55.75	C
ATOM	2461	O	VAL	A	319	-10.462	0.684	52.897	1.00	61.67	O
ATOM	2462	CB	VAL	A	319	-8.592	-1.836	52.559	1.00	55.25	C
ATOM	2463	CG1	VAL	A	319	-8.317	-1.322	53.926	1.00	67.14	C
ATOM	2464	CG2	VAL	A	319	-9.693	-2.886	52.643	1.00	68.84	C
ATOM	2465	N	GLY	A	320	-11.389	-0.585	51.299	1.00	60.23	N
ATOM	2466	CA	GLY	A	320	-12.732	-0.156	51.610	1.00	58.79	C
ATOM	2467	C	GLY	A	320	-13.744	-1.189	51.171	1.00	57.37	C
ATOM	2468	O	GLY	A	320	-13.393	-2.298	50.764	1.00	56.38	O
ATOM	2469	N	VAL	A	321	-15.015	-0.817	51.290	1.00	51.37	N

ATOM	2470	CA	VAL	A	321	-16.139	-1.706	51.048	1.00	46.30	C
ATOM	2471	C	VAL	A	321	-17.299	-0.865	50.548	1.00	57.99	C
ATOM	2472	O	VAL	A	321	-17.268	0.366	50.588	1.00	67.12	O
ATOM	2473	CB	VAL	A	321	-16.581	-2.488	52.304	1.00	50.15	C
ATOM	2474	CG1	VAL	A	321	-15.465	-3.370	52.836	1.00	66.83	C
ATOM	2475	CG2	VAL	A	321	-17.083	-1.566	53.382	1.00	54.18	C
ATOM	2476	N	ASN	A	322	-18.333	-1.535	50.065	1.00	57.75	N
ATOM	2477	CA	ASN	A	322	-19.557	-0.865	49.653	1.00	65.84	C
ATOM	2478	C	ASN	A	322	-20.648	-1.212	50.659	1.00	60.95	C
ATOM	2479	O	ASN	A	322	-20.448	-2.024	51.551	1.00	60.80	O
ATOM	2480	CB	ASN	A	322	-19.954	-1.260	48.222	1.00	68.19	C
ATOM	2481	CG	ASN	A	322	-18.780	-1.184	47.238	1.00	69.68	C
ATOM	2482	OD1	ASN	A	322	-17.638	-0.829	47.618	1.00	58.87	O
ATOM	2483	ND2	ASN	A	322	-19.054	-1.528	45.962	1.00	70.67	N
ATOM	2484	N	LYS	A	323	-21.819	-0.598	50.513	1.00	58.94	N
ATOM	2485	CA	LYS	A	323	-22.791	-0.539	51.613	1.00	65.95	C
ATOM	2486	C	LYS	A	323	-23.697	-1.741	51.657	1.00	71.20	C
ATOM	2487	O	LYS	A	323	-24.048	-2.238	52.737	1.00	87.10	O
ATOM	2488	CB	LYS	A	323	-23.683	0.685	51.468	1.00	79.78	C
ATOM	2489	CG	LYS	A	323	-22.959	1.838	50.950	1.00	85.39	C
ATOM	2490	CD	LYS	A	323	-21.769	2.008	51.854	1.00	90.44	C
ATOM	2491	CE	LYS	A	323	-21.021	3.165	51.371	1.00	92.45	C
ATOM	2492	NZ	LYS	A	323	-20.189	4.182	51.911	1.00	79.93	N
ATOM	2493	N	ASP	A	324	-24.230	-2.084	50.504	1.00	70.35	N
ATOM	2494	CA	ASP	A	324	-25.186	-3.157	50.393	1.00	71.39	C
ATOM	2495	C	ASP	A	324	-24.428	-4.267	49.682	1.00	62.08	C
ATOM	2496	O	ASP	A	324	-24.839	-4.735	48.614	1.00	68.69	O
ATOM	2497	CB	ASP	A	324	-26.414	-2.655	49.641	1.00	72.29	C
ATOM	2498	CG	ASP	A	324	-27.021	-1.379	50.279	1.00	84.51	C
ATOM	2499	OD1	ASP	A	324	-26.264	-0.486	50.731	1.00	83.41	O
ATOM	2500	OD2	ASP	A	324	-28.274	-1.248	50.323	1.00	95.92	O
ATOM	2501	N	GLU	A	325	-23.264	-4.632	50.244	1.00	57.56	N
ATOM	2502	CA	GLU	A	325	-22.439	-5.657	49.618	1.00	63.72	C
ATOM	2503	C	GLU	A	325	-23.075	-7.030	49.731	1.00	68.07	C
ATOM	2504	O	GLU	A	325	-22.870	-7.872	48.852	1.00	71.95	O
ATOM	2505	CB	GLU	A	325	-21.043	-5.660	50.219	1.00	57.33	C
ATOM	2506	CG	GLU	A	325	-20.198	-4.580	49.591	1.00	62.15	C
ATOM	2507	CD	GLU	A	325	-18.727	-4.797	49.771	1.00	62.62	C
ATOM	2508	OE1	GLU	A	325	-17.965	-4.405	48.870	1.00	54.94	O
ATOM	2509	OE2	GLU	A	325	-18.340	-5.387	50.795	1.00	70.53	O
ATOM	2510	N	GLY	A	326	-23.874	-7.262	50.775	1.00	61.22	N
ATOM	2511	CA	GLY	A	326	-24.585	-8.519	50.896	1.00	59.38	C
ATOM	2512	C	GLY	A	326	-25.709	-8.732	49.894	1.00	61.69	C
ATOM	2513	O	GLY	A	326	-26.059	-9.886	49.624	1.00	66.28	O

ATOM	2514	N	THR	A	327	-26.284	-7.660	49.335	1.00	61.51	N
ATOM	2515	CA	THR	A	327	-27.545	-7.811	48.605	1.00	70.77	C
ATOM	2516	C	THR	A	327	-27.404	-8.658	47.344	1.00	68.35	C
ATOM	2517	O	THR	A	327	-28.333	-9.402	47.004	1.00	67.38	O
ATOM	2518	CB	THR	A	327	-28.137	-6.454	48.218	1.00	70.64	C
ATOM	2519	OG1	THR	A	327	-27.228	-5.762	47.344	1.00	71.09	O
ATOM	2520	CG2	THR	A	327	-28.446	-5.629	49.455	1.00	64.11	C
ATOM	2521	N	ALA	A	328	-26.285	-8.551	46.624	1.00	56.06	N
ATOM	2522	CA	ALA	A	328	-26.190	-9.247	45.342	1.00	63.09	C
ATOM	2523	C	ALA	A	328	-26.424	-10.753	45.480	1.00	65.87	C
ATOM	2524	O	ALA	A	328	-26.987	-11.382	44.579	1.00	71.17	O
ATOM	2525	CB	ALA	A	328	-24.834	-8.993	44.699	1.00	64.55	C
ATOM	2526	N	PHE	A	329	-26.007	-11.352	46.594	1.00	64.74	N
ATOM	2527	CA	PHE	A	329	-25.971	-12.808	46.657	1.00	54.43	C
ATOM	2528	C	PHE	A	329	-27.294	-13.426	47.054	1.00	57.32	C
ATOM	2529	O	PHE	A	329	-27.434	-14.648	46.954	1.00	62.00	O
ATOM	2530	CB	PHE	A	329	-24.882	-13.271	47.623	1.00	50.05	C
ATOM	2531	CG	PHE	A	329	-23.557	-12.631	47.372	1.00	59.67	C
ATOM	2532	CD1	PHE	A	329	-23.240	-11.394	47.942	1.00	61.29	C
ATOM	2533	CD2	PHE	A	329	-22.630	-13.249	46.544	1.00	54.73	C
ATOM	2534	CE1	PHE	A	329	-22.017	-10.778	47.696	1.00	53.93	C
ATOM	2535	CE2	PHE	A	329	-21.398	-12.648	46.279	1.00	54.91	C
ATOM	2536	CZ	PHE	A	329	-21.093	-11.401	46.864	1.00	55.51	C
ATOM	2537	N	LEU	A	330	-28.267	-12.637	47.497	1.00	65.26	N
ATOM	2538	CA	LEU	A	330	-29.502	-13.242	47.979	1.00	65.89	C
ATOM	2539	C	LEU	A	330	-30.473	-13.568	46.862	1.00	67.36	C
ATOM	2540	O	LEU	A	330	-31.487	-14.224	47.117	1.00	71.54	O
ATOM	2541	CB	LEU	A	330	-30.181	-12.352	49.034	1.00	72.32	C
ATOM	2542	CG	LEU	A	330	-29.252	-11.741	50.109	1.00	71.82	C
ATOM	2543	CD1	LEU	A	330	-29.932	-10.597	50.865	1.00	71.08	C
ATOM	2544	CD2	LEU	A	330	-28.730	-12.801	51.095	1.00	55.36	C
ATOM	2545	N	VAL	A	331	-30.184	-13.154	45.636	1.00	71.57	N
ATOM	2546	CA	VAL	A	331	-31.009	-13.577	44.519	1.00	67.88	C
ATOM	2547	C	VAL	A	331	-30.448	-14.852	43.936	1.00	69.40	C
ATOM	2548	O	VAL	A	331	-30.848	-15.270	42.845	1.00	80.72	O
ATOM	2549	CB	VAL	A	331	-31.109	-12.498	43.443	1.00	58.26	C
ATOM	2550	CG1	VAL	A	331	-32.038	-11.427	43.925	1.00	69.58	C
ATOM	2551	CG2	VAL	A	331	-29.734	-11.960	43.136	1.00	61.42	C
ATOM	2552	N	TYR	A	332	-29.526	-15.485	44.650	1.00	60.12	N
ATOM	2553	CA	TYR	A	332	-28.990	-16.762	44.199	1.00	75.41	C
ATOM	2554	C	TYR	A	332	-29.185	-17.878	45.219	1.00	80.40	C
ATOM	2555	O	TYR	A	332	-28.393	-18.831	45.251	1.00	66.65	O
ATOM	2556	CB	TYR	A	332	-27.510	-16.649	43.849	1.00	76.97	C
ATOM	2557	CG	TYR	A	332	-27.223	-15.897	42.583	1.00	83.12	C

ATOM	2558	CD1	TYR	A	332	-25.955	-15.536	42.273	1.00	78.93	C
ATOM	2559	CD2	TYR	A	332	-28.225	-15.575	41.696	1.00	82.62	C
ATOM	2560	CE1	TYR	A	332	-25.698	-14.869	41.122	1.00	71.93	C
ATOM	2561	CE2	TYR	A	332	-27.932	-14.891	40.561	1.00	82.64	C
ATOM	2562	CZ	TYR	A	332	-26.640	-14.535	40.311	1.00	76.01	C
ATOM	2563	OH	TYR	A	332	-26.321	-13.850	39.160	1.00	79.87	O
ATOM	2564	N	GLY	A	333	-30.224	-17.788	46.047	1.00	84.41	N
ATOM	2565	CA	GLY	A	333	-30.527	-18.861	46.966	1.00	74.10	C
ATOM	2566	C	GLY	A	333	-31.650	-18.545	47.927	1.00	70.85	C
ATOM	2567	O	GLY	A	333	-32.436	-19.432	48.271	1.00	78.13	O
ATOM	2568	N	ALA	A	334	-31.763	-17.290	48.345	1.00	73.74	N
ATOM	2569	CA	ALA	A	334	-32.512	-17.061	49.583	1.00	83.90	C
ATOM	2570	C	ALA	A	334	-34.016	-16.966	49.313	1.00	80.18	C
ATOM	2571	O	ALA	A	334	-34.440	-16.189	48.447	1.00	72.37	O
ATOM	2572	CB	ALA	A	334	-32.019	-15.803	50.290	1.00	73.86	C
ATOM	2573	N	PRO	A	335	-34.840	-17.705	50.061	1.00	75.98	N
ATOM	2574	CA	PRO	A	335	-36.265	-17.821	49.718	1.00	71.50	C
ATOM	2575	C	PRO	A	335	-37.010	-16.513	49.944	1.00	70.37	C
ATOM	2576	O	PRO	A	335	-36.878	-15.875	50.992	1.00	79.37	O
ATOM	2577	CB	PRO	A	335	-36.767	-18.920	50.663	1.00	74.39	C
ATOM	2578	CG	PRO	A	335	-35.526	-19.606	51.165	1.00	75.27	C
ATOM	2579	CD	PRO	A	335	-34.495	-18.529	51.227	1.00	73.08	C
ATOM	2580	N	GLY	A	336	-37.823	-16.139	48.960	1.00	68.38	N
ATOM	2581	CA	GLY	A	336	-38.540	-14.884	48.961	1.00	81.91	C
ATOM	2582	C	GLY	A	336	-37.855	-13.772	48.191	1.00	71.70	C
ATOM	2583	O	GLY	A	336	-38.432	-12.685	48.050	1.00	69.18	O
ATOM	2584	N	PHE	A	337	-36.652	-14.013	47.692	1.00	62.58	N
ATOM	2585	CA	PHE	A	337	-35.859	-12.990	47.035	1.00	68.65	C
ATOM	2586	C	PHE	A	337	-35.917	-13.167	45.526	1.00	72.37	C
ATOM	2587	O	PHE	A	337	-35.772	-14.277	45.012	1.00	77.70	O
ATOM	2588	CB	PHE	A	337	-34.404	-13.041	47.513	1.00	79.05	C
ATOM	2589	CG	PHE	A	337	-34.186	-12.434	48.879	1.00	75.74	C
ATOM	2590	CD1	PHE	A	337	-34.264	-13.215	50.019	1.00	76.45	C
ATOM	2591	CD2	PHE	A	337	-33.899	-11.086	49.018	1.00	75.31	C
ATOM	2592	CE1	PHE	A	337	-34.069	-12.661	51.272	1.00	86.52	C
ATOM	2593	CE2	PHE	A	337	-33.708	-10.521	50.273	1.00	81.71	C
ATOM	2594	CZ	PHE	A	337	-33.789	-11.306	51.400	1.00	81.77	C
ATOM	2595	N	SER	A	338	-36.125	-12.064	44.819	1.00	83.64	N
ATOM	2596	CA	SER	A	338	-36.157	-12.067	43.366	1.00	65.67	C
ATOM	2597	C	SER	A	338	-35.668	-10.724	42.851	1.00	62.90	C
ATOM	2598	O	SER	A	338	-35.872	-9.680	43.477	1.00	69.96	O
ATOM	2599	CB	SER	A	338	-37.565	-12.345	42.838	1.00	71.58	C
ATOM	2600	OG	SER	A	338	-37.723	-11.778	41.552	1.00	76.61	O
ATOM	2601	N	LYS	A	339	-35.020	-10.761	41.696	1.00	66.39	N

ATOM	2602	CA	LYS	A	339	-34.669	-9.538	40.988	1.00	70.40	C
ATOM	2603	C	LYS	A	339	-35.893	-8.773	40.476	1.00	68.43	C
ATOM	2604	O	LYS	A	339	-35.752	-7.629	40.038	1.00	74.52	O
ATOM	2605	CB	LYS	A	339	-33.737	-9.918	39.838	1.00	67.60	C
ATOM	2606	CG	LYS	A	339	-33.278	-8.801	38.957	1.00	68.69	C
ATOM	2607	CD	LYS	A	339	-32.274	-9.322	37.969	1.00	70.09	C
ATOM	2608	CE	LYS	A	339	-32.765	-10.546	37.235	1.00	62.34	C
ATOM	2609	NZ	LYS	A	339	-33.620	-10.116	36.102	1.00	63.91	N
ATOM	2610	N	ASP	A	340	-37.094	-9.346	40.552	1.00	67.79	N
ATOM	2611	CA	ASP	A	340	-38.247	-8.780	39.866	1.00	62.50	C
ATOM	2612	C	ASP	A	340	-39.391	-8.391	40.803	1.00	79.39	C
ATOM	2613	O	ASP	A	340	-40.518	-8.176	40.331	1.00	70.45	O
ATOM	2614	CB	ASP	A	340	-38.715	-9.757	38.793	1.00	59.33	C
ATOM	2615	CG	ASP	A	340	-37.611	-10.066	37.779	1.00	73.48	C
ATOM	2616	OD1	ASP	A	340	-37.028	-11.163	37.833	1.00	72.50	O
ATOM	2617	OD2	ASP	A	340	-37.300	-9.192	36.936	1.00	82.65	O
ATOM	2618	N	ASN	A	341	-39.131	-8.274	42.113	1.00	78.74	N
ATOM	2619	CA	ASN	A	341	-40.072	-7.659	43.046	1.00	74.72	C
ATOM	2620	C	ASN	A	341	-39.291	-7.041	44.203	1.00	80.15	C
ATOM	2621	O	ASN	A	341	-38.062	-7.154	44.285	1.00	73.39	O
ATOM	2622	CB	ASN	A	341	-41.134	-8.661	43.528	1.00	75.90	C
ATOM	2623	CG	ASN	A	341	-40.551	-9.830	44.324	1.00	84.14	C
ATOM	2624	OD1	ASN	A	341	-39.606	-9.670	45.091	1.00	86.44	O
ATOM	2625	ND2	ASN	A	341	-41.141	-11.013	44.159	1.00	86.57	N
ATOM	2626	N	ASN	A	342	-40.023	-6.378	45.108	1.00	85.30	N
ATOM	2627	CA	ASN	A	342	-39.394	-5.666	46.218	1.00	80.45	C
ATOM	2628	C	ASN	A	342	-38.835	-6.611	47.274	1.00	84.25	C
ATOM	2629	O	ASN	A	342	-38.033	-6.174	48.110	1.00	91.52	O
ATOM	2630	CB	ASN	A	342	-40.392	-4.685	46.840	1.00	80.96	C
ATOM	2631	CG	ASN	A	342	-41.783	-5.277	46.981	1.00	86.72	C
ATOM	2632	OD1	ASN	A	342	-42.272	-5.965	46.077	1.00	92.66	O
ATOM	2633	ND2	ASN	A	342	-42.436	-5.000	48.109	1.00	83.00	N
ATOM	2634	N	SER	A	343	-39.250	-7.886	47.257	1.00	87.62	N
ATOM	2635	CA	SER	A	343	-38.632	-8.962	48.048	1.00	84.17	C
ATOM	2636	C	SER	A	343	-38.828	-8.764	49.550	1.00	86.65	C
ATOM	2637	O	SER	A	343	-37.897	-8.957	50.337	1.00	87.07	O
ATOM	2638	CB	SER	A	343	-37.141	-9.115	47.726	1.00	78.64	C
ATOM	2639	OG	SER	A	343	-36.929	-9.574	46.408	1.00	70.26	O
ATOM	2640	N	ILE	A	344	-40.042	-8.401	49.957	1.00	84.24	N
ATOM	2641	CA	ILE	A	344	-40.343	-8.183	51.373	1.00	91.87	C
ATOM	2642	C	ILE	A	344	-40.468	-9.556	52.030	1.00	79.60	C
ATOM	2643	O	ILE	A	344	-41.481	-10.234	51.867	1.00	79.82	O
ATOM	2644	CB	ILE	A	344	-41.607	-7.338	51.561	1.00	88.52	C
ATOM	2645	CG1	ILE	A	344	-41.479	-6.038	50.771	1.00	81.53	C

ATOM	2646	CG2	ILE	A	344	-41.837	-7.016	53.027	1.00	91.35	C
ATOM	2647	CD1	ILE	A	344	-40.224	-5.250	51.095	1.00	77.74	C
ATOM	2648	N	ILE	A	345	-39.438	-9.982	52.766	1.00	84.53	N
ATOM	2649	CA	ILE	A	345	-39.476	-11.302	53.387	1.00	92.13	C
ATOM	2650	C	ILE	A	345	-40.145	-11.217	54.752	1.00	86.69	C
ATOM	2651	O	ILE	A	345	-40.160	-10.174	55.417	1.00	79.76	O
ATOM	2652	CB	ILE	A	345	-38.092	-11.977	53.525	1.00	78.97	C
ATOM	2653	CG1	ILE	A	345	-37.064	-11.019	54.121	1.00	77.97	C
ATOM	2654	CG2	ILE	A	345	-37.639	-12.565	52.205	1.00	76.28	C
ATOM	2655	CD1	ILE	A	345	-35.851	-11.726	54.623	1.00	68.00	C
ATOM	2656	N	THR	A	346	-40.706	-12.352	55.160	1.00	85.36	N
ATOM	2657	CA	THR	A	346	-41.190	-12.600	56.507	1.00	90.34	C
ATOM	2658	C	THR	A	346	-40.109	-13.332	57.301	1.00	94.23	C
ATOM	2659	O	THR	A	346	-39.207	-13.952	56.730	1.00	94.52	O
ATOM	2660	CB	THR	A	346	-42.486	-13.423	56.489	1.00	89.32	C
ATOM	2661	OG1	THR	A	346	-42.894	-13.692	57.834	1.00111.94		O
ATOM	2662	CG2	THR	A	346	-42.268	-14.761	55.793	1.00	84.80	C
ATOM	2663	N	ARG	A	347	-40.222	-13.259	58.636	1.00	93.12	N
ATOM	2664	CA	ARG	A	347	-39.149	-13.693	59.533	1.00	84.82	C
ATOM	2665	C	ARG	A	347	-38.772	-15.136	59.301	1.00	85.11	C
ATOM	2666	O	ARG	A	347	-37.590	-15.505	59.389	1.00	84.60	O
ATOM	2667	CB	ARG	A	347	-39.546	-13.491	60.999	1.00	82.39	C
ATOM	2668	CG	ARG	A	347	-39.019	-14.541	61.993	1.00	88.11	C
ATOM	2669	CD	ARG	A	347	-39.148	-13.972	63.430	1.00	94.91	C
ATOM	2670	NE	ARG	A	347	-38.235	-14.590	64.408	1.00101.77		N
ATOM	2671	CZ	ARG	A	347	-37.610	-13.930	65.387	1.00	84.57	C
ATOM	2672	NH1	ARG	A	347	-36.838	-14.593	66.244	1.00	89.77	N
ATOM	2673	NH2	ARG	A	347	-37.737	-12.614	65.481	1.00	66.51	N
ATOM	2674	N	LYS	A	348	-39.789	-15.967	59.044	1.00	95.91	N
ATOM	2675	CA	LYS	A	348	-39.535	-17.374	58.765	1.00	95.75	C
ATOM	2676	C	LYS	A	348	-38.661	-17.536	57.529	1.00	91.03	C
ATOM	2677	O	LYS	A	348	-37.718	-18.345	57.520	1.00	86.55	O
ATOM	2678	CB	LYS	A	348	-40.849	-18.108	58.576	1.00	84.45	C
ATOM	2679	CG	LYS	A	348	-40.667	-19.471	57.983	1.00	90.56	C
ATOM	2680	CD	LYS	A	348	-41.592	-19.706	56.813	1.00	89.22	C
ATOM	2681	CE	LYS	A	348	-40.985	-20.689	55.835	1.00	84.61	C
ATOM	2682	NZ	LYS	A	348	-42.050	-21.123	54.907	1.00	91.86	N
ATOM	2683	N	GLU	A	349	-38.966	-16.776	56.472	1.00	84.25	N
ATOM	2684	CA	GLU	A	349	-38.096	-16.770	55.305	1.00	86.03	C
ATOM	2685	C	GLU	A	349	-36.703	-16.285	55.681	1.00	79.22	C
ATOM	2686	O	GLU	A	349	-35.697	-16.779	55.152	1.00	79.17	O
ATOM	2687	CB	GLU	A	349	-38.701	-15.899	54.197	1.00	87.80	C
ATOM	2688	CG	GLU	A	349	-39.991	-16.426	53.574	1.00	80.25	C
ATOM	2689	CD	GLU	A	349	-40.684	-15.379	52.700	1.00	97.62	C

```
ATOM   2690  OE1 GLU A 349     -40.289 -14.193  52.740  1.00 90.77           O
ATOM   2691  OE2 GLU A 349     -41.633 -15.738  51.971  1.00110.63           O
ATOM   2692  N   PHE A 350     -36.624 -15.340  56.612  1.00 67.92           N
ATOM   2693  CA  PHE A 350     -35.321 -14.865  57.046  1.00 74.44           C
ATOM   2694  C   PHE A 350     -34.547 -15.974  57.732  1.00 76.49           C
ATOM   2695  O   PHE A 350     -33.321 -16.065  57.593  1.00 75.36           O
ATOM   2696  CB  PHE A 350     -35.473 -13.668  57.987  1.00 78.98           C
ATOM   2697  CG  PHE A 350     -34.177 -13.212  58.584  1.00 68.12           C
ATOM   2698  CD1 PHE A 350     -33.464 -12.193  58.010  1.00 58.24           C
ATOM   2699  CD2 PHE A 350     -33.652 -13.837  59.700  1.00 72.50           C
ATOM   2700  CE1 PHE A 350     -32.261 -11.792  58.556  1.00 66.91           C
ATOM   2701  CE2 PHE A 350     -32.444 -13.446  60.234  1.00 60.93           C
ATOM   2702  CZ  PHE A 350     -31.760 -12.419  59.681  1.00 63.39           C
ATOM   2703  N   GLN A 351     -35.244 -16.798  58.512  1.00 85.25           N
ATOM   2704  CA  GLN A 351     -34.591 -17.904  59.195  1.00 79.69           C
ATOM   2705  C   GLN A 351     -34.145 -18.972  58.207  1.00 81.78           C
ATOM   2706  O   GLN A 351     -33.080 -19.577  58.382  1.00 85.60           O
ATOM   2707  CB  GLN A 351     -35.529 -18.485  60.247  1.00 83.09           C
ATOM   2708  CG  GLN A 351     -35.717 -17.562  61.436  1.00 86.88           C
ATOM   2709  CD  GLN A 351     -36.760 -18.064  62.401  1.00 82.27           C
ATOM   2710  OE1 GLN A 351     -37.888 -18.355  62.002  1.00 89.27           O
ATOM   2711  NE2 GLN A 351     -36.393 -18.174  63.676  1.00 64.29           N
ATOM   2712  N   GLU A 352     -34.923 -19.210  57.151  1.00 75.77           N
ATOM   2713  CA  GLU A 352     -34.427 -20.167  56.175  1.00 86.95           C
ATOM   2714  C   GLU A 352     -33.347 -19.543  55.298  1.00 74.54           C
ATOM   2715  O   GLU A 352     -32.416 -20.245  54.885  1.00 73.51           O
ATOM   2716  CB  GLU A 352     -35.565 -20.741  55.329  1.00 81.88           C
ATOM   2717  CG  GLU A 352     -35.089 -21.945  54.523  1.00 91.35           C
ATOM   2718  CD  GLU A 352     -36.045 -22.353  53.414  1.00111.56           C
ATOM   2719  OE1 GLU A 352     -35.686 -23.266  52.630  1.00109.95           O
ATOM   2720  OE2 GLU A 352     -37.148 -21.764  53.332  1.00115.27           O
ATOM   2721  N   GLY A 353     -33.422 -18.235  55.042  1.00 66.86           N
ATOM   2722  CA  GLY A 353     -32.306 -17.565  54.394  1.00 72.11           C
ATOM   2723  C   GLY A 353     -30.987 -17.803  55.110  1.00 73.79           C
ATOM   2724  O   GLY A 353     -29.959 -18.063  54.474  1.00 67.88           O
ATOM   2725  N   LEU A 354     -31.004 -17.744  56.447  1.00 72.44           N
ATOM   2726  CA  LEU A 354     -29.773 -17.898  57.217  1.00 69.84           C
ATOM   2727  C   LEU A 354     -29.160 -19.283  57.040  1.00 81.52           C
ATOM   2728  O   LEU A 354     -27.932 -19.399  56.933  1.00 84.51           O
ATOM   2729  CB  LEU A 354     -30.017 -17.641  58.702  1.00 76.38           C
ATOM   2730  CG  LEU A 354     -30.090 -16.238  59.288  1.00 71.03           C
ATOM   2731  CD1 LEU A 354     -29.856 -16.368  60.774  1.00 78.54           C
ATOM   2732  CD2 LEU A 354     -29.063 -15.335  58.673  1.00 62.93           C
ATOM   2733  N   LYS A 355     -29.987 -20.355  57.042  1.00 75.82           N
```

ATOM	2734	CA	LYS	A	355	-29.444	-21.705	56.820	1.00	77.46	C
ATOM	2735	C	LYS	A	355	-28.823	-21.879	55.448	1.00	82.86	C
ATOM	2736	O	LYS	A	355	-27.953	-22.739	55.292	1.00	84.93	O
ATOM	2737	CB	LYS	A	355	-30.472	-22.843	56.976	1.00	75.49	C
ATOM	2738	CG	LYS	A	355	-30.507	-23.558	58.353	1.00	87.50	C
ATOM	2739	CD	LYS	A	355	-29.183	-24.309	58.687	1.00	97.62	C
ATOM	2740	CE	LYS	A	355	-28.775	-24.262	60.220	1.00101.98		C
ATOM	2741	NZ	LYS	A	355	-27.567	-25.028	60.572	1.00104.50		N
ATOM	2742	N	ILE	A	356	-29.305	-21.189	54.417	1.00	72.10	N
ATOM	2743	CA	ILE	A	356	-28.582	-21.340	53.168	1.00	69.33	C
ATOM	2744	C	ILE	A	356	-27.206	-20.713	53.300	1.00	68.83	C
ATOM	2745	O	ILE	A	356	-26.200	-21.296	52.883	1.00	76.44	O
ATOM	2746	CB	ILE	A	356	-29.380	-20.771	51.989	1.00	71.60	C
ATOM	2747	CG1	ILE	A	356	-30.787	-21.366	51.982	1.00	69.17	C
ATOM	2748	CG2	ILE	A	356	-28.674	-21.079	50.704	1.00	80.82	C
ATOM	2749	CD1	ILE	A	356	-31.739	-20.632	51.079	1.00	70.28	C
ATOM	2750	N	PHE	A	357	-27.126	-19.566	53.953	1.00	60.86	N
ATOM	2751	CA	PHE	A	357	-25.873	-18.840	54.024	1.00	69.67	C
ATOM	2752	C	PHE	A	357	-25.010	-19.268	55.197	1.00	59.79	C
ATOM	2753	O	PHE	A	357	-23.870	-18.816	55.310	1.00	67.39	O
ATOM	2754	CB	PHE	A	357	-26.158	-17.336	54.067	1.00	67.76	C
ATOM	2755	CG	PHE	A	357	-26.515	-16.752	52.732	1.00	56.16	C
ATOM	2756	CD1	PHE	A	357	-25.539	-16.268	51.896	1.00	52.43	C
ATOM	2757	CD2	PHE	A	357	-27.831	-16.715	52.301	1.00	63.85	C
ATOM	2758	CE1	PHE	A	357	-25.866	-15.724	50.659	1.00	56.01	C
ATOM	2759	CE2	PHE	A	357	-28.164	-16.168	51.064	1.00	60.52	C
ATOM	2760	CZ	PHE	A	357	-27.174	-15.680	50.243	1.00	56.37	C
ATOM	2761	N	PHE	A	358	-25.517	-20.138	56.057	1.00	58.98	N
ATOM	2762	CA	PHE	A	358	-24.746	-20.683	57.172	1.00	69.91	C
ATOM	2763	C	PHE	A	358	-25.224	-22.118	57.380	1.00	83.11	C
ATOM	2764	O	PHE	A	358	-25.765	-22.460	58.426	1.00	92.94	O
ATOM	2765	CB	PHE	A	358	-24.962	-19.865	58.456	1.00	69.40	C
ATOM	2766	CG	PHE	A	358	-24.631	-18.400	58.361	1.00	61.78	C
ATOM	2767	CD1	PHE	A	358	-23.341	-17.926	58.599	1.00	61.69	C
ATOM	2768	CD2	PHE	A	358	-25.637	-17.478	58.126	1.00	63.31	C
ATOM	2769	CE1	PHE	A	358	-23.058	-16.554	58.529	1.00	56.76	C
ATOM	2770	CE2	PHE	A	358	-25.359	-16.105	58.062	1.00	61.59	C
ATOM	2771	CZ	PHE	A	358	-24.071	-15.651	58.263	1.00	60.01	C
ATOM	2772	N	PRO	A	359	-25.036	-22.997	56.406	1.00	80.42	N
ATOM	2773	CA	PRO	A	359	-25.779	-24.269	56.457	1.00	89.93	C
ATOM	2774	C	PRO	A	359	-25.446	-25.112	57.675	1.00	93.32	C
ATOM	2775	O	PRO	A	359	-26.316	-25.841	58.175	1.00100.81		O
ATOM	2776	CB	PRO	A	359	-25.417	-24.938	55.124	1.00	89.13	C
ATOM	2777	CG	PRO	A	359	-24.082	-24.339	54.739	1.00	83.85	C

```
ATOM   2778  CD  PRO A 359     -23.890 -23.044  55.502  1.00 81.97           C
ATOM   2779  N   GLY A 360     -24.231 -25.003  58.191  1.00 81.36           N
ATOM   2780  CA  GLY A 360     -23.947 -25.662  59.431  1.00 96.66           C
ATOM   2781  C   GLY A 360     -22.494 -25.615  59.830  1.00113.99           C
ATOM   2782  O   GLY A 360     -21.684 -26.206  59.102  1.00129.65           O
ATOM   2783  N   VAL A 361     -22.102 -24.931  60.920  1.00111.71           N
ATOM   2784  CA  VAL A 361     -22.861 -23.990  61.792  1.00107.00           C
ATOM   2785  C   VAL A 361     -24.148 -24.530  62.493  1.00106.07           C
ATOM   2786  O   VAL A 361     -25.171 -24.858  61.877  1.00 93.11           O
ATOM   2787  CB  VAL A 361     -23.172 -22.656  61.021  1.00 95.86           C
ATOM   2788  CG1 VAL A 361     -23.867 -21.676  61.933  1.00 75.88           C
ATOM   2789  CG2 VAL A 361     -21.888 -22.025  60.478  1.00 86.98           C
ATOM   2790  N   SER A 362     -24.056 -24.561  63.824  1.00112.83           N
ATOM   2791  CA  SER A 362     -25.073 -25.118  64.710  1.00112.49           C
ATOM   2792  C   SER A 362     -26.371 -24.317  64.650  1.00 98.47           C
ATOM   2793  O   SER A 362     -26.399 -23.164  64.222  1.00 98.24           O
ATOM   2794  CB  SER A 362     -24.569 -25.134  66.153  1.00108.93           C
ATOM   2795  OG  SER A 362     -24.447 -23.809  66.650  1.00 93.48           O
ATOM   2796  N   GLU A 363     -27.460 -24.941  65.113  1.00102.14           N
ATOM   2797  CA  GLU A 363     -28.748 -24.261  65.070  1.00 95.07           C
ATOM   2798  C   GLU A 363     -28.729 -23.043  65.958  1.00 98.26           C
ATOM   2799  O   GLU A 363     -29.316 -22.011  65.618  1.00112.92           O
ATOM   2800  CB  GLU A 363     -29.896 -25.184  65.484  1.00103.93           C
ATOM   2801  CG  GLU A 363     -31.321 -24.595  65.278  1.00 96.76           C
ATOM   2802  CD  GLU A 363     -31.652 -23.454  66.228  1.00104.17           C
ATOM   2803  OE1 GLU A 363     -32.120 -22.385  65.762  1.00111.89           O
ATOM   2804  OE2 GLU A 363     -31.395 -23.612  67.440  1.00 98.30           O
ATOM   2805  N   PHE A 364     -28.074 -23.120  67.101  1.00 90.90           N
ATOM   2806  CA  PHE A 364     -28.135 -21.925  67.913  1.00 95.35           C
ATOM   2807  C   PHE A 364     -27.367 -20.772  67.269  1.00 96.64           C
ATOM   2808  O   PHE A 364     -27.892 -19.656  67.180  1.00 92.01           O
ATOM   2809  CB  PHE A 364     -27.627 -22.137  69.314  1.00108.43           C
ATOM   2810  CG  PHE A 364     -27.746 -20.905  70.071  1.00112.92           C
ATOM   2811  CD1 PHE A 364     -26.876 -20.570  71.050  1.00111.08           C
ATOM   2812  CD2 PHE A 364     -28.784 -20.032  69.756  1.00115.51           C
ATOM   2813  CE1 PHE A 364     -27.025 -19.380  71.642  1.00115.48           C
ATOM   2814  CE2 PHE A 364     -28.915 -18.856  70.371  1.00118.67           C
ATOM   2815  CZ  PHE A 364     -28.033 -18.542  71.335  1.00124.79           C
ATOM   2816  N   GLY A 365     -26.125 -21.010  66.836  1.00105.99           N
ATOM   2817  CA  GLY A 365     -25.346 -19.992  66.145  1.00 96.23           C
ATOM   2818  C   GLY A 365     -26.230 -19.100  65.300  1.00 82.21           C
ATOM   2819  O   GLY A 365     -26.079 -17.877  65.296  1.00 61.28           O
ATOM   2820  N   LYS A 366     -27.224 -19.708  64.645  1.00 83.51           N
ATOM   2821  CA  LYS A 366     -28.170 -18.922  63.866  1.00 87.90           C
```

```
ATOM   2822  C   LYS A 366     -29.069 -18.082  64.747  1.00 74.84           C
ATOM   2823  O   LYS A 366     -29.312 -16.913  64.442  1.00 81.11           O
ATOM   2824  CB  LYS A 366     -28.971 -19.817  62.915  1.00 88.36           C
ATOM   2825  CG  LYS A 366     -28.122 -20.106  61.766  1.00 93.46           C
ATOM   2826  CD  LYS A 366     -28.397 -21.394  61.115  1.00 98.47           C
ATOM   2827  CE  LYS A 366     -27.081 -22.011  60.976  1.00105.71           C
ATOM   2828  NZ  LYS A 366     -26.514 -22.131  62.301  1.00102.56           N
ATOM   2829  N   GLU A 367     -29.533 -18.611  65.861  1.00 81.60           N
ATOM   2830  CA  GLU A 367     -30.482 -17.783  66.581  1.00 84.62           C
ATOM   2831  C   GLU A 367     -29.788 -16.553  67.150  1.00 81.30           C
ATOM   2832  O   GLU A 367     -30.374 -15.463  67.191  1.00 74.57           O
ATOM   2833  CB  GLU A 367     -31.187 -18.597  67.653  1.00 93.43           C
ATOM   2834  CG  GLU A 367     -32.663 -18.410  67.555  1.00 93.15           C
ATOM   2835  CD  GLU A 367     -33.081 -17.084  68.123  1.00 83.86           C
ATOM   2836  OE1 GLU A 367     -32.224 -16.400  68.716  1.00 88.93           O
ATOM   2837  OE2 GLU A 367     -34.256 -16.704  67.952  1.00 77.91           O
ATOM   2838  N   SER A 368     -28.511 -16.677  67.495  1.00 84.00           N
ATOM   2839  CA  SER A 368     -27.839 -15.496  67.998  1.00 74.92           C
ATOM   2840  C   SER A 368     -27.504 -14.529  66.866  1.00 76.86           C
ATOM   2841  O   SER A 368     -27.556 -13.317  67.083  1.00 77.59           O
ATOM   2842  CB  SER A 368     -26.579 -15.860  68.781  1.00 75.80           C
ATOM   2843  OG  SER A 368     -25.538 -16.277  67.934  1.00 86.68           O
ATOM   2844  N   ILE A 369     -27.179 -15.015  65.653  1.00 82.66           N
ATOM   2845  CA  ILE A 369     -27.123 -14.108  64.502  1.00 63.60           C
ATOM   2846  C   ILE A 369     -28.460 -13.432  64.321  1.00 67.92           C
ATOM   2847  O   ILE A 369     -28.526 -12.209  64.155  1.00 77.09           O
ATOM   2848  CB  ILE A 369     -26.726 -14.817  63.194  1.00 65.20           C
ATOM   2849  CG1 ILE A 369     -25.424 -15.604  63.296  1.00 84.00           C
ATOM   2850  CG2 ILE A 369     -26.589 -13.808  62.063  1.00 76.87           C
ATOM   2851  CD1 ILE A 369     -25.083 -16.249  61.970  1.00 65.80           C
ATOM   2852  N   LEU A 370     -29.541 -14.221  64.323  1.00 64.23           N
ATOM   2853  CA  LEU A 370     -30.847 -13.607  64.161  1.00 67.57           C
ATOM   2854  C   LEU A 370     -30.996 -12.543  65.223  1.00 69.37           C
ATOM   2855  O   LEU A 370     -30.994 -11.355  64.913  1.00 81.00           O
ATOM   2856  CB  LEU A 370     -31.970 -14.642  64.246  1.00 68.84           C
ATOM   2857  CG  LEU A 370     -33.457 -14.299  64.395  1.00 66.34           C
ATOM   2858  CD1 LEU A 370     -33.954 -13.801  63.104  1.00 76.14           C
ATOM   2859  CD2 LEU A 370     -34.250 -15.524  64.709  1.00 91.20           C
ATOM   2860  N   PHE A 371     -30.900 -12.938  66.489  1.00 82.82           N
ATOM   2861  CA  PHE A 371     -31.071 -11.985  67.581  1.00 88.74           C
ATOM   2862  C   PHE A 371     -30.370 -10.665  67.285  1.00 82.88           C
ATOM   2863  O   PHE A 371     -31.028  -9.659  67.040  1.00 78.89           O
ATOM   2864  CB  PHE A 371     -30.569 -12.575  68.900  1.00 79.23           C
ATOM   2865  CG  PHE A 371     -30.581 -11.591  70.031  1.00 75.08           C
```

```
ATOM   2866  CD1 PHE A 371     -31.786 -11.224  70.608  1.00 83.29           C
ATOM   2867  CD2 PHE A 371     -29.412 -11.011  70.502  1.00 77.28           C
ATOM   2868  CE1 PHE A 371     -31.847 -10.294  71.649  1.00 68.29           C
ATOM   2869  CE2 PHE A 371     -29.459 -10.068  71.553  1.00 80.23           C
ATOM   2870  CZ  PHE A 371     -30.683  -9.724  72.129  1.00 67.68           C
ATOM   2871  N   HIS A 372     -29.040 -10.690  67.157  1.00 84.03           N
ATOM   2872  CA  HIS A 372     -28.254  -9.458  67.095  0.91 76.08           C
ATOM   2874  C   HIS A 372     -28.721  -8.515  65.983  1.00 75.81           C
ATOM   2875  O   HIS A 372     -28.484  -7.309  66.064  1.00 80.08           O
ATOM   2876  CB  HIS A 372     -26.765  -9.805  66.933  0.91 74.30           C
ATOM   2878  CG  HIS A 372     -25.841  -8.618  66.935  0.91 76.40           C
ATOM   2880  ND1 HIS A 372     -25.063  -8.271  68.021  0.91 79.48           N
ATOM   2882  CD2 HIS A 372     -25.546  -7.716  65.967  0.91 80.56           C
ATOM   2884  CE1 HIS A 372     -24.343  -7.201  67.726  0.91 77.45           C
ATOM   2886  NE2 HIS A 372     -24.615  -6.847  66.484  0.91 79.30           N
ATOM   2888  N   TYR A 373     -29.377  -9.019  64.942  1.00 77.47           N
ATOM   2889  CA  TYR A 373     -29.792  -8.134  63.854  1.00 72.65           C
ATOM   2890  C   TYR A 373     -31.279  -7.820  63.875  1.00 81.76           C
ATOM   2891  O   TYR A 373     -31.679  -6.736  63.444  1.00 89.13           O
ATOM   2892  CB  TYR A 373     -29.424  -8.749  62.495  1.00 64.63           C
ATOM   2893  CG  TYR A 373     -28.001  -8.481  62.046  1.00 61.28           C
ATOM   2894  CD1 TYR A 373     -27.725  -7.709  60.923  1.00 58.05           C
ATOM   2895  CD2 TYR A 373     -26.925  -8.958  62.778  1.00 65.54           C
ATOM   2896  CE1 TYR A 373     -26.390  -7.456  60.521  1.00 61.20           C
ATOM   2897  CE2 TYR A 373     -25.602  -8.703  62.387  1.00 63.68           C
ATOM   2898  CZ  TYR A 373     -25.336  -7.955  61.266  1.00 56.20           C
ATOM   2899  OH  TYR A 373     -24.020  -7.710  60.898  1.00 53.40           O
ATOM   2900  N   THR A 374     -32.112  -8.731  64.371  1.00 86.68           N
ATOM   2901  CA  THR A 374     -33.560  -8.513  64.425  1.00 90.07           C
ATOM   2902  C   THR A 374     -33.979  -7.358  65.301  1.00 94.76           C
ATOM   2903  O   THR A 374     -35.190  -7.141  65.546  1.00 95.18           O
ATOM   2904  CB  THR A 374     -34.280  -9.729  64.990  1.00 90.59           C
ATOM   2905  OG1 THR A 374     -34.209  -9.685  66.418  1.00 97.66           O
ATOM   2906  CG2 THR A 374     -33.698 -10.926  64.546  1.00 87.26           C
ATOM   2907  N   ASP A 375     -33.041  -6.595  65.817  1.00 82.43           N
ATOM   2908  CA  ASP A 375     -33.417  -5.679  66.890  0.71 97.41           C
ATOM   2910  C   ASP A 375     -33.988  -4.440  66.240  1.00101.31           C
ATOM   2911  O   ASP A 375     -33.358  -3.385  66.108  1.00 93.89           O
ATOM   2912  CB  ASP A 375     -32.270  -5.350  67.821  0.71102.67           C
ATOM   2914  CG  ASP A 375     -32.666  -4.312  68.863  0.71 96.03           C
ATOM   2916  OD1 ASP A 375     -33.863  -4.304  69.198  0.71 97.25           O
ATOM   2918  OD2 ASP A 375     -31.822  -3.509  69.323  0.71 82.08           O
ATOM   2920  N   TRP A 376     -35.245  -4.615  65.828  1.00109.62           N
ATOM   2921  CA  TRP A 376     -35.923  -3.638  65.007  1.00101.63           C
```

```
ATOM   2922  C   TRP A 376     -35.899  -2.294  65.669  1.00 96.17           C
ATOM   2923  O   TRP A 376     -35.904  -2.168  66.896  1.00107.03           O
ATOM   2924  CB  TRP A 376     -37.355  -4.064  64.683  1.00 98.56           C
ATOM   2925  CG  TRP A 376     -37.361  -5.256  63.844  1.00 99.97           C
ATOM   2926  CD1 TRP A 376     -38.239  -6.299  63.895  1.00110.42           C
ATOM   2927  CD2 TRP A 376     -36.383  -5.595  62.858  1.00 92.86           C
ATOM   2928  NE1 TRP A 376     -37.887  -7.253  62.973  1.00105.70           N
ATOM   2929  CE2 TRP A 376     -36.743  -6.847  62.335  1.00102.16           C
ATOM   2930  CE3 TRP A 376     -35.239  -4.955  62.357  1.00 83.04           C
ATOM   2931  CZ2 TRP A 376     -36.005  -7.469  61.342  1.00103.44           C
ATOM   2932  CZ3 TRP A 376     -34.511  -5.571  61.393  1.00 95.61           C
ATOM   2933  CH2 TRP A 376     -34.895  -6.815  60.884  1.00102.43           C
ATOM   2934  N   VAL A 377     -35.796  -1.309  64.818  1.00101.81           N
ATOM   2935  CA  VAL A 377     -35.884   0.060  65.152  1.00106.52           C
ATOM   2936  C   VAL A 377     -37.086   0.600  64.341  1.00108.86           C
ATOM   2937  O   VAL A 377     -36.883   1.419  63.441  1.00 93.66           O
ATOM   2938  CB  VAL A 377     -34.513   0.732  64.831  1.00103.09           C
ATOM   2939  CG1 VAL A 377     -34.021   0.322  63.447  1.00102.77           C
ATOM   2940  CG2 VAL A 377     -34.556   2.246  64.940  1.00 96.04           C
ATOM   2941  N   ASP A 378     -38.341   0.151  64.570  1.00117.82           N
ATOM   2942  CA  ASP A 378     -38.850  -0.962  65.413  1.00119.23           C
ATOM   2943  C   ASP A 378     -39.651  -1.931  64.489  1.00118.04           C
ATOM   2944  O   ASP A 378     -39.485  -1.880  63.263  1.00112.97           O
ATOM   2945  CB  ASP A 378     -39.732  -0.418  66.554  1.00109.72           C
ATOM   2946  CG  ASP A 378     -40.022  -1.455  67.644  1.00119.49           C
ATOM   2947  OD1 ASP A 378     -41.111  -2.064  67.602  1.00115.51           O
ATOM   2948  OD2 ASP A 378     -39.182  -1.642  68.553  1.00121.83           O
ATOM   2949  N   ASP A 379     -40.525  -2.788  65.047  1.00122.04           N
ATOM   2950  CA  ASP A 379     -41.166  -3.869  64.284  1.00122.92           C
ATOM   2951  C   ASP A 379     -42.091  -3.363  63.167  1.00112.29           C
ATOM   2952  O   ASP A 379     -42.790  -4.159  62.525  1.00101.29           O
ATOM   2953  CB  ASP A 379     -41.933  -4.825  65.233  1.00116.99           C
ATOM   2954  CG  ASP A 379     -43.151  -4.175  65.919  1.00123.72           C
ATOM   2955  OD1 ASP A 379     -43.141  -2.955  66.206  1.00122.33           O
ATOM   2956  OD2 ASP A 379     -44.136  -4.905  66.173  1.00121.54           O
ATOM   2957  N   GLN A 380     -42.055  -2.051  62.898  1.00108.59           N
ATOM   2958  CA  GLN A 380     -42.989  -1.360  62.011  1.00114.14           C
ATOM   2959  C   GLN A 380     -42.882  -1.887  60.574  1.00118.56           C
ATOM   2960  O   GLN A 380     -43.499  -2.897  60.206  1.00110.94           O
ATOM   2961  CB  GLN A 380     -42.739   0.172  62.085  1.00104.61           C
ATOM   2962  CG  GLN A 380     -41.355   0.681  61.574  1.00110.38           C
ATOM   2963  CD  GLN A 380     -40.326   0.951  62.620  1.00114.78           C
ATOM   2964  OE1 GLN A 380     -40.590   0.825  63.804  1.00119.86           O
ATOM   2965  NE2 GLN A 380     -39.100   1.231  62.181  1.00113.79           N
```

```
ATOM   2966  N   ARG A 381     -42.131  -1.153  59.766  1.00111.30           N
ATOM   2967  CA  ARG A 381     -41.514  -1.298  58.461  1.00102.02           C
ATOM   2968  C   ARG A 381     -41.462  -2.728  57.935  1.00102.77           C
ATOM   2969  O   ARG A 381     -40.870  -3.616  58.567  1.00105.57           O
ATOM   2970  CB  ARG A 381     -40.113  -0.703  58.575  1.00 97.02           C
ATOM   2971  CG  ARG A 381     -39.334  -0.629  57.331  1.00102.51           C
ATOM   2972  CD  ARG A 381     -37.868  -0.599  57.672  1.00100.94           C
ATOM   2973  NE  ARG A 381     -37.090  -0.135  56.539  1.00104.53           N
ATOM   2974  CZ  ARG A 381     -36.868   1.148  56.295  1.00107.06           C
ATOM   2975  NH1 ARG A 381     -36.157   1.509  55.230  1.00104.67           N
ATOM   2976  NH2 ARG A 381     -37.358   2.064  57.128  1.00 89.93           N
ATOM   2977  N   PRO A 382     -42.056  -2.978  56.765  1.00 97.08           N
ATOM   2978  CA  PRO A 382     -42.038  -4.338  56.212  1.00103.07           C
ATOM   2979  C   PRO A 382     -40.679  -4.724  55.659  1.00 99.47           C
ATOM   2980  O   PRO A 382     -40.360  -5.918  55.586  1.00 95.27           O
ATOM   2981  CB  PRO A 382     -43.101  -4.276  55.102  1.00 88.82           C
ATOM   2982  CG  PRO A 382     -43.822  -2.970  55.309  1.00 81.42           C
ATOM   2983  CD  PRO A 382     -42.814  -2.061  55.907  1.00 87.46           C
ATOM   2984  N   GLU A 383     -39.862  -3.734  55.293  1.00 97.62           N
ATOM   2985  CA  GLU A 383     -38.589  -3.981  54.631  1.00 84.49           C
ATOM   2986  C   GLU A 383     -37.570  -4.655  55.528  1.00 82.59           C
ATOM   2987  O   GLU A 383     -36.590  -5.198  55.013  1.00 78.03           O
ATOM   2988  CB  GLU A 383     -37.989  -2.661  54.127  1.00 89.87           C
ATOM   2989  CG  GLU A 383     -38.825  -1.932  53.080  1.00 94.20           C
ATOM   2990  CD  GLU A 383     -39.696  -0.839  53.673  1.00 98.72           C
ATOM   2991  OE1 GLU A 383     -40.927  -0.862  53.440  1.00 93.92           O
ATOM   2992  OE2 GLU A 383     -39.143   0.061  54.344  1.00 91.60           O
ATOM   2993  N   ASN A 384     -37.761  -4.628  56.850  1.00 95.91           N
ATOM   2994  CA  ASN A 384     -36.588  -4.748  57.707  1.00 95.89           C
ATOM   2995  C   ASN A 384     -36.018  -6.163  57.768  1.00 92.56           C
ATOM   2996  O   ASN A 384     -34.799  -6.308  57.924  1.00 93.53           O
ATOM   2997  CB  ASN A 384     -36.876  -4.211  59.106  1.00 88.51           C
ATOM   2998  CG  ASN A 384     -36.031  -2.992  59.418  1.00 96.71           C
ATOM   2999  OD1 ASN A 384     -34.910  -2.861  58.913  1.00100.32           O
ATOM   3000  ND2 ASN A 384     -36.552  -2.097  60.255  1.00103.57           N
ATOM   3001  N   TYR A 385     -36.830  -7.214  57.613  1.00 83.53           N
ATOM   3002  CA  TYR A 385     -36.221  -8.530  57.455  1.00 79.05           C
ATOM   3003  C   TYR A 385     -35.359  -8.579  56.200  1.00 79.58           C
ATOM   3004  O   TYR A 385     -34.220  -9.057  56.235  1.00 74.99           O
ATOM   3005  CB  TYR A 385     -37.282  -9.621  57.438  1.00 86.89           C
ATOM   3006  CG  TYR A 385     -37.735  -9.966  58.834  1.00 95.95           C
ATOM   3007  CD1 TYR A 385     -36.836 -10.464  59.776  1.00 88.99           C
ATOM   3008  CD2 TYR A 385     -39.055  -9.780  59.221  1.00 99.58           C
ATOM   3009  CE1 TYR A 385     -37.242 -10.766  61.072  1.00 85.57           C
```

```
ATOM   3010  CE2 TYR A 385     -39.473 -10.079  60.505  1.00 96.88           C
ATOM   3011  CZ  TYR A 385     -38.560 -10.561  61.433  1.00 94.47           C
ATOM   3012  OH  TYR A 385     -38.987 -10.869  62.710  1.00 96.31           O
ATOM   3013  N   ARG A 386     -35.874  -8.050  55.089  1.00 81.61           N
ATOM   3014  CA  ARG A 386     -35.100  -8.023  53.850  1.00 82.69           C
ATOM   3015  C   ARG A 386     -33.737  -7.348  54.048  1.00 81.99           C
ATOM   3016  O   ARG A 386     -32.693  -7.915  53.703  1.00 78.56           O
ATOM   3017  CB  ARG A 386     -35.911  -7.324  52.758  1.00 72.71           C
ATOM   3018  CG  ARG A 386     -35.234  -7.195  51.410  1.00 63.80           C
ATOM   3019  CD  ARG A 386     -35.926  -6.115  50.573  1.00 69.06           C
ATOM   3020  NE  ARG A 386     -35.333  -4.789  50.779  1.00 73.01           N
ATOM   3021  CZ  ARG A 386     -35.902  -3.642  50.411  1.00 78.93           C
ATOM   3022  NH1 ARG A 386     -37.097  -3.649  49.830  1.00 79.98           N
ATOM   3023  NH2 ARG A 386     -35.287  -2.482  50.636  1.00 76.23           N
ATOM   3024  N   GLU A 387     -33.717  -6.139  54.612  1.00 75.44           N
ATOM   3025  CA  GLU A 387     -32.423  -5.483  54.751  1.00 76.19           C
ATOM   3026  C   GLU A 387     -31.547  -6.218  55.749  1.00 74.13           C
ATOM   3027  O   GLU A 387     -30.321  -6.264  55.590  1.00 72.55           O
ATOM   3028  CB  GLU A 387     -32.595  -4.022  55.155  1.00 76.82           C
ATOM   3029  CG  GLU A 387     -33.850  -3.390  54.602  1.00 86.81           C
ATOM   3030  CD  GLU A 387     -33.741  -1.886  54.489  1.00 98.17           C
ATOM   3031  OE1 GLU A 387     -33.224  -1.405  53.449  1.00 94.14           O
ATOM   3032  OE2 GLU A 387     -34.156  -1.193  55.452  1.00100.11           O
ATOM   3033  N   ALA A 388     -32.157  -6.831  56.755  1.00 75.54           N
ATOM   3034  CA  ALA A 388     -31.366  -7.518  57.762  1.00 68.96           C
ATOM   3035  C   ALA A 388     -30.541  -8.641  57.146  1.00 69.36           C
ATOM   3036  O   ALA A 388     -29.369  -8.826  57.500  1.00 67.43           O
ATOM   3037  CB  ALA A 388     -32.280  -8.039  58.859  1.00 61.39           C
ATOM   3038  N   LEU A 389     -31.116  -9.378  56.192  1.00 72.28           N
ATOM   3039  CA  LEU A 389     -30.408 -10.537  55.650  1.00 71.49           C
ATOM   3040  C   LEU A 389     -29.312 -10.108  54.677  1.00 64.07           C
ATOM   3041  O   LEU A 389     -28.306 -10.811  54.523  1.00 56.94           O
ATOM   3042  CB  LEU A 389     -31.400 -11.492  54.982  1.00 59.81           C
ATOM   3043  CG  LEU A 389     -30.890 -12.799  54.375  1.00 65.24           C
ATOM   3044  CD1 LEU A 389     -29.927 -13.487  55.351  1.00 56.53           C
ATOM   3045  CD2 LEU A 389     -32.060 -13.702  54.025  1.00 78.29           C
ATOM   3046  N   GLY A 390     -29.475  -8.955  54.025  1.00 65.67           N
ATOM   3047  CA  GLY A 390     -28.365  -8.396  53.275  1.00 64.59           C
ATOM   3048  C   GLY A 390     -27.213  -7.993  54.179  1.00 65.82           C
ATOM   3049  O   GLY A 390     -26.043  -8.273  53.883  1.00 58.45           O
ATOM   3050  N   ASP A 391     -27.525  -7.354  55.309  1.00 65.71           N
ATOM   3051  CA  ASP A 391     -26.455  -6.884  56.178  1.00 66.80           C
ATOM   3052  C   ASP A 391     -25.668  -8.053  56.764  1.00 68.41           C
ATOM   3053  O   ASP A 391     -24.428  -8.004  56.809  1.00 64.02           O
```

ATOM	3054	CB	ASP	A	391	-27.032	-5.981	57.261	1.00 63.99	C
ATOM	3055	CG	ASP	A	391	-27.558	-4.667	56.695	1.00 77.53	C
ATOM	3056	OD1	ASP	A	391	-26.883	-4.054	55.829	1.00 82.13	O
ATOM	3057	OD2	ASP	A	391	-28.677	-4.268	57.079	1.00 82.07	O
ATOM	3058	N	VAL	A	392	-26.355	-9.135	57.169	1.00 59.66	N
ATOM	3059	CA	VAL	A	392	-25.634	-10.265	57.759	1.00 55.78	C
ATOM	3060	C	VAL	A	392	-24.625	-10.827	56.768	1.00 61.52	C
ATOM	3061	O	VAL	A	392	-23.451	-11.035	57.105	1.00 58.23	O
ATOM	3062	CB	VAL	A	392	-26.599	-11.353	58.267	1.00 59.91	C
ATOM	3063	CG1	VAL	A	392	-27.418	-10.851	59.471	1.00 65.92	C
ATOM	3064	CG2	VAL	A	392	-27.504	-11.811	57.177	1.00 54.00	C
ATOM	3065	N	VAL	A	393	-25.056	-11.031	55.517	1.00 61.75	N
ATOM	3066	CA	VAL	A	393	-24.173	-11.585	54.497	1.00 50.58	C
ATOM	3067	C	VAL	A	393	-23.047	-10.613	54.175	1.00 60.31	C
ATOM	3068	O	VAL	A	393	-21.883	-11.017	54.044	1.00 54.36	O
ATOM	3069	CB	VAL	A	393	-24.991	-11.952	53.253	1.00 55.33	C
ATOM	3070	CG1	VAL	A	393	-24.098	-12.555	52.199	1.00 66.77	C
ATOM	3071	CG2	VAL	A	393	-26.066	-12.949	53.633	1.00 59.99	C
ATOM	3072	N	GLY	A	394	-23.370	-9.318	54.066	1.00 66.21	N
ATOM	3073	CA	GLY	A	394	-22.350	-8.330	53.742	1.00 62.24	C
ATOM	3074	C	GLY	A	394	-21.338	-8.149	54.862	1.00 65.62	C
ATOM	3075	O	GLY	A	394	-20.120	-8.142	54.621	1.00 56.61	O
ATOM	3076	N	ASP	A	395	-21.826	-8.011	56.109	1.00 60.70	N
ATOM	3077	CA	ASP	A	395	-20.917	-7.755	57.226	1.00 60.72	C
ATOM	3078	C	ASP	A	395	-20.058	-8.972	57.515	1.00 62.89	C
ATOM	3079	O	ASP	A	395	-18.840	-8.846	57.729	1.00 61.26	O
ATOM	3080	CB	ASP	A	395	-21.685	-7.345	58.485	1.00 63.34	C
ATOM	3081	CG	ASP	A	395	-22.368	-5.984	58.351	1.00 79.73	C
ATOM	3082	OD1	ASP	A	395	-23.376	-5.785	59.072	1.00 74.01	O
ATOM	3083	OD2	ASP	A	395	-21.911	-5.127	57.533	1.00 71.30	O
ATOM	3084	N	TYR	A	396	-20.675	-10.158	57.514	1.00 55.61	N
ATOM	3085	CA	TYR	A	396	-19.927	-11.384	57.757	1.00 59.59	C
ATOM	3086	C	TYR	A	396	-18.848	-11.573	56.699	1.00 61.54	C
ATOM	3087	O	TYR	A	396	-17.666	-11.763	57.024	1.00 60.65	O
ATOM	3088	CB	TYR	A	396	-20.884	-12.580	57.776	1.00 62.34	C
ATOM	3089	CG	TYR	A	396	-20.230	-13.917	58.051	1.00 62.96	C
ATOM	3090	CD1	TYR	A	396	-19.433	-14.089	59.175	1.00 55.66	C
ATOM	3091	CD2	TYR	A	396	-20.440	-15.018	57.213	1.00 57.64	C
ATOM	3092	CE1	TYR	A	396	-18.839	-15.294	59.449	1.00 61.45	C
ATOM	3093	CE2	TYR	A	396	-19.845	-16.248	57.481	1.00 50.95	C
ATOM	3094	CZ	TYR	A	396	-19.041	-16.374	58.606	1.00 63.16	C
ATOM	3095	OH	TYR	A	396	-18.427	-17.563	58.939	1.00 55.39	O
ATOM	3096	N	ASN	A	397	-19.230	-11.452	55.422	1.00 57.64	N
ATOM	3097	CA	ASN	A	397	-18.378	-11.896	54.324	1.00 62.84	C

ATOM	3098	C	ASN	A	397	-17.378	-10.853	53.848	1.00	61.93	C
ATOM	3099	O	ASN	A	397	-16.354	-11.231	53.257	1.00	56.38	O
ATOM	3100	CB	ASN	A	397	-19.228	-12.334	53.131	1.00	58.55	C
ATOM	3101	CG	ASN	A	397	-20.098	-13.551	53.448	1.00	63.74	C
ATOM	3102	OD1	ASN	A	397	-19.608	-14.689	53.514	1.00	63.54	O
ATOM	3103	ND2	ASN	A	397	-21.399	-13.316	53.637	1.00	52.70	N
ATOM	3104	N	PHE	A	398	-17.633	-9.561	54.072	1.00	59.19	N
ATOM	3105	CA	PHE	A	398	-16.787	-8.564	53.433	1.00	56.58	C
ATOM	3106	C	PHE	A	398	-16.398	-7.439	54.362	1.00	61.70	C
ATOM	3107	O	PHE	A	398	-15.207	-7.145	54.520	1.00	62.53	O
ATOM	3108	CB	PHE	A	398	-17.484	-7.958	52.220	1.00	57.26	C
ATOM	3109	CG	PHE	A	398	-17.862	-8.959	51.177	1.00	59.67	C
ATOM	3110	CD1	PHE	A	398	-19.150	-9.471	51.134	1.00	63.98	C
ATOM	3111	CD2	PHE	A	398	-16.936	-9.390	50.240	1.00	55.78	C
ATOM	3112	CE1	PHE	A	398	-19.512	-10.391	50.175	1.00	60.91	C
ATOM	3113	CE2	PHE	A	398	-17.280	-10.320	49.287	1.00	48.44	C
ATOM	3114	CZ	PHE	A	398	-18.574	-10.820	49.246	1.00	54.50	C
ATOM	3115	N	ILE	A	399	-17.399	-6.778	54.945	1.00	69.00	N
ATOM	3116	CA	ILE	A	399	-17.144	-5.552	55.693	1.00	61.98	C
ATOM	3117	C	ILE	A	399	-16.265	-5.846	56.899	1.00	64.31	C
ATOM	3118	O	ILE	A	399	-15.115	-5.393	56.964	1.00	68.67	O
ATOM	3119	CB	ILE	A	399	-18.465	-4.876	56.091	1.00	51.82	C
ATOM	3120	CG1	ILE	A	399	-19.113	-4.262	54.852	1.00	53.23	C
ATOM	3121	CG2	ILE	A	399	-18.220	-3.816	57.139	1.00	55.30	C
ATOM	3122	CD1	ILE	A	399	-20.555	-3.875	55.071	1.00	64.42	C
ATOM	3123	N	CYS	A	400	-16.770	-6.636	57.851	1.00	56.86	N
ATOM	3124	CA	CYS	A	400	-16.000	-6.841	59.076	1.00	59.87	C
ATOM	3125	C	CYS	A	400	-14.619	-7.411	58.810	1.00	58.54	C
ATOM	3126	O	CYS	A	400	-13.673	-7.025	59.522	1.00	59.87	O
ATOM	3127	CB	CYS	A	400	-16.780	-7.714	60.065	1.00	48.43	C
ATOM	3128	SG	CYS	A	400	-18.297	-6.892	60.532	1.00	70.74	S
ATOM	3129	N	PRO	A	401	-14.414	-8.288	57.832	1.00	62.83	N
ATOM	3130	CA	PRO	A	401	-13.030	-8.706	57.560	1.00	64.42	C
ATOM	3131	C	PRO	A	401	-12.152	-7.545	57.118	1.00	64.57	C
ATOM	3132	O	PRO	A	401	-11.047	-7.386	57.654	1.00	64.84	O
ATOM	3133	CB	PRO	A	401	-13.197	-9.790	56.484	1.00	64.35	C
ATOM	3134	CG	PRO	A	401	-14.597	-10.340	56.716	1.00	63.46	C
ATOM	3135	CD	PRO	A	401	-15.408	-9.152	57.158	1.00	62.22	C
ATOM	3136	N	ALA	A	402	-12.620	-6.710	56.182	1.00	58.10	N
ATOM	3137	CA	ALA	A	402	-11.817	-5.583	55.708	1.00	62.69	C
ATOM	3138	C	ALA	A	402	-11.356	-4.708	56.870	1.00	74.19	C
ATOM	3139	O	ALA	A	402	-10.153	-4.482	57.060	1.00	72.93	O
ATOM	3140	CB	ALA	A	402	-12.614	-4.751	54.700	1.00	62.50	C
ATOM	3141	N	LEU	A	403	-12.317	-4.211	57.660	1.00	73.95	N

```
ATOM   3142  CA  LEU A 403     -12.013  -3.487  58.892  1.00 65.78           C
ATOM   3143  C   LEU A 403     -10.995  -4.248  59.728  1.00 71.47           C
ATOM   3144  O   LEU A 403      -9.905  -3.742  60.014  1.00 72.06           O
ATOM   3145  CB  LEU A 403     -13.300  -3.246  59.692  1.00 56.81           C
ATOM   3146  CG  LEU A 403     -14.291  -2.303  59.008  1.00 58.89           C
ATOM   3147  CD1 LEU A 403     -15.709  -2.531  59.490  1.00 55.83           C
ATOM   3148  CD2 LEU A 403     -13.875  -0.845  59.204  1.00 61.34           C
ATOM   3149  N   GLU A 404     -11.321  -5.492  60.097  1.00 67.92           N
ATOM   3150  CA  GLU A 404     -10.421  -6.250  60.955  1.00 63.54           C
ATOM   3151  C   GLU A 404      -9.116  -6.587  60.237  1.00 63.78           C
ATOM   3152  O   GLU A 404      -8.088  -6.793  60.896  1.00 66.97           O
ATOM   3153  CB  GLU A 404     -11.114  -7.506  61.479  1.00 60.47           C
ATOM   3154  CG  GLU A 404     -10.703  -8.782  60.779  1.00 77.91           C
ATOM   3155  CD  GLU A 404     -11.177 -10.067  61.489  1.00 94.19           C
ATOM   3156  OE1 GLU A 404     -11.582 -11.059  60.807  1.00 87.29           O
ATOM   3157  OE2 GLU A 404     -11.132 -10.086  62.748  1.00101.96           O
ATOM   3158  N   PHE A 405      -9.119  -6.617  58.898  1.00 72.03           N
ATOM   3159  CA  PHE A 405      -7.851  -6.707  58.178  1.00 76.13           C
ATOM   3160  C   PHE A 405      -7.044  -5.449  58.427  1.00 70.50           C
ATOM   3161  O   PHE A 405      -6.007  -5.492  59.098  1.00 70.83           O
ATOM   3162  CB  PHE A 405      -8.029  -6.891  56.666  1.00 71.48           C
ATOM   3163  CG  PHE A 405      -6.726  -6.773  55.882  1.00 69.12           C
ATOM   3164  CD1 PHE A 405      -5.823  -7.834  55.826  1.00 68.17           C
ATOM   3165  CD2 PHE A 405      -6.407  -5.603  55.204  1.00 70.04           C
ATOM   3166  CE1 PHE A 405      -4.623  -7.721  55.118  1.00 71.06           C
ATOM   3167  CE2 PHE A 405      -5.212  -5.488  54.508  1.00 73.36           C
ATOM   3168  CZ  PHE A 405      -4.327  -6.547  54.442  1.00 69.67           C
ATOM   3169  N   THR A 406      -7.523  -4.327  57.884  1.00 71.19           N
ATOM   3170  CA  THR A 406      -6.732  -3.105  57.927  1.00 76.34           C
ATOM   3171  C   THR A 406      -6.400  -2.705  59.350  1.00 73.79           C
ATOM   3172  O   THR A 406      -5.334  -2.126  59.591  1.00 75.79           O
ATOM   3173  CB  THR A 406      -7.458  -1.959  57.240  1.00 75.12           C
ATOM   3174  OG1 THR A 406      -6.786  -0.768  57.505  1.00 80.96           O
ATOM   3175  CG2 THR A 406      -8.835  -1.837  57.733  1.00 75.22           C
ATOM   3176  N   LYS A 407      -7.295  -3.006  60.295  1.00 75.24           N
ATOM   3177  CA  LYS A 407      -7.063  -2.678  61.697  1.00 74.43           C
ATOM   3178  C   LYS A 407      -5.713  -3.189  62.128  1.00 74.34           C
ATOM   3179  O   LYS A 407      -4.868  -2.451  62.639  1.00 84.21           O
ATOM   3180  CB  LYS A 407      -8.149  -3.296  62.566  1.00 70.72           C
ATOM   3181  CG  LYS A 407      -8.272  -2.657  63.909  1.00 70.85           C
ATOM   3182  CD  LYS A 407      -9.503  -3.178  64.602  1.00 82.24           C
ATOM   3183  CE  LYS A 407     -10.736  -2.962  63.709  1.00 79.29           C
ATOM   3184  NZ  LYS A 407     -12.010  -3.422  64.352  1.00 84.16           N
ATOM   3185  N   LYS A 408      -5.469  -4.444  61.856  1.00 68.54           N
```

```
ATOM   3186  CA  LYS A 408      -4.247  -5.004  62.366  1.00 78.52           C
ATOM   3187  C   LYS A 408      -3.071  -4.823  61.416  1.00 78.39           C
ATOM   3188  O   LYS A 408      -1.915  -4.934  61.836  1.00 93.21           O
ATOM   3189  CB  LYS A 408      -4.544  -6.428  62.686  1.00 83.35           C
ATOM   3190  CG  LYS A 408      -3.456  -7.276  62.676  1.00 86.29           C
ATOM   3191  CD  LYS A 408      -3.781  -7.905  61.375  1.00 96.19           C
ATOM   3192  CE  LYS A 408      -2.883  -9.043  61.301  1.00102.58           C
ATOM   3193  NZ  LYS A 408      -2.757  -9.690  59.948  1.00 97.74           N
ATOM   3194  N   PHE A 409      -3.320  -4.459  60.172  1.00 73.34           N
ATOM   3195  CA  PHE A 409      -2.191  -4.096  59.329  1.00 83.90           C
ATOM   3196  C   PHE A 409      -1.571  -2.768  59.762  1.00 77.74           C
ATOM   3197  O   PHE A 409      -0.359  -2.574  59.606  1.00 78.85           O
ATOM   3198  CB  PHE A 409      -2.632  -4.050  57.861  1.00 86.40           C
ATOM   3199  CG  PHE A 409      -1.500  -3.917  56.901  1.00 72.65           C
ATOM   3200  CD1 PHE A 409      -0.951  -5.042  56.313  1.00 77.53           C
ATOM   3201  CD2 PHE A 409      -0.981  -2.677  56.591  1.00 78.32           C
ATOM   3202  CE1 PHE A 409       0.096  -4.931  55.429  1.00 78.28           C
ATOM   3203  CE2 PHE A 409       0.071  -2.554  55.722  1.00 80.71           C
ATOM   3204  CZ  PHE A 409       0.614  -3.683  55.134  1.00 82.76           C
ATOM   3205  N   SER A 410      -2.374  -1.860  60.326  1.00 80.01           N
ATOM   3206  CA  SER A 410      -1.851  -0.569  60.774  1.00 85.44           C
ATOM   3207  C   SER A 410      -1.081  -0.692  62.098  1.00 88.00           C
ATOM   3208  O   SER A 410      -0.059  -0.014  62.294  1.00 79.29           O
ATOM   3209  CB  SER A 410      -3.001   0.443  60.875  1.00 75.00           C
ATOM   3210  OG  SER A 410      -4.010   0.026  61.794  1.00 70.70           O
ATOM   3211  N   GLU A 411      -1.511  -1.590  62.996  1.00 83.71           N
ATOM   3212  CA  GLU A 411      -0.913  -1.696  64.330  1.00 99.28           C
ATOM   3213  C   GLU A 411       0.563  -2.063  64.294  1.00101.61           C
ATOM   3214  O   GLU A 411       1.180  -2.274  65.347  1.00103.99           O
ATOM   3215  CB  GLU A 411      -1.638  -2.726  65.204  1.00 89.27           C
ATOM   3216  CG  GLU A 411      -3.116  -2.859  64.934  1.00 91.90           C
ATOM   3217  CD  GLU A 411      -3.910  -3.288  66.154  1.00 94.58           C
ATOM   3218  OE1 GLU A 411      -5.048  -2.800  66.349  1.00 87.41           O
ATOM   3219  OE2 GLU A 411      -3.385  -4.105  66.932  1.00109.91           O
ATOM   3220  N   TRP A 412       1.130  -2.158  63.092  1.00 88.42           N
ATOM   3221  CA  TRP A 412       2.553  -2.390  62.896  1.00 91.18           C
ATOM   3222  C   TRP A 412       3.210  -1.219  62.182  1.00 96.51           C
ATOM   3223  O   TRP A 412       4.254  -1.386  61.539  1.00104.21           O
ATOM   3224  CB  TRP A 412       2.794  -3.700  62.141  1.00 94.09           C
ATOM   3225  CG  TRP A 412       2.180  -4.871  62.849  1.00106.58           C
ATOM   3226  CD1 TRP A 412       0.850  -5.081  63.120  1.00105.43           C
ATOM   3227  CD2 TRP A 412       2.884  -5.932  63.492  1.00105.49           C
ATOM   3228  NE1 TRP A 412       0.687  -6.244  63.835  1.00106.53           N
ATOM   3229  CE2 TRP A 412       1.923  -6.779  64.083  1.00104.14           C
```

ATOM	3230	CE3	TRP	A	412	4.237	-6.264	63.602	1.00102.61	C
ATOM	3231	CZ2	TRP	A	412	2.273	-7.925	64.769	1.00108.23	C
ATOM	3232	CZ3	TRP	A	412	4.580	-7.397	64.281	1.00102.77	C
ATOM	3233	CH2	TRP	A	412	3.606	-8.207	64.872	1.00109.28	C
ATOM	3234	N	GLY	A	413	2.601	-0.041	62.271	1.00 87.99	N
ATOM	3235	CA	GLY	A	413	3.257	1.188	61.895	1.00 93.49	C
ATOM	3236	C	GLY	A	413	2.960	1.703	60.510	1.00 91.17	C
ATOM	3237	O	GLY	A	413	3.474	2.769	60.145	1.00 86.88	O
ATOM	3238	N	ASN	A	414	2.141	1.011	59.731	1.00 92.27	N
ATOM	3239	CA	ASN	A	414	1.965	1.421	58.347	1.00 92.35	C
ATOM	3240	C	ASN	A	414	0.727	2.288	58.157	1.00 88.34	C
ATOM	3241	O	ASN	A	414	-0.255	2.200	58.905	1.00 89.80	O
ATOM	3242	CB	ASN	A	414	1.910	0.201	57.437	1.00 95.26	C
ATOM	3243	CG	ASN	A	414	3.265	-0.445	57.281	1.00 99.51	C
ATOM	3244	OD1	ASN	A	414	3.491	-1.570	57.768	1.00 90.68	O
ATOM	3245	ND2	ASN	A	414	4.200	0.279	56.632	1.00 81.32	N
ATOM	3246	N	ASN	A	415	0.808	3.146	57.144	1.00 87.70	N
ATOM	3247	CA	ASN	A	415	-0.270	4.059	56.791	1.00 95.57	C
ATOM	3248	C	ASN	A	415	-1.393	3.310	56.098	1.00 83.73	C
ATOM	3249	O	ASN	A	415	-1.194	2.756	55.017	1.00 84.38	O
ATOM	3250	CB	ASN	A	415	0.258	5.157	55.872	1.00 93.34	C
ATOM	3251	CG	ASN	A	415	0.775	6.348	56.632	1.00 84.96	C
ATOM	3252	OD1	ASN	A	415	0.838	6.334	57.870	1.00 78.34	O
ATOM	3253	ND2	ASN	A	415	1.165	7.389	55.895	1.00 70.57	N
ATOM	3254	N	ALA	A	416	-2.576	3.319	56.692	1.00 84.90	N
ATOM	3255	CA	ALA	A	416	-3.732	2.701	56.074	1.00 82.62	C
ATOM	3256	C	ALA	A	416	-4.847	3.729	55.961	1.00 79.70	C
ATOM	3257	O	ALA	A	416	-5.041	4.543	56.867	1.00 83.66	O
ATOM	3258	CB	ALA	A	416	-4.187	1.475	56.871	1.00 68.69	C
ATOM	3259	N	PHE	A	417	-5.563	3.693	54.836	1.00 71.27	N
ATOM	3260	CA	PHE	A	417	-6.693	4.570	54.561	1.00 73.79	C
ATOM	3261	C	PHE	A	417	-7.896	3.722	54.174	1.00 77.65	C
ATOM	3262	O	PHE	A	417	-7.781	2.869	53.284	1.00 78.81	O
ATOM	3263	CB	PHE	A	417	-6.362	5.540	53.431	1.00 81.98	C
ATOM	3264	CG	PHE	A	417	-5.031	6.209	53.578	1.00 89.16	C
ATOM	3265	CD1	PHE	A	417	-4.929	7.419	54.246	1.00 83.07	C
ATOM	3266	CD2	PHE	A	417	-3.880	5.638	53.049	1.00 87.05	C
ATOM	3267	CE1	PHE	A	417	-3.705	8.048	54.387	1.00 88.01	C
ATOM	3268	CE2	PHE	A	417	-2.645	6.266	53.188	1.00 85.67	C
ATOM	3269	CZ	PHE	A	417	-2.557	7.470	53.860	1.00 89.45	C
ATOM	3270	N	PHE	A	418	-9.046	3.964	54.819	1.00 71.20	N
ATOM	3271	CA	PHE	A	418	-10.250	3.156	54.627	1.00 53.12	C
ATOM	3272	C	PHE	A	418	-11.379	3.978	54.024	1.00 56.57	C
ATOM	3273	O	PHE	A	418	-11.630	5.106	54.463	1.00 74.50	O

ATOM	3274	CB	PHE	A	418	-10.723	2.552	55.941	1.00 56.70	C
ATOM	3275	CG	PHE	A	418	-11.600	1.350	55.761	1.00 64.95	C
ATOM	3276	CD1	PHE	A	418	-12.950	1.496	55.461	1.00 69.06	C
ATOM	3277	CD2	PHE	A	418	-11.085	0.073	55.905	1.00 59.93	C
ATOM	3278	CE1	PHE	A	418	-13.767	0.385	55.281	1.00 63.54	C
ATOM	3279	CE2	PHE	A	418	-11.895	-1.038	55.733	1.00 64.32	C
ATOM	3280	CZ	PHE	A	418	-13.239	-0.879	55.416	1.00 60.26	C
ATOM	3281	N	TYR	A	419	-12.072	3.411	53.034	1.00 57.27	N
ATOM	3282	CA	TYR	A	419	-13.147	4.112	52.341	1.00 62.96	C
ATOM	3283	C	TYR	A	419	-14.454	3.350	52.481	1.00 60.56	C
ATOM	3284	O	TYR	A	419	-14.479	2.154	52.778	1.00 64.89	O
ATOM	3285	CB	TYR	A	419	-12.839	4.343	50.839	1.00 64.17	C
ATOM	3286	CG	TYR	A	419	-12.958	3.112	49.939	1.00 68.23	C
ATOM	3287	CD1	TYR	A	419	-11.830	2.422	49.521	1.00 57.27	C
ATOM	3288	CD2	TYR	A	419	-14.204	2.661	49.495	1.00 63.31	C
ATOM	3289	CE1	TYR	A	419	-11.945	1.308	48.718	1.00 72.24	C
ATOM	3290	CE2	TYR	A	419	-14.327	1.554	48.704	1.00 62.27	C
ATOM	3291	CZ	TYR	A	419	-13.202	0.870	48.306	1.00 74.16	C
ATOM	3292	OH	TYR	A	419	-13.334	-0.257	47.497	1.00 61.66	O
ATOM	3293	N	TYR	A	420	-15.549	4.061	52.230	1.00 52.42	N
ATOM	3294	CA	TYR	A	420	-16.885	3.480	52.291	1.00 62.05	C
ATOM	3295	C	TYR	A	420	-17.671	4.137	51.163	1.00 72.81	C
ATOM	3296	O	TYR	A	420	-17.962	5.339	51.229	1.00 69.64	O
ATOM	3297	CB	TYR	A	420	-17.494	3.694	53.708	1.00 51.85	C
ATOM	3298	CG	TYR	A	420	-18.843	3.026	54.044	1.00 63.93	C
ATOM	3299	CD1	TYR	A	420	-18.961	1.639	54.128	1.00 73.87	C
ATOM	3300	CD2	TYR	A	420	-19.960	3.786	54.426	1.00 65.69	C
ATOM	3301	CE1	TYR	A	420	-20.182	1.034	54.459	1.00 70.87	C
ATOM	3302	CE2	TYR	A	420	-21.192	3.195	54.705	1.00 65.24	C
ATOM	3303	CZ	TYR	A	420	-21.281	1.821	54.757	1.00 64.90	C
ATOM	3304	OH	TYR	A	420	-22.476	1.216	55.041	1.00 78.44	O
ATOM	3305	N	PHE	A	421	-18.001	3.358	50.119	1.00 69.81	N
ATOM	3306	CA	PHE	A	421	-18.430	3.912	48.828	1.00 69.00	C
ATOM	3307	C	PHE	A	421	-19.954	3.837	48.699	1.00 75.66	C
ATOM	3308	O	PHE	A	421	-20.497	2.747	48.480	1.00 80.33	O
ATOM	3309	CB	PHE	A	421	-17.731	3.151	47.702	1.00 64.84	C
ATOM	3310	CG	PHE	A	421	-18.257	3.466	46.347	1.00 66.92	C
ATOM	3311	CD1	PHE	A	421	-17.867	4.611	45.676	1.00 75.52	C
ATOM	3312	CD2	PHE	A	421	-19.195	2.646	45.772	1.00 58.46	C
ATOM	3313	CE1	PHE	A	421	-18.398	4.923	44.413	1.00 74.28	C
ATOM	3314	CE2	PHE	A	421	-19.728	2.935	44.537	1.00 77.31	C
ATOM	3315	CZ	PHE	A	421	-19.331	4.074	43.838	1.00 71.73	C
ATOM	3316	N	GLU	A	422	-20.663	4.976	48.862	1.00 71.52	N
ATOM	3317	CA	GLU	A	422	-22.118	4.929	49.023	1.00 66.53	C

ATOM	3318	C	GLU	A	422	-22.936	5.415	47.848	1.00	70.39	C
ATOM	3319	O	GLU	A	422	-24.157	5.522	47.994	1.00	60.16	O
ATOM	3320	CB	GLU	A	422	-22.579	5.703	50.253	1.00	74.38	C
ATOM	3321	CG	GLU	A	422	-21.790	6.909	50.628	1.00	85.84	C
ATOM	3322	CD	GLU	A	422	-22.091	7.339	52.053	1.00	95.59	C
ATOM	3323	OE1	GLU	A	422	-22.078	6.491	52.976	1.00	82.57	O
ATOM	3324	OE2	GLU	A	422	-22.436	8.527	52.240	1.00106.07		O
ATOM	3325	N	HIS	A	423	-22.345	5.689	46.694	1.00	72.28	N
ATOM	3326	CA	HIS	A	423	-23.187	6.043	45.565	1.00	72.16	C
ATOM	3327	C	HIS	A	423	-23.622	4.765	44.848	1.00	69.01	C
ATOM	3328	O	HIS	A	423	-22.790	3.905	44.541	1.00	70.29	O
ATOM	3329	CB	HIS	A	423	-22.488	6.997	44.592	1.00	74.36	C
ATOM	3330	CG	HIS	A	423	-23.336	7.353	43.401	1.00	70.90	C
ATOM	3331	ND1	HIS	A	423	-24.677	7.668	43.507	1.00	65.79	N
ATOM	3332	CD2	HIS	A	423	-23.043	7.431	42.081	1.00	71.63	C
ATOM	3333	CE1	HIS	A	423	-25.169	7.936	42.311	1.00	67.19	C
ATOM	3334	NE2	HIS	A	423	-24.200	7.793	41.426	1.00	79.19	N
ATOM	3335	N	ARG	A	424	-24.926	4.641	44.592	1.00	64.73	N
ATOM	3336	CA	ARG	A	424	-25.440	3.641	43.658	1.00	64.90	C
ATOM	3337	C	ARG	A	424	-25.538	4.248	42.255	1.00	76.43	C
ATOM	3338	O	ARG	A	424	-26.111	5.329	42.083	1.00	76.32	O
ATOM	3339	CB	ARG	A	424	-26.811	3.127	44.104	1.00	56.33	C
ATOM	3340	CG	ARG	A	424	-27.603	2.463	43.006	1.00	54.85	C
ATOM	3341	CD	ARG	A	424	-28.926	1.936	43.489	1.00	55.82	C
ATOM	3342	NE	ARG	A	424	-29.729	1.574	42.339	1.00	63.65	N
ATOM	3343	CZ	ARG	A	424	-30.008	0.324	42.000	1.00	74.03	C
ATOM	3344	NH1	ARG	A	424	-29.593	-0.680	42.754	1.00	73.07	N
ATOM	3345	NH2	ARG	A	424	-30.732	0.094	40.906	1.00	71.25	N
ATOM	3346	N	SER	A	425	-24.978	3.552	41.257	1.00	77.34	N
ATOM	3347	CA	SER	A	425	-24.982	4.049	39.877	1.00	68.78	C
ATOM	3348	C	SER	A	425	-26.387	4.396	39.397	1.00	69.81	C
ATOM	3349	O	SER	A	425	-27.346	3.654	39.629	1.00	67.81	O
ATOM	3350	CB	SER	A	425	-24.367	3.011	38.935	1.00	82.38	C
ATOM	3351	OG	SER	A	425	-24.390	3.451	37.583	1.00	83.24	O
ATOM	3352	N	SER	A	426	-26.502	5.534	38.710	1.00	84.40	N
ATOM	3353	CA	SER	A	426	-27.754	5.880	38.044	1.00	82.99	C
ATOM	3354	C	SER	A	426	-28.135	4.859	36.979	1.00	77.61	C
ATOM	3355	O	SER	A	426	-29.313	4.780	36.611	1.00	67.75	O
ATOM	3356	CB	SER	A	426	-27.647	7.283	37.434	1.00	69.12	C
ATOM	3357	OG	SER	A	426	-26.543	7.373	36.546	1.00	63.99	O
ATOM	3358	N	LYS	A	427	-27.167	4.061	36.509	1.00	76.83	N
ATOM	3359	CA	LYS	A	427	-27.353	3.082	35.445	1.00	76.44	C
ATOM	3360	C	LYS	A	427	-27.467	1.649	35.946	1.00	76.44	C
ATOM	3361	O	LYS	A	427	-27.517	0.728	35.122	1.00	81.95	O

ATOM	3362	CB	LYS	A	427	-26.189	3.139	34.450	1.00	67.17	C
ATOM	3363	CG	LYS	A	427	-26.113	4.340	33.578	1.00	66.29	C
ATOM	3364	CD	LYS	A	427	-24.746	4.364	32.914	1.00	78.14	C
ATOM	3365	CE	LYS	A	427	-24.568	3.266	31.871	1.00	71.25	C
ATOM	3366	NZ	LYS	A	427	-23.319	3.508	31.099	1.00	71.13	N
ATOM	3367	N	LEU	A	428	-27.485	1.428	37.251	1.00	68.07	N
ATOM	3368	CA	LEU	A	428	-27.444	0.063	37.767	1.00	70.12	C
ATOM	3369	C	LEU	A	428	-28.676	-0.730	37.330	1.00	59.50	C
ATOM	3370	O	LEU	A	428	-29.813	-0.304	37.589	1.00	56.10	O
ATOM	3371	CB	LEU	A	428	-27.337	0.076	39.293	1.00	68.91	C
ATOM	3372	CG	LEU	A	428	-27.027	-1.290	39.902	1.00	71.72	C
ATOM	3373	CD1	LEU	A	428	-25.745	-1.793	39.286	1.00	67.35	C
ATOM	3374	CD2	LEU	A	428	-26.882	-1.230	41.422	1.00	64.82	C
ATOM	3375	N	PRO	A	429	-28.502	-1.882	36.677	1.00	56.09	N
ATOM	3376	CA	PRO	A	429	-29.654	-2.616	36.143	1.00	63.74	C
ATOM	3377	C	PRO	A	429	-30.330	-3.509	37.154	1.00	69.11	C
ATOM	3378	O	PRO	A	429	-31.286	-4.208	36.793	1.00	68.88	O
ATOM	3379	CB	PRO	A	429	-29.037	-3.453	35.013	1.00	62.60	C
ATOM	3380	CG	PRO	A	429	-27.699	-3.759	35.504	1.00	64.08	C
ATOM	3381	CD	PRO	A	429	-27.244	-2.570	36.353	1.00	67.23	C
ATOM	3382	N	TRP	A	430	-29.847	-3.521	38.364	1.00	68.56	N
ATOM	3383	CA	TRP	A	430	-30.453	-4.288	39.427	1.00	53.33	C
ATOM	3384	C	TRP	A	430	-31.457	-3.423	40.169	1.00	60.78	C
ATOM	3385	O	TRP	A	430	-31.308	-2.200	40.215	1.00	76.55	O
ATOM	3386	CB	TRP	A	430	-29.389	-4.777	40.386	1.00	60.29	C
ATOM	3387	CG	TRP	A	430	-28.420	-5.750	39.796	1.00	60.16	C
ATOM	3388	CD1	TRP	A	430	-27.216	-5.463	39.211	1.00	60.13	C
ATOM	3389	CD2	TRP	A	430	-28.553	-7.179	39.763	1.00	58.96	C
ATOM	3390	NE1	TRP	A	430	-26.597	-6.627	38.800	1.00	58.59	N
ATOM	3391	CE2	TRP	A	430	-27.396	-7.692	39.129	1.00	62.10	C
ATOM	3392	CE3	TRP	A	430	-29.539	-8.073	40.200	1.00	58.73	C
ATOM	3393	CZ2	TRP	A	430	-27.197	-9.057	38.931	1.00	58.11	C
ATOM	3394	CZ3	TRP	A	430	-29.344	-9.432	40.004	1.00	55.84	C
ATOM	3395	CH2	TRP	A	430	-28.181	-9.910	39.374	1.00	66.31	C
ATOM	3396	N	PRO	A	431	-32.487	-4.043	40.742	1.00	65.02	N
ATOM	3397	CA	PRO	A	431	-33.492	-3.292	41.516	1.00	72.65	C
ATOM	3398	C	PRO	A	431	-32.922	-2.326	42.544	1.00	66.24	C
ATOM	3399	O	PRO	A	431	-31.817	-2.517	43.065	1.00	63.28	O
ATOM	3400	CB	PRO	A	431	-34.302	-4.397	42.212	1.00	75.34	C
ATOM	3401	CG	PRO	A	431	-34.025	-5.627	41.462	1.00	71.10	C
ATOM	3402	CD	PRO	A	431	-32.742	-5.488	40.728	1.00	68.02	C
ATOM	3403	N	GLU	A	432	-33.693	-1.274	42.832	1.00	69.39	N
ATOM	3404	CA	GLU	A	432	-33.303	-0.311	43.861	1.00	82.44	C
ATOM	3405	C	GLU	A	432	-33.100	-0.989	45.213	1.00	76.95	C

ATOM	3406	O	GLU	A	432	-32.166	-0.631	45.948	1.00	65.84	O
ATOM	3407	CB	GLU	A	432	-34.351	0.812	43.961	1.00	70.44	C
ATOM	3408	CG	GLU	A	432	-34.405	1.713	42.728	0.81	72.01	C
ATOM	3409	CD	GLU	A	432	-33.049	2.046	42.080	0.42	76.69	C
ATOM	3410	OE1	GLU	A	432	-32.050	2.363	42.786	0.39	73.31	O
ATOM	3411	OE2	GLU	A	432	-32.956	1.913	40.829	0.85	79.16	O
ATOM	3412	N	TRP	A	433	-33.926	-1.992	45.548	1.00	65.07	N
ATOM	3413	CA	TRP	A	433	-33.813	-2.555	46.886	1.00	73.12	C
ATOM	3414	C	TRP	A	433	-32.428	-3.128	47.152	1.00	73.93	C
ATOM	3415	O	TRP	A	433	-32.053	-3.286	48.320	1.00	79.96	O
ATOM	3416	CB	TRP	A	433	-34.895	-3.616	47.150	1.00	75.05	C
ATOM	3417	CG	TRP	A	433	-34.783	-4.947	46.435	1.00	86.85	C
ATOM	3418	CD1	TRP	A	433	-35.576	-5.402	45.411	1.00	91.95	C
ATOM	3419	CD2	TRP	A	433	-33.846	-6.008	46.711	1.00	83.36	C
ATOM	3420	NE1	TRP	A	433	-35.189	-6.674	45.036	1.00	78.04	N
ATOM	3421	CE2	TRP	A	433	-34.128	-7.062	45.810	1.00	70.51	C
ATOM	3422	CE3	TRP	A	433	-32.797	-6.165	47.628	1.00	82.07	C
ATOM	3423	CZ2	TRP	A	433	-33.400	-8.240	45.799	1.00	68.08	C
ATOM	3424	CZ3	TRP	A	433	-32.073	-7.332	47.613	1.00	80.92	C
ATOM	3425	CH2	TRP	A	433	-32.378	-8.359	46.703	1.00	83.04	C
ATOM	3426	N	MET	A	434	-31.653	-3.428	46.104	1.00	74.21	N
ATOM	3427	CA	MET	A	434	-30.308	-3.954	46.298	1.00	72.43	C
ATOM	3428	C	MET	A	434	-29.328	-2.882	46.735	1.00	68.20	C
ATOM	3429	O	MET	A	434	-28.300	-3.218	47.324	1.00	71.29	O
ATOM	3430	CB	MET	A	434	-29.783	-4.631	45.021	1.00	74.60	C
ATOM	3431	CG	MET	A	434	-30.504	-5.943	44.674	1.00	80.87	C
ATOM	3432	SD	MET	A	434	-29.743	-6.999	43.396	1.00	75.32	S
ATOM	3433	CE	MET	A	434	-30.965	-8.297	43.356	1.00	61.28	C
ATOM	3434	N	GLY	A	435	-29.612	-1.613	46.454	1.00	66.01	N
ATOM	3435	CA	GLY	A	435	-28.762	-0.551	46.972	1.00	73.31	C
ATOM	3436	C	GLY	A	435	-27.393	-0.534	46.322	1.00	58.91	C
ATOM	3437	O	GLY	A	435	-27.249	-0.778	45.120	1.00	62.16	O
ATOM	3438	N	VAL	A	436	-26.372	-0.237	47.126	1.00	59.93	N
ATOM	3439	CA	VAL	A	436	-25.025	0.041	46.607	1.00	66.08	C
ATOM	3440	C	VAL	A	436	-24.284	-1.292	46.566	1.00	66.19	C
ATOM	3441	O	VAL	A	436	-23.610	-1.695	47.514	1.00	66.31	O
ATOM	3442	CB	VAL	A	436	-24.296	1.094	47.437	1.00	66.54	C
ATOM	3443	CG1	VAL	A	436	-22.813	1.100	47.115	1.00	70.62	C
ATOM	3444	CG2	VAL	A	436	-24.870	2.439	47.146	1.00	73.55	C
ATOM	3445	N	MET	A	437	-24.389	-1.970	45.432	1.00	65.52	N
ATOM	3446	CA	MET	A	437	-24.080	-3.387	45.379	1.00	65.17	C
ATOM	3447	C	MET	A	437	-22.583	-3.652	45.391	1.00	66.04	C
ATOM	3448	O	MET	A	437	-21.769	-2.813	44.989	1.00	70.48	O
ATOM	3449	CB	MET	A	437	-24.697	-4.016	44.136	1.00	61.43	C

ATOM	3450	CG	MET	A	437	-26.142	-4.412	44.349	1.00	62.11	C
ATOM	3451	SD	MET	A	437	-26.827	-4.933	42.782	1.00	58.99	S
ATOM	3452	CE	MET	A	437	-25.960	-6.476	42.467	1.00	57.97	C
ATOM	3453	N	HIS	A	438	-22.233	-4.833	45.902	1.00	59.65	N
ATOM	3454	CA	HIS	A	438	-20.921	-5.425	45.684	1.00	64.61	C
ATOM	3455	C	HIS	A	438	-20.544	-5.345	44.202	1.00	64.95	C
ATOM	3456	O	HIS	A	438	-21.385	-5.553	43.320	1.00	62.23	O
ATOM	3457	CB	HIS	A	438	-20.979	-6.876	46.185	1.00	62.63	C
ATOM	3458	CG	HIS	A	438	-19.691	-7.638	46.073	1.00	63.09	C
ATOM	3459	ND1	HIS	A	438	-18.605	-7.397	46.892	1.00	63.31	N
ATOM	3460	CD2	HIS	A	438	-19.352	-8.708	45.314	1.00	51.86	C
ATOM	3461	CE1	HIS	A	438	-17.634	-8.244	46.593	1.00	52.51	C
ATOM	3462	NE2	HIS	A	438	-18.067	-9.055	45.651	1.00	45.81	N
ATOM	3463	N	GLY	A	439	-19.295	-4.968	43.925	1.00	61.73	N
ATOM	3464	CA	GLY	A	439	-18.802	-4.909	42.559	1.00	70.91	C
ATOM	3465	C	GLY	A	439	-19.032	-3.604	41.803	1.00	68.85	C
ATOM	3466	O	GLY	A	439	-18.315	-3.343	40.827	1.00	61.35	O
ATOM	3467	N	TYR	A	440	-19.993	-2.771	42.215	1.00	57.09	N
ATOM	3468	CA	TYR	A	440	-20.350	-1.578	41.459	1.00	60.83	C
ATOM	3469	C	TYR	A	440	-19.631	-0.325	41.937	1.00	64.95	C
ATOM	3470	O	TYR	A	440	-20.073	0.792	41.651	1.00	51.57	O
ATOM	3471	CB	TYR	A	440	-21.862	-1.409	41.464	1.00	63.16	C
ATOM	3472	CG	TYR	A	440	-22.415	-2.480	40.580	1.00	64.89	C
ATOM	3473	CD1	TYR	A	440	-22.785	-3.706	41.106	1.00	69.25	C
ATOM	3474	CD2	TYR	A	440	-22.441	-2.310	39.188	1.00	60.60	C
ATOM	3475	CE1	TYR	A	440	-23.248	-4.721	40.274	1.00	75.99	C
ATOM	3476	CE2	TYR	A	440	-22.896	-3.304	38.348	1.00	60.98	C
ATOM	3477	CZ	TYR	A	440	-23.299	-4.518	38.893	1.00	67.85	C
ATOM	3478	OH	TYR	A	440	-23.758	-5.536	38.080	1.00	53.04	O
ATOM	3479	N	GLU	A	441	-18.531	-0.507	42.667	1.00	76.13	N
ATOM	3480	CA	GLU	A	441	-17.508	0.522	42.826	1.00	70.04	C
ATOM	3481	C	GLU	A	441	-16.602	0.626	41.605	1.00	69.30	C
ATOM	3482	O	GLU	A	441	-16.093	1.708	41.306	1.00	75.01	O
ATOM	3483	CB	GLU	A	441	-16.700	0.193	44.070	1.00	68.91	C
ATOM	3484	CG	GLU	A	441	-16.346	-1.279	44.089	1.00	71.18	C
ATOM	3485	CD	GLU	A	441	-15.022	-1.614	44.827	1.00	86.33	C
ATOM	3486	OE1	GLU	A	441	-14.841	-1.287	46.023	1.00	96.19	O
ATOM	3487	OE2	GLU	A	441	-14.185	-2.290	44.240	1.00	71.92	O
ATOM	3488	N	ILE	A	442	-16.429	-0.471	40.860	1.00	68.65	N
ATOM	3489	CA	ILE	A	442	-15.284	-0.601	39.951	1.00	67.14	C
ATOM	3490	C	ILE	A	442	-15.364	0.329	38.753	1.00	68.14	C
ATOM	3491	O	ILE	A	442	-14.330	0.866	38.329	1.00	75.83	O
ATOM	3492	CB	ILE	A	442	-15.076	-2.064	39.494	1.00	75.67	C
ATOM	3493	CG1	ILE	A	442	-14.574	-2.942	40.644	1.00	80.40	C

ATOM	3494	CG2	ILE	A	442	-14.016	-2.162	38.417	1.00	71.87	C
ATOM	3495	CD1	ILE	A	442	-13.353	-2.310	41.269	1.00	92.76	C
ATOM	3496	N	GLU	A	443	-16.554	0.526	38.167	1.00	71.00	N
ATOM	3497	CA	GLU	A	443	-16.612	1.373	36.978	1.00	63.62	C
ATOM	3498	C	GLU	A	443	-16.187	2.794	37.299	1.00	69.66	C
ATOM	3499	O	GLU	A	443	-15.520	3.440	36.480	1.00	71.93	O
ATOM	3500	CB	GLU	A	443	-18.002	1.333	36.353	1.00	62.42	C
ATOM	3501	CG	GLU	A	443	-19.131	1.732	37.258	1.00	71.56	C
ATOM	3502	CD	GLU	A	443	-20.415	0.968	36.950	1.00	86.09	C
ATOM	3503	OE1	GLU	A	443	-20.601	-0.136	37.527	1.00	76.38	O
ATOM	3504	OE2	GLU	A	443	-21.217	1.462	36.124	1.00	89.05	O
ATOM	3505	N	PHE	A	444	-16.511	3.268	38.505	1.00	72.48	N
ATOM	3506	CA	PHE	A	444	-16.038	4.572	38.949	1.00	63.75	C
ATOM	3507	C	PHE	A	444	-14.520	4.592	39.087	1.00	66.13	C
ATOM	3508	O	PHE	A	444	-13.875	5.559	38.668	1.00	73.01	O
ATOM	3509	CB	PHE	A	444	-16.701	4.944	40.273	1.00	62.81	C
ATOM	3510	CG	PHE	A	444	-18.148	5.312	40.147	1.00	63.75	C
ATOM	3511	CD1	PHE	A	444	-19.122	4.339	40.049	1.00	66.30	C
ATOM	3512	CD2	PHE	A	444	-18.540	6.625	40.160	1.00	68.11	C
ATOM	3513	CE1	PHE	A	444	-20.460	4.673	39.935	1.00	62.01	C
ATOM	3514	CE2	PHE	A	444	-19.882	6.958	40.054	1.00	73.54	C
ATOM	3515	CZ	PHE	A	444	-20.838	5.978	39.943	1.00	65.03	C
ATOM	3516	N	VAL	A	445	-13.928	3.536	39.656	1.00	60.76	N
ATOM	3517	CA	VAL	A	445	-12.475	3.516	39.834	1.00	64.09	C
ATOM	3518	C	VAL	A	445	-11.767	3.570	38.485	1.00	66.14	C
ATOM	3519	O	VAL	A	445	-10.800	4.318	38.303	1.00	71.03	O
ATOM	3520	CB	VAL	A	445	-12.045	2.286	40.657	1.00	67.94	C
ATOM	3521	CG1	VAL	A	445	-10.560	2.023	40.474	1.00	68.17	C
ATOM	3522	CG2	VAL	A	445	-12.356	2.498	42.143	1.00	58.29	C
ATOM	3523	N	PHE	A	446	-12.243	2.792	37.516	1.00	74.92	N
ATOM	3524	CA	PHE	A	446	-11.665	2.787	36.181	1.00	67.45	C
ATOM	3525	C	PHE	A	446	-12.054	4.011	35.363	1.00	71.27	C
ATOM	3526	O	PHE	A	446	-11.468	4.238	34.294	1.00	79.01	O
ATOM	3527	CB	PHE	A	446	-12.061	1.498	35.450	1.00	66.42	C
ATOM	3528	CG	PHE	A	446	-11.157	0.343	35.768	1.00	65.66	C
ATOM	3529	CD1	PHE	A	446	-11.437	-0.506	36.826	1.00	69.75	C
ATOM	3530	CD2	PHE	A	446	-9.993	0.140	35.043	1.00	63.61	C
ATOM	3531	CE1	PHE	A	446	-10.577	-1.558	37.140	1.00	66.99	C
ATOM	3532	CE2	PHE	A	446	-9.132	-0.892	35.350	1.00	65.25	C
ATOM	3533	CZ	PHE	A	446	-9.419	-1.745	36.399	1.00	62.42	C
ATOM	3534	N	GLY	A	447	-13.013	4.801	35.834	1.00	64.27	N
ATOM	3535	CA	GLY	A	447	-13.333	6.057	35.190	1.00	73.52	C
ATOM	3536	C	GLY	A	447	-14.243	5.980	33.989	1.00	68.87	C
ATOM	3537	O	GLY	A	447	-14.146	6.847	33.109	1.00	69.74	O

```
ATOM   3538  N   LEU A 448     -15.127   4.979  33.926  1.00 71.61           N
ATOM   3539  CA  LEU A 448     -16.115   4.925  32.849  1.00 68.33           C
ATOM   3540  C   LEU A 448     -17.031   6.153  32.807  1.00 80.34           C
ATOM   3541  O   LEU A 448     -17.406   6.562  31.692  1.00 86.43           O
ATOM   3542  CB  LEU A 448     -16.942   3.626  32.940  1.00 63.04           C
ATOM   3543  CG  LEU A 448     -16.346   2.220  32.663  1.00 61.76           C
ATOM   3544  CD1 LEU A 448     -17.387   1.220  32.155  1.00 49.32           C
ATOM   3545  CD2 LEU A 448     -15.149   2.223  31.727  1.00 81.12           C
ATOM   3546  N   PRO A 449     -17.418   6.789  33.925  1.00 70.48           N
ATOM   3547  CA  PRO A 449     -18.289   7.971  33.806  1.00 73.71           C
ATOM   3548  C   PRO A 449     -17.582   9.251  33.333  1.00 70.98           C
ATOM   3549  O   PRO A 449     -18.271  10.153  32.831  1.00 62.65           O
ATOM   3550  CB  PRO A 449     -18.856   8.118  35.223  1.00 70.14           C
ATOM   3551  CG  PRO A 449     -18.689   6.795  35.827  1.00 61.47           C
ATOM   3552  CD  PRO A 449     -17.386   6.325  35.319  1.00 60.53           C
ATOM   3553  N   LEU A 450     -16.249   9.358  33.475  1.00 67.41           N
ATOM   3554  CA  LEU A 450     -15.481  10.438  32.845  1.00 70.32           C
ATOM   3555  C   LEU A 450     -15.804  10.588  31.365  1.00 82.37           C
ATOM   3556  O   LEU A 450     -15.665  11.676  30.796  1.00 82.72           O
ATOM   3557  CB  LEU A 450     -13.977  10.187  32.971  1.00 62.20           C
ATOM   3558  CG  LEU A 450     -13.230  10.540  34.260  1.00 69.16           C
ATOM   3559  CD1 LEU A 450     -13.992  10.102  35.468  1.00 69.56           C
ATOM   3560  CD2 LEU A 450     -11.822   9.969  34.267  1.00 75.67           C
ATOM   3561  N   GLU A 451     -16.196   9.491  30.741  1.00 90.28           N
ATOM   3562  CA  GLU A 451     -16.658   9.410  29.362  1.00 82.33           C
ATOM   3563  C   GLU A 451     -18.074  10.003  29.280  1.00 85.23           C
ATOM   3564  O   GLU A 451     -18.954   9.638  30.066  1.00 85.43           O
ATOM   3565  CB  GLU A 451     -16.581   7.921  29.000  1.00 86.58           C
ATOM   3566  CG  GLU A 451     -16.903   7.377  27.656  1.00104.11           C
ATOM   3567  CD  GLU A 451     -16.680   8.267  26.491  1.00104.83           C
ATOM   3568  OE1 GLU A 451     -17.491   9.131  26.227  1.00100.37           O
ATOM   3569  OE2 GLU A 451     -15.697   8.098  25.804  1.00102.29           O
ATOM   3570  N   ARG A 452     -18.310  10.966  28.387  1.00 85.03           N
ATOM   3571  CA  ARG A 452     -19.513  11.790  28.570  1.00 97.27           C
ATOM   3572  C   ARG A 452     -20.661  11.395  27.661  1.00 89.51           C
ATOM   3573  O   ARG A 452     -21.784  11.908  27.803  1.00 80.05           O
ATOM   3574  CB  ARG A 452     -19.198  13.278  28.376  1.00 89.27           C
ATOM   3575  CG  ARG A 452     -20.304  14.175  28.951  1.00101.01           C
ATOM   3576  CD  ARG A 452     -20.729  15.289  28.008  1.00109.34           C
ATOM   3577  NE  ARG A 452     -21.836  14.889  27.127  1.00104.41           N
ATOM   3578  CZ  ARG A 452     -23.128  15.099  27.373  1.00 94.97           C
ATOM   3579  NH1 ARG A 452     -23.501  15.692  28.504  1.00 96.32           N
ATOM   3580  NH2 ARG A 452     -24.038  14.694  26.489  1.00 99.15           N
ATOM   3581  N   ARG A 453     -20.406  10.475  26.781  1.00 92.64           N
```

```
ATOM   3582  CA  ARG A 453     -21.369  10.002  25.828  1.00 92.23           C
ATOM   3583  C   ARG A 453     -22.172   8.769  26.379  1.00 95.73           C
ATOM   3584  O   ARG A 453     -22.866   8.073  25.619  1.00 91.75           O
ATOM   3585  CB  ARG A 453     -20.554   9.714  24.567  1.00 87.43           C
ATOM   3586  CG  ARG A 453     -21.118  10.053  23.192  1.00101.99           C
ATOM   3587  CD  ARG A 453     -22.441   9.368  22.768  1.00118.53           C
ATOM   3588  NE  ARG A 453     -22.494   7.937  23.181  1.00129.58           N
ATOM   3589  CZ  ARG A 453     -23.472   7.047  22.950  1.00116.82           C
ATOM   3590  NH1 ARG A 453     -24.499   7.345  22.136  1.00110.06           N
ATOM   3591  NH2 ARG A 453     -23.355   5.833  23.469  1.00110.09           N
ATOM   3592  N   ASP A 454     -22.141   8.522  27.716  1.00100.43           N
ATOM   3593  CA  ASP A 454     -22.404   7.189  28.273  1.00 97.06           C
ATOM   3594  C   ASP A 454     -23.548   7.091  29.285  1.00 83.48           C
ATOM   3595  O   ASP A 454     -23.717   6.020  29.877  1.00 78.43           O
ATOM   3596  CB  ASP A 454     -21.139   6.617  28.929  1.00 90.53           C
ATOM   3597  CG  ASP A 454     -19.925   6.813  28.081  1.00 97.69           C
ATOM   3598  OD1 ASP A 454     -19.359   5.801  27.601  1.00108.97           O
ATOM   3599  OD2 ASP A 454     -19.582   7.992  27.824  1.00 99.64           O
ATOM   3600  N   ASN A 455     -24.325   8.151  29.519  1.00 77.48           N
ATOM   3601  CA  ASN A 455     -25.562   8.151  30.323  1.00 79.98           C
ATOM   3602  C   ASN A 455     -25.307   8.202  31.836  1.00 75.55           C
ATOM   3603  O   ASN A 455     -26.264   8.073  32.620  1.00 67.93           O
ATOM   3604  CB  ASN A 455     -26.412   6.926  29.982  1.00 30.00           C
ATOM   3605  CG  ASN A 455     -27.026   7.009  28.598  1.00 30.00           C
ATOM   3606  OD1 ASN A 455     -27.193   8.096  28.045  1.00 30.00           O
ATOM   3607  ND2 ASN A 455     -27.366   5.857  28.031  1.00 30.00           N
ATOM   3608  N   TYR A 456     -24.070   8.367  32.284  1.00 71.73           N
ATOM   3609  CA  TYR A 456     -23.881   8.701  33.681  1.00 72.72           C
ATOM   3610  C   TYR A 456     -24.313  10.138  33.930  1.00 78.39           C
ATOM   3611  O   TYR A 456     -24.310  10.982  33.031  1.00 82.46           O
ATOM   3612  CB  TYR A 456     -22.422   8.545  34.097  1.00 80.04           C
ATOM   3613  CG  TYR A 456     -21.954   7.129  34.107  1.00 76.04           C
ATOM   3614  CD1 TYR A 456     -21.283   6.603  33.020  1.00 82.90           C
ATOM   3615  CD2 TYR A 456     -22.183   6.307  35.203  1.00 83.70           C
ATOM   3616  CE1 TYR A 456     -20.845   5.286  33.007  1.00 91.81           C
ATOM   3617  CE2 TYR A 456     -21.751   4.976  35.208  1.00 87.11           C
ATOM   3618  CZ  TYR A 456     -21.081   4.471  34.099  1.00 89.50           C
ATOM   3619  OH  TYR A 456     -20.633   3.162  34.062  1.00 77.18           O
ATOM   3620  N   THR A 457     -24.660  10.419  35.176  1.00 72.24           N
ATOM   3621  CA  THR A 457     -25.010  11.777  35.518  1.00 62.32           C
ATOM   3622  C   THR A 457     -23.750  12.590  35.780  1.00 70.18           C
ATOM   3623  O   THR A 457     -22.698  12.054  36.161  1.00 58.39           O
ATOM   3624  CB  THR A 457     -25.918  11.810  36.738  1.00 70.06           C
ATOM   3625  OG1 THR A 457     -25.167  11.419  37.893  1.00 69.16           O
```

ATOM	3626	CG2	THR	A	457	-27.099	10.865	36.535	1.00	65.62	C
ATOM	3627	N	LYS	A	458	-23.890	13.910	35.561	1.00	76.17	N
ATOM	3628	CA	LYS	A	458	-22.800	14.871	35.735	1.00	73.70	C
ATOM	3629	C	LYS	A	458	-22.138	14.717	37.105	1.00	74.99	C
ATOM	3630	O	LYS	A	458	-20.902	14.747	37.218	1.00	68.56	O
ATOM	3631	CB	LYS	A	458	-23.347	16.301	35.514	1.00	59.76	C
ATOM	3632	CG	LYS	A	458	-22.319	17.419	35.501	1.00	54.14	C
ATOM	3633	CD	LYS	A	458	-21.225	17.179	34.484	1.00	59.96	C
ATOM	3634	CE	LYS	A	458	-20.041	18.104	34.739	1.00	71.32	C
ATOM	3635	NZ	LYS	A	458	-18.865	17.900	33.846	1.00	66.80	N
ATOM	3636	N	ALA	A	459	-22.948	14.507	38.151	1.00	73.19	N
ATOM	3637	CA	ALA	A	459	-22.399	14.283	39.484	1.00	74.19	C
ATOM	3638	C	ALA	A	459	-21.552	13.029	39.533	1.00	78.52	C
ATOM	3639	O	ALA	A	459	-20.603	12.953	40.325	1.00	83.96	O
ATOM	3640	CB	ALA	A	459	-23.519	14.182	40.521	1.00	77.34	C
ATOM	3641	N	GLU	A	460	-21.886	12.027	38.716	1.00	78.58	N
ATOM	3642	CA	GLU	A	460	-21.111	10.796	38.761	1.00	74.66	C
ATOM	3643	C	GLU	A	460	-19.783	10.965	38.051	1.00	74.36	C
ATOM	3644	O	GLU	A	460	-18.786	10.359	38.473	1.00	65.81	O
ATOM	3645	CB	GLU	A	460	-21.914	9.649	38.165	1.00	72.97	C
ATOM	3646	CG	GLU	A	460	-23.137	9.356	38.993	1.00	71.61	C
ATOM	3647	CD	GLU	A	460	-24.040	8.317	38.370	1.00	70.14	C
ATOM	3648	OE1	GLU	A	460	-23.965	8.119	37.136	1.00	70.45	O
ATOM	3649	OE2	GLU	A	460	-24.859	7.736	39.112	1.00	61.54	O
ATOM	3650	N	GLU	A	461	-19.747	11.807	37.006	1.00	73.60	N
ATOM	3651	CA	GLU	A	461	-18.471	12.136	36.381	1.00	76.72	C
ATOM	3652	C	GLU	A	461	-17.547	12.844	37.372	1.00	81.13	C
ATOM	3653	O	GLU	A	461	-16.334	12.587	37.399	1.00	73.53	O
ATOM	3654	CB	GLU	A	461	-18.677	12.987	35.123	1.00	57.29	C
ATOM	3655	CG	GLU	A	461	-17.341	13.481	34.551	1.00	65.79	C
ATOM	3656	CD	GLU	A	461	-17.459	14.485	33.401	1.00	83.85	C
ATOM	3657	OE1	GLU	A	461	-16.391	14.869	32.863	1.00	83.67	O
ATOM	3658	OE2	GLU	A	461	-18.594	14.874	33.016	1.00	78.23	O
ATOM	3659	N	ILE	A	462	-18.095	13.720	38.217	1.00	74.30	N
ATOM	3660	CA	ILE	A	462	-17.187	14.367	39.157	1.00	81.05	C
ATOM	3661	C	ILE	A	462	-16.837	13.415	40.305	1.00	82.73	C
ATOM	3662	O	ILE	A	462	-15.717	13.463	40.834	1.00	81.72	O
ATOM	3663	CB	ILE	A	462	-17.744	15.718	39.655	1.00	73.14	C
ATOM	3664	CG1	ILE	A	462	-16.697	16.436	40.506	1.00	65.26	C
ATOM	3665	CG2	ILE	A	462	-19.015	15.546	40.428	1.00	82.95	C
ATOM	3666	CD1	ILE	A	462	-15.386	16.670	39.789	1.00	62.10	C
ATOM	3667	N	LEU	A	463	-17.745	12.508	40.683	1.00	78.80	N
ATOM	3668	CA	LEU	A	463	-17.372	11.496	41.665	1.00	73.98	C
ATOM	3669	C	LEU	A	463	-16.256	10.617	41.117	1.00	74.95	C

```
ATOM   3670  O   LEU A 463     -15.269  10.332  41.809  1.00 76.81           O
ATOM   3671  CB  LEU A 463     -18.590  10.661  42.076  1.00 66.19           C
ATOM   3672  CG  LEU A 463     -18.369   9.544  43.125  1.00 70.49           C
ATOM   3673  CD1 LEU A 463     -17.452   9.953  44.274  1.00 56.32           C
ATOM   3674  CD2 LEU A 463     -19.699   9.058  43.693  1.00 64.71           C
ATOM   3675  N   SER A 464     -16.365  10.230  39.850  1.00 68.91           N
ATOM   3676  CA  SER A 464     -15.378   9.322  39.281  1.00 81.43           C
ATOM   3677  C   SER A 464     -14.022  10.014  39.114  1.00 76.14           C
ATOM   3678  O   SER A 464     -12.981   9.459  39.492  1.00 67.58           O
ATOM   3679  CB  SER A 464     -15.910   8.760  37.953  1.00 79.07           C
ATOM   3680  OG  SER A 464     -15.043   7.794  37.372  1.00 71.52           O
ATOM   3681  N   ARG A 465     -14.016  11.238  38.565  1.00 75.32           N
ATOM   3682  CA  ARG A 465     -12.757  11.963  38.396  1.00 75.71           C
ATOM   3683  C   ARG A 465     -12.034  12.070  39.726  1.00 76.26           C
ATOM   3684  O   ARG A 465     -10.817  11.862  39.806  1.00 67.93           O
ATOM   3685  CB  ARG A 465     -13.012  13.355  37.790  1.00 77.94           C
ATOM   3686  CG  ARG A 465     -11.745  14.187  37.414  1.00 80.31           C
ATOM   3687  CD  ARG A 465     -11.349  14.066  35.941  1.00 80.05           C
ATOM   3688  NE  ARG A 465     -12.445  14.402  35.026  1.00 88.17           N
ATOM   3689  CZ  ARG A 465     -12.314  14.551  33.707  1.00103.32           C
ATOM   3690  NH1 ARG A 465     -11.133  14.373  33.124  1.00 90.56           N
ATOM   3691  NH2 ARG A 465     -13.376  14.842  32.958  1.00 94.51           N
ATOM   3692  N   SER A 466     -12.791  12.337  40.794  1.00 88.03           N
ATOM   3693  CA  SER A 466     -12.221  12.425  42.134  1.00 92.03           C
ATOM   3694  C   SER A 466     -11.567  11.109  42.547  1.00 87.00           C
ATOM   3695  O   SER A 466     -10.373  11.067  42.864  1.00 86.53           O
ATOM   3696  CB  SER A 466     -13.309  12.823  43.131  1.00 83.90           C
ATOM   3697  OG  SER A 466     -14.503  12.107  42.877  1.00 93.38           O
ATOM   3698  N   ILE A 467     -12.341  10.018  42.550  1.00 77.91           N
ATOM   3699  CA  ILE A 467     -11.809   8.731  42.998  1.00 76.50           C
ATOM   3700  C   ILE A 467     -10.600   8.323  42.156  1.00 83.89           C
ATOM   3701  O   ILE A 467      -9.597   7.815  42.685  1.00 73.59           O
ATOM   3702  CB  ILE A 467     -12.923   7.667  42.968  1.00 76.34           C
ATOM   3703  CG1 ILE A 467     -14.038   8.036  43.959  1.00 70.18           C
ATOM   3704  CG2 ILE A 467     -12.354   6.269  43.221  1.00 71.60           C
ATOM   3705  CD1 ILE A 467     -15.228   7.054  44.016  1.00 54.78           C
ATOM   3706  N   VAL A 468     -10.668   8.552  40.837  1.00 77.77           N
ATOM   3707  CA  VAL A 468      -9.539   8.252  39.960  1.00 64.47           C
ATOM   3708  C   VAL A 468      -8.289   8.992  40.421  1.00 86.64           C
ATOM   3709  O   VAL A 468      -7.182   8.442  40.373  1.00 88.90           O
ATOM   3710  CB  VAL A 468      -9.882   8.593  38.497  1.00 72.13           C
ATOM   3711  CG1 VAL A 468      -8.635   8.544  37.631  1.00 71.80           C
ATOM   3712  CG2 VAL A 468     -10.938   7.649  37.951  1.00 69.83           C
ATOM   3713  N   LYS A 469      -8.437  10.248  40.876  1.00 83.93           N
```

```
ATOM   3714  CA  LYS A 469      -7.271  11.016  41.312  1.00 77.79           C
ATOM   3715  C   LYS A 469      -6.711  10.463  42.625  1.00 72.20           C
ATOM   3716  O   LYS A 469      -5.492  10.291  42.764  1.00 71.15           O
ATOM   3717  CB  LYS A 469      -7.627  12.511  41.431  1.00 91.50           C
ATOM   3718  CG  LYS A 469      -6.523  13.509  40.962  1.00 82.26           C
ATOM   3719  CD  LYS A 469      -7.021  14.969  40.880  1.00 98.96           C
ATOM   3720  CE  LYS A 469      -8.082  15.211  39.778  1.00100.20           C
ATOM   3721  NZ  LYS A 469      -8.975  16.407  40.017  1.00 84.45           N
ATOM   3722  N   ARG A 470      -7.587  10.157  43.595  1.00 73.76           N
ATOM   3723  CA  ARG A 470      -7.121   9.608  44.873  1.00 84.16           C
ATOM   3724  C   ARG A 470      -6.358   8.310  44.653  1.00 78.18           C
ATOM   3725  O   ARG A 470      -5.178   8.182  45.025  1.00 62.58           O
ATOM   3726  CB  ARG A 470      -8.302   9.349  45.819  1.00 83.37           C
ATOM   3727  CG  ARG A 470      -9.438  10.357  45.772  1.00 85.29           C
ATOM   3728  CD  ARG A 470     -10.019  10.578  47.172  1.00 75.97           C
ATOM   3729  NE  ARG A 470     -10.906  11.759  47.181  1.00 97.79           N
ATOM   3730  CZ  ARG A 470     -11.022  12.618  48.200  1.00 91.38           C
ATOM   3731  NH1 ARG A 470     -11.860  13.660  48.111  1.00 86.65           N
ATOM   3732  NH2 ARG A 470     -10.286  12.420  49.290  1.00 82.86           N
ATOM   3733  N   TRP A 471      -7.042   7.332  44.048  1.00 78.54           N
ATOM   3734  CA  TRP A 471      -6.425   6.076  43.631  1.00 73.30           C
ATOM   3735  C   TRP A 471      -5.112   6.290  42.906  1.00 74.45           C
ATOM   3736  O   TRP A 471      -4.126   5.583  43.166  1.00 73.81           O
ATOM   3737  CB  TRP A 471      -7.369   5.321  42.717  1.00 76.36           C
ATOM   3738  CG  TRP A 471      -8.117   4.292  43.381  1.00 79.97           C
ATOM   3739  CD1 TRP A 471      -8.087   2.956  43.129  1.00 69.55           C
ATOM   3740  CD2 TRP A 471      -9.065   4.496  44.413  1.00 73.51           C
ATOM   3741  NE1 TRP A 471      -8.967   2.313  43.953  1.00 82.09           N
ATOM   3742  CE2 TRP A 471      -9.579   3.240  44.757  1.00 75.82           C
ATOM   3743  CE3 TRP A 471      -9.531   5.627  45.084  1.00 84.32           C
ATOM   3744  CZ2 TRP A 471     -10.535   3.080  45.743  1.00 72.45           C
ATOM   3745  CZ3 TRP A 471     -10.470   5.470  46.051  1.00 86.89           C
ATOM   3746  CH2 TRP A 471     -10.968   4.206  46.378  1.00 80.70           C
ATOM   3747  N   ALA A 472      -5.112   7.199  41.926  1.00 79.08           N
ATOM   3748  CA  ALA A 472      -3.867   7.654  41.316  1.00 80.44           C
ATOM   3749  C   ALA A 472      -2.851   8.040  42.371  1.00 82.30           C
ATOM   3750  O   ALA A 472      -1.819   7.384  42.526  1.00 79.95           O
ATOM   3751  CB  ALA A 472      -4.131   8.860  40.415  1.00 82.92           C
ATOM   3752  N   ASN A 473      -3.125   9.124  43.094  1.00 77.41           N
ATOM   3753  CA  ASN A 473      -2.126   9.689  43.992  1.00 86.82           C
ATOM   3754  C   ASN A 473      -1.653   8.672  45.033  1.00 78.80           C
ATOM   3755  O   ASN A 473      -0.453   8.616  45.352  1.00 75.77           O
ATOM   3756  CB  ASN A 473      -2.691  10.950  44.655  1.00 89.91           C
ATOM   3757  CG  ASN A 473      -2.820  12.118  43.687  1.00 84.40           C
```

ATOM	3758	OD1	ASN	A	473	-1.983	12.301	42.796	1.00	88.89	O
ATOM	3759	ND2	ASN	A	473	-3.867	12.917	43.863	1.00	90.35	N
ATOM	3760	N	PHE	A	474	-2.562	7.835	45.543	1.00	69.98	N
ATOM	3761	CA	PHE	A	474	-2.108	6.763	46.425	1.00	81.62	C
ATOM	3762	C	PHE	A	474	-1.063	5.893	45.735	1.00	81.34	C
ATOM	3763	O	PHE	A	474	-0.146	5.386	46.393	1.00	76.78	O
ATOM	3764	CB	PHE	A	474	-3.271	5.892	46.923	1.00	72.59	C
ATOM	3765	CG	PHE	A	474	-2.826	4.756	47.828	1.00	72.84	C
ATOM	3766	CD1	PHE	A	474	-2.571	4.970	49.175	1.00	82.98	C
ATOM	3767	CD2	PHE	A	474	-2.626	3.477	47.325	1.00	81.26	C
ATOM	3768	CE1	PHE	A	474	-2.141	3.912	50.017	1.00	77.32	C
ATOM	3769	CE2	PHE	A	474	-2.195	2.429	48.158	1.00	82.10	C
ATOM	3770	CZ	PHE	A	474	-1.953	2.655	49.507	1.00	64.96	C
ATOM	3771	N	ALA	A	475	-1.169	5.722	44.409	1.00	71.20	N
ATOM	3772	CA	ALA	A	475	-0.197	4.896	43.697	1.00	75.91	C
ATOM	3773	C	ALA	A	475	1.198	5.545	43.668	1.00	89.46	C
ATOM	3774	O	ALA	A	475	2.173	4.933	44.125	1.00	81.63	O
ATOM	3775	CB	ALA	A	475	-0.706	4.582	42.289	1.00	83.35	C
ATOM	3776	N	LYS	A	476	1.326	6.781	43.130	1.00	91.69	N
ATOM	3777	CA	LYS	A	476	2.627	7.480	43.123	1.00	87.23	C
ATOM	3778	C	LYS	A	476	3.139	7.781	44.521	1.00	84.67	C
ATOM	3779	O	LYS	A	476	4.277	7.430	44.857	1.00	86.92	O
ATOM	3780	CB	LYS	A	476	2.609	8.820	42.355	1.00	89.55	C
ATOM	3781	CG	LYS	A	476	2.120	8.852	40.923	1.00	88.00	C
ATOM	3782	CD	LYS	A	476	0.599	8.709	40.906	1.00	89.69	C
ATOM	3783	CE	LYS	A	476	0.090	8.221	39.552	1.00106.45		C
ATOM	3784	NZ	LYS	A	476	-1.218	7.476	39.590	1.00107.95		N
ATOM	3785	N	TYR	A	477	2.372	8.544	45.298	1.00	75.11	N
ATOM	3786	CA	TYR	A	477	2.909	9.188	46.484	1.00	77.35	C
ATOM	3787	C	TYR	A	477	2.437	8.552	47.776	1.00	87.60	C
ATOM	3788	O	TYR	A	477	2.850	8.998	48.851	1.00	87.57	O
ATOM	3789	CB	TYR	A	477	2.567	10.689	46.500	1.00	83.30	C
ATOM	3790	CG	TYR	A	477	2.686	11.378	45.161	1.00	85.36	C
ATOM	3791	CD1	TYR	A	477	3.818	11.200	44.368	1.00	91.11	C
ATOM	3792	CD2	TYR	A	477	1.694	12.236	44.706	1.00	75.50	C
ATOM	3793	CE1	TYR	A	477	3.945	11.823	43.131	1.00	89.87	C
ATOM	3794	CE2	TYR	A	477	1.816	12.875	43.472	1.00	82.78	C
ATOM	3795	CZ	TYR	A	477	2.944	12.663	42.688	1.00	91.11	C
ATOM	3796	OH	TYR	A	477	3.073	13.288	41.457	1.00	94.21	O
ATOM	3797	N	GLY	A	478	1.591	7.528	47.712	1.00	89.05	N
ATOM	3798	CA	GLY	A	478	1.146	6.898	48.937	1.00	88.35	C
ATOM	3799	C	GLY	A	478	0.260	7.742	49.835	1.00	81.38	C
ATOM	3800	O	GLY	A	478	0.203	7.488	51.042	1.00	77.61	O
ATOM	3801	N	ASN	A	479	-0.439	8.741	49.291	1.00	71.80	N

ATOM	3802	CA	ASN	A	479	-1.488	9.388	50.055	1.00	76.72	C
ATOM	3803	C	ASN	A	479	-2.683	9.603	49.132	1.00	78.24	C
ATOM	3804	O	ASN	A	479	-2.524	10.144	48.023	1.00	78.82	O
ATOM	3805	CB	ASN	A	479	-1.025	10.709	50.685	1.00	91.75	C
ATOM	3806	CG	ASN	A	479	-2.060	11.305	51.649	1.00	95.52	C
ATOM	3807	OD1	ASN	A	479	-3.161	11.702	51.244	1.00	91.76	O
ATOM	3808	ND2	ASN	A	479	-1.736	11.283	52.951	1.00	91.05	N
ATOM	3809	N	PRO	A	480	-3.873	9.190	49.543	1.00	74.17	N
ATOM	3810	CA	PRO	A	480	-5.041	9.296	48.659	1.00	73.80	C
ATOM	3811	C	PRO	A	480	-5.570	10.717	48.556	1.00	84.96	C
ATOM	3812	O	PRO	A	480	-6.779	10.960	48.663	1.00	84.14	O
ATOM	3813	CB	PRO	A	480	-6.067	8.372	49.324	1.00	87.31	C
ATOM	3814	CG	PRO	A	480	-5.368	7.711	50.502	1.00	84.44	C
ATOM	3815	CD	PRO	A	480	-4.188	8.547	50.829	1.00	82.91	C
ATOM	3816	N	ASN	A	481	-4.658	11.660	48.331	1.00	94.00	N
ATOM	3817	CA	ASN	A	481	-4.958	13.087	48.305	1.00	83.94	C
ATOM	3818	C	ASN	A	481	-5.473	13.525	46.934	1.00	95.53	C
ATOM	3819	O	ASN	A	481	-4.992	13.055	45.898	1.00100.58		O
ATOM	3820	CB	ASN	A	481	-3.727	13.905	48.697	1.00	30.00	C
ATOM	3821	CG	ASN	A	481	-3.391	13.785	50.171	1.00	30.00	C
ATOM	3822	OD1	ASN	A	481	-4.251	13.463	50.990	1.00	30.00	O
ATOM	3823	ND2	ASN	A	481	-2.135	14.043	50.515	1.00	30.00	N
ATOM	3824	N	GLU	A	482	-6.496	14.387	46.935	1.00	97.05	N
ATOM	3825	CA	GLU	A	482	-6.861	15.225	45.784	1.00106.27		C
ATOM	3826	C	GLU	A	482	-6.787	16.653	46.316	1.00118.02		C
ATOM	3827	O	GLU	A	482	-7.719	17.125	46.980	1.00121.42		O
ATOM	3828	CB	GLU	A	482	-8.251	14.901	45.213	1.00109.83		C
ATOM	3829	CG	GLU	A	482	-8.818	15.986	44.249	1.00114.95		C
ATOM	3830	CD	GLU	A	482	-10.230	15.672	43.712	1.00118.94		C
ATOM	3831	OE1	GLU	A	482	-11.125	15.350	44.530	1.00112.62		O
ATOM	3832	OE2	GLU	A	482	-10.438	15.757	42.473	1.00108.90		O
ATOM	3833	N	THR	A	483	-5.675	17.334	46.030	1.00124.64		N
ATOM	3834	CA	THR	A	483	-5.204	18.434	46.869	1.00132.70		C
ATOM	3835	C	THR	A	483	-5.615	19.837	46.413	1.00134.06		C
ATOM	3836	O	THR	A	483	-5.197	20.813	47.051	1.00115.77		O
ATOM	3837	CB	THR	A	483	-3.667	18.376	46.997	1.00119.56		C
ATOM	3838	OG1	THR	A	483	-3.059	18.326	45.697	1.00129.19		O
ATOM	3839	CG2	THR	A	483	-3.223	17.183	47.839	1.00	96.90	C
ATOM	3840	N	GLN	A	484	-6.428	19.997	45.365	1.00124.08		N
ATOM	3841	CA	GLN	A	484	-6.575	21.364	44.886	1.00130.41		C
ATOM	3842	C	GLN	A	484	-8.003	21.917	44.876	1.00134.36		C
ATOM	3843	O	GLN	A	484	-8.164	23.126	45.086	1.00128.97		O
ATOM	3844	CB	GLN	A	484	-5.926	21.507	43.491	1.00130.60		C
ATOM	3845	CG	GLN	A	484	-6.794	21.229	42.286	1.00125.57		C

```
ATOM   3846  CD  GLN A 484      -7.321  22.520  41.672  1.00122.05           C
ATOM   3847  OE1 GLN A 484      -6.578  23.494  41.506  1.00112.71           O
ATOM   3848  NE2 GLN A 484      -8.601  22.529  41.321  1.00117.92           N
ATOM   3849  N   ASN A 485      -9.040  21.104  44.681  1.00129.29           N
ATOM   3850  CA  ASN A 485     -10.397  21.671  44.656  1.00125.86           C
ATOM   3851  C   ASN A 485     -11.097  21.532  46.008  1.00121.76           C
ATOM   3852  O   ASN A 485     -12.235  21.058  46.102  1.00114.29           O
ATOM   3853  CB  ASN A 485     -11.229  21.050  43.539  1.00120.13           C
ATOM   3854  CG  ASN A 485     -12.326  21.988  43.046  1.00112.12           C
ATOM   3855  OD1 ASN A 485     -12.111  22.770  42.124  1.00104.61           O
ATOM   3856  ND2 ASN A 485     -13.502  21.915  43.662  1.00114.64           N
ATOM   3857  N   ASN A 486     -10.394  21.951  47.070  1.00116.60           N
ATOM   3858  CA  ASN A 486     -10.956  22.125  48.418  1.00103.86           C
ATOM   3859  C   ASN A 486     -11.687  20.889  48.932  1.00107.11           C
ATOM   3860  O   ASN A 486     -12.565  21.000  49.790  1.00101.16           O
ATOM   3861  CB  ASN A 486     -11.897  23.325  48.465  1.00106.74           C
ATOM   3862  CG  ASN A 486     -11.285  24.560  47.866  1.00121.64           C
ATOM   3863  OD1 ASN A 486     -10.077  24.616  47.636  1.00125.99           O
ATOM   3864  ND2 ASN A 486     -12.114  25.566  47.605  1.00120.88           N
ATOM   3865  N   SER A 487     -11.346  19.702  48.434  1.00106.73           N
ATOM   3866  CA  SER A 487     -12.083  18.517  48.842  1.00 99.34           C
ATOM   3867  C   SER A 487     -11.468  17.901  50.090  1.00 94.11           C
ATOM   3868  O   SER A 487     -10.263  18.011  50.340  1.00 90.53           O
ATOM   3869  CB  SER A 487     -12.148  17.474  47.720  1.00110.78           C
ATOM   3870  OG  SER A 487     -13.275  16.614  47.881  1.00107.47           O
ATOM   3871  N   THR A 488     -12.341  17.267  50.878  1.00 91.68           N
ATOM   3872  CA  THR A 488     -12.053  16.505  52.089  1.00 90.47           C
ATOM   3873  C   THR A 488     -10.649  15.918  52.175  1.00 92.07           C
ATOM   3874  O   THR A 488     -10.183  15.288  51.220  1.00 85.22           O
ATOM   3875  CB  THR A 488     -13.067  15.370  52.192  1.00 88.88           C
ATOM   3876  OG1 THR A 488     -14.390  15.907  52.052  1.00 85.31           O
ATOM   3877  CG2 THR A 488     -12.926  14.631  53.522  1.00 93.49           C
ATOM   3878  N   SER A 489      -9.968  16.110  53.306  1.00 85.81           N
ATOM   3879  CA  SER A 489      -8.718  15.397  53.521  1.00 86.17           C
ATOM   3880  C   SER A 489      -9.009  14.005  54.035  1.00 95.93           C
ATOM   3881  O   SER A 489      -9.976  13.797  54.777  1.00 99.88           O
ATOM   3882  CB  SER A 489      -7.799  16.098  54.517  1.00 91.13           C
ATOM   3883  OG  SER A 489      -6.610  15.319  54.688  1.00 65.61           O
ATOM   3884  N   TRP A 490      -8.134  13.059  53.660  1.00 92.26           N
ATOM   3885  CA  TRP A 490      -8.330  11.651  53.959  1.00 73.39           C
ATOM   3886  C   TRP A 490      -7.451  11.266  55.144  1.00 71.70           C
ATOM   3887  O   TRP A 490      -6.226  11.156  54.980  1.00 77.30           O
ATOM   3888  CB  TRP A 490      -7.985  10.807  52.733  1.00 82.96           C
ATOM   3889  CG  TRP A 490      -8.425   9.368  52.832  1.00 82.32           C
```

ATOM	3890	CD1	TRP	A	490	-8.464	8.592	53.954	1.00	78.07	C
ATOM	3891	CD2	TRP	A	490	-8.864	8.530	51.760	1.00	82.19	C
ATOM	3892	NE1	TRP	A	490	-8.932	7.344	53.666	1.00	71.19	N
ATOM	3893	CE2	TRP	A	490	-9.171	7.270	52.318	1.00	78.92	C
ATOM	3894	CE3	TRP	A	490	-9.052	8.728	50.379	1.00	85.35	C
ATOM	3895	CZ2	TRP	A	490	-9.632	6.216	51.549	1.00	70.08	C
ATOM	3896	CZ3	TRP	A	490	-9.506	7.686	49.618	1.00	76.30	C
ATOM	3897	CH2	TRP	A	490	-9.796	6.444	50.201	1.00	80.50	C
ATOM	3898	N	PRO	A	491	-8.016	11.055	56.335	1.00	68.53	N
ATOM	3899	CA	PRO	A	491	-7.218	10.612	57.492	1.00	61.62	C
ATOM	3900	C	PRO	A	491	-6.614	9.224	57.323	1.00	66.31	C
ATOM	3901	O	PRO	A	491	-7.072	8.400	56.530	1.00	84.00	O
ATOM	3902	CB	PRO	A	491	-8.241	10.601	58.634	1.00	71.74	C
ATOM	3903	CG	PRO	A	491	-9.588	10.553	57.949	1.00	73.93	C
ATOM	3904	CD	PRO	A	491	-9.416	11.331	56.695	1.00	69.56	C
ATOM	3905	N	VAL	A	492	-5.595	8.952	58.120	1.00	60.45	N
ATOM	3906	CA	VAL	A	492	-5.053	7.600	58.230	1.00	72.99	C
ATOM	3907	C	VAL	A	492	-5.933	6.764	59.162	1.00	80.70	C
ATOM	3908	O	VAL	A	492	-6.337	7.221	60.237	1.00	84.97	O
ATOM	3909	CB	VAL	A	492	-3.591	7.659	58.721	1.00	73.76	C
ATOM	3910	CG1	VAL	A	492	-3.187	6.389	59.464	1.00	74.80	C
ATOM	3911	CG2	VAL	A	492	-2.651	7.907	57.558	1.00	68.31	C
ATOM	3912	N	PHE	A	493	-6.247	5.531	58.753	1.00	80.41	N
ATOM	3913	CA	PHE	A	493	-6.946	4.583	59.625	1.00	86.97	C
ATOM	3914	C	PHE	A	493	-5.960	3.962	60.616	1.00	80.90	C
ATOM	3915	O	PHE	A	493	-5.024	3.253	60.219	1.00	73.00	O
ATOM	3916	CB	PHE	A	493	-7.632	3.492	58.796	1.00	86.66	C
ATOM	3917	CG	PHE	A	493	-8.422	2.457	59.612	1.00	77.36	C
ATOM	3918	CD1	PHE	A	493	-7.780	1.369	60.204	1.00	68.23	C
ATOM	3919	CD2	PHE	A	493	-9.814	2.545	59.715	1.00	65.80	C
ATOM	3920	CE1	PHE	A	493	-8.503	0.419	60.907	1.00	71.82	C
ATOM	3921	CE2	PHE	A	493	-10.547	1.599	60.409	1.00	57.19	C
ATOM	3922	CZ	PHE	A	493	-9.892	0.533	61.011	1.00	70.17	C
ATOM	3923	N	LYS	A	494	-6.173	4.229	61.903	1.00	86.95	N
ATOM	3924	CA	LYS	A	494	-5.419	3.613	62.987	1.00	90.11	C
ATOM	3925	C	LYS	A	494	-6.370	2.849	63.903	1.00	80.69	C
ATOM	3926	O	LYS	A	494	-7.578	3.107	63.929	1.00	76.44	O
ATOM	3927	CB	LYS	A	494	-4.631	4.669	63.802	1.00	83.05	C
ATOM	3928	CG	LYS	A	494	-3.197	4.892	63.333	1.00	78.09	C
ATOM	3929	CD	LYS	A	494	-2.249	3.879	63.970	1.00	89.43	C
ATOM	3930	CE	LYS	A	494	-1.093	3.503	63.052	1.00	83.80	C
ATOM	3931	NZ	LYS	A	494	-0.518	4.665	62.314	1.00103.16		N
ATOM	3932	N	SER	A	495	-5.824	1.885	64.638	1.00	80.21	N
ATOM	3933	CA	SER	A	495	-6.566	1.332	65.763	1.00	87.90	C

```
ATOM   3934  C   SER A 495      -6.814   2.438  66.784  1.00 99.28           C
ATOM   3935  O   SER A 495      -6.025   3.383  66.897  1.00108.57           O
ATOM   3936  CB  SER A 495      -5.792   0.186  66.410  1.00 80.43           C
ATOM   3937  OG  SER A 495      -5.795  -0.955  65.588  1.00 79.53           O
ATOM   3938  N   THR A 496      -7.909   2.293  67.541  1.00 84.08           N
ATOM   3939  CA  THR A 496      -8.512   3.294  68.431  1.00 87.12           C
ATOM   3940  C   THR A 496      -9.464   4.177  67.634  1.00 79.29           C
ATOM   3941  O   THR A 496     -10.667   3.909  67.598  1.00 80.15           O
ATOM   3942  CB  THR A 496      -7.503   4.194  69.179  1.00 84.65           C
ATOM   3943  OG1 THR A 496      -6.737   4.959  68.241  1.00 93.16           O
ATOM   3944  CG2 THR A 496      -6.555   3.382  70.064  1.00 83.49           C
ATOM   3945  N   GLU A 497      -8.942   5.229  66.995  1.00 86.28           N
ATOM   3946  CA  GLU A 497      -9.808   6.214  66.347  1.00 82.46           C
ATOM   3947  C   GLU A 497     -10.623   5.594  65.216  1.00 81.09           C
ATOM   3948  O   GLU A 497     -11.835   5.819  65.125  1.00 77.01           O
ATOM   3949  CB  GLU A 497      -8.974   7.393  65.835  1.00 90.56           C
ATOM   3950  CG  GLU A 497      -8.818   8.536  66.832  1.00 89.04           C
ATOM   3951  CD  GLU A 497      -9.925   9.590  66.692  1.00105.35           C
ATOM   3952  OE1 GLU A 497     -10.817   9.407  65.836  1.00115.77           O
ATOM   3953  OE2 GLU A 497      -9.916  10.593  67.439  1.00 97.90           O
ATOM   3954  N   GLN A 498      -9.964   4.827  64.335  1.00 85.86           N
ATOM   3955  CA  GLN A 498     -10.598   4.055  63.258  1.00 73.45           C
ATOM   3956  C   GLN A 498     -11.415   4.957  62.331  1.00 77.13           C
ATOM   3957  O   GLN A 498     -12.632   4.820  62.192  1.00 72.70           O
ATOM   3958  CB  GLN A 498     -11.465   2.923  63.825  1.00 77.15           C
ATOM   3959  CG  GLN A 498     -10.688   1.742  64.426  1.00 79.08           C
ATOM   3960  CD  GLN A 498     -11.592   0.831  65.240  1.00 86.58           C
ATOM   3961  OE1 GLN A 498     -12.814   1.001  65.245  1.00 89.56           O
ATOM   3962  NE2 GLN A 498     -10.998  -0.120  65.952  1.00 84.27           N
ATOM   3963  N   LYS A 499     -10.717   5.886  61.683  1.00 79.72           N
ATOM   3964  CA  LYS A 499     -11.382   6.850  60.816  1.00 79.19           C
ATOM   3965  C   LYS A 499     -11.435   6.363  59.364  1.00 74.29           C
ATOM   3966  O   LYS A 499     -10.466   5.801  58.832  1.00 67.51           O
ATOM   3967  CB  LYS A 499     -10.713   8.233  60.922  1.00 83.66           C
ATOM   3968  CG  LYS A 499     -11.192   9.046  62.170  1.00 89.85           C
ATOM   3969  CD  LYS A 499     -10.246  10.148  62.700  1.00 87.34           C
ATOM   3970  CE  LYS A 499     -10.870  11.570  62.615  1.00 76.99           C
ATOM   3971  NZ  LYS A 499     -12.359  11.679  62.770  1.00 73.68           N
ATOM   3972  N   TYR A 500     -12.594   6.566  58.736  1.00 69.47           N
ATOM   3973  CA  TYR A 500     -12.805   6.225  57.337  1.00 70.82           C
ATOM   3974  C   TYR A 500     -13.478   7.370  56.587  1.00 72.89           C
ATOM   3975  O   TYR A 500     -14.298   8.111  57.146  1.00 62.33           O
ATOM   3976  CB  TYR A 500     -13.655   4.960  57.192  1.00 66.73           C
ATOM   3977  CG  TYR A 500     -15.083   5.045  57.697  1.00 59.05           C
```

ATOM	3978	CD1	TYR	A	500	-15.392	4.705	58.997	1.00	62.09	C
ATOM	3979	CD2	TYR	A	500	-16.123	5.410	56.853	1.00	63.36	C
ATOM	3980	CE1	TYR	A	500	-16.688	4.757	59.456	1.00	67.71	C
ATOM	3981	CE2	TYR	A	500	-17.426	5.460	57.303	1.00	58.06	C
ATOM	3982	CZ	TYR	A	500	-17.703	5.123	58.608	1.00	66.16	C
ATOM	3983	OH	TYR	A	500	-18.994	5.172	59.094	1.00	70.54	O
ATOM	3984	N	LEU	A	501	-13.143	7.489	55.301	1.00	64.94	N
ATOM	3985	CA	LEU	A	501	-13.714	8.518	54.442	1.00	73.48	C
ATOM	3986	C	LEU	A	501	-14.823	7.906	53.607	1.00	67.84	C
ATOM	3987	O	LEU	A	501	-14.665	6.804	53.077	1.00	82.81	O
ATOM	3988	CB	LEU	A	501	-12.658	9.137	53.526	1.00	82.82	C
ATOM	3989	CG	LEU	A	501	-13.195	9.798	52.240	1.00	84.56	C
ATOM	3990	CD1	LEU	A	501	-13.984	11.068	52.579	1.00	87.56	C
ATOM	3991	CD2	LEU	A	501	-12.066	10.139	51.297	1.00	79.66	C
ATOM	3992	N	THR	A	502	-15.933	8.612	53.481	1.00	57.80	N
ATOM	3993	CA	THR	A	502	-17.004	8.067	52.663	1.00	75.16	C
ATOM	3994	C	THR	A	502	-16.938	8.642	51.246	1.00	76.49	C
ATOM	3995	O	THR	A	502	-16.276	9.652	50.991	1.00	77.05	O
ATOM	3996	CB	THR	A	502	-18.361	8.340	53.300	1.00	69.12	C
ATOM	3997	OG1	THR	A	502	-18.521	9.747	53.458	1.00	72.70	O
ATOM	3998	CG2	THR	A	502	-18.465	7.671	54.658	1.00	67.21	C
ATOM	3999	N	LEU	A	503	-17.621	7.970	50.314	1.00	69.29	N
ATOM	4000	CA	LEU	A	503	-17.560	8.313	48.894	1.00	62.66	C
ATOM	4001	C	LEU	A	503	-18.962	8.377	48.304	1.00	69.25	C
ATOM	4002	O	LEU	A	503	-19.652	7.356	48.249	1.00	68.62	O
ATOM	4003	CB	LEU	A	503	-16.705	7.299	48.127	1.00	48.16	C
ATOM	4004	CG	LEU	A	503	-15.220	7.349	48.489	1.00	56.05	C
ATOM	4005	CD1	LEU	A	503	-14.369	6.287	47.798	1.00	51.46	C
ATOM	4006	CD2	LEU	A	503	-14.695	8.728	48.164	1.00	62.46	C
ATOM	4007	N	ASN	A	504	-19.373	9.560	47.834	1.00	74.02	N
ATOM	4008	CA	ASN	A	504	-20.684	9.716	47.201	1.00	80.65	C
ATOM	4009	C	ASN	A	504	-20.716	11.014	46.406	1.00	79.71	C
ATOM	4010	O	ASN	A	504	-19.748	11.781	46.395	1.00	85.13	O
ATOM	4011	CB	ASN	A	504	-21.795	9.768	48.230	1.00	76.44	C
ATOM	4012	CG	ASN	A	504	-21.606	10.914	49.141	1.00	82.27	C
ATOM	4013	OD1	ASN	A	504	-21.841	12.050	48.768	1.00	89.43	O
ATOM	4014	ND2	ASN	A	504	-21.076	10.649	50.304	1.00	89.80	N
ATOM	4015	N	THR	A	505	-21.894	11.299	45.824	1.00	79.13	N
ATOM	4016	CA	THR	A	505	-22.087	12.490	44.996	1.00	78.49	C
ATOM	4017	C	THR	A	505	-22.159	13.778	45.818	1.00	85.65	C
ATOM	4018	O	THR	A	505	-21.663	14.820	45.375	1.00	90.68	O
ATOM	4019	CB	THR	A	505	-23.367	12.353	44.153	1.00	72.53	C
ATOM	4020	OG1	THR	A	505	-24.511	12.208	45.003	1.00	75.12	O
ATOM	4021	CG2	THR	A	505	-23.278	11.153	43.235	1.00	67.41	C

```
ATOM   4022  N   GLU A 506     -22.742  13.726  47.016  1.00 88.89           N
ATOM   4023  CA  GLU A 506     -23.073  14.917  47.788  1.00 87.21           C
ATOM   4024  C   GLU A 506     -21.841  15.449  48.530  1.00 93.69           C
ATOM   4025  O   GLU A 506     -21.056  16.185  47.921  1.00 98.35           O
ATOM   4026  CB  GLU A 506     -24.322  14.589  48.631  1.00 80.85           C
ATOM   4027  CG  GLU A 506     -24.253  13.511  49.732  1.00 97.05           C
ATOM   4028  CD  GLU A 506     -23.567  13.920  51.034  1.00100.09           C
ATOM   4029  OE1 GLU A 506     -23.538  15.137  51.331  1.00 96.03           O
ATOM   4030  OE2 GLU A 506     -23.011  13.046  51.768  1.00107.05           O
ATOM   4031  N   SER A 507     -21.624  15.128  49.799  1.00 77.70           N
ATOM   4032  CA  SER A 507     -20.397  15.511  50.482  1.00 97.57           C
ATOM   4033  C   SER A 507     -19.646  14.239  50.826  1.00 90.55           C
ATOM   4034  O   SER A 507     -20.246  13.176  50.994  1.00 87.84           O
ATOM   4035  CB  SER A 507     -20.644  16.308  51.788  1.00104.75           C
ATOM   4036  OG  SER A 507     -21.536  15.604  52.645  1.00103.67           O
ATOM   4037  N   THR A 508     -18.338  14.363  50.965  1.00 92.63           N
ATOM   4038  CA  THR A 508     -17.515  13.264  51.435  1.00 83.65           C
ATOM   4039  C   THR A 508     -17.140  13.534  52.893  1.00 78.32           C
ATOM   4040  O   THR A 508     -16.408  14.484  53.176  1.00 81.83           O
ATOM   4041  CB  THR A 508     -16.303  13.109  50.511  1.00 86.32           C
ATOM   4042  OG1 THR A 508     -15.540  14.318  50.494  1.00 97.09           O
ATOM   4043  CG2 THR A 508     -16.773  12.839  49.068  1.00 76.40           C
ATOM   4044  N   ARG A 509     -17.697  12.745  53.820  1.00 78.55           N
ATOM   4045  CA  ARG A 509     -17.479  12.934  55.250  1.00 76.21           C
ATOM   4046  C   ARG A 509     -16.341  12.074  55.770  1.00 68.89           C
ATOM   4047  O   ARG A 509     -15.931  11.091  55.154  1.00 77.86           O
ATOM   4048  CB  ARG A 509     -18.730  12.613  56.082  1.00 78.10           C
ATOM   4049  CG  ARG A 509     -20.011  13.306  55.674  1.00 87.14           C
ATOM   4050  CD  ARG A 509     -21.091  12.305  55.410  1.00 81.26           C
ATOM   4051  NE  ARG A 509     -20.738  11.542  54.223  0.26 87.83           N
ATOM   4052  CZ  ARG A 509     -21.299  10.390  53.880  0.58 93.61           C
ATOM   4053  NH1 ARG A 509     -22.232   9.828  54.644  1.00 93.69           N
ATOM   4054  NH2 ARG A 509     -20.887   9.797  52.780  1.00 87.80           N
ATOM   4055  N   ILE A 510     -15.863  12.453  56.948  1.00 62.67           N
ATOM   4056  CA  ILE A 510     -14.961  11.642  57.744  1.00 64.46           C
ATOM   4057  C   ILE A 510     -15.772  11.050  58.887  1.00 74.24           C
ATOM   4058  O   ILE A 510     -16.500  11.777  59.580  1.00 67.03           O
ATOM   4059  CB  ILE A 510     -13.783  12.471  58.274  1.00 67.74           C
ATOM   4060  CG1 ILE A 510     -13.131  13.276  57.148  1.00 73.68           C
ATOM   4061  CG2 ILE A 510     -12.788  11.573  58.970  1.00 72.48           C
ATOM   4062  CD1 ILE A 510     -13.619  14.737  57.067  1.00 76.32           C
ATOM   4063  N   MET A 511     -15.687   9.727  59.056  1.00 77.51           N
ATOM   4064  CA  MET A 511     -16.399   9.014  60.111  1.00 73.57           C
ATOM   4065  C   MET A 511     -15.480   7.994  60.781  1.00 75.17           C
```

ATOM	4066	O	MET	A	511	-14.416	7.632	60.268	1.00	76.39	O
ATOM	4067	CB	MET	A	511	-17.650	8.301	59.579	1.00	63.95	C
ATOM	4068	CG	MET	A	511	-18.404	9.058	58.502	1.00	69.92	C
ATOM	4069	SD	MET	A	511	-19.756	10.044	59.153	1.00	91.32	S
ATOM	4070	CE	MET	A	511	-21.057	8.816	59.229	1.00	77.20	C
ATOM	4071	N	THR	A	512	-15.928	7.526	61.943	1.00	74.68	N
ATOM	4072	CA	THR	A	512	-15.271	6.510	62.751	1.00	76.98	C
ATOM	4073	C	THR	A	512	-16.205	5.310	62.912	1.00	80.71	C
ATOM	4074	O	THR	A	512	-17.396	5.399	62.602	1.00	88.47	O
ATOM	4075	CB	THR	A	512	-14.931	7.084	64.124	1.00	72.89	C
ATOM	4076	OG1	THR	A	512	-16.100	7.738	64.630	1.00	85.34	O
ATOM	4077	CG2	THR	A	512	-13.823	8.095	64.003	1.00	64.41	C
ATOM	4078	N	LYS	A	513	-15.673	4.177	63.403	1.00	69.23	N
ATOM	4079	CA	LYS	A	513	-16.525	3.108	63.933	1.00	62.80	C
ATOM	4080	C	LYS	A	513	-17.366	2.464	62.820	1.00	71.74	C
ATOM	4081	O	LYS	A	513	-18.576	2.280	62.953	1.00	81.69	O
ATOM	4082	CB	LYS	A	513	-17.401	3.712	65.072	1.00	93.52	C
ATOM	4083	CG	LYS	A	513	-18.923	3.309	65.287	1.00	89.90	C
ATOM	4084	CD	LYS	A	513	-19.630	3.897	66.534	1.00	98.77	C
ATOM	4085	CE	LYS	A	513	-19.469	5.451	66.652	1.00	94.12	C
ATOM	4086	NZ	LYS	A	513	-20.316	6.292	65.717	1.00	89.46	N
ATOM	4087	N	LEU	A	514	-16.749	2.135	61.682	1.00	65.91	N
ATOM	4088	CA	LEU	A	514	-17.623	1.611	60.634	1.00	67.34	C
ATOM	4089	C	LEU	A	514	-18.265	0.338	61.170	1.00	64.08	C
ATOM	4090	O	LEU	A	514	-17.567	-0.562	61.650	1.00	55.19	O
ATOM	4091	CB	LEU	A	514	-16.894	1.366	59.308	1.00	69.15	C
ATOM	4092	CG	LEU	A	514	-17.717	0.833	58.114	1.00	61.51	C
ATOM	4093	CD1	LEU	A	514	-19.111	1.422	58.063	1.00	63.50	C
ATOM	4094	CD2	LEU	A	514	-17.026	1.270	56.870	1.00	53.29	C
ATOM	4095	N	ARG	A	515	-19.600	0.349	61.211	1.00	63.87	N
ATOM	4096	CA	ARG	A	515	-20.438	-0.667	61.843	1.00	67.35	C
ATOM	4097	C	ARG	A	515	-19.747	-1.443	62.969	1.00	64.83	C
ATOM	4098	O	ARG	A	515	-19.476	-2.639	62.833	1.00	70.88	O
ATOM	4099	CB	ARG	A	515	-20.950	-1.607	60.758	1.00	73.37	C
ATOM	4100	CG	ARG	A	515	-21.583	-0.859	59.608	1.00	71.54	C
ATOM	4101	CD	ARG	A	515	-21.959	-1.774	58.458	1.00	70.10	C
ATOM	4102	NE	ARG	A	515	-22.933	-2.774	58.876	1.00	75.60	N
ATOM	4103	CZ	ARG	A	515	-24.250	-2.583	58.889	1.00	81.28	C
ATOM	4104	NH1	ARG	A	515	-25.058	-3.563	59.296	1.00	83.60	N
ATOM	4105	NH2	ARG	A	515	-24.759	-1.424	58.487	1.00	88.13	N
ATOM	4106	N	ALA	A	516	-19.467	-0.774	64.092	1.00	70.43	N
ATOM	4107	CA	ALA	A	516	-18.658	-1.390	65.140	1.00	64.07	C
ATOM	4108	C	ALA	A	516	-19.446	-2.423	65.937	1.00	62.34	C
ATOM	4109	O	ALA	A	516	-18.906	-3.493	66.255	1.00	62.98	O

ATOM	4110	CB	ALA	A	516	-18.083	-0.322	66.070	1.00	66.43	C
ATOM	4111	N	GLN	A	517	-20.715	-2.129	66.256	1.00	64.27	N
ATOM	4112	CA	GLN	A	517	-21.553	-3.072	67.000	1.00	70.68	C
ATOM	4113	C	GLN	A	517	-21.629	-4.413	66.289	1.00	70.98	C
ATOM	4114	O	GLN	A	517	-21.279	-5.462	66.857	1.00	55.33	O
ATOM	4115	CB	GLN	A	517	-22.970	-2.516	67.194	1.00	77.01	C
ATOM	4116	CG	GLN	A	517	-23.943	-3.547	67.802	1.00	82.68	C
ATOM	4117	CD	GLN	A	517	-25.355	-3.466	67.233	0.54	82.10	C
ATOM	4118	OE1	GLN	A	517	-25.793	-2.399	66.802	0.66	81.95	O
ATOM	4119	NE2	GLN	A	517	-26.085	-4.589	67.266	0.83	77.16	N
ATOM	4120	N	GLN	A	518	-22.100	-4.378	65.031	1.00	78.35	N
ATOM	4121	CA	GLN	A	518	-22.159	-5.573	64.195	1.00	68.23	C
ATOM	4122	C	GLN	A	518	-20.812	-6.275	64.138	1.00	63.95	C
ATOM	4123	O	GLN	A	518	-20.699	-7.443	64.529	1.00	57.68	O
ATOM	4124	CB	GLN	A	518	-22.627	-5.217	62.787	1.00	59.25	C
ATOM	4125	CG	GLN	A	518	-24.033	-4.664	62.725	1.00	69.25	C
ATOM	4126	CD	GLN	A	518	-24.063	-3.137	62.716	1.00	78.49	C
ATOM	4127	OE1	GLN	A	518	-23.074	-2.472	63.058	1.00	74.14	O
ATOM	4128	NE2	GLN	A	518	-25.208	-2.574	62.333	1.00	73.62	N
ATOM	4129	N	CYS	A	519	-19.772	-5.572	63.668	1.00	52.66	N
ATOM	4130	CA	CYS	A	519	-18.506	-6.249	63.399	1.00	63.76	C
ATOM	4131	C	CYS	A	519	-18.002	-6.996	64.610	1.00	63.27	C
ATOM	4132	O	CYS	A	519	-17.366	-8.048	64.484	1.00	68.37	O
ATOM	4133	CB	CYS	A	519	-17.456	-5.264	62.905	1.00	62.56	C
ATOM	4134	SG	CYS	A	519	-17.717	-5.007	61.179	1.00	70.10	S
ATOM	4135	N	ARG	A	520	-18.309	-6.497	65.792	1.00	70.98	N
ATOM	4136	CA	ARG	A	520	-17.674	-7.085	66.948	1.00	81.95	C
ATOM	4137	C	ARG	A	520	-18.455	-8.301	67.447	1.00	79.56	C
ATOM	4138	O	ARG	A	520	-17.899	-9.132	68.173	1.00	87.02	O
ATOM	4139	CB	ARG	A	520	-17.480	-6.029	68.025	1.00	82.28	C
ATOM	4140	CG	ARG	A	520	-17.713	-6.559	69.321	1.00	87.39	C
ATOM	4141	CD	ARG	A	520	-17.976	-5.532	70.340	1.00	94.99	C
ATOM	4142	NE	ARG	A	520	-18.487	-6.186	71.395	1.00	97.32	N
ATOM	4143	CZ	ARG	A	520	-17.858	-6.426	72.403	1.00	95.00	C
ATOM	4144	NH1	ARG	A	520	-18.361	-7.074	73.289	1.00	85.02	N
ATOM	4145	NH2	ARG	A	520	-16.706	-6.041	72.489	1.00	108.36	N
ATOM	4146	N	PHE	A	521	-19.730	-8.433	67.053	1.00	75.73	N
ATOM	4147	CA	PHE	A	521	-20.394	-9.736	67.100	1.00	70.95	C
ATOM	4148	C	PHE	A	521	-19.640	-10.746	66.237	1.00	72.65	C
ATOM	4149	O	PHE	A	521	-19.237	-11.815	66.710	1.00	80.67	O
ATOM	4150	CB	PHE	A	521	-21.854	-9.590	66.639	1.00	73.77	C
ATOM	4151	CG	PHE	A	521	-22.527	-10.896	66.255	1.00	71.91	C
ATOM	4152	CD1	PHE	A	521	-23.262	-11.612	67.182	1.00	69.57	C
ATOM	4153	CD2	PHE	A	521	-22.454	-11.392	64.952	1.00	78.12	C

```
ATOM   4154  CE1 PHE A 521     -23.892 -12.809  66.840  1.00 72.19           C
ATOM   4155  CE2 PHE A 521     -23.079 -12.601  64.599  1.00 78.62           C
ATOM   4156  CZ  PHE A 521     -23.802 -13.307  65.553  1.00 73.94           C
ATOM   4157  N   TRP A 522     -19.430 -10.409  64.959  1.00 75.86           N
ATOM   4158  CA  TRP A 522     -18.795 -11.334  64.027  1.00 73.39           C
ATOM   4159  C   TRP A 522     -17.331 -11.573  64.365  1.00 78.10           C
ATOM   4160  O   TRP A 522     -16.803 -12.645  64.058  1.00 79.15           O
ATOM   4161  CB  TRP A 522     -18.903 -10.814  62.590  1.00 70.10           C
ATOM   4162  CG  TRP A 522     -20.310 -10.746  62.038  1.00 70.91           C
ATOM   4163  CD1 TRP A 522     -21.008  -9.615  61.700  1.00 64.14           C
ATOM   4164  CD2 TRP A 522     -21.186 -11.855  61.759  1.00 70.49           C
ATOM   4165  NE1 TRP A 522     -22.262  -9.956  61.236  1.00 59.29           N
ATOM   4166  CE2 TRP A 522     -22.395 -11.320  61.260  1.00 57.52           C
ATOM   4167  CE3 TRP A 522     -21.066 -13.245  61.887  1.00 70.36           C
ATOM   4168  CZ2 TRP A 522     -23.475 -12.126  60.894  1.00 60.57           C
ATOM   4169  CZ3 TRP A 522     -22.142 -14.046  61.515  1.00 64.33           C
ATOM   4170  CH2 TRP A 522     -23.325 -13.484  61.025  1.00 67.68           C
ATOM   4171  N   THR A 523     -16.655 -10.594  64.969  1.00 77.66           N
ATOM   4172  CA  THR A 523     -15.211 -10.704  65.130  1.00 83.67           C
ATOM   4173  C   THR A 523     -14.825 -11.331  66.459  1.00 88.59           C
ATOM   4174  O   THR A 523     -13.801 -12.017  66.536  1.00 94.00           O
ATOM   4175  CB  THR A 523     -14.537  -9.325  64.990  1.00 82.92           C
ATOM   4176  OG1 THR A 523     -13.137  -9.483  64.717  1.00 88.66           O
ATOM   4177  CG2 THR A 523     -14.668  -8.516  66.267  1.00 76.14           C
ATOM   4178  N   SER A 524     -15.626 -11.136  67.504  1.00 90.65           N
ATOM   4179  CA  SER A 524     -15.206 -11.527  68.842  1.00 91.73           C
ATOM   4180  C   SER A 524     -15.858 -12.807  69.313  1.00 97.47           C
ATOM   4181  O   SER A 524     -15.230 -13.575  70.051  1.00109.61           O
ATOM   4182  CB  SER A 524     -15.507 -10.425  69.864  1.00 92.94           C
ATOM   4183  OG  SER A 524     -16.881 -10.066  69.880  1.00 94.50           O
ATOM   4184  N   PHE A 525     -17.106 -13.055  68.916  1.00100.25           N
ATOM   4185  CA  PHE A 525     -17.892 -14.119  69.525  1.00 98.11           C
ATOM   4186  C   PHE A 525     -18.303 -15.200  68.534  1.00 93.59           C
ATOM   4187  O   PHE A 525     -18.085 -16.390  68.805  1.00 94.79           O
ATOM   4188  CB  PHE A 525     -19.115 -13.511  70.229  1.00 78.00           C
ATOM   4189  CG  PHE A 525     -20.279 -14.457  70.364  1.00101.44           C
ATOM   4190  CD1 PHE A 525     -20.108 -15.716  70.908  1.00115.79           C
ATOM   4191  CD2 PHE A 525     -21.559 -14.052  70.056  1.00106.20           C
ATOM   4192  CE1 PHE A 525     -21.168 -16.581  71.045  1.00125.27           C
ATOM   4193  CE2 PHE A 525     -22.630 -14.916  70.219  1.00112.89           C
ATOM   4194  CZ  PHE A 525     -22.435 -16.174  70.711  1.00110.31           C
ATOM   4195  N   PHE A 526     -18.900 -14.833  67.400  1.00 94.81           N
ATOM   4196  CA  PHE A 526     -19.205 -15.857  66.403  1.00 91.43           C
ATOM   4197  C   PHE A 526     -18.044 -16.804  66.121  1.00 86.41           C
```

```
ATOM   4198  O   PHE A 526     -18.313 -17.995  65.882  1.00 89.38           O
ATOM   4199  CB  PHE A 526     -19.738 -15.225  65.108  1.00 78.86           C
ATOM   4200  CG  PHE A 526     -20.357 -16.231  64.127  1.00 65.03           C
ATOM   4201  CD1 PHE A 526     -21.690 -16.591  64.229  1.00 64.83           C
ATOM   4202  CD2 PHE A 526     -19.629 -16.733  63.052  1.00 58.56           C
ATOM   4203  CE1 PHE A 526     -22.276 -17.464  63.315  1.00 66.81           C
ATOM   4204  CE2 PHE A 526     -20.206 -17.602  62.140  1.00 61.76           C
ATOM   4205  CZ  PHE A 526     -21.534 -17.966  62.268  1.00 62.29           C
ATOM   4206  N   PRO A 527     -16.773 -16.379  66.140  1.00 88.70           N
ATOM   4207  CA  PRO A 527     -15.698 -17.348  65.917  1.00100.05           C
ATOM   4208  C   PRO A 527     -15.741 -18.557  66.836  1.00105.34           C
ATOM   4209  O   PRO A 527     -15.053 -19.540  66.539  1.00117.65           O
ATOM   4210  CB  PRO A 527     -14.419 -16.512  66.142  1.00 96.77           C
ATOM   4211  CG  PRO A 527     -14.884 -15.049  66.311  1.00 96.28           C
ATOM   4212  CD  PRO A 527     -16.282 -15.017  65.783  1.00 93.51           C
ATOM   4213  N   LYS A 528     -16.565 -18.567  67.887  1.00 97.62           N
ATOM   4214  CA  LYS A 528     -16.592 -19.701  68.813  1.00110.71           C
ATOM   4215  C   LYS A 528     -17.608 -20.775  68.432  1.00128.46           C
ATOM   4216  O   LYS A 528     -17.309 -21.970  68.545  1.00135.94           O
ATOM   4217  CB  LYS A 528     -16.893 -19.203  70.225  1.00 90.93           C
ATOM   4218  CG  LYS A 528     -15.887 -18.169  70.685  1.00110.06           C
ATOM   4219  CD  LYS A 528     -15.955 -17.891  72.179  1.00 99.22           C
ATOM   4220  CE  LYS A 528     -16.633 -16.560  72.460  1.00 80.76           C
ATOM   4221  NZ  LYS A 528     -15.675 -15.419  72.438  1.00 95.11           N
ATOM   4222  N   VAL A 529     -18.806 -20.372  68.012  1.00124.24           N
ATOM   4223  CA  VAL A 529     -19.899 -21.291  67.694  1.00132.91           C
ATOM   4224  C   VAL A 529     -19.487 -22.447  66.760  1.00140.68           C
ATOM   4225  O   VAL A 529     -18.587 -22.333  65.913  1.00142.38           O
ATOM   4226  CB  VAL A 529     -21.072 -20.496  67.093  1.00128.51           C
ATOM   4227  CG1 VAL A 529     -21.350 -19.268  67.957  1.00104.89           C
ATOM   4228  CG2 VAL A 529     -20.774 -20.093  65.632  1.00117.58           C
TER    4229      VAL A 529
```

Table S1. Free energy of binding, ΔG_{bind}, estimated by molecular docking of compounds **14**, **15**, **22** and **23** into the active site of AChE (4EY7.pdb), along with the number of conformational clusters, distribution of conformations, and docking results of the reference compound donepezil and galantamine.

Ligand	ΔG_{bind}/kcal mol^{-1}		Number of distinctive conformational clusters	Distribution of conformations within clusters with $n > 1$ (n = cluster population)
	lowest	highest		
**14**	−9.50	−9.33	1	25
**15**	−9.49	−9.21	1	25
**22**	−9.32	−8.53	5	7, 2, 4, 7, 5
**23**	−9.46	−8.70	4	8, 3, 10, 4
donepezil	−11.37	−10.75	4	22
galantamine	−10.11	−10.10	1	25

Table S2. Free energies of binding, ΔG_{bind}, obtained by molecular docking of listed molecules into the active site of BChE (7AIY.pdb), along with the number of conformational clusters, distribution of conformations, and docking results of the reference compounds donepezil and galantamine.

Ligand	ΔG_{bind}/kcal mol^{-1}		Number of distinctive conformational clusters	Distribution of conformations within clusters with $n > 1$ (n = cluster population)
	lowest	highest		
**14**	−8.63	−8.52	1	25
**15**	−8.54	−8.40	1	24

**22**	−7.79	−7.45	5	5, 2, 9, 3, 5
**23**	−7.89	−7.49	4	2, 8, 4, 9
donepezil	−9.77	−8.16	4	2, 5, 15, 3
galantamine	−7.32	−7.15	1	25

Table S3. The mutagenic potential of thienobenzo-triazoles **14–21** and triazolium salts **22–31** by Lhasa M7 evaluation (green square – negative, red square – positive, white square – no data available); grey highlight – negative, orange highlight – positive, white – strongly negative).

Structure	ICH M7 Class	Derek Prediction	Sarah Prediction	Overall In Silico
**14**	Class 3	− − − □	+ □ □ □	Positive
**15**	Class 3	− − − □	+ □ □ □	Positive
**16**	Class 3	− − − □	+ □ □ □	Positive
**17**	Class 3	− − − □	+ □ □ □	Positive
**18**	Inconclusive	− − − □	□ □ □ □	Negative
**19**	Class 3	− − − □	+ □ □ □	Positive
**20**	Class 3	− − − □	+ □ □ □	Positive
**21**	Class 3	− − − □	+ □ □ □	Positive
**22**	Inconclusive	− − □ □	□ □ □ □	Inconclusive
**23**	Inconclusive	− − □ □	□ □ □ □	Inconclusive
**24**	Inconclusive	− − □ □	□ □ □ □	Inconclusive
**25**	Inconclusive	− − □ □	□ □ □ □	Inconclusive
**26**	Inconclusive	− − □ □	□ □ □ □	Inconclusive
**27**	Inconclusive	− − □ □	□ □ □ □	Inconclusive
**28**	Inconclusive	− − □ □	□ □ □ □	Inconclusive
**29**	Inconclusive	− − □ □	□ □ □ □	Inconclusive
**30**	Inconclusive	− − □ □	□ □ □ □	Inconclusive
**31**	Inconclusive	− − □ □	□ □ □ □	Inconclusive
